# Total Syntheses of Nominal and Actual Prorocentin

**DOI:** 10.1021/jacs.2c12529

**Published:** 2023-01-18

**Authors:** Raphael J. Zachmann, Kenzo Yahata, Mira Holzheimer, Maxime Jarret, Cornelia Wirtz, Alois Fürstner

**Affiliations:** Max-Planck-Institut für Kohlenforschung, 45478 Mülheim/Ruhr, Germany

## Abstract

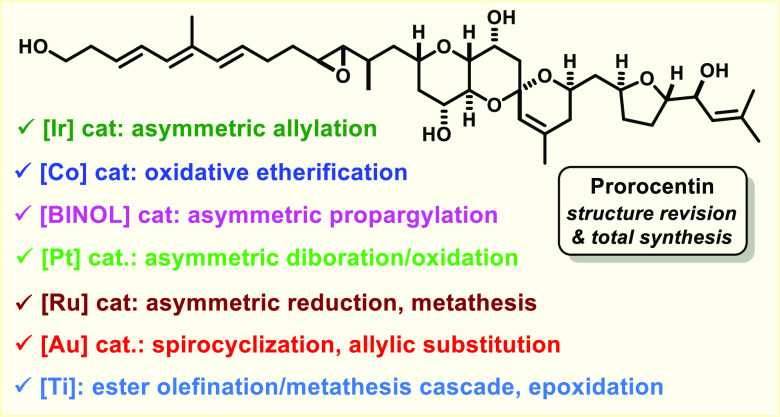

The dinoflagellate-derived polyether prorocentin is a co-metabolite of the archetypical serine/threonine phosphatase inhibitor okadaic acid. Whereas a structural relationship cannot be missed and a biosynthetic link was proposed, it is currently unknown whether there is any parallel in the bioactivity profile of these natural products. However, it was insinuated in the past that the structure assigned to prorocentin might need to be revised. Indeed, re-examination of the published spectra cast doubts as to the constitution of the fused/spirotricyclic BCD-ring system in the core. To clarify this issue, a flexible synthesis blueprint was devised that allowed us to obtain the originally proposed structure as well as the most plausible amended structure. The key to success was late-stage gold-catalyzed spirocyclization reactions that furnished the isomeric central segments with excellent selectivity. The lexicon of catalytic transformations used to make the required cyclization precursors comprised a titanium-mediated ester methylenation/metathesis cascade, a rare example of a gold-catalyzed allylic substitution, and chain extensions via organocatalytic asymmetric aldehyde propargylation. A wing sector to be attached to the isomeric cores was obtained by Krische allylation, followed by a superbly selective cobalt-catalyzed oxidative cyclization of the resulting di-unsaturated alcohol with the formation of a 2,5-*trans*-disubstituted tetrahydrofuran; the remaining terminal alkene was elaborated into an appropriate handle for fragment coupling by platinum-catalyzed asymmetric diboration/oxidation. The assembly of the different building blocks to the envisaged isomeric target compounds proved that the structure of prorocentin needs to be revised as disclosed herein.

## Introduction

**Scheme 1:**
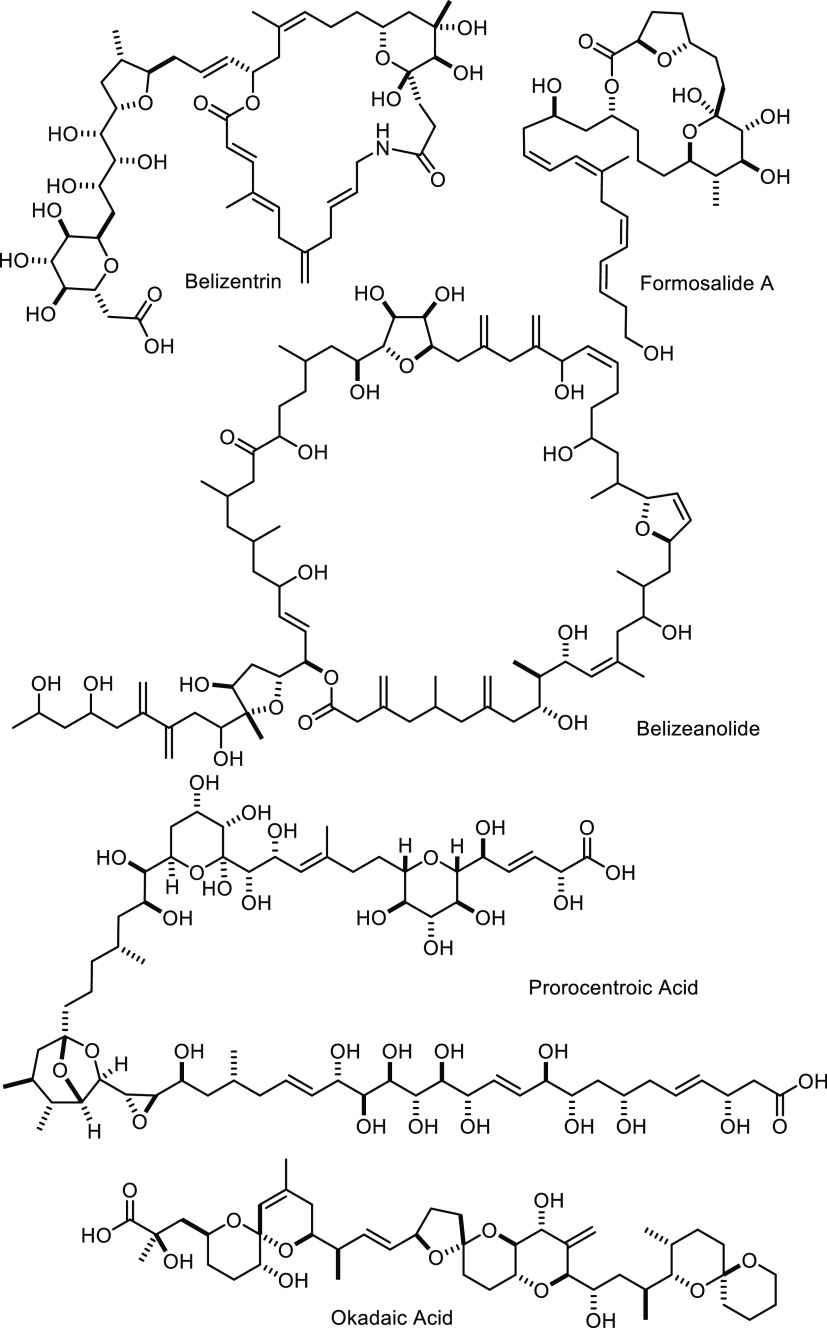
Selected Natural Products Derived from *P. lima*

Despite their rather simple morphology, many dinoflagellates comprise disproportionally large genomes that encode for secondary metabolites of stunning molecular intricacy.^1^ The members of the *Prorocentrum* genus are representative: as some of them cause severe ailment such as diarrhetic shellfish poisoning if they reach the human foodchain, their metabolomes were subject to intense scrutiny in the past. Belizentrin,^2,3^ belizeanolide,^4^ formosalides,^5,6^ prorocentroic acid,^7^ limaol,^8,9^ and okadaic acid^10,11^ are no more than a small selection that illustrates the diversity and complexity of natural products derived from these monocellular organisms (Scheme 1). Arguably, the most prominent among them is okadaic acid, which is an archetypical serine/threonine phosphatase inhibitor.^12,13^ As such, it became an indispensable tool for investigations into the role that these ubiquitous enzymes play in cellular signaling, cell cycle control, apoptosis, or tumor promotion. Moreover, okadaic acid exhibits cytotoxic, neurotoxic, genotoxic, immunotoxic, and potentially carcinogenic properties.^12,13^

The strain *P. lima* PL021117001 collected off the northern coast of Taiwan produces, in addition to okadaic acid, yet another secondary metabolite named prorocentin.^14^ Although the structural features and apparent constitutional “irregularities” found in both compounds suggest that they might share parts of the biosynthesis pathway, little more is known about the biological properties of prorocentin than its fairly modest cytotoxicity against two human cancer cell lines and the apparent lack of antimicrobial activity against *Staphylococcus aureus*.^14^

**Scheme 2:**
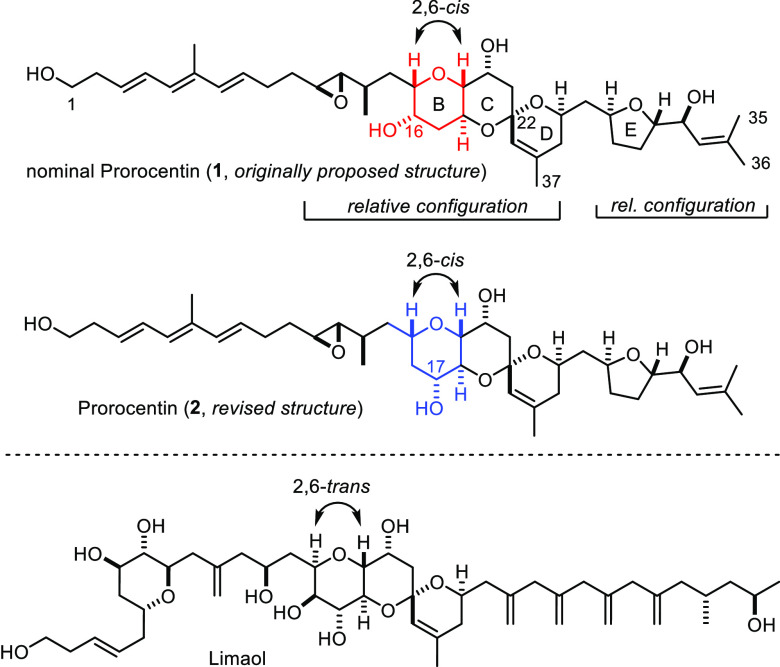
Structures of Prorocentin to be Considered. Comparison with Limaol

The proposed molecular structure of prorocentin (**1**) comprises a polyketide chain adorned with four pendant methyl substituents and 13 chiral centers (Scheme 2). An all-*trans* triene entity, two additional site-isolated olefins, four −OH groups, a *trans*-epoxide, a 6/6/6 *trans*-fused/spirocyclic triether core, and a 2,5-*trans*-configured tetrahydrofuran decorate the C39 (!) carbon framework. It is important to note that the isolation team has only elucidated the relative configuration of the tricyclic core and the tetrahydrofuran entity but could not determine the stereochemical relationship between these sectors.

Equally unknown is the absolute configuration of prorocentin. We surmised, however, that it would be antipodal to the configuration (arbitrarily) drawn by the isolation team.^14^ This expectation was based on the close resemblance to the C40 polyketide limaol derived from a different *P. lima* strain harvested in Korea,^8^ the absolute configuration of which was recently confirmed by total synthesis.^9^ What makes this extrapolation somewhat uncertain, however, is a subtle yet distinct stereochemical divergence between these apparent siblings: specifically, the fused B-ring of prorocentin features a 2,6-*cis* arrangement, whereas the analogous ring in limaol is 2,6-*trans*-configured.

For a compound of such prominent biosynthetic pedigree, structural intricacy, and exceedingly short supply,^15^ prorocentin received surprisingly little attention from the synthetic community in the past. Only a model study directed toward the C22–C37 segment was published, from which no firm conclusion as to the unknown stereochemical interrelation between the core and the side chain could be drawn.^16^ This very preliminary report, which appeared in the press in 2011, stands in striking contrast to a short symposium proceeding published by the same group already a year earlier, which insinuates that a total synthesis had been completed at that time.^17^ Moreover, a structure revision for prorocentin was proposed: as shown in **2**, the constitution of the B-ring was amended, although the reasons that led to this particular revision had not been outlined at all.^17^

Although certainly alerted by this preliminary report, the lack of any experimental data left us with no other choice than taking a fresh and unbiased approach. To this end, we started with a critical reassessment of the published nuclear magnetic resonance (NMR) data of the isolated natural product. Despite this exercise, we ultimately faced the need to make two different tricyclic core segments and join them to the appropriate side chains (potentially in both enantiomeric forms) in order to establish the correct constitution as well as the relative and absolute configuration of this challenging target compound. The successful completion of this ambitious project is reported below.

## Results and Discussion

### Reassessment of the Spectroscopic Data

**Scheme 3:**
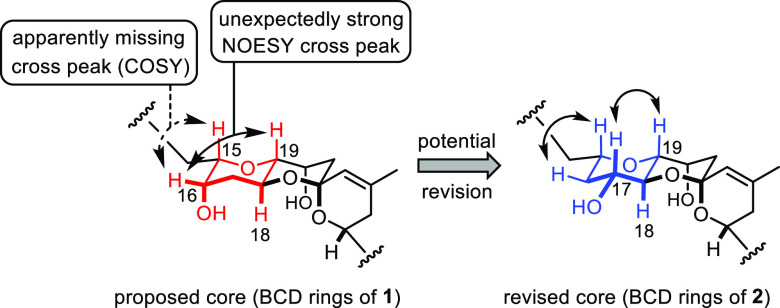
Key Spectroscopic Features Fitting Better to the Revised Core Structure Comprised in 2

A meticulous reevaluation of the published NMR spectra of prorocentin did indeed raise doubts concerning the proposed structure. Two data points were particularly alarming. First, the COSY spectrum contained in the Supporting Information of the isolation paper^14^ seems to lack the expected cross peak between H15 (δ_H_ = 3.55 ppm) and presumed H16 that supposedly resonates at δ_H_ = 3.80 ppm (Scheme 3). Rather, H15 seems to show a cross peak with a proton signal at δ_H_ = 1.41 ppm (for edited spectra, see the Supporting Information). This spectral fingerprint suggests that the branching site on the core (C15) is flanked by a −CH_2_– rather than a −CHOH group.

Certain NOESY data were also deemed suspicious. Specifically, the published spectrum shows a cross peak between the signal assigned to the presumably equatorially disposed H16 (δ_H_ = 3.80 ppm) and the axial H19 (δ_H_ = 3.00 ppm) at the bridgehead of the diether ring system; although such a cross peak cannot be excluded in the first place, the intensity of the signal is rather counterintuitive.^14^

Both observations speak for a structure in which the −OH group is shifted by one position from C16 to C17 and is—most likely—equatorially rather than axially disposed on the *trans*-decaline type diether scaffold. In this case, however, the signal of the axially oriented H18 is expected to be a dd (or t) as a consequence of two large *J*-couplings to the vicinal protons, whereas the isolation paper reports a multiplet at δ_H_ = 3.81 ppm. However, this spectral region is fairly crowded: most notably, putative H16 resonates at δ_H_ = 3.80 ppm (m),^14^ and signal overlap may have obscured the assignments. Anyway, these issues together with the too low resolution of the published spectra of the natural product did not allow us to reach a firm and unambiguous conclusion. Therefore, we felt the need to make both isomers **1** and **2**, which only differ in their core region, in order to clarify the issue.

### Strategic Considerations

**Scheme 4:**
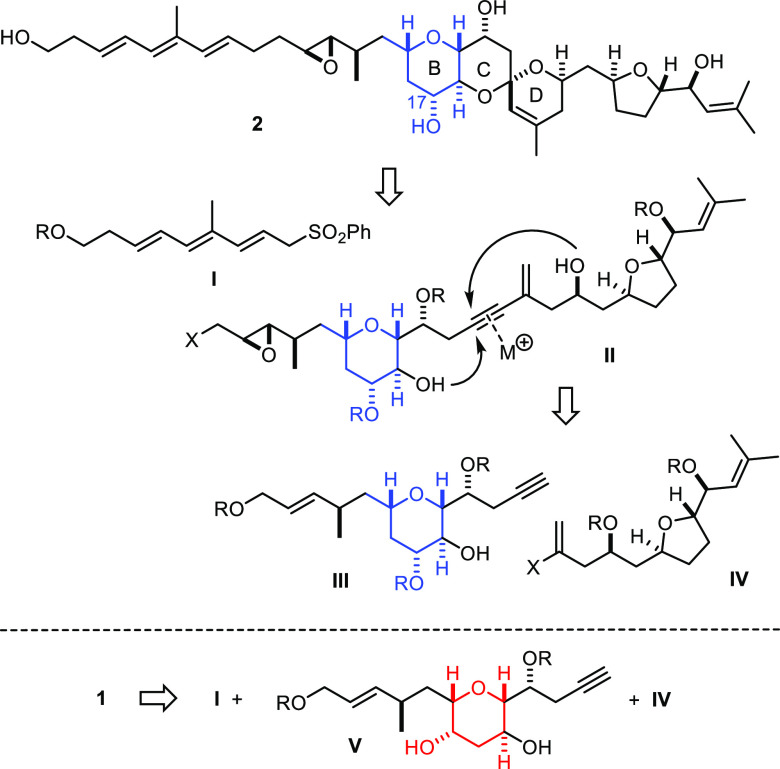
Retrosynthetic Analysis

In view of the size and complexity of the target and the outlook that more than one isomeric product might be needed to answer the open questions, a convergent assembly route was of key strategic relevance. Based on the lessons learned in our previous approach to limaol,^9^ a gold-catalyzed spirocyclization reaction of an appropriate alkyne precursor was deemed the ideal gateway to the isomeric tricyclic BCD ring systems in **1** and **2** (Scheme 4).^18−22^ An alkyne is a ketone surrogate that allows potential sensitivity issues associated with the use of highly functionalized carbonyl compounds to be avoided altogether; on treatment with a carbophilic Lewis acid, however, the triple bond is amenable to highly chemoselective activation in the presence of other π-bonds and heteroatoms alike.^23^

Moreover, an alkyne should pay additional dividends during the assembly process. In the present case, it invites coupling of the respective “central” segment **III** (or **V**) and the “eastern” building block **IV** via a Sonogashira reaction.^24^ Although we were prepared to make the latter in both enantiomeric forms in view of the uncertain interrelation between the core and side chain, a model exercise in computational spectroscopy had suggested that the depicted antipode of **IV** is the much more likely candidate.^25^ In any case, the allylic alcohol terminus present in the core would then serve for the installation of the yet missing *trans*-epoxide, followed by attachment of the lateral polyunsaturated side chain **I**. As already mentioned above, we chose to make the synthons with the absolute configuration shown in Scheme 4 based on the expectation that prorocentin and limaol might belong to the same enantiomeric series. If correct, these premises reduce the synthetic burden in that only the two isomeric central fragments **III** and **V** and the two wing sections **I** and **IV** need to be prepared; these building blocks can then be elaborated into the conceived product structures **2** and **1**, respectively, by a chemically highly conserved assembly process and endgame.

### Western Fragment

**Scheme 5:**
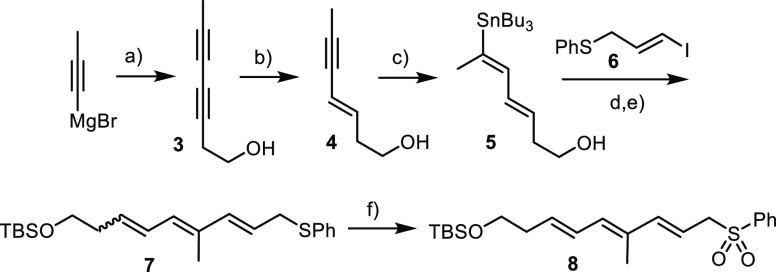
Western Fragment Reagents and conditions: (a) (1) I_2_, THF, −78 °C; (2) but-3-yn-1-ol, CuI (15 mol %), pyrrolidine, −78 °C to RT, 68%; (b) LiAlH_4_, THF, 53%; (c) Bu_3_SnSnBu_3_, BuLi, CuCN, H_2_O, THF, −78 to −40 °C, then **4**, −10 °C, 78%; (d) TBSCl, imidazole, DMAP (20 mol %), 95%; (e) **6**, Pd(PPh_3_)_4_ (10 mol %), CuTC, Ph_2_PO_2_NBu_4_, DMF, *E*/*Z* ≈ 10:1; and (f) Na_2_WO_4_ (25 mol %), aq H_2_O_2_, MeOH/benzene, 0 °C to RT, 63% (over both steps).

Commercial propynylmagnesium bromide was quenched with iodine, and the resulting crude alkynyl iodide coupled with 3-butyn-1-ol to furnish diyne **3** (Scheme 5). Upon treatment with LiAlH_4_, this compound underwent a hydroxy-directed *trans*-reduction to give enyne **4**.^26^

The subsequent stannylcupration with excess Bu_3_SnLi/CuCN in protic medium at low temperature gave stannane **5** with appreciable selectivity.^27^ After protection of the primary alcohol, the compound was subjected to Stille–Migita coupling with the known alkenyl iodide **6**(28) under conditions previously developed in this laboratory for particularly sensitive substrates.^29,30^ Even then, partial isomerization could not be fully suppressed; this issue, however, was readily solved since the all-*E* configured product could be separated from isomeric compounds after oxidation of thioether **7** to the corresponding phenyl sulfone **8** in readiness for late-stage fragment coupling.

### Eastern Fragment

**Scheme 6:**
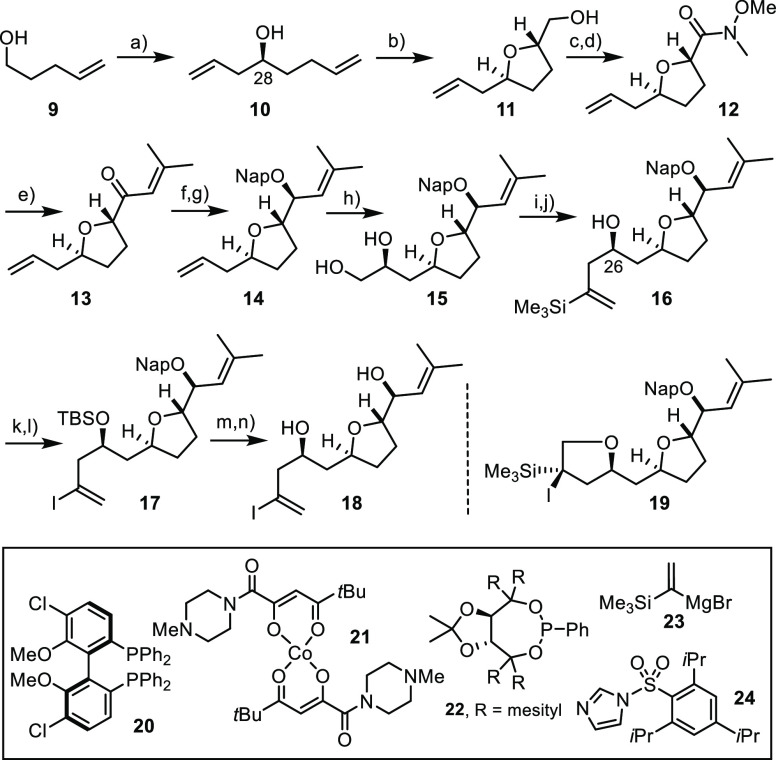
Eastern Fragment Reagents and conditions: (a) allyl acetate, [Ir(cod)Cl)]_2_ (2.5 mol %), **20** (5 mol %), Cs_2_CO_3_, 4-chloro-3-nitrobenzoic acid, THF, 81% [2 g scale], 96% ee; (b) **21** (10 mol %), *t*BuOOH, *i*PrOH, O_2_, 55 °C, dr > 20:1, 72% [4 g scale]; (c) PIDA, TEMPO (30 mol %), MeCN, H_2_O, 86% [3.3 g scale]; (d) *N*,*O*-dimethyl hydroxylamine hydrochloride, CDI, CH_2_Cl_2_, 89% [2.8 g scale]; (e) (1) 2-methyl-1-propenylmagnesium bromide, THF, 0 °C; (2) DBU cat., CH_2_Cl_2_, 98% [2.1 g scale]; (f) NaBH_4_, CeCl_3_·7H_2_O, MeOH, −78 °C, dr > 20:1, 94% [2.0 g scale]; (g) 2-(bromomethyl)naphthalene, NaH, TBAI, THF/DMF (2:1), 90% [0.6 g scale]; (h) (1) Pt(dba)_3_ (3 mol %), **22** (4 mol %), B_2_pin_2_, THF, 60 °C; (2) NaBO_3_·4H_2_O, THF/H_2_O, dr > 20:1, 76% [2.1 g scale]; (i) NaH, THF, then **24**, 0 °C to RT; (j) **23**, CuCN (10 mol %), THF, 0 °C, 74% (over both steps) [850 mg scale]; (k) TBSOTf, 2,6-lutidine, CH_2_Cl_2_, 0 °C to RT, 99% [200 mg scale]; (l) NIS, 2,6-lutidine, HFIP, THF, 0 °C, 80% [260 mg scale]; (m) TBAF, HOAc, THF, 83% [220 mg scale]; (n) DDQ, CH_2_Cl_2_, pH 7 buffer, 98% [110 mg scale]; the scales of substrates shown in this and the other schemes refer to the single largest batches.

Because of excellent previous experiences with the cobalt-catalyzed oxidative Mukaiyama cyclization of unsaturated alcohols,^31−33^ we resorted to this transformation for the formation of the 2,5-*trans*-disubstitued tetrahydrofuran ring contained in the eastern sector of prorocentin, using compound **10** as the substrate (Scheme 6).^34^ Although the secondary alcohol is flanked by two different alkenes, we have previously shown that the desired oxidative 5-*exo*-*trig* cyclization outcompetes other conceivable reaction modes.^34a^ This substrate was best prepared from commercial **9** and allyl acetate by means of an asymmetric Krische allylation using [Ir(cod)Cl]_2_ as a precatalyst in combination with the BIPHEP ligand **20**.^35^ Since alcohol **10** is rather volatile, the yields were consistently better when the reaction was performed on a larger scale; it is distinguished by an excellent level of induction (96% ee).^36^

As expected, this di-unsaturated compound underwent a highly selective cobalt-catalyzed oxidative etherificaton to furnish the desired *trans*-configured THF derivative **11** in 72% yield as the only isomer; to this end, cobalt complex **21** proved optimal.^37,38^ Subsequent oxidation of the newly formed primary alcohol to the corresponding acid was followed by elaboration into enone **13** by the addition of 2-methyl-1-propenylmagnesium bromide to Weinreb amide **12**. Somewhat unexpectedly, the crude material contained variable amounts of ketone with the double bond out of conjugation; exposure to catalytic DBU rectified the outcome and gave the desired product in almost quantitative yield. Equally rewarding was the excellent diastereoselectivity with which the carbonyl group of **13** could be reduced to the required allylic alcohol under Luche conditions.^39^ To secure stability as well as ready deprotection at a later stage, the −OH group was converted into the corresponding 2-naphthylmethyl (rather than a simple benzyl) ether **14** prior to transformation of the alkene terminus into the functionality needed for the envisaged late-stage fragment union.^40,41^

After some experimentation, it was found that a platinum-catalyzed asymmetric diborylation/oxidation sequence under the auspices of **22** as the ligand served this purpose very well.^42,43^ The reaction furnished diol **15** in 76% yield (dr > 20:1) and proved nicely scalable.^36^ Upon treatment with excess NaH and the bulky sulfonyl imidazole derivative **24**, the diol was converted into the corresponding epoxide, which was immediately subjected to ring opening with the silylated Grignard reagent **23** in the presence of catalytic CuCN to give product **16** in good overall yield.^44^

This compound is stable for extended periods of time and therefore served as the “stock form” of the eastern fragment. Prior to use, however, the alkenylsilane must be converted into an alkenyl halide. All attempts at affecting this transformation in the presence of the unprotected C26–OH group (prorocentin numbering) failed;^45^ the ether derivative **19** was the only detectable product in the crude mixture. The problem was remedied by transiently capping the −OH group as TBS-ether, which underwent clean iodo-desilylation on treatment with NIS in CH_2_Cl_2_/HFIP to furnish alkenyl iodide **17**;^46^ the medium was supplemented with 2,6-lutidine to ensure a basic pH in order to keep the silyl ether intact at all time. Only after the iodo-desilylation had been completed, the silyl and naphthylmethyl protecting groups were successively removed with the aid of TBAF/HOAc and DDQ, respectively, to give product **18** in readiness for fragment coupling.^41^

It is also worth mentioning that access to the enantiomeric form of this building block could follow the exact same route since all critical stereodetermining transformations are under ligand control. As will be shown below, however, it actually proved unnecessary to make *ent*-**18** in order to complete the project.

### “Original” Central Fragment

**Scheme 7:**
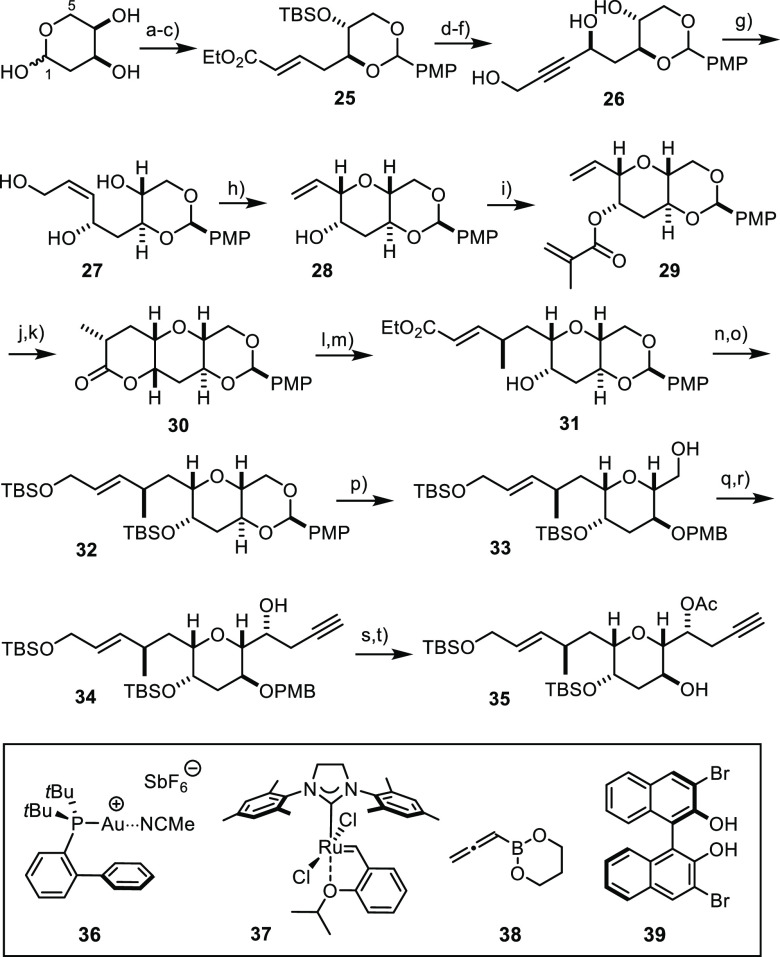
Core of Nominal Prorocentin Reagents and conditions: (a) Ph_3_P=CHCOOEt, THF, reflux; (b) *p*MeOC_6_H_4_CH(OMe)_2_, CSA (20 mol %), CH_2_Cl_2_; (c) TBSCl, imidazole, DMF, 49% (over three steps) [10 g scale]; (d) O_3_, CH_2_Cl_2_, −78 °C, then PPh_3_, 92% [2.4 g scale]; (e) TBSOCH_2_C≡CH, Zn(OTf)_2_, (−)-*N*-methylephedrine, Et_3_N, toluene; (f) TBAF, THF, dr > 20:1, 65% (over both steps) [5 g scale]; (g) H_2_, Lindlar catalyst, quinoline, EtOAc, 0 °C, 91% [2.6 g scale]; (h) **36** (5 mol %), 2,6-di-*tert*-butylpyridine (15 mol %), THF/CH_2_Cl_2_, MS 4 Å, dr > 20:1, 86% [950 mg scale]; (i) methacryloyl chloride, Et_3_N, DMAP (15 mol %), CH_2_Cl_2_, 86% [765 mg scale]; (j) **37** (15 mol %), toluene, reflux, 67–77% [1.3 g scale]; (k) Pd/C, H_2_ (1 atm), EtOAc, dr > 20:1, 95% [1.05 g scale]; (l) Dibal-H, CH_2_Cl_2_, −78 °C, 96% [1.01 g scale]; (m) Ph_3_P=CHCOOEt, THF/toluene, 80 °C, 58–74% [970 mg scale]; (n) Dibal-H, THF, −78 °C, quant. [670 mg scale]; (o) TBSOTf, 2,6-lutidine, CH_2_Cl_2_, −78 °C, 90% [330 mg scale]; (p) Dibal-H, CH_2_Cl_2_, −78 °C, 93% [600 mg scale]; (q) oxalyl chloride, DMSO, Et_3_N, CH_2_Cl_2_, −78 °C; (r) **38**, **39** (30 mol %), toluene, 68% (over both steps) + 16% of isomer [500 mg scale]; (s) Ac_2_O, pyridine, DMAP (10 mol %), CH_2_Cl_2_, quant. [460 mg scale]; and (t) DDQ, CH_2_Cl_2_, pH 7 buffer, 66% [500 mg scale].

Based on our hypothesis concerning the absolute configuration of prorocentin, 2-deoxy-d-ribose was chosen as the entry point for the synthesis of the central fragment composed in the originally proposed structure **1** (Scheme 7). A literature-known sequence of Wittig reaction, thermodynamic acetalization, and TBS protection afforded multigram quantities of product **25**.^47^ Next, ozonolysis followed by Carreira alkynylation^48^ with TBS-protected propargyl alcohol as the reagent and global desilylation led to triol **26**, again on scale. After Lindlar hydrogenation of the triple bond, which proceeded without significant amounts of overreduction only when performed in a non-protic medium at 0 °C,^49^ the resulting product **27** served the formation of the distinguishing 2,6-*cis* configured future B-ring very well. Specifically, this rather challenging stereochemical pattern was set by gold-catalyzed allylic substitution under buffered conditions.^50^ The reaction is convenient as the alcohol to be activated does not need to be converted into a better leaving group; rather, the triol **27** can be used as is. We found that the reaction was best carried out with the cationic gold complex **36**; under these conditions, a high yield was secured on a gram scale. Most importantly, however, the observed stereoselectivity was excellent, and the outcome matched the prediction, as confirmed by X-ray diffraction analysis of the resulting product **28** (see the Supporting Information). Esterification on treatment with methacryloyl chloride led to diene **29**, which is polymerization-prone and therefore needed to be processed without delay. As expected, treatment with the second-generation Hoveyda–Grubbs-type catalyst **37**(51) transformed this substrate into the corresponding unsaturated lactone by RCM.^52^ For the curvature of this rigid tricyclic olefin, simple hydrogenation over Pd/C sufficed to give **30** as the only product, in which the methyl branch is properly set, as confirmed by NOESY data. Semi-reduction of the lactone with Dibal-H in CH_2_Cl_2_ was followed by chain extension of the resulting hemiketal via Wittig olefination to form enoate **31** in good yield.

Subtle differences in reactivity deserve mentioning at this point. While the lactone carbonyl of **30** was readily reduced with Dibal-H in CH_2_Cl_2_, the conversion of the ester group in **31** to the corresponding allylic alcohol mandated the use of excess Dibal-H in THF. This reagent left the *p*-methoxybenzylidene acetal intact even on prolonged stirring; in CH_2_Cl_2_, in contrast, Dibal-H eventually allowed the acetal ring of **32** to be cleaved and the primary alcohol **33** to be secured in high yield on gram scale.

Oxidation to the corresponding aldehyde was best performed under Swern conditions.^53^ The subsequent asymmetric propargylation proceeded nicely under an organocatalytic setting originally developed by Schaus and Barnett for ketones,^54^ which we had already adapted to elaborate aldehyde substrates in our total synthesis of limaol.^9,55^ A Mosher ester analysis confirmed that the newly formed alcohol of the major product had been properly set.^36^ Simple adjustment of the protecting groups converted compound **34** into the central fragment **35** to be incorporated into nominal prorocentin as outlined below.^56^

### “Revised” Central Fragment

**Scheme 8:**
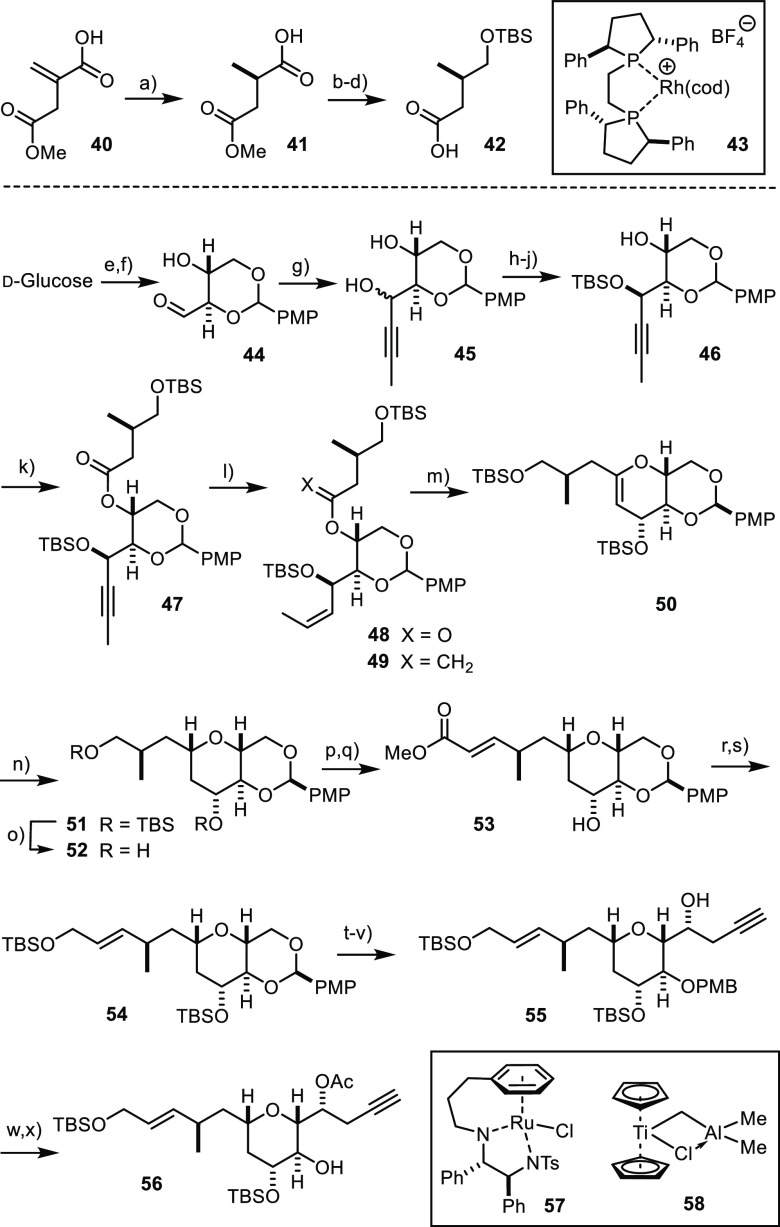
Revised Core Reagents and conditions: (a) **43** (0.03 mol %), H_2_ (15 bar), MeOH, 98% ee, quant. [8.5 g scale]; (b) BH_3_ SMe_2_, THF, −30 °C; (c) TBSCl, imidazole, CH_2_Cl_2_, 92% (over both steps) [8.5 g scale]; (d) LiOH, THF, MeOH, H_2_O, 0 °C to RT, quant. [13.1 g scale]; (e) *p*MeOC_6_H_4_CH(OMe)_2_, *p*TsOH·H_2_O, DMF; (f) NaIO_4_, NaOAc, HOAc, aq MeOH, 56% (over two steps) [11 g scale]; (g) propynylmagnesium bromide, THF, dr = 2:1, 81% [4.2 g scale]; (h) MnO_2_, CH_2_Cl_2_, 61% [8 g scale]; (i) **57** (5 mol %), HCOOH/Et_3_N, CH_2_Cl_2_, dr > 20:1, 91% [4.8 g scale]; (j) TBSCl, imidazole, CH_2_Cl_2_, 87% [4.4 g scale]; (k) **42**, EDC, DMAP, CH_2_Cl_2_, 97% [1.4 g scale]; (l) H_2_ (1 atm), Lindlar catalyst, quinoline (30 mol %), EtOAc, 0 °C, 98% [7.1 g scale]; (m) **58**, THF/toluene, 47% [7 g scale]; (n) H_2_ (1 bar), Pt/C cat., EtOH, 0 °C, dr > 20:1, 74–83% [3.2 g scale]; (o) TBAF, THF, 81% [2.4 g scale]; (p) Dess–Martin periodinane, NaHCO_3_, CH_2_Cl_2_, 0 °C; (q) Ph_3_P=CHCOOMe, CH_2_Cl_2_, 86% (over both steps) [1.1 g scale]; (r) Dibal-H, THF, −78 °C, 83% [1.1 g scale]; (s) TBSOTf, 2,6-lutidine, CH_2_Cl_2_, −78 °C, 95% [940 mg scale]; (t) Dibal-H, CH_2_Cl_2_, −78 °C, 87–93% [1.45 g scale]; (u) oxalyl chloride, DMSO, Et_3_N, CH_2_Cl_2_, −78 to 0 °C; (v) **38**, **39** (30 mol %), toluene, dr > 20:1, 44–59% yield (over two steps) [1.16 g scale]; (w) Ac_2_O, pyridine, DMAP, CH_2_Cl_2_, 90% [610 mg scale]; and (x) DDQ, CH_2_Cl_2_, pH 7 buffer, 77% [580 mg scale].

Because of the different oxygenation pattern of the revised central BCD ring system, a more involved route had to be devised. It starts with the conversion of d-glucose into β-hydroxy aldehyde **44** on a multigram scale (Scheme 8).^57,58^ Although α-branched and hence expected to be more amenable to asymmetric Carreira alkynylation than the corresponding aldehyde passed through en route to product **26**,^48^ the reaction with propyne as the nucleophile essentially met with failure; this result came as a surprise since our group (and others) had already performed successful asymmetric propynylations under these conditions in the past.^59−61^ This setback forced us to pursue a three-step workaround, commencing with the high-yielding addition of propynylmagnesium bromide to **44**, oxidation of the mixture of diastereomeric alcohols **45** thus formed with MnO_2_, followed by stereoselective-modified Noyori-type transfer hydrogenation of the resulting ynone using the tethered ruthenium catalyst **57**.^62,63^

The selective protection of the propargylic site proceeded uneventfully on scale, thus setting the stage for the esterification of the remaining −OH group in **46** with acid **42**. This compound, in turn, derives from itaconic acid monoester **40** by asymmetric hydrogenation using the particularly effective rhodium catalyst **43**,^64,65^ chemoselective reduction of acid **41** with borane, and subsequent adjustment of the protecting groups.

With compound **47** in hand, the stage was set for one of the most demanding transformations of the entire total synthesis endeavor reported herein. In careful consideration of the pertinent literature,^66−68^ the triple bond in **47** was first reduced to the corresponding *Z*-alkene **48**.^69^ When treated with a large excess of Tebbe’s reagent (**58**), best generated in situ from Cp_2_TiCl_2_ and Me_3_Al,^70^ in a mixture of THF and toluene, the ester group first succumbed to olefination with the formation of enol ether **49**. On prolonged stirring, diene **49** reacted further to give the desired cyclic enol ether **50**. Although the yield of product was only 47%, this olefination/metathesis cascade could be performed on a >7 g scale, such that a good material throughput was secured. In contrast to what the literature precedent had suggested, however, the use of the Petasis reagent in lieu of the original Tebbe complex failed to afford product **50**;^66^ rather, the reaction invariably stopped at the stage of **49**. The same is true for the Takai–Utimoto reagent, which did not induce ring closure either.^71^ All attempts at converting **49** in a separate step into the cyclic enol ether **50** with the aid of classical Schrock or Grubbs-type catalysts for olefin metathesis were to no avail either.^69,72^

The hydrogenation of **50** proceeded stereoselectively to set the distinctive 2,6-*cis*-substitution pattern of the B-ring of prorocentin;^73^ this outcome is clearly manifested in the *J*-coupling pattern of the protons on the rim. Of all catalysts tested, only platinum on charcoal in EtOH gave well reproducible results without jeopardizing the aromatic ring of the *p*-methoxybenzylidene acetal. Although the selective deprotection of the primary TBS-ether in **51** is possible with HF·pyridine in pyridine, it was not overly high yielding (66%). Rather, it proved advantageous to cleave both silyl groups at the same time and subject the resulting diol **52** to a pleasingly effective mono-oxidation. As it turned out, Dess–Martin periodinane^74^ in CH_2_Cl_2_ at 0 °C transformed the primary alcohol into the corresponding aldehyde without touching the secondary −OH group to any noticeable extent. The crude product was then subjected to chain extension by Wittig olefination, and the resulting ester **53** was reduced with Dibal-H in THF.

From this point onward, the route follows the steps described above for the original core segment **35**. Thus, reduction of the ester terminus of **53** was followed by protection of both −OH groups in the resulting product, which furnished silyl ether derivative **54**. This compound was subjected to regioselective cleavage of the acetal on treatment with Dibal-H in CH_2_Cl_2_. The resulting primary alcohol was oxidized, and the aldehyde thus formed was instantly subjected to ligand-controlled asymmetric propargylation,^54^ which afforded **55** as the only detectable product.^36^ Acetylation, followed by oxidative cleavage of the PMB-ether, furnished the fully functional core segment **56** as needed for the projected end game.

### Actual Prorocentin

With good amounts of all the required building blocks in hand, the completion of the total syntheses of the two conceived targets **1** and **2** could be performed in parallel. Although the individual products of the two series are isomeric to each other, only minor differences were observed from the chemical point of view. Therefore, the discussion can be focused on the conquest of actual prorocentin.

**Scheme 9:**
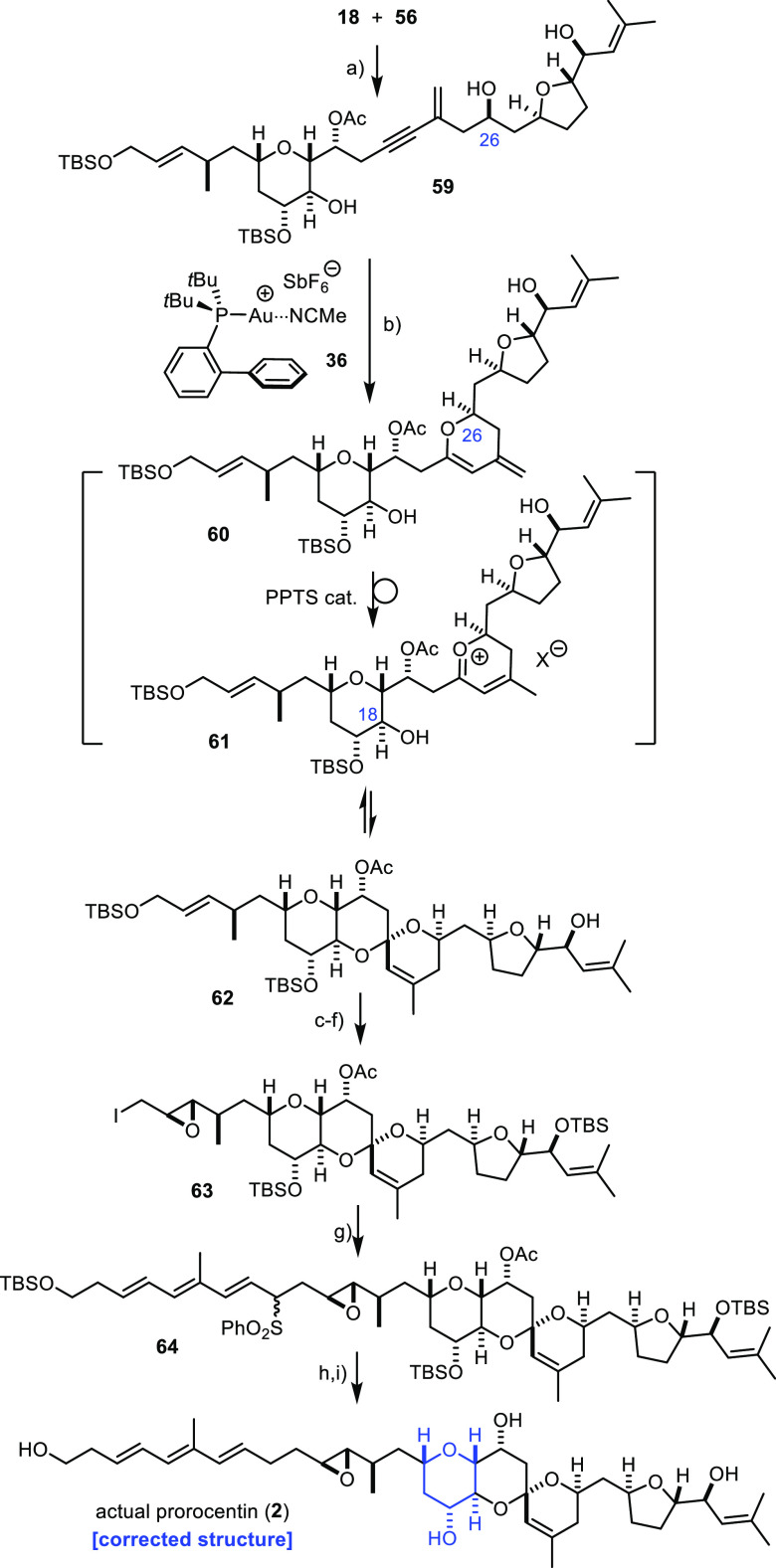
Completion of the Total Synthesis of Actual Prorocentin Reagents and conditions: (a) Pd_2_(dba)_3_ (5 mol %), CuI (20 mol %), PPh_3_ (20 mol %), *i*Pr_2_NH, 91% [200 mg scale]; (b) **36** (10 mol %), PPTS (10 mol %), CH_2_Cl_2_, 69% [71 mg scale]; (c) TBSOTf, 2,6-lutidine, CH_2_Cl_2_, −78 °C, 92% [145 mg scale]; (d) HF pyridine, pyridine, 92% [152 mg scale]; (e) Ti(O*i*Pr)_4_ (15 mol %), l-(+)-DIPT, cumene hydroperoxide, CH_2_Cl_2_, −25 °C, dr > 20:1, 84% [40 mg scale]; (f) I_2_, PPh_3_, imidazole, CH_2_Cl_2_, 0 °C, 88% [34 mg scale]; (g) **8**, *n*BuLi, THF, DMPU, then 63, −78 to −60 °C, dr ≈ 3:2, 86% [34 mg scale]; (h) (1) LiBHEt_3_, THF, −78 to −20 °C; (2) [(dppp)PdCl_2_] (10 mol %), LiBHEt_3_, −10 °C, 49% [34 mg scale]; and (i) HF pyridine, THF, pyridine, 50% (40% HPLC).

The first critical fragment union consisted of the palladium-catalyzed Sonogashira coupling of the terminal alkyne **56** with the eastern fragment **18** (Scheme 9).^24^ The reaction proceeded slowly but cleanly, provided *i*Pr_2_NH was chosen as the reaction medium. Gratifyingly, the resulting polyfunctionalized enyne **59** was well behaved in the subsequent gold/Brønsted acid co-catalyzed spirocyclization, which afforded product **62** as the only isomer in 69% yield. It is the gold catalyst **36** that triggers the decisive first 6-*endo*-*dig* cyclization by the attack of the C26–OH group onto the distal end of the triple bond; as a result, the required 6/6-spirocyclic array ultimately prevails over all other conceivable regioisomeric spiroacetals.^75^ The resulting enol ether **60** is then likely activated by the admixed PPTS co-catalyst to generate a reactive oxocarbenium cation **61** in situ, which gets trapped by the sterically more hindered secondary C18–OH group residing on the pre-existing tetrahydropyran ring.^76^ As this second step is reversible, the exclusive formation of the doubly anomeric array of **62** is readily understood. Moreover, we suppose that the observed selective migration of the *exo*-methylene group to the endocyclic position also occurs upon protonation of enol ether **60**.^77,78^ This order of events can be inferred from model studies in a closely related series (see the Supporting Information).

The completion of the total synthesis commenced by unveiling the primary allylic alcohol, which underwent a Sharpless epoxidation to introduce the yet missing *trans*-configured oxirane.^79^ Subsequent conversion of the −OH group into the corresponding primary iodide **63** was followed by C-alkylation of the deprotonated sulfone **8** to complete the carbon framework of prorocentin; neither the oxirane nor the acetate group of **63** interfered with this step, and the sensitive triene subunit also remained untouched as long as the reaction was carried out at low temperature in THF/DMPU.^80^ Product **64** thus formed was then treated with LiBHEt_3_ in THF to first cleave the axially disposed acetate decorating the C-ring.^81^ The mixture was then supplemented with catalytic [(dppp)PdCl_2_], followed by a second portion of LiBHEt_3_; this reagent combination entailed desulfonation by the formation of a transient allylpalladium species upon insertion into the allylic sulfone, followed by trapping of this intermediate by the admixed hydride donor.^82,83^ This delicate one-pot maneuver left the epoxide ring and the triene entity intact, although the desired product was isolated in modest 49% yield as the only defined compound present in the crude reaction mixture. Global deprotection with buffered HF pyridine in THF/pyridine then afforded product **2**.

As foreshadowed by the reassessment of the published NMR data of the natural product and in line with the preliminary conference proceeding,^17^ it is this structurally revised compound **2** which represents prorocentin; the perfect match of the recorded and the reported ^1^H and ^13^C NMR data leaves no doubt whatsoever (for details, see the Supporting Information). Moreover, the rotatory power of our synthetic samples of **2** [[α]_D_^25^ = −12.0 (c 0.1, MeOH)] corresponds very well to the literature [[α]_D_^25^ = −12.7 (c 0.2, MeOH)].^14^ This comparison also confirms our original assumption that prorocentin and limaol belong to the same enantiomeric series.

### Nominal Prorocentin

**Scheme 10:**
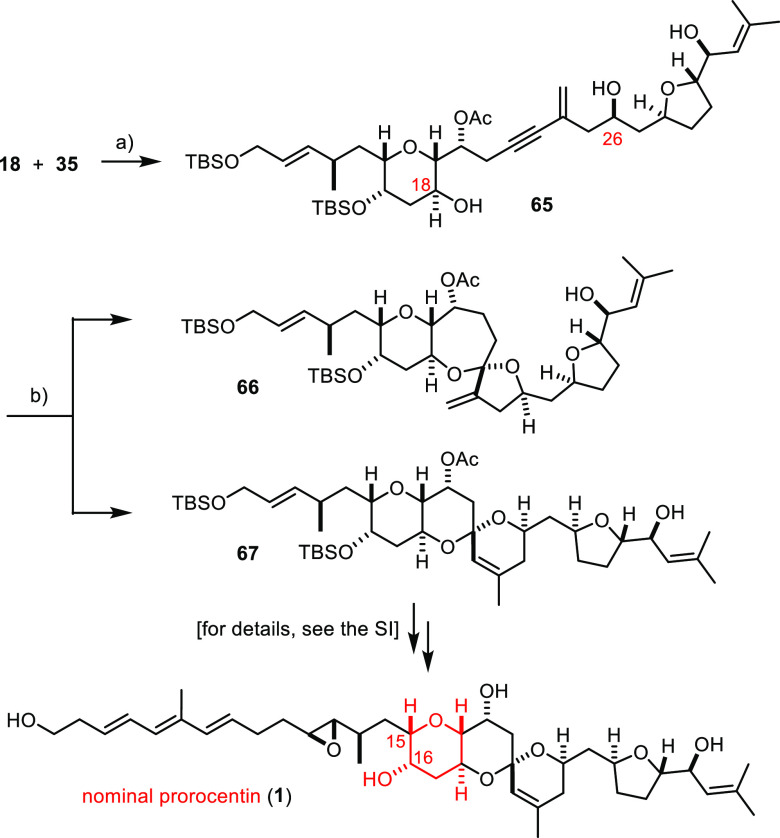
Completion of the Total Synthesis of Nominal Prorocentin Reagents and conditions: (a) Pd_2_(dba)_3_ (5 mol %), CuI (20 mol %), PPh_3_ (20 mol %), *i*Pr_2_NEt, THF, 99%; and (b) **36** (10 mol %), PPTS (10 mol %), CH_2_Cl_2_, **67**:**66** ≈ 10:1 (NMR), 84%.

Although the spectral and analytical data of synthetic **2** left no room for surprise, we nevertheless completed the total synthesis of nominal prorocentin (**1**) as the target structure originally proposed by the isolation team (Scheme 10).^14^ Starting from the isomeric central fragment **35**, the route leading to this target compound followed the exact same steps and therefore does not need detailed comments. An exception is the gold-catalyzed spiroketalization; although again high yielding, the reaction was slightly less selective in regioisomeric terms. In addition to the desired 6/6/6 fused/spirotricyclic product **67**, small but non-negligible amounts of the 6/7/5 configured product **66** were detected. This side reaction suggests that the secondary C18–OH group flanked by a methylene unit in the cyclization precursor **65** is less hindered than that in compound **59**, which resides adjacent to a bulky TBS-ether; for this gentle relief of steric pressure, this hydroxy group starts to interfere in the actual gold-catalyzed hydroalkoxylation of the triple bond, although the attack by the C26–OH is still dominant. Gratifyingly, the isomeric spiroacetal **66** could be removed by flash chromatography, and the synthesis of **1** could hence be completed with pure **67** in analogy to what has been described above (for the full Scheme, see the Supporting Information).

The spectral properties of **1** clearly deviate from those of the natural product. Indeed, it is the regions *exo* as well as *endo* about the branching point C15 that are unmistakably at variance with the reported data. The originally proposed structure of prorocentin (**1**) is hence definitely incorrect and needs to be replaced with the isomeric structure **2**, which depicts the correct constitution and stereostructure of this intriguing marine natural product.

## Conclusions

In the past, our group—like many others—had to revise the structure (and/or the proposed bioactivity) of numerous presumed natural products;^84,85^ the prorocentin case is another important entry in this list. We are stunned, however, by the fact that the correct solution had obviously been found more than a decade ago by another team but had not been prominently published.^17^

In any case, the conquest of prorocentin (**2**) and its non-natural isomer **1** by the entirely new approach disclosed herein denotes yet another triumph of π-acid catalysis in natural product synthesis. This success, however, has only been possible in concert with numerous other metal-catalyzed and organocatalytic reactions that paved the way to the required substrates. While multiple recourse to palladium catalysis, an RCM with a Grubbs-type catalyst, the Sharpless epoxidation, and catalytic (asymmetric) reductions do not come as a surprise, the demanding RCM of an enol ether by the Tebbe reagent, although long known in the literature, is noteworthy; the same is true for the exquisitely regio- and diastereoselective Mukaiyama-type oxidative etherification of a di-unsaturated alcohol derivative. Moreover, the platinum-catalyzed asymmetric diboration/oxidation of a terminal alkene and the doubly diastereomeric propargylations of chiral aldehydes that proceeded under stringent catalyst control were found equally enabling; these examples bear witness to the tremendous progress in asymmetric catalysis during the last decade and augur well for future applications.

We hope that our samples will allow us, in cooperation with expert partners, to shed light on the so far largely unknown activity of **2**. This investigation is driven by the firm belief that the evolution of natural products as intricate as prorocentin comes along with innate biological implications.

## Supporting Information Available

Experimental section including characterization data and NMR spectra of new compounds; crystallographic summary (compound **28**); reassessed spectra of the natural product; supporting computational data; and details concerning the total synthesis of nominal prorocentin (PDF)
