# Supplementary material for: Total Syntheses of Nominal and Actual Prorocentin
Source: J Am Chem Soc. 2023 Jan 18;145(4):2584–95. doi: 10.1021/jacs.2c12529 (PMC9896551; doi:10.1021/jacs.2c12529)
Supplement: Supplementary file 1 — ja2c12529_si_001.pdf [file ja2c12529_si_001.pdf]

SUPPORTING INFORMATION

Total Syntheses of Nominal and Actual Prorocentin

Raphael J. Zachmann, Kenzo Yahata, Mira Holzheimer, Maxime Jarret, Cornelia Wirtz, and Alois Fürstner*

Max-Planck-Institut für Kohlenforschung, 45470 Mülheim/Ruhr, Germany

Email: fuerstner@kofo.mpg.de

TABLE OF CONTENTS

SUPPORTING CRYSTALLOGRAPHIC INFORMATION	S2
REASSESSMENT OF THE 2D NMR SPECTRA OF THE NATURAL PRODUCT	S5
SUPPORTING COMPUTATIONAL DATA	S8
MODEL STUDY: PROGRESSION OF THE SPIROCYCLIZATION REACTION	S9
COMPLETION OF THE TOTAL SYNTHESIS OF NOMINAL PROROCENTIN	S12
GENERAL INFORMATION	S13
THE WESTERN FRAGMENT	S14
THE EASTERN FRAGMENT	S16
THE CENTRAL FRAGMENT OF NOMINAL PROROCENTIN	S26
THE REVISED CENTRAL FRAGMENT	S37
NOMINAL PROROCENTIN	S49
ACTUAL PROROCENTIN	S61
COPIES OF SPECTRA	S73
REFERENCES	S154

SUPPORTING CRYSTALLOGRAPHIC INFORMATION

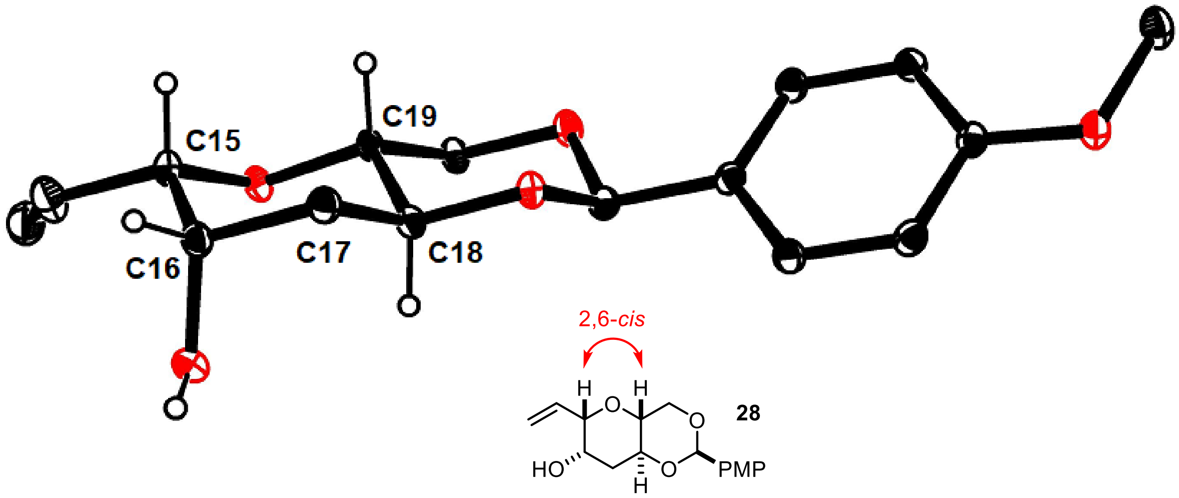

Figure S1. Structure of compound **28** formed by the gold catalyzed allylic substitution reaction in the solid state. All H-atoms are omitted for clarity except for those at the branching point (C15), at C16 carrying the axially disposed –OH group, and the junction of the fused *trans*-decaline-type ether ring system (prorocentin numbering scheme). Atomic displacement ellipsoids are shown at the 50% probability level.

X-Ray Crystal Structure Analysis of Compound 28: $C_{16}H_{20}O_5$, M_r = 292.32 g mol^{-1}, colorless prism, crystal size 0.157 x 0.144 x 0.123 mm^3, monoclinic, space group *C*2 [5], *a* = 19.7449(5) Å, *b* = 8.0966(2) Å, *c* = 10.8814(3) Å, β = 121.6560(10)°, *V* = 1480.75(7) Å^3, *T* = 100(2) K, *Z* = 4, D_{calc} = 1.311 g·cm^3, λ = 1.54178 Å, $\mu(Cu\text{-}K_\alpha)$ = 0.803 mm^{-1}, Gaussian absorption correction (T_{min} = 0.91, T_{max} 0.94), Bruker-AXS Kappa Mach3 with APEX-II detector and IµS microfocus X-ray source, 4.774 < θ < 70.943°, 25460 measured reflections, 2785 independent reflections, 2308 reflections with $I > 2\sigma(I)$, R_{int} = 0.0519. The structure was solved by *SHELXT* and refined by full-matrix least-squares (*SHELXL*) against F^2 to R_1 = 0.034 [$I > 2\sigma(I)$], wR_2 = 0.119 [all data], 192 parameters, absolute structure parameter Flack (x) = –0.05(6), Largest diff. peak and hole = 0.6 (1.07 Å from H2) and –0.3 (0.52 Å from C1) e·Å^{-3}.

Complete .cif-data of the compound are available under the CCDC number **CCDC-2219417**.

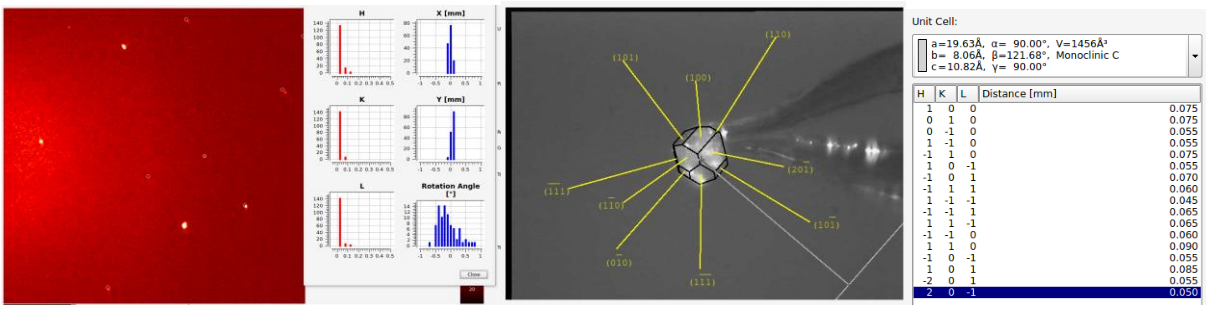

Figure S2. Crystal faces and unit cell determination/refinement.

INTENSITY STATISTICS FOR DATASET # 1 13066sadabs.raw

Resolution	#Data	#Theory	%Complete	Redundancy	Mean I	Mean I/s	Rmerge	Rsigma
Inf - 3.37	42	42	100.0	9.26	144.08	90.70	0.0212	0.0108
3.37 - 2.23	97	97	100.0	9.42	45.07	83.88	0.0269	0.0104
2.23 - 1.77	141	141	100.0	10.34	34.58	76.61	0.0261	0.0107
1.77 - 1.54	141	141	100.0	9.78	18.95	65.73	0.0279	0.0127
1.54 - 1.41	138	138	100.0	8.77	12.42	52.56	0.0314	0.0152
1.41 - 1.30	145	145	100.0	7.40	9.51	39.64	0.0388	0.0194
1.30 - 1.23	129	129	100.0	7.29	11.48	35.51	0.0472	0.0216
1.23 - 1.17	139	139	100.0	13.63	9.93	39.67	0.0510	0.0170
1.17 - 1.11	168	168	100.0	13.35	14.16	48.86	0.0449	0.0150
1.11 - 1.07	134	135	99.3	12.56	8.10	39.36	0.0516	0.0203
1.07 - 1.03	149	149	100.0	12.35	7.21	35.02	0.0563	0.0207
1.03 - 1.00	126	128	98.4	11.28	6.02	29.57	0.0895	0.0350
1.00 - 0.97	140	145	96.6	10.75	4.99	23.76	0.1588	0.0326
0.97 - 0.95	128	133	96.2	10.29	3.65	19.65	0.1658	0.0487
0.95 - 0.92	163	169	96.4	9.47	2.87	16.85	0.2037	0.0560
0.92 - 0.90	120	125	96.0	9.42	2.73	17.08	0.2203	0.0584
0.90 - 0.88	146	153	95.4	9.44	2.08	15.89	0.2438	0.0673
0.88 - 0.86	169	177	95.5	5.88	2.20	10.17	0.2228	0.2608
0.86 - 0.84	156	165	94.5	2.04	1.86	2.07	0.3414	0.9222
0.84 - 0.83	84	89	94.4	2.21	1.32	1.83	0.3228	1.0803
0.83 - 0.82	130	166	78.3	1.55	0.98	1.45	0.3777	1.4172
0.92 - 0.82	805	875	92.0	5.09	1.90	8.39	0.2422	0.4598
Inf - 0.82	2785	2874	96.9	8.86	11.63	33.35	0.0496	0.0367

Table S1. Bond lengths [Å] and angles [°] of compound **28**; crystallographic numbering scheme as shown in the insert.

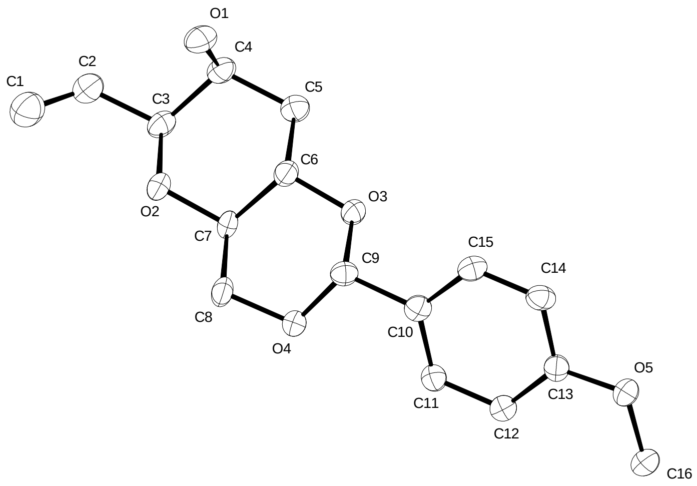

O(1)-C(4)	1.429(4)		
O(2)-C(3)	1.448(4)		
O(2)-C(7)	1.437(3)		
O(3)-C(6)	1.427(3)		
O(3)-C(9)	1.420(4)		
O(4)-C(8)	1.442(3)		
O(4)-C(9)	1.403(4)		
O(5)-C(13)	1.372(3)		
O(5)-C(16)	1.429(4)	C(1)-C(2)	1.265(6)
C(2)-C(3)	1.499(5)	C(3)-C(4)	1.534(5)
C(4)-C(5)	1.521(4)	C(5)-C(6)	1.515(4)
C(6)-C(7)	1.525(4)	C(7)-C(8)	1.511(4)
C(9)-C(10)	1.502(4)	C(10)-C(11)	1.383(4)
C(10)-C(15)	1.397(4)	C(11)-C(12)	1.397(4)
C(12)-C(13)	1.383(4)	C(13)-C(14)	1.396(4)
C(14)-C(15)	1.374(4)		

C(7)-O(2)-C(3)	110.9(2)	C(9)-O(3)-C(6)	110.2(2)
C(9)-O(4)-C(8)	111.3(2)	C(13)-O(5)-C(16)	116.7(2)
C(1)-C(2)-C(3)	128.8(4)	O(2)-C(3)-C(2)	111.0(3)
O(2)-C(3)-C(4)	111.2(2)	C(2)-C(3)-C(4)	112.1(3)
O(1)-C(4)-C(3)	107.9(3)	O(1)-C(4)-C(5)	111.1(2)
C(5)-C(4)-C(3)	110.9(2)	C(6)-C(5)-C(4)	107.8(2)
O(3)-C(6)-C(5)	110.5(2)	O(3)-C(6)-C(7)	109.4(2)
C(5)-C(6)-C(7)	109.7(2)	O(2)-C(7)-C(6)	108.8(2)
O(2)-C(7)-C(8)	110.2(2)	C(8)-C(7)-C(6)	110.0(2)
O(4)-C(8)-C(7)	108.2(2)	O(3)-C(9)-C(10)	107.5(2)
O(4)-C(9)-O(3)	111.2(2)	O(4)-C(9)-C(10)	110.9(2)
C(11)-C(10)-C(9)	122.8(3)	C(11)-C(10)-C(15)	118.7(3)
C(15)-C(10)-C(9)	118.5(2)	C(10)-C(11)-C(12)	120.9(3)
C(13)-C(12)-C(11)	119.5(3)	O(5)-C(13)-C(12)	124.9(3)
O(5)-C(13)-C(14)	115.3(3)	C(12)-C(13)-C(14)	119.9(3)
C(15)-C(14)-C(13)	120.0(3)	C(14)-C(15)-C(10)	120.9(3)

REASSESSMENT OF THE 2D NMR SPECTRA OF THE NATURAL PRODUCT

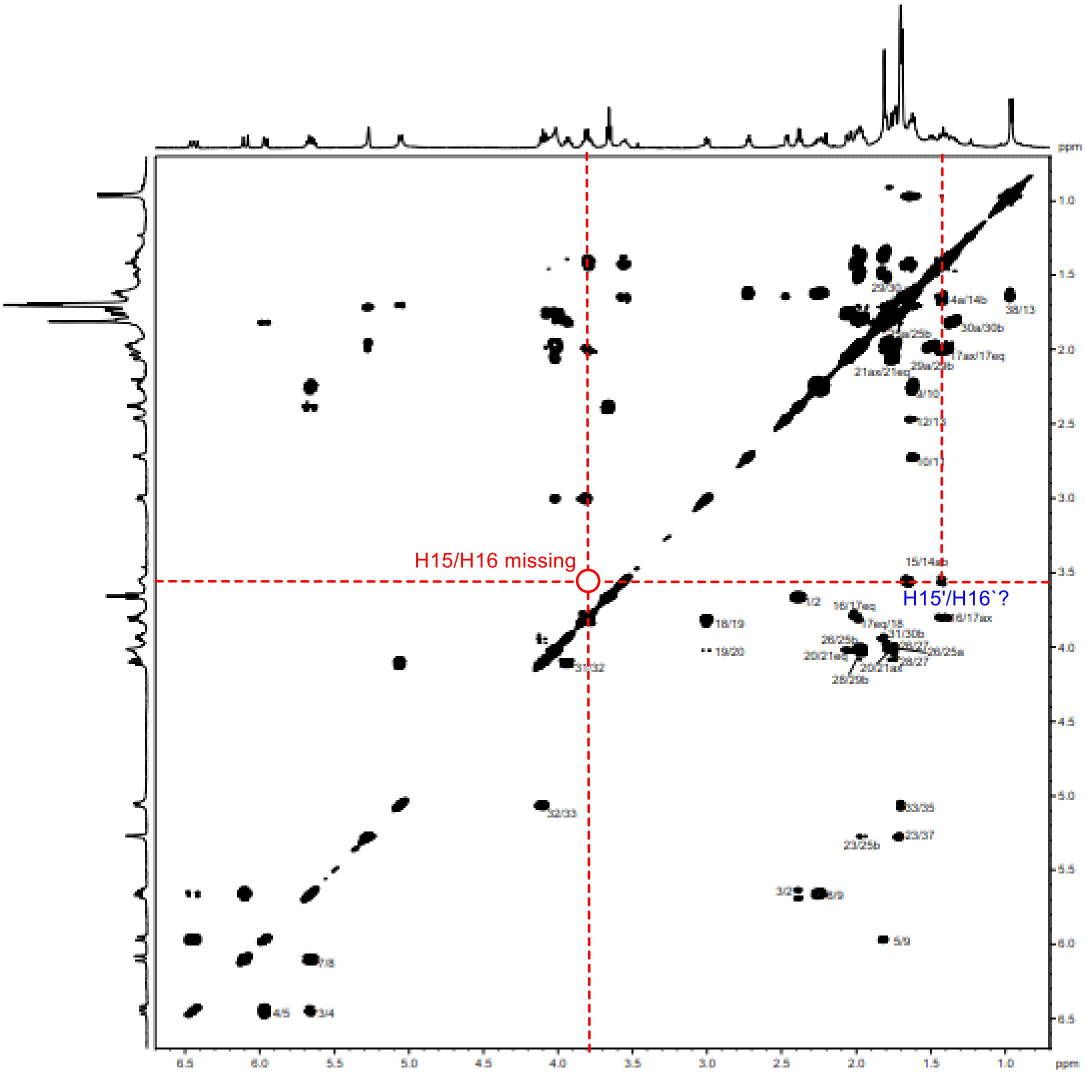

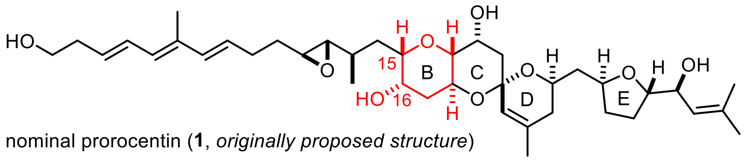

nominal prorocentin (**1**, *originally proposed structure*)

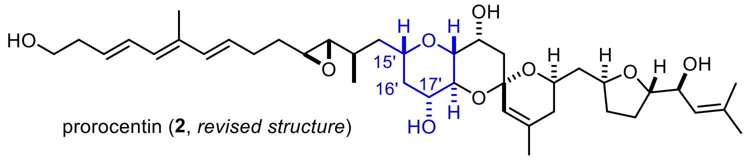

prorocentin (**2**, *revised structure*)

tabulated shifts (*Org. Lett.* **2005**, *7*, 3893)

H14: 1.42/1.65 ppm
H15: 3.55 ppm
H16: 3.80 ppm (= H17' ?)
$H17_{ax}$: 1.41 ppm (= $H16'_{ax}$?)
$H17_{eq}$: 2.00 ppm (= $H16'_{eq}$?)
H19: 3.00 ppm

Figure S3. Reevaluation of the published COSY NMR spectrum of natural prorocentin. The insert shows the originally assigned structure **1** and the most likely revised structure **2**

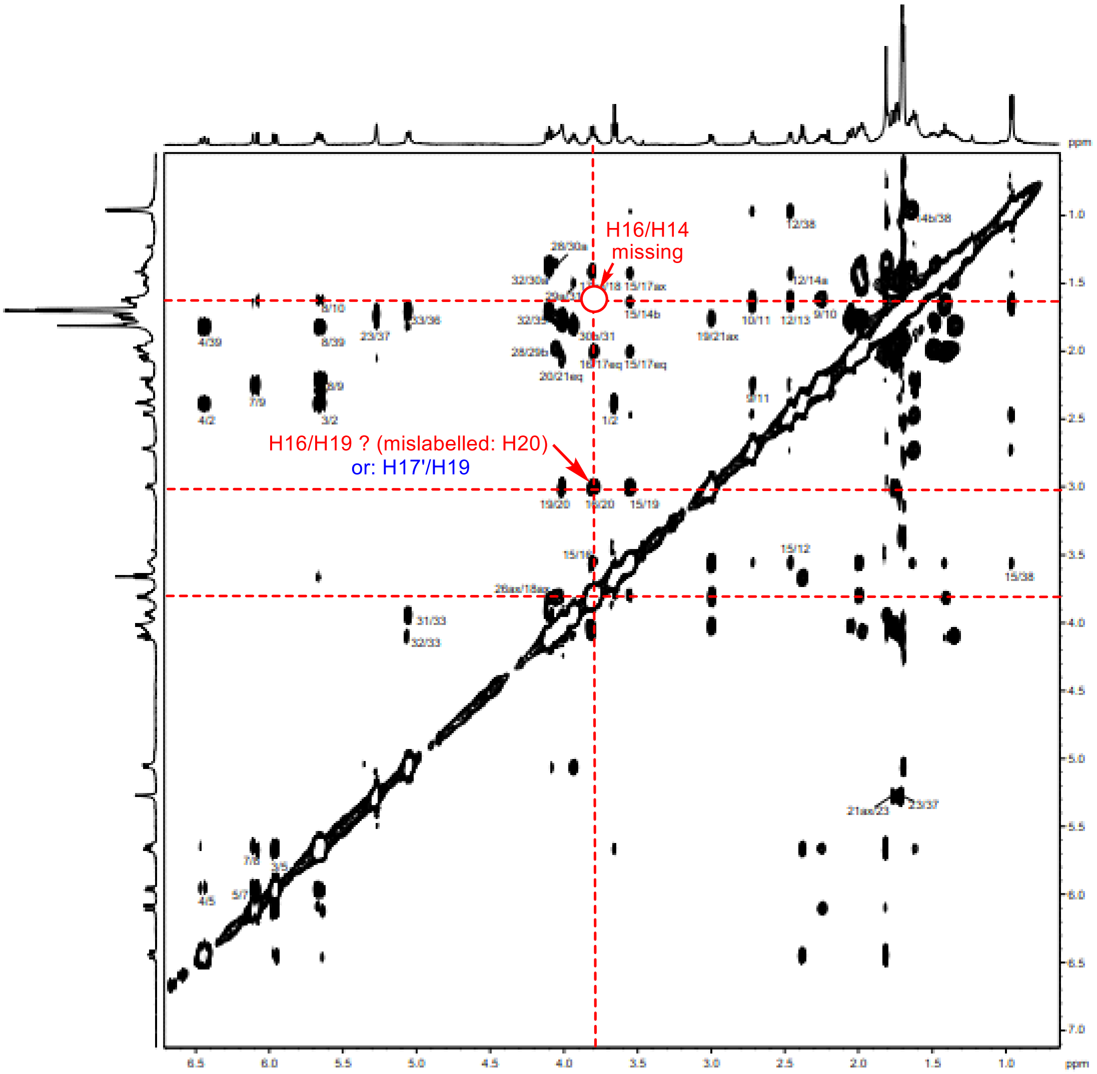

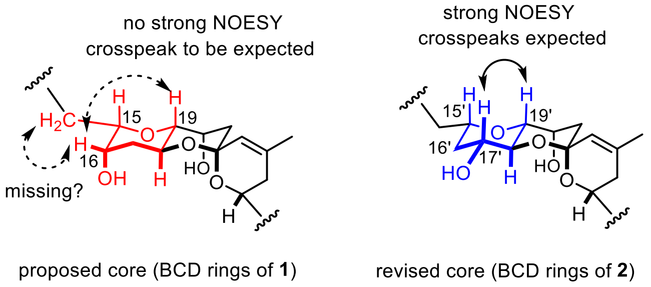

tabulated shifts (*Org. Lett.* **2005**, *7*, 3893)

H14: 1.42/1.65 ppm
H15: 3.55 ppm
H16: 3.80 ppm (= H17' ?)
$H17_{ax}$: 1.41 ppm (= $H16'_{ax}$?)
$H17_{eq}$: 2.00 ppm (= $H16'_{eq}$?)
H19: 3.00 ppm

Figure S4. Reevaluation of the NOESY NMR spectrum of natural prorocentin; note that the cross peak for the signals at δ_H = 3.80 ppm (= H16) and δ_H = 3.00 ppm (H = 19) has been erroneously labeled H16/H20 in the depicted spectrum.

Although the reassessment of the COSY and NOESY data suggested that prorocentin is more likely described by the revised structure **2**, an uncertainty arose, however, from the fact that the signal of

the axially oriented H18 in the revised structure **2** is expected to be a dd (or t) as a consequence of two large *J*-couplings to the vicinal protons, whereas the isolation paper reports for H18 a multiplet at δ_H = 3.81 ppm. The spectral region is fairly crowded: most notably, putative H16 resonates at δ_H = 3.80 ppm (m);[1] it is therefore possible that signal overlap has obscured the assignments. Anyway, these ambiguities together with the fairly small size and low resolution of the published spectra of the natural product did not allow a firm and unambiguous conclusion to be reached.

SUPPORTING COMPUTATIONAL DATA

The isolation team established only the relative configuration of the (incorrectly assigned) BCD-ring system and the relative configuration of the lateral *trans*-tetrahydrofuran segment; no firm data were provided from which the stereochemical relationship between these two sectors could be inferred.

Computational spectroscopy might allow to gain information as to the most likely stereochemical pattern. To this end, DP4+ probability calculations were performed for the following six isomers according to the protocol developed by Sarotti et al.[2,3,4] The computed chemical shielding tensors were compared with the experimental NMR data of prorocentin. Because of the high score,[5] isomer 3 was taken as the starting point for the total synthesis endeavor despite the fact that this early model study had been based on compounds featuring incorrectly configured epoxide rings.

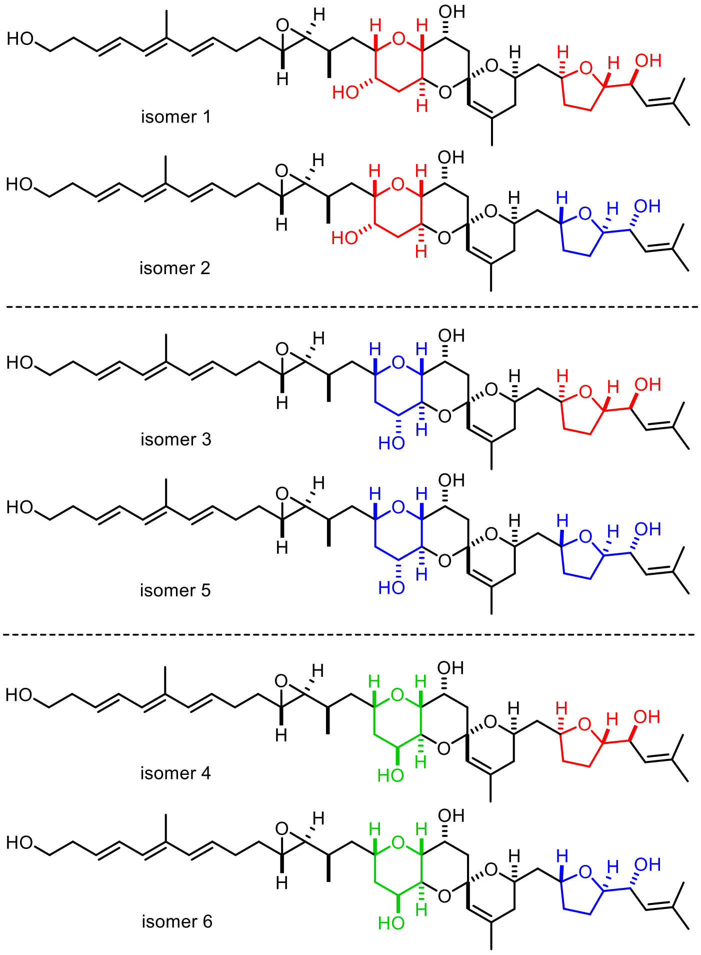

Table S2. Results of the DP4+ (128) probability calculation based on the experimental NMR data of prorocentin

Isomer	1	2	3	4	5	6
DP4+ (^{1}H data)	0.00%	1.11%	97.44%	0.00%	0.00%	1.44%
DP4+ (^{13}C data)	0.00%	0.00%	100.00%	0.00%	0.00%	0.00%

MODEL STUDY: PROGRESSION OF THE SPIROCYCLIZATION REACTION

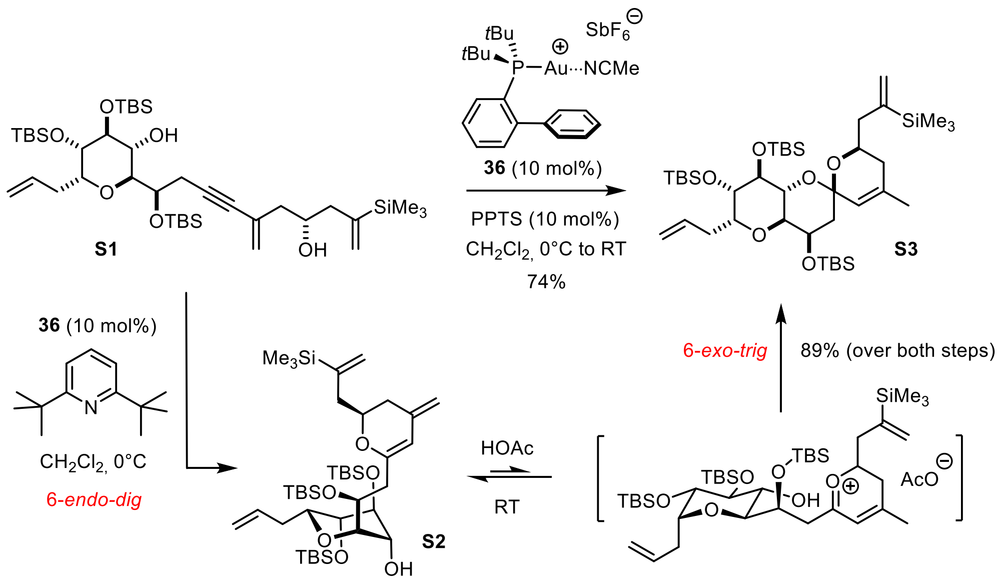

Scheme S1. One-pot versus stepwise spirocyclization of a model compound; the recorded NMR data indicate that the substrate **S1** as well as the enol ether **S2** primarily formed in the gold catalyzed step adopt a conformation in which the bulky TBS-ethers on the tetrahydropyran ring are axially disposed.

Compound S3. 2,6-Di-*tert*-butylpyridine (0.13 mL, 0.59 mmol) and (acetonitrile)[(2-biphenyl)di-*tert*-butylphosphine]gold(I) hexafluoroantimonate (**36**, 22 mg, 28 µmol) were added to a solution of enyne **S1** (0.43 g, 0.56 mmol) in CH_2Cl_2 (5.5 mL) at 0 °C. The mixture was stirred at 0 °C for 5 h and then directly filtered through Celite. The filtrate was concentrated to afford the corresponding enol ether contaminated with the residual pyridine base. Attempts at obtaining analytically pure samples by flash chromatography resulted in decomposition; however, a full data set of the enol ether **S2** could be deduced from the spectra of the crude mixture (see below).

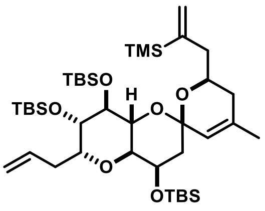

This residue was taken up in acetic acid (5.5 mL) and the mixture was stirred at room temperature for 30 min, then diluted with *tert*-butyl methyl ether (20 mL) and cooled to 0 °C. Saturated aq. $NaHCO_3$ (10 mL) was added slowly and additional solid $NaHCO_3$ was added until gas evolution had ceased. The aqueous phase was extracted with *tert*-butyl methyl ether (3 × 8 mL) and the combined organic fractions were washed with brine (10 mL), dried over anhydrous Na_2SO_4, filtered, and concentrated. The residue was purified by flash chromatography (hexanes/*tert*-butyl methyl ether 100:1) to give the title compound as a pale yellow oil (0.38 g, 89%). $[\alpha]_D^{20}$ = +44.9 (c = 1.12, $CHCl_3$); ^{1}H NMR ($CDCl_3$, 400 MHz): δ 5.82 (ddt, *J* = 17.0, 10.3, 6.7 Hz, 1H), 5.77 – 5.71 (m, 1H), 5.42 (dd, *J* = 3.0, 1.3 Hz, 1H), 5.18 (p, *J* = 1.3 Hz, 1H), 5.09 (dd, *J* = 17.3, 1.8 Hz, 1H), 5.08 – 5.00 (m, 1H), 4.15 (ddt, *J* = 10.4, 8.8, 4.5 Hz,

1H), 4.01 (q, *J* = 3.0 Hz, 1H), 3.95 – 3.82 (m, 2H), 3.67 (t, *J* = 8.4 Hz, 1H), 3.61 (dd, *J* = 8.5, 4.9 Hz, 1H), 3.28 (dd, *J* = 10.0, 2.9 Hz, 1H), 2.54 (ddt, *J* = 15.3, 5.0, 1.8 Hz, 1H), 2.47 – 2.38 (m, 2H), 2.16 (dd, *J* = 15.2, 8.3 Hz, 1H), 1.91 – 1.69 (m, 4H), 1.72 – 1.63 (m, 5H), 0.92 (s, 9H), 0.89 (s, 8H), 0.88 (s, 7H), 0.11 (s, 3H), 0.09 (s, 12H), 0.07 (s, 3H), 0.05 (s, 3H), 0.02 (s, 3H), 0.01 (s, 4H); ^{13}C NMR ($CDCl_3$, 101 MHz): δ 148.2, 135.9, 135.6, 125.7, 124.4, 116.2, 95.0, 76.5, 74.2, 73.4, 70.7, 68.7, 66.8, 66.1, 42.5, 40.9, 34.9, 29.8, 26.5, 26.4, 26.1, 22.9, 18.7, 18.6, 18.4, −1.6, −3.5, −3.5, −3.7, −4.1, −4.3, −5.1; IR (film, cm^{-1}): 2953, 2928, 2857, 1472, 1250, 1205, 1090, 1038, 968, 836, 776, 671; HRMS (ESI) for $C_{40}H_{79}O_6Si_4$ [M+H]$^+$: calcd. 767.4948; found 767.4952.

Table S3. NMR data of the enol ether **S2;** numbering scheme as shown in the insert

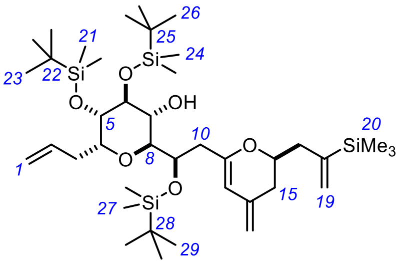

atom number	^{1}H NMR (CDCl$_3$, 500 MHz)			^{13}C NMR (CDCl$_3$, 126 MHz)	
	δ [ppm]	***J*** [Hz]	**COSY**	δ [ppm]	**HMBC**
1-*cis*	5.06	1.9 (1-*trans*), 10.3 (2)	2	116.9	
1-*trans*	5.1	17.2 (2), 1.9 (1-*cis*)	2		
2	5.82	17.2 (1-*trans*), 10.3 (1-*cis*)	1-*cis*, 1-*trans*, 3′, 3″	135.1	3′, 3″
3′	2.38	-	2, 4	32.4	1-*cis*, 1-*trans*
3″	2.26	-	2, 4		
4	3.83	9.8, 4.0, 4.0	3′, 3″, 5	72.7	5
5	3.55	5.8 (6)	4, 6	72.4	3′, 6
6	3.79	5.8 (5), 6.0 (7)	5, 7	73.3	5, 7
7	3.62	6.0 (8), 6.0 (30), 6.0 (6)	6, 8, 30	71	5, 6, 8
8	3.56	6.0 (7)	7, 9	78	7, 9, 10
9	4.35	-	8, 10′, 10″	70.7	8, 10″
10′	2.56	-	9	39.5	8, 12
10″	2.21	-	9		
11	-	-	-	154.3	10′, 10″, 12
12	5.32	-	-	104	10″, 14′, 14″
13	-	-	-	137.9	15′, 15″
14′	4.61	-	14″	105	12
14″	4.41	-	14′		
15′	2.38	-	16	34.5	12, 14′, 14″, 17′, 17″
15″	2.18	-	16		
16	4.06	-	15′, 15″, 17′, 17″	75.3	15′, 15″, 17′, 17″
17′	2.57	-	16	41.3	-
17″	2.35	-	16		
18	-	-	-	147.8	17′, 17″, 19′, 19″, 20, 20′, 20″
19′	5.65	-	19″	127.5	17′, 17″
19″	5.45	-	19′		
20	0.1	-	-	–1.38	18
21, 24, 27	0.06 – 0.14	-	-	–4.66 – –3.71	-
22, 25, 28	-	-	-	18.1 – 18.2	-
23, 26, 29	0.86 – 0.93	-	-	25.9 – 26.1	-
30	3.07	6.0 (7)	7	-	-

COMPLETION OF THE TOTAL SYNTHESIS OF NOMINAL PROROCENTIN (Full Scheme 10, Main Text)

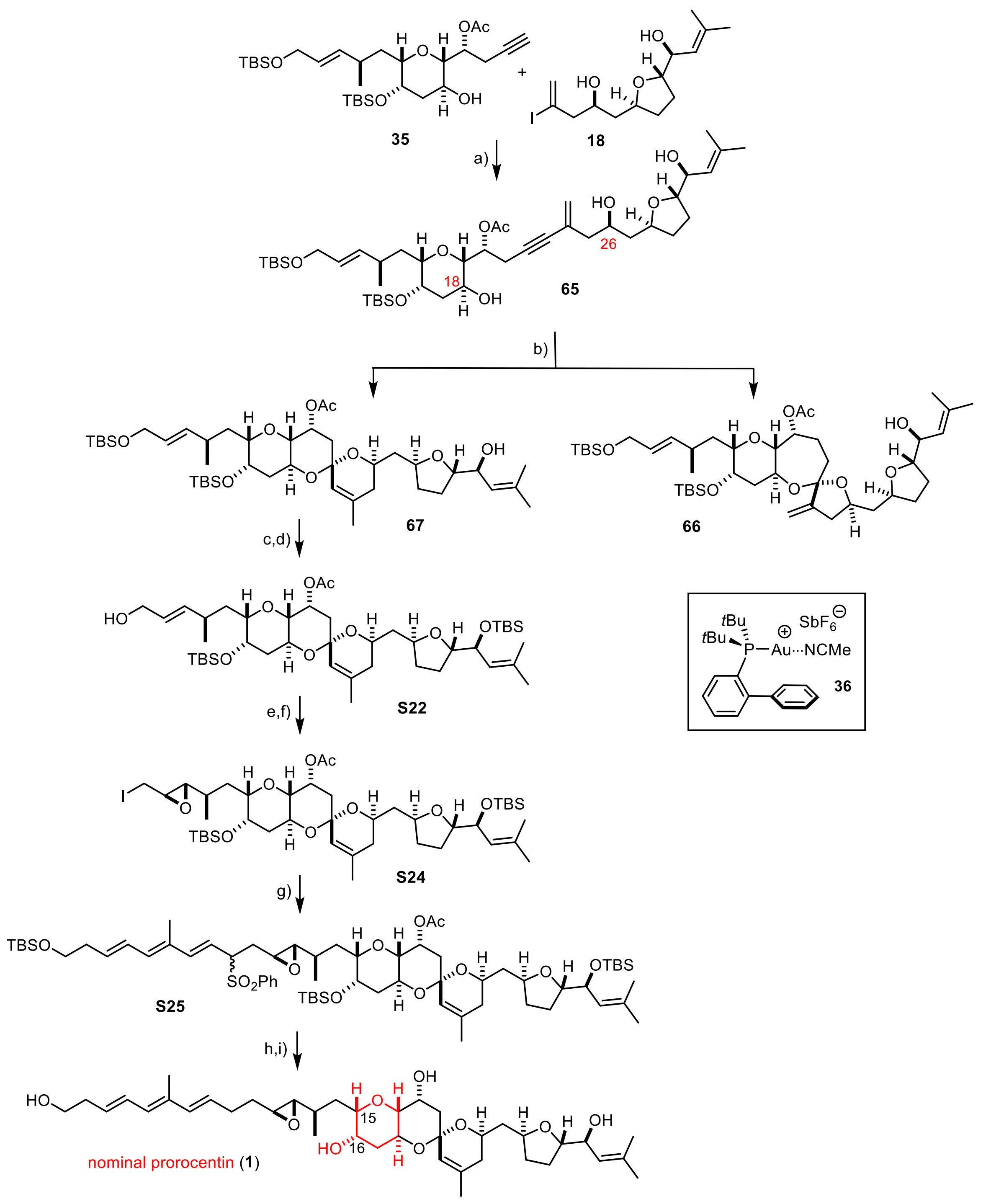

Scheme S2. (a) $Pd_2(dba)_3$ (5 mol%), CuI (20 mol%), PPh_3 (20 mol%), *i*Pr_2NEt, THF, 99%; (b) **36** (10 mol%), PPTS (10 mol%), CH_2Cl_2, **67**:**66** ≈ 10:1 (NMR), 84%; (c) TBSOTf, 2,6-lutidine, CH_2Cl_2, 0°C, 66%; (d) HF·pyridine, pyridine, 0°C to RT, 91%; (e) Ti(O*i*Pr)$_4$, L-(+)-DIPT, cumene hydroperoxide, CH_2Cl_2, –30°C, dr > 20:1, 99%; (f) I_2, PPh_3, imidazole, CH_2Cl_2, 0°C, 92%; (g) *n*BuLi, THF, DMPU, then **8**, –78°C to –60°C, dr ≈ 3:2, 79%; (h) (i) $LiBHEt_3$, THF, –20°C; (ii) [(dppp)$PdCl_2$] (10 mol%), $LiBHEt_3$, –10°C, 56%; (i) HF·pyridine, THF, pyridine, 51% (20% after HPLC).

GENERAL INFORMATION

Unless stated otherwise, all reactions were carried out under argon in flame-dried glassware, ensuring rigorously inert conditions. The solvents were purified by distillation over the indicated drying agents and were stored and handled under argon: THF, Et_2O (Mg/anthracene); hexanes, toluene (Na/K); NEt_3, diisopropylamine, diisopropylethylamine, 2,6-lutidine, pyridine, *tert*-butyl methyl ether, CH_2Cl_2, DMPU (CaH_2); MeOH (Mg; stored over 3Å MS); DMSO, DMF, 1,4-dioxane, and CH_3CN were dried by an adsorption solvent purification system based on molecular sieves.

Thin layer chromatography (TLC): Macherey-Nagel precoated plates (POLYGRAM®SIL/UV254); Flash chromatography: Merck silica gel 60 (40-63 µm or 15-40 µm - referred to as "fine silica") with pre-distilled or HPLC grade solvents.

NMR spectra were recorded on Bruker AV III 400, AV III 500, AV III 600, or AV NEO 600 spectrometers in the solvents indicated; chemical shifts (δ) are given in ppm relative to TMS, coupling constants (*J*) in Hz. The solvent signals were used as references[6] and the chemical shifts converted to the TMS scale ($CDCl_3$: δ_C = 77.16 ppm; residual $CHCl_3$: δ_H = 7.26 ppm; CD_2Cl_2: δ_C = 53.84 ppm; residual $CHDCl_2$: δ_H = 5.32 ppm; C_6D_6: δ_C = 128.06 ppm; residual C_6HD_5: δ_H = 7.16 ppm; CD_3OD: δ_C = 49.00 ppm; residual CHD_2OD: δ_H = 3.31 ppm; $D_3C(C{=}O)CD_3$: δ_C = 29.84 ppm; residual $D_3C(C{=}O)CHD_2$: δ_H = 2.05 ppm).

For the sake of comparison with the published data of prorocentin,[1] the chemical shifts of synthetic prorocentin were referenced to $CDCl_3$: δ_C = 77.00 ppm.

IR: Alpha Platinum ATR (Bruker), wavenumbers ($\tilde{\nu}$) in cm^{-1}.

MS (EI): Finnigan MAT 8200 (70 eV), DI-MS (EI): Finnigan MAT SSQ 7000, ESI-MS: ESQ 3000 (Bruker) or Thermo Scientific LTQ-FT or Thermo Scientific Exactive. HRMS: Bruker APEX III FT-MS (7 T magnet) or MAT 95 (Finnigan) or Thermo Scientific LTQ-FT or Thermo Scientific Exactive. GC-MS was measured on a Shimadzu GCMS-QP2010 Ultra instrument.

Hydrogen gas (N50, ≥99.999 Vol.%) was purchased from Air Liquide and was used without further purification. Hydrogen was handled with standard balloon techniques.

Unless stated otherwise, all commercially available compounds (abcr, Acros, TCI, Aldrich, Alfa Aesar) were used without further purification.

Light-sensitive reactions were carried out in glassware wrapped in tin foil with the fume hood light turned off.

THE WESTERN FRAGMENT

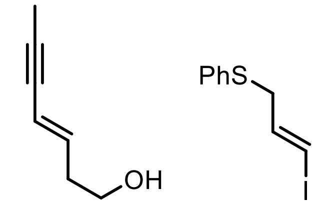

4 **6**

Compounds **4**[7] and **6**[8] were prepared according to literature procedures.

(3*E*,5*E*)-6-(Tributylstannyl)hepta-3,5-dien-1-ol (5). A solution of *n*-BuLi (1.6 M in hexanes, 14.1 mL, 22.5 mmol) was added dropwise to a solution of hexabutyldistannane (11.4 mL, 22.5 mL) in THF (14 mL) at –78 °C. The mixture was warmed to –40 °C and stirring was continued for 30 min, before the solution was cooled back to –78 °C and transferred into a suspension of CuCN (1.01 g, 11.3 mmol) in THF (12 mL), causing a color change from yellow to orange. After warming to –40 °C and additional stirring for 30 min, the solution was cooled again to –78 °C and H_2O (507 µL, 28.1 mmol) was added. The mixture was warmed to –40 °C and stirring was continued for 15 min, before it was cooled back to –78 °C and a solution of alkyne **4** (310 mg, 2.81 mmol) in THF (10 mL) was introduced. The mixture was warmed to –10 °C and stirring was continued overnight at this temperature. The reaction was quenched with brine (50 mL) and the mixture was extracted with *tert*-butyl methyl ether (3 × 50 mL). The combined organic layers were washed with brine and dried over Na_2SO_4. The residue was purified by flash chromatography (silica, pentane/*tert*-butyl methyl ether 2:1 + 1% NEt_3) to provide the title compound as a colorless oil (880 mg, 78%). ^{1}H NMR (400 MHz, CD_2Cl_2) δ 6.59 – 6.47 (m, 1H), 6.17 (ddt, *J* = 10.6, 2.5, 1.7 Hz, 1H), 5.61 (dt, *J* = 14.7, 7.2 Hz, 1H), 3.64 (q, *J* = 6.2 Hz, 2H), 2.41 – 2.31 (m, 2H), 2.06 – 1.89 (m, 3H), 1.59 – 1.26 (m, 13H), 1.02 – 0.79 (m, 15H). ^{13}C NMR (101 MHz, CD_2Cl_2) δ 142.5, 139.1, 129.8, 128.3, 62.4, 36.7, 29.6, 27.8, 19.9, 13.9, 9.4. ^{119}Sn NMR (149 MHz, CD_2Cl_2) δ –38.1. IR (film) ṽ 3323, 2956, 2924, 2871, 2852, 1463, 1376, 1045, 963, 688, 666 cm^{-1}. HRMS (ESI$^{+/-}$) calcd. for $C_{19}H_{38}OSnNa$ $[M+Na]^+$: 425.18368; found: 425.18348.

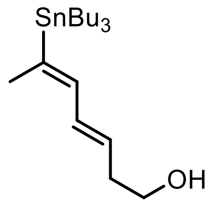

***tert*-Butyldimethyl(((3*E*,5*E*)-6-(tributylstannyl)hepta-3,5-dien-1-yl)oxy)silane (S4).** Imidazole (70.2 mg, 1.03 mmol) and DMAP (12.6 mg, 103 µmol) were added to a solution of stannane **5** (207 mg, 516 µmol) in CH_2Cl_2 (6 mL). The mixture was cooled to 0 °C and TBSCl (117 mg, 774 µmol) was added in one portion. The mixture was warmed to ambient temperature and stirring was continued for 1.5 h. The reaction was quenched upon addition of sat. aq. $NaHCO_3$. (20 mL). The layers were separated and the

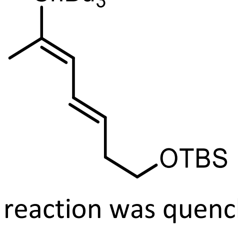

aqueous phase was extracted with CH_2Cl_2 (3 × 15 mL). The combined organic layers were washed with brine and dried over Na_2SO_4. The solvent was removed under reduced pressure and the residue was purified by flash chromatography (silica, pentane/*tert*-butyl methyl ether/NEt_3 30:1:1) to provide the title compound as a colorless oil (252 mg, 95%). ^{1}H NMR (400 MHz, CD_2Cl_2) δ 6.48 (ddt, *J* = 15.2, 10.6, 1.4 Hz, 1H), 6.15 (ddt, *J* = 10.6, 2.3, 1.6 Hz, 1H), 5.62 (dt, *J* = 14.7, 7.1 Hz, 1H), 3.66 (t, *J* = 6.7 Hz, 2H), 2.32 (qd, *J* = 6.8, 1.5 Hz, 2H), 2.04 – 1.90 (m, 3H), 1.58 – 1.40 (m, 6H), 1.38 – 1.24 (m, 6H), 1.01 – 0.80 (m, 24H), 0.05 (s, 6H). ^{13}C NMR (101 MHz, CD_2Cl_2) δ 141.6, 139.5, 130.4, 127.5, 63.4, 36.9, 29.6, 27.8, 26.1, 19.8, 18.6, 13.9, 9.4, –5.2. ^{119}Sn NMR (149 MHz, CD_2Cl_2) δ –38.2. IR (film) ṽ 2955, 2926, 2855, 1463, 1377, 1254, 1103, 962, 835, 811, 775, 671, 665 cm^{-1}. HRMS (ESI$^+$) calcd. for $C_{25}H_{52}OSiSnNa$ [M+Na]$^+$: 539.27016; found: 539.27035.

Compound 8. A pre-dried Schlenk tube charged with tetrabutylammonium diphenylphosphinate

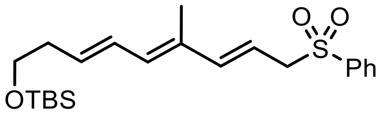

(220 mg, 479 µmol) was flame-dried under high vacuum for a few seconds until the solid material melted. Upon reaching ambient temperature, the atmosphere was exchanged for Ar and degassed DMF (3.4 mL) was added. Stannane **S4** (150 mg, 291 µmol), alkenyl iodide **6** (85.8 mg, 311 µmol), copper thiophene-2-carboxylate (CuTC, 86.3 mg, 453 µmol) and $Pd(PPh_3)_4$ (33.6 mg, 29 µmol) were added and the resulting mixture stirred for 1 h. The reaction was quenched with H_2O (5 mL) and the mixture was filtered through a plug of Celite, which was carefully rinsed with Et_2O (25 mL). The layers were separated and the aqueous phase was extracted with Et_2O (3 × 15 mL). The combined organic layers were washed with brine and dried over Na_2SO_4. The solvent was removed under reduced pressure and the residue was filtered through a short silica plug, which was rinsed with hexanes/*tert*-butyl methyl ether (20:1, 50 mL). The combined filtrate was concentrated to afford the crude thioether **7** as a mixture of double bond isomers (*E/Z* ≈ 10:1, ^{1}H NMR), which was used in the next step without further purification. Characteristic data: ^{1}H NMR (400 MHz, CD_2Cl_2) δ 7.38 – 7.15 (m, 5H), 6.41 (ddt, *J* = 15.1, 11.1, 1.4 Hz, 1H), 6.15 (dd, *J* = 15.4, 1.0 Hz, 1H), 6.01 – 5.93 (m, 1H), 5.72 (ddt, *J* = 15.0, 10.9, 7.2 Hz, 2H), 3.70 – 3.62 (m, 4H), 2.37 – 2.29 (m, 2H), 1.80 (d, *J* = 1.1 Hz, 3H), 0.89 (s, 9H), 0.04 (s, 6H).

The crude material was dissolved in MeOH (2 mL) and benzene (1.2 mL), before $Na_2WO_4{\cdot}H_2O$ (24.0 mg, 73 µmol) was added. The mixture was cooled to 0 °C and a solution of H_2O_2 (35% *w/w* in water, 141 µL, 1.46 mmol) was added dropwise. After 40 min at 0°C, stirring was continued at ambient temperature overnight. The mixture was extracted with EtOAc (3 × 15 mL), the combined organic layers were washed with brine and dried over Na_2SO_4. The solvent was removed under reduced pressure and the residue was purified by flash chromatography (fine silica, hexanes/NEt_3 5:1) to provide the title compound as a colorless oil (79 mg, 63% over two steps). ^{1}H NMR (400 MHz, CD_2Cl_2) δ 7.87 – 7.79 (m, 2H), 7.70 – 7.61 (m, 1H), 7.60 – 7.51 (m, 2H), 6.40 (ddt, *J* = 15.1, 11.1, 1.4 Hz, 1H), 6.05 (dq, *J* = 15.6,

1.1 Hz, 1H), 5.95 (d, *J* = 11.1 Hz, 1H), 5.78 (dt, *J* = 14.7, 7.2 Hz, 1H), 5.50 (dt, *J* = 15.4, 7.6 Hz, 1H), 3.85 (dd, *J* = 7.6, 1.1 Hz, 2H), 3.66 (t, *J* = 6.5 Hz, 2H), 2.40 – 2.29 (m, 2H), 1.80 (d, *J* = 1.3 Hz, 3H), 0.88 (s, 9H), 0.04 (s, 6H). ^{13}C NMR (101 MHz, CD_2Cl_2) δ 144.0, 139.1, 134.3, 134.0, 133.6, 132.5, 129.4, 128.8, 128.5, 113.6, 63.1, 61.0, 37.1, 26.0, 18.6, 12.6, –5.2. IR (film) ṽ 2954, 2927, 2856, 1320, 1308, 1254, 1153, 1137, 1087, 968, 835, 775, 735, 689, 532 cm^{-1}. HRMS (ESI$^+$) calcd. for $C_{22}H_{34}O_3SSiNa$ [M+Na]$^+$: 429.189015; found: 429.188980.

THE EASTERN FRAGMENT

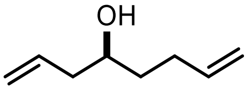

(*S*)-Octa-1,7-dien-4-ol (10). Two flame-dried pressure-Schlenk flasks were charged each with bis-(1,5-cyclooctadiene)-diiridium(I)-dichloride (390 mg, 580 µmol), (*R*)-Cl-MeO-BIPHEP (**20**) (757 mg, 1.16 mmol), 4-Cl-3-MeO-benzoic acid (468 mg, 2.32 mmol), cesium carbonate (1.51 g, 4.65 mmol) and 4-pentenol (**9**) (2.4 mL, 23.2 mmol). THF (110 mL) and (after stirring for 2 min) allyl acetate (25.0 mL, 232 mmol) were successively added to each flask. The flasks were sealed and the resulting mixtures were stirred at 100 °C for 3 d. After cooling to ambient temperature, the contents of the two flasks were combined and the solvent was carefully evaporated (≤ 35 °C bath temperature, 250 mbar). The residue was purified by flash chromatography (silica, pentane/Et_2O 9:1 → 7:1 → 2:1) to give the title compound as a colorless liquid (4.78 g, 81%, 96% *ee* (determined at the stage of the derived p-nitrobezoate, see below). $[\alpha]_{20}^{D}$ = –11.7 (c = 1.10, $CHCl_3$). ^{1}H NMR (400 MHz, $CDCl_3$) δ 5.93 – 5.73 (m, 2H), 5.17 – 4.94 (m, 4H), 3.72 – 3.61 (m, 1H), 2.30 (dddt, *J* = 13.7, 6.8, 4.4, 1.3 Hz, 1H), 2.25 – 2.08 (m, 3H), 1.74 (sbr, 1H, OH), 1.60 – 1.52 (m, 2H). ^{13}C NMR (101 MHz, $CDCl_3$) δ 138.6, 134.8, 118.3, 114.9, 70.3, 42.1, 36.0, 30.1. IR (film) ṽ 3357, 3077, 2978, 2932, 2854, 1641, 1341, 1120, 1076, 994, 912, 859, 637 cm^{-1}. HRMS (EI) calcd. for $C_8H_{14}O$ [M]$^+$: 126.10392; found: 126.10383.

The absolute configuration was determined by Mosher ester analysis:

Preparation of the (*S*)- and (*R*)-MTPA Esters of Alcohol 10. (*R*)-(–)-MTPA-Cl (25 µL, 134 µmol) was added to a solution of alcohol **10** (15.2 mg, 97.6 µmol), NEt_3 (40 µL, 287 µmol) and DMAP (3.0 mg, 24.6 µmol) in CH_2Cl_2 (1 mL) and stirring was continued overnight. *tert*-Butyl methyl ether (10 mL) was added and the mixture was washed with sat. aq. $NaHCO_3$ (2 × 10 mL), HCl (1 M, 2 × 10 mL) and brine (10 mL). The organic phase was dried over $MgSO_4$ and the solvent was removed under reduced pressure to provide the corresponding (*S*)-MTPA ester as a colorless oil (25.1 mg, 75%). ^{1}H NMR (400 MHz, $CDCl_3$) δ 7.59 – 7.52 (m, 2H), 7.43 – 7.37 (m, 3H), 5.83 – 5.64 (m, 2H), 5.21 – 5.08 (m, 3H), 4.99 – 4.91 (m, 2H), 3.56 (s, 3H), 2.44 (ddt, *J* = 7.3, 5.9, 1.3 Hz, 2H), 2.05 – 1.87 (m, 2H), 1.77 – 1.62 (m, 2H).

The (*R*)-MTPA ester was prepared analogously using (*S*)-(+)-MTPA-Cl as the reagent. ^{1}H NMR (400 MHz, $CDCl_3$) δ 7.58 – 7.50 (m, 2H), 7.43 – 7.37 (m, 3H), 5.77 (ddt, *J* = 16.9, 10.2, 6.6 Hz, 1H), 5.71 – 5.58 (m,

1H), 5.16 (dq, J = 7.6, 5.7 Hz, 1H), 5.07 – 4.96 (m, 4H), 3.55 (s, 3H), 2.37 (ddq, J = 7.4, 6.1, 1.3 Hz, 2H), 2.16 – 2.02 (m, 2H), 1.85 – 1.64 (m, 2H).

Table S4. Determination of absolute configuration of the alcohol center in **10** via Mosher ester analysis.[9] The recorded NMR data ($CDCl_3$) suggest that the chiral center C4 is *(S)*-configured.

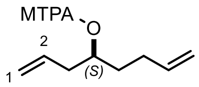

Atom number	(*S*)-ester δ [ppm]	(*R*)-ester δ [ppm]	$\Delta\delta^{S-R}$ [ppm]
1	5.15	5.01	+0.14
	4.95		-0.06
2	5.74	5.64	+0.10
3	2.44	2.37	+0.07
4	5.15	5.16	-0.01
5	1.69	1.73	-0.04
6	1.96	2.10	-0.14
7	5.74	5.77	-0.03
8	5.15	5.01	+0.14
	4.95		-0.06

(*S*)-Octa-1,7-dien-4-yl 4-nitrobenzoate (S5). 4-Nitrobenzoyl chloride (53.0 mg, 290 µmol) was added to a solution of alcohol **10** (18.0 mg, 140 µmol), DMAP (4.3 mg, 40 µmol) and Et_3N (40.0 µL, 290 µmol) in CH_2Cl_2 (1.4 mL). The mixture was stirred for 1 h before the reaction was quenched with sat. aq. NH_4Cl (5 mL). The layers were separated and the aqueous phase was extracted with *tert*-butyl methyl ether (3 × 5 mL). The combined organic extracts were dried over $MgSO_4$ and concentrated. The residue was purified by flash chromatography (silica, hexanes/*tert*-butyl methyl ether 50:1) to give the title compound as a pale yellow oil (30.9 mg , 79%, 96% *ee*). ^{1}H NMR (400 MHz, $CDCl_3$) δ 8.33 – 8.23 (m, 2H), 8.24 – 8.16 (m, 2H), 5.87 – 5.73 (m, 2H), 5.29 – 5.18 (m, 1H), 5.16 – 5.06 (m, 2H), 5.05 – 4.94 (m, 2H), 2.48 (tdt, J = 6.4, 2.6, 1.3 Hz, 2H), 2.15 (dtdt, J = 8.2, 6.8, 4.3, 1.4 Hz, 2H), 1.93 – 1.74 (m, 2H). ^{13}C NMR (101 MHz, $CDCl_3$) δ 164.4, 150.6, 137.5, 136.1, 133.2, 130.8, 123.7, 118.5, 115.5, 74.9, 38.8, 32.9, 29.8. IR (film) ṽ 3079, 2979, 2943, 2862, 1722, 1642, 1607, 1528, 1349, 1273, 1117, 1103, 1015, 994, 918, 874, 836, 784, 720 cm^{-1}. HRMS (ESI$^+$) calcd. for $C_{15}H_{17}NO_4Na$ [M+Na]$^+$: 298.10498; found: 298.10459.

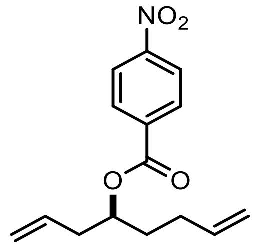

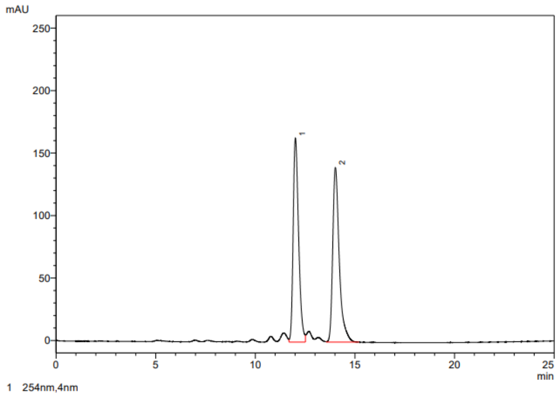

PDA Ch1 254nm

Peak #	Ret. Time	Area %	Name
1	12,02	49,37	1. Enantiomer
2	14,02	50,63	2. Enantiomer
Total		100,00	

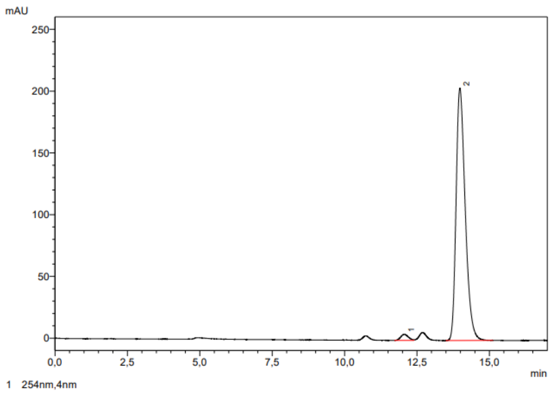

PDA Ch1 254nm

Peak #	Ret. Time	Area %	Name
1	12,07	1,99	1. Enantiomer
2	13,98	98,01	2. Enantiomer 96.0 % ee
Total		100,00	

Figure S5. HPLC traces of racemic and enantiomerically enriched **S5**. Column: 150 mm Chiralpak IG-3, 3 µm, 4.6 mm ∅; n-heptane/2-propanol = 99.5:0.5 (*v/v*), flow: 1.0 mL/min at 298 K.

((2*S*,5*S*)-5-Allyltetrahydrofuran-2-yl)methanol (11). Co(nmp)$_2$ (**21**, 1.85 g, 3.30 mmol) was added to a solution of alcohol **10** (4.11 g, 32.6 mmol) in *i*-PrOH (160 mL). O_2 was bubbled through the solution for 15 min before *t*-butyl hydroperoxide (5.5 M in decane, 0.6 mL 3.3 mmol) was added. The mixture was stirred at 55 °C for 17 h under O_2 atmosphere (balloon). After cooling to ambient temperature, the solvent was carefully evaporated (35 °C water bath temperature, 100 mbar) and the residue was purified by flash chromatography (silica, pentane/Et_2O 4:1 → 2:1 → 1.1) to provide the title compound as a colorless oil (3.36 g, 72%, *dr* >20:1). $[\alpha]_D^{20}$ = +7.3 (*c* = 0.06, $CHCl_3$). ^1H NMR (400 MHz, $CDCl_3$) δ 5.81 (ddt, *J* = 17.2, 10.2, 7.0 Hz, 1H), 5.15 – 5.00 (m, 2H), 4.13 (dtd, *J* = 7.5, 6.3, 3.3 Hz, 1H), 4.03 (dq, *J* = 7.9, 6.1 Hz, 1H), 3.64 (dd, *J* = 11.5, 3.3 Hz, 1H), 3.49 (dd, *J* = 11.5, 6.1 Hz, 1H), 2.37 (dddt, *J* = 14.4, 7.3, 6.2, 1.4 Hz, 1H), 2.30 – 2.18 (m, 1H), 2.06 – 1.93 (m, 3H), 1.74 – 1.54 (m, 2H). ^{13}C NMR (101 MHz, $CDCl_3$) δ 134.9, 117.1, 79.3, 78.8, 65.1, 40.2, 31.6, 27.5. IR (film) ṽ 3411, 3077, 2970, 2928, 2871, 1642, 1444, 1370, 1040, 996, 913, 881, 684, 612 cm^{-1}. HRMS (ESI$^+$) calcd. for $C_8H_{15}O_2$ [M+H]$^+$: 143.10666; found: 143.10683.

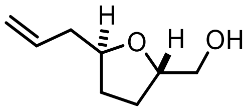

(2*S*,5*S*)-5-Allyltetrahydrofuran-2-carboxylic acid (S6). H_2O (4.2 mL, 233 mmol), TEMPO (1.09 g, 6.96 mmol) and diacetoxy iodobenzene (16.4 g, 51.1 mmol) were added to a solution of alcohol **11** (3.30 g, 23.2 mmol) in MeCN (46 mL). The mixture was stirred for 3 h before sat. aq. NH_4Cl (100 mL) was introduced. The mixture was extracted with *tert*-butyl methyl ether (3 × 100 mL) and the combined organic extracts were dried over $MgSO_4$ and concentrated. The residue was purified by flash chromatography (silica, hexanes/*tert*-butyl methyl ether 7:1 + 1% AcOH → 2:1 + 1% AcOH) to give the title compound as a colorless oil (3.11 g, 86%). $[\alpha]_D^{20}$ = −45.0 (c = 1.10, $CHCl_3$). ^1H NMR (400 MHz, $CDCl_3$) δ 9.59 (br. s., 1H), 5.80 (ddt, *J* = 17.2,

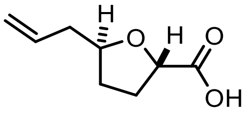

10.3, 7.0 Hz, 1H), 5.15 – 5.06 (m, 2H), 4.55 (dd, *J* = 8.2, 5.9 Hz, 1H), 4.24 (dq, *J* = 7.6, 6.0 Hz, 1H), 2.44 – 2.34 (m, 2H), 2.28 (dtt, *J* = 14.3, 6.6, 1.3 Hz, 1H), 2.16 – 1.97 (m, 2H), 1.72 – 1.57 (m, 1H). ^{13}C NMR (101 MHz, $CDCl_3$) δ 177.4, 134.1, 117.7, 80.6, 76.6, 39.6, 30.5, 30.1. IR (film) ṽ 3438, 3077, 2978, 2934, 1722, 1642, 1433, 1358, 1192, 1083, 998, 918, 878, 763, 658 cm^{-1}. HRMS (ESI$^-$) calcd. for $C_8H_{11}O_2$ $[M-H]^-$: 155.07137; found: 155.07139.

(*2S,5S*)-5-Allyl-N-methoxy-N-methyltetrahydrofuran-2-carboxamide (12). 1,1'-Carbonyldiimidazole (4.36 g, 26.9 mmol) was added to a solution of carboxylic acid **S6** (2.80 g, 17.9 mmol) in CH_2Cl_2 (90 mL) at 0 °C. The mixture was stirred at ambient for 1 h. The mixture was cooled to 0 °C before *N,O*-dimethylhydroxylamine hydrochloride (3.50 g, 35.9 mmol) was added. Stirring was continued at ambient temperature overnight. Sat. aq. NH_4Cl (100 mL) was introduced, the layers were separated and the aqueous phase was extracted with *tert*-butyl methyl ether (2 × 100 mL). The combined organic layers were dried over $MgSO_4$ and concentrated. The residue was purified by flash chromatography (silica, hexanes/EtOAc 3:1 → 2:1) to provide the title compound as a colorless oil (3.19 g, 89%). $[\alpha]_D^{20}$ = −15.0 (c = 1.00, $CHCl_3$). ^{1}H NMR (400 MHz, $CDCl_3$) δ 5.81 (ddt, *J* = 17.2, 10.2, 6.9 Hz, 1H), 5.14 – 5.00 (m, 2H), 4.89 – 4.81 (m, 1H), 4.25 (dq, *J* = 8.0, 6.0 Hz, 1H), 3.69 (s, 3H), 3.17 (s, 3H), 2.41 (dddt, *J* = 14.2, 7.1, 5.8, 1.4 Hz, 1H), 2.32 – 2.13 (m, 2H), 2.11 – 1.95 (m, 2H), 1.62 – 1.50 (m, 1H). ^{13}C NMR (101 MHz, $CDCl_3$) δ 174.2, 134.7, 117.0, 79.8, 75.2, 61.4, 39.8, 32.4, 30.7, 29.4. IR (film) ṽ 3077, 2975, 2937, 1775, 1668, 1462, 1443, 1386, 1317, 1177, 1069, 995, 916, 831, 711, 613, 494, 436 cm^{-1}. HRMS (ESI$^+$) calcd. for $C_{10}H_{17}NO_3Na$ $[M+Na]^+$: 222.11006; found: 222.11020.

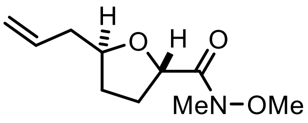

1-((*2S,5S*)-5-Allyltetrahydrofuran-2-yl)-3-methylbut-2-en-1-one (13). 2-Methyl-1-propenylmagnesium bromide (0.5 M in THF, 26 mL, 13.0 mmol) was added to a solution of amide **12** (2.11 g, 10.6 mmol) in THF (35 mL) at 0 °C. Stirring was continued for 2 h before sat. aq. NH_4Cl (75 mL) was added. The mixture was extracted with *tert*-butyl methyl ether (3 × 75 mL), the combined extracts were dried over $MgSO_4$, and the solvent was removed under reduced pressure. The residue was dissolved in CH_2Cl_2 (45 mL) and DBU (320 µL, 2.14 mmol) was added. After stirring for 1 h, sat. aq. NH_4Cl (75 mL) was introduced. The layers were separated and the aqueous phase was extracted with *tert*-butyl methyl ether (2 × 75 mL). The combined organic phases were washed with brine (75 mL) and dried over $MgSO_4$ to provide the title compound as a pale yellow oil (2.01 g, 98%). $[\alpha]_D^{20}$ = −53.5 (c = 1.00, $CHCl_3$). ^{1}H NMR (400 MHz, $CDCl_3$) δ 6.31 (hept, *J* = 1.3 Hz, 1H), 5.82 (ddt, *J* = 17.2, 10.2, 6.9 Hz, 1H), 5.19 – 5.01 (m, 2H), 4.40 (dd, *J* = 7.9, 6.8 Hz, 1H), 4.15 (dq, *J* = 7.9, 6.1 Hz, 1H), 2.42 (dddt, *J* = 14.2, 7.1, 5.9, 1.4 Hz, 1H), 2.28 (ddt, *J* = 14.3, 7.4, 1.4 Hz, 1H), 2.25 – 2.18 (m, 1H), 2.17 (d, *J* = 1.3 Hz, 3H), 2.02 – 1.95 (m, 1H), 1.94 – 1.88 (m, 4H), 1.63 – 1.54 (m, 1H). ^{13}C NMR (101 MHz, $CDCl_3$) δ 201.7, 158.5, 134.6, 119.5, 117.2, 83.8, 80.0, 39.9,

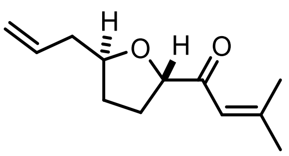

30.9, 29.4, 28.3, 21.2. IR (film) ṽ 3077, 2976, 2933, 2873, 1684, 1615, 1443, 1378, 1229, 1072, 1030, 997, 915, 883, 844, 699, 659, 563, 464. cm^{-1}. HRMS (EI) calcd. for $C_{12}H_{18}O_2$ $[M]^+$: 194.13013; found: 194.12997.

(*S*)-1-((*2S,5S*)-5-Allyltetrahydrofuran-2-yl)-3-methylbut-2-en-1-ol (**S7**). Cerium chloride heptahydrate

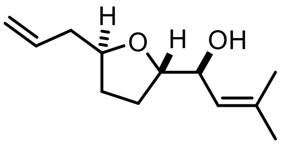

(5.75 g, 15.4 mmol) was added to a solution of ketone **13** (2.00 g, 10.3 mmol) in MeOH (100 mL) and the resulting mixture was stirred for 30 min. After cooling to −78 °C, $NaBH_4$ (779 mg, 20.6 mmol) was added in one portion and stirring was continued at this temperature for 45 min. The reaction was quenched upon careful addition of sat. aq. NH_4Cl (100 mL). The mixture was extracted with *tert*-butyl methyl ether (3 × 100 mL), the combined extracts were washed with water (100 mL) and brine (50 mL) and were dried over $MgSO_4$. The solvent was removed under reduced pressure to provide the title compound as a pale yellow oil (1.91 g, 94%, *dr* >20:1) $[\alpha]_D^{20}$ = +14.8 (c = 0.30, $CHCl_3$). ^{1}H NMR (400 MHz, $CDCl_3$) δ 5.81 (ddt, *J* = 17.2, 10.2, 7.0 Hz, 1H), 5.16 – 4.99 (m, 3H), 4.14 (dd, *J* = 9.0, 7.7 Hz, 1H), 4.03 (pd, *J* = 6.1, 1.3 Hz, 1H), 3.92 – 3.81 (m, 1H), 2.38 (dddt, *J* = 14.2, 7.2, 6.0, 1.4 Hz, 1H), 2.23 (dtt, *J* = 13.9, 6.8, 1.3 Hz, 1H), 2.04 – 1.96 (m, 2H), 1.94 – 1.83 (m, 1H), 1.74 (d, *J* = 1.4 Hz, 3H), 1.73 (d, *J* = 1.4 Hz, 3H), 1.66 – 1.47 (m, 2H). ^{13}C NMR (101 MHz, $CDCl_3$) δ 138.0, 134.9, 123.5, 117.1, 82.7, 78.6, 71.3, 40.1, 31.5, 27.9, 26.1, 18.8. IR (film) ṽ 3446, 3076, 2972, 2913, 1642, 1445, 1376, 1062, 1025, 914 cm^{-1}. HRMS (ESI$^+$) calcd. for $C_{12}H_{20}O_2Na$ $[M+Na]^+$: 219.13555; found: 219.13563.

The absolute configuration was determined by Mosher ester analysis:

Preparation of the (*S*)- and (*R*)-MTPA Esters of Alcohol S7. (*R*)-(–)-MTPA-Cl (15 µL, 80.1 µmol) was added to a solution of alcohol **S7** (12.1 mg, 61.6 µmol), NEt_3 (26 µL, 187 µmol) and DMAP (1.9 mg, 15.6 µmol) in CH_2Cl_2 (0.7 mL) and stirring was continued overnight. *tert*-Butyl methyl ether (10 mL) was added and the mixture was washed with sat. aq. $NaHCO_3$ (2 × 10 mL), HCl (1 M, 2 × 10 mL) and brine (10 mL). The organic layer was dried over $MgSO_4$ and the solvent was removed under reduced pressure to provide the corresponding (*S*)-MTPA ester as a colorless oil (21.3 mg, 84%). ^{1}H NMR (400 MHz, $CDCl_3$) δ 7.62 – 7.55 (m, 2H), 7.41 – 7.33 (m, 4H), 5.79 (ddt, *J* = 17.1, 10.2, 6.9 Hz, 1H), 5.65 (dd, *J* = 9.6, 8.1 Hz, 1H), 5.11 – 4.98 (m, 3H), 4.16 – 4.08 (m, 1H), 4.04 (dq, *J* = 8.2, 6.1 Hz, 1H), 3.61 (s, 3H), 2.36 – 2.19 (m, 2H), 2.02 – 1.90 (m, 2H), 1.80 (d, *J* = 1.4 Hz, 3H), 1.72 (d, *J* = 1.4 Hz, 3H), 1.59 – 1.50 (m, 2H).

The (*R*)-MTPA ester was prepared analogously using (*S*)-(+)-MTPA-Cl as the reagent. ^{1}H NMR (400 MHz, $CDCl_3$) δ 7.62 – 7.56 (m, 2H), 7.41 – 7.34 (m, 3H), 5.83 – 5.72 (m, 1H), 5.69 (dd, *J* = 9.7, 6.7 Hz, 1H), 5.23 (dp, *J* = 9.6, 1.4 Hz, 1H), 5.09 – 4.97 (m, 2H), 4.07 (q, *J* = 6.8 Hz, 1H), 3.97 – 3.89 (m, 1H), 3.55 (s, 3H), 2.33 – 2.25 (m, 1H), 2.21 – 2.13 (m, 1H), 1.93 – 1.85 (m, 2H), 1.80 (d, *J* = 1.4 Hz, 3H), 1.77 (d, *J* = 1.5 Hz, 3H), 1.54 – 1.42 (m, 2H).

Table S5. Determination of absolute configuration of the newly set chiral center in **S7** via Mosher ester analysis.[9] The recorded NMR data ($CDCl_3$) suggest that the chiral center C8 is *(S)*-configured. Arbitrary numbering shown in the insert.

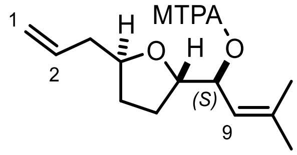

Atom number	(*S*)-ester δ [ppm]	(*R*)-ester δ [ppm]	$\Delta\delta^{S-R}$ [ppm]
1	5.03	5.03	0.00
2	5.79	5.77	+0.02
3	2.27	2.29	-0.02
		2.17	+0.10
4	4.04	3.93	+0.11
5/6	1.96	1.89	+0.07
	1.54	1.48	+0.06
7	4.11	4.07	+0.04
8	5.65	5.69	-0.04
9	5.79	5.23	+0.56
10/11	1.86	1.80	+0.06
	1.72	1.77	-0.05

(*2S,5S*)-2-Allyl-5-((*S*)-3-methyl-1-(naphthalen-2-ylmethoxy)but-2-en-1-yl)tetrahydrofuran (14). A

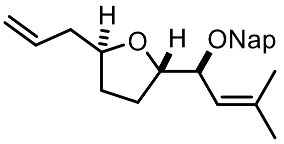

solution of alcohol **S7** (625 mg, 3.18 mmol) in THF (10 mL) was added to a mixture of 2-(bromomethyl)-naphthalene (1.41 g, 6.37 mmol) and tetrabutylammonium iodide (235 mg, 0.64 mmol). DMF (5 mL) and NaH (153 mg, 6.37 mmol) were added and stirring was continued overnight. Sat. aq. NH_4Cl (50 mL) was introduced, the mixture was extracted with *tert*-butyl methyl ether (3 × 50 mL), and the combined organic layers were dried over $MgSO_4$. The solvent was removed under reduced pressure and the residue was purified by flash chromatography (silica, hexanes/*tert*-butyl methyl ether 97:3 → 95:5) to provide the title compound as a colorless oil (964 mg, 90%). $[\alpha]_D^{20}$ = +33.5 (c = 0.50, $CHCl_3$). ^1H NMR (400 MHz, $CDCl_3$) δ 7.81 (m, 4H), 7.53 – 7.40 (m, 3H), 5.84 (ddt, *J* = 17.2, 10.2, 7.0 Hz, 1H), 5.22 – 5.01 (m, 3H), 4.80 (d, *J* = 13.3 Hz, 1H), 4.59 (d, *J* = 12.8 Hz, 1H), 4.11 (q, *J* = 6.8 Hz, 1H), 4.06 – 3.95 (m, 2H), 2.44 (dddt, *J* = 13.9, 7.0, 5.6, 1.4 Hz, 1H), 2.24 (dtt, *J* = 14.0, 7.1, 1.3 Hz, 1H), 2.00 – 1.83 (m, 2H), 1.78 (d, *J* = 1.4 Hz, 3H), 1.68 – 1.57 (m, 4H), 1.56 – 1.47 (m, 1H). ^{13}C NMR (101 MHz, $CDCl_3$) δ 138.0, 136.9, 135.3, 133.5, 133.0, 128.0, 127.8, 126.3, 126.1, 126.0, 125.7, 122.7, 116.8, 81.6, 78.9, 77.4, 69.7, 40.3, 31.3, 28.2, 26.2, 18.9. IR (film) ṽ 3056, 2973, 2913, 2864, 1444, 1376, 1067, 914, 854, 816, 752. cm^{-1}. HRMS (ESI^+) calcd. for $C_{23}H_{28}O_2Na$ $[M+Na]^+$: 359.19815; found: 359.19829.

(*S*)-3-((*2S,5S*)-5-((*S*)-3-Methyl-1-(naphthalen-2-ylmethoxy)but-2-en-1-yl)tetrahydrofuran-2-yl)-propane-1,2-diol (15).

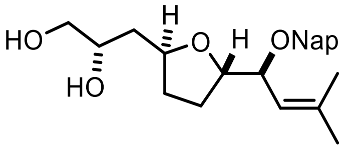

A mixture of Pt(dba)$_3$ (170 mg, 0.19 mmol), ligand **(*R,R*)-22** (228 mg, 0.25 mmol) and B_2(pin)$_2$ (2.08 g, 8.19 mmol) were dried in high vacuum (2 cycles with Ar) before THF (43 mL) was added. The resulting mixture was stirred at 80 °C for 1 h. After reaching ambient temperature, a solution of alkene **14** (2.12 g, 6.30 mmol) in THF (20 mL) was added and stirring was continued at 60 °C overnight. After cooling to ambient temperature, H_2O (50 mL) and sodium perborate tetrahydrate (3.88 g, 25.2 mmol) were introduced and the resulting mixture was stirred for another 2 h before sat. aq. $Na_2S_2O_3$ (15 mL) and sat. aq. NH_4Cl (75 mL) were added. The mixture was extracted with *tert*-butyl methyl ether (3 × 75 mL), the combined organic layers were dried over $MgSO_4$ and concentrated under reduced pressure. The residue was purified by flash chromatography (silica, hexanes/EtOAc 1:1 → 2:3) to provide the title compound as a colorless oil (1.77 g, 76%, *dr* >20:1). $[\alpha]_D^{20}$ = +28.3 (c = 0.50, $CHCl_3$). ^{1}H NMR (400 MHz, $CDCl_3$) δ 7.85 – 7.76 (m, 4H), 7.54 – 7.41 (m, 3H), 5.15 (dp, *J* = 9.5, 1.4 Hz, 1H), 4.77 (d, *J* = 12.4 Hz, 1H), 4.53 (d, *J* = 12.4 Hz, 1H), 4.22 – 4.14 (m, 1H), 4.11 (dt, *J* = 7.9, 6.5 Hz, 1H), 4.03 (dd, *J* = 9.5, 6.4 Hz, 1H), 3.98 – 3.91 (m, 1H), 3.65 (dd, *J* = 11.2, 3.9 Hz, 1H), 3.53 (dd, *J* = 11.2, 5.1 Hz, 1H), 2.93 (s, 2H), 2.10 – 1.98 (m, 1H), 1.98 – 1.83 (m, 1H), 1.80 (d, *J* = 1.5 Hz, 3H), 1.71 – 1.57 (m, 6H), 1.55 – 1.47 (m, 1H). ^{13}C NMR (101 MHz, $CDCl_3$) δ 138.4, 136.5, 133.4, 133.0, 128.1, 128.0, 127.8, 126.5, 126.1, 125.8, 122.2, 81.7, 78.7, 77.2, 71.6, 69.8, 66.6, 38.8, 32.8, 27.8, 26.2, 18.9. IR (film) ṽ 3419, 3055, 2914, 2869, 1509, 1443, 1376, 1061, 953, 856, 817, 752 cm^{-1}. HRMS (ESI$^+$) calcd. for $C_{23}H_{30}O_4Na$ [M+Na]$^+$: 393.20363; found: 393.20365.

The absolute configuration was determined by Mosher ester analysis:

Preparation of the (*S*)- and (*R*)-MTPA Esters of Diol 15. (*R*)-(–)-MTPA-Cl (11 µL, 58.7 µmol) was added to a solution of diol **15** (7.2 mg, 19.4 µmol), NEt_3 (20 µL, 140 µmol) and DMAP (0.6 mg, 4.9 µmol) in CH_2Cl_2 (0.2 mL) and stirring was continued overnight. Sat. aq. NH_4Cl (5 mL) was added and the mixture was extracted with CH_2Cl_2 (3 × 5 mL). The combined organic layers were dried over $MgSO_4$ and the solvent was removed under reduced pressure. The residue was purified by flash chromatography (silica, hexanes/*tert*-butyl methyl ether 17:3) to provide the corresponding (*S*)-MTPA ester as a colorless oil (8.6 mg, 55%). ^{1}H NMR (400 MHz, $CDCl_3$) δ 7.85 – 7.72 (m, 4H), 7.56 – 7.39 (m, 7H), 7.39 – 7.26 (m, 6H), 5.45 (qd, *J* = 7.3, 6.9, 2.2 Hz, 1H), 5.12 (dp, *J* = 9.6, 1.4 Hz, 1H), 4.81 – 4.69 (m, 2H), 4.53 (d, *J* = 12.4 Hz, 1H), 4.46 (dd, *J* = 12.6, 6.7 Hz, 1H), 4.04 (dt, *J* = 7.8, 6.4 Hz, 1H), 3.95 (dd, *J* = 9.5, 6.3 Hz, 1H), 3.76 (tt, *J* = 8.2, 5.0 Hz, 1H), 3.44 (d, *J* = 1.2 Hz, 3H), 3.36 (d, *J* = 1.2 Hz, 3H), 1.89 – 1.80 (m, 4H), 1.78 (d, *J* = 1.4 Hz, 3H), 1.61 (app. d, *J* = 1.4 Hz, 3H), 1.46 – 1.31 (m, 1H).

The (*R*)-MTPA ester was prepared analogously using (*S*)-(+)-MTPA-Cl as the reagent. ^{1}H NMR (400 MHz, $CDCl_3$) δ 7.84 – 7.74 (m, 4H), 7.51 – 7.40 (m, 7H), 7.38 – 7.27 (m, 6H), 5.51 (dtd, *J* = 7.9, 5.3, 2.4 Hz, 1H),

5.15 (dp, J = 9.6, 1.4 Hz, 1H), 4.77 (d, J = 12.6 Hz, 1H), 4.65 (dd, J = 12.5, 2.4 Hz, 1H), 4.54 (d, J = 12.4 Hz, 1H), 4.40 (dd, J = 12.6, 4.8 Hz, 1H), 4.08 (dt, J = 7.6, 6.4 Hz, 1H), 3.99 (dd, J = 9.6, 6.1 Hz, 1H), 3.90 – 3.79 (m, 1H), 3.41 (dd, J = 4.0, 1.2 Hz, 6H), 2.00 – 1.82 (m, 4H), 1.79 (d, J = 1.4 Hz, 3H), 1.66 – 1.60 (m, 4H), 1.46 – 1.37 (m, 1H).

Table S6. Determination of absolute configuration of the newly set chiral center in compound **15** via Mosher ester analysis. The recorded NMR data ($CDCl_3$) suggest that the chiral center C2 is (*S*)-configured.[9] Arbitrary numbering scheme as shown in the insert.

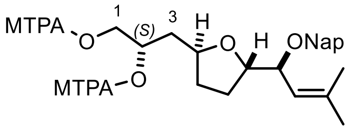

Atom number	(*S*)-ester δ [ppm]	(*R*)-ester δ [ppm]	$\Delta\delta^{S-R}$ [ppm]
1	4.77	4.65	+0.12
	4.47	4.40	+0.07
3	1.88	1.94	-0.06
	1.80	1.84	-0.04
4	3.76	3.84	-0.08
5	1.83	1.88	-0.05
	1.38	1.41	-0.03
6	1.84	1.88	-0.04
	1.56	1.63	-0.07
7	4.04	4.08	-0.04
8	3.95	3.99	-0.04

Alkenylsilane 16. NaH (278 mg, 11.6 mmol) was added in one portion to a solution of diol **15** (857 mg, 2.31 mmol) in THF (46 mL) at 0 °C. After warming to ambient temperature, the mixture was stirred for 30 min, before it was re-cooled to 0 °C and sulfonyl imidazole **24** (851 mg, 2.54 mmol) was added. Sirring was continued for 2 h at ambient temperature before sat. aq. NH_4Cl (50 mL) was introduced and the mixture was extracted with *tert*-butyl methyl ether (3 × 50 mL). The combined extracts were washed with water (50 mL) and brine (50 mL) and were dried over $MgSO_4$. The solvent was removed under reduced pressure to provide the crude epoxide, which was used in the next step without further purification.

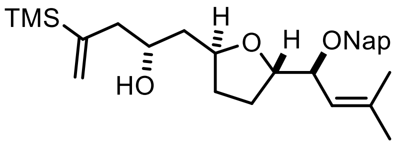

The crude epoxide was dissolved in THF (20 mL) and the mixture was cooled to 0 °C. CuCN (20.8 mg, 0.23 mmol) and (1-(trimethylsilyl)vinyl)magnesium bromide (0.5 M in THF, 9.3 mL, 4.65 mmol) were successively added and stirring was continued for 2 h. For work up, sat. aq. NH_4Cl (50 mL) was introduced, the mixture was extracted with *tert*-butyl methyl ether (3 × 50 mL), the combined extracts were washed with brine and dried over $MgSO_4$. The solvent was removed under reduced pressure and

the residue was purified by flash chromatography (silica, hexanes/*tert*-butyl methyl ether 85:15) to provide the title compound as a colorless oil (774 mg, 74%). $[\alpha]_D^{20}$ = +31.9 (c = 0.60, $CHCl_3$). ^1H NMR (400 MHz, $CDCl_3$) δ 7.85 – 7.75 (m, 4H), 7.51 – 7.42 (m, 3H), 5.65 (dt, *J* = 2.9, 1.4 Hz, 1H), 5.45 (dt, *J* = 3.0, 0.8 Hz, 1H), 5.17 (dp, *J* = 9.5, 1.4 Hz, 1H), 4.77 (d, *J* = 12.5 Hz, 1H), 4.54 (d, *J* = 12.5 Hz, 1H), 4.16 – 4.08 (m, 2H), 4.04 (dd, *J* = 9.5, 6.0 Hz, 1H), 3.97 (dtd, *J* = 9.0, 6.7, 2.2 Hz, 1H), 2.46 (ddt, *J* = 13.7, 6.7, 1.3 Hz, 1H), 2.26 (ddd, *J* = 13.9, 6.9, 2.0 Hz, 1H), 2.04 (dddd, *J* = 11.7, 8.1, 5.9, 3.2 Hz, 1H), 1.88 (dddd, *J* = 12.4, 8.1, 6.6, 3.2 Hz, 1H), 1.79 (d, *J* = 1.4 Hz, 3H), 1.73 – 1.63 (m, 2H), 1.62 (d, *J* = 1.4 Hz, 3H), 1.56 – 1.42 (m, 2H), 0.11 (s, 9H). ^{13}C NMR (101 MHz, $CDCl_3$) δ 149.4, 138.1, 136.7, 133.4, 133.0, 128.1, 128.0, 127.8, 127.2, 126.4, 126.1, 126.0, 125.8, 122.5, 81.7, 80.0, 77.1, 70.5, 69.9, 44.8, 41.8, 32.8, 27.7, 26.2, 18.9, -1.1. IR (film) ṽ 3493, 3051, 2954, 2910, 1443, 1247, 1076, 928, 837, 755 cm^{-1}. HRMS (ESI+) calcd. for $C_{28}H_{40}O_3SiNa$ $[M+Na]^+$: 475.26389; found: 475.26381.

Compound S8. TBSOTf (0.14 mL, 0.61 mmol) was added to a solution of 2,6-lutidine (0.16 mL, 1.37 mmol) and alkenylsilane **16** (211 mg, 0.47 mmol) in CH_2Cl_2 (5 mL) at 0 °C. After stirring for 15 min, the mixture was warmed to ambient temperature and stirring was continued overnight. Sat. aq. NH_4Cl (15 mL) was introduced, the layers were separated, and the aqueous phase was extracted with *tert*-butyl methyl ether (3 × 15 mL). The combined extracts were washed with brine, dried over $MgSO_4$ and concentrated. The residue was purified by flash chromatography (silica, hexanes/*tert*-butyl methyl ether 24:1) to provide the title compound as a colorless oil (261 mg, 99%). $[\alpha]_D^{20}$ = +12.6 (c = 0.30, $CHCl_3$). ^1H NMR (400 MHz, $CDCl_3$) δ 7.85 – 7.76 (m, 4H), 7.54 – 7.39 (m, 3H), 5.63 (dd, *J* = 3.0, 1.4 Hz, 1H), 5.42 (d, *J* = 3.2 Hz, 1H), 5.21 – 5.14 (m, 1H), 4.78 (d, *J* = 12.6 Hz, 1H), 4.60 (d, *J* = 12.6 Hz, 1H), 4.14 (tt, *J* = 8.1, 5.6 Hz, 1H), 4.08 – 4.02 (m, 2H), 3.86 (tdd, *J* = 7.5, 5.6, 4.3 Hz, 1H), 2.45 – 2.36 (m, 1H), 2.32 (dd, *J* = 13.5, 7.9 Hz, 1H), 1.99 (dddd, *J* = 11.8, 8.2, 5.7, 3.4 Hz, 1H), 1.92 – 1.79 (m, 2H), 1.77 (d, *J* = 1.4 Hz, 3H), 1.69 – 1.55 (m, 5H), 1.41 (ddt, *J* = 11.8, 9.4, 8.1 Hz, 1H), 0.86 (s, 9H), 0.09 (s, 9H), 0.05 (s, 3H), 0.03 (s, 3H). ^{13}C NMR (101 MHz, $CDCl_3$) δ 148.5, 137.8, 136.9, 133.5, 133.0, 128.0, 128.0, 128.0, 127.8, 126.3, 126.1, 126.0, 125.7, 122.8, 81.0, 77.7, 76.7, 69.9, 69.6, 45.0, 42.5, 32.0, 28.2, 26.2, 26.1, 18.8, 18.2, –1.1, –3.8, –4.3. IR (film) ṽ 3053, 2954, 2929, 2856, 1461, 1376, 1248, 1067, 835, 774, 758 cm^{-1}. HRMS (ESI+) calcd. for $C_{34}H_{54}O_3Si_2Na$ $[M+Na]^+$: 589.35037; found: 589.35111.

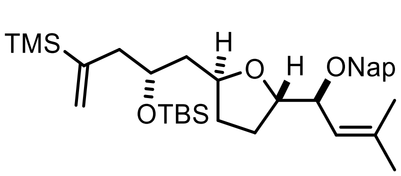

Iodide 17. Under exclusion of light, *N*-iodosuccinimide (310 mg, 1.38 mmol) and HFIP (1.2 mL) were added to a solution of 2,6-lutidine (270 µL, 2.32 mmol) and alkenylsilane **S8** (260 mg, 460 µmol) in CH_2Cl_2 (3.6 mL) at 0 °C. Stirring was continued for 2 h in the dark, before sat. aq. $Na_2S_2O_3$ (7 mL) and sat. aq. NH_4Cl (15 mL) were introduced. The layers were separated and the aqueous phase was extracted with CH_2Cl_2 (3 × 20 mL). The combined extracts were dried over $MgSO_4$, the solvent was

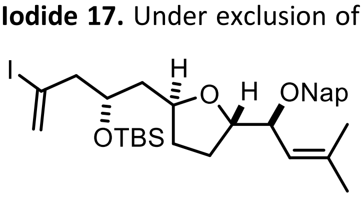

removed under reduced pressure, and the residue was purified by flash chromatography (silica, hexanes/*tert*-butyl methyl ether 24:1) to provide the title compound as a colorless oil (228 mg, 80%). $[\alpha]_D^{20}$ = +13.2 (c = 0.20, $CHCl_3$). ^{1}H NMR (400 MHz, $CDCl_3$) δ 7.81 (ddd, *J* = 8.4, 4.7, 1.8 Hz, 4H), 7.54 – 7.39 (m, 3H), 6.11 (d, *J* = 1.3 Hz, 1H), 5.74 (d, *J* = 1.3 Hz, 1H), 5.22 – 5.14 (m, 1H), 4.79 (d, *J* = 12.4 Hz, 1H), 4.61 (d, *J* = 12.5 Hz, 1H), 4.14 (dq, *J* = 8.2, 6.4 Hz, 1H), 4.12 – 3.98 (m, 3H), 2.68 – 2.52 (m, 2H), 2.07 – 1.95 (m, 1H), 1.92 – 1.81 (m, 2H), 1.78 (m, 3H), 1.68 – 1.59 (m, 5H), 1.53 – 1.45 (m, 1H), 0.87 (s, 9H), 0.10 (s, 3H), 0.07 (s, 3H). ^{13}C NMR (101 MHz, $CDCl_3$) δ 137.9, 136.9, 133.5, 133.0, 128.4, 128.0, 127.8, 126.3, 126.1, 126.0, 125.7, 122.8, 108.5, 81.3, 77.7, 76.0, 70.0, 69.2, 52.9, 42.2, 32.4, 28.3, 26.3, 26.0, 18.9, 18.2, –4.0, –4.3. IR (film) ṽ 3054, 2928, 2855, 1618, 1462, 1375, 1254, 1086, 835, 811, 775 cm^{-1}. HRMS (ESI$^+$) calcd. for $C_{31}H_{45}O_3ISiNa$ $[M+Na]^+$: 643.20749; found: 643.20756.

Alcohol S9. Glacial acetic acid (200 µL, 3.50 mmol) and TBAF trihydrate (1.11 g, 3.51 mmol) were added to a solution of compound **17** (218 mg, 350 µmol) in THF (3.6 mL). The mixture was stirred for 6 d before the reaction was quenched with sat. aq. NH_4Cl (25 mL). The mixture was extracted with *tert*-butyl methyl ether (3 × 25 mL), the combined organic extracts were washed with brine, dried over $MgSO_4$ and concentrated. The residue was purified by flash chromatography (silica, hexanes/*tert*-butyl methyl ether 3:1) to give the title compound as a colorless oil (148 mg, 83%). $[\alpha]_D^{20}$ = +36.9 (c = 0.30, $CHCl_3$). ^{1}H NMR (400 MHz, $CDCl_3$) δ 7.93 – 7.74 (m, 4H), 7.55 – 7.39 (m, 3H), 6.17 (q, *J* = 1.3 Hz, 1H), 5.82 (d, *J* = 1.4 Hz, 1H), 5.17 (dp, *J* = 9.5, 1.4 Hz, 1H), 4.78 (d, *J* = 13.0 Hz, 1H), 4.54 (d, *J* = 12.6 Hz, 1H), 4.23 – 4.05 (m, 3H), 4.03 (dd, *J* = 9.5, 6.1 Hz, 1H), 2.65 (ddd, *J* = 14.3, 7.1, 1.2 Hz, 1H), 2.44 (ddd, *J* = 14.3, 5.7, 1.2 Hz, 1H), 2.06 (dddd, *J* = 11.7, 8.1, 5.9, 3.1 Hz, 1H), 1.94 – 1.82 (m, 1H), 1.79 (d, *J* = 1.4 Hz, 3H), 1.75 – 1.61 (m, 2H), 1.62 (d, *J* = 1.4 Hz, 3H), 1.60 – 1.43 (m, 2H). ^{13}C NMR (101 MHz, $CDCl_3$) δ 138.2, 136.6, 133.4, 133.0, 128.2, 128.1, 128.1, 127.8, 126.4, 126.1, 126.1, 125.8, 122.4, 107.7, 81.8, 79.9, 76.9, 70.5, 69.8, 52.7, 41.1, 32.8, 27.6, 26.2, 18.9. IR (film) ṽ 3468, 2930, 2909, 2867, 1617, 1443, 1376, 1122, 1075, 894, 856, 815, 752 cm^{-1}. HRMS (ESI$^+$) calcd. for $C_{25}H_{31}O_3INa$ $[M+Na]^+$: 529.12101; found: 529.12114.

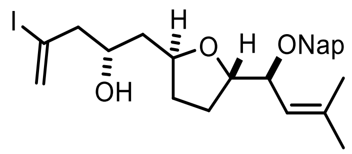

Eastern Fragment 18. Sodium phosphate buffer pH 7 (0.2 M, 1 mL) and 2,3-dichloro-5,6-dicyano-1,4-benzoquinone (98.6 mg, 430 µmol) were added to a solution of alcohol **S9** (110 mg, 220 µmol) in CH_2Cl_2 (4 mL). The mixture was stirred for 1 h before sat. aq. $Na_2S_2O_3$ (20 mL) was added. The mixture was extracted with CH_2Cl_2 (3 x 20 mL), the combined organic phases were dried over $MgSO_4$ and the solvent was removed under reduced pressure. The residue was purified by flash chromatography (silica, hexanes/*tert*-butyl methyl ether 1:2) to provide the title compound as a yellow oil (77.9 mg, 98%). $[\alpha]_D^{20}$ = +17.4 (c = 0.35, $CHCl_3$). ^{1}H NMR (400 MHz, $CDCl_3$) δ 6.16 (q, *J* = 1.3 Hz, 1H), 5.82 (d, *J* = 1.4 Hz,

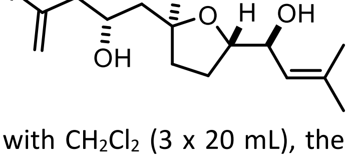

1H), 5.13 (dp, J = 9.0, 1.4 Hz, 1H), 4.26 (dddd, J = 9.4, 7.4, 5.9, 3.3 Hz, 1H), 4.17 (dd, J = 8.9, 6.8 Hz, 1H), 4.11 (ddq, J = 9.8, 5.3, 2.4 Hz, 1H), 3.95 (q, J = 6.8 Hz, 1H), 2.60 (ddd, J = 14.3, 7.5, 1.2 Hz, 1H), 2.45 (ddd, J = 14.3, 5.2, 1.3 Hz, 1H), 2.21 – 2.06 (m, 1H), 1.99 – 1.87 (m, 1H), 1.74 (d, J = 1.4 Hz, 3H), 1.72 (d, J = 1.4 Hz, 3H), 1.70 – 1.54 (m, 4H). ^{13}C NMR (101 MHz, $CDCl_3$) δ 138.1, 128.3, 123.4, 107.4, 83.1, 79.9, 70.8, 70.2, 52.7, 41.2, 32.9, 27.4, 26.1, 18.8. IR (film) ṽ 3435, 2925, 2855, 1721, 1617, 1445, 1376, 1075, 1028, 896 cm^{-1}. HRMS (ESI$^+$) calcd. for $C_{14}H_{23}O_3INa$ [M+Na]$^+$: 389.05841; found: 389.05846.

THE CENTRAL FRAGMENT OF NOMINAL PROROCENTIN

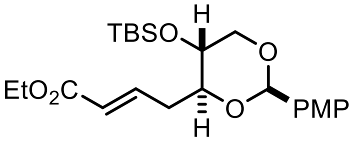

25

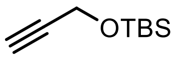

S10

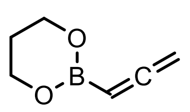

38

The compounds **25**,[10] **S10**,[11] and **38**[12] were prepared according to literature procedures.

2-((2*R*,4*S*,5*R*)-5-((*tert*-Butyldimethylsilyl)oxy)-2-(4-methoxyphenyl)-1,3-dioxan-4-yl)acetaldehyde (S11).

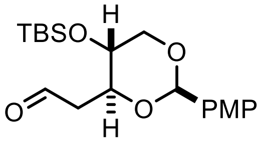

Ozone was bubbled through a solution of compound **25** (2.46 g, 5.63 mmol) in CH_2Cl_2 (28 mL) at –78 °C until a slight blue color persisted. The solution was purged with Ar for 15 min before PPh_3 (2.22 g, 8.45 mmol) was added. The mixture was allowed to warm to ambient temperature and stirring was continued for 3 h. The solvent was removed under reduced pressure and the residue was purified by flash chromatography (silica, hexanes/EtOAc 20:1 → 6:1) to provide the title compound as a colorless oil (1.89 g, 92%). $[\alpha]_{20}^{D}$ = –41.7 (c = 1.30, CH_2Cl_2). ^{1}H NMR (400 MHz, $CDCl_3$): δ 9.84 (dd, J = 2.6, 1.8 Hz, 1H), 7.39-7.36 (m, 2H), 6.89-6.86 (m, 2H), 5.50 (s, 1H), 4.20 (dd, J = 8.7, 3.1 Hz, 1H), 4.13 (td, J = 8.4, 3.8 Hz, 1H), 3.79 (s, 3H), 3.67-3.58 (m, 2H), 2.82 (ddd, J = 16.4, 3.7, 1.8 Hz, 1H), 2.66 (ddd, J = 16.4, 8.5, 2.7 Hz, 1H), 0.89 (s, 9H), 0.10 (s, 6H). ^{13}C NMR (101 MHz, $CDCl_3$) δ 200.5, 160.1, 129.9, 127.4, 113.7, 101.0, 77.7, 71.8, 66.4, 55.3, 45.9, 25.7, 17.9, –4.1, –4.7. IR (film) ṽ 2955, 2930, 2856, 1728, 1615, 1518, 1248, 1097, 1010, 856, 831, 775 cm^{-1}. HRMS (ESI$^+$) calcd. for $C_{19}H_{30}O_5SiNa$ [M+Na]$^+$: 389.17547; found: 389.17550.

(*S*)-5-((2*R*,4*S*,5*R*)-5-Hydroxy-2-(4-methoxyphenyl)-1,3-dioxan-4-yl)pent-2-yne-1,4-diol (26). $Zn(OTf)_2$

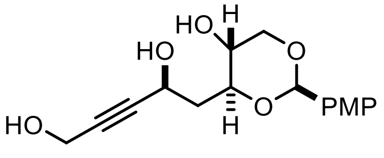

(5.45 g, 15.0 mmol) was dried under high vacuum at 125 °C for 12 h. After cooling to ambient temperature, (–)-*N*-methylephedrine (2.95 g, 16.5 mmol) was added and the mixture was dried in high vacuum for 30 min. Toluene (10 mL) and Et_3N (2.30 mL, 16.5 mmol) were added and the mixture was

stirred for 30 min before a solution of propargylic silyl ether **S10** (2.79 g, 16.4 mmol) in toluene (15 mL) was added dropwise over a period of 20 min. The mixture was stirred for additional 60 min, before a solution of aldehyde **S11** (5.00 g, 13.6 mmol) in toluene (20 mL) was added dropwise over the course of 4 h. Stirring was continued overnight before the reaction was quenched with sat. aq. NH_4Cl (50 mL). The layers were separated and the aqueous phase was extracted with EtOAc (2 × 50 mL). The combined organic layers were washed with brine and dried over Na_2SO_4. The solvent was removed under reduced pressure and the residue was filtered through a short pad of silica, which was rinsed with pentane/*tert*-butyl methyl ether (5:1 → 2:1, 200 mL). The combined filtrates were evaporated under reduced pressure to give the crude propargylic alcohol (6.11 g), which was used in the next step without further purification.

TBAF·$3H_2O$ (10.8 g, 34.2 mmol) was added in portions over the course of 10 min to a solution of the crude propargylic alcohol (6.11 g) in THF (50 mL) at 0 °C. the cooling bath was removed and stirring was continued overnight. The reaction was quenched upon addition of H_2O (90 mL) and brine (60 mL). The mixture was extracted with EtOAc (3 × 100 mL), the combined organic layers were washed with brine and dried over Na_2SO_4. The solvent was removed under reduced pressure and the residue was purified by flash chromatography (silica, hexanes/EtOAc/EtOH 10:4:1 → 4:4:1) to provide the title compound as an off-white solid material (2.71 g, dr > 20:1, 65% over two steps). $[\alpha]_{20}^{D}$ = –50.7 (c = 1.00, EtOH). ^{1}H NMR (400 MHz, [D$_4$]-MeOH): δ 7.39-7.35 (m, 2H), 6.91-6.87 (m, 2H), 5.46 (s, 1H), 4.61 (ddt, *J* = 10.1, 3.2, 1.7, 1H), 4.20 (d, *J* = 1.8 Hz, 2H), 4.17 (dd, *J* = 10.7, 5.2 Hz, 1H), 3.81-3.76 (m, 1H), 3.78 (s, 3H), 3.56 (t, *J* = 10.4 Hz, 1H), 3.43 (ddd, *J* = 10.1, 9.2, 5.1 Hz, 1H), 2.31 (ddd, *J* = 14.4, 10.2, 2.6 Hz, 1H), 1.82 (ddd, *J* = 14.4, 9.8, 3.2 Hz, 1H). ^{13}C NMR (101 MHz, [D$_4$]-MeOH): δ 161.5, 131.9, 128.6 (2C), 114.3 (2C), 102.1, 87.3, 83.4, 79.3, 72.4, 66.7, 58.4, 55.7, 50.8, 41.9. IR (film) ṽ 3460, 3327, 3156, 2973, 2865, 1518, 1387, 1241, 1058, 1031, 996, 812, 782 cm^{-1}. HRMS (ESI$^+$) calcd. for $C_{16}H_{20}O_6Na$ [M+Na]$^+$: 331.11521; found: 331.11528.

(*S,Z*)-5-((2*R*,4*S*,5*R*)-5-Hydroxy-2-(4-methoxyphenyl)-1,3-dioxan-4-yl)pent-2-ene-1,4-diol (27).

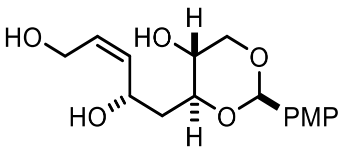

Lindlar catalyst (5% *w/w*, 370 mg, 174 µmol) and quinoline (300 µL, 2.54 mmol) were added to a solution of propargylic alcohol **26** (2.61 g, 8.48 mmol) in EtOAc (84 mL). The mixture was cooled to 0 °C, the flask was closed with a rubber septum and a hydrogen-filled balloon was connected. Under vigorous stirring, the gas atmosphere was exchanged via an outlet cannula. After 5 min, the outlet cannula was removed (keeping the balloon attached) and the mixture was stirred under hydrogen atmosphere for 2 h. The suspension was filtered through a short pad of Celite, which was rinsed with EtOH (20 mL). The combined filtrates were evaporated under reduced pressure and the residue was purified by flash chromatography (silica, hexanes/EtOAc/EtOH 55:36:9 → 5:4:1) to provide the title compound as a

colorless oil (2.38 g, 91%). $[\alpha]_{20}^{D}$ = –40.0 (c = 1.00, EtOH). ^{1}H NMR (400 MHz, [D$_4$]-MeOH): δ 7.41-7.37 (m, 2H), 6.91-6.87 (m, 2H), 5.61-5.52 (m, 2H), 5.47 (s, 1H), 4.71 (ddd, *J* = 10.1, 7.1, 3.2 Hz, 1H), 4.17 (m, 3H), 3.81-3.76 (m, 1H), 3.77 (s, 3H), 3.57 (t, *J* = 10.5 Hz, 1H), 3.46-3.40 (m, 1H), 2.14 (ddd, *J* = 14.3, 10.0, 2.8 Hz, 1H), 1.58 (ddd, *J* = 14.4, 9.4, 3.2 Hz, 1H). ^{13}C NMR (101 MHz, [D$_4$]-MeOH): δ 161.4, 135.5, 131.9, 130.6, 128.5 (2C), 114.4 (2C), 102.1, 79.8, 72.3, 66.8, 64.2, 58.7, 55.7, 41.4. IR (film) ṽ 3172, 2977, 2934, 2859, 1515, 1301, 1242, 1069, 1039, 1014, 980, 821 cm^{-1}. HRMS (ESI^{+}) calcd. for $C_{16}H_{22}O_6Na$ [M+Na]$^{+}$ 333.13086; found: 333.13068.

(2*R*,4a*R*,6*S*,7*S*,8a*S*)-2-(4-Methoxyphenyl)-6-vinylhexahydropyrano[3,2-*d*][1,3]dioxin-7-ol (28).

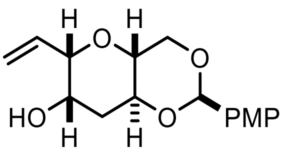

Under the exclusion of light, molecular sieves 4Å (320 mg) and (JohnPhos)Au(MeCN)SbF$_6$ (**36**) (118 mg, 153 µmol) were added to a solution of allylic alcohol **27** (950 mg, 3.06 mmol) and 2,6-di-*tert*-butylpyridine (103 µL, 459 µmol) in CH_2Cl_2/THF (1:1, 25 mL). The mixture was stirred for 3 d before it was filtered through a short pad of Celite, which was rinsed with CH_2Cl_2 (100 mL). The combined filtrates were evaporated under reduced pressure and the residue was purified by flash chromatography (silica, hexanes/EtOAc 7:3 → 3:2) to provide the title compound as a white solid material (774 mg, dr > 20:1, 86%). Mp = 119-120°C. $[\alpha]_{20}^{D}$ = –6.7 (c = 1.00, EtOH). ^{1}H NMR (400 MHz, [D$_4$]-MeOH): δ 7.39-7.36 (m, 2H), 6.90-6.87 (m, 2H), 5.87 (ddd, *J* = 17.4, 10.8, 5.7 Hz, 1H), 5.56 (s, 1H), 5.32 (dt, *J* = 17.4, 1.7 Hz, 1H), 5.20 (dt, *J* = 10.7, 1.6 Hz, 1H), 4.20 (dd, *J* = 10.4, 5.0 Hz, 1H), 4.06 (dq, *J* = 5.7, 1.5 Hz, 1H), 4.00-3.93 (m, 2H), 3.78 (s, 3H), 3.77 (t, *J* = 10.2 Hz, 1H), 3.42 (ddd, *J* = 10.1, 9.3, 4.9 Hz, 1H), 2.20 (ddd, *J* = 12.8, 4.3, 3.3 Hz, 1H), 1.86 (td, *J* = 12.5, 3.0, 1H). ^{13}C NMR (101 MHz, [D$_4$]-MeOH): δ 161.5, 136.8, 131.7, 128.7 (2C), 117.1, 114.4 (2C), 103.2, 82.4, 75.3, 75.0, 70.1, 70.0, 55.7, 37.4. IR (film) ṽ 3442, 2938, 2878, 2551, 1515, 1245, 1147, 1094, 1072, 1028, 1002, 959, 826 cm^{-1}. HRMS (ESI^{+}) calcd. for $C_{16}H_{21}O_5$ [M+H]$^{+}$ 293.13835; found: 293.13826.

For the structure of this compound in the solid state, see Figure S1.

(2*R*,4a*R*,6*S*,7S,8a*S*)-2-(4-Methoxyphenyl)-6-vinylhexahydropyrano[3,2-*d*][1,3]dioxin-7-yl meth-acrylate (29).

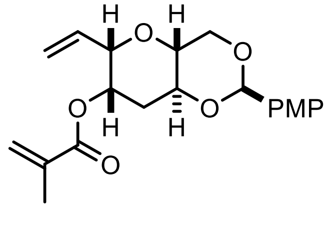

NEt$_3$ (1.50 mL, 10.8 mmol) and methacryloyl chloride (650 µL, 6.72 mmol) were added to a solution of alcohol **28** (765 mg, 2.62 mmol) in CH_2Cl_2 (27 mL), followed by DMAP (65 mg, 532 µmol). After stirring overnight, sat. aq. $NaHCO_3$ (50 mL) was introduced and the mixture was extracted with CH_2Cl_2 (3 × 50 mL). The combined organic layers were washed with brine and dried over Na_2SO_4. The solvent was removed under reduced pressure and the residue was purified by flash chromatography (silica, hexanes/*tert*-butyl methyl ether 4:1) to provide a colorless oil (809 mg, 86%).

Over the course of several hours, the material tends to polymerize, resulting in the formation of a transparent gel. After isolation, it should therefore be used immediately in the subsequent step.

$[\alpha]_{20}^{D}$ = –18.3 (c = 0.30, acetone). ^{1}H NMR (400 MHz, [D$_6$]-acetone): δ 7.39 (d, *J* = 8.5 Hz, 2H), 6.90 (d, *J* = 8.8 Hz, 2H), 6.12 (dd, *J* = 1.8, 1.0 Hz, 1H), 5.79 (ddd, *J* = 17.4, 10.8, 5.2 Hz, 1H), 5.65 (quint, *J* = 1.6 Hz, 1H), 5.62 (s, 1H), 5.31 (dt, *J* = 17.4, 1.7 Hz, 1H), 5.26-5.24 (m, 1H), 5.13 (dt, *J* = 10.8, 1.7 Hz, 1H), 4.35 (dq, *J* = 5.2, 1.6 Hz, 1H), 4.21 (dd, *J* = 10.3, 4.9 Hz, 1H), 3.91 (ddd, *J* = 11.9, 9.1, 4.5 Hz, 1H), 3.79 (s, 3H), 3.77 (t, *J* = 10.2 Hz, 1H), 3.51 (ddd, *J* = 10.1, 9.2, 4.9 Hz, 1H), 2.25 (ddd, *J* = 13.4, 4.4, 3.1 Hz, 1H), 2.02 (ddd, *J* = 13.4, 11.9, 3.1 Hz, 1H), 1.94 (dd, *J* = 1.5, 1.0 Hz, 3H). ^{13}C NMR (101 MHz, [D$_6$]-acetone): δ 166.7, 160.9, 137.4, 135.7, 131.6, 128.4 (2C), 126.0, 116.8, 114.1 (2C), 102.4, 79.8, 75.0, 74.3, 71.8, 69.6, 55.5, 34.3, 18.4. IR (film) ṽ 2959, 2929, 2871, 1715, 1517, 1292, 1248, 1156, 1090, 1031, 989, 933, 828 cm^{-1}. HRMS (ESI$^+$) calcd. for $C_{20}H_{25}O_6$ [M+H]$^+$ 361.16457; found: 361.16501.

(2*R*,4a*R*,5a*S*,9a*S*,10a*S*)-2-(4-Methoxyphenyl)-7-methyl-4,4a,5a,9a,10,10a-hexahydro-8*H*-pyrano-

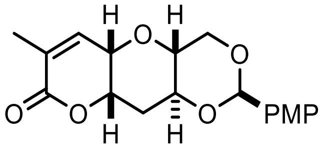

[2',3':5,6]pyrano[3,2-*d*][1,3]dioxin-8-one (S12). Hoveyda Grubbs II catalyst (**37**, 342 mg, 545 µmol) was added to a solution of diene **29** (1.32 g, 3.67 µmol) in toluene (200 mL) at 110 °C and the resulting mixture was stirred at this temperature for 75 min. After cooling to ambient temperature, the solvent was removed under reduced pressure and the residue was purified by flash chromatography (silica, pentane/*tert*-butyl methyl ether 1:1 → 0:1) to provide the title compound as a white, amorphous solid material (815 mg, 67%).

When carried out on smaller scale (73 mg) under otherwise identical conditions, the yield was 77%.

$[\alpha]_{20}^{D}$ = –20.2 (c = 0.65, acetone). ^{1}H NMR (400 MHz, CD_2Cl_2) δ 7.42 – 7.33 (m, 2H), 6.92 – 6.83 (m, 2H), 6.61 (dq, *J* = 6.3, 1.6 Hz, 1H), 5.52 (s, 1H), 4.60 (td, *J* = 3.2, 2.1 Hz, 1H), 4.20 (dd, *J* = 10.5, 5.0 Hz, 1H), 4.04 (dd, *J* = 6.3, 1.9 Hz, 1H), 3.93 (ddd, *J* = 11.8, 9.1, 4.4 Hz, 1H), 3.79 (s, 3H), 3.68 (t, *J* = 10.3 Hz, 1H), 3.44 (ddd, *J* = 10.1, 9.1, 5.0 Hz, 1H), 2.50 (ddd, *J* = 13.8, 4.5, 2.8 Hz, 1H), 2.02 – 1.90 (m, 4H). ^{13}C NMR (101 MHz, CD_2Cl_2) δ 164.9, 160.5, 135.2, 133.3, 130.5, 127.8, 113.8, 102.1, 75.4, 73.6, 72.8, 69.1, 68.3, 55.6, 33.6, 17.3. IR (film) ṽ 2969, 2924, 2867, 1719, 1249, 1091, 1031, 986, 962, 833, 818, 564, 537 cm^{-1}. HRMS (ESI$^+$) calcd. for $C_{18}H_{20}O_6Na$ [M+Na]$^+$: 355.11521; found: 355.11493.

(2*R*,4a*R*,5a*S*,7*R*,9a*S*,10a*S*)-2-(4-Methoxyphenyl)-7-methyloctahydro-8*H*-pyrano[2',3':5,6]pyrano-

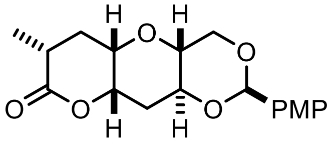

[3,2-*d*][1,3]dioxin-8-one (30). Pd/C (10% *w/w*, 347 mg, 358 µmol) was added in one portion to a solution of olefin **S12** (1.05 g, 3.16 mmol) in EtOAc (165 mL). The flask was closed with a rubber septum and a hydrogen-filled balloon was connected. Under vigorous stirring, the gas atmosphere was exchanged via an outlet cannula. After 5 min, the outlet cannula was removed (keeping the balloon attached) and

the mixture was stirred under hydrogen atmosphere for 7 h. The suspension was filtered through a short pad of Celite and the filter cake was carefully rinsed with EtOAc (30 mL). The combined filtrates were evaporated to give a grey, amorphous solid material, which was used without further purification (1.01 g, dr > 20:1, 95%). $[\alpha]_{20}^{D}$ = –238.0 (c = 0.10, acetone). ^{1}H NMR (400 MHz, CD_2Cl_2) δ 7.41 – 7.33 (m, 2H), 6.91 – 6.83 (m, 2H), 5.52 (s, 1H), 4.49 (td, *J* = 3.2, 1.5 Hz, 1H), 4.18 (dd, *J* = 10.4, 5.0 Hz, 1H), 4.02 (ddt, *J* = 7.0, 2.8, 1.6 Hz, 1H), 3.91 (ddd, *J* = 11.7, 9.2, 4.6 Hz, 1H), 3.79 (s, 3H), 3.69 (t, *J* = 10.3 Hz, 1H), 3.41 (ddd, *J* = 10.1, 9.2, 5.0 Hz, 1H), 2.58 – 2.41 (m, 3H), 1.92 (ddd, *J* = 13.6, 11.7, 3.3 Hz, 1H), 1.52 – 1.37 (m, 1H), 1.23 – 1.13 (m, 3H). ^{1}H NMR (400 MHz, [D$_6$]-acetone) δ 7.39 (d, *J* = 8.7 Hz, 2H), 6.90 (d, *J* = 8.8 Hz, 2H), 5.62 (s, 1H), 4.69 (dd, *J* = 4.5, 3.1 Hz, 1H), 4.15 (ddd, *J* = 8.9, 2.8, 1.6 Hz, 1H), 4.12 (dd, *J* = 10.3, 5.0 Hz, 1H), 3.85 (ddd, *J* = 11.7, 9.1, 4.5 Hz, 1H), 3.79 (s, 3H), 3.68 (t, *J* = 10.3 Hz, 1H), 3.41 (ddd, *J* = 10.1, 9.3, 5.0 Hz, 1H), 2.73-2.57 (m, 2H), 2.32 (ddd, *J* = 13.5, 4.5, 3.1 Hz, 1H), 1.96 (ddd, *J* = 13.6, 11.8, 3.4 Hz, 1H), 1.33 (ddd, *J* = 13.9, 11.4, 2.1 Hz, 1H), 1.12 (d, *J* = 11.4 Hz, 3H). ^{13}C NMR (101 MHz, CD_2Cl_2) δ 175.0, 160.5, 130.6, 127.8, 113.8, 75.0, 73.7, 73.5, 72.7, 69.2, 55.6, 34.0, 33.2, 32.2, 15.7. ^{13}C NMR (101 MHz, [D$_6$]-acetone) δ 175.2, 160.9, 131.6, 128.4 (2C), 114.1 (2C), 102.4, 75.1, 74.3, 73.8, 73.2, 69.4, 55.5, 34.6, 33.5, 32.2, 15.8. IR (film) ṽ 2923, 2856, 1747, 1251, 1170, 1106, 1088, 1025, 983, 818, 557, 532 cm^{-1}. HRMS (ESI$^+$) calcd. for $C_{18}H_{22}O_6Na$ [M+Na]$^+$: 357.13086; found: 357.13078.

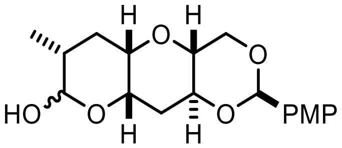

(2*R*,4a*R*,5a*S*,7*R*,9a*S*,10a*S*)-2-(4-Methoxyphenyl)-7-methyloctahydro-4H-pyrano[2',3':5,6]pyrano-[3,2-*d*][1,3]dioxin-8-ol (S13). A solution of Dibal-H (1 M in CH_2Cl_2, 3.30 mL, 3.30 mmol) was added dropwise to a solution of lactone **30** (1.01 g, 3.01 mmol) in CH_2Cl_2 (100 mL) at –78 °C. Stirring was continued for 15 min before MeOH (200 µL) and Rochelle´s salt solution (100 mL) were added successively. After warming to ambient temperature, the mixture was vigorously stirred overnight. The layers were separated and the aqueous phase was extracted with CH_2Cl_2 (3 × 100 mL). The combined organic layers were washed with brine and dried over Na_2SO_4. Removal of the solvent provided the title compound as an mixture of diastereoisomers (1.04 g, 96%). Spectral data of the major isomer: ^{1}H NMR (400 MHz, CD_2Cl_2) δ 7.41 – 7.32 (m, 2H), 6.87 (d, *J* = 8.9 Hz, 2H), 5.51 (s, 1H), 5.01 – 4.97 (m, 1H), 4.27 – 4.24 (m, 1H), 4.23 – 4.16 (m, 1H), 3.90 – 3.82 (m, 1H), 3.79 (s, 3H), 3.66 (t, *J* = 10.3 Hz, 1H), 3.60 (dt, *J* = 4.1, 2.0 Hz, 1H), 3.28 (dddd, *J* = 10.1, 9.0, 7.7, 5.0 Hz, 1H), 2.57 (d, *J* = 3.1 Hz, 1H), 2.18 – 2.08 (m, 1H), 1.96 – 1.68 (m, 2H), 1.56 – 1.52 (m, 1H), 1.14 (d, *J* = 7.4 Hz, 3H). ^{13}C NMR (101 MHz, CD_2Cl_2) δ 160.4, 131.0, 127.8, 113.8, 102.0, 97.4, 74.7, 73.6, 72.8, 69.8, 65.2, 55.6, 34.5, 30.6, 29.3, 19.0. IR (film) ṽ 2927, 1518, 1386, 1249, 1095, 1020, 1010, 970, 829, 736 cm^{-1}. HRMS (ESI$^+$) calcd. for $C_{18}H_{24}O_6Na$ [M+Na]$^+$: 359.14651; found: 359.14633.

Ethyl (*R*,*E*)-5-((2*R*,4a*R*,6*S*,7*S*,8a*S*)-7-hydroxy-2-(4-methoxyphenyl)hexahydropyrano[3,2-*d*][1,3]-dioxin-6-yl)-4-methylpent-2-enoate (31).

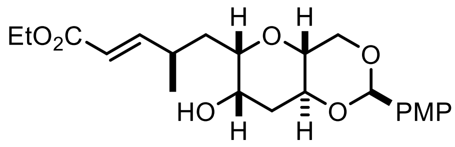

Ethyl 2-(triphenyl-λ^5-phosphaneylidene)acetate (2.00 g, 5.75 mmol) was added in one portion to a solution of lactol **S13** (967 mg, 2.88 mmol) in toluene/THF (1:1, 4 mL). The mixture was stirred at 80 °C overnight. A second batch of ethyl 2-(triphenyl-λ^5-phosphaneylidene)acetate (500 mg, 2.30 mmol) was added and stirring was continued overnight. After cooling to ambient temperature, the solvent was removed under reduced pressure and the residue was purified by flash chromatography (silica, pentane/*tert*-butyl methyl ether 1:2, then CH_2Cl_2/*tert*-butyl methyl ether 6:1) to provide the title compound as a yellow oil (674 mg, 58%; *66%* brsm).

When carried out on smaller scale (55 mg) under otherwise identical conditions, the reaction provided the desired product in 74% yield.

$[\alpha]_{20}^{D}$ = –77.6 (c = 1.00, CH_2Cl_2). ^{1}H NMR (400 MHz, CD_2Cl_2) δ 7.40 – 7.31 (m, 2H), 6.91 – 6.83 (m, 2H), 6.83 (s, 1H), 5.81 (dd, *J* = 15.7, 1.0 Hz, 1H), 5.50 (s, 1H), 4.21 (dd, *J* = 10.4, 4.9 Hz, 1H), 4.16 (q, *J* = 7.1 Hz, 2H), 3.90 – 3.80 (m, 2H), 3.80 (s, 3H), 3.70 (t, *J* = 10.3 Hz, 1H), 3.46 (ddd, *J* = 10.0, 3.3, 1.4 Hz, 1H), 3.33 (ddd, *J* = 10.2, 9.2, 4.9 Hz, 1H), 2.66 – 2.51 (m, 1H), 2.22 (ddd, *J* = 12.9, 4.5, 3.2 Hz, 1H), 1.85 – 1.73 (m, 3H), 1.43 (ddd, *J* = 14.2, 9.7, 3.3 Hz, 1H), 1.28 (t, *J* = 7.1 Hz, 3H), 1.09 (d, *J* = 6.8 Hz, 3H). ^{13}C NMR (101 MHz, CD_2Cl_2) δ 166.9, 160.5, 153.6, 130.8, 127.8, 121.0, 113.8, 102.2, 78.3, 74.7, 74.4, 69.6, 69.5, 60.6, 55.6, 38.0, 37.2, 33.6, 20.5, 14.5. IR (film) ṽ 1714, 1518, 1369, 1303, 1274, 1249, 1181, 1160, 1093, 1032, 999, 974, 830 cm^{-1}. HRMS (ESI$^+$) calcd. for $C_{22}H_{30}O_7Na$ [M+Na]$^+$: 429.18837; found: 429.18850.

(2*R*,4a*R*,6*S*,7*S*,8a*S*)-6-((*R*,*E*)-5-Hydroxy-2-methylpent-3-en-1-yl)-2-(4-methoxyphenyl)hexahydro-pyrano[3,2-*d*][1,3]dioxin-7-ol (S14).

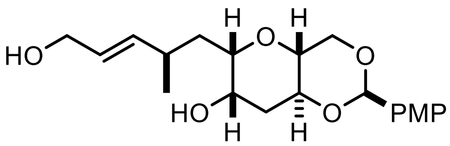

A solution of Dibal-H (1 M in CH_2Cl_2, 6.60 mL, 6.60 mmol) was added dropwise to a solution of ethyl acrylate **31** (670 mg, 1.65 mmol) in THF (100 mL) at –78 °C. Stirring was continued overnight at this temperature before MeOH (2 mL) and Rochelle´s salt solution (200 mL) were added. After warming to ambient temperature, the mixture was vigorously stirred overnight. The layers were separated and the aqueous phase was extracted with CH_2Cl_2 (3 × 100 mL). The combined organic layers were washed with brine and dried over Na_2SO_4. Removal of the solvent provided the title compound as a colorless oil (599 mg, quant.). $[\alpha]_{20}^{D}$ = –46.5 (c = 0.31, CH_2Cl_2). ^{1}H NMR (400 MHz, CD_2Cl_2) δ 7.44 – 7.24 (m, 2H), 7.03 – 6.79 (m, 2H), 5.64 (dtd, *J* = 15.3, 5.6, 0.7 Hz, 1H), 5.56 – 5.45 (m, 2H), 4.21 (dd, *J* = 10.4, 4.9 Hz, 1H), 4.07 (d, *J* = 5.5 Hz, 2H), 3.85 (ddd, *J* = 11.8, 9.1, 4.5 Hz, 2H), 3.79 (s, 3H), 3.70 (t, *J* = 10.3 Hz, 1H), 3.51 (ddd, *J* = 9.6, 3.7, 1.4 Hz, 1H), 3.33 (ddd, *J* = 10.1, 9.1, 4.9 Hz, 1H), 2.43 – 2.31 (m, 1H), 2.23 (ddd, *J* = 12.9, 4.5, 3.1 Hz, 1H), 1.84 – 1.75

(m, 2H), 1.68 (ddd, J = 14.2, 9.6, 4.7 Hz, 1H), 1.41 – 1.29 (m, 2H), 1.03 (d, J = 6.8 Hz, 3H). ^{13}C NMR (101 MHz, CD_2Cl_2) δ 160.5, 137.6, 130.9, 129.1, 127.8, 113.8, 102.2, 78.7, 74.8, 74.4, 69.7, 69.6, 63.8, 55.6, 38.6, 37.2, 33.5, 21.5. IR (film) ṽ 2927, 2866, 1615, 1518, 1390, 1250, 1173, 1093, 1033, 1003, 972, 830 cm^{-1}. HRMS (ESI$^+$) calcd. for $C_{20}H_{28}O_6Na$ $[M+Na]^+$: 387.17781; found: 387.17809.

***tert*-Butyl(((*R*,*E*)-5-((2*R*,4a*R*,6*S*,7*S*,8a*S*)-7-((*tert*-butyldimethylsilyl)oxy)-2-(4-methoxyphenyl)hexahydropyrano[3,2-*d*][1,3]dioxin-6-yl)-4-methyl-pent-2-en-1-yl)oxy)dimethylsilane (32).**

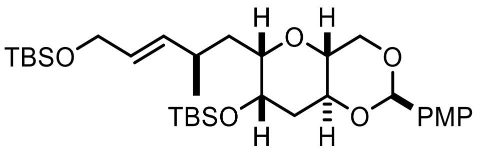

TBSOTf (930 µL, 4.05 mmol) was added dropwise to a solution of diol **S14** (329 mg, 903 µmol) and 2,6-lutidine (1.00 mL, 8.59 mmol) in CH_2Cl_2 (18 mL) at –78 °C. Stirring was continued for 2 h before sat. aq. NH_4Cl (50 mL) was added. The mixture was warmed to ambient temperature and the layers were separated. The aqueous phase was extracted with CH_2Cl_2 (3 × 50 mL). The combined organic phases were washed with brine and dried over Na_2SO_4. The solvent was removed under reduced pressure to provide the title compound as a colorless oil (482 mg, 90%). $[\alpha]_{20}^{D}$ = –55.6 (c = 1.20, CH_2Cl_2). ^{1}H NMR (400 MHz, CD_2Cl_2) δ 7.40 – 7.33 (m, 2H), 6.96 – 6.81 (m, 2H), 5.61 – 5.41 (m, 3H), 4.18 (dd, J = 10.3, 4.9 Hz, 1H), 4.13 (dd, J = 4.9, 1.2 Hz, 2H), 3.90 – 3.82 (m, 2H), 3.79 (s, 3H), 3.70 (t, J = 10.3 Hz, 1H), 3.44 (ddd, J = 9.7, 3.2, 1.3 Hz, 1H), 3.29 (ddd, J = 10.3, 9.0, 4.9 Hz, 1H), 2.42 – 2.28 (m, 1H), 2.13 (ddd, J = 12.7, 4.3, 3.2 Hz, 1H), 1.80 – 1.62 (m, 2H), 1.24 – 1.13 (m, 1H), 1.00 (d, J = 6.8 Hz, 3H), 0.94 (s, 9H), 0.91 (s, 9H), 0.10 (m, 6H), 0.07 (s, 6H). ^{13}C NMR (101 MHz, CD_2Cl_2) δ 160.4, 136.2, 131.1, 129.1, 127.9, 113.8, 102.1, 78.9, 74.9, 74.3, 70.6, 69.7, 64.2, 55.6, 39.5, 37.9, 33.2, 26.1, 26.1, 21.5, 18.7, 18.4, –4.5, –4.7, –5.0. IR (film) ṽ 2953, 2929, 2856, 1518, 1463, 1388, 1249, 1095, 1037, 969, 832, 774 cm^{-1}. HRMS (ESI$^{+/-}$) calcd. for $C_{32}H_{57}O_6Si_2$ $[M+H]^+$: 593.36882; found: 593.36936.

((2*R*,3*S*,5*S*,6*S*)-5-((*tert*-Butyldimethylsilyl)oxy)-6-((*R*,*E*)-5-((*tert*-butyldimethylsilyl)oxy)-2-methyl-pent-3-en-1-yl)-3-((4-methoxybenzyl)oxy)tetrahydro-2*H*-pyran-2-yl)methanol (33).

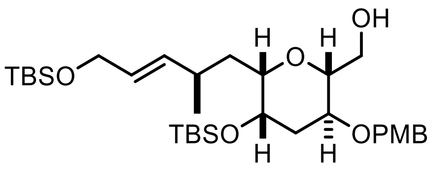

A solution of Dibal-H (1 M in CH_2Cl_2, 4.50 mL, 4.50 mmol) was added dropwise to a solution of acetal **32** (597 mg, 1.00 mmol) in CH_2Cl_2 (30 mL) at –78 °C. Stirring was continued for 2 d before MeOH (2 mL) and Rochelle´s salt solution (100 mL) were added. After warming to ambient temperature, the mixture was vigorously stirred overnight. The layers were separated and the aqueous phase was extracted with CH_2Cl_2 (3 × 50 mL). The combined organic layers were washed with brine and dried over Na_2SO_4. The solvent was removed under reduced pressure and the residue was purified by flash chromatography (silica, pentane/*tert*-butyl methyl ether 4:1) to provide the title compound as a colorless oil (560 mg, 93%). $[\alpha]_{20}^{D}$ = –3.5 (c = 1.00, CH_2Cl_2). ^{1}H NMR (400 MHz, CD_2Cl_2) δ 7.26 – 7.18 (m, 2H), 6.89 – 6.81 (m, 2H), 5.56 – 5.39 (m, 2H), 4.48 (d, J = 11.3 Hz, 1H), 4.35 (d, J = 11.3 Hz, 1H), 4.13 – 4.08 (m, 2H), 3.83 – 3.72 (m, 5H), 3.61 (ddt, J = 15.0, 11.3, 5.2 Hz,

2H), 3.35 (ddd, *J* = 9.6, 3.2, 1.2 Hz, 1H), 3.22 (ddd, *J* = 9.3, 5.8, 3.4 Hz, 1H), 2.40 – 2.22 (m, 2H), 2.07 (t, *J* = 6.4 Hz, 1H), 1.64 (ddd, *J* = 14.2, 9.6, 4.9 Hz, 1H), 1.54 – 1.46 (m, 1H), 1.19 (ddd, *J* = 14.0, 9.6, 3.2 Hz, 1H), 0.99 (d, *J* = 6.8 Hz, 3H), 0.89 (s, 18H), 0.07 – 0.04 (m, 12H). ^{13}C NMR (101 MHz, CD_2Cl_2) δ 159.7, 136.2, 131.0, 129.8, 128.9, 114.1, 80.9, 77.9, 70.9, 70.3, 64.1, 63.7, 55.6, 39.4, 38.0, 33.3, 26.1, 26.1, 21.4, 18.7, 18.4, –4.4, –4.7, –5.0. IR (film) ṽ 2953, 2929, 2856, 1513, 1462, 1251, 1075, 1039, 962, 834, 774 cm^{-1}. HRMS (ESI$^+$) calcd. for $C_{32}H_{58}O_6Si_2Na$ [M+Na]$^+$: 617.36642; found: 617.36716.

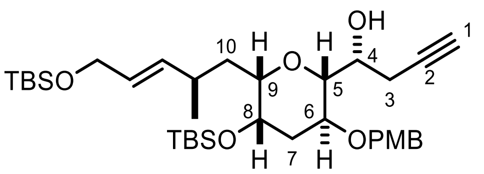

Compound 34. DMSO (300 µL, 4.22 mmol) was added to a solution of oxalyl chloride (150 µL, 1.75 mmol) in CH_2Cl_2 (12.5 mL) at −78 °C. Stirring was continued for 15 min, before a solution of alcohol **33** (500 mg, 840 µmol) in CH_2Cl_2 (4 mL) was added. The mixture was stirred for 15 min before NEt_3 (0.6 mL, 4.30 mmol) was introduced. After additional 15 min, the mixture was warmed to 0 °C and stirring was continued for another 15 min. Sat. aq. NH_4Cl (30 mL) was added and the layers were separated. The aqueous phase was extracted with *tert*-butyl methyl ether (3 × 100 mL), the combined organic layers were washed with brine and dried over Na_2SO_4. The solvent was removed under reduced pressure to provide the crude aldehyde, which was directly used in the next step without further purification.

A solution of the crude aldehyde in toluene (12 mL) was added to a solution of (*R*)-(+)-3,3′-dibromo-1,1′-bi-2-naphthol (**39**, 111 mg, 250 µmol) and allenyl boronate **38** (165 mg, 1.33 mmol) in toluene (2.5 mL). The mixture was stirred overnight, the solvent was removed under reduced pressure, and the residue was purified by flash chromatography (fine silica, hexanes/*tert*-butyl methyl ether 8:1) to provide the title compound as a colorless oil (360 mg, 68% over two steps). A second fraction contained the epimeric alcohol (19%), which was discarded. $[\alpha]_{20}^{D}$ = 13.5 (c = 1.00, CH_2Cl_2). ^{1}H NMR (400 MHz, $CDCl_3$) δ 7.25 – 7.18 (m, 2H), 6.89 – 6.83 (m, 2H), 5.61 – 5.52 (m, 1H), 5.42 (ddt, *J* = 15.3, 8.3, 1.5 Hz, 1H), 4.56 (d, *J* = 11.0 Hz, 1H), 4.35 (d, *J* = 11.0 Hz, 1H), 4.12 (dd, *J* = 5.0, 1.5 Hz, 2H), 3.91 (ddd, *J* = 7.1, 6.3, 3.9 Hz, 1H), 3.86 – 3.76 (m, 5H), 3.71 (d, *J* = 3.3 Hz, 1H), 3.32 (ddd, *J* = 10.2, 2.6, 1.2 Hz, 1H), 3.26 – 3.18 (m, 1H), 2.59 – 2.45 (m, 2H), 2.33 (dt, *J* = 12.8, 3.9 Hz, 2H), 1.98 (t, *J* = 2.6 Hz, 1H), 1.66 (ddd, *J* = 14.2, 10.1, 4.2 Hz, 1H), 1.54 (ddd, *J* = 13.4, 11.2, 2.6 Hz, 1H), 1.12 – 1.04 (m, 1H), 0.99 (d, *J* = 6.8 Hz, 3H), 0.90 (s, 18H), 0.09 – 0.04 (m, 12H). ^{13}C NMR (101 MHz, $CDCl_3$) δ 159.6, 135.7, 129.8, 129.5, 128.9, 114.2, 81.8, 79.6, 78.0, 74.2, 72.4, 70.0, 69.9, 69.6, 55.4, 39.4, 37.4, 33.1, 29.9, 26.1, 26.0, 23.0, 21.6, 18.6, 18.3, –4.4, –4.6, –4.9, –4.9. IR (film) ṽ 2953, 2928, 2856, 1514, 1463, 1376, 1252, 1087, 1039, 835, 775 cm^{-1}. HRMS (ESI$^+$) calcd. for $C_{35}H_{60}O_6Si_2Na$ [M+Na]$^+$: 655.38207; found: 655.38237.

The absolute configuration of the major product was determined by Mosher ester analysis:

Preparation of the (*S*)- and (*R*)-MTPA Esters of Alcohol 34. (*R*)-(–)-MTPA-Cl (6.1 µL, 33 µmol) was added to a solution of alcohol **34** (3.2 mg, 5.1 µmol), NEt_3 (7.0 µL, 50.6 µmol) and DMAP (0.3 mg, 2.5 µmol) in CH_2Cl_2 (0.3 mL). The mixture was stirred overnight, before sat. aq. $NaHCO_3$ (5 mL) was added. The mixture was extracted with *tert*-butyl methyl ether (3 × 10 mL) and the combined organic layers were washed with brine and dried over Na_2SO_4. The residue was purified by flash chromatography (silica, hexanes/*tert*-butyl methyl ether 10:1) to provide (*S*)-MTPA ester as a colorless oil (1.8 mg, 42%); for the spectral data, see Table S7.

The (*R*)-MTPA ester was prepared analogously using (*S*)-(+)-MTPA-Cl as the reagent; for the spectral data, see Table S7.

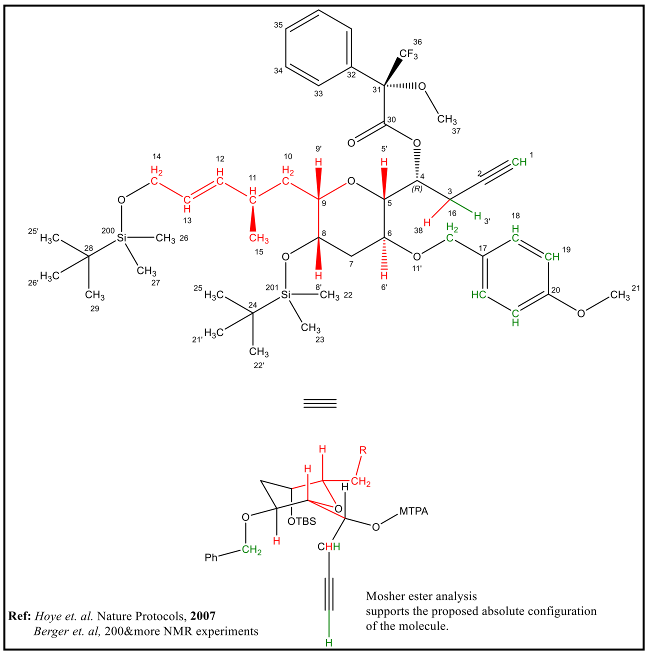

Table S7. Determination of absolute configuration of the newly set chiral center in homopropargylic alcohol **34** via Mosher ester analysis.[9] The recorded NMR data ($CDCl_3$) suggest that the alcohol at C4 is *(R)*-configured. Arbitrary numbering scheme as shown in the insert.

Atom number	*(S)*-ester δ [ppm]	*(R)*-ester δ [ppm]	$\Delta\delta^{SR}$
1 C	70.24	70.30	
H	1.96	1.89	0.07
2 C	81.04	80.42	
3 C	18.79	19.56	
H'	2.7	2.65	0.05
H''	2.38	2.45	-0.07
4 C	74.73	75.22	
H	5.64	5.54	0.1
5 C	80.04	79.71	
5' H	3.38	3.49	-0.11
6 C	68.98	69.66	
6' H	3.67	3.75	-0.08
7 C	37.54	37.87	
H'	2.24	2.22	0.02
H''	1.46	1.49	-0.03
8 C	69.45	69.59	
8' H	3.64	3.67	-0.03

9 C	77.98	78.11	
9' H	3.19	3.3	-0.11
10 C	39.2	39.21	
H'	1.51	1.6	-0.09
H''	1.09	1.14	-0.05
11 C	32.9	32.87	
H	2.19	2.29	-0.1
12 C	136.18	136	
H	5.37	5.42	-0.05
13 C	128.07	128.4	
H	5.37	5.46	-0.09
14 C	63.99	63.98	
H2	4.03	4.09	-0.06
15 C	21.33	21.49	
H3	0.9	0.97	-0.07
16 C	70.11	70.38	
H'	4.52	4.42	0.1
H''	4.37	4.27	0.1
17 C	130.15	130.21	
18 C	129.72	129.69	
H	7.27	7.17	0.1
19 C	114.14	114.05	
H	6.89	6.85	0.04
20 C	159.49	159.43	
21 C	55.44	55.43	
H3	3.81	3.8	0.01
22 C	-4.31	-4.71	
H3	0.03	0.01	0.02
23 C	-4.81	-4.38	
H3	0	0.01	-0.01
24 C	18.15	18.19	
25 C	25.91	25.93	
H3	0.86	0.84	0.02
26 C	-4.91	-4.92	
H3	0.04	0.05	-0.01
27 C	-4.93	-4.9	
H3	0.04	0.05	-0.01
28 C	18.58	18.59	
29 C	26.12	26.13	
H3	0.89	0.9	-0.01
30 C	166.09	166.05	
31 C	84.82	84.75	
32 C	132.44	132.72	
33 C	127.86	127.73	
H	7.62	7.61	0.01

34 C	129.61	128.38	
H	7.38	7.37	0.01
35 C	128.37	129.53	
H	7.37	7.37	0
36 C	123.42	123.53	
37 C	55.68	55.84	
H3	3.58	3.6	-0.02
200 Si	19.75	17.94	
201 Si	17.66	19.81	
202 F	-71.48	-71.94	

Acetate S15. Pyridine (120 µL, 1.45 mmol), Ac_2O (100 µL, 1.09 mmol), and DMAP (8.9 mg, 72.7 µmol) were added to a solution of the homopropargylic alcohol **34** (460 mg, 727 µmol) in CH_2Cl_2 (7.0 mL). Stirring was continued for 36 h before sat. aq. $NaHCO_3$ (5 mL) was added. After separation of the layers, the aqueous phase was extracted with CH_2Cl_2 (3 × 10 mL). The combined organic layers were washed with brine and dried over Na_2SO_4. The solvent was removed under reduced pressure and the residue was purified by flash chromatography (silica, hexanes/*tert*-butyl methyl ether 10:1) to provide the title compound as a colorless oil (485 mg, 99%). $[\alpha]_{20}^{D}$ = –15.3 (c = 1.00, CH_2Cl_2). ^{1}H NMR (400 MHz, $CDCl_3$) δ 7.29 – 7.23 (m, 2H), 6.91 – 6.84 (m, 2H), 5.52 (dt, *J* = 15.7, 5.3 Hz, 1H), 5.45 – 5.33 (m, 2H), 4.50 (d, *J* = 11.3 Hz, 1H), 4.34 (d, *J* = 11.3 Hz, 1H), 4.13 – 4.06 (m, 2H), 3.79 (s, 3H), 3.73 – 3.62 (m, 2H), 3.36 (dd, *J* = 9.5, 3.2 Hz, 1H), 3.26 (ddd, *J* = 9.9, 2.9, 1.1 Hz, 1H), 2.59 (ddd, *J* = 17.0, 8.1, 2.7 Hz, 1H), 2.44 – 2.32 (m, 2H), 2.25 (ddd, *J* = 12.9, 4.4, 3.5 Hz, 1H), 1.99 (s, 3H), 1.92 – 1.90 (m, 1H), 1.71 – 1.59 (m, 1H), 1.47 (ddd, *J* = 13.2, 11.0, 2.5 Hz, 1H), 1.12 – 1.03 (m, 1H), 0.99 (d, *J* = 6.7 Hz, 3H), 0.90 (s, 9H), 0.88 (s, 9H), 0.05 (s, 6H), 0.04 (s, 3H), 0.02 (s, 3H). ^{13}C NMR (101 MHz, $CDCl_3$) δ 170.4, 159.4, 136.1, 130.4, 129.7, 128.6, 114.0, 81.2, 79.9, 77.7, 71.8, 70.3, 70.1, 69.6, 69.5, 64.1, 55.4, 39.0, 37.7, 33.0, 26.1, 25.9, 21.4, 21.2, 19.3, 18.6, 18.2, –4.3, –4.8, –4.9. IR (film) ṽ 2953, 2929, 2856, 1741, 1513, 1462, 1368, 1240, 1071, 965, 831, 773, 636 cm^{-1}. HRMS (ESI$^{+/-}$) calcd. for $C_{37}H_{62}O_7Si_2Na$ $[M+Na]^+$: 697.39263; found: 697.39321.

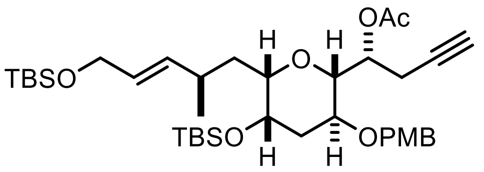

Compound 35. 2,3-Dichloro-5,6-dicyano-1,4-benzoquinone (252 mg, 1.11 mmol) and pH 7 phosphate buffer (0.05 M, 2.0 mL) were added to a solution of acetate **S15** (500 mg, 741 µmol) in CH_2Cl_2 (8.0 mL). The mixture was vigorously stirred for 2 h before sat. aq. $NaHCO_3$ (10 mL) was introduced. The resulting mixture was filtered through a short pad of Celite, which was rinsed CH_2Cl_2 (20 mL). The layers were separated and the aqueous

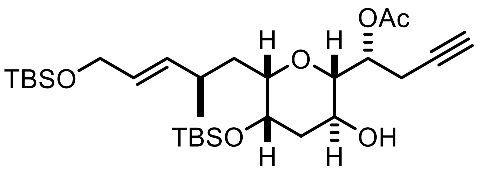

layer was extracted with CH_2Cl_2 (5 × 10 mL). The combined organic layers were washed with brine and dried over Na_2SO_4. The solvent was removed under reduced pressure and the residue was purified by flash chromatography (silica, hexanes/*tert*-butyl methyl ether 5:1) to provide the title compound as a pale yellow oil (273 mg, 0.491 mmol, 66%). $[\alpha]_{20}^{D}$ = –25.5 (c = 1.00, CH_2Cl_2). ^{1}H NMR (400 MHz, CD_2Cl_2) δ 5.54 (dtd, *J* = 15.3, 5.1, 0.8 Hz, 1H), 5.42 (ddt, *J* = 15.3, 8.4, 1.5 Hz, 1H), 5.20 (ddd, *J* = 6.7, 5.4, 4.2 Hz, 1H), 4.11 (dd, *J* = 5.1, 1.5 Hz, 2H), 3.96 – 3.84 (m, 1H), 3.73 – 3.64 (m, 1H), 3.32 – 3.21 (m, 2H), 2.76 – 2.58 (m, 2H), 2.42 – 2.29 (m, 1H), 2.12 (ddd, *J* = 12.9, 4.8, 3.3 Hz, 1H), 2.07 (s, 3H), 2.01 (t, *J* = 2.7 Hz, 1H), 1.87 (d, *J* = 5.4 Hz, 1H), 1.67 (ddd, *J* = 14.1, 10.0, 4.7 Hz, 1H), 1.52 (ddd, *J* = 12.9, 11.2, 2.7 Hz, 1H), 1.13 (ddd, *J* = 14.1, 10.0, 3.0 Hz, 1H), 1.00 (d, *J* = 6.8 Hz, 3H), 0.90 (s, 9H), 0.90 (s, 9H), 0.08 (s, 3H), 0.06 (s, 6H), 0.04 (s, 3H). ^{13}C NMR (101 MHz, CD_2Cl_2) δ 170.6, 136.2, 129.1, 81.3, 81.0, 78.0, 72.8, 70.0, 64.7, 64.2, 41.7, 39.3, 33.4, 26.1, 26.0, 21.5, 21.3, 19.8, 18.6, 18.3, –4.5, –4.8, –5.0. IR (film) ṽ 3425, 2953, 2929, 2856, 1737, 1462, 1371, 1251, 1099, 1068, 1038, 964, 832, 773, 635 cm^{-1}. HRMS (ESI$^{+/-}$) calcd. for $C_{29}H_{54}O_6Si_2Na$ $[M+Na]^+$: 577.33512; found: 577.33506.

THE REVISED CENTRAL FRAGMENT

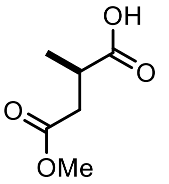

41

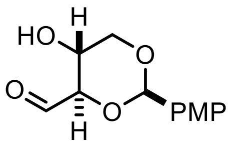

44

The compounds **41**[13] and **44**[14] were prepared according to literature procedures.

(2*R*,4*S*,5*R*)-4-(1-Hydroxybut-2-yn-1-yl)-2-(4-methoxyphenyl)-1,3-dioxan-5-ol (45). 1-Propynylmagnesium bromide (0.5 M in THF, 100 mL, 50 mmol) was added to a solution of aldehyde **44** (4.19 g, 17.6 mmol) in THF (75 mL) at 0 °C. The mixture was warmed to ambient temperature and stirring was continued overnight. Sat. aq. NH_4Cl (130 mL) and brine (70 mL) were introduced, the layers were separated and the aqueous layer was extracted with EtOAc (5 x 100 mL). The combined organic layers were washed with brine and dried over Na_2SO_4. The solvent was removed under reduced pressure and the residue was purified by flash chromatography (silica, CH_2Cl_2/diethyl ether 3:1) to provide the title compound as mixture of diastereoisomers (dr ≈ 2:1, 3.97 mg, 81%).

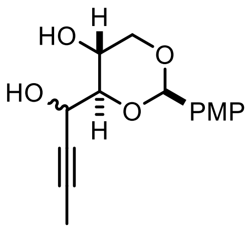

Data of the major (*S*)-isomer: $[\alpha]_{20}^{D}$ = –11.8 (c = 1.00, CH_2Cl_2). ^{1}H NMR (400 MHz, CD_2Cl_2) δ 7.43 – 7.34 (m, 2H), 6.93 – 6.84 (m, 2H), 5.48 (s, 1H), 4.63 (m, 1H), 4.25 (dd, *J* = 10.8, 5.5 Hz, 1H), 3.99 (dddd, *J* =

10.2, 9.1, 5.5, 3.4 Hz, 1H), 3.80 (s, 3H), 3.70 (dd, J = 9.1, 4.8 Hz, 1H), 3.59 (dd, J = 10.8, 10.2 Hz, 1H), 2.71 (d, J = 3.6 Hz, 1H), 2.57 (d, J = 6.2 Hz, 1H), 1.88 (d, J = 2.2 Hz, 3H). ^{13}C NMR (101 MHz, CD_2Cl_2) δ 160.6, 130.4, 127.9, 113.9, 101.3, 83.8, 83.0, 77.1, 70.8, 64.1, 63.8, 55.7, 3.8. IR (film) ṽ 3403, 1615, 1519, 1462, 1394, 1304, 1250, 1173, 1109, 1080, 1029, 989, 832 cm^{-1}. HRMS (ESI$^+$) calcd. for $C_{15}H_{18}O_5Na$ [M+Na]$^+$: 301.10464; found: 301.10469.

Data of the minor (R)-isomer: $[\alpha]_{20}^{D}$ = –13.3 (c = 1.00, CH_2Cl_2). ^{1}H NMR (400 MHz, CD_2Cl_2) δ 7.43 – 7.34 (m, 2H), 6.93 – 6.85 (m, 2H), 5.49 (s, 1H), 4.61 (ddq, J = 6.5, 4.4, 2.2 Hz, 1H), 4.25 (dd, J = 10.8, 5.5 Hz, 1H), 4.00 (dddd, J = 10.2, 9.2, 5.5, 3.8 Hz, 1H), 3.80 (s, 3H), 3.66 (dd, J = 9.2, 4.1 Hz, 1H), 3.59 (dd, J = 10.8, 10.2 Hz, 1H), 2.62 – 2.59 (m, 2H), 1.89 (d, J = 2.2 Hz, 3H). ^{13}C NMR (101 MHz, CD_2Cl_2) δ 160.6, 130.4, 127.9, 113.9, 101.4, 83.6, 83.0, 77.5, 70.8, 63.5, 62.8, 55.7, 3.8. IR (film) ṽ 3410, 1615, 1518, 1393, 1304, 1249, 1174, 1133, 1077, 1030, 977, 831 cm^{-1}. HRMS (GC-EI) calcd. for $C_{15}H_{18}O_5$ [M]$^+$: 278.11488; found: 278.11503.

1-((2*R*,4*R*,5*R*)-5-Hydroxy-2-(4-methoxyphenyl)-1,3-dioxan-4-yl)but-2-yn-1-one (S16). MnO_2 (62.5 g, 719 mmol) was added to a solution of alcohol **45** (8.00 g, 28.7 mmol) in CH_2Cl_2 (280 mL). After stirring for 2 h, the mixture was filtered through a pad of Celite, which was carefully rinsed with CH_2Cl_2 (100 mL). The combined filtrates were evaporated under reduced pressure and the residue was purified by flash chromatography (silica, CH_2Cl_2/diethyl ether 12:1 → 10:1) to provide the title compound as a yellow oil (4.82 g, 61%). $[\alpha]_{20}^{D}$ = –21.2 (c = 1.00, CH_2Cl_2). ^{1}H NMR (400 MHz, CD_2Cl_2) δ 7.48 – 7.37 (m, 2H), 6.95 – 6.87 (m, 2H), 5.53 (s, 1H), 4.29 (dd, J = 11.0, 5.3 Hz, 1H), 4.05 (d, J = 9.1 Hz, 1H), 3.96 (dddd, J = 10.0, 9.0, 5.3, 2.9 Hz, 1H), 3.81 (s, 3H), 3.62 (dd, J = 11.0, 9.9 Hz, 1H), 2.91 (d, J = 2.9 Hz, 1H), 2.10 (s, 3H). ^{13}C NMR (101 MHz, CD_2Cl_2) δ 186.7, 160.7, 130.05, 127.9, 113.9, 101.5, 97.2, 84.9, 78.7, 70.5, 63.5, 55.7, 4.7. IR (film) ṽ 2215, 1673, 1615, 1519, 1393, 1303, 1250, 1174, 1124, 1081, 1032, 980, 833 cm^{-1}. HRMS (ESI$^+$) calcd. for $C_{15}H_{16}O_5Na$ [M+Na]$^+$: 299.08899; found: 299.08941.

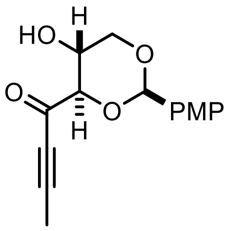

(2*R*,4*S*,5*R*)-4-((*R*)-1-Hydroxybut-2-yn-1-yl)-2-(4-methoxyphenyl)-1,3-dioxan-5-ol ((*R*)-45). A solution of ketone **S16** (4.82 g, 17.4 mmol) in CH_2Cl_2 (250 mL) was added to a mixture of RuCl[*(S,S)*-Teth-TsDpen] (**57**, 230 mg, 370 µmol), NEt_3 (10 mL) and formic acid (6.8 mL) and the resulting mixture was stirred for 2 h. Sat. aq. $NaHCO_3$ (100 mL) and brine (100 mL) were added, the layers were separated and the aqueous layer was extracted with CH_2Cl_2 (5 x 100 mL). The combined organic layers were washed with brine and dried over Na_2SO_4. The solvent was removed under reduced pressure and the residue was purified by flash chromatography (silica, CH_2Cl_2/diethyl ether 3:1) to provide the title compound as a white solid (4.40 g, dr > 20:1, 91%). For the spectral data, see above.

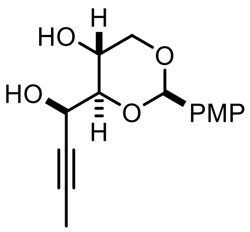

(2*R*,4*R*,5*R*)-4-((*R*)-1-((*tert*-Butyldimethylsilyl)oxy)but-2-yn-1-yl)-2-(4-methoxyphenyl)-1,3-dioxan-5-ol (46).

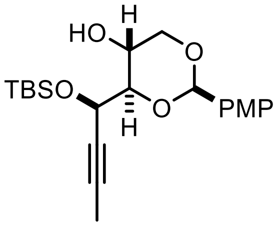

Imidazole (2.15 g, 31.6 mmol) and TBSCl (2.62 g, 17.4 mmol) were added to a solution of diol (*R*)-**45** (4.40 g, 15.8 mmol) in CH_2Cl_2 (200 mL) and the resulting mixture was stirred overnight. Sat. aq. $NaHCO_3$ (100 mL) was added, the layers were separated and the aqueous layer was extracted with CH_2Cl_2 (3 x 100 mL). The combined organic layers were washed with brine and dried over Na_2SO_4. The solvent was removed under reduced pressure and the residue was purified by flash chromatography (silica, hexanes/*tert*-butyl methyl ether 3:1) to provide the title compound as a colorless oil (5.99 g, 87%). $[\alpha]_{20}^{D}$ = +26.8 (c = 1.00, CH_2Cl_2). ^{1}H NMR (400 MHz, CD_2Cl_2) δ 7.41 – 7.33 (m, 2H), 6.91 – 6.83 (m, 2H), 5.45 (s, 1H), 4.70 (dq, *J* = 4.4, 2.2 Hz, 1H), 4.25 (dd, *J* = 10.7, 5.5 Hz, 1H), 4.15 (dddd, *J* = 10.0, 8.9, 5.5, 1.1 Hz, 1H), 3.79 (s, 3H), 3.65 (dd, *J* = 8.9, 4.6 Hz, 1H), 3.62 – 3.55 (m, 1H), 3.29 (d, *J* = 1.1 Hz, 1H), 1.90 (d, *J* = 2.2 Hz, 3H), 0.92 (s, 9H), 0.19 (s, 3H), 0.16 (s, 3H). ^{13}C NMR (101 MHz, CD_2Cl_2) δ 160.5, 130.6, 127.9, 113.8, 101.3, 84.1, 81.5, 77.1, 70.7, 66.8, 63.6, 55.6, 25.8, 18.4, 3.8, –4.6, –5.2. IR (film) ṽ 2955, 2930, 2857, 1519, 1390, 1250, 1173, 1135, 1082, 1037, 835, 782 cm^{-1}. HRMS (ESI$^+$) calcd. for $C_{21}H_{32}O_5SiNa$ [M+Na]$^+$: 415.19112; found: 415.19124.

Methyl (*R*)-4-((*tert*-butyldimethylsilyl)oxy)-3-methylbutanoate (S17). $BH_3{\cdot}Me_2S$ (6.89 mL, 72.5 mmol)

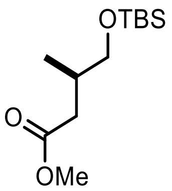

was added dropwise to a solution of acid **41** (8.47 g, 58.0 mmol) in THF (40 mL) at –30 °C. The mixture was warmed to ambient temperature over the course of 1 h and stirring was continued for 3 h. After cooling to 0 °C, MeOH (12 mL) was added and all volatile materials were removed in high vacuum. This procedure was repeated twice to provide the crude alcohol, which was used in the next step without further purification.

The crude alcohol was dissolved in CH_2Cl_2 (50 mL), imidazole (11.8 g, 174 mmol) and TBSCl (13.1 g, 87.0 mmol) were added, and the resulting mixture was stirred overnight. Sat. aq. NH_4Cl (50 mL) was introduced, the layers were separated and the aqueous layer was extracted with Et_2O (3 x 100 mL). The combined organic phases were washed with brine and dried over Na_2SO_4. The solvent was removed under reduced pressure and the residue was purified by flash chromatography (silica, hexanes/*tert*-butyl methyl ether 20:1) to provide the title compound as a colorless oil (13.1 g, 92% over two steps). $[\alpha]_{20}^{D}$ = +5.6 (c = 1.07, $CHCl_3$); lit.[15]: $[\alpha]_{20}^{D}$ = +4.3 (c = 1.05, $CHCl_3$). ^{1}H NMR (400 MHz, CD_2Cl_2) δ 3.63 (s, 3H), 3.49 (dd, *J* = 9.8, 5.2 Hz, 1H), 3.40 (dd, *J* = 9.9, 6.4 Hz, 1H), 2.52 – 2.40 (m, 1H), 2.14 – 2.03 (m, 2H), 0.91 (d, *J* = 6.5 Hz, 3H), 0.89 (s, 9H), 0.04 (s, 6H). ^{13}C NMR (101 MHz, CD_2Cl_2) δ 173.8, 67.8, 51.6, 38.2, 33.5, 26.0, 18.6, 16.7, –5.4. IR (film) ṽ 2954, 2930, 2857, 1740, 1436, 1253, 1193, 1174, 1092, 1034, 1008, 835, 814, 775 cm^{-1}. HRMS (ESI$^+$) calcd. for $C_{12}H_{26}O_3SiNa$ [M+Na]$^+$: 269.15434; found: 269.15416.

(*R*)-4-((*tert*-Butyldimethylsilyl)oxy)-3-methylbutanoic acid (42). LiOH (6.34 g, 265 mmol) was added

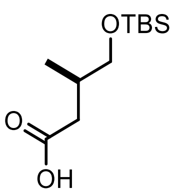

in one portion to a solution of ester **S17** (13.1 g, 53 mmol) in a mixture of THF/MeOH/H_2O (2:1:1, 200 mL) at 0 °C. After warming to ambient temperature, the mixture was stirred for 3 h. The mixture was diluted with CH_2Cl_2 (80 mL) and acidified to pH ≈ 5 by dropwise addition of HCl (1 M, 132 mL). The layers were separated and the aqueous phase was extracted with CH_2Cl_2 (3 x 20 mL). The combined organic layers were washed with brine and dried over Na_2SO_4. The solvent was removed under reduced pressure to provide the title compound as a colorless oil (12.3 g, quant.). $[\alpha]_{20}^{D}$ = +8.2 (c = 1.00, CH_2Cl_2). ^{1}H NMR (400 MHz, CD_2Cl_2) δ 10.54 (br. S, 1H), 3.57 (dd, *J* = 9.9, 5.0 Hz, 1H), 3.47 – 3.37 (m, 1H), 2.57 – 2.45 (m, 1H), 2.23 – 2.05 (m, 2H), 0.95 (d, *J* = 6.6 Hz, 3H), 0.90 (s, 9H), 0.06 (s, 6H). ^{13}C NMR (101 MHz, CD_2Cl_2) δ 178.3, 68.1, 38.7, 33.2, 26.0, 18.6, 16.7, –5.4, –5.4. IR (film) ṽ 2955, 2930, 2886, 2857, 1708, 1472, 1411, 1254, 1094, 1033, 835, 814, 776 cm^{-1}. HRMS (ESI$^-$) calcd. for $C_{11}H_{23}O_3Si$ [M–H]$^-$: 231.14220; found: 231.14232.

Compound 47. DMAP (853 mg, 6.98 mmol) and *N*-(3-dimethylaminopropyl)-*N*′-ethylcarbodiimide hydrochloride (2.01 g, 10.5 mmol) were added to a solution of alcohol **46** (1.37 g, 3.49 mmol) and acid

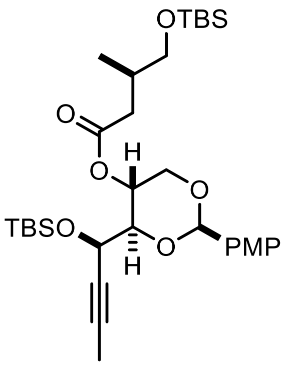

42 (1.67 g, 7.19 mmol) in CH_2Cl_2 (120 mL). After stirring for 3 d, the mixture was poured into a mixture of H_2O (50 mL) and brine (50 mL), the layers were separated and the aqueous layer was extracted with CH_2Cl_2 (3 x 100 mL). The combined organic layers were washed with brine and dried over Na_2SO_4. The solvent was removed under reduced pressure and the residue was purified by flash chromatography (silica, hexanes/*tert*-butyl methyl ether 10:1) to provide the title compound as a colorless oil (2.05 g, 97%). $[\alpha]_{20}^{D}$ = –36.0 (c = 1.00, CH_2Cl_2). ^{1}H NMR (400 MHz, CD_2Cl_2) δ 7.45 – 7.36 (m, 2H), 6.93 – 6.85 (m, 2H), 5.48 (s, 1H), 5.04 (td, *J* = 9.8, 5.4 Hz, 1H), 4.64 – 4.54 (m, 1H), 4.38 (dd, *J* = 10.5, 5.4 Hz, 1H), 3.84 – 3.77 (m, 4H), 3.55 (t, *J* = 10.3 Hz, 1H), 3.51 – 3.39 (m, 2H), 2.56 – 2.45 (m, 1H), 2.13 – 2.01 (m, 2H), 1.83 (d, *J* = 2.2 Hz, 3H), 0.93 (d, *J* = 6.4 Hz, 3H), 0.90 (d, *J* = 0.8 Hz, 18H), 0.12 (s, 3H), 0.08 (s, 3H), 0.05 (s, 6H). ^{13}C NMR (101 MHz, CD_2Cl_2) δ 172.1, 160.6, 130.6, 128.0, 113.8, 101.7, 82.4, 81.8, 77.9, 68.0, 67.7, 63.7, 63.1, 55.7, 38.2, 33.4, 26.1, 26.0, 18.6, 18.5, 16.6, 3.8, –4.3, –5.0, –5.3, –5.4. IR (film) ṽ 2955, 2929, 2857, 1743, 1250, 1170, 1091, 1033, 835, 777 cm^{-1}. HRMS (ESI$^+$) calcd. for $C_{32}H_{54}O_7Si_2Na$ [M+Na]$^+$: 629.33003; found: 629.32986.

Compound 48. Lindlar catalyst (5% *w/w*, 2.50 g, 1.17 mmol) was added to a solution of alkyne **47** (7.12 g, 11.7 mmol) and quinoline (420 µL, 3.56 mmol) in EtOAc (230 mL). The suspension was cooled to 0 °C, hydrogen was bubbled through for 5 min, and stirring was continued under hydrogen atmosphere (balloon) for 80 min. The mixture was filtered through a short pad of Celite, which was carefully rinsed with EtOAc (30 mL). The combined filtrates were evaporated under reduced pressure and the residue was purified by flash chromatography (silica, hexanes/*tert*-butyl methyl ether 10:1) to provide the title compound as a colorless oil (7.01 g, 98%).

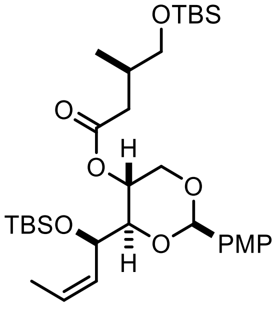

$[\alpha]_{20}^{D}$ = –27.4 (c = 1.00, CH_2Cl_2). ^1H NMR (400 MHz, CD_2Cl_2) δ 7.43 – 7.34 (m, 2H), 6.92 – 6.84 (m, 2H), 5.61 – 5.48 (m, 2H), 5.42 (s, 1H), 4.98 (td, *J* = 9.8, 5.5 Hz, 1H), 4.74 – 4.65 (m, 1H), 4.37 (dd, *J* = 10.5, 5.5 Hz, 1H), 3.80 (s, 3H), 3.70 (dd, *J* = 9.6, 3.4 Hz, 1H), 3.59 – 3.46 (m, 2H), 3.42 (dd, *J* = 9.9, 6.4 Hz, 1H), 2.57 – 2.43 (m, 1H), 2.15 – 2.02 (m, 2H), 1.68 (d, *J* = 5.1 Hz, 3H), 0.94 (d, *J* = 6.3 Hz, 3H), 0.90 (s, 9H), 0.87 (s, 9H), 0.06 (s, 6H), 0.01 (s, 3H), 0.00 (s, 3H). ^{13}C NMR (101 MHz, CD_2Cl_2) δ 172.2, 160.5, 130.9, 130.8, 127.9, 125.5, 113.8, 101.5, 82.6, 68.2, 67.6, 67.3, 63.6, 55.6, 38.2, 33.4, 26.1, 26.0, 18.6, 18.5, 16.6, 13.6, –4.1, –4.8, –5.3, –5.4. IR (film) ṽ 2955, 2929, 2856, 1743, 1518, 1250, 1171, 1148, 1095, 1031, 983, 834, 776 cm^{-1}. HRMS (ESI$^+$) calcd. for $C_{32}H_{56}O_7Si_2Na$ $[M+Na]^+$: 631.34568; found: 631.34566.

Enol Ether 50. Under exclusion of light, a solution of Tebbe´s reagent (**58**) (0.5 M in toluene, 550 mL, 275 mmol; prepared *in-situ* before use)[16] was added to a solution of ester **48** (7.00 g, 11.5 mmol) in THF (450 mL). Stirring was continued overnight, before a second batch of Tebbe´s reagent (0.5 M in toluene, 184 mL, 92.0 mmol) and additional THF (100 mL) were added. After stirring overnight, the mixture was cooled to 0 °C and the reaction was quenched by *extremely careful* addition of aq. NaOH (3 M, 377 mL, 1.13 mol). The solution turned dark blue and a blue, oily precipitate was formed. After stirring for 10 min at 0 °C and additional 10 min at ambient temperature, *tert*-butyl methyl ether (250 mL) was introduced and the organic supernatant was transferred via cannula to a column packed with a short pad of Celite topped by Na_2SO_4, which was rinsed with further *tert*-butyl methyl ether (100 mL). This extraction procedure was repeated twice. The combined organic filtrates were dried over Na_2SO_4 and the solvent was removed under reduced pressure. The residue was purified by flash chromatography (silica, hexanes/*tert*-butyl methyl ether/NEt_3 50:1:1) to provide the title compound as a yellow oil (3.22 g, 47%).

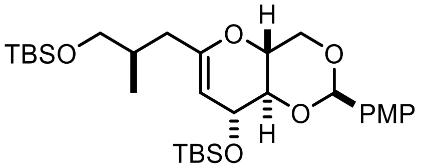

$[\alpha]_{20}^{D}$ = –31.3 (c = 1.00, CH_2Cl_2). ^1H NMR (400 MHz, CD_2Cl_2) δ 7.45 – 7.38 (m, 2H), 6.93 – 6.85 (m, 2H), 5.53 (s, 1H), 4.50 – 4.43 (m, 2H), 4.37 – 4.22 (m, 1H), 3.83 – 3.74 (m, 5H), 3.68 (dd, *J* = 9.8, 7.1 Hz, 1H), 3.46 (dd, *J* = 9.8, 5.2 Hz, 1H), 3.41 (dd, *J* = 9.8, 5.5 Hz, 1H), 2.18 – 2.06 (m, 1H), 1.89 – 1.77 (m, 2H), 0.96 – 0.82 (m, 21H), 0.08 (s, 3H), 0.06 (s, 3H), 0.05 (s, 6H). ^{13}C NMR (101 MHz, CD_2Cl_2) δ 160.4, 154.0, 130.6, 127.7, 113.7, 102.1,

101.5, 81.2, 69.0, 68.9, 68.4, 67.6, 55.6, 37.3, 26.1, 26.0, 18.6, 18.5, 16.8, 1.2, –4.3, –4.6, –5.3. IR (film) ṽ 2954, 2929, 2889, 2857, 1518, 1378, 1250, 1186, 1102, 1070, 1038, 1006, 908, 835, 776 cm^{-1}. HRMS (ESI^+) calcd. for $C_{30}H_{53}O_6Si_2$ $[M+H]^+$: 565.33752; found: 565.33759.

Compound 51. Enol ether **50** (3.20 g, 5.66 mmol) and Pt/C (10% *w/w*, 5.60 g, 2.87 mmol) were suspended in EtOH (200 mL). Hydrogen was bubbled through the suspension for 5 min at 0°C and stirring was continued under hydrogen atmosphere (balloon) at this temperature for 3 h. The mixture was filtered through a short pad of Celite, which was carefully rinsed with *tert*-butyl methyl ether (30 mL). The combined filtrates were evaporated under reduced pressure and the residue was purified by flash chromatography (silica, hexanes/*tert*-butyl methyl ether/NEt_3 50:1:1) to provide the title compound as a colorless oil (2.43 g, dr > 20:1, 74%).

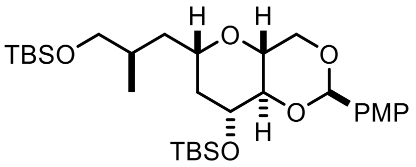

When carried out on smaller scale (226 mg) under otherwise identical conditions, the reaction provided the desired product in 83% yield.

$[\alpha]_{20}^{D}$ = –31.4 (c = 1.00, CH_2Cl_2). ^1H NMR (400 MHz, CD_2Cl_2) δ 7.47 – 7.34 (m, 2H), 6.91 – 6.83 (m, 2H), 5.48 (s, 1H), 4.19 (dd, *J* = 10.3, 4.6 Hz, 1H), 3.86 (ddd, *J* = 10.8, 8.3, 5.3 Hz, 1H), 3.79 (s, 3H), 3.70 – 3.55 (m, 2H), 3.46 (dd, *J* = 9.8, 5.5 Hz, 1H), 3.41 (dd, *J* = 9.8, 6.1 Hz, 1H), 3.37 – 3.26 (m, 2H), 1.95 (ddd, *J* = 13.3, 5.3, 2.1 Hz, 1H), 1.75 (dq, *J* = 12.8, 6.4 Hz, 1H), 1.51 – 1.30 (m, 3H), 0.92 – 0.88 (m, 12H), 0.86 (s, 9H), 0.06 (s, 3H), 0.04 (s, 6H), 0.02 (s, 3H). ^{13}C NMR (101 MHz, CD_2Cl_2) δ 160.3, 131.0, 127.8, 113.6, 101.7, 84.4, 75.2, 71.3, 70.8, 69.4, 68.3, 55.6, 41.9, 39.6, 32.9, 26.1, 25.9, 18.6, 18.5, 17.7, –4.3, –4.7, –5.3. IR (film) ṽ 2954, 2929, 2883, 2856, 1518, 1250, 1171, 1104, 1038, 859, 836, 777 cm^{-1}. HRMS (ESI^+) calcd. for $C_{30}H_{54}O_6Si_2Na$ $[M+Na]^+$: 589.33470; found: 589.33478.

Compound 52. A solution of TBAF (1 M in THF, 9.20 mL, 9.20 mmol) was added dropwise to a solution of silyl ether **51** (2.35 g, 4.15 mmol) in THF (40 mL) at 0 °C. The mixture was warmed to ambient temperature and stirring was continued for 5 h. A second portion of TBAF (1 M in THF, 1.24 mL, 1.24 mmol) was added and stirring continued for another 40 min, before sat. aq. NH_4Cl (50 mL) and *tert-b*utyl methyl ether (100 mL) were introduced. The layers were separated and the aqueous phase was extracted with *tert*-butyl methyl ether (4 × 100 mL). The combined organic layers were washed with brine and dried over Na_2SO_4. The solvent was removed under reduced pressure and the residue was purified by flash chromatography (silica, hexanes/*tert*-butyl methyl ether 1:1 → 1:2) to provide the title compound as a white solid material (1.13 g, 81%). $[\alpha]_{20}^{D}$ = –20.7 (c = 1.00, CH_2Cl_2). ^1H NMR (400 MHz, CD_2Cl_2) δ 7.43 – 7.35 (m, 2H), 6.92 – 6.84 (m, 2H), 5.50 (s, 1H), 4.26 – 4.16 (m, 1H), 3.88 (dtd, *J* = 11.0, 5.3, 2.5 Hz, 1H), 3.80 (s, 3H), 3.75 – 3.60 (m, 2H), 3.45 (t, *J* = 5.9 Hz, 2H), 3.41 – 3.32 (m, 2H), 2.41 (d, *J* = 2.7 Hz, 1H), 2.04 (ddd, *J* = 13.1, 5.2, 2.2 Hz, 1H),

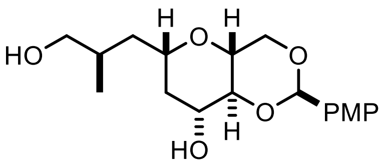

1.83 (dq, J = 12.9, 6.4 Hz, 1H), 1.74 (t, J = 6.0 Hz, 1H), 1.53 – 1.42 (m, 3H), 0.93 (d, J = 6.9 Hz, 3H). ^{13}C NMR (101 MHz, CD_2Cl_2) δ 160.6, 130.6, 127.9, 113.9, 102.1, 84.3, 74.8, 70.8, 69.8, 69.3, 67.9, 55.7, 39.6, 39.4, 32.9, 17.3. IR (film) ṽ 2927, 2869, 1615, 1518, 1380, 1250, 1174, 1099, 1032, 990, 831 cm^{-1}. HRMS (ESI$^+$) calcd. for $C_{18}H_{26}O_6Na$ [M+Na]$^+$: 361.16216; found: 361.16188.

Compound 53. Dess-Martin periodinane (1.71 g, 4.03 mmol) was added in one portion to a mixture of

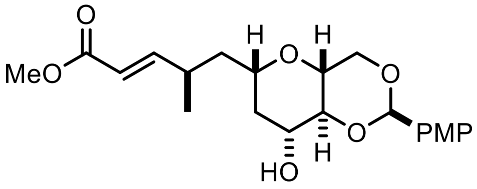

alcohol **52** (1.13 g, 3.35 mmol) and $NaHCO_3$ (1.70 g, 20.2 mmol) in CH_2Cl_2 (150 mL) at 0 °C. Stirring was continued for 50 min before a second batch of Dess-Martin periodinane (284 mg, 670 mmol) was added. After 25 min, sat. aq. $NaHCO_3$ (50 mL) and sat aq. $Na_2S_2O_3$ (50 mL) were introduced, the mixture was warmed to ambient temperature and stirring was continued for 40 min. The layers were separated and the aqueous layer was extracted with CH_2Cl_2 (3 × 100 mL). The combined organic layers were washed with brine and dried over Na_2SO_4. The solvent was removed to provide the crude aldehyde, which was directly used in the next step without further purification.

Methyl (triphenylphosphoranylidene)acetate (5.60 g, 16.8 mmol) was added to a solution of the crude aldehyde in CH_2Cl_2 (3 × 100 mL) and the resulting mixture was stirred overnight. The solvent was removed under reduced pressure and the residue was purified by flash chromatography (silica, hexanes/*tert*-butyl methyl ether 1:1 → 1:2) to provide the title compound as a colorless oil (1.39 g, 86% over two steps). $[\alpha]_{20}^{D}$ = –63.4 (c = 0.50, CH_2Cl_2). ^{1}H NMR (400 MHz, CD_2Cl_2) δ 7.41 – 7.35 (m, 2H), 6.93 – 6.85 (m, 2H), 6.80 (dd, J = 15.7, 8.6 Hz, 1H), 5.81 (dd, J = 15.6, 1.0 Hz, 1H), 5.49 (s, 1H), 4.22 (dd, J = 10.3, 4.7 Hz, 1H), 3.89 – 3.82 (m, 1H), 3.79 (s, 3H), 3.70 (s, 3H), 3.65 (t, J = 10.1 Hz, 1H), 3.51 (tdd, J = 11.6, 3.4, 2.2 Hz, 1H), 3.39 – 3.22 (m, 2H), 2.67 – 2.53 (m, 1H), 2.41 (d, J = 2.6 Hz, 1H), 1.99 (ddd, J = 13.0, 5.2, 2.2 Hz, 1H), 1.63 (ddd, J = 14.1, 9.5, 4.7 Hz, 1H), 1.50 – 1.38 (m, 2H), 1.06 (d, J = 6.9 Hz, 3H). ^{13}C NMR (101 MHz, CD_2Cl_2) δ 167.3, 160.6, 153.9, 130.6, 127.9, 120.5, 113.9, 102.1, 84.3, 74.3, 70.7, 69.7, 69.3, 55.7, 51.7, 42.2, 39.7, 33.5, 20.4. IR (film) ṽ 2870, 1720, 1616, 1518, 1250, 1174, 1097, 1032, 831 cm^{-1}. HRMS (ESI$^+$) calcd. for $C_{21}H_{28}O_7Na$ [M+Na]$^+$: 415.17272; found: 415.17222.

Compound S18. A solution of Dibal-H (1 M in CH_2Cl_2, 11.4 mL, 11.4 mmol) was added dropwise to a

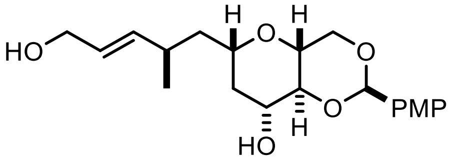

solution of compound **53** (1.11 g, 2.84 mmol) in THF (180 mL) at –78 °C. Stirring was continued overnight before a second portion of Dibal-H (1 M in CH_2Cl_2, 3 mL, 3 mmol) was introduced. After stirring for 3 d, MeOH (2.5 mL), Rochelle´s salt solution (150 mL) and EtOAc (100 mL) were added and the mixture was vigorously stirred for 1 h at ambient temperature. The layers were separated and the aqueous phase was extracted with EtOAc (3 × 100 mL). The combined organic layers were washed with brine and dried over Na_2SO_4. The solvent

was removed under reduced pressure and the residue was purified by flash chromatography (silica, hexanes/*tert*-butyl methyl ether 1:2 → 0:1) to provide the title compound as a colorless oil (857 mg, 83%). $[\alpha]_{20}^{D}$ = –15.4 (c = 1.00, CH_2Cl_2). ^{1}H NMR (400 MHz, CD_2Cl_2) δ 7.43 – 7.34 (m, 2H), 6.92 – 6.84 (m, 2H), 5.62 (dtd, *J* = 15.4, 5.5, 0.7 Hz, 1H), 5.54 – 5.46 (m, 2H), 4.23 (dd, *J* = 10.3, 4.7 Hz, 1H), 4.06 (t, *J* = 4.6 Hz, 2H), 3.86 (ddd, *J* = 15.8, 8.0, 5.1 Hz, 1H), 3.79 (s, 3H), 3.66 (t, *J* = 10.0 Hz, 1H), 3.60 – 3.49 (m, 1H), 3.40 – 3.23 (m, 2H), 2.47 – 2.35 (m, 2H), 2.00 (ddd, *J* = 13.0, 5.2, 2.2 Hz, 1H), 1.60 – 1.51 (m, 1H), 1.43 (dt, *J* = 13.0, 11.3 Hz, 1H), 1.33 (ddd, *J* = 14.0, 9.6, 3.7 Hz, 1H), 1.00 (d, *J* = 6.8 Hz, 3H). ^{13}C NMR (101 MHz, CD_2Cl_2) δ 160.6, 137.6, 130.6, 128.9, 127.9, 113.9, 102.1, 84.4, 74.6, 70.7, 69.9, 69.4, 63.8, 55.7, 43.0, 39.8, 33.4, 21.3. IR (film) ṽ 2955, 2924, 2867, 1518, 1378, 1249, 1173, 1096, 1031, 973, 829 cm^{-1}. HRMS (ESI$^+$) calcd. for $C_{20}H_{28}O_6Na$ $[M+Na]^+$: 387.17781; found: 387.17773.

Compound 54. TBSOTf (2.40 mL, 10.5 mmol) was added dropwise to a solution of diol **S18** (940 mg, 2.58 mmol) and 2,6-lutidine (2.40 mL, 20.6 mmol) in CH_2Cl_2 (125 mL) at –78 °C. Stirring was continued for 1 h before sat. aq. $NaHCO_3$ (100 mL) was introduced. The mixture was warmed to ambient temperature and the layers were separated. The aqueous phase was extracted with *tert*-butyl methyl ether (3 × 100 mL). The combined organic layers were washed with brine and dried over Na_2SO_4. The solvent was removed under reduced pressure and the residue was purified by flash chromatography (silica, hexanes/*tert*-butyl methyl ether 10:1) to provide the title compound as a colorless oil (1.49 g, 95%). $[\alpha]_{20}^{D}$ = –43.8 (c = 1.00, CH_2Cl_2). ^{1}H NMR (400 MHz, CD_2Cl_2) δ 7.78 – 7.69 (m, 2H), 7.28 – 7.17 (m, 2H), 5.92 – 5.75 (m, 3H), 4.55 (dd, *J* = 10.3, 4.8 Hz, 1H), 4.47 (dt, *J* = 4.8, 0.9 Hz, 2H), 4.20 (ddd, *J* = 10.8, 8.5, 5.3 Hz, 1H), 4.14 (s, 3H), 3.99 (t, *J* = 10.1 Hz, 1H), 3.92 – 3.83 (m, 1H), 3.72 – 3.55 (m, 2H), 2.81 – 2.67 (m, 1H), 2.24 (ddd, *J* = 13.2, 5.3, 2.1 Hz, 1H), 1.88 – 1.76 (m, 2H), 1.68 – 1.59 (m, 1H), 1.33 (d, *J* = 6.8 Hz, 3H), 1.26 (s, 9H), 1.21 (s, 9H), 0.42 (s, 6H), 0.40 (s, 3H), 0.36 (s, 3H). ^{13}C NMR (101 MHz, CD_2Cl_2) δ 160.3, 136.1, 131.0, 129.1, 127.8, 113.6, 101.7, 84.4, 74.6, 71.2, 70.8, 69.4, 64.2, 55.6, 43.1, 41.9, 33.3, 26.1, 25.9, 21.4, 18.7, 18.5, –4.3, –4.7, –5.0. IR (film) ṽ 2953, 2928, 2856, 1250, 1103, 1071, 1038, 859, 835, 777 cm^{-1}. HRMS (ESI$^+$) calcd. for $C_{32}H_{56}O_6Si_2Na$ $[M+Na]^+$: 615.35076; found: 615.35072.

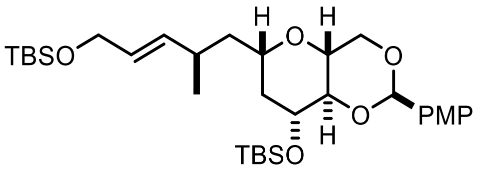

Compound S19. A solution of Dibal-H (1 M in CH_2Cl_2, 12.3 mL, 12.3 mmol) was added dropwise to a solution of acetal **54** (1.45 g, 2.45 mmol) in CH_2Cl_2 (64 mL) at –78 °C. Stirring was continued for 4 h before MeOH (0.5 mL), Rochelle´s salt solution (200 mL) and EtOAc (100 mL) were added. The resulting mixture was vigorously stirred overnight at ambient temperature. The layers were separated and the aqueous phase was extracted with EtOAc (3 × 100 mL). The combined organic layers were washed with brine and dried over Na_2SO_4. The solvent

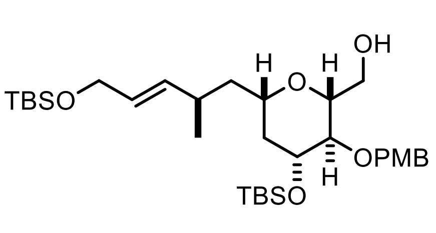

was removed under reduced pressure and the residue was purified by flash chromatography (silica, hexanes/*tert*-butyl methyl ether 4:1) to provide the title compound as a colorless oil (1.27 mg, 87%).

When carried out on smaller scale (192 mg) under otherwise identical conditions, the reaction provided the desired product in 93% yield.

$[\alpha]_{20}^{D}$ = –9.8 (c = 1.00, CH_2Cl_2). ^1H NMR (400 MHz, CD_2Cl_2) δ 7.29 – 7.20 (m, 2H), 6.90 – 6.82 (m, 2H), 5.56 – 5.40 (m, 2H), 4.80 (d, *J* = 10.9 Hz, 1H), 4.50 (d, *J* = 10.9 Hz, 1H), 4.14 – 4.08 (m, 2H), 3.84 – 3.68 (m, 5H), 3.54 (dt, *J* = 10.9, 5.1 Hz, 1H), 3.40 (tdd, *J* = 11.2, 3.5, 1.9 Hz, 1H), 3.21 – 3.10 (m, 2H), 2.39 (dtd, *J* = 9.7, 6.8, 4.7 Hz, 1H), 1.89 (t, *J* = 6.4 Hz, 1H), 1.82 (ddd, *J* = 12.9, 5.1, 1.9 Hz, 1H), 1.57 – 1.46 (m, 1H), 1.44 – 1.24 (m, 2H), 0.98 (d, *J* = 6.8 Hz, 3H), 0.93 (s, 9H), 0.90 (s, 9H), 0.10 (s, 3H), 0.09 (s, 3H), 0.06 (s, 6H). ^{13}C NMR (101 MHz, CD_2Cl_2) δ 159.6, 136.2, 131.3, 129.9, 128.9, 114.0, 80.4, 79.2, 74.9, 74.8, 73.5, 64.1, 63.2, 55.6, 43.1, 41.9, 33.5, 26.1, 26.1, 21.4, 18.7, 18.2, –4.2, –4.4, –5.0. IR (film) ṽ 2953, 2928, 2884, 2856, 1514, 1249, 1091, 1060, 1040, 972, 836, 776 cm^{-1}. HRMS (ESI^+) calcd. for $C_{32}H_{58}O_6Si_2Na$ $[M+Na]^+$: 617.36641; found: 617.36628.

Compound 55. A solution of DMSO (700 µL, 9.85 mmol) in CH_2Cl_2 (1 mL) was added to a solution of

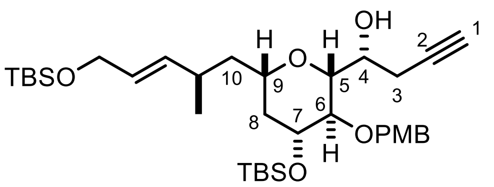

oxalyl chloride (350 µL, 4.08 mmol) in CH_2Cl_2 (30 mL) at –78 °C. Stirring was continued for 15 min, before a solution of alcohol **S19** (1.16 g, 1.95 mmol) in CH_2Cl_2 (10 mL) was added. The mixture was stirred for 15 min, before NEt_3 (1.4 mL, 10.0 mmol) was introduced. After additional 15 min, the mixture was warmed to 0 °C and stirred for another 20 min. Sat. aq. NH_4Cl (50 mL) was added and the layers were separated. The aqueous phase was extracted with *tert*-butyl methyl ether (3 × 100 mL), the combined organic layers were washed with brine and dried over Na_2SO_4. The solvent was removed under reduced pressure to provide the crude aldehyde, which was directly used in the next step without further purification.

A solution of the crude aldehyde in toluene (50 mL) was added to a solution of (*R*)-(+)-3,3′-dibromo-1,1′-bi-2-naphthol (**39**, 305 mg, 687 µmol) and allenyl boronate **38** (362 mg, 2.92 mmol) in toluene (7.5 mL). The resulting mixture was stirred overnight before the solvent was removed under reduced pressure. The residue was purified by flash chromatography (fine silica, hexanes/*tert*-butyl methyl ether 8:1) to provide the title compound as a colorless oil (548 mg, dr > 20:1, 44% over two steps).

When carried out on smaller scale (100 mg) under otherwise identical conditions, the reaction provided the desired product in 59% yield over two steps.

$[\alpha]_{20}^{D}$ = 6.6 (c = 0.50, CH_2Cl_2). ^1H NMR (400 MHz, CD_2Cl_2) δ 7.31 – 7.24 (m, 2H), 6.92 – 6.82 (m, 2H), 5.59 – 5.50 (m, 1H), 5.43 (ddt, *J* = 15.3, 8.2, 1.4 Hz, 1H), 4.96 (d, *J* = 10.9 Hz, 1H), 4.60 (d, *J* = 10.9 Hz, 1H), 4.11 (dd, *J* = 5.0, 1.4 Hz, 2H), 3.85 (ddd, *J* = 11.1, 8.3, 5.1 Hz, 1H), 3.81 – 3.75 (m, 4H), 3.37 (dddd, *J* =

12.1, 9.6, 2.7, 2.7 Hz, 1H), 3.27 (d, J = 3.9 Hz, 1H), 3.25 (dd, J = 9.4, 8.3 Hz, 1H), 3.13 (dd, J = 9.4, 6.0 Hz, 1H), 2.44 – 2.33 (m, 3H), 1.97 (t, J = 2.7 Hz, 1H), 1.82 (ddd, J = 13.0, 5.1, 2.0 Hz, 1H), 1.47 (ddd, J = 13.9, 9.6, 4.3 Hz, 1H), 1.38 (dt, J = 13.0, 11.3 Hz, 1H), 1.31 – 1.20 (m, 1H), 0.97 (d, J = 6.8 Hz, 3H), 0.93 (s, 9H), 0.90 (s, 9H), 0.13 (s, 3H), 0.11 (s, 3H), 0.06 (s, 6H). ^{13}C NMR (101 MHz, CD_2Cl_2) δ 159.9, 135.9, 130.5, 130.1, 129.2, 114.2, 83.3, 82.3, 78.9, 75.4, 74.9, 73.7, 71.9, 69.6, 64.1, 55.6, 43.2, 42.0, 33.5, 26.1, 26.1, 23.1, 21.5, 18.7, 18.2, –4.0, –4.3, –5.0. IR (film) ṽ 2954, 2928, 2896, 2857, 1515, 1463, 1251, 1087, 1040, 836, 776 cm^{-1}. HRMS (ESI$^+$) calcd. for $C_{35}H_{60}O_6Si_2Na$ [M+Na]$^+$: 655.38206; found: 655.38195.

The absolute configuration was determined by Mosher ester analysis:

Preparation of the (*S*)- and (*R*)-MTPA Esters of Alcohol 55. (*R*)-(–)-MTPA-Cl (4.0 µL, 21 µmol) was added to a solution of alcohol **55** (2.0 mg, 3.2 µmol), NEt_3 (5.0 µL, 36 µmol) and DMAP (0.2 mg, 1.6 µmol) in CH_2Cl_2 (0.5 mL). The mixture was stirred overnight, before sat. aq. $NaHCO_3$ (5 mL) was added. The mixture was extracted with *tert*-butyl methyl ether (3 × 10 mL) and the combined organic layers were washed with brine and dried over Na_2SO_4. The residue was purified by flash chromatography (silica, hexanes/*tert*-butyl methyl ether 10:1) to provide the corresponding (*S*)-MTPA ester as a colorless oil (1.2 mg, 45%); for the spectral data, see Table S8.

The (*R*)-MTPA ester was prepared analogously using (*S*)-(+)-MTPA-Cl as the reagent; for the spectral data, see Table S8.

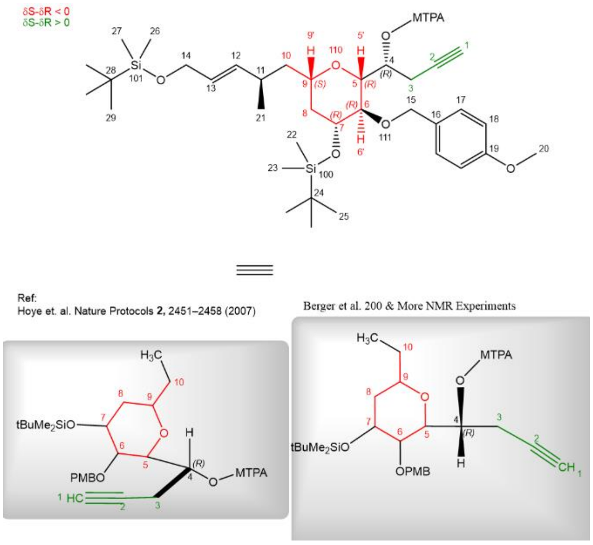

Table S8. Determination of absolute configuration of the newly formed chiral center in homopropargylic alcohol **55** via Mosher ester analysis.[9] The recorded NMR data ($CDCl_3$) suggest that the chiral center C4 is *(R)*-configured. Arbitrary numbering scheme as shown in the insert.

Atom number	*(S)-ester* δ [ppm]	*(R)-ester* δ [ppm]	$\Delta\delta^{SR}$
1 C	70.52	70.45	
H	2.01	1.90	0.11
2 C	81.05	80.49	
3 C	19.50	19.44	
H'	2.22	2.22	0.00
H''	2.61	2.56	0.06
4 C	75.11	75.16	
H	5.53	5.51	0.02
5 C	78.86	78.59	

5' H	3.24	3.39	-0.15
6 C	79.00	79.35	
6' H	2.92	3.14	-0.22
7 C	75.24	75.30	
H	3.73	3.79	-0.07
8 C	41.50	41.66	
Hax	1.22	1.32	-0.10
Heq	1.74	1.82	-0.08
9 C	73.95	73.86	
9' H	3.21	3.37	-0.15
10 C	43.00	43.15	
H'	1.38	1.47	-0.10
H''	1.24	1.31	-0.07
11 C	33.43	33.36	
H	2.23	2.33	-0.10
12 C	136.32	136.10	
H	5.40	5.46	-0.06
13 C	128.48	128.79	
H	5.40	5.46	-0.06
14 C	64.11	64.07	
H2	4.04	4.08	-0.04
15 C	74.47	74.48	
H'	4.49	4.47	0.03
H''	4.79	4.84	-0.05
16 C	130.71	130.64	
17 C	130.02	129.97	
H	7.29	7.22	0.07
18 C	114.26	114.19	
H	6.90	6.87	0.03
19 C	159.74	159.68	
20 C	55.59	55.57	
H3	3.80	3.79	0.01
21 C	21.25	21.42	
H3	0.91	0.96	-0.05
22 C	-4.38	-4.34	
H3	0.10	0.11	-0.01
23 C	-4.14	-4.15	
H3	0.08	0.09	-0.02
24 C	18.16	18.19	
25 C	26.04	26.05	
H3	0.92	0.92	0.00
26 C	-5.01	-5.02	
H3	0.04	0.05	-0.01
27 C	-5.01	-5.02	
H3	0.04	0.05	-0.01

28 C	18.64	18.65	
29 C	26.11	26.11	
H3	0.89	0.89	0.00
30 C	166.11	166.12	
31 C	84.90	85.08	
32 C	132.90	132.76	
33 C	127.85	128.02	
H	7.60	7.59	0.00
34 C	128.70	128.70	
H	7.41	7.40	0.01
35 C	130.08	130.01	
H	7.42	7.42	0.00
36 C	123.85	123.85	
36' F	-71.93	-72.05	
37 C	56.15	55.90	
H3	3.61	3.56	0.05
100 Si	19.03	19.27	
101 Si	19.43	19.49	-0.06

Compound S20. Acetic anhydride (460 µL, 4.87 mmol), pyridine (480 µL, 5.93 mmol) and DMAP (235 mg, 1.93 mmol) were added to a solution of homopropargylic alcohol **55** (610 mg, 964 µmol) in CH_2Cl_2 (60 mL) at 0 °C. The mixture was stirred at ambient temperature overnight. Sat. aq. $NaHCO_3$ (50 mL) and brine (50 mL) were introduced and the mixture was extracted with *tert*-butyl methyl ether (3 x 100 mL). The combined organic layers were washed with brine and dried over Na_2SO_4. The solvent was removed under reduced pressure to provide the title compound as a colorless oil (583 mg, 90%). $[\alpha]_{20}^{D}$ = –19.8 (c = 1.00, CH_2Cl_2). ^{1}H NMR (400 MHz, CD_2Cl_2) δ 7.34 – 7.28 (m, 2H), 6.91 – 6.86 (m, 2H), 5.53 (dt, *J* = 15.2, 5.2 Hz, 1H), 5.41 (ddt, *J* = 15.3, 8.3, 1.4 Hz, 1H), 5.27 (ddd, *J* = 8.9, 4.9, 1.8 Hz, 1H), 4.85 (d, *J* = 10.9 Hz, 1H), 4.60 (d, *J* = 11.0 Hz, 1H), 4.10 (dt, *J* = 5.3, 1.7 Hz, 2H), 3.83 – 3.71 (m, 4H), 3.34 – 3.24 (m, 2H), 3.12 (dd, *J* = 9.9, 8.1 Hz, 1H), 2.51 (ddd, *J* = 17.1, 8.9, 2.7 Hz, 1H), 2.47 – 2.35 (m, 1H), 2.18 (ddd, *J* = 17.0, 4.9, 2.7 Hz, 1H), 2.02 (s, 3H), 1.96 (t, *J* = 2.7 Hz, 1H), 1.79 (ddd, *J* = 12.9, 5.1, 1.9 Hz, 1H), 1.48 (ddd, *J* = 14.1, 9.5, 4.7 Hz, 1H), 1.34 (dt, *J* = 12.9, 11.3 Hz, 1H), 1.29 – 1.20 (m, 1H), 0.98 (d, *J* = 6.8 Hz, 3H), 0.93 (s, 9H), 0.89 (s, 9H), 0.11 (s, 3H), 0.09 (s, 3H), 0.05 (s, 6H). ^{13}C NMR (101 MHz, CD_2Cl_2) δ 170.4, 159.7, 136.1, 130.9, 130.0, 129.1, 114.2, 81.3, 79.7, 79.2, 75.5, 74.5, 73.5, 71.7, 69.9, 64.2, 55.6, 42.9, 41.6, 33.5, 26.1, 26.1, 21.3, 21.2, 19.0, 18.6, 18.2, –4.2, –4.4, –5.0, –5.0. IR (film) ṽ 2954, 2929, 2856,

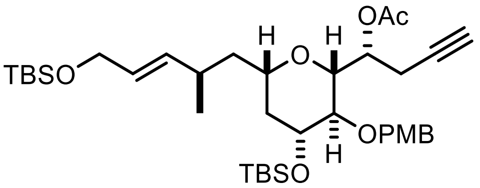

1744, 1514, 1250, 1092, 1057, 1035, 837, 776 cm^{-1}. HRMS (ESI^{+}) calcd. for $C_{37}H_{62}O_7Si_2Na$ [M+Na]$^{+}$: 697.39263; found: 697.39232.

Compound 56. pH 7 Phosphate buffer solution (10 mL) and 2,3-dichloro-5,6-dicyano-1,4-benzoquinone (292 mg, 1.28 mmol) were added to a solution of PMB ether **S20** (578 mg, 856 µmol) in CH_2Cl_2 (40 mL) at 0 °C. The mixture was stirred at ambient temperature for 85 min. Additional 2,3-dichloro-5,6-dicyano-1,4-benzoquinone (292 mg, 1.28 mmol) was added in two portions over the course of 60 min. Brine (30 mL) and sat. aq. $NaHCO_3$ (100 mL) were introduced, the layers were separated and the aqueous layer was extracted with *tert*-butyl methyl ether (3 × 100 mL). The combined organic layers were washed with brine and dried over Na_2SO_4. The solvent was removed under reduced pressure and the residue was purified by flash chromatography (fine silica, hexanes/*tert*-butyl methyl ether 5:1) to provide the title compound as yellow oil (366 mg, 77%). $[\alpha]_{20}^{D}$ = –22.4 (c = 0.70, CH_2Cl_2). ^{1}H NMR (400 MHz, CD_2Cl_2) δ 5.54 (dt, *J* = 15.3, 5.3 Hz, 1H), 5.41 (ddt, *J* = 15.3, 8.4, 1.4 Hz, 1H), 5.27 (ddd, *J* = 7.0, 6.0, 3.1 Hz, 1H), 4.11 (d, *J* = 4.9 Hz, 2H), 3.57 (ddd, *J* = 11.1, 7.6, 5.0 Hz, 1H), 3.41 – 3.33 (m, 1H), 3.33 – 3.23 (m, 2H), 2.62 (dd, *J* = 2.7, 1.6 Hz, 1H), 2.60 (d, *J* = 2.7 Hz, 1H), 2.51 – 2.35 (m, 1H), 2.28 (d, *J* = 2.2 Hz, 1H), 2.06 (s, 3H), 2.00 (t, *J* = 2.7 Hz, 1H), 1.77 (ddd, *J* = 12.8, 5.0, 1.9 Hz, 1H), 1.53 – 1.46 (m, 1H), 1.33 (dt, *J* = 12.8, 11.3 Hz, 1H), 1.25 (ddd, *J* = 13.6, 9.7, 3.1 Hz, 1H), 0.98 (d, *J* = 6.8 Hz, 3H), 0.90 (s, 9H), 0.89 (s, 9H), 0.10 (s, 3H), 0.08 (s, 3H), 0.06 (s, 6H). ^{13}C NMR (101 MHz, CD_2Cl_2) δ 170.5, 136.1, 129.2, 81.0, 78.6, 75.2, 74.2, 73.9, 71.9, 69.9, 64.2, 43.0, 40.7, 33.5, 26.1, 25.9, 21.4, 21.3, 19.7, 18.7, 18.3, –4.1, –4.5, –5.0. IR (film) ṽ 2953, 2929, 2857, 1742, 1251, 1130, 1098, 1036, 836, 777 cm^{-1}. HRMS (ESI^{+}) calcd. for $C_{29}H_{54}O_6Si_2Na$ [M+Na]$^{+}$: 577.33511; found: 577.33512.

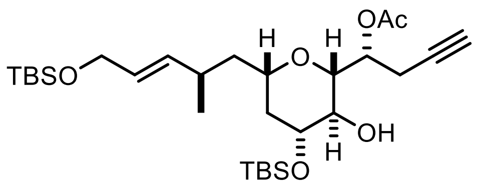

NOMINAL PROROCENTIN

Enyne 65. DIPEA (26 µL, 149 µmol), CuI (1.1 mg, 5.8 µmol), PPh_3 (1.6 mg, 6.1 µmol), and $Pd_2(dba)_3$ (1.4 mg, 1.5 µmol) were added to a solution of alkenyl iodide **18** (11.0 mg, 30 µmol) and alkyne **35** (18.3 mg, 33 µmol) in degassed THF (1.0 mL). The mixture was stirred for 48 h, before sat. aq. NH_4Cl (2 mL) was added. After separation of the layers, the aqueous phase was extracted with EtOAc (3 × 10 mL). The combined organic layers were washed with brine and dried over Na_2SO_4. The solvent was removed under reduced pressure and the residue was purified by flash chromatography (silica,

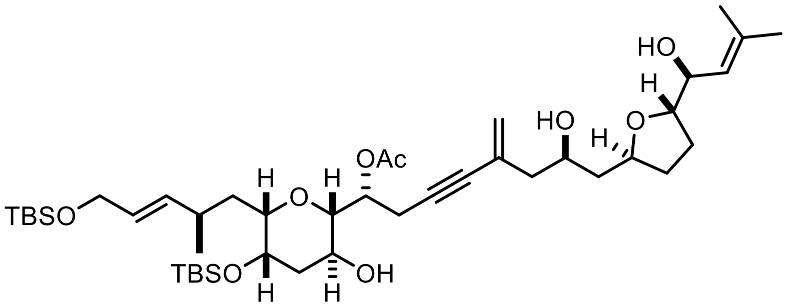

hexanes/EtOAc 3:2 → 1:1) to provide the title compound as a pale yellow oil (23.8 mg, 99%). $[\alpha]_{20}^{D}$ = −17.2 (c = 1.00, CH_2Cl_2). ^{1}H NMR (400 MHz, C_6D_6) δ 5.81 (ddd, *J* = 7.0, 5.4, 2.8 Hz, 1H), 5.72 (dt, *J* = 15.3, 5.2 Hz, 1H), 5.56 (ddt, *J* = 15.3, 8.4, 1.6 Hz, 1H), 5.45 (d, *J* = 2.3 Hz, 1H), 5.28 (dqq, *J* = 8.9, 1.5, 1.5 Hz, 1H), 5.15 (d, *J* = 2.3 Hz, 1H), 4.35 – 4.26 (m, 2H), 4.21 (dd, *J* = 8.9, 6.9 Hz, 1H), 4.16 (dd, *J* = 5.2, 1.6 Hz, 2H), 4.00 (dddd, *J* = 9.1, 9.1, 5.5, 3.4 Hz, 1H), 3.88 (ddd, *J* = 7.0, 7.0, 6.9 Hz, 1H), 3.72 (brs, 1H), 3.66 (brs, 1H), 3.54 (dd, *J* = 9.8, 2.8 Hz, 1H), 3.39 (t, *J* = 3.2 Hz, 1H), 3.27 (dd, *J* = 10.7, 3.2 Hz, 1H), 3.18 (dd, *J* = 17.2, 7.0 Hz, 1H), 3.17 (brs, 1H), 2.83 (dd, *J* = 17.2, 5.4 Hz, 1H), 2.64 – 2.47 (m, 1H), 2.35 (dd, *J* = 13.6, 7.9 Hz, 1H), 2.28 (dd, *J* = 13.6, 4.9 Hz, 1H), 2.17 (ddd, *J* = 13.1, 4.8, 3.4 Hz, 1H), 1.94 – 1.85 (m, 1H), 1.84 (s, 3H), 1.73 – 1.63 (m, 1H), 1.61 (s, 3H), 1.60 (s, 3H), 1.59 – 1.39 (m, 5H), 1.24 – 1.11 (m, 2H), 1.04 (d, *J* = 6.7 Hz, 3H), 1.00 (s, 18H), 0.10 (s, 3H), 0.09 (s, 3H), 0.04 (s, 3H), 0.00 (s, 3H). ^{13}C NMR (101 MHz, C_6D_6) δ 170.0, 136.3, 136.1, 129.6, 129.1, 125.1, 122.2, 88.1, 83.4, 82.7, 79.2, 77.9, 72.7, 71.0, 70.1, 70.0, 64.2, 63.3, 45.7, 42.0, 41.9, 39.4, 33.6, 33.0, 27.6, 26.2, 26.1, 26.0, 21.5, 20.9, 20.2, 18.7, 18.6, 18.4, −4.3, −4.7, −4.8, −4.9. IR (film) ṽ 3416, 2954, 2929, 2857, 1738, 1462, 1374, 1252, 1103, 1070, 1038, 971, 835, 775 cm^{-1}. HRMS (ESI$^{+/-}$) calcd. for $C_{43}H_{76}O_9Si_2Na$ [M+Na]$^+$: 815.49201; found: 815.49176.

Spiroketal 67. PPTS (1.4 mg, 5.75 µmol) and (JohnPhos)Au(MeCN)SbF_6 (**36**, 4.4 mg, 5.75 µmol) were added to a solution of enyne **65** (45.6 mg, 57.5 µmol) in CH_2Cl_2 (6.2 mL). The mixture was stirred for 15 min before sat. aq. NH_4Cl (10 mL) was introduced. The layers were separated and the aqueous phase was extracted with *tert*-butyl methyl ether (3 x 10 mL). The combined extracts were dried over Na_2SO_4 and concentrated. The residue was purified by flash chromatography (CH_2Cl_2/hexanes/*tert*-butyl methyl ether 4:3:1 + 1% NEt_3) to provide the title compound as a colorless oil (38.5 mg, 84%, dr >20:1). $[\alpha]_{20}^{D}$ = −54.1 (c = 1.00, CH_2Cl_2). ^{1}H NMR (400 MHz, C_6D_6) δ 5.69 (dt, *J* = 15.3, 5.1 Hz, 1H), 5.63 – 5.54 (m, 1H), 5.41 (q, *J* = 3.2 Hz, 1H), 5.33 (dp, *J* = 8.7, 1.4 Hz, 1H), 5.25 (p, *J* = 1.1 Hz, 1H), 4.67 (ddd, *J* = 11.8, 9.6, 4.7 Hz, 1H), 4.28 – 4.09 (m, 5H), 3.98 (q, *J* = 7.1 Hz, 1H), 3.48 (t, *J* = 3.5 Hz, 1H), 3.38 (ddd, *J* = 9.5, 3.2, 1.1 Hz, 1H), 3.13 (dd, *J* = 9.6, 3.0 Hz, 1H), 2.58 (dt, *J* = 14.3, 7.5 Hz, 1H), 2.46 (dd, *J* = 15.0, 3.0 Hz, 1H), 2.36 (d, *J* = 2.8 Hz, 1H), 2.20 – 2.10 (m, 1H), 2.06 (ddd, *J* = 13.2, 7.6, 5.3 Hz, 1H), 2.00 – 1.86 (m, 2H), 1.90 (s, 3H), 1.85 – 1.70 (m, 2H), 1.67 – 1.52 (m, 11H), 1.49 (s, 3H), 1.41 (ddt, *J* = 11.9, 9.1, 8.0 Hz, 1H), 1.23 (ddd, *J* = 13.9, 9.7, 3.3 Hz, 1H), 1.11 (s, 9H), 1.01 (s, 9H), 0.98 (d, *J* = 6.8 Hz, 3H), 0.14 (s, 3H), 0.10 (s, 6H), 0.06 (s, 3H). ^{13}C NMR (151 MHz, C_6D_6) δ 170.4, 136.5, 136.3, 136.0, 128.9, 125.2, 124.9, 95.4, 82.9, 78.6, 78.1, 75.9, 71.5, 70.3, 68.3, 65.2, 64.2, 60.8, 42.3, 39.6, 38.9, 38.5, 34.9, 33.3, 33.2, 28.2, 26.2, 26.2, 26.0, 22.8, 21.5, 21.2, 18.7, 18.6, 18.5,

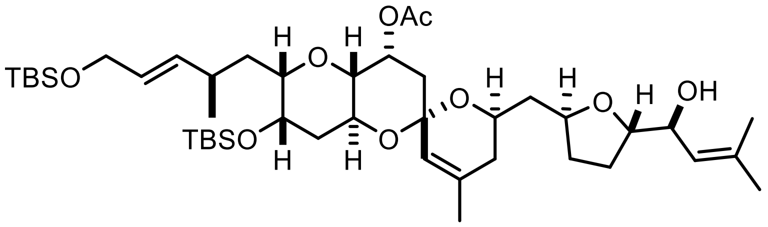

−4.1, −4.7, −4.9 (2 x). IR (film) ṽ 3479, 2953, 2928, 2856, 1736, 1462, 1376, 1250, 1199, 1090, 963, 833, 773 cm^{-1}. HRMS (ESI$^{+/-}$) calcd. for $C_{43}H_{76}O_9Si_2Na$ [M+Na]$^+$: 815.49201; found: 815.49171.

Compound S21. 2,6-Lutidine (17 µL, 146 µmol) and TBSOTf (17 µL, 74.0 µmol) were added to a solution of spiroketal **67** (43.6 mg, 55.0 µmol) in CH_2Cl_2 (1.5 mL) at 0 °C. The mixture was stirred for 90 min before sat. aq. NH_4Cl (15 mL) was introduced. The layers were separated and the aqueous phase was extracted with *tert*-butyl methyl ether (3 x 15 mL). The combined organic extracts were dried over Na_2SO_4 and concentrated to provide the title compound as a colorless oil (33.0 mg, 66%).

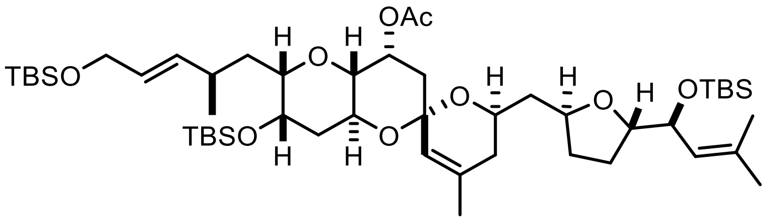

$[\alpha]_{20}^{D}$ = −44.4 (c = 1.00, CH_2Cl_2). ^1H NMR (400 MHz, C_6D_6) δ 5.68 (dt, *J* = 15.3, 5.0 Hz, 1H), 5.59 (ddt, *J* = 15.3, 8.0, 1.3 Hz, 1H), 5.41 (q, *J* = 3.1 Hz, 1H), 5.31 (dqq, *J* = 9.1, 1.4, 1.4 Hz, 1H), 5.27 – 5.24 (m, 1H), 4.69 (ddd, *J* = 11.9, 9.6, 4.8 Hz, 1H), 4.38 (dd, *J* = 9.2, 6.0 Hz, 1H), 4.31 – 4.23 (m, 1H), 4.21 – 4.05 (m, 4H), 3.49 (t, *J* = 3.4 Hz, 1H), 3.38 (ddd, *J* = 9.5, 3.3, 1.1 Hz, 1H), 3.14 (dd, *J* = 9.6, 3.0 Hz, 1H), 2.65 – 2.52 (m, 1H), 2.47 (dd, *J* = 14.9, 3.0 Hz, 1H), 2.16 (dt, *J* = 12.7, 4.5 Hz, 1H), 2.01 (td, *J* = 8.5, 4.2 Hz, 1H), 1.98 – 1.84 (m, 3H), 1.92 (s, 3H), 1.82 – 1.49 (m, 6H), 1.60 (s, 3H), 1.60 (s, 3H), 1.56 (s, 3H), 1.48 – 1.37 (m, 1H), 1.23 (ddd, *J* = 13.9, 9.7, 3.3 Hz, 1H), 1.12 (s, 9H), 1.05 (s, 9H), 1.00 (s, 9H), 0.98 (d, *J* = 6.8 Hz, 3H), 0.19 (s, 3H), 0.16 (s, 3H), 0.15 (s, 3H), 0.09 (s, 6H), 0.08 (s, 3H). ^{13}C NMR (101 MHz, C_6D_6) δ 170.4, 136.5, 136.2, 133.3, 128.9, 126.3, 124.9, 95.5, 83.1, 78.5, 78.1, 75.7, 73.0, 70.3, 68.4, 65.4, 64.2, 60.7, 42.1, 39.7, 38.9, 38.4, 34.8, 33.4, 33.3, 28.1, 26.3, 26.2, 26.0, 22.9, 21.5, 21.3, 18.7, 18.6, 18.5, −4.1, −4.2, −4.3, −4.6, −4.9. IR (film) ṽ 2954, 2928, 2856, 1738, 1462, 1376, 1251, 1200, 1091, 1066, 965, 833, 774 cm^{-1}. HRMS (ESI$^{+/-}$) calcd. for $C_{49}H_{90}O_9Si_3Na$ [M+Na]$^+$: 929.57848; found: 929.57878.

Allylic alcohol S22. HF·pyridine (40 µL) was added to a solution of silyl ether **S21** (33.0 mg, 36.4 µmol) in pyridine (1.0 mL) at 0 °C. The mixture was warmed to ambient temperature and stirring was continued for 20 h before sat. aq. $NaHCO_3$ (1 mL) and EtOAc (1 mL) were introduced. After separation of the layers, the aqueous phase was extracted with EtOAc (3 × 1 mL). The combined extracts were washed with brine and dried over Na_2SO_4. The solvent was removed under reduced pressure and the residue was purified by flash chromatography (silica, hexanes/*tert*-butyl methyl ether 2:1 → 1:1) to provide the title compound as a colorless oil (26.2 mg, 91%).

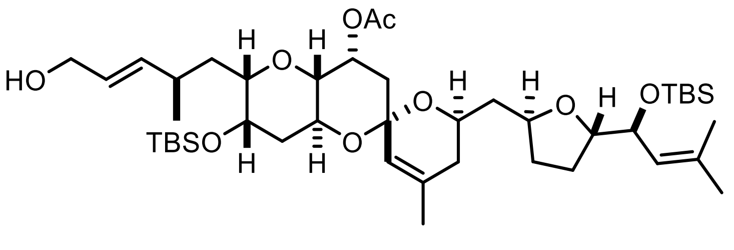

$[\alpha]_{20}^{D}$ = −36.4 (c = 1.00, CH_2Cl_2). ^1H NMR (400 MHz, C_6D_6) δ 5.62 (dt, *J* = 15.8, 5.3 Hz, 1H), 5.50 (ddt, *J* = 15.3, 8.1, 1.3

Hz, 1H), 5.40 (q, *J* = 3.0 Hz, 1H), 5.32 (dp, *J* = 9.1, 1.4 Hz, 1H), 5.25 (dt, *J* = 2.5, 1.1 Hz, 1H), 4.66 (ddd, *J* = 11.9, 9.6, 4.8 Hz, 1H), 4.38 (dd, *J* = 9.1, 6.1 Hz, 1H), 4.32 – 4.20 (m, 1H), 4.19 – 4.06 (m, 2H), 3.95 (dd, *J* = 5.3, 1.3 Hz, 2H), 3.44 (t, *J* = 3.4 Hz, 1H), 3.31 (ddd, *J* = 9.8, 3.0, 1.1 Hz, 1H), 3.09 (dd, *J* = 9.6, 3.0 Hz, 1H), 2.61 – 2.47 (m, 1H), 2.41 (dd, *J* = 15.0, 3.0 Hz, 1H), 2.13 (dt, *J* = 12.7, 4.1 Hz, 1H), 2.02 (ddd, *J* = 13.4, 8.6, 4.8 Hz, 1H), 1.98 – 1.84 (m, 3H), 1.92 (s, 3H), 1.84 – 1.61 (m, 5H), 1.60 (s, 3H), 1.60 (s, 3H), 1.57 (s, 3H), 1.50 (td, *J* = 12.7, 2.5 Hz, 1H), 1.41 (ddt, *J* = 11.8, 10.0, 8.2 Hz, 1H), 1.23 – 1.12 (m, 1H), 1.11 (s, 9H), 1.05 (s, 9H), 0.96 (d, *J* = 6.8 Hz, 3H), 0.19 (s, 3H), 0.15 (s, 6H), 0.07 (s, 3H). ^{13}C NMR (101 MHz, C_6D_6) δ 170.5, 137.1, 136.2, 133.3, 129.0, 126.3, 124.8, 95.4, 83.1, 78.3, 78.0, 75.7, 73.0, 70.4, 68.3, 65.4, 63.5, 60.7, 42.1, 39.6, 38.9, 38.4, 34.8, 33.4, 33.3, 28.0, 26.3, 26.2, 26.0, 22.9, 21.4, 21.3, 18.7, 18.6, 18.5, –4.1, –4.2, –4.3, –4.6. IR (film) ṽ 3459, 2954, 2928, 2856, 1736, 1462, 1376, 1252, 1200, 1089, 965, 835, 774 cm^{-1}. HRMS (ESI$^{+/-}$) calcd. for $C_{43}H_{76}O_9Si_2Na$ [M+Na]$^+$: 815.49092; found: 815.49201.

Compound S23. A solution of (+)-diisopropyl L-tartrate (5 µL, 23.7 µmol) in CH_2Cl_2 (150 µL) was added to a suspension of powdered molecular sieves 4Å (10 mg) in CH_2Cl_2 (200 µL) at –30 °C. A solution of Ti(O*i*-Pr)$_4$ (5 µL, 16.9 µmol) in CH_2Cl_2 (150 µL) was then added and stirring was continued for 30 min. Cumene hydroperoxide (80% w/w, 18 µL, 97.5 µmol) was added and stirring was continued for another 30 min before a solution of allylic alcohol **S22** (25.1 mg, 31.6 µmol) in CH_2Cl_2 (500 µL) was introduced. The mixture was stirred at this temperature for another 3 h before the reaction was quenched upon addition of a solution of NaOH (50 mg) in brine (0.5 mL). The mixture was warmed to 0 °C and stirring was continued for 1 h. The suspension was filtered through a short pad of Celite, which was carefully rinsed with CH_2Cl_2 (20 mL). The combined filtrates were evaporated and the residue was purified by flash chromatography (silica, hexanes/*tert*-butyl methyl ether 1:1 + 1% NEt_3 → 2:3 + 1% NEt_3 → 1:2 + 1% NEt_3) to provide the title compound as a colorless oil (25.4 mg, 99%, dr > 20:1).

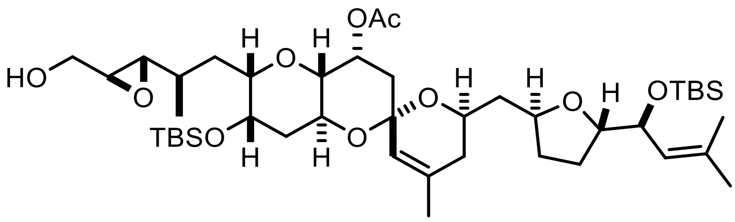

$[\alpha]_{20}^{D}$ = –30.1 (c = 1.00, CH_2Cl_2). ^{1}H NMR (400 MHz, C_6D_6) δ 5.36 – 5.29 (m, 2H), 5.27 (p, *J* = 1.1 Hz, 1H), 4.65 (ddd, *J* = 11.9, 9.6, 4.7 Hz, 1H), 4.38 (dd, *J* = 9.1, 6.1 Hz, 1H), 4.31 – 4.21 (m, 1H), 4.18 – 4.06 (m, 2H), 3.55 (d, *J* = 3.9 Hz, 2H), 3.40 – 3.30 (m, 2H), 3.09 (dd, *J* = 9.7, 3.0 Hz, 1H), 2.92 (td, *J* = 3.9, 2.2 Hz, 1H), 2.70 (dd, *J* = 7.6, 2.2 Hz, 1H), 2.42 (dd, *J* = 14.9, 3.0 Hz, 1H), 2.13 (dt, *J* = 12.8, 4.4 Hz, 1H), 2.02 (ddd, *J* = 13.3, 8.6, 4.6 Hz, 1H), 1.98 – 1.85 (m, 3H), 1.94 (s, 3H), 1.83 – 1.61 (m, 6H), 1.60 (s, 3H), 1.60 (s, 3H), 1.58 (s, 3H), 1.49 (td, *J* = 12.7, 2.5 Hz, 1H), 1.45 – 1.36 (m, 1H), 1.30 (s, 1H), 1.22 – 1.12 (m, 1H), 1.10 (s, 9H), 1.05 (s, 9H), 0.99 (d, *J* = 6.7 Hz, 3H), 0.19 (s, 3H), 0.16 (s, 3H), 0.15 (s, 3H), 0.05 (s, 3H). ^{13}C NMR (101 MHz, C_6D_6) δ 170.5, 136.4, 133.3, 126.3, 124.7, 95.4, 83.1, 78.1, 77.6, 75.7, 73.0, 70.1, 68.2, 65.4, 62.5, 60.5, 60.3, 58.1, 42.1, 38.9, 38.3, 36.3, 34.8, 33.5, 32.2, 28.0, 26.2, 26.0, 22.9, 21.2,

18.7, 18.6, 18.5, 17.8, –4.1, –4.2, –4.3, –4.7. IR (film) ṽ 3450, 2955, 2928, 2856, 1737, 1462, 1376, 1252, 1201, 1090, 1060, 964, 834, 774 cm^{-1}. HRMS (ESI$^{+/-}$) calcd. for $C_{43}H_{76}O_{10}Si_2Na$ [M+Na]$^+$: 831.48692; found: 831.48624.

Iodide S24. A solution of epoxide **S23** (12.7 mg, 42.0 µmol) in CH_2Cl_2 (0.5 mL) was added to a mixture of imidazole (3.2 mg, 47.1 µmol) and PPh_3 (6.2 mg, 23.5 µmol). The resulting mixture was cooled to 0 °C before I_2 (6.0 mg, 23.5 µmol) was added in one portion, and stirring was continued for 5 h. *tert-B*utyl methyl ether (20 mL) was introduced and the mixture was successively washed with sat. aq. $Na_2S_2O_3$ (20 mL) and brine (20 mL). The combined organic layers were evaporated under reduced pressure and the residue was purified by flash chromatography (silica, hexanes/*tert*-butyl methyl ether 5:1 → 4:1) to provide the title compound as a colorless oil (13.2 mg, 92%). $[\alpha]_{20}^{D}$ = –26.4 (c = 1.00, CH_2Cl_2). ^{1}H NMR (400 MHz, CD_2Cl_2) δ 5.21 (p, *J* = 1.2 Hz, 1H), 5.13 (dp, *J* = 9.3, 1.4 Hz, 1H), 5.08 (q, *J* = 3.1 Hz, 1H), 4.40 (ddd, *J* = 11.9, 9.6, 4.7 Hz, 1H), 4.22 (dd, *J* = 9.1, 5.8 Hz, 1H), 4.08 (tt, *J* = 8.4, 5.0 Hz, 1H), 4.04 – 3.95 (m, 1H), 3.86 (td, *J* = 7.7, 5.8 Hz, 1H), 3.79 (t, *J* = 3.5 Hz, 1H), 3.64 (ddd, *J* = 10.0, 3.4, 1.2 Hz, 1H), 3.33 – 3.23 (m, 2H), 3.08 – 2.98 (m, 2H), 2.57 (dd, *J* = 8.0, 1.9 Hz, 1H), 2.11 (dd, *J* = 15.0, 3.0 Hz, 1H), 2.09 – 2.03 (m, 1H), 2.03 – 1.91 (m, 2H), 2.00 (s, 3H), 1.92 – 1.76 (m, 4H), 1.75 – 1.58 (m, 4H), 1.70 (d, *J* = 1.5 Hz, 3H), 1.69 (t, *J* = 1.2 Hz, 3H), 1.65 (d, *J* = 1.4 Hz, 3H), 1.56 – 1.39 (m, 2H), 1.35 – 1.24 (m, 1H), 1.00 (d, *J* = 6.8 Hz, 3H), 0.95 (s, 9H), 0.86 (s, 9H), 0.11 (s, 3H), 0.06 (s, 3H), 0.03 (s, 3H), 0.00 (s, 3H). ^{13}C NMR (101 MHz, CD_2Cl_2) δ 171.1, 137.6, 133.5, 126.1, 123.7, 95.3, 83.0, 77.7, 77.6, 75.7, 72.9, 69.9, 68.2, 66.9, 65.5, 60.5, 58.4, 41.8, 38.7, 38.0, 35.9, 34.7, 33.3, 32.6, 27.9, 26.1, 26.0, 26.0, 22.9, 21.5, 18.7, 18.5, 18.4, 17.9, 5.9, –4.3, –4.3, –4.6, –4.8. IR (film) ṽ 2955, 2928, 2855, 1737, 1462, 1375, 1252, 1201, 1091, 965, 835, 775 cm^{-1}. HRMS (ESI$^+$) calcd. for $C_{43}H_{76}IO_9Si_2$ [M+H]$^+$: 919.40672; found: 919.40742.

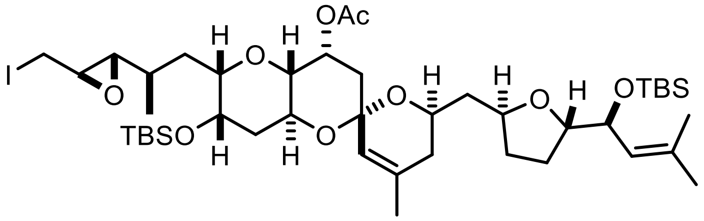

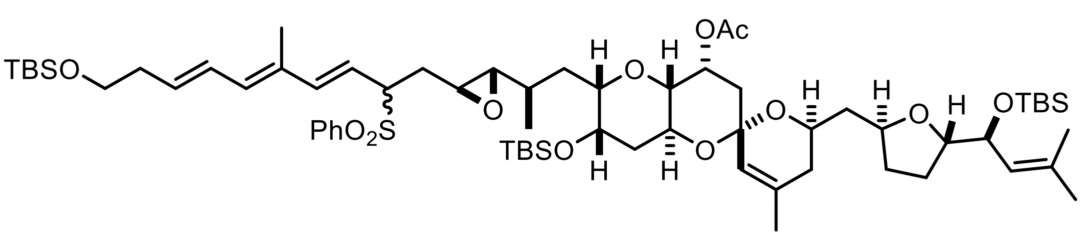

Compound S25. A solution of *n*-BuLi (1.6 M in hexanes, 46 µL, 74 µmol) was added dropwise to a solution of sulfone **8** (34.0 mg, 84 µmol) and DMPU (45 µL, 15 µmol) in THF (2 mL) at –80 °C. The mixture was stirred at this temperature for 60 min before a solution of iodide **S24** (22.6 mg, 24.6 µmol) in THF (1.3 mL) was added and stirring was continued for 30 min. The mixture was warmed to –60 °C and stirred for another 1 h 10 min. The reaction was quenched at this temperature by addition of sat.

aq. NH_4Cl (3 mL). After reaching ambient temperature, the mixture was extracted with EtOAc (3 × 15 mL). The combined organic layers were washed with brine and dried over Na_2SO_4. The solvent was removed under reduced pressure and the residue was purified by flash chromatography (silica, hexanes/*tert*-butyl methyl ether 3:1 + 1% NEt_3 → 2:1 + 1% NEt_3) to provide the title compound as an inseparable mixture of diastereoisomers (dr ≈ 3:2, 23.2 mg, 79%). $[\alpha]_{20}^{D}$ = –25.9 (c = 1.00, CH_2Cl_2). ^1H NMR (400 MHz, CD_2Cl_2) δ 7.84 – 7.71 (m, 2H), 7.71 – 7.60 (m, 1H), 7.54 (td, *J* = 7.0, 1.8 Hz, 2H), 6.40 (ddt, *J* = 15.2, 11.2, 1.5 Hz, 1H), 6.06 – 5.96 (m, 1H), 5.96 – 5.86 (m, 1H), 5.77 (dt, *J* = 14.8, 7.2 Hz, 1H), 5.48 – 5.33 (m, 1H), 5.27 – 5.17 (m, 1H), 5.17 – 5.09 (m, 1H), 5.09 – 4.95 (m, 1H), 4.44 – 4.30 (m, 1H), 4.28 – 4.17 (m, 1H), 4.14 – 4.04 (m, 1H), 4.04 – 3.94 (m, 1H), 3.91 – 3.82 (m, 1H), 3.82 – 3.70 (m, 2H), 3.70 – 3.60 (m, 2H), 3.46 – 3.31 (m, 1H), 3.15 – 3.04 (m, 1H), 2.87 (ddt, *J* = 4.1, 4.1, 2.1 Hz, 1H, minor isomer), 2.76 (ddt, *J* = 5.6, 3.5, 2.2 Hz, 1H, major isomer), 2.56 – 2.42 (m, 1H), 2.42 – 2.26 (m, 2H), 2.17 – 1.91 (m, 8H), 1.90 – 1.56 (m, 20H), 1.57 – 1.39 (m, 2H), 1.36 – 1.20 (m, 1H), 1.01 – 0.76 (m, 30H), 0.17 – -0.08 (m, 18H). ^{13}C NMR (101 MHz, CD_2Cl_2) δ 171.1, 171.0, 143.8, 142.7, 137.7, 137.6, 137.6, 137.5, 134.5, 134.3, 134.1, 134.1, 133.9, 133.7, 133.5, 132.3, 132.1, 129.6, 129.5, 129.2, 129.2, 128.5, 128.4, 126.1, 123.8, 123.8, 119.5, 118.4, 95.3, 83.0, 78.1, 78.0, 77.9, 75.7, 72.9, 69.7, 69.5, 68.2, 68.1, 67.5, 67.3, 65.5, 64.0, 63.0, 62.8, 60.4, 55.9, 55.0, 41.8, 38.7, 38.6, 38.1, 38.0, 37.1, 35.8, 35.6, 34.7, 33.3, 32.3, 32.1, 31.7, 31.5, 30.1, 27.9, 26.0, 26.0, 26.0, 22.9, 21.5, 21.4, 18.7, 18.6, 18.5, 18.4, 17.7, 17.7, 12.7, 12.7, –4.2, –4.3, –4.3, –4.6, –4.8, –4.9, –5.2. IR (film) ṽ 2954, 2928, 2856, 1736, 1463, 1376, 1306, 1252, 1201, 1147, 1086, 966, 834, 775 cm^{-1}. HRMS (ESI$^+$) calcd. for $C_{65}H_{108}O_{12}SSi_3Na$ $[M+Na]^+$: 1219.67615; found: 1219.67609.

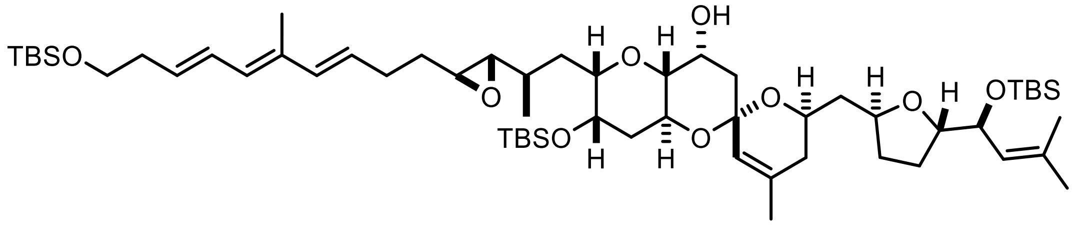

Compound S26. A solution of lithium triethylborohydride (1 M in THF, 46 µL, 46 µmol) in THF (100 µL) was added dropwise to a solution of sulfone **S25** (22.1 mg, 18.4 µmol) in THF (3.5 mL) at –80 °C. After 20 min, the mixture was warmed to –20 °C and stirring was continued for 60 min. $PdCl_2$(dppp) (2.2 mg, 3.7 µmol) and a second portion of lithium triethylborohydride (1 M in THF, 33 µL, 33 µmol) in THF (80 µL) were added and stirring was continued for 10 min, before the mixture was warmed to –10 °C. After 60 min, the reaction was carefully quenched with H_2O (10 mL). After reaching ambient temperature, the mixture was extracted with *tert*-butyl methyl ether (3 × 15 mL). The combined organic layers were washed with brine and dried over Na_2SO_4, the solvent was removed under reduced pressure, and the residue was purified by flash chromatography (silica, hexanes/*tert*-butyl methyl ether 4:1 + 1% NEt_3) to provide the title compound as a colorless oil (10.4 mg, 56%). $[\alpha]_{20}^{D}$ = –15.8

(c = 0.50, CH_2Cl_2). ^{1}H NMR (400 MHz, C_6D_6) δ 6.50 (dd, *J* = 14.4, 10.4 Hz, 1H), 6.21 (d, *J* = 15.6 Hz, 1H), 6.09 (d, *J* = 11.2 Hz, 1H), 5.82 – 5.58 (m, 2H), 5.37 – 5.25 (m, 2H), 4.67 – 4.53 (m, 2H), 4.39 (ddd, *J* = 9.1, 5.7, 1.1 Hz, 2H), 4.18 (dd, *J* = 9.0, 3.2 Hz, 1H), 4.12 (q, *J* = 7.2 Hz, 1H), 4.03 – 3.90 (m, 1H), 3.74 (dd, *J* = 9.1, 1.0 Hz, 1H), 3.58 (td, *J* = 6.6, 1.0 Hz, 2H), 3.53 (s, 1H), 3.44 (dd, *J* = 9.6, 3.4 Hz, 1H), 3.07 – 2.98 (m, 1H), 2.88 (td, *J* = 4.5, 2.1 Hz, 1H), 2.56 – 2.47 (m, 1H), 2.39 – 2.13 (m, 6H), 2.04 – 1.52 (m, 11H), 1.81 (s, 3H), 1.60 (s, 6H), 1.57 (s, 3H), 1.44 – 1.21 (m, 3H), 1.10 (d, *J* = 6.6 Hz, 3H), 1.08 (s, 9H), 1.05 (s, 9H), 0.99 (s, 9H), 0.18 (s, 3H), 0.17 (s, 3H), 0.16 (s, 3H), 0.09 (s, 3H), 0.07 (s, 3H), 0.06 (s, 3H). ^{13}C NMR (101 MHz, C_6D_6) δ 136.1, 136.0, 133.5, 133.4, 131.4, 130.3, 129.2, 128.3, 126.2, 124.3, 97.0, 83.1, 80.7, 78.3, 75.7, 72.8, 70.4, 66.9, 66.2, 63.2, 63.0, 60.3, 57.4, 41.8, 41.4, 38.6, 37.2, 36.2, 34.6, 33.5, 33.0, 32.5, 30.2, 29.9, 27.7, 26.3, 26.2, 26.2, 26.0, 22.9, 18.7, 18.6, 18.5, 18.1, 12.9, –4.0, –4.2, –4.4, –4.6, –5.1. IR (film) ṽ 3531, 2954, 2927, 2856, 1462, 1386, 1253, 1102, 965, 834, 775 cm^{-1}. HRMS (ESI$^+$) calcd. for $C_{57}H_{102}O_9Si_3Na$ $[M+Na]^+$: 1037.67239; found: 1037.67267.

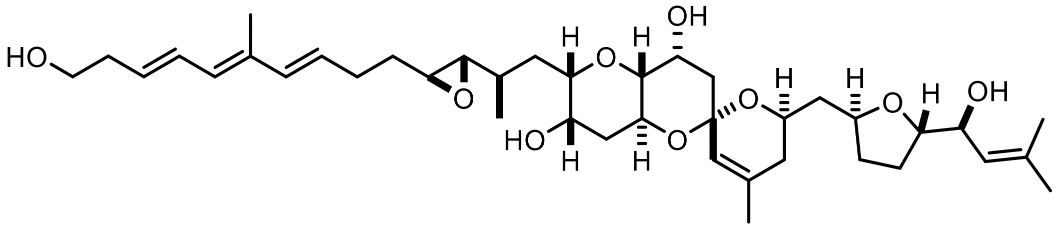

Nominal Prorocentin (1). A solution of HF·pyridine (20 µL) and pyridine (60 µL, 742 µmol) in THF (320 µL) was added dropwise to a solution of silyl ether **S27** (10 mg, 9.85 µmol) in THF (600 µL). The mixture was stirred at ambient temperature for 60 h before sat. aq. $NaHCO_3$ (15 mL) was added. The mixture was extracted with EtOAc (5 × 15 mL), the combined organic layers were washed with brine and dried over Na_2SO_4. The solvent was removed under reduced pressure and the residue was purified by flash chromatography (silica, hexanes/*tert*-butyl methyl ether 1:3 → 0:1 + 1% NEt_3) to provide the title compound as colorless oil (0.5 mg) and a mixture of partially TBS-protected compounds.

The mixture of partly TBS-protected compounds was dissolved in THF (600 µL). To this solution was added a solution of HF·pyridine (20 µL) and pyridine (60 µL, 742 µmol) in THF (320 µL). After stirring for 60 h at rt, the reaction was quenched with sat. aq. $NaHCO_3$ (15 mL). The mixture was extracted with EtOAc (5 × 15 mL), the combined organic layers were washed with brine and dried over Na_2SO_4. The solvent was removed under reduced pressure and the residue was purified by flash chromatography (silica, hexanes/*tert*-butyl methyl ether 1:3 → 0:1 + 1% NEt_3) to provide the title compound as a colorless oil (0.9 mg). This deprotection process of the recovered mixture of partly TBS-cleaved material was repeated twice to provide an additional crop of the title compound (2.0 mg). The combined product (3.4 mg, 51%) was further purified with preparative HPLC (YMC Triart C18 column, 50 mm x 10.0 mm i.D., Methanol/H_2O = 72:28, 4.7 mL/min) to give the title compound in analytically

pure form as a colorless oil (1.3 mg, 20%). $[\alpha]_{25}^{D}$ = −7.0 (c = 0.20, MeOH). ^{1}H NMR (600 MHz, $CDCl_3$) δ 6.46 (ddt, *J* = 15.0, 11.1, 1.4 Hz, 1H), 6.12 (d, *J* = 15.5 Hz, 1H), 5.98 (d, *J* = 11.1 Hz, 1H), 5.68 (dt, *J* = 15.6, 7.0 Hz, 1H), 5.68 (dt, *J* = 14.9, 7.3 Hz, 1H), 5.42 (d, *J* = 10.1 Hz, 1H), 5.31 (tt, *J* = 2.6, 1.4 Hz, 1H), 5.12 (d, *J* = 8.0 Hz, 1H), 5.01 (dp, *J* = 9.0, 1.4 Hz, 1H), 4.67 (s (br), 1H), 4.37 (ddd, *J* = 11.8, 9.9, 4.8 Hz, 1H), 4.16 (t, *J* = 9.0 Hz, 1H), 4.14 – 3.97 (m, 4H), 3.73 (s(br), 1H), 3.68 (t, *J* = 6.2 Hz, 2H), 3.54 (ddd, *J* = 8.9, 4.3, 1.4 Hz, 1H), 3.04 (dd, *J* = 10.0, 2.5 Hz, 1H), 2.75 (ddd, *J* = 6.4, 5.0, 2.3 Hz, 1H), 2.49 (dd, *J* = 7.4, 2.3 Hz, 1H), 2.41 (qd, *J* = 6.5, 1.3 Hz, 2H), 2.34 – 2.19 (m, 3H), 2.08 (dd, *J* = 14.3, 3.5 Hz, 1H), 2.06 – 2.00 (m, 1H), 1.98 – 1.87 (m, 2H), 1.83 (d, *J* = 1.2 Hz, 3H), 1.83 – 1.73 (m, 5H), 1.73 (d, *J* = 1.4 Hz, 3H), 1.71 (d, *J* = 1.2 Hz, 3H), 1.71 (d, *J* = 1.5 Hz, 3H), 1.67 – 1.59 (m, 3H), 1.55 (s(br), 1H), 1.54 – 1.39 (m, 4H), 1.02 (d, *J* = 6.3 Hz, 3H). ^{13}C NMR (151 MHz, $CDCl_3$) δ 136.9, 136.9, 135.4, 133.8, 130.0, 129.8, 129.1, 128.3, 123.7, 122.4, 96.2, 83.4, 80.2, 78.7, 78.4, 70.9, 68.8, 67.7, 67.1, 63.5, 62.0, 60.9, 58.1, 41.1, 40.2, 37.8, 36.5, 35.4, 34.9, 33.5, 32.5, 32.3, 29.4, 27.5, 26.0, 22.9, 18.7, 17.5, 12.8. IR (film) ṽ 3435, 2960, 2921, 2852, 1445, 1260, 1095, 1018, 966, 797 cm^{-1}. HRMS (ESI^{+}) calcd. for $C_{39}H_{60}O_9Na$ [M+Na]$^{+}$: 695.41295; found: 695.41316.

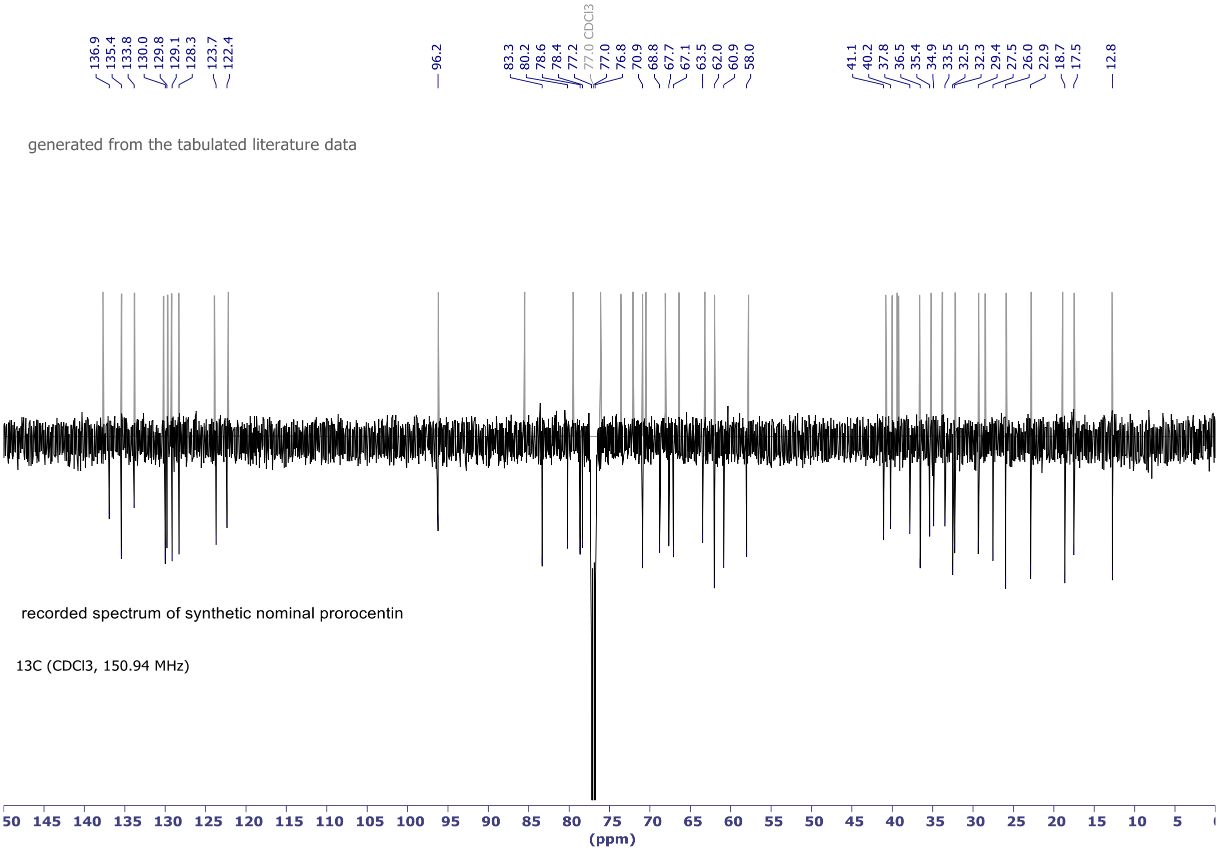

Figure S6. Visual comparison of the ^{13}C NMR data of authentic prorocentin (up) with those of synthetic nominal prorocentin (**1**, down); for the sake of comparison, the spectrum was referenced to $CDCl_3$: δ_C = 77.00 pp as used in the isolation paper.[1] Note that the shown spectrum (up) was generated (MestReNova) by converting the tabulated ^{13}C NMR data of the natural product into a formal spectrum;

the intensity of the lines is arbitrarily set to be identical for all signals; for a tabular survey of the exact numbers, see Table S9.

Table S9. NMR data of synthetic nominal prorocentin (**1**); numbering scheme as shown in the insert.

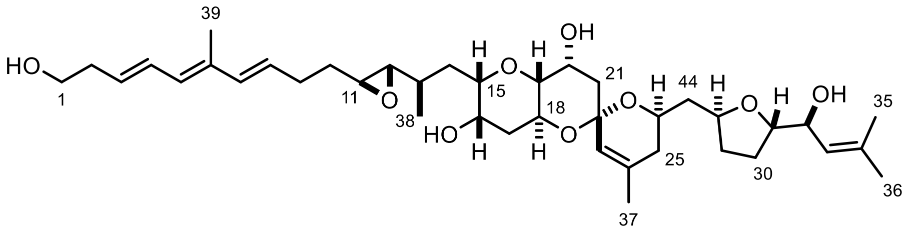

Atom number	δ [ppm]	COSY	J [Hz]	HSQC	HMBC	NOESY/EXSY
1 C	62.04			1	2, 3	
H2	3.68	2	6.23, 6.23	1	2, 3	1-OH, 2, 3
1-OH O						
H	1.55					1, H2O,16-OH, 18a, 20-OH, 31a, 32, 32-OH
2 C	36.55			2	1, 3, 4	
H2	2.41	1, 3, 4	6.53, 6.53, 6.53, 1.25	2	1, 3, 4	1, 3, 4
3 C	129.98			3	1, 2, 5	
H	5.68	2, 4	15.00(4), 7.35, 7.35	3	1, 2, 5	1, 2, 5
4 C	129.79			4	2, 39	
H	6.46	2, 3, 5	11.11(5), 15.00(3), 1.38, 1.38	4	2	2, 39
5 C	129.14			5	3, 7, 39	
H	5.98	4, 39	11.11(4)	5	3, 7, 39	3, 7
6 C	133.84				7, 8, 39	
7 C	135.41			7	5, 9, 39	
H	6.12	8, 9	15.53(8)	7	5, 6, 9, 39	5, 9
8 C	128.29			8	9, 10	
H	5.68	7, 9	15.53(7), 6.97, 6.97	8	6, 9, 10	39
9 C	29.36			9	7, 8, 10	
H2	2.26	7, 8, 10		9	7, 8, 10, 11	7, 11
10 C	32.29			10	8, 9, 11	
H2	1.63	9, 11		10	8, 9, 11, 12	11, 12
11 C	58.05			11	9, 10	
H	2.75	10, 12	6.39, 5.00, 2.25	11	10	9, 10, 12, 13, 38
12 C	63.48			12	10, 13, 38	
H	2.49	11, 13	7.39, 2.25	12	13	10, 11, 14b, 15a, 38
13 C	32.54			13	12, 14a, 14b, 15a, 38	

H	1.54	12, 38		13	12, 14	11, 15a, 16a, 38
14 C	34.91			14a, 14b	13, 15a, 38	
Ha	1.77	14b, 15a, 38	9.45, 9.45, 9.45, 7.70	14	13, 15, 16	14b, 38
Hb	1.53	14a, 15a		14	13	12, 14a, 38
15 C	78.37			15a	14a	
15a H	3.54	14a, 14b	8.93, 4.25, 1.39	15	13, 14, 16	12, 13, 16a, 17b, 19a, 38
16 C	68.81			16a	14a, 15a, 17a	
16-OH						
O						
H	5.12	16a	7.96(16a)			H2O, 1-OH, 20-OH, 32-OH
16a H	3.73	16-OH, 17a, 17b	7.96(16-OH)	16		13, 15a, 17a, 17b
17 C	37.83			17a, 17b		
Ha	2.31	16a, 17b, 18a	12.39, 4.13, 4.13	17	16, 18	16a, 17b, 18a
Hb	1.62	16a, 17a, 18a	12.37, 12.34, 2.91	17	18	15a, 16a, 17a, 19a
18 C	60.86			18a	17a, 17b	
18a H	4.37	17a, 17b, 19a	11.84, 9.90, 4.80	18	20	1-OH, 17a, 20-OH
19 C	80.19			19a	21a	
19a H	3.04	18a, 20	9.96, 2.51	19		15a, 17b, 20, 21b
20 C	67.11			20	18a, 20-OH, 21a	
H	4.03	19a, 20-OH, 21a, 21b	9.86, 3.00, 3.00, 3.00	20		19a
20-OH						
O						
H	5.42	20	10.06		20, 21	H2O,1-OH, 16-OH, 18a, 21a, 32-OH
21 C	40.24			21a, 21b	20-OH	
Ha	2.08	20, 21b	14.31, 3.51	21	19, 20, 22	20-OH
Hb	1.80	20, 21a	14.18, 2.94	21	22, 23	19a, 23
22 C	96.24				21a, 21b, 23	
23 C	122.36			23	21b, 25b, 37	
H	5.31	25a, 37	2.61, 2.61, 1.43, 1.43	23	22, 25, 37	21b, 37
24 C	136.91				25a, 25b, 37	
25 C	35.39			25a, 25b	23, 27b, 37	
Ha	1.94	23, 25b, 26a, 37		25	24, 26	

Hb	1.81	25a, 26a		25	23, 24, 37	26a
26 C	67.65			26a	25a, 27a, 27b, 28a	
26a H	4.08	25a, 25b, 27b		26		25b
27 C	41.10			27a, 27b		
Ha	1.81	27b		27	26, 28	
Hb	1.75	26a, 27a, 28a		27	25, 26, 28	
28 C	78.65			28a	27a, 27b, 31a	
28a H	4.11	27b, 29a, 29b		28	26	29a
29 C	33.48			29a		
Ha	2.04	28a, 29b, 30b	10.68, 7.27, 5.19, 2.42	29		28a, 29b, 30b
Hb	1.50	28a, 29a, 30a	10.97, 9.09, 9.09, 8.47			29a, 30a
30 C	27.54			30a, 30b		
Ha	1.90	29b, 30b, 31a		30		29b, 30b, 31a
Hb	1.44	29a, 30a, 31a		30	31, 32	29a, 30a, 32
31 C	83.37			31a	30b, 32, 35, 36	
31a H	4.00	30a, 30b, 32	9.27, 6.60, 6.60	31	28	1-OH, 30a, 33
32 C	70.91			32	30b	
H	4.16	31a, 33	9.00, 9.00	32	31, 33, 34	1-OH, 30b, 35
32-OH O						
H	4.67					H2O,1-OH, 16-OH, 20-OH
33 C	123.71			33	32, 35, 36	
H	5.01	32, 35, 36	8.99, 1.39, 1.39, 1.36, 1.36	33	35, 36	31a, 36
34 C	136.89				32, 35, 36	
35 C	18.65			35	33, 36	
H3	1.73	33	1.36	35	31, 33, 34, 36	32
36 C	25.99			36	33, 35	
H3	1.71	33		36	31, 33, 34, 35	33
37 C	22.89			37	23, 25b	
H3	1.71	23, 25a	1.23	37	23, 24, 25	23
38 C	17.54			38		
H3	1.02	13, 14a	6.28	38	12, 13, 14	11, 12, 13, 14a, 14b, 15a
39 C	12.75			39	5, 7	
H3	1.83	5	1.20	39	4, 5, 6, 7	4, 8

Table S10. Comparison of ^{13}C NMR data of authentic prorocentin (δ (lit.))[1] and synthetic **1**; for the sake of comparison, the spectrum was referenced to $CDCl_3$: δ_C = 77.00 pp as used in the isolation paper. Numbering scheme as shown in the insert. Shift differences $\Delta\delta \geq 1$ ppm are color coded in red.

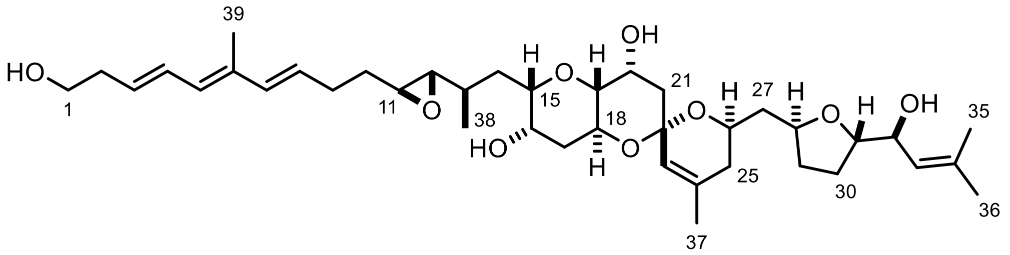

Atom number	δ (lit.) [ppm]	δ (**1**) [ppm]	Δδ (δ(lit.) – δ(**1**))
1	62.0	62.0	±0.0
2	36.6	36.5	+0.1
3	130.2	130.0	+0.2
4	129.7	129.8	–0.1
5	129.2	129.1	+0.1
6	133.8	133.8	±0.0
7	135.4	135.4	±0.0
8	128.3	128.3	±0.0
9	29.3	29.4	–0.1
10	32.2	32.3	–0.1
11	57.8	58.1	–0.3
12	63.2	63.5	–0.3
13	32.2	32.5	–0.3
14	39.2	34.9	+4.3
15	73.6	78.4	–4.8
16	70.5	68.8	+1.7
17	39.4	37.8	+1.6
18	70.9	60.9	+10.0
19	76.1	80.2	–4.1
20	66.4	67.1	–0.7
21	40.0	40.2	–0.2
22	96.2	96.2	±0.0
23	122.2	122.4	–0.2
24	137.7	136.9	+0.8
25	35.2	35.4	–0.2
26	68.1	67.7	+0.4
27	40.8	41.1	–0.3
28	79.5	78.7	+0.8
29	33.8	33.5	+0.3
30	28.5	27.5	+1.0
31	85.5	83.4	+2.1
32	72.1	70.9	+1.2
33	123.9	123.7	+0.2
34	135.4	136.9	–1.5
35	18.9	18.7	+0.2
36	25.9	26.0	–0.1
37	22.8	22.9	–0.1
38	17.5	17.5	±0.0
39	12.8	12.8	±0.0

ACTUAL PROROCENTIN

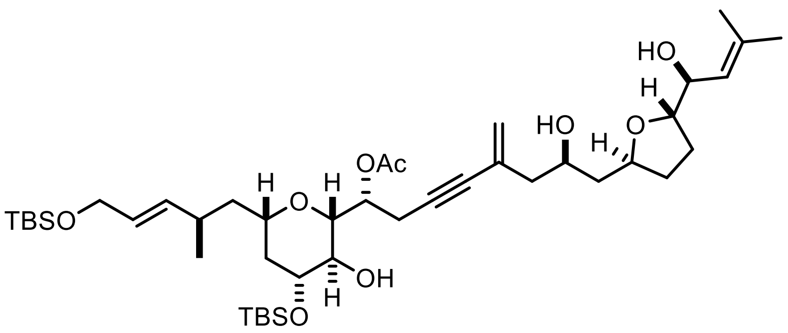

Enyne 59. Degassed diisopropylamine (1.1 mL) was added to a mixture of alkenyl iodide **18** (202 mg, 550 µmol), alkyne **56** (200 mg, 360 µmol), CuI (14.0 mg, 73.5 µmol), PPh_3 (19.1 mg, 72.8 µmol) and $Pd_2(dba)_3$ (16.7 mg, 18.2 µmol). The resulting mixture was stirred overnight before the reaction was quenched with sat. aq. NH_4Cl (20 mL). The mixture was extracted with *tert*-butyl methyl ether (3 × 20 mL), the combined organic layers were washed with water (20 mL) and brine (20 mL) and were dried over $MgSO_4$. The solvent was removed under reduced pressure and the residue was purified by flash chromatography (silica, hexanes/*tert*-butyl methyl ether 1:1) to provide the title compound as a colorless oil (225 mg, 91%). $[\alpha]_D^{20}$ = −2.8 (c = 0.70, CH_2Cl_2). ^1H NMR (400 MHz, CD_2Cl_2) δ 5.53 (dt, *J* = 15.4, 4.9 Hz, 1H), 5.41 (ddt, *J* = 15.2, 8.4, 1.4 Hz, 1H), 5.35 – 5.30 (m, 1H), 5.29 (d, *J* = 2.1 Hz, 1H), 5.22 (dd, *J* = 2.2, 1.1 Hz, 1H), 5.08 (dp, *J* = 9.0, 1.4 Hz, 1H), 4.22 – 4.16 (m, 1H), 4.14 (dd, *J* = 9.1, 7.2 Hz, 1H), 4.10 (dd, *J* = 5.1, 1.5 Hz, 2H), 4.04 (dtd, *J* = 7.3, 5.6, 3.5 Hz, 1H), 3.88 (q, *J* = 7.0 Hz, 1H), 3.55 (ddd, *J* = 11.1, 8.0, 5.0 Hz, 1H), 3.41 – 3.31 (m, 3H), 3.27 (dd, *J* = 9.8, 2.8 Hz, 1H), 2.84 (d, *J* = 3.0 Hz, 1H, OH), 2.77 (dd, *J* = 17.2, 5.7 Hz, 1H), 2.66 (dd, *J* = 17.2, 7.6 Hz, 1H), 2.51 – 2.37 (m, 2H), 2.28 (dd, *J* = 13.6, 7.4 Hz, 1H), 2.21 (dd, *J* = 13.4, 5.6 Hz, 1H), 2.12 – 2.03 (m, 3H), 1.93 – 1.85 (m, 1H), 1.77 (ddd, *J* = 12.8, 5.1, 1.7 Hz, 1H), 1.73 (d, *J* = 1.4 Hz, 3H), 1.71 (d, *J* = 1.4 Hz, 3H), 1.70 – 1.65 (m, 1H), 1.59 – 1.48 (m, 4H), 1.34 (dt, *J* = 13.1, 11.4 Hz, 1H), 1.25 (ddd, *J* = 13.8, 10.0, 3.2 Hz, 1H), 0.98 (d, *J* = 6.8 Hz, 3H), 0.89 (m, 18H), 0.09 (s, 3H), 0.08 (s, 3H), 0.05 (s, 6H). ^{13}C NMR (101 MHz, CD_2Cl_2) δ 170.5, 137.5, 136.1, 129.1 (2x C), 124.0, 122.7, 87.2, 83.4, 82.2, 79.6, 79.2, 75.1, 73.8 (2x C), 71.9, 71.1, 69.9, 64.1, 45.7, 42.9, 42.1, 40.8, 33.5, 33.0, 27.7, 26.1, 26.0, 25.9, 21.3, 20.1, 18.8, 18.6, 18.3, −4.3, −4.4, −5.0, −5.0. IR (film) ṽ 3434, 2928, 2952, 2856, 1740, 1611, 1442, 1462, 1375, 1250, 1092, 1031, 971, 940, 883, 836, 777, 671 cm^{-1}. HRMS ($ESI^{+/-}$) calcd. for $C_{43}H_{76}O_9Si_2Na$ $[M+Na]^+$: 815.49201; found: 815.49213.

Spiroketal 62. PPTS (2.2 mg, 9.0 µmol) and $(JohnPhos)Au(MeCN)SbF_6$ (**36**, 6.9 mg, 9.0 µmol) were

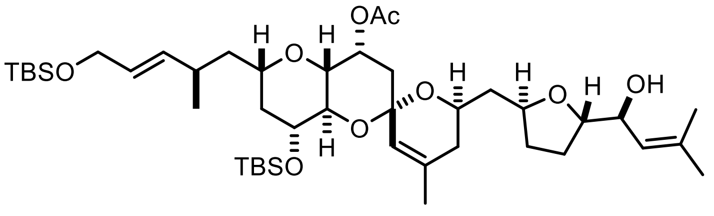

added to a solution of enyne **59** (71.1 mg, 89.6 µmol) in CH_2Cl_2 (2 mL). The mixture was stirred for 40 min before sat. aq. NH_4Cl (10 mL) was introduced. The layers were separated and the aqueous phase was extracted with CH_2Cl_2 (3 × 15 mL). The combined extracts were washed with brine

and dried over Na_2SO_4. The solvent was removed under reduced pressure and the residue was purified by flash chromatography (silica, hexanes/*tert*-butyl methyl ether 7:3 → 2:1) to provide the title compound as a colorless oil (46 mg, 69%). $[\alpha]_D^{20}$ = −21.5 (c = 0.33, $CHCl_3$). ^{1}H NMR (600 MHz, CD_2Cl_2) δ 5.51 (dtd, *J* = 15.3, 5.1, 0.6 Hz, 1H), 5.43 (ddt, *J* = 15.3, 8.1, 1.3 Hz, 1H), 5.25 – 5.18 (m, 1H), 5.08 (dhept., *J* = 9.0, 1.4 Hz, 1H), 5.06 (q, *J* = 3.0 Hz, 1H), 4.29 (tt, *J* = 7.9, 5.3 Hz, 1H), 4.16 – 4.08 (m, 3H), 3.99 (ddt, *J* = 11.1, 9.7, 3.3 Hz, 1H), 3.86 – 3.80 (m, 1H), 3.73 (dd, *J* = 9.7, 8.9 Hz, 1H), 3.65 (ddd, *J* = 10.9, 8.8, 5.1 Hz, 1H), 3.42 (dddd, *J* = 11.1, 9.0, 3.6, 1.8 Hz, 1H), 3.10 (dd, *J* = 9.7, 3.2 Hz, 1H), 2.53 (s, 1H), 2.41 – 2.33 (m, 1H), 2.33 – 2.26 (m, 1H), 2.14 (dd, *J* = 15.0, 3.0 Hz, 1H), 2.07 (s, 3H), 1.95 – 1.86 (m, 3H), 1.79 (ddd, *J* = 13.0, 5.1, 1.9 Hz, 1H), 1.77 – 1.70 (m, 8H), 1.70 – 1.65 (m, 3H), 1.58 – 1.47 (m, 4H), 1.39 (dt, *J* = 13.1, 11.2 Hz, 1H), 1.25 (ddd, *J* = 14.0, 9.7, 3.6 Hz, 1H), 0.97 (d, *J* = 6.8 Hz, 3H), 0.90 (s, 9H), 0.87 (s, 9H), 0.06 (d, *J* = 0.5 Hz, 6H), 0.04 (d, *J* = 0.4 Hz, 3H), 0.03 (d, *J* = 0.4 Hz, 3H). ^{13}C NMR (151 MHz, CD_2Cl_2) δ 171.2, 137.4, 136.9, 136.4, 129.0, 124.1, 123.7, 95.0, 83.0, 77.0, 75.2, 74.3, 72.2, 71.4, 70.6, 68.6, 65.7, 64.2, 43.0, 41.7, 41.6, 38.4, 35.8, 33.3, 33.0, 28.1, 26.1, 26.1, 26.0, 22.8, 21.6, 21.3, 18.9, 18.7, 18.5, −4.2, −4.9, −5.0. IR (film) ṽ 3446, 2927, 2953, 2855, 1738, 1681, 1443, 1462, 1379, 1249, 1202, 1175, 1154, 1107, 1071, 960, 835, 815, 776, 667, 605, 424 cm^{-1}. HRMS (ESI$^{+/-}$) calcd. for $C_{43}H_{76}O_9Si_2Na$ $[M+Na]^+$: 815.49274; found: 815.49213.

Compound S27. TBSOTf (70 µL, 305 µmol) was added dropwise to a solution of compound **62** (145 mg, 183 µmol) and 2,6-lutidine (70 µL, 601 µmol) in CH_2Cl_2 (5 mL) at −78 °C. The resulting mixture was stirred for 2 h before sat. aq. NH_4Cl (30 mL) was introduced. The mixture was extracted

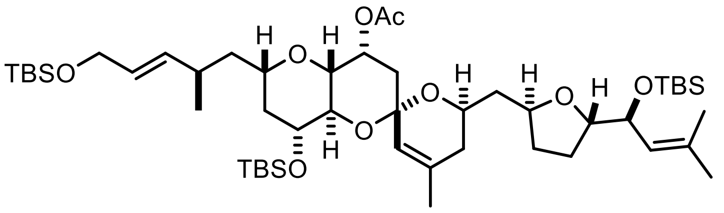

with CH_2Cl_2 (3 × 30 mL), the combined extracts were washed with brine and dried over $MgSO_4$. The solvent was removed under reduced pressure and the residue was purified by flash chromatography (silica, hexanes/*tert*-butyl methyl ether 9:1) to give the title compound as a colorless oil (152 mg, 92%). $[\alpha]_D^{20}$ = −36.4 (c = 0.33, CH_2Cl_2). ^{1}H NMR (400 MHz, CD_2Cl_2) δ 5.55 – 5.40 (m, 2H), 5.20 (p, *J* = 1.3 Hz, 1H), 5.14 (dp, *J* = 9.1, 1.4 Hz, 1H), 5.05 (q, *J* = 3.1 Hz, 1H), 4.32 – 4.22 (m, 2H), 4.11 (d, *J* = 5.2 Hz, 2H), 3.97 (ddt, *J* = 11.0, 9.1, 3.7 Hz, 1H), 3.87 (ddd, *J* = 7.9, 6.7, 5.7 Hz, 1H), 3.71 – 3.60 (m, 2H), 3.42 (dddd, *J* = 11.2, 9.2, 3.7, 1.9 Hz, 1H), 3.08 (dd, *J* = 9.3, 3.1 Hz, 1H), 2.43 – 2.31 (m, 1H), 2.29 – 2.21 (m, 1H), 2.14 (dd, *J* = 15.0, 3.0 Hz, 1H), 2.05 (s, 3H), 1.96 – 1.86 (m, 2H), 1.86 – 1.77 (m, 2H), 1.76 – 1.69 (m, 5H), 1.69 – 1.67 (m, 3H), 1.67 – 1.63 (m, 4H), 1.56 – 1.45 (m, 3H), 1.39 (td, *J* = 11.0, 2.1 Hz, 1H), 1.30 – 1.22 (m, 1H), 0.97 (d, *J* = 6.7 Hz, 3H), 0.90 (s, 9H), 0.87 (d, *J* = 1.0 Hz, 18H), 0.06 (s, 6H), 0.04 (s, 6H), 0.03 (s, 3H), 0.01 (s, 3H). ^{13}C NMR (101 MHz, CD_2Cl_2) δ 171.2, 136.9, 136.4, 133.4, 129.0, 126.3, 123.6, 94.9, 83.0, 76.7, 75.3, 74.4, 73.0, 72.3, 70.5, 68.6, 65.2, 64.2, 43.0, 42.2, 41.7, 38.4, 35.6, 33.3, 32.8, 28.1, 26.1, 26.1, 26.1, 22.8, 21.8, 21.4, 18.8, 18.7, 18.6, 18.5, −4.2, −4.2, −4.4, −4.9, −5.0. IR (film) ṽ 2928, 2953, 2856,

2885, 1738, 1680, 1462, 1472, 1442, 1360, 1379, 1295, 1248, 1201, 1108, 1067, 1005, 958, 832, 774, 666, 604, 575, 516, 476, 424 cm^{-1}. HRMS ($ESI^{+/-}$) calcd. for $C_{49}H_{90}O_9Si_3Na$ $[M+Na]^+$: 929.57849; found: 929.57894.

Compound S28. HF·pyridine (180 µL) was added dropwise to a solution of silyl ether **S27** (152 mg, 168 µmol) in pyridine (4.6 mL) at 0 °C. The mixture was stirred at ambient temperature overnight. For work up, the solution was cooled to 0 °C before sat. aq. $NaHCO_3$ (15 mL) was added dropwise. After reaching ambient temperature, the mixture was extracted with *tert*-butyl methyl ether (3 × 15 mL). The combined organic layers were washed with brine and dried over Na_2SO_4. The solvent was removed under reduced pressure and the residue was purified by flash chromatography (silica, hexanes/*tert*-butyl methyl ether 2:1 → 1:1) to provide the title compound as a colorless oil (122 mg, 92%). $[\alpha]_D^{20}$ = −34.3 (c = 0.33, CH_2Cl_2). ^1H NMR (400 MHz, CD_2Cl_2) δ 5.60 (dt, *J* = 15.4, 5.4 Hz, 1H), 5.49 (ddt, *J* = 15.3, 7.9, 1.2 Hz, 1H), 5.22 – 5.19 (m, 1H), 5.14 (dp, *J* = 9.1, 1.4 Hz, 1H), 5.07 (q, *J* = 3.1 Hz, 1H), 4.33 – 4.21 (m, 2H), 4.05 (dd, *J* = 5.5, 1.1 Hz, 2H), 3.97 (ddt, *J* = 11.0, 9.0, 3.7 Hz, 1H), 3.87 (ddd, *J* = 7.9, 6.8, 5.7 Hz, 1H), 3.70 – 3.60 (m, 2H), 3.42 (tdd, *J* = 9.0, 3.7, 2.0 Hz, 1H), 3.09 (dd, *J* = 9.3, 3.1 Hz, 1H), 2.44 – 2.35 (m, 1H), 2.25 (dddd, *J* = 11.3, 7.5, 5.5, 3.1 Hz, 1H), 2.12 (dd, *J* = 15.0, 3.0 Hz, 1H), 2.05 (s, 3H), 1.94 – 1.83 (m, 3H), 1.82 – 1.74 (m, 3H), 1.71 (d, *J* = 1.4 Hz, 3H), 1.68 – 1.64 (m, 7H), 1.56 – 1.46 (m, 3H), 1.44 – 1.31 (m, 2H), 0.98 (d, *J* = 6.8 Hz, 3H), 0.87 (d, *J* = 0.8 Hz, 18H), 0.05 – 0.02 (m, 9H), 0.01 (s, 3H). ^{13}C NMR (101 MHz, CD_2Cl_2) δ 171.3, 137.8, 137.0, 133.4, 128.7, 126.3, 123.6, 94.9, 83.0, 76.6, 75.2, 74.2, 73.0, 72.2, 70.5, 68.5, 65.2, 63.8, 42.9, 42.2, 41.7, 38.4, 35.6, 33.3, 32.8, 28.1, 26.1, 26.1, 22.8, 21.8, 21.3, 18.8, 18.6, 18.5, −4.2, −4.2, −4.5, −4.9. IR (film) ṽ 3461, 2952, 2927, 2855, 1737, 1680, 1462, 1442, 1380, 1248, 1201, 1149, 1108, 1069, 1005, 958, 870, 834, 776, 714, 666, 606, 577, 493, 422 cm^{-1}. HRMS ($ESI^{+/-}$) calcd. for $C_{43}H_{76}O_9Si_2Na$ $[M+Na]^+$: 815.49201; found: 815.49147.

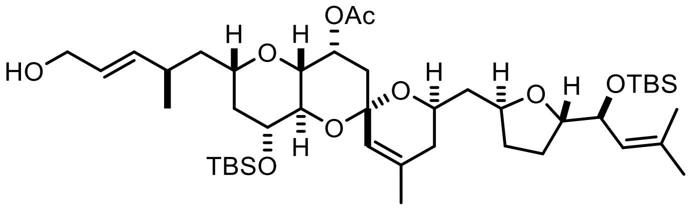

Epoxide S29. A solution of (+)-diisopropyl L-tartrate (2.1 µL, 10 µmol) in CH_2Cl_2 (48 µL) was added to a suspension of powdered molecular sieves 4Å (10 mg) in CH_2Cl_2 (300 µL) at −25 °C. A solution of $Ti(O\textit{i}\text{-}Pr)_4$ (2.2 µL, 7.6 µmol) in CH_2Cl_2 (72 µL) was then added and stirring was continued for 40 min. Cumene hydroperoxide (80% w/w, 30 µL, 162 µmol) was introduced and stirring was continued for another 30 min before a solution of allylic alcohol **S28** (40.0 mg, 50.4 µmol) in CH_2Cl_2 (800 µL) was added. The resulting mixture was stirred at this temperature overnight. A solution of NaOH (75 mg) in brine

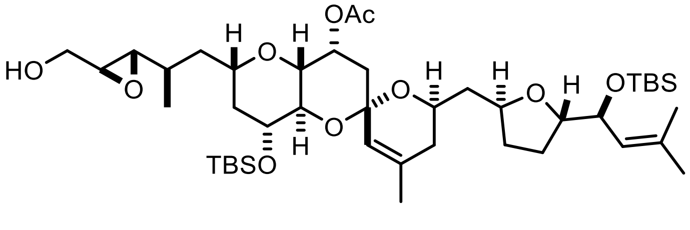

(0.75 mL) was introduced, the mixture was warmed to 0 °C and stirring was continued for 1 h. The suspension was filtered through a short pad of Celite, which was carefully rinsed with CH_2Cl_2 (20 mL). The combined filtrates were evaporated and the residue was purified by flash chromatography (silica, hexanes/*tert*-butyl methyl ether 11:10) to provide the title compound as a colorless oil (34.4 mg, dr > 20:1, 84%). $[\alpha]_{20}^{D}$ = –26.7 (c = 1.00, CH_2Cl_2). ^{1}H NMR (400 MHz, CD_2Cl_2) δ 5.20 (p, *J* = 1.2 Hz, 1H), 5.13 (dp, *J* = 9.2, 1.4 Hz, 1H), 5.06 (q, *J* = 2.6 Hz, 1H), 4.25 (ddd, *J* = 14.8, 8.8, 4.4 Hz, 2H), 3.96 (ddt, *J* = 11.0, 8.9, 3.7 Hz, 1H), 3.86 (ddd, *J* = 7.9, 6.7, 5.6 Hz, 1H), 3.79 (dt, *J* = 12.4, 3.7 Hz, 1H), 3.70 – 3.48 (m, 4H), 3.15 – 3.07 (m, 1H), 2.91 (ddd, *J* = 4.3, 3.0, 2.3 Hz, 1H), 2.70 (dd, *J* = 7.5, 2.3 Hz, 1H), 2.28 – 2.19 (m, 1H), 2.11 (dd, *J* = 15.0, 3.0 Hz, 1H), 2.04 (s, 3H), 1.98 – 1.79 (m, 4H), 1.78 – 1.64 (m, 13H), 1.54 – 1.34 (m, 5H), 1.01 (d, *J* = 6.5 Hz, 3H), 0.86 (d, *J* = 0.8 Hz, 18H), 0.04 (s, 3H), 0.03 (s, 6H), 0.01 (s, 3H). ^{13}C NMR (101 MHz, CD_2Cl_2) δ 171.2, 137.0, 133.4, 126.2, 123.4, 94.8, 82.9, 76.6, 75.3, 73.7, 72.9, 71.9, 70.3, 68.3, 65.1, 62.3, 60.2, 58.5, 42.1, 41.6, 39.7, 38.4, 35.5, 32.7, 32.5, 28.0, 26.1, 26.0, 26.0, 22.8, 21.7, 18.7, 18.6, 18.4, 17.7, –4.3, –4.3, –4.5, –5.0. IR (film) ṽ 2955, 2928, 2888 , 2856, 1738, 1382, 1250, 1203, 1150, 1109, 1071, 1050, 1006, 960, 877, 835, 777 cm^{-1}. HRMS (ESI$^{+/-}$) calcd. for $C_{43}H_{76}O_{10}Si_2Na$ [M+Na]$^+$: 831.48692; found: 831.48652.

Iodide 63. A solution of alcohol **S29** (34.0 mg, 42.0 µmol) in CH_2Cl_2 (1 mL) was added to a mixture of

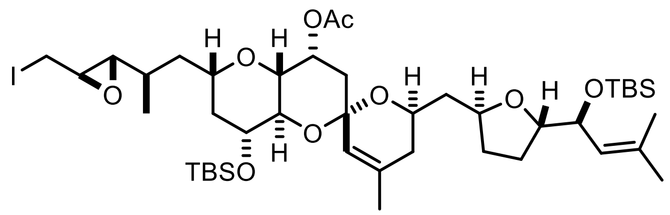

imidazole (10.2 mg, 150 µmol) and PPh_3 (17.4 mg, 66.3 µmol). The resulting solution was cooled to 0 °C before I_2 (16.0 mg, 63.0 µmol) was added in one portion and stirring was continued for 2 h 45 min. *tert*-Butyl methyl ether (20 mL) was introduced and the mixture was successively washed with sat. aq. $Na_2S_2O_3$ (20 mL) and brine (20 mL). The organic layer was evaporated under reduced pressure and the residue was purified by flash chromatography (silica, hexanes/*tert*-butyl methyl ether 5:1 → 4:1) to provide the title compound as a yellow oil (34 mg, 88%). $[\alpha]_{20}^{D}$ = –19.1 (c = 1.00, CH_2Cl_2). ^{1}H NMR (400 MHz, CD_2Cl_2) δ 5.21 (p, *J* = 1.2 Hz, 1H), 5.13 (dp, *J* = 9.1, 1.4 Hz, 1H), 5.07 (q, *J* = 3.1 Hz, 1H), 4.26 (dtd, *J* = 14.7, 8.8, 5.5 Hz, 2H), 3.96 (ddt, *J* = 11.0, 9.1, 3.7 Hz, 1H), 3.86 (ddd, *J* = 7.9, 6.7, 5.6 Hz, 1H), 3.74 – 3.63 (m, 3H), 3.34 – 3.21 (m, 2H), 3.08 – 3.00 (m, 2H), 2.57 (dd, *J* = 7.7, 1.7 Hz, 1H), 2.31 – 2.19 (m, 1H), 2.12 (dd, *J* = 15.0, 3.0 Hz, 1H), 2.04 (s, 3H), 1.97 – 1.79 (m, 4H), 1.76 (t, *J* = 3.2 Hz, 1H), 1.74 – 1.63 (m, 11H), 1.55 – 1.31 (m, 6H), 0.99 (d, *J* = 6.6 Hz, 3H), 0.86 (s, 18H), 0.04 (s, 3H), 0.03 (s, 6H), 0.01 (s, 3H). ^{13}C NMR (101 MHz, CD_2Cl_2) δ 171.1, 137.0, 133.3, 126.2, 123.4, 94.8, 82.9, 76.6, 75.1, 73.5, 72.9, 71.9, 70.4, 68.3, 66.8, 65.1, 58.3, 42.1, 41.7, 39.6, 38.3, 35.5, 32.7, 32.7, 28.0, 26.1, 26.0, 26.0, 22.8, 21.7, 18.8, 18.6, 18.5, 17.8, 5.9, –4.3, –4.3, –4.5, –5.0. IR (film) ṽ 2953, 2928, 2855, 1737, 1249, 1109, 1070, 959, 835, 776 cm^{-1}. HRMS (ESI$^+$) calcd. for $C_{43}H_{75}IO_9Si_2Na$ [M+Na]$^+$: 941.38866; found: 941.38788.

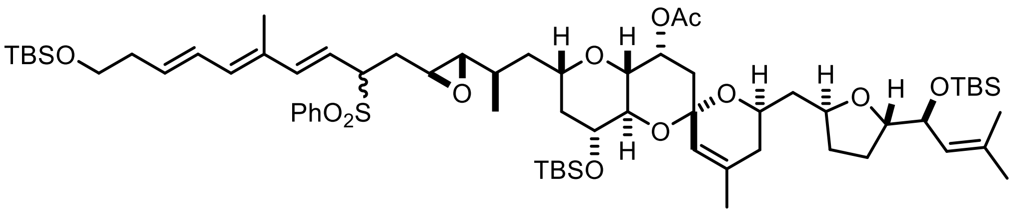

Compound 64. A solution of *n*-BuLi (1.6 M in hexanes, 70 µL, 133 µmol) was added dropwise to a solution of sulfone **8** (54.0 mg, 133 µmol) and DMPU (70 µL, 579 µmol) in THF (3 mL) at –80 °C. Stirring was continued for 60 min at this temperature before a solution of iodide **63** (34.0 mg, 37.0 µmol) in THF (1.3 mL) was added. After 30 min, the mixture was warmed to –60 °C and stirring was continued for 70 min. The reaction was quenched by addition of sat. aq. NH_4Cl (3 mL). After reaching ambient temperature, the mixture was extracted with EtOAc (3 × 15 mL). The combined organic layers were washed with brine and dried over Na_2SO_4, the solvent was removed under reduced pressure, and the residue was purified by flash chromatography (silica, hexanes/*tert*-butyl methyl ether 3:1 + 1% NEt_3) to provide the title compound as an inseparable mixture of diastereoisomers (dr ≈ 3:2, 38.0 mg, 86%,). $[\alpha]_{20}^{D}$ = –13.0 (c = 1.00, CH_2Cl_2). ^{1}H NMR (400 MHz, CD_2Cl_2) δ 7.83 – 7.73 (m, 2H), 7.65 (tq, *J* = 6.9, 1.5 Hz, 1H), 7.54 (t, *J* = 7.6 Hz, 2H), 6.49 – 6.34 (m, 1H), 6.04 – 5.86 (m, 2H), 5.77 (dt, *J* = 14.7, 7.2 Hz, 1H), 5.47 – 5.34 (m, 1H), 5.20 (s, 1H), 5.13 (d, *J* = 9.1 Hz, 1H), 5.02 (dq, *J* = 17.5, 3.1 Hz, 1H), 4.32 – 4.19 (m, 2H), 4.00 – 3.91 (m, 1H), 3.86 (q, *J* = 6.8 Hz, 1H), 3.82 – 3.72 (m, 1H), 3.69 – 3.61 (m, 4H), 3.45 (d, *J* = 11.1 Hz, 1H), 3.12 – 3.03 (m, 1H), 2.87 (dt, *J* = 6.2, 3.1 Hz, 1H, minor isomer), 2.76 (dt, *J* = 8.5, 2.8 Hz, 1H, major isomer), 2.58 – 2.42 (m, 1H), 2.33 (q, *J* = 6.8 Hz, 2H), 2.27 – 2.18 (m, 1H), 2.16 – 1.30 (m, 31H), 0.97 – 0.91 (m, 3H), 0.90 – 0.83 (m, 27H), 0.08 – –0.03 (m, 18H). ^{13}C NMR (101 MHz, CD_2Cl_2) δ 171.0, 143.8, 142.7, 137.7, 137.4, 137.0, 136.9, 134.5, 134.4, 134.1, 133.9, 133.7, 133.4, 132.2, 132.1, 129.6, 129.4, 129.3, 128.4, 126.2, 123.5, 119.5, 118.3, 94.8, 82.9, 76.6, 75.3, 73.9, 72.9, 71.9, 70.3, 68.2, 67.7, 67.3, 65.1, 63.9, 63.0, 62.6, 55.9, 55.0, 42.1, 41.6, 39.6, 39.4, 38.3, 37.1, 35.5, 32.7, 32.4, 32.2, 31.8, 31.4, 28.0, 26.1, 26.0, 26.0, 22.8, 21.7, 18.8, 18.6, 18.5, 17.7, 17.5, 12.7, –4.2, –4.3, –4.5, –4.9, –5.2. IR (film) ṽ 2953, 2928, 2856, 1737, 1249, 1147, 1106, 1084, 1006, 969, 835, 776 cm^{-1}. HRMS (ESI$^+$) calcd. for $C_{65}H_{108}O_{12}SSi_3Na$ $[M+Na]^+$: 1219.67615; found: 1219.67619.

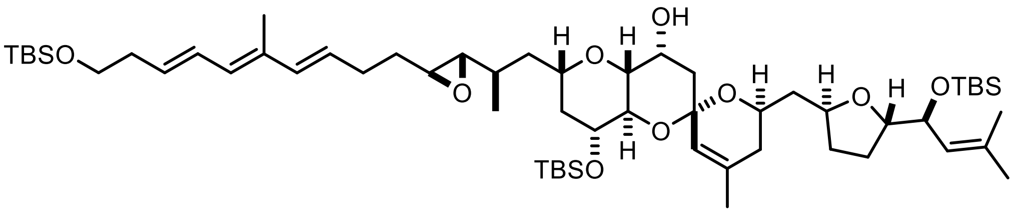

Compound S30. A solution of lithium triethylborohydride (1 M in THF, 80 µL, 80 µmol) in THF (190 µL) was added dropwise to a solution of sulfone **64** (34.0 mg, 28.4 µmol) in THF (6 mL) at –80 °C. After 15 min, the mixture was warmed to –20 °C and stirring was continued for 100 min. A second portion

of lithium triethylborohydride (1 M in THF, 60 µL, 60 µmol) in THF (140 µL) was added dropwise and stirring was continued for 5 min before $PdCl_2$(dppp) (1.9 mg, 3.2 µmol) was introduced and the mixture was warmed to –10 °C. After 80 min, the reaction was quenched with H_2O (10 mL). After reaching ambient temperature, the mixture was extracted with *tert*-butyl methyl ether (3 × 15 mL). The combined organic layers were washed with brine and dried over Na_2SO_4, the solvent was removed under reduced pressure, and the residue was purified by flash chromatography (silica, hexanes/*tert*-butyl methyl ether 4:1 + 1% NEt_3) to provide the title compound as colorless oil (14.1 mg, 49%). $[\alpha]_{20}^{D}$ = –1.4 (c = 1.00, CH_2Cl_2). ^{1}H NMR (400 MHz, CD_2Cl_2) δ 6.55 – 6.31 (m, 1H), 6.17 – 6.08 (m, 1H), 5.96 (d, *J* = 11.1 Hz, 1H), 5.70 (dq, *J* = 15.6, 6.8 Hz, 2H), 5.21 (p, *J* = 1.2 Hz, 1H), 5.18 – 5.07 (m, 1H), 4.27 (dd, *J* = 9.1, 5.4 Hz, 1H), 4.12 – 4.01 (m, 2H), 3.95 (dq, *J* = 9.3, 3.1 Hz, 1H), 3.89 (td, *J* = 7.2, 5.4 Hz, 1H), 3.71 (ddd, *J* = 10.9, 8.7, 5.1 Hz, 1H), 3.65 (t, *J* = 6.7 Hz, 2H), 3.58 – 3.46 (m, 3H), 2.95 (dd, *J* = 9.9, 2.9 Hz, 1H), 2.74 (ddd, *J* = 6.9, 4.8, 2.2 Hz, 1H), 2.43 (dd, *J* = 7.4, 2.2 Hz, 1H), 2.40 – 2.30 (m, 2H), 2.25 (dt, *J* = 10.1, 7.0 Hz, 2H), 2.21 – 2.13 (m, 1H), 2.08 – 1.74 (m, 11H), 1.75 – 1.63 (m, 11H), 1.63 – 1.51 (m, 3H), 1.47 – 1.33 (m, 3H), 0.99 (d, *J* = 6.3 Hz, 3H), 0.88 (s, 9H), 0.88 – 0.85 (m, 18H), 0.04 (s, 9H), 0.04 (s, 3H), 0.03 (s, 3H), 0.01 (s, 3H). ^{13}C NMR (101 MHz, CD_2Cl_2) δ 136.7, 135.6, 133.6, 133.6, 131.6, 129.9, 128.9, 128.4, 126.0, 122.9, 96.6, 83.0, 77.5, 76.4, 73.9, 72.6, 71.9, 70.1, 67.0, 66.3, 63.2, 63.1, 58.1, 41.8, 41.7, 40.9, 39.9, 37.1, 35.4, 33.0, 32.7, 32.7, 29.8, 27.6, 26.1, 26.0, 26.0, 22.8, 18.8, 18.5, 18.4, 17.9, 12.8, –4.3, –4.3, –4.6, –4.9, –5.2. IR (film) ṽ 2953, 2928, 2856, 1471, 1463, 1253, 1104, 1088, 1005, 959, 894, 835, 776 cm^{-1}. HRMS (ESI$^+$) calcd. for $C_{57}H_{102}O_9Si_3Na$ [M+Na]$^+$: 1037.67239; found: 1037.67155.

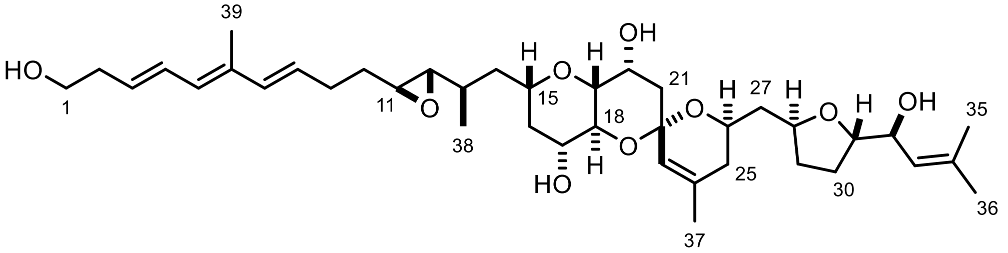

Prorocentin (2). A solution of HF·pyridine (30 µL) in THF (200 µL) was added dropwise to a solution of silyl ether **S30** (9 mg, 9 µmol) and pyridine (90 µL, 1,11 mmol) in THF (600 µL). Stirring was continued for 39 h before pH 7 phosphate buffer solution (15 mL) was added. The mixture was extracted with *tert*-butyl methyl ether (5 × 15 mL), the combined organic layers were washed with brine and dried over Na_2SO_4. The solvent was removed under reduced pressure and the residue was purified by flash chromatography (silica, *tert*-butyl methyl ether + 1% NEt_3) to provide the title compound as white oil (3 mg, 50%). This material was further purified with preparative HPLC (column: 50 mm Eclipse Plus C18,1.8 µm, 4.6 mm i.D., Methanol/H_2O = 75:25, 1.0 mL/min) to give an analytically pure sample of the title compound as a white amorphous solid (2.4 mg, 40%). $[\alpha]_{25}^{D}$ = –12.0 (c = 0.10, MeOH); lit.[1]: $[\alpha]_{25}^{D}$ = –12.7 (c = 0.20, MeOH). ^{1}H NMR (600 MHz, $CDCl_3$ (referenced to $CHCl_3$: δ_H = 7.26 ppm)): δ 6.46 (dd,

J = 15.0, 11.1 Hz, 1H), 6.12 (d, *J* = 15.5 Hz, 1H), 5.98 (d, *J* = 11.1 Hz, 1H), 5.75 – 5.64 (m, 2H), 5.29 (dt, *J* = 2.6, 1.3 Hz, 1H), 5.08 (dhept, *J* = 8.2, 1.4 Hz, 1H), 5.01 (d, *J* = 11.1 Hz, 1H), 4.61 (s, 1H), 4.12 (dd, *J* = 8.9, 8.2 Hz, 1H), 4.10 – 4.01 (m, 3H), 3.95 (ddd, *J* = 9.4, 8.0, 6.2 Hz, 1H), 3.87 – 3.77 (m, 2H), 3.68 (t, *J* = 6.2 Hz, 2H), 3.59 – 3.55 (m, 1H), 3.02 (dd, *J* = 9.5, 3.0 Hz, 1H), 2.74 (td, *J* = 5.7, 2.3 Hz, 1H), 2.57 (s, 1H), 2.48 (dd, *J* = 7.1, 2.3 Hz, 1H), 2.40 (dtd, *J* = 7.3, 6.2, 1.3 Hz, 2H), 2.32 – 2.21 (m, 2H), 2.07 (dd, *J* = 14.3, 3.3 Hz, 1H), 2.04 – 1.95 (m, 3H), 1.88 – 1.75 (m, 8H), 1.75 – 1.70 (m, 9H), 1.69 – 1.60 (m, 4H), 1.56 – 1.32 (m, 5H), 0.98 (d, *J* = 6.5 Hz, 3H). ^{13}C NMR (151 MHz, $CDCl_3$) δ 137.7, 135.4, 135.4, 133.7, 130.2, 129.8, 129.2, 129.0, 128.3, 123.9, 122.1, 96.2, 85.5, 79.5, 76.1, 73.5, 72.1, 70.9, 70.5, 68.1, 66.4, 63.2, 62.0, 57.8, 40.8, 40.0, 39.4, 39.2, 36.6, 35.2, 33.8, 32.2, 32.2, 29.7, 29.3, 28.5, 25.9, 22.9, 18.9, 17.5, 12.8. IR (film) ṽ 3448, 3429, 3419, 3404, 2925, 2861, 1442, 1428, 1381, 1175, 1101, 1086, 1059, 1032, 966 cm^{-1}. HRMS (ESI$^+$) calcd. for $C_{39}H_{60}O_9Na$ [M+Na]$^+$: 695.412954; found: 695.413580.

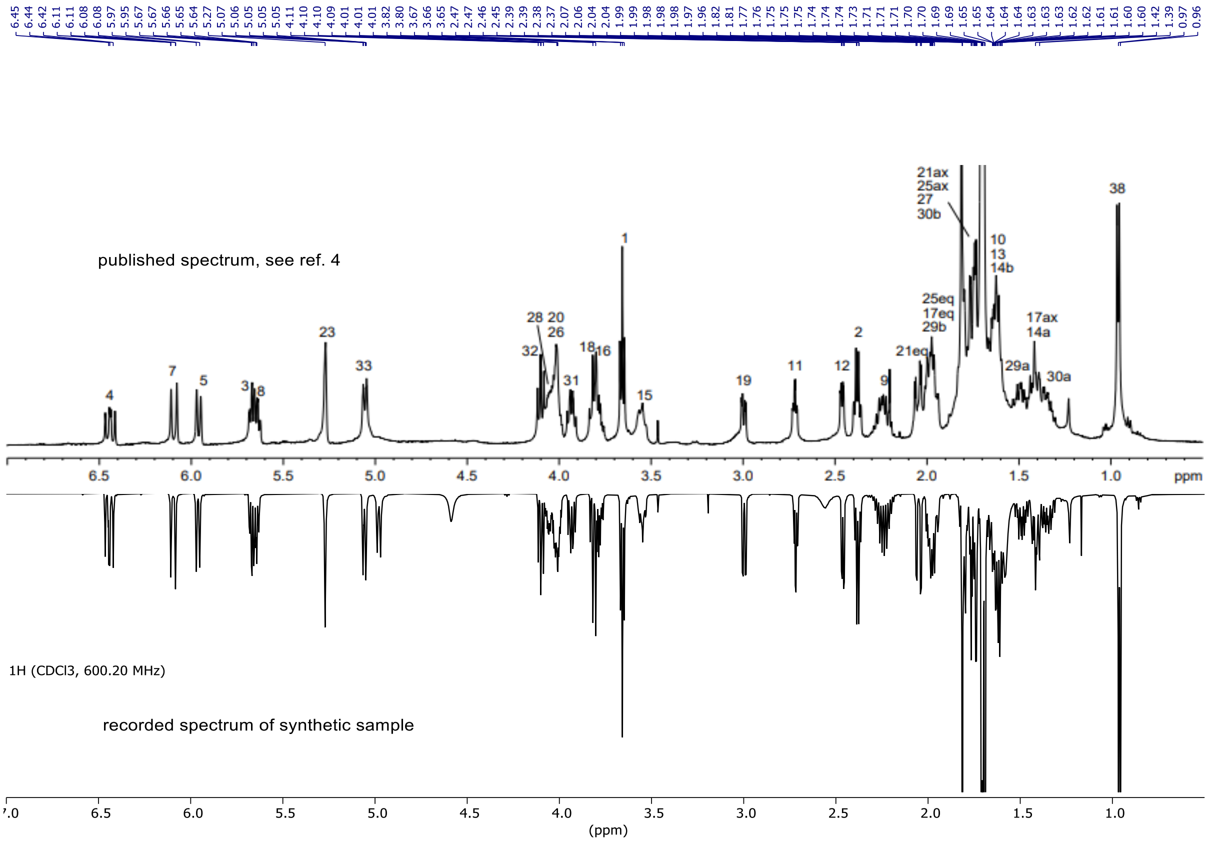

Figure S7. Visual comparison of the ^{1}H NMR data of authentic prorocentin as shown in the SI of ref.[1] with those of synthetic **2**; for the sake of comparison, the spectrum was referenced to $CDCl_3$: δ = 7.24 ppm as used in the isolation paper. Note that the shown spectrum and the corresponding peak labeling (up) were adapted from ref.[1]; for a tabular survey of the exact numbers, see

Table **S12**.

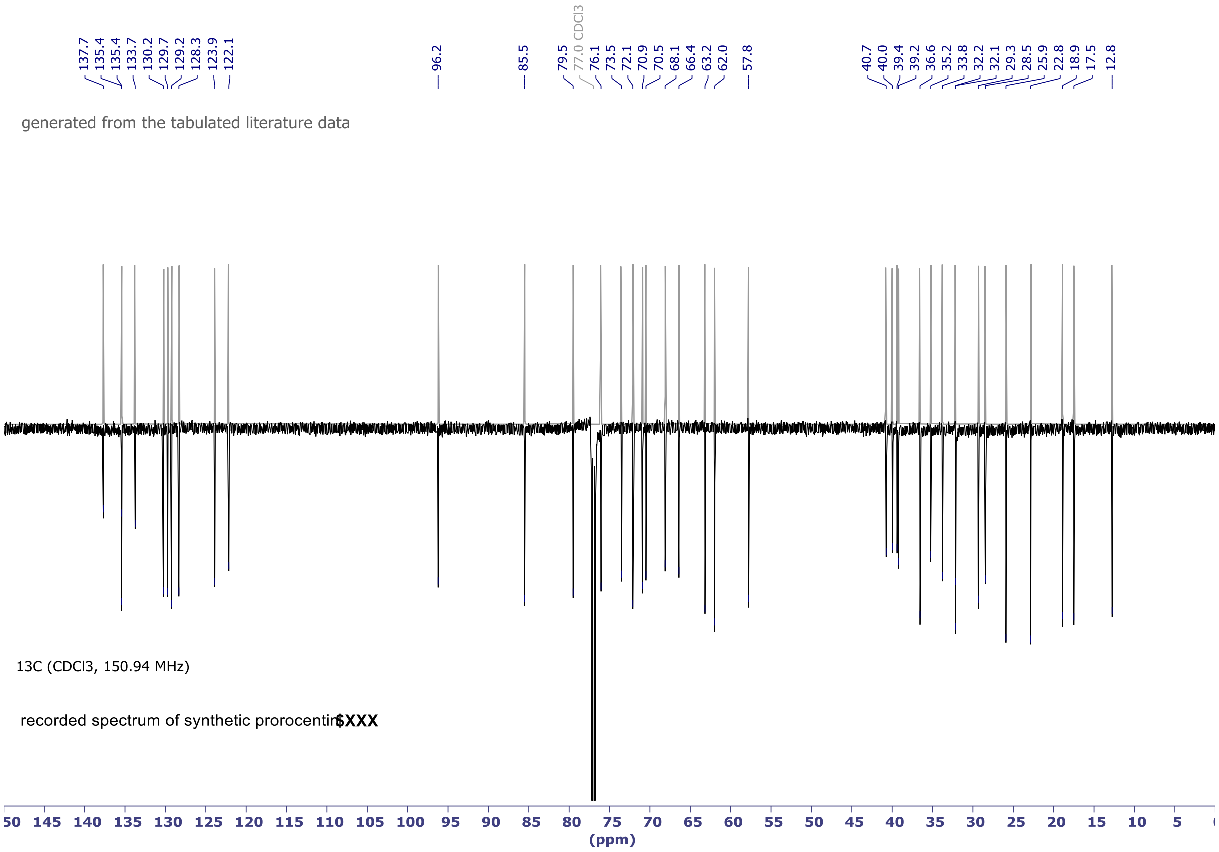

Figure S8. Visual comparison of the ^{13}C NMR data of authentic prorocentin (up) with those of synthetic **2** (down); for the sake of comparison, the spectrum was referenced to $CDCl_3$: δ_C = 77.00 ppm as used in the isolation paper.[1] Note that the spectrum of authentic prorocentin was generated (MestReNova) by converting the tabulated ^{13}C NMR data into a formal spectrum; the intensity of the lines is arbitrarily set to be identical for all signals; for a tabular survey of the exact numbers, see Table S11.

Table S11. NMR data ($CDCl_3$) of synthetic prorocentin (**2**); for the sake of comparison, the spectral data compiled in this Table were referenced to $CDCl_3$: δ_H = 7.24 ppm and δ_C = 77.00 ppm in analogy to the isolation paper;[4] numbering scheme shown in the insert.

Atom number	δ [ppm]	J [Hz]	COSY	HSQC	HMBC	NOESY
1 C	62.0			1	2, 3	
H2	3.66	6.20, 6.20	2	1	2, 3	2, 3
1' O H						
2 C	36.6			2	1, 3, 4	
H2	2.38	7.30, 6.20, 6.20, 1.40	1, 3, 4	2	1, 3, 4	1, 3, 4
3 C	130.2			3	1, 2, 5	
H	5.66	14.90, 7.30, 7.30	2, 4	3	1, 2, 5	1, 2, 5
4 C	129.7			4	2, 5	
H	6.44	15.00, 11.10, 1.40, 1.40	2, 3, 5	4	2, 5, 6	2, 39
5 C	129.2			5	3, 4, 7, 39	
H	5.96	11.10	4, 39	5	3, 4, 7, 39	3, 7
6 C	133.8				4, 7, 8, 39	
7 C	135.4			7	5, 9, 39	
H	6.10	15.50	8, 9	7	5, 6, 9, 39	5, 9
8 C	128.3			8	9, 10	
H	5.66	15.40, 6.90, 6.90	7, 9	8	6, 9, 10	39
9 C	29.3			9	7, 8, 10, 11	
H2	2.25		7, 8, 10	9	7, 8, 10, 11	7, 11
10 C	32.2			10	8, 9, 11, 12	
H2	1.62		9, 11	10	8, 9, 11, 12	11, 12
11 C	57.8			11	9, 10	
H	2.72	5.70, 5.70, 2.30	10, 12	11	9, 10	9, 10, 13, 38
12 C	63.2			12	10, 13, 14a, 14b, 38	
H	2.46	7.10, 2.30	11, 13	12	10, 13, 38	10, 14a, 15', 38
13 C	32.1			13	12, 14a, 14b, 38	
H	1.64		12, 14a, 14b, 38	13	12, 14, 38	11, 15'
14 C	39.2			14a, 14b	13, 16ax, 38	
Ha	1.42	12.70, 10.80, 10.80	13, 14b, 15'	14	12, 13, 15, 16, 38	12, 15', 16eq
Hb	1.66		13, 14a, 15'	14	12, 13, 15, 16, 38	15', 38
15 C	73.5			15'	14a, 14b, 16ax, 19'	
15' H	3.55		14a, 14b, 16ax, 16eq	15		12, 13, 14a, 14b, 16eq, 17, 19', 38
16 C	39.4			16ax, 16eq	14a, 14b	
Hax	1.40		15', 16eq, 17	16	14, 15, 17, 18	18'
Heq	1.99	12.70, 4.70, 2.00	15', 16ax, 17	16	17, 18	14a, 15', 17
17 C	70.5			17	16ax, 16eq, 18', 19'	
H	3.78		16ax, 16eq, 18'	17	18	15', 16eq, 19'
17' O H						
18 C	70.9			18'	16ax, 16eq, 17, 19', 20	
18' H	3.82	9.40, 9.40	17, 19'	18	17, 19, 20	16ax, 20', 26', 28'
19 C	76.1			19'	18', 20, 21eq	
19' H	3.00	9.50, 3.00	18', 20	19	15, 17, 18	15', 17, 20, 21ax

20 C	66.4			20	18', 20', 21eq	
H	4.01	11.00, 3.20, 3.20, 3.20	19', 20', 21ax, 21eq	20	18, 19, 21, 22	19', 21eq
20' O						
H	4.99	11.00	20		20, 21	18', 21eq
21 C	40.0			21ax, 21eq	20, 20'	
Hax	1.75	14.30, 3.10	20, 21eq	21	22, 23	19', 23
Heq	2.05	14.30, 3.40	20, 21ax	21	19, 20, 22	20, 20'
22 C	96.2				20, 21ax, 21eq, 23	
23 C	122.1			23	21ax, 25a, 25b, 37	
H	5.27		25a, 25b, 37	23	22, 25, 37	21ax, 37
24 C	137.7				25a, 25b, 37	
25 C	35.2			25a, 25b	23, 27, 37	
Ha	1.79		23, 25b, 26'	25	23, 24, 26, 37	26'
Hb	1.97		23, 25a, 26'	25	23, 24, 26	
26 C	68.1			26'	25a, 25b, 27, 28'	
26' H	4.01		25a, 25b, 27	26	28	18', 25a, 27, 28'
27 C	40.7			27	29a	
H2	1.74		26', 28'	27	25, 26, 28, 29	26', 28'
28 C	79.5			28'	26', 27, 29a, 30b, 31'	
28' H	4.06		27, 29a, 29b	28	26	18', 26', 27, 29b
29 C	33.8			29a, 29b	27, 30b	
Ha	1.49		28', 29b, 30a, 30b	29	27, 28	
Hb	1.98		28', 29a, 30a, 30b	29	30, 31	28'
30 C	28.5			30a, 30b	29b	
Ha	1.35		29a, 29b, 30b, 31'	30	31, 32	32
Hb	1.81		29a, 29b, 30a, 31'	30	28, 29	31', 33
31 C	85.5			31'	29b, 30a, 32, 33, 35, 36	
31' H	3.93	9.20, 8.00, 6.20	30a, 30b, 32	31	28, 32	30b, 33
32 C	72.1			32	30a, 31'	
H	4.10	9.20, 8.20	31', 33	32	31, 33, 34	30a, 32', 35
32' O						
H	4.59				33	32
33 C	123.9			33	32, 32', 35, 36	
H	5.06	9.10, 1.40, 1.40, 1.40, 1.40, 1.40, 1.40	32, 35, 36	33	31, 35, 36	30b, 31', 36
34 C	135.4				32, 35, 36	
35 C	18.9			35	33, 36	
H3	1.70	1.40, 1.40, 1.40	33	35	31, 33, 34, 36	32
36 C	25.9			36	33, 35	
H3	1.69	1.20	33	36	31, 33, 34, 35	33
37 C	22.8			37	23, 25a	
H3	1.71		23	37	23, 24, 25	23
38 C	17.5			38	12, 13, 14a, 14b	
H3	0.96	6.50	13	38	12, 13, 14	11, 12, 14b, 15'
39 C	12.8			39	5, 7	
H3	1.82	1.20	5	39	5, 6, 7	4, 8

Table S12. Comparison of ^{1}H NMR data ($CDCl_3$) of authentic prorocentin (δ (lit.))[1] and synthetic **2**; for the sake of comparison, the spectral data shown in this Table were referenced to $CHCl_3$: δ_H = 7.24 ppm in analogy to the isolation paper. Numbering scheme as shown in the insert.

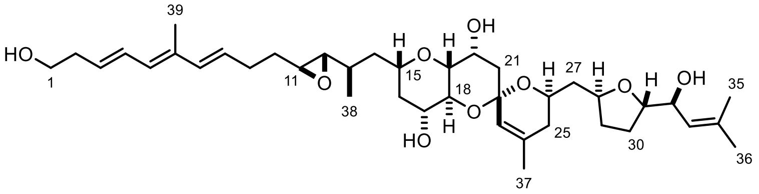

**Atom number**	**δ**(lit.) [ppm]	**δ**(**2**) [ppm]	**Δδ (δ** (lit.) **– δ** (**2**))
1	3.66	3.66	±0.00
2	2.38	2.38	±0.00
3	5.65	5.66	–0.01
4	6.44	6.44	±0.00
5	5.96	5.96	±0.00
6	-	-	-
7	6.10	6.10	±0.00
8	5.56	5.66	–0.10
9	2.25	2.25	±0.00
10	1.62	1.62	±0.00
11	2.72	2.72	±0.00
12	2.46	2.46	±0.00
13	1.62	1.64	–0.02
14	1.42/1.65	1.42/1.66	±0.00/–0.01
15	3.55	3.55	±0.00
16	3.80	1.40*/1.99*	+2.40/+1.81
17	1.41/2.00	3.78*	–2.37/–1.78
18	3.81	3.82	–0.01
19	3.00	3.00	±0.00
20	4.02	4.01	+0.01
21	1.76/2.05	1.75/2.05	+0.01/±0.00
22	-	-	-
23	5.27	5.27	±0.00
24	-	-	-
25	1.79/1.97	1.79/1.97	±0.00/±0.00
26	4.01	4.01	±0.00
27	1.75	1.74	+0.01
28	4.06	4.06	±0.00
29	1.49/1.98	1.49/1.98	±0.00/±0.00
30	1.34/1.81	1.35/1.81	–0.01/±0.00
31	3.93	3.93	±0.00
32	4.10	4.10	±0.00
33	5.02	5.06	–0.04
34	-	-	-
35	1.70	1.70	±0.00
36	1.69	1.69	±0.00
37	1.71	1.71	±0.00
38	0.92	0.96	–0.04
39	1.81	1.82	–0.01

*Those protons were misassigned from the isolation team.

Table S13. Comparison of ^{13}C NMR data ($CDCl_3$) of authentic prorocentin (δ (lit.))[1] and synthetic **2**; for the sake of comparison, the spectral data shown in this Table were referenced to $CDCl_3$: δ_C = 77.00 ppm in analogy to the isolation paper. Numbering scheme as shown in the insert.

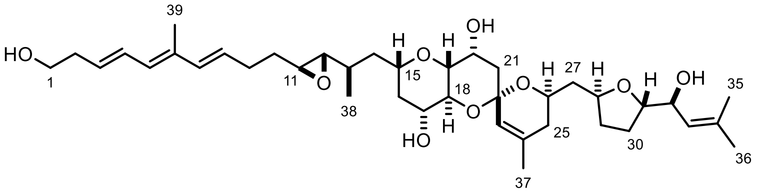

Atom number	δ (lit.) [ppm]	δ (**2**) [ppm]	**Δδ (δ** (lit.) – **δ (2))**
1	62.0	62.0	±0.0
2	36.6	36.6	±0.0
3	130.2	130.2	±0.0
4	129.7	129.7	±0.0
5	129.2	129.2	±0.0
6	133.8	133.8	±0.0
7	135.4	135.4	±0.0
8	128.3	128.3	±0.0
9	29.3	29.3	±0.0
10	32.2	32.2	±0.0
11	57.8	57.8	±0.0
12	63.2	63.2	±0.0
13	32.2	32.1	+0.1
14	39.2	39.2	±0.0
15	73.6	73.5	+0.1
16	70.5*	39.4	+31.1
17	39.4*	70.5	–31.1
18	70.9	70.9	±0.0
19	76.1	76.1	±0.0
20	66.4	66.4	±0.0
21	40.0	40.0	±0.0
22	96.2	96.2	±0.0
23	122.2	122.1	+0.1
24	137.7	137.7	±0.0
25	35.2	35.2	±0.0
26	68.1	68.1	±0.0
27	40.8	40.7	+0.1
28	79.5	79.5	±0.0
29	33.8	33.8	±0.0
30	28.5	28.5	±0.0
31	85.5	85.5	±0.0
32	72.1	72.1	±0.0
33	123.9	123.9	±0.0
34	135.4	135.4	±0.0
35	18.9	18.9	±0.0
36	25.9	25.9	±0.0
37	22.8	22.8	±0.0
38	17.5	17.5	±0.0
39	12.8	12.8	±0.0

*Those two carbons were misassigned from the isolation team.

COPIES OF SPECTRA

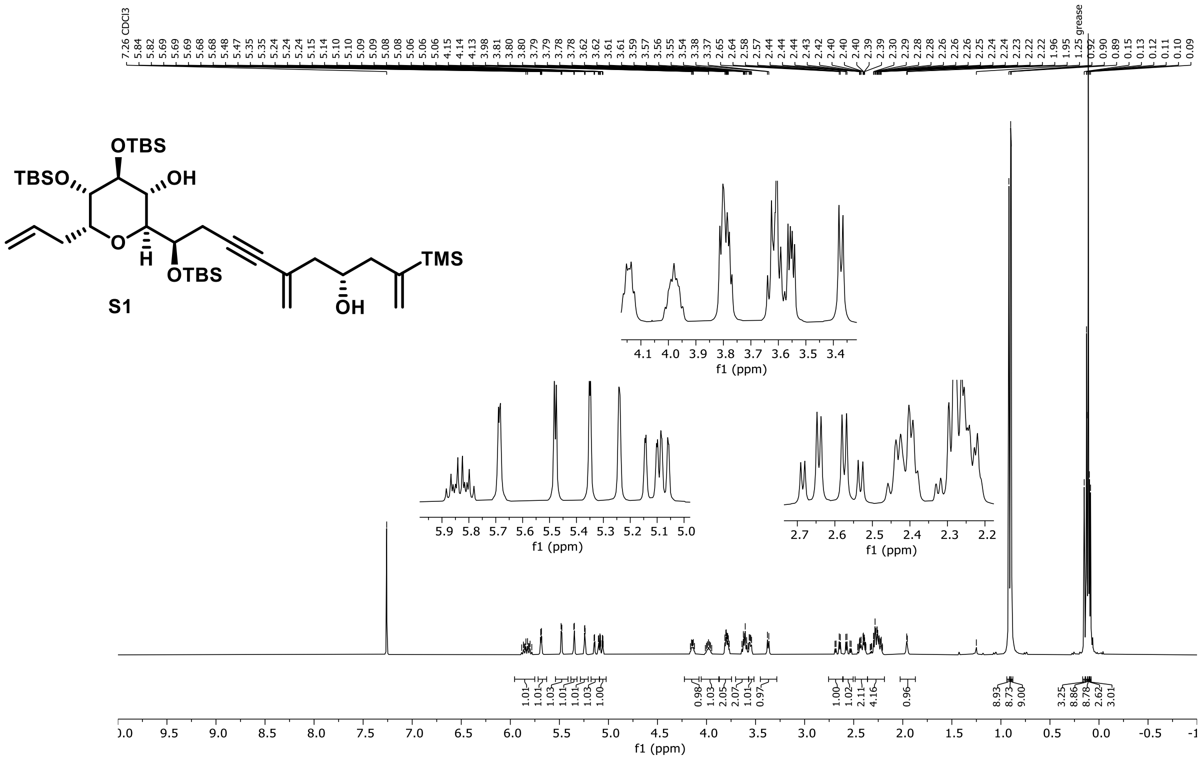

S1

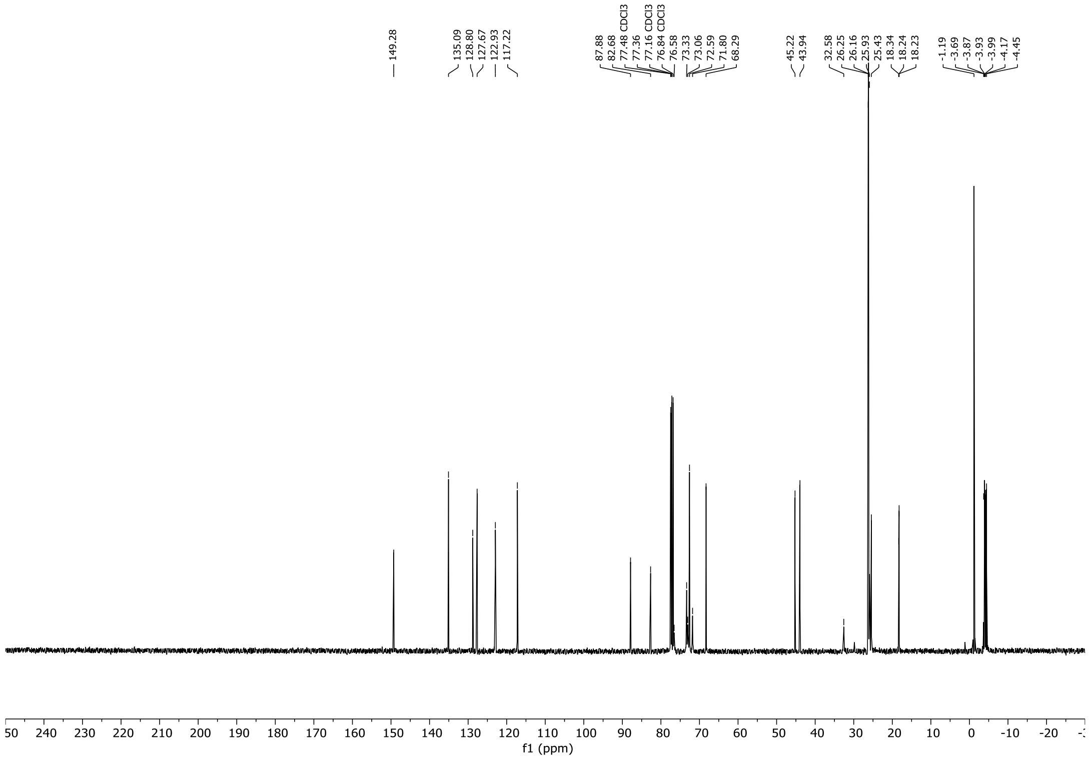

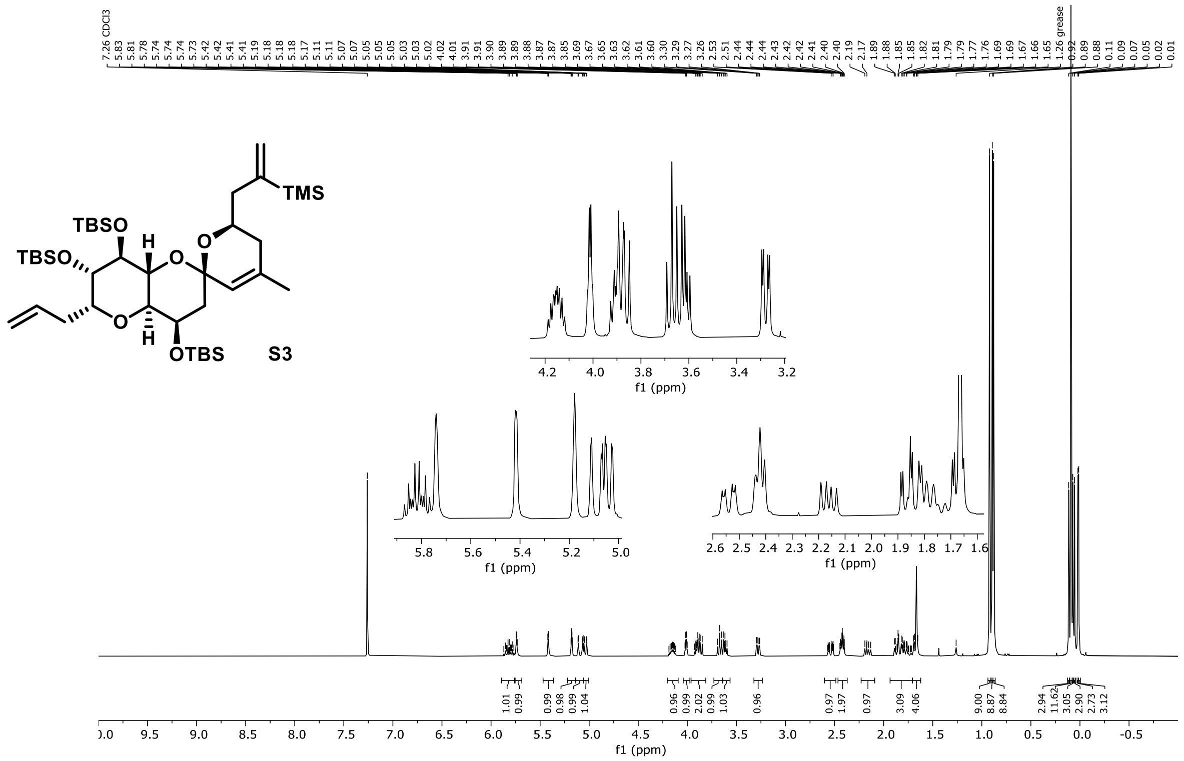
TMS
TBSO
TBSO
H
O
O
O
H
OTBS
S3
f1 (ppm)

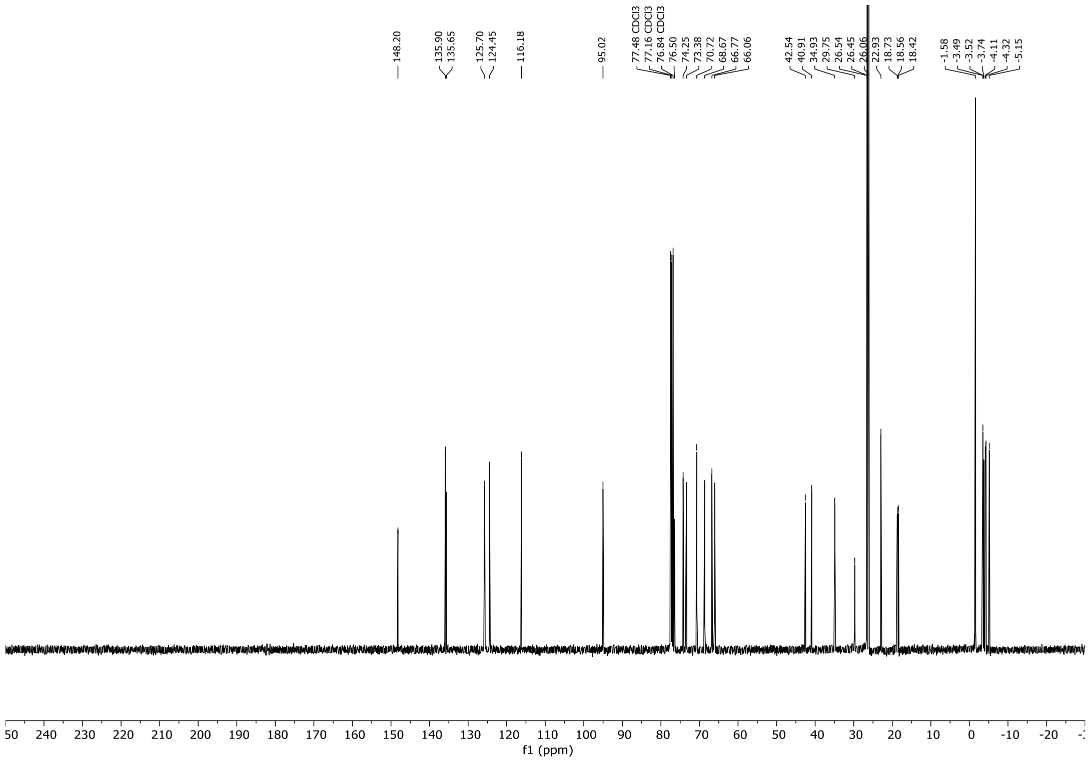
f1 (ppm)

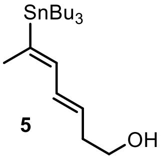

5

1H (CD2Cl2, 400.12 MHz)

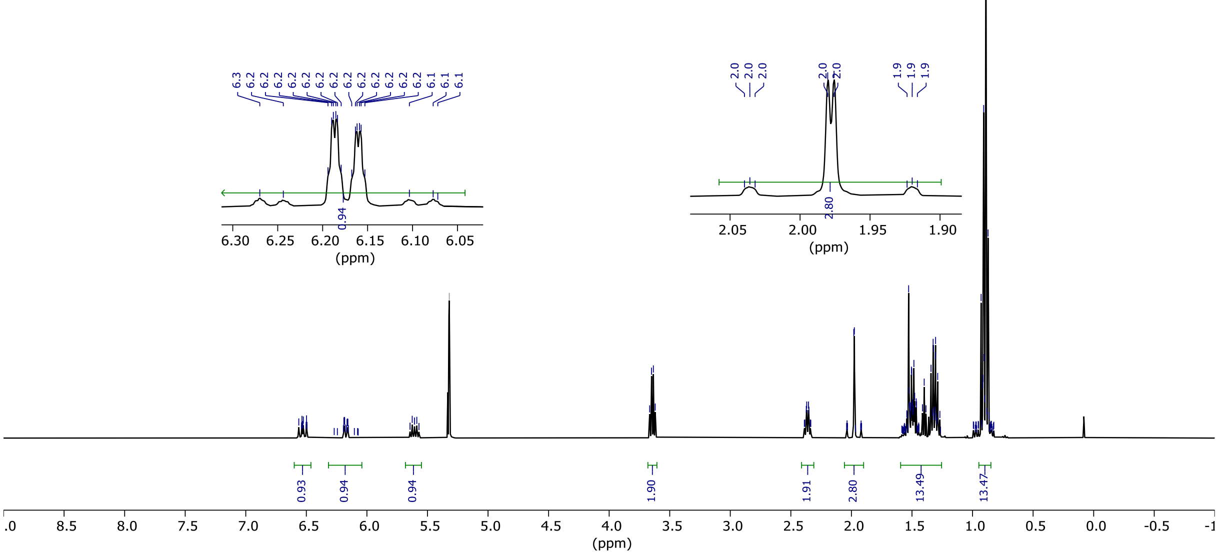

13C (CD2Cl2, 100.62 MHz)

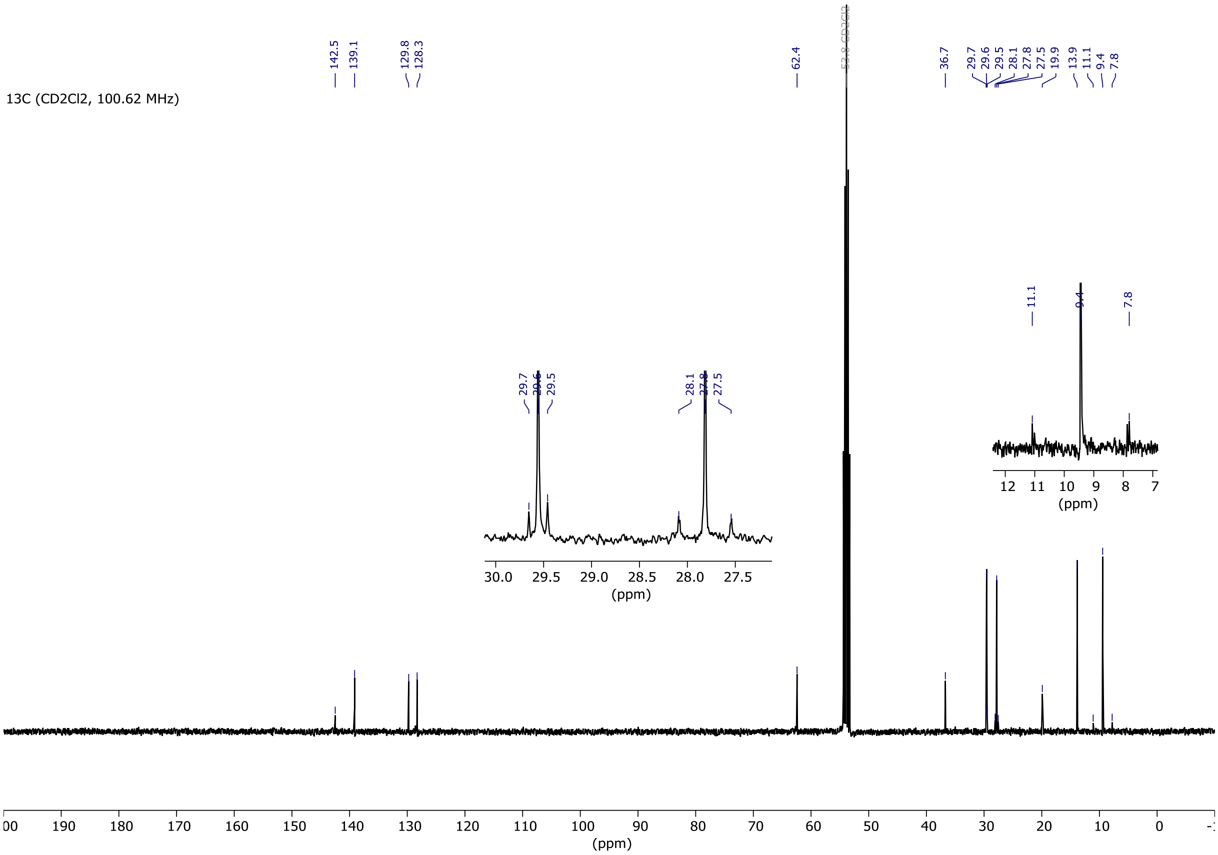

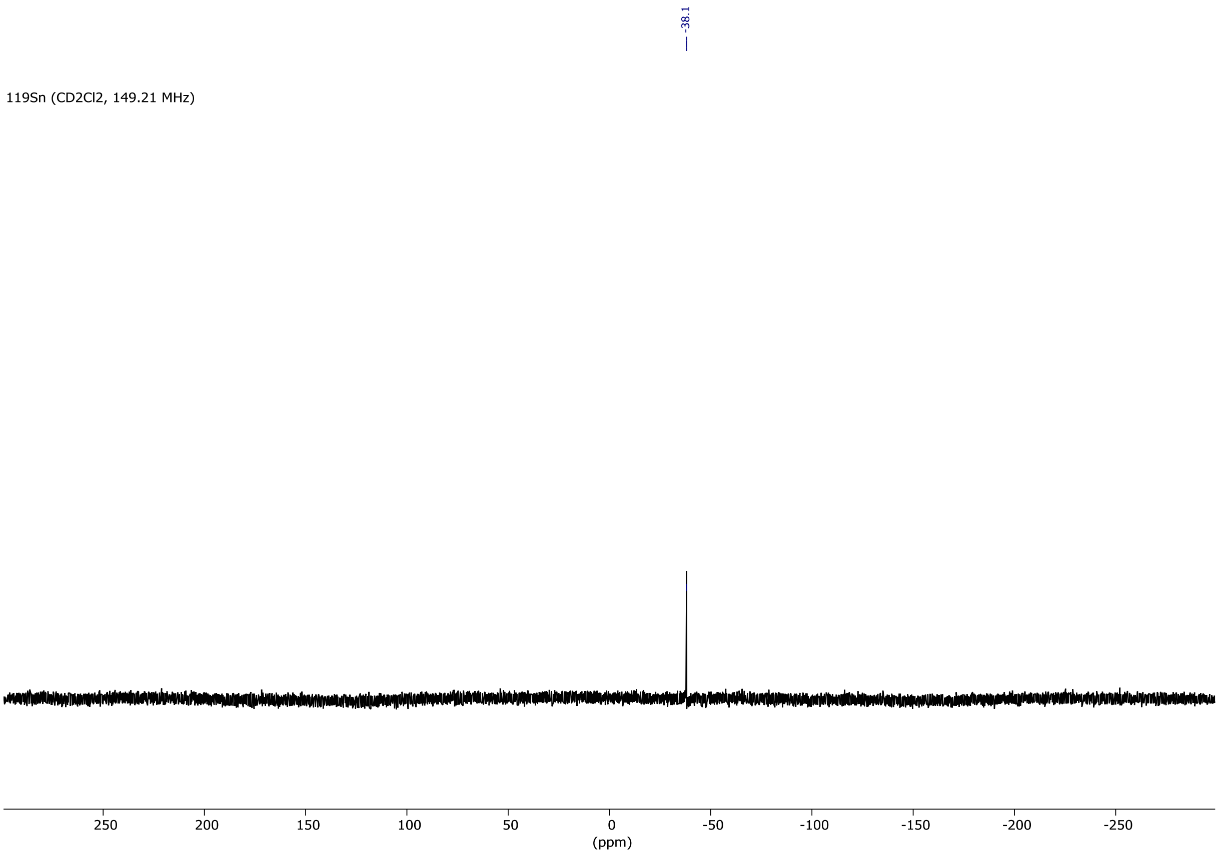
119Sn (CD2Cl2, 149.21 MHz)
-38.1
250
200
150
100
50
0
-50
-100
-150
-200
-250
(ppm)

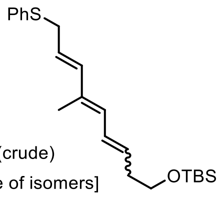

7 (crude)
[mixture of isomers]

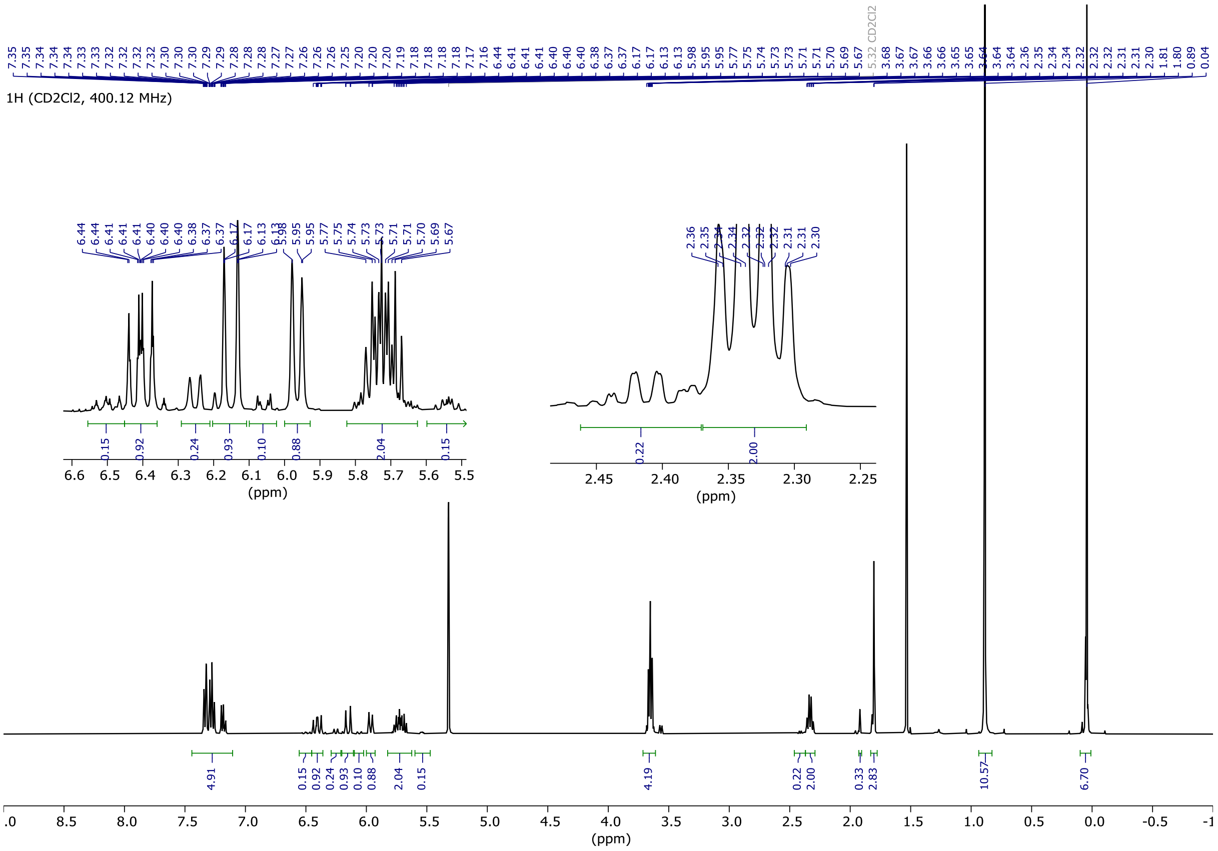

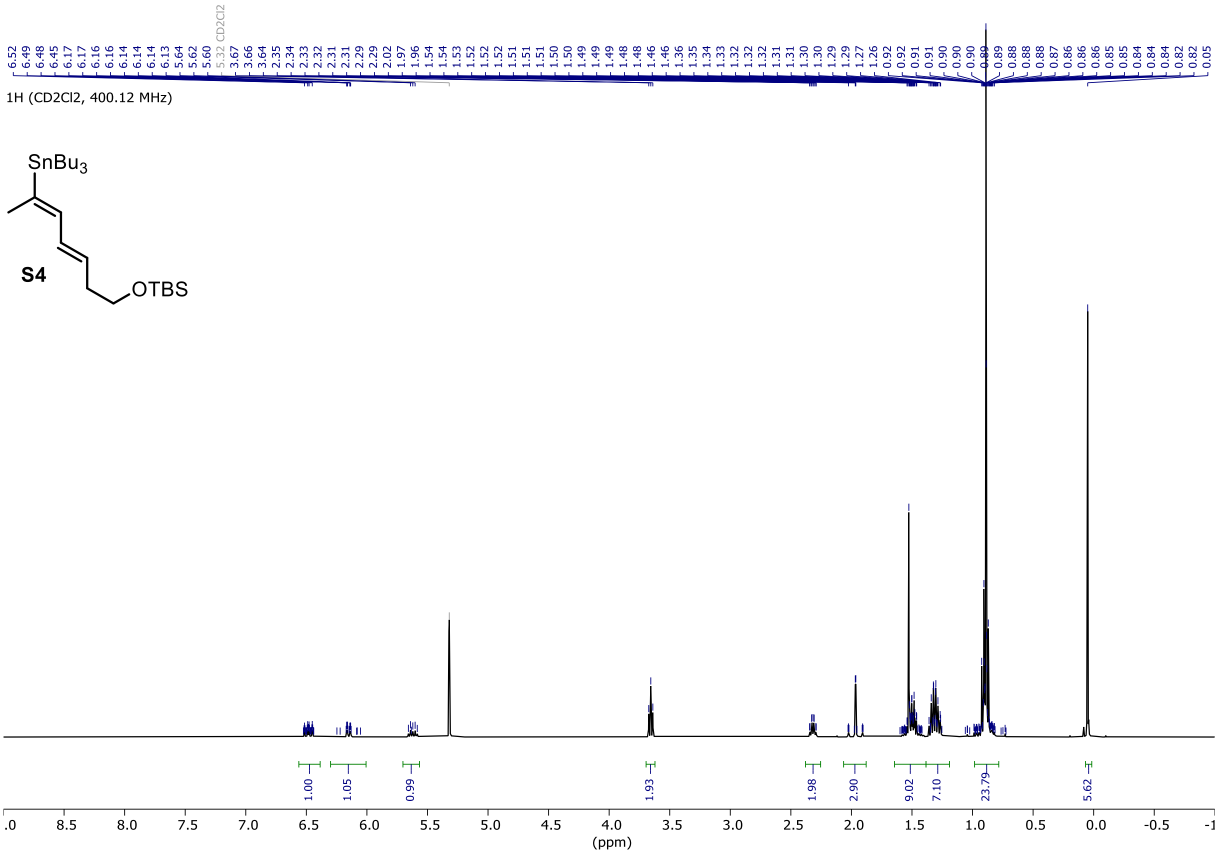
6.52 6.49 6.48 6.45 6.17 6.16 6.16 6.14 6.14 6.14 6.13 5.64 5.62 5.60 5.32 CD2Cl2 3.67 3.66 3.64 2.35 2.34 2.33 2.32 2.31 2.31 2.29 2.29 2.02 1.97 1.96 1.54 1.54 1.53 1.52 1.52 1.51 1.51 1.51 1.50 1.49 1.49 1.49 1.48 1.48 1.46 1.36 1.35 1.34 1.33 1.32 1.32 1.32 1.31 1.31 1.30 1.30 1.29 1.29 1.27 1.26 0.92 0.92 0.91 0.91 0.90 0.90 0.90 0.89 0.89 0.88 0.88 0.88 0.87 0.86 0.86 0.85 0.84 0.84 0.84 0.82 0.05
1H (CD2Cl2, 400.12 MHz)
SnBu3
S4
OTBS
1.00 1.05 0.99 1.93 1.98 2.90 9.02 7.10 23.79 5.62
(ppm)

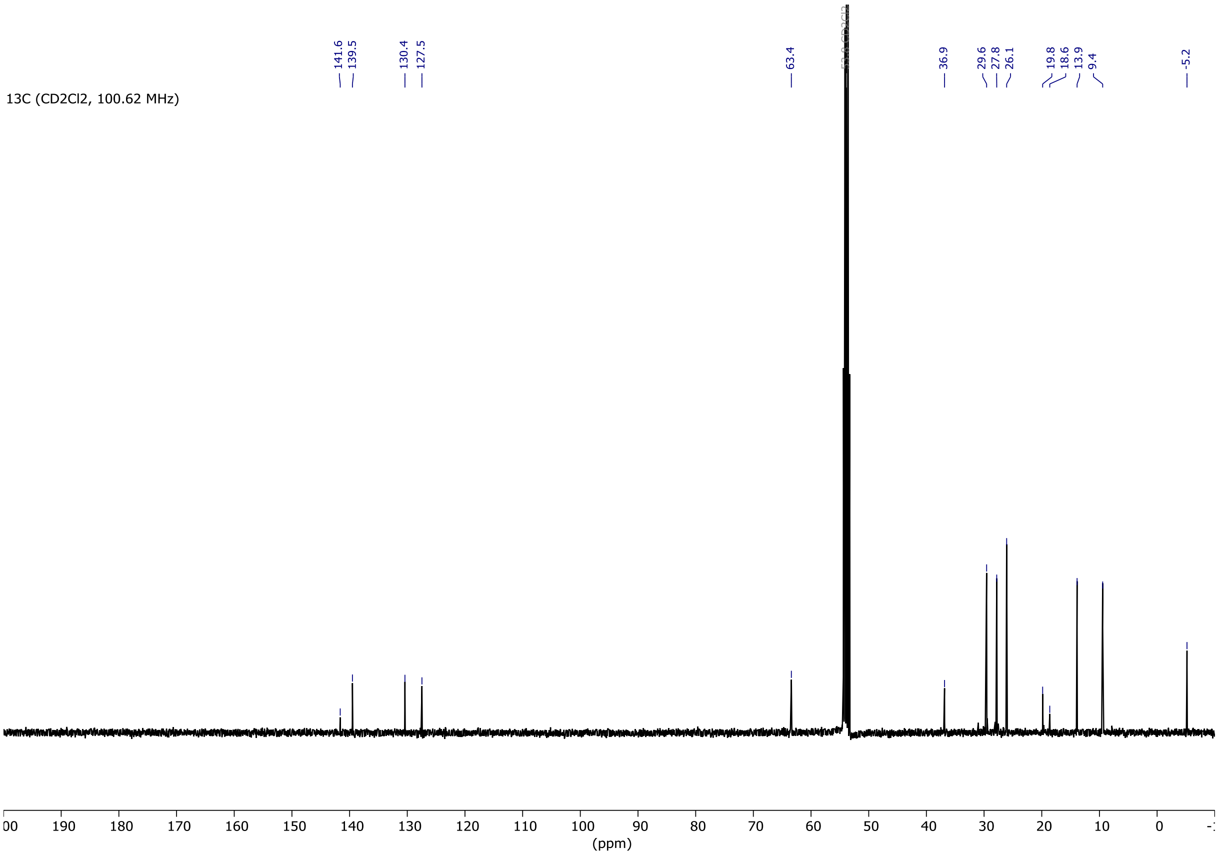
141.6 139.5 130.4 127.5 63.4 36.9 29.6 27.8 26.1 19.8 18.6 13.9 9.4 -5.2
13C (CD2Cl2, 100.62 MHz)
(ppm)

119Sn (CD2Cl2, 149.21 MHz)

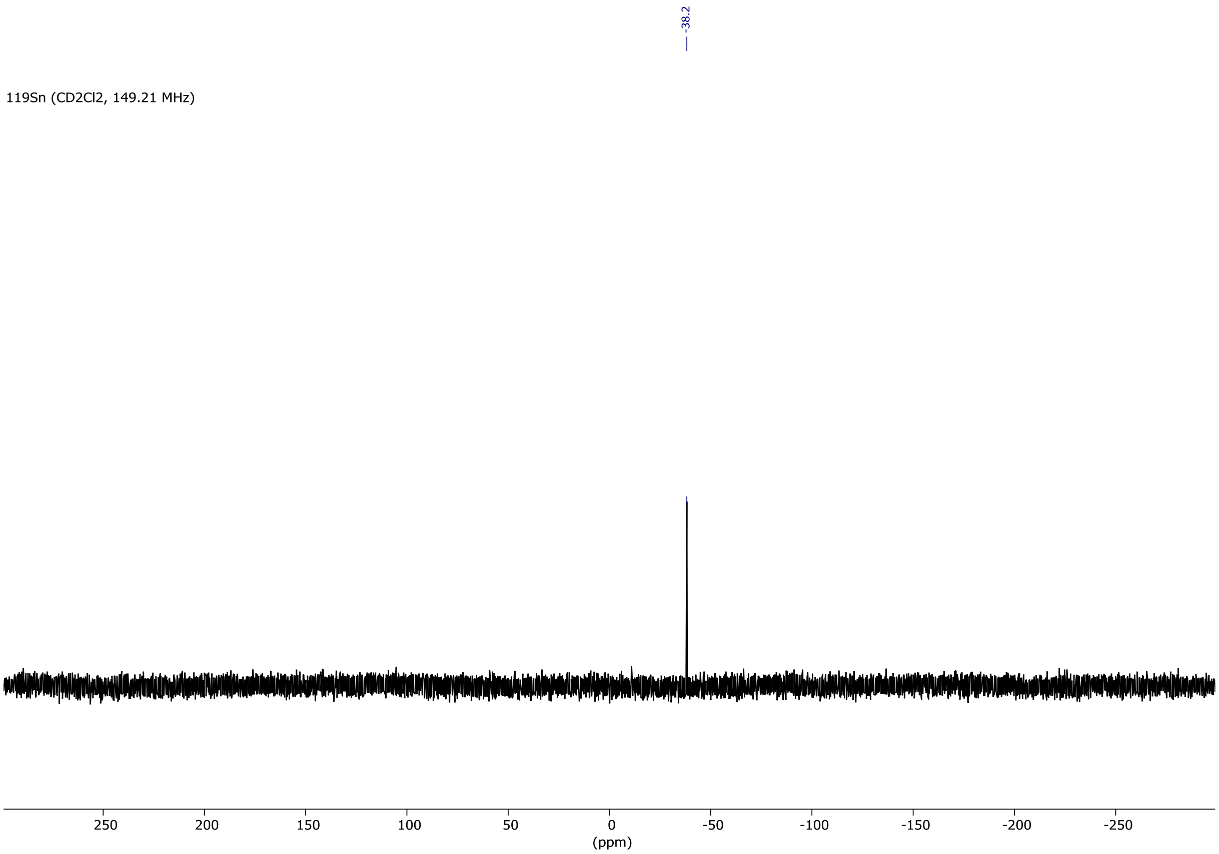

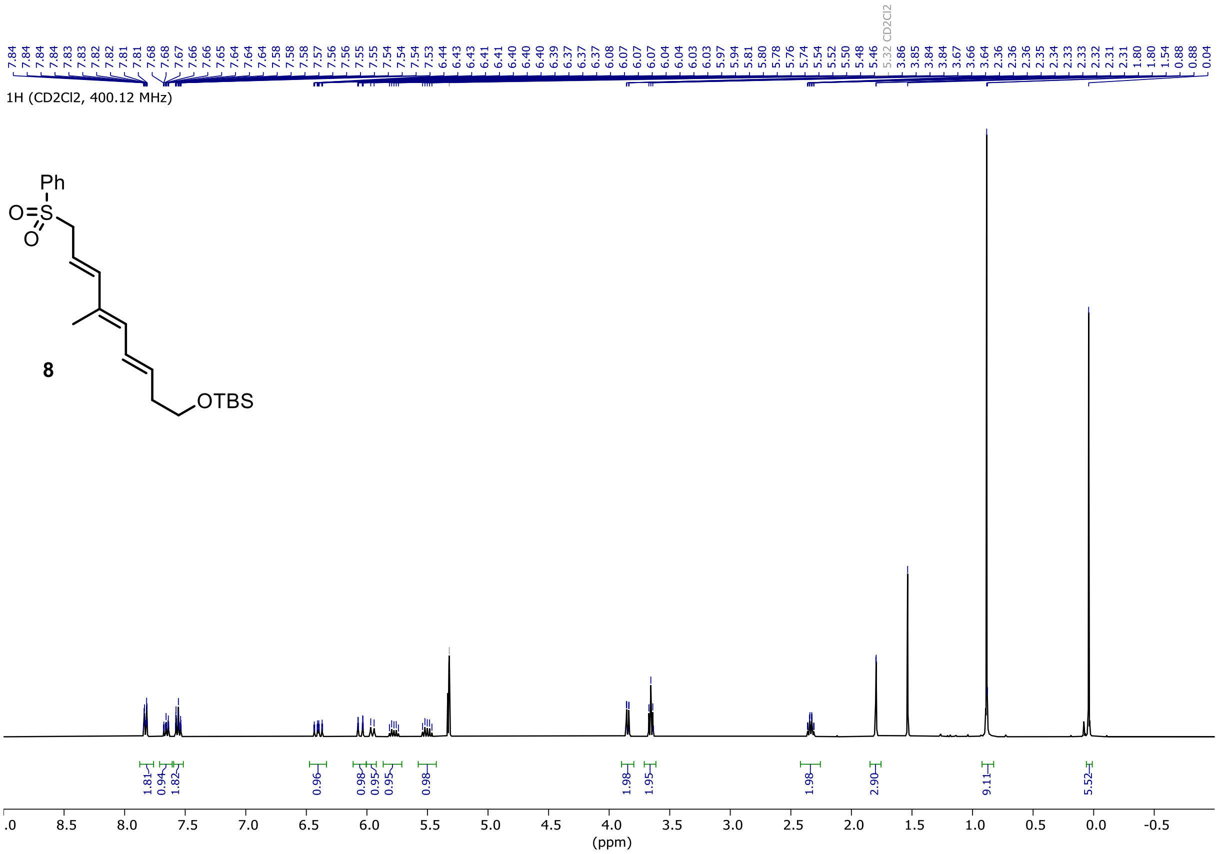
7.84 7.84 7.84 7.83 7.83 7.82 7.82 7.81 7.81 7.68 7.68 7.67 7.66 7.65 7.64 7.64 7.58 7.58 7.57 7.56 7.56 7.55 7.55 7.54 7.54 7.53 6.44 6.43 6.43 6.41 6.40 6.40 6.39 6.37 6.37 6.37 6.08 6.07 6.07 6.04 6.04 6.03 5.97 5.94 5.81 5.80 5.78 5.76 5.74 5.54 5.52 5.50 5.48 5.46 5.32 CD2Cl2 3.86 3.85 3.84 3.84 3.67 3.66 3.64 2.36 2.36 2.36 2.35 2.34 2.33 2.33 2.32 2.31 2.31 1.80 1.80 1.54 0.88 0.04
1H (CD2Cl2, 400.12 MHz)
Ph
O=S
O
8
OTBS
1.81 0.94 1.82 0.96 0.98 0.95 0.95 0.98 1.98 1.95 1.98 2.90 9.11 5.52
(ppm)

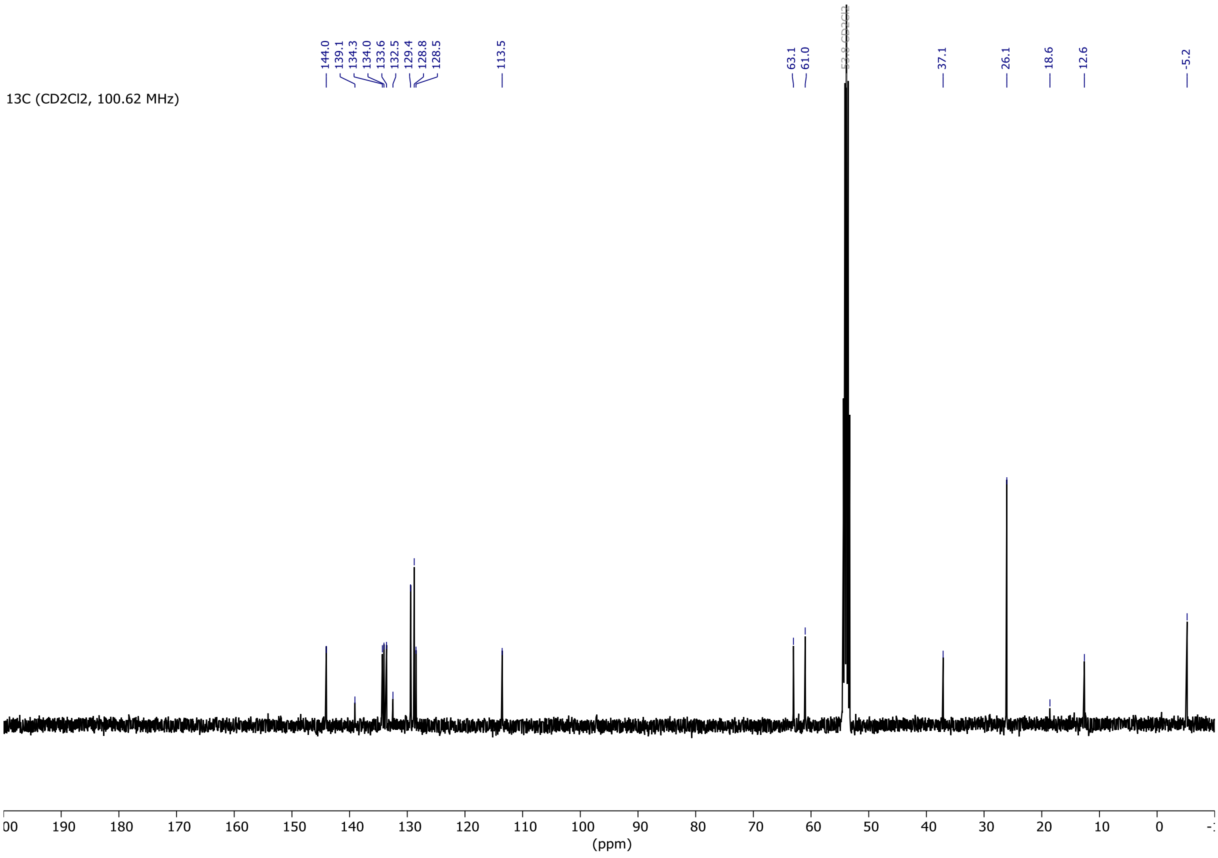
144.0 139.1 134.3 134.0 133.6 132.5 129.4 128.8 128.5 113.5 63.1 61.0 37.1 26.1 18.6 12.6 -5.2
13C (CD2Cl2, 100.62 MHz)
(ppm)

7.26 CDCl3 5.87 5.86 5.85 5.85 5.84 5.84 5.83 5.82 5.81 5.81 5.81 5.81 5.80 5.80 5.79 5.78 5.15 5.15 5.14 5.14 5.14 5.14 5.11 5.11 5.11 5.10 5.06 5.06 5.02 5.02 4.98 4.97 4.95 4.95 3.69 3.68 3.67 3.66 3.65 2.30 2.29 2.28 2.28 2.28 2.27 2.26 2.26 2.23 2.21 2.21 2.19 2.19 2.19 2.19 2.18 2.17 2.17 2.17 2.17 2.16 2.16 2.16 2.15 2.15 2.15 2.14 2.14 2.14 2.13 2.13 2.12 2.12 2.12 1.74 1.59 1.59 1.58 1.57 1.57 1.56 1.55 1.55 1.54 1.53

1H (CDCl3, 400.12 MHz)

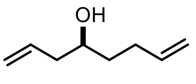

10

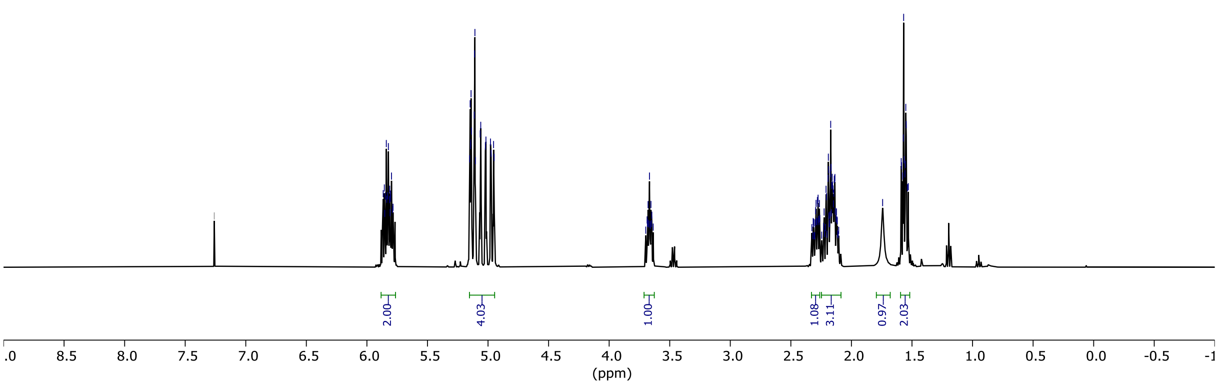

138.6 134.8 118.3 114.9 77.2 CDCl3 70.3 42.1 36.0 30.1

13C (CDCl3, 100.62 MHz)

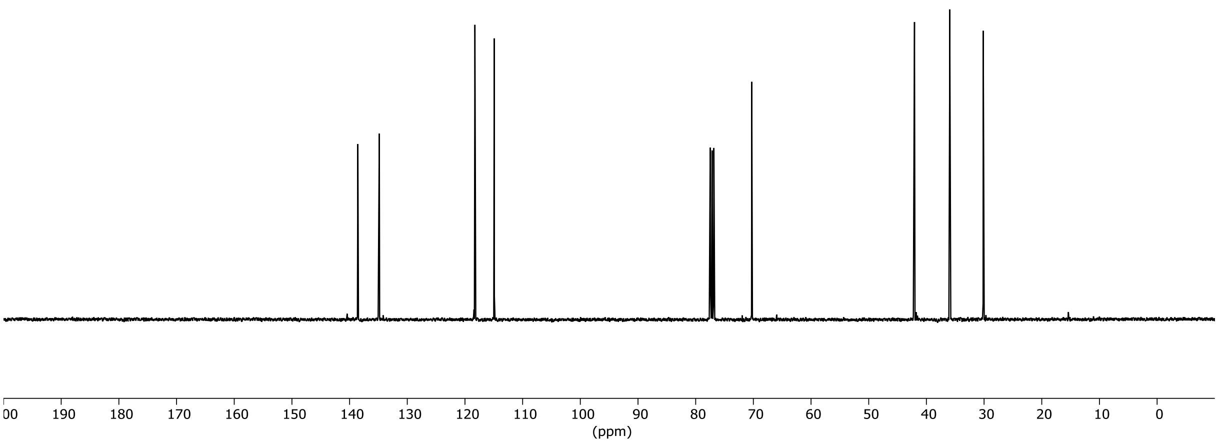

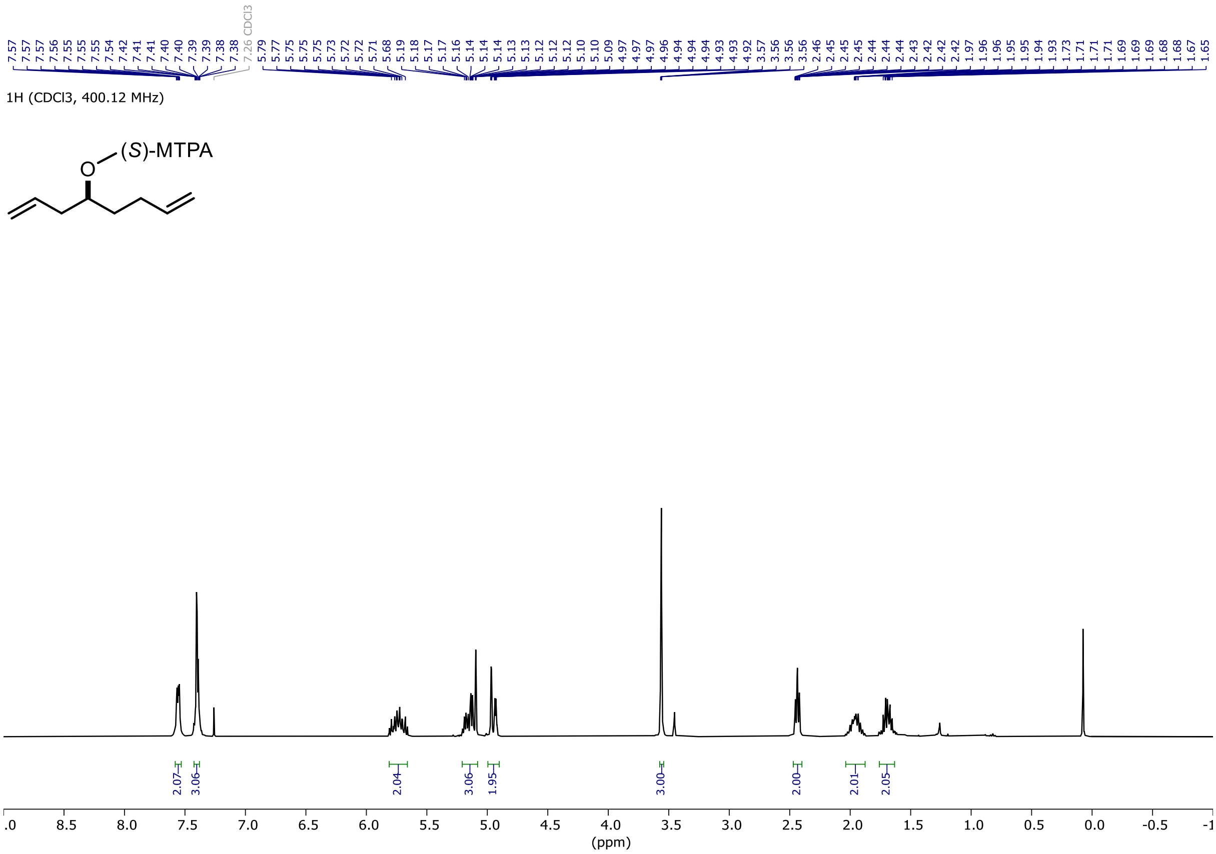

1H (CDCl3, 400.12 MHz)
(S)-MTPA
O
2.07
3.06
2.04
3.06
1.95
3.00
2.00
2.01
2.05
(ppm)

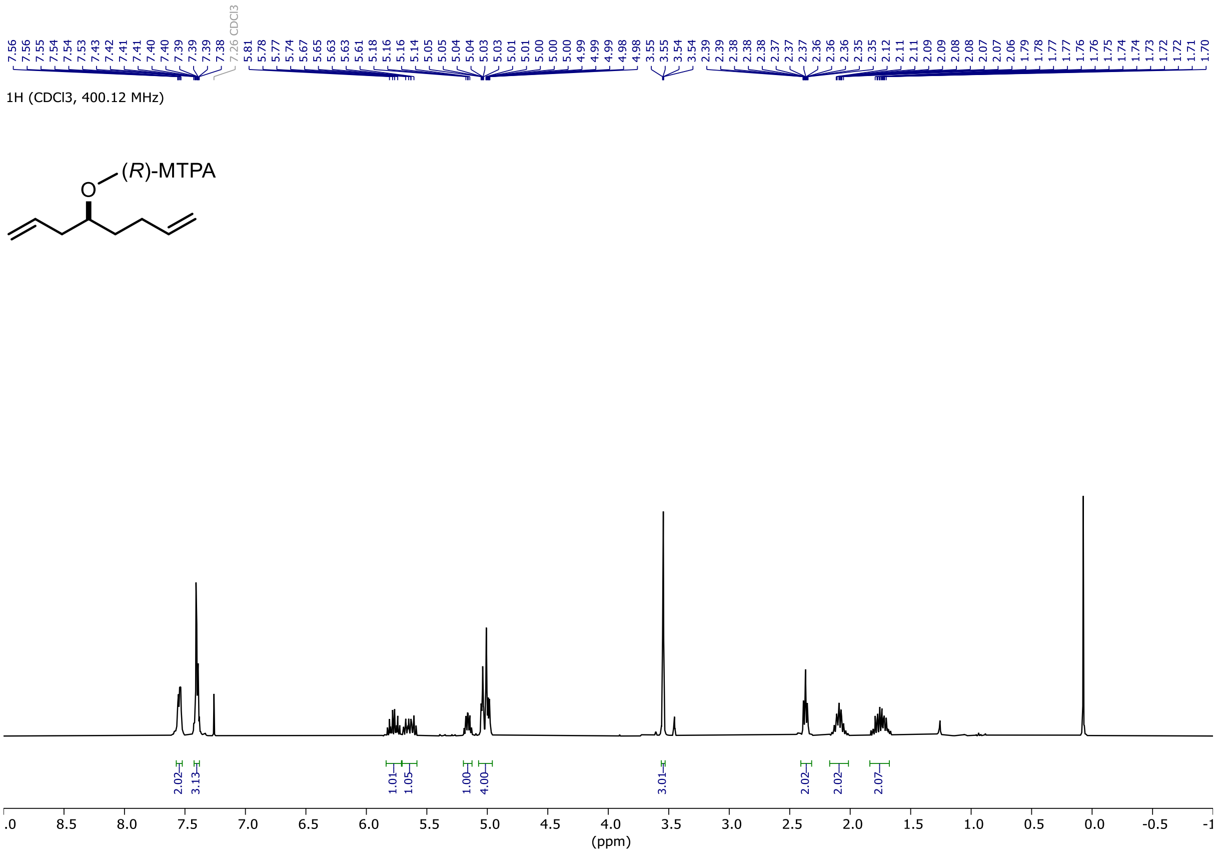

1H (CDCl3, 400.12 MHz)
(R)-MTPA
O
2.02
3.13
1.01
1.05
1.00
4.00
3.01
2.02
2.02
2.07
(ppm)

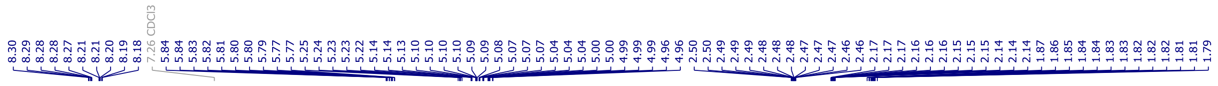

1H (CDCl3, 400.12 MHz)

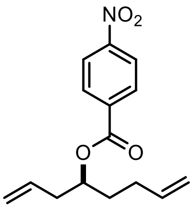

S5

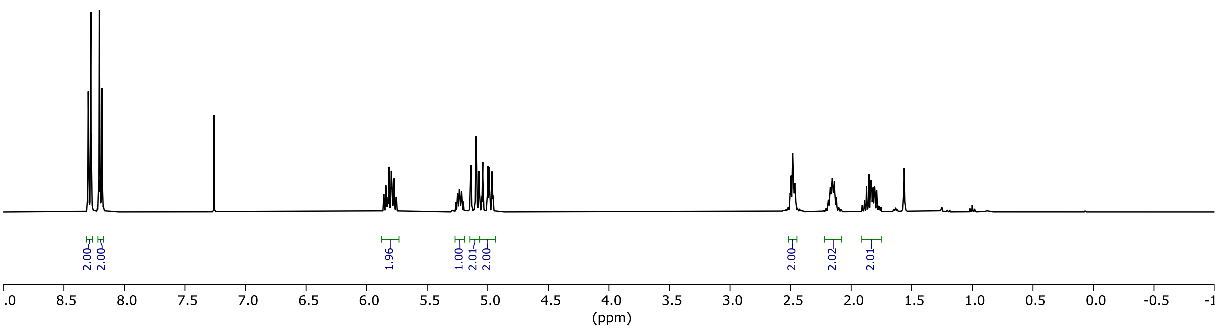

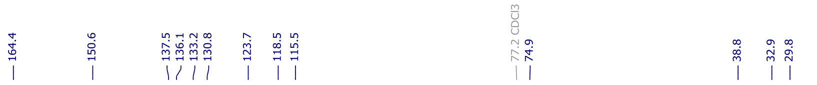

13C (CDCl3, 100.62 MHz)

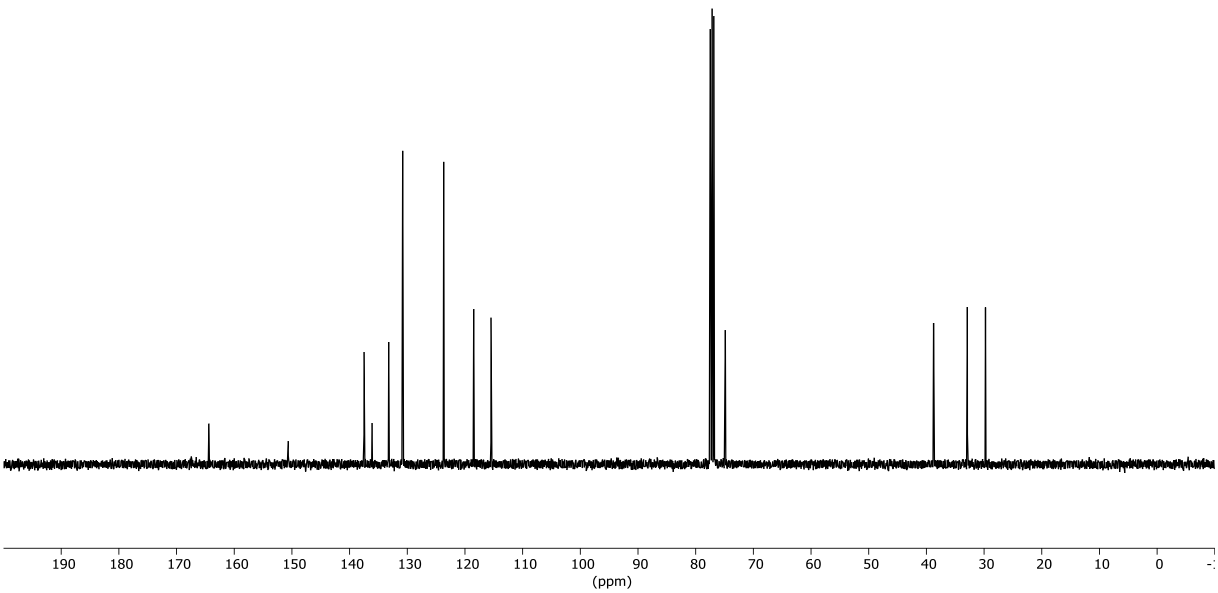

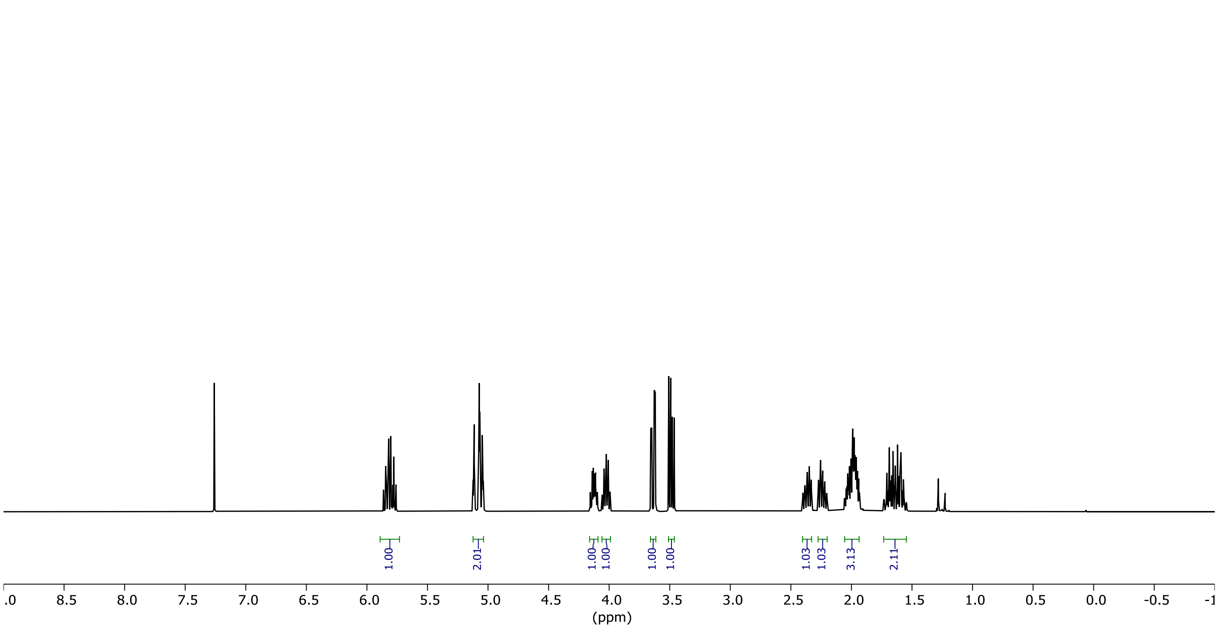
7.26 CDCl3
5.85 5.82 5.80 5.78 5.12 5.12 5.12 5.12 5.11 5.08 5.08 5.07 5.07 5.07 5.06 5.05 5.04 5.05 5.04 5.04 4.14 4.13 4.13 4.12 4.12 4.12 4.11 4.04 4.03 4.02 4.01 3.66 3.65 3.63 3.62 3.51 3.49 3.48 3.46 2.37 2.37 2.35 2.35 2.33 2.27 2.25 2.25 2.24 2.22 2.03 2.02 2.02 2.01 2.00 2.00 2.00 1.99 1.99 1.99 1.98 1.98 1.97 1.97 1.96 1.96 1.95 1.95 1.94 1.71 1.71 1.69 1.68 1.67 1.66 1.64 1.62 1.61 1.60 1.59 1.57 1.57
1H (CDCl3, 400.12 MHz)
1.00 2.01 1.00 1.00 1.00 1.00 1.03 1.03 3.13 2.11
(ppm)

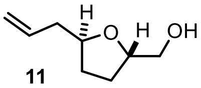

11

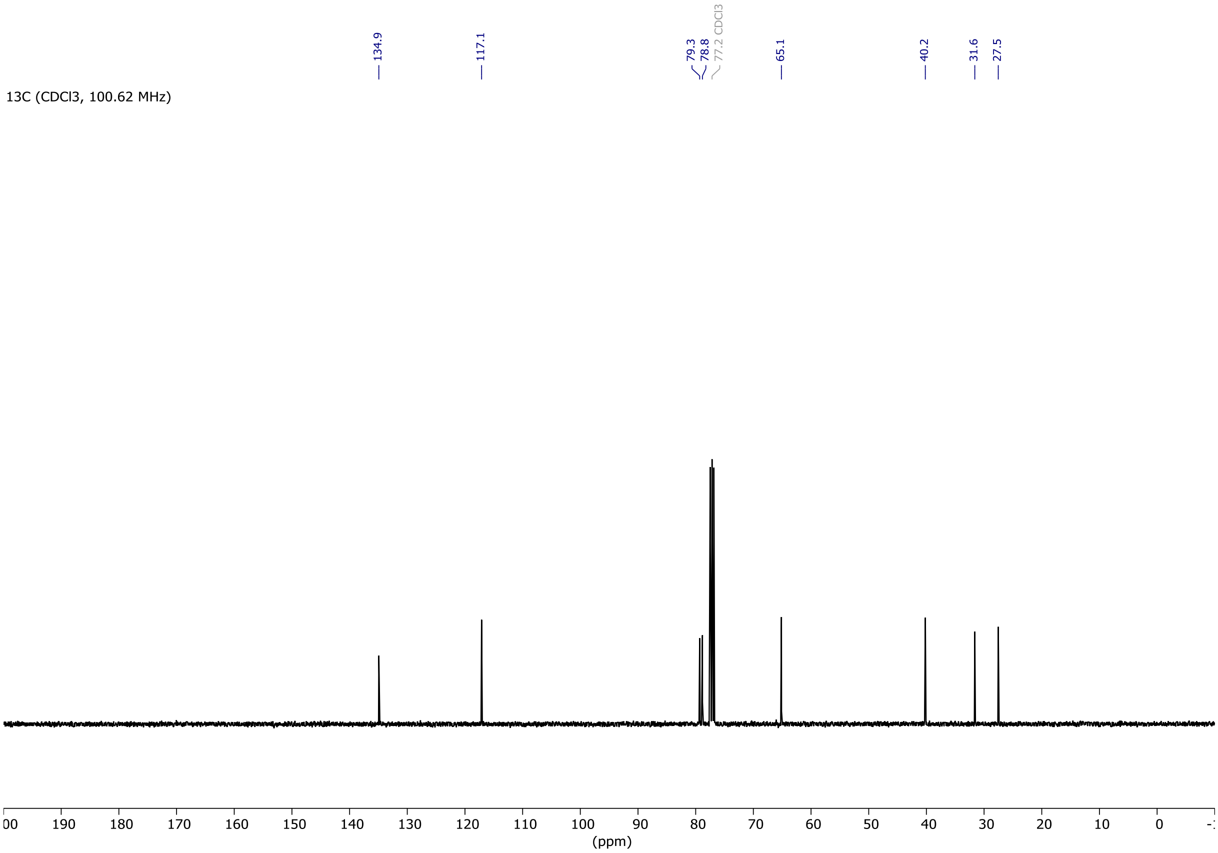
134.9
117.1
79.3
78.8
77.2 CDCl3
65.1
40.2
31.6
27.5
13C (CDCl3, 100.62 MHz)
(ppm)

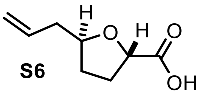

S6

1H (CDCl3, 400.12 MHz)

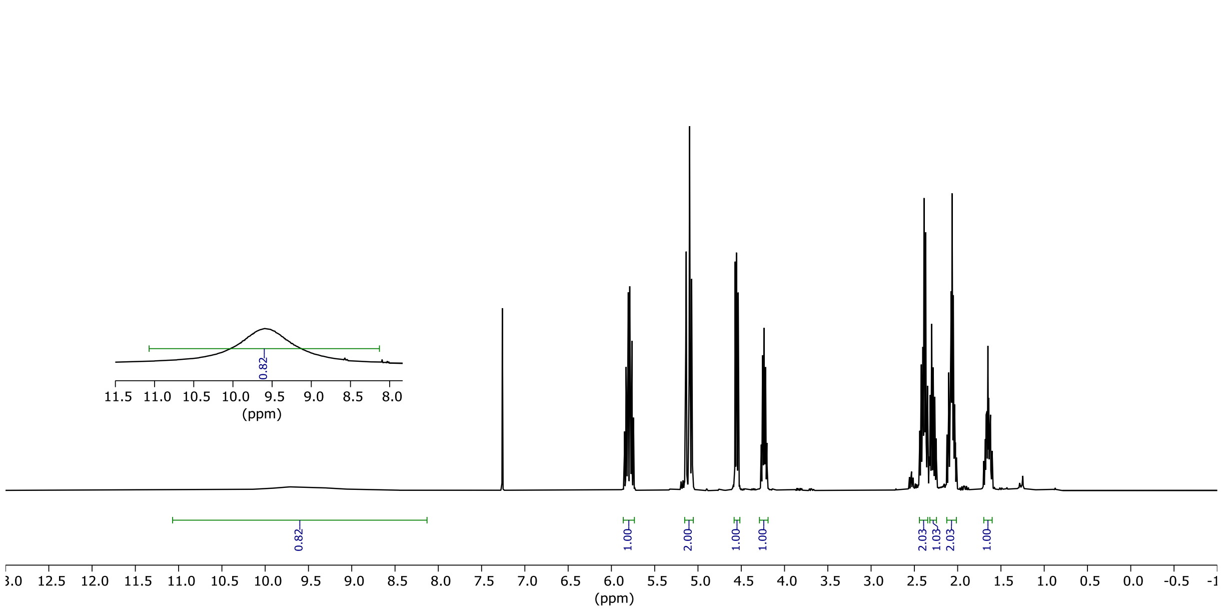

13C (CDCl3, 100.62 MHz)

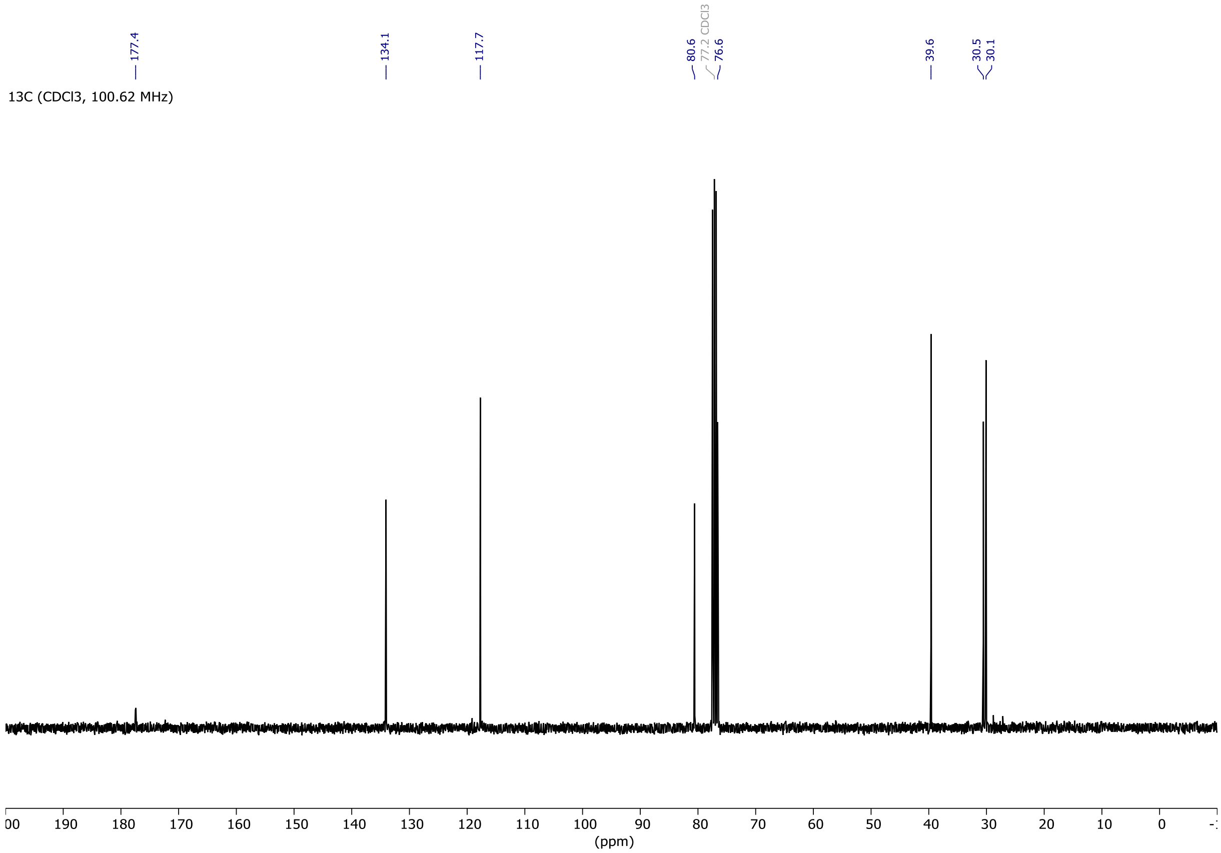

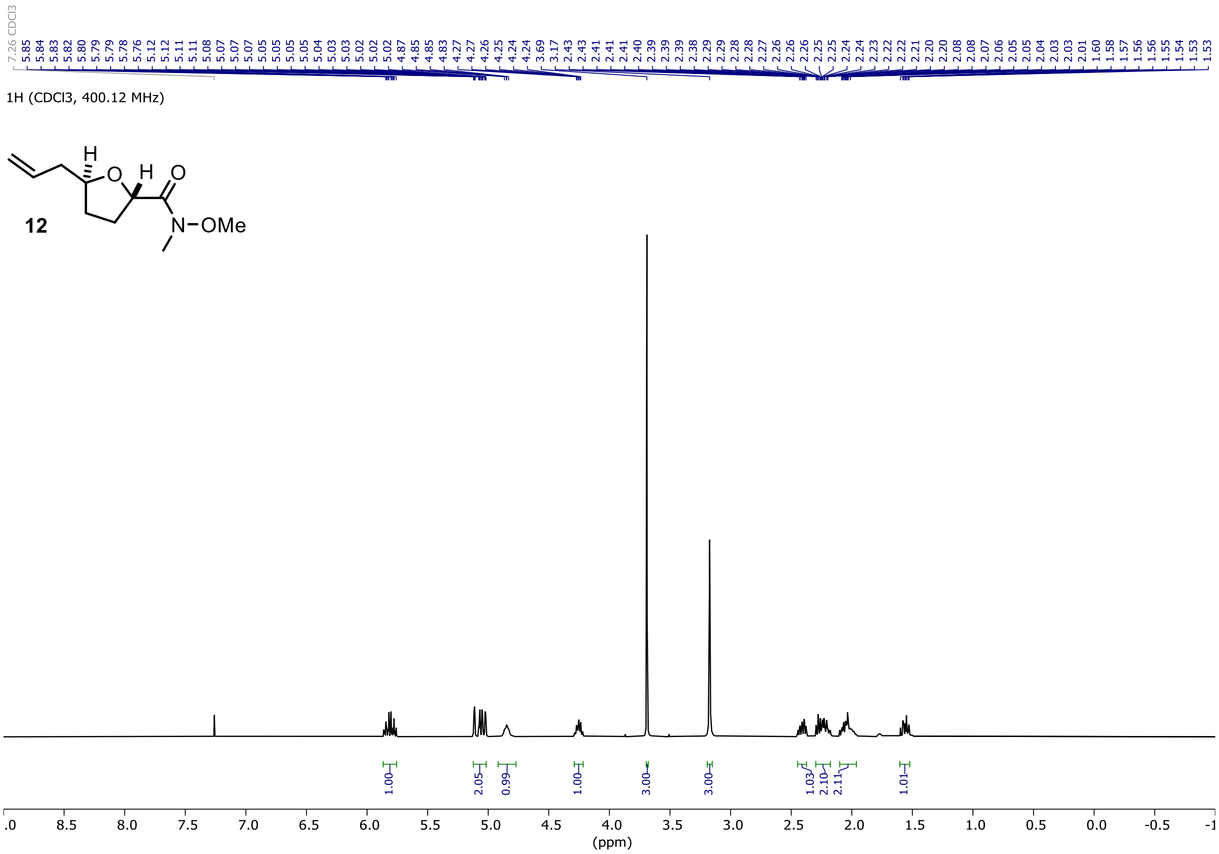
7.26 CDCl3
5.85 5.84 5.83 5.82 5.80 5.79 5.79 5.78 5.76 5.12 5.12 5.11 5.11 5.08 5.07 5.07 5.05 5.05 5.05 5.04 5.03 5.03 5.02 5.02 4.87 4.85 4.85 4.83 4.27 4.26 4.25 4.24 4.24 3.69 3.17 2.43 2.43 2.41 2.41 2.40 2.39 2.39 2.39 2.38 2.29 2.29 2.28 2.28 2.27 2.26 2.26 2.26 2.25 2.25 2.24 2.24 2.23 2.22 2.22 2.21 2.20 2.20 2.08 2.08 2.07 2.06 2.05 2.05 2.04 2.03 2.03 2.01 1.60 1.58 1.57 1.56 1.55 1.54 1.53 1.53
1H (CDCl3, 400.12 MHz)
H
O
H
O
N–OMe
12
1.00
2.05
0.99
1.00
3.00
3.00
1.03
2.10
2.11
1.01
(ppm)

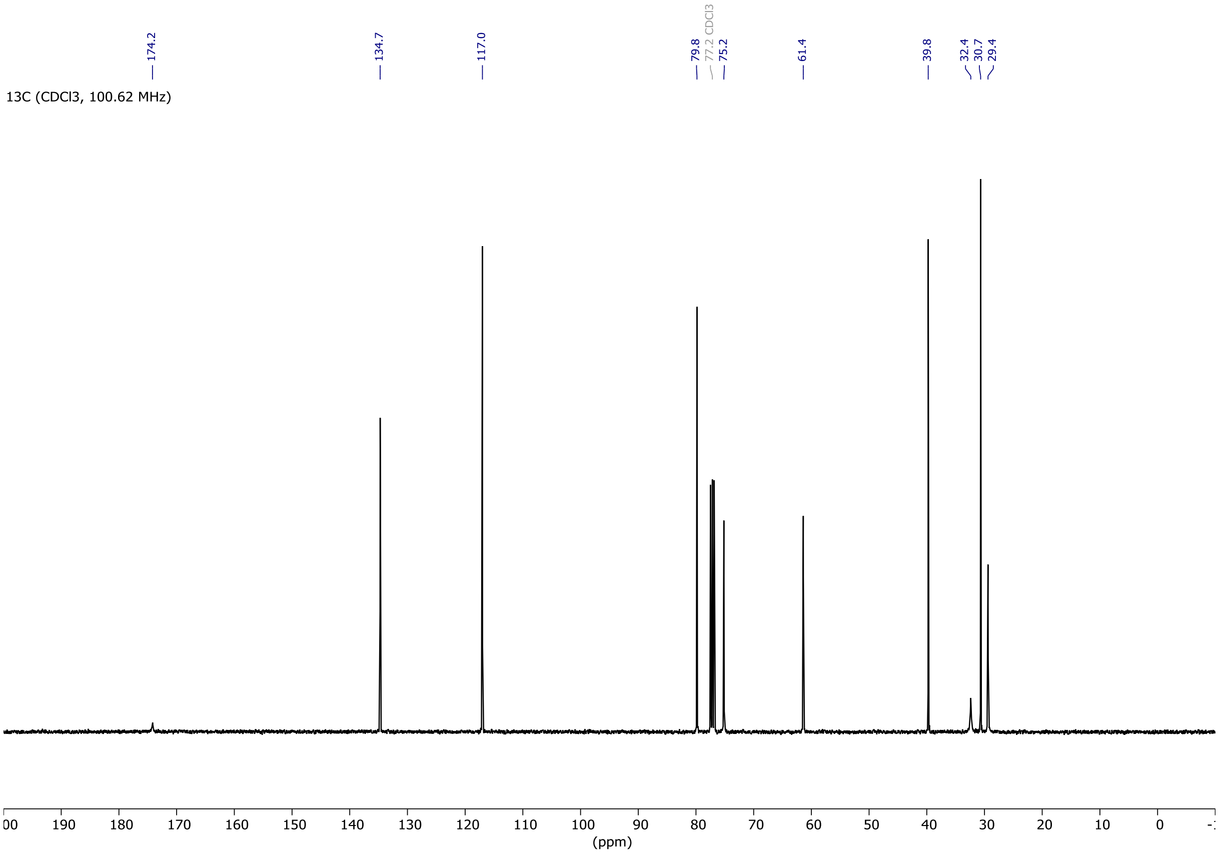
174.2
134.7
117.0
79.8
77.2 CDCl3
75.2
61.4
39.8
32.4
30.7
29.4
13C (CDCl3, 100.62 MHz)
(ppm)

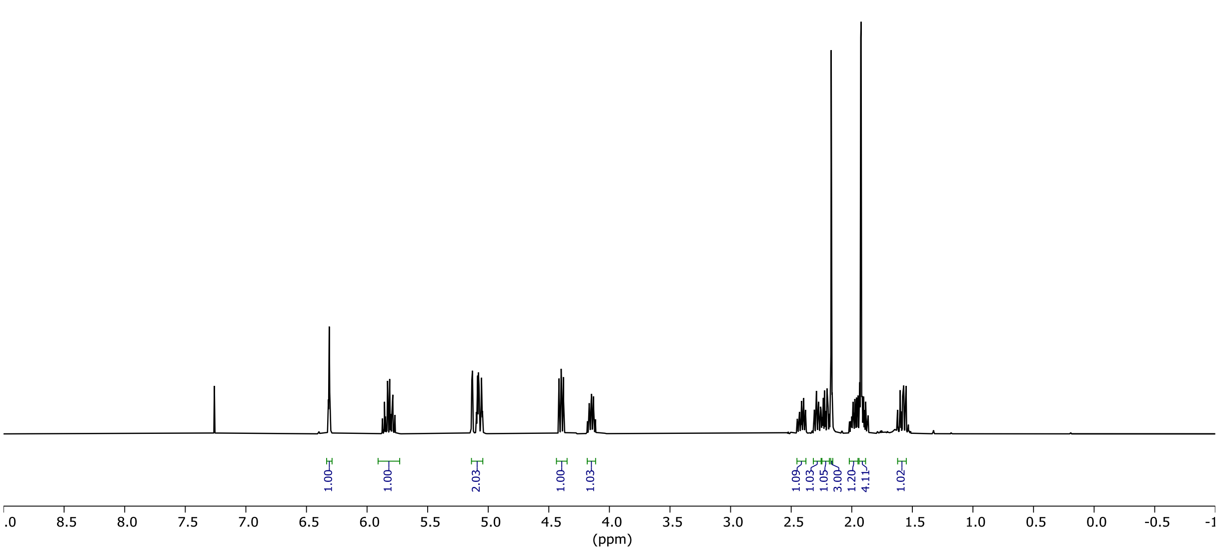
7.26 CDCl3
6.32
6.32
6.31
6.31
6.31
5.86
5.83
5.81
5.79
5.14
5.14
5.13
5.13
5.13
5.13
5.10
5.09
5.09
5.09
5.08
5.08
5.08
5.07
5.06
5.06
5.05
5.05
5.05
4.42
4.40
4.38
4.17
4.15
4.15
4.13
2.44
2.43
2.42
2.41
2.40
2.40
2.38
2.31
2.30
2.29
2.29
2.27
2.27
2.26
2.25
2.23
2.23
2.22
2.20
2.20
2.19
2.17
2.17
1.99
1.97
1.97
1.96
1.95
1.95
1.95
1.94
1.93
1.93
1.92
1.92
1.91
1.91
1.90
1.89
1.88
1.62
1.60
1.59
1.58
1.57
1.56
1.55
1H (CDCl3, 400.12 MHz)
1.00
1.00
2.03
1.00
1.03
1.09
1.03
1.05
3.00
1.20
4.11
1.02
(ppm)

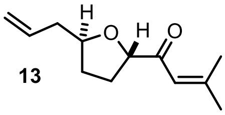

13

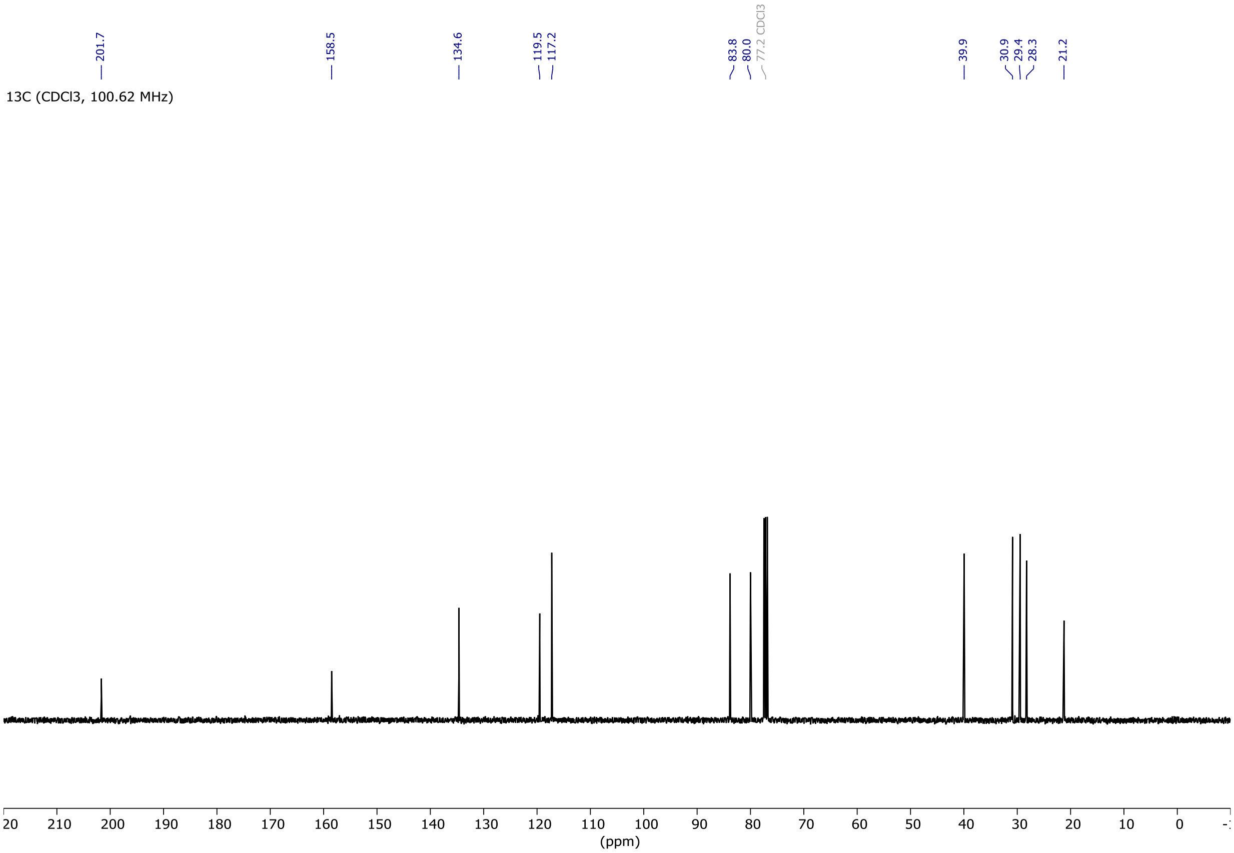
201.7
158.5
134.6
119.5
117.2
83.8
80.0
77.2 CDCl3
39.9
30.9
29.4
28.3
21.2
13C (CDCl3, 100.62 MHz)
(ppm)

1H (CDCl3, 400.12 MHz)

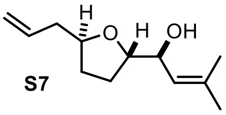

S7

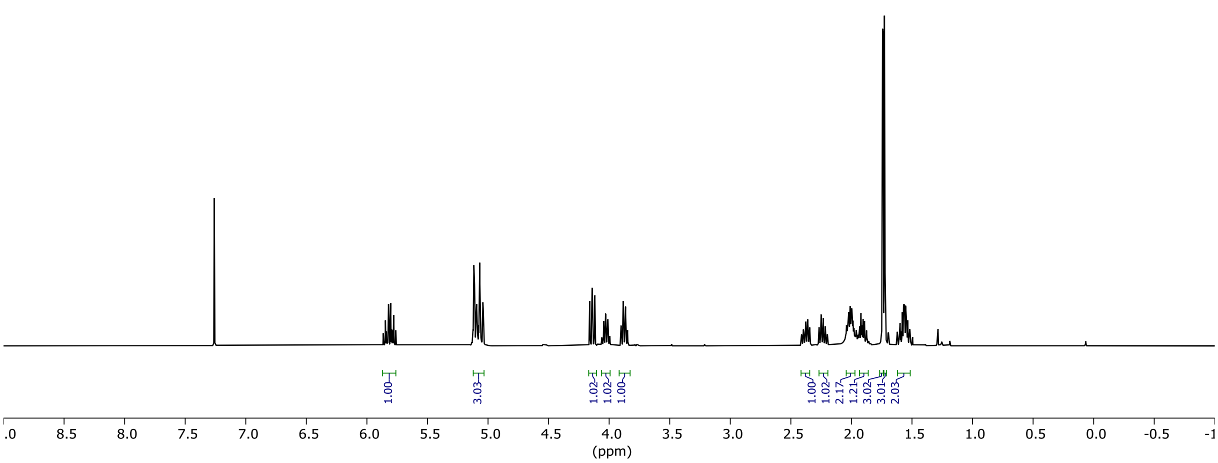

13C (CDCl3, 100.62 MHz)

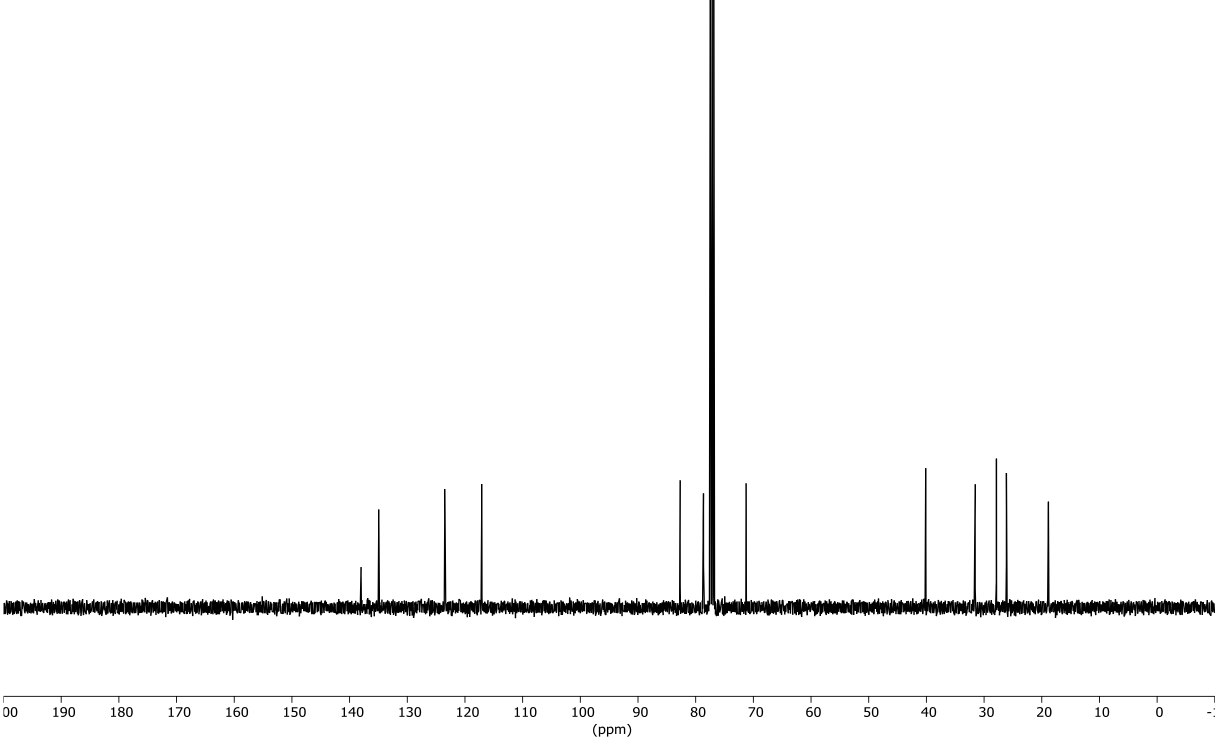

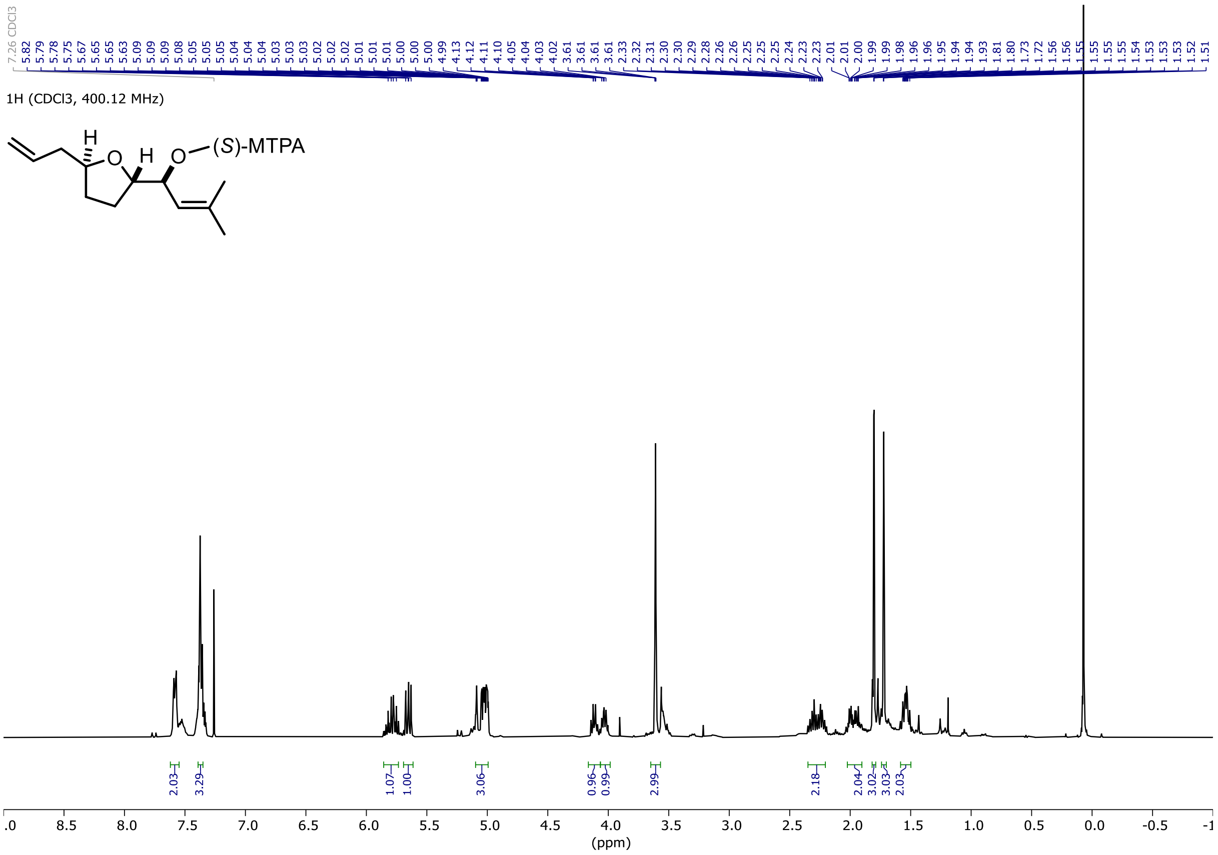
1H (CDCl3, 400.12 MHz)
O—(S)-MTPA
(ppm)

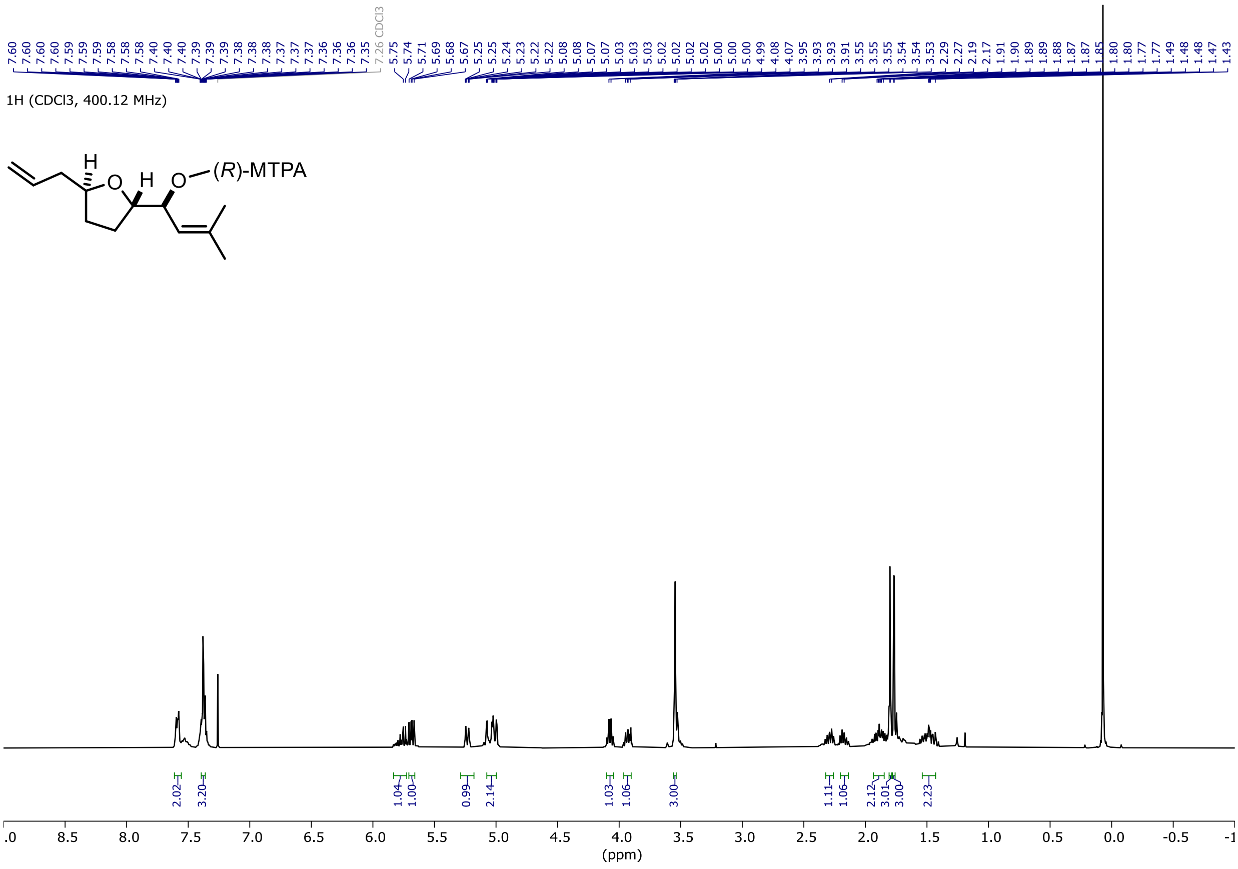
1H (CDCl3, 400.12 MHz)
O—(R)-MTPA
(ppm)

1H (CDCl3, 400.12 MHz)

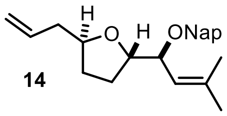

14

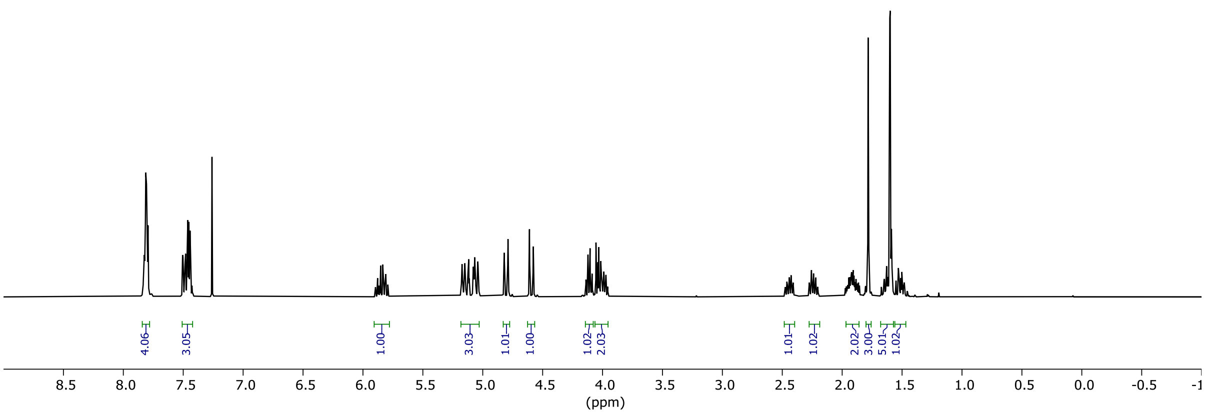

13C (CDCl3, 100.62 MHz)

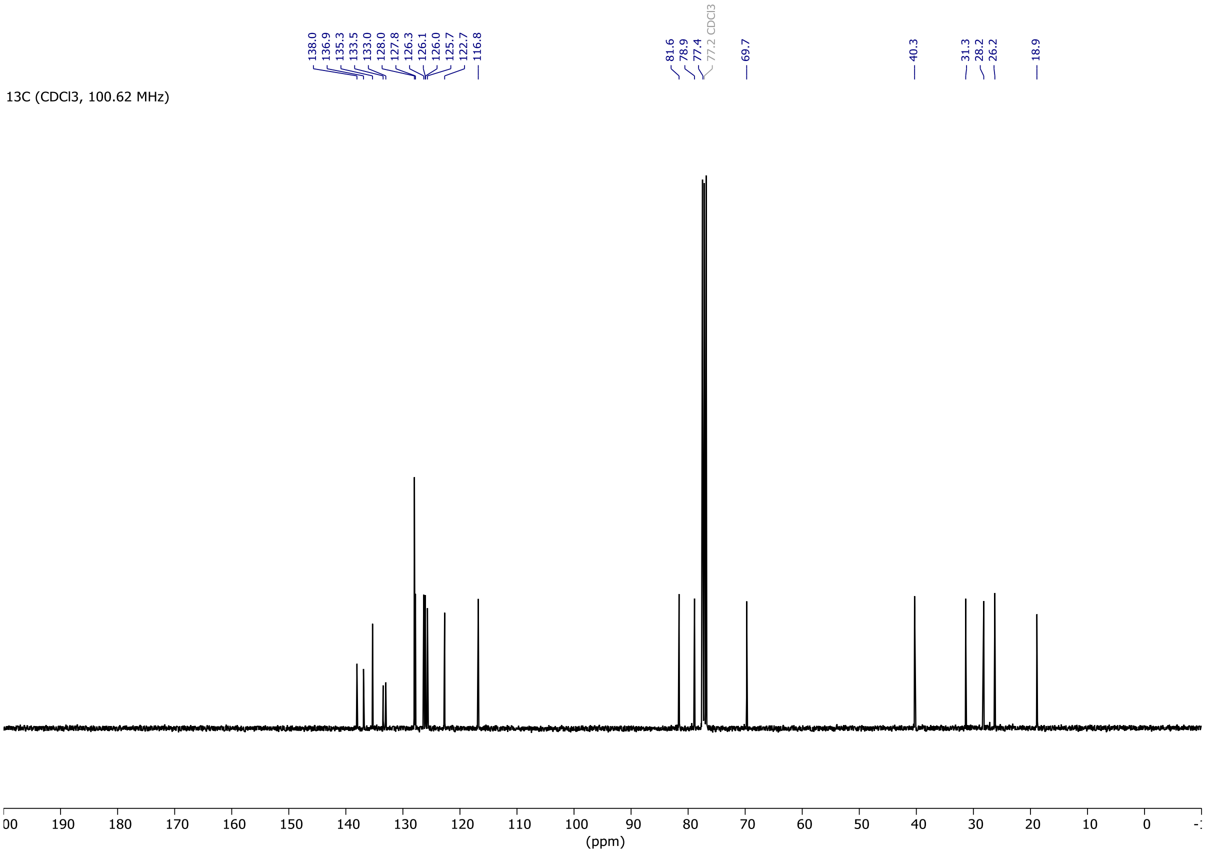

1H (CDCl3, 400.12 MHz)

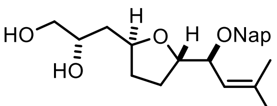

15

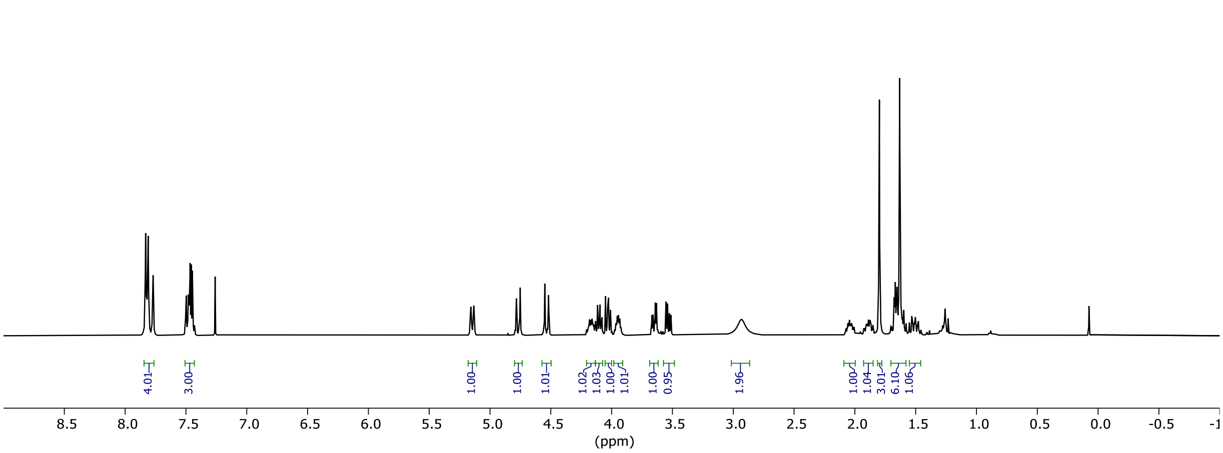

13C (CDCl3, 100.62 MHz)

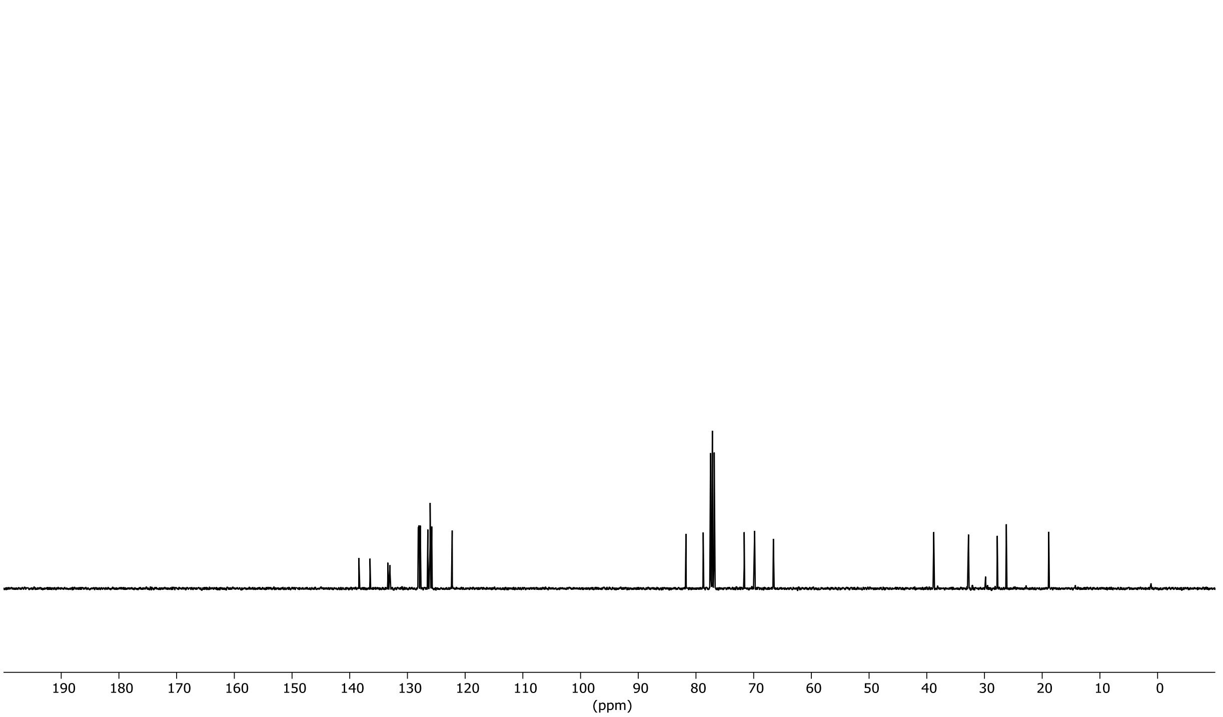

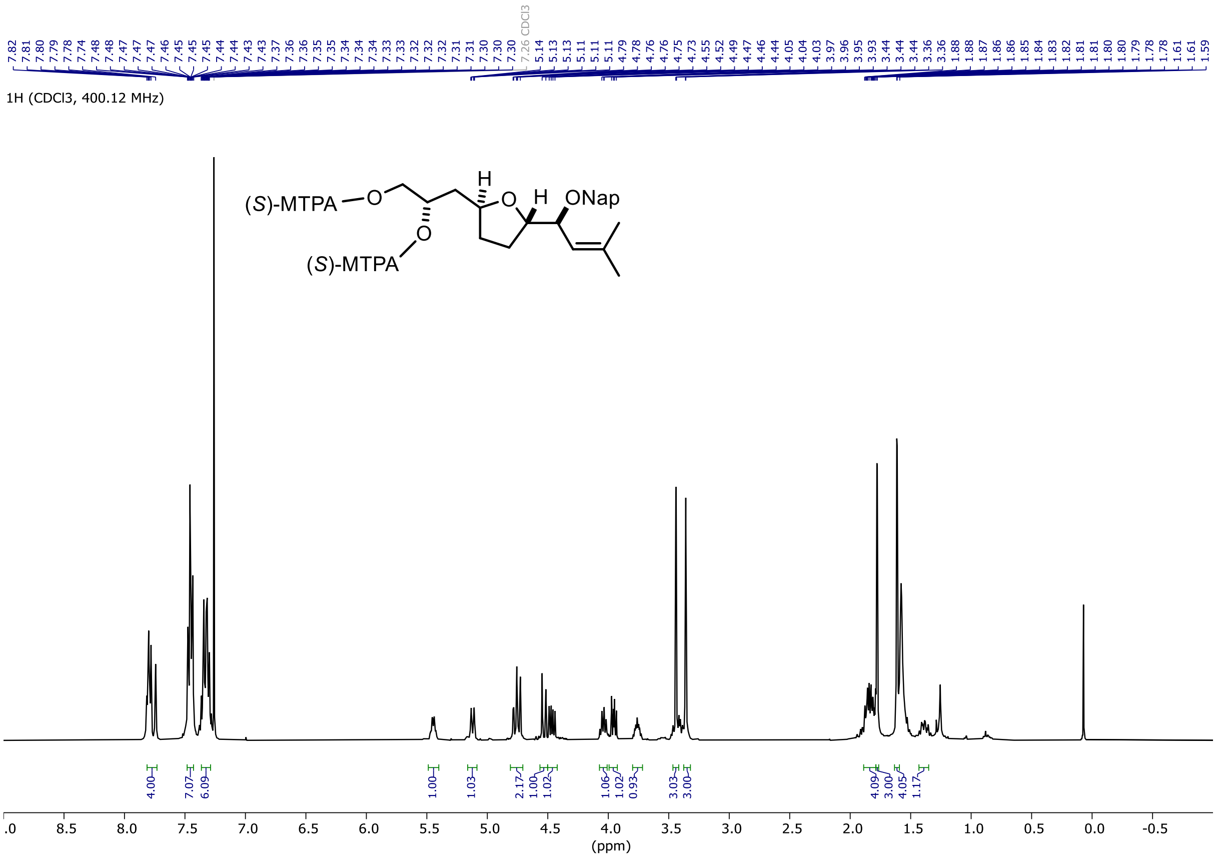
1H (CDCl3, 400.12 MHz)
(S)-MTPA
(S)-MTPA
ONap
(ppm)

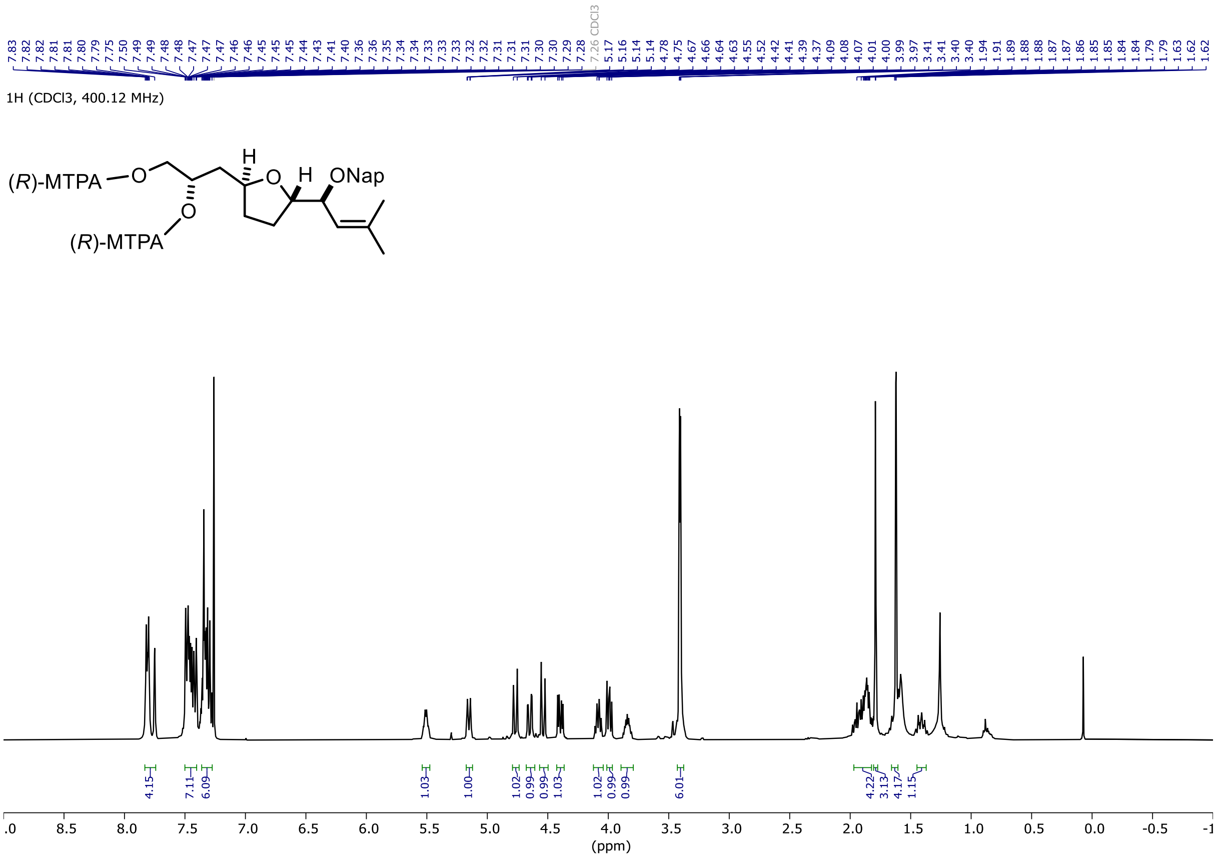
1H (CDCl3, 400.12 MHz)
(R)-MTPA
(R)-MTPA
ONap
(ppm)

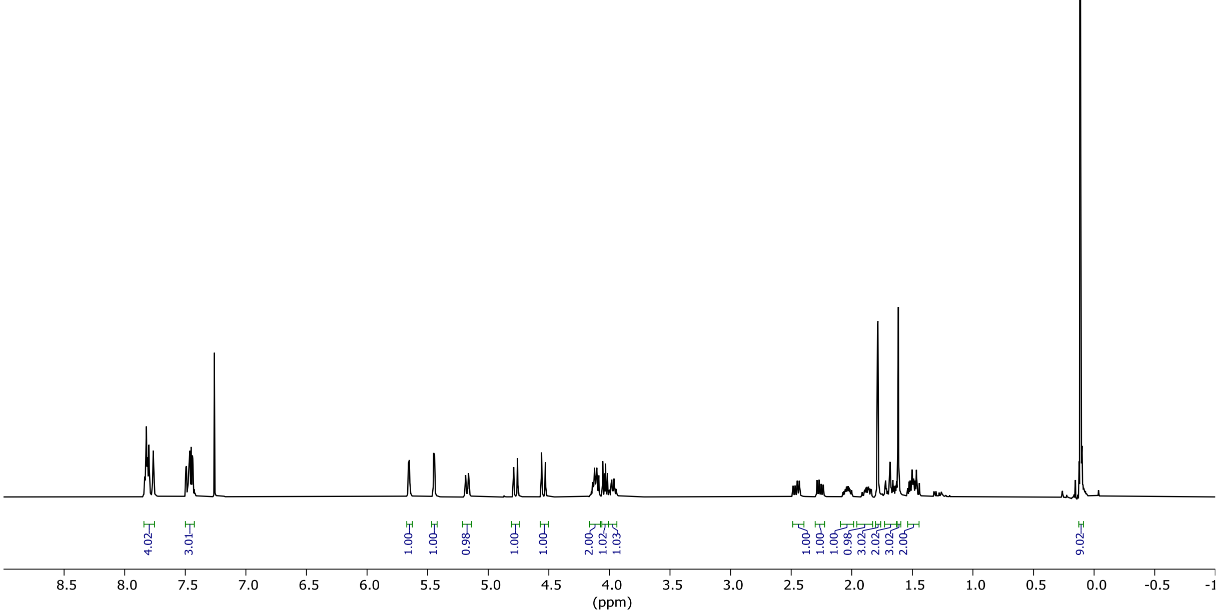
7.83 7.83 7.83 7.82 7.82 7.81 7.81 7.81 7.80 7.77 7.76 7.76 7.49 7.49 7.47 7.46 7.46 7.46 7.45 7.45 7.44 7.44
7.26 CDCl3
5.66 5.66 5.65 5.65 5.45 5.45 5.45 5.44 5.44 5.44 5.19 5.18 5.17 5.16 5.16 4.79 4.76 4.56 4.53 4.12 4.11 4.10 4.09 4.06 4.04 4.03 4.02 3.98 3.96 2.43 2.29 2.27 1.79 1.78 1.72 1.69 1.69 1.68 1.68 1.66 1.63 1.62 1.62 1.52 1.50 1.49 1.49 1.49 1.48 1.47 1.46 0.12 0.11 0.11 0.10 0.10
1H (CDCl3, 400.12 MHz)
4.02 3.01 1.00 1.00 0.98 1.00 1.00 2.00 1.02 1.03 1.00 1.00 1.00 0.98 3.02 2.02 3.02 2.00 9.02
8.5 8.0 7.5 7.0 6.5 6.0 5.5 5.0 4.5 4.0 3.5 3.0 2.5 2.0 1.5 1.0 0.5 0.0 -0.5 -1
(ppm)

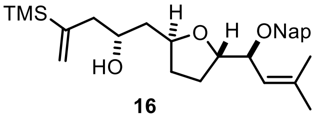
TMS
HO
H
O
H
ONap

16

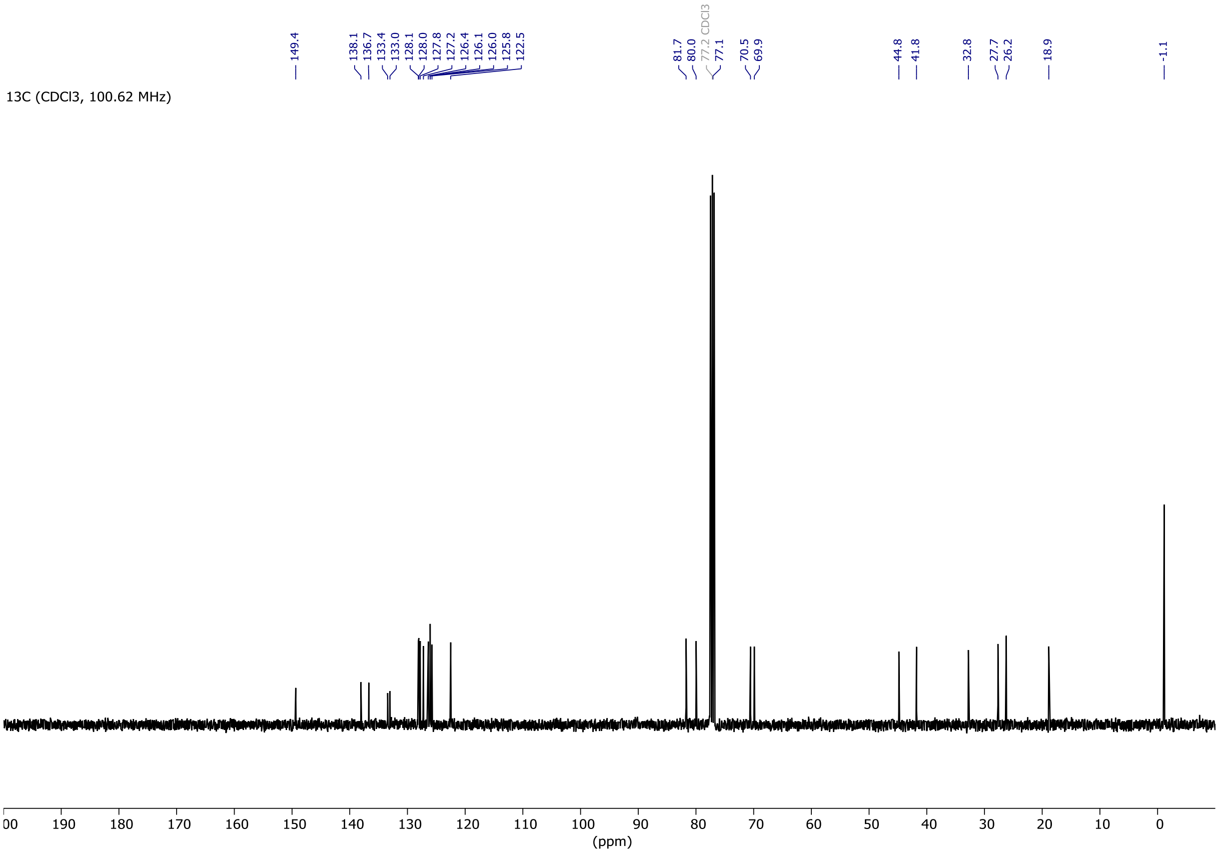
149.4
138.1 136.7 133.4 133.0 128.1 128.0 127.8 127.2 126.4 126.1 126.0 125.8 122.5
81.7 80.0 77.2 CDCl3 77.1 70.5 69.9
44.8 41.8 32.8 27.7 26.2 18.9 -1.1
13C (CDCl3, 100.62 MHz)
00 190 180 170 160 150 140 130 120 110 100 90 80 70 60 50 40 30 20 10 0
(ppm)

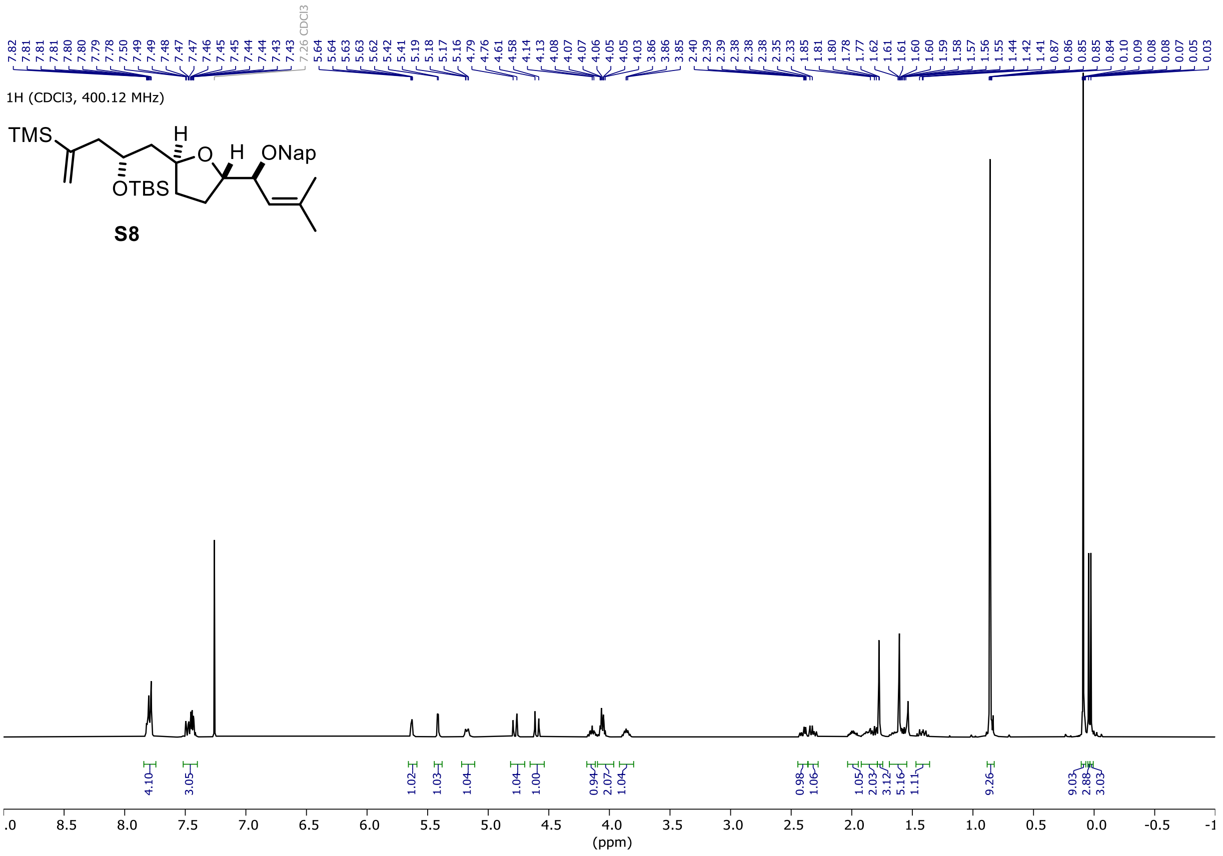
1H (CDCl3, 400.12 MHz)
TMS
OTBS
ONap
S8
(ppm)

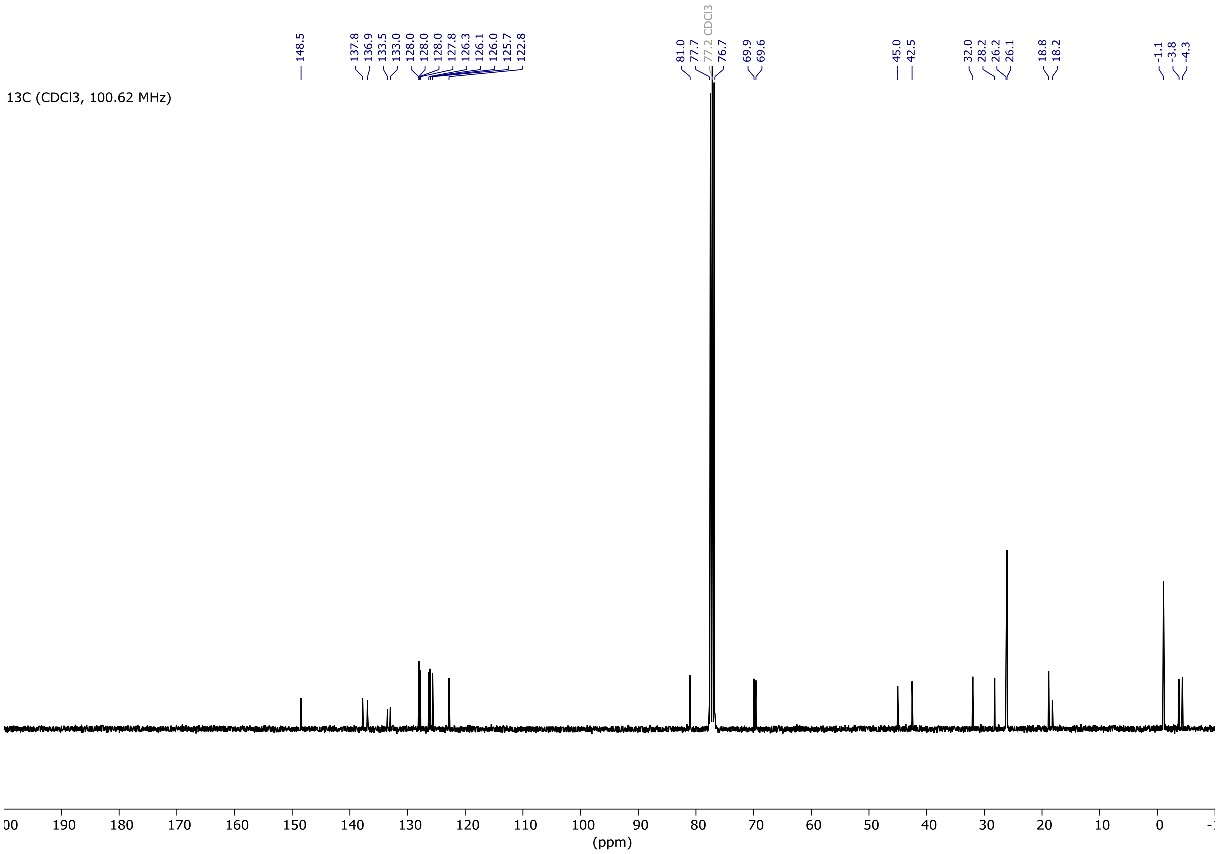
13C (CDCl3, 100.62 MHz)
(ppm)

1H (CDCl3, 400.12 MHz)

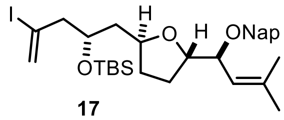

17

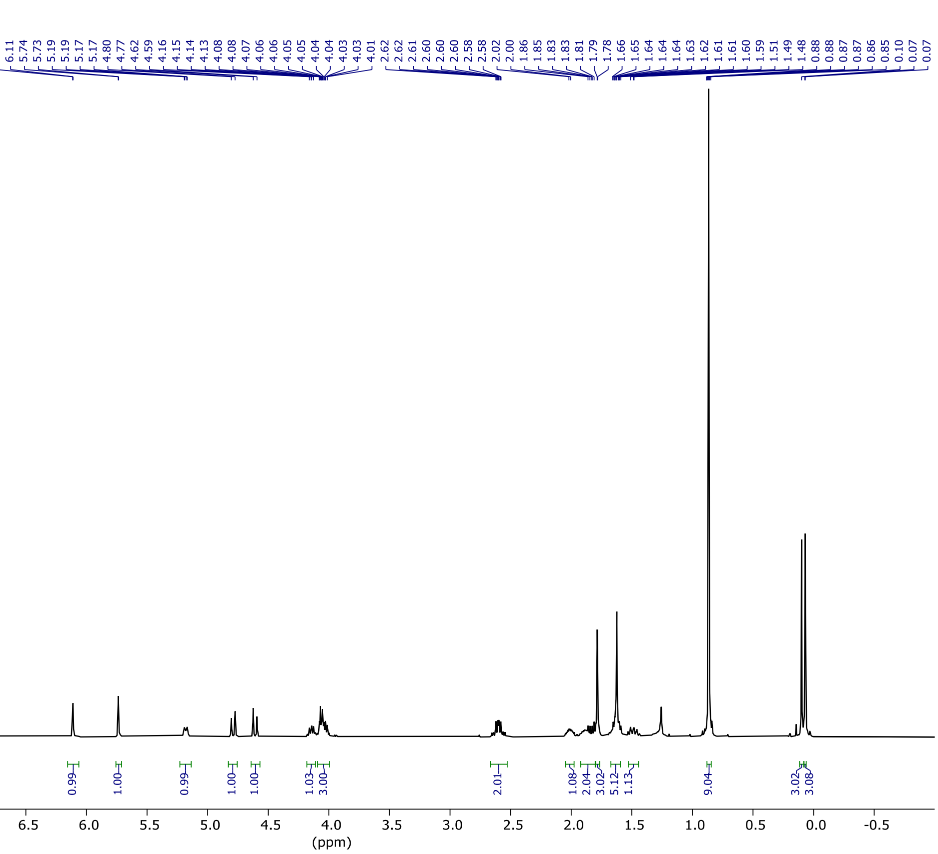

13C (CDCl3, 100.62 MHz)

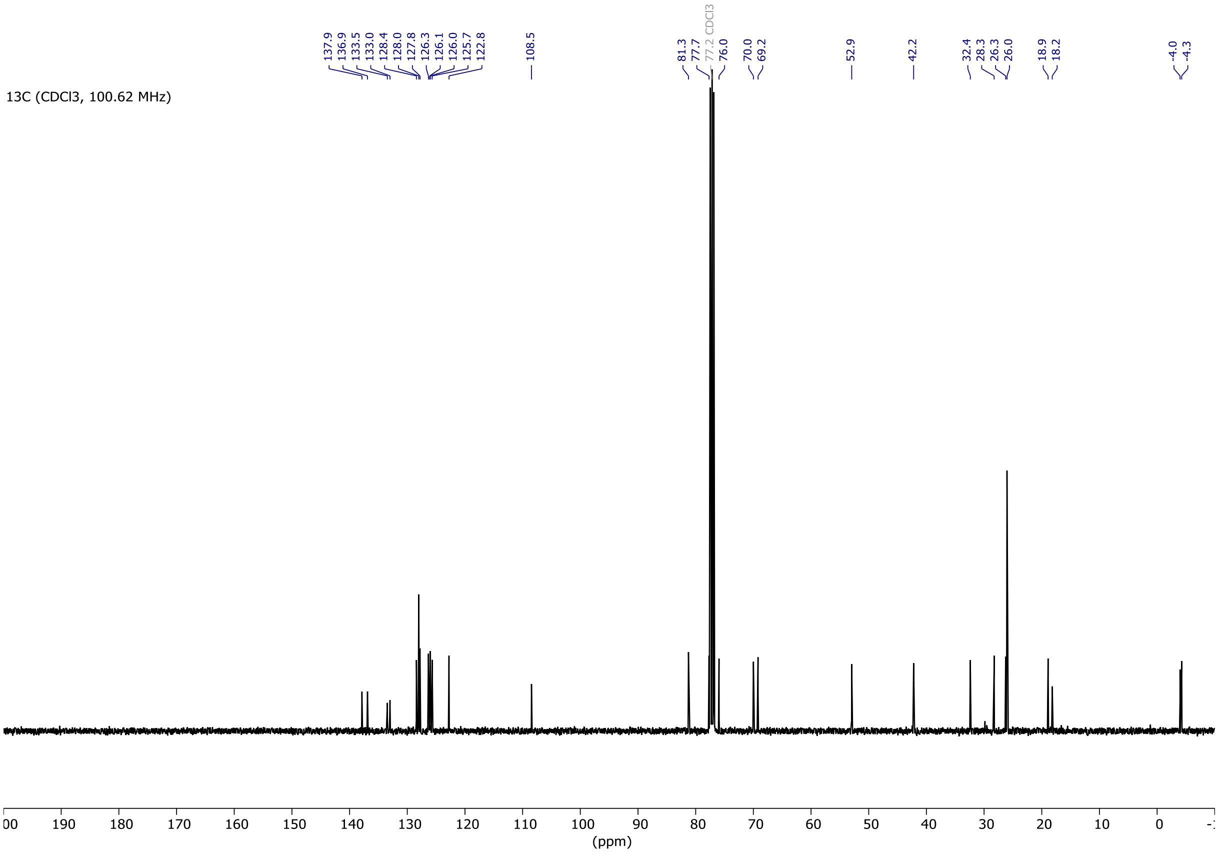

1H (CDCl3, 400.12 MHz)

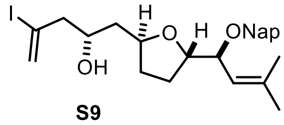

S9

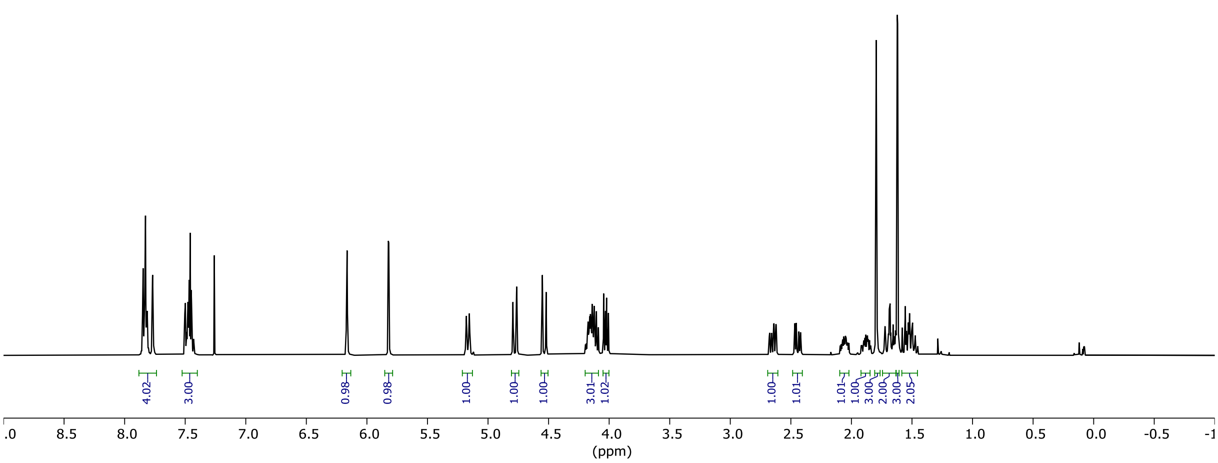

13C (CDCl3, 100.62 MHz)

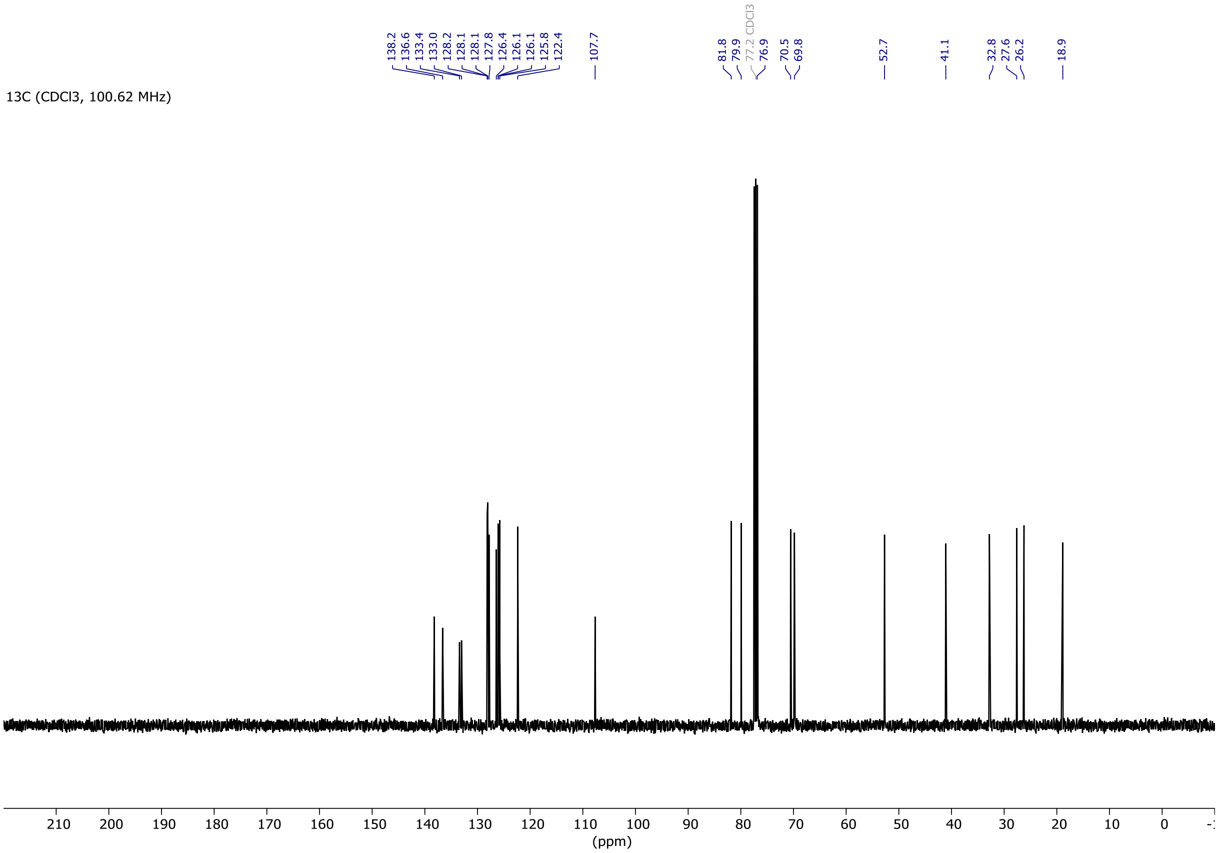

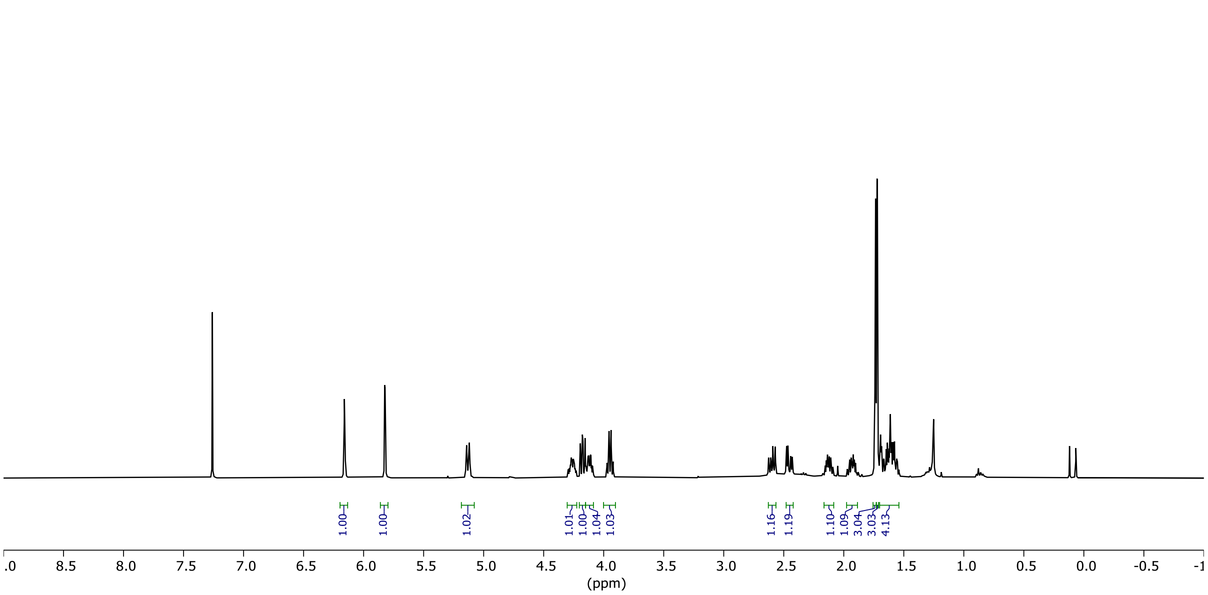
7.26 CDCl3
6.16 6.16 6.16 6.15 5.82 5.82 5.15 5.14 5.14 5.12 5.12 5.12 4.27 4.27 4.26 4.26 4.25 4.19 4.18 4.17 4.16 4.13 4.12 4.12 4.12 4.11 4.11 3.97 3.96 3.94 3.92 2.63 2.62 2.61 2.61 2.59 2.59 2.57 2.57 2.48 2.48 2.47 2.46 2.44 2.44 2.43 2.43 2.15 2.14 2.14 2.12 2.12 2.11 1.95 1.94 1.92 1.92 1.91 1.90 1.74 1.73 1.72 1.72 1.70 1.69 1.69 1.69 1.67 1.65 1.64 1.63 1.63 1.62 1.61 1.61 1.60 1.59 1.59 1.58 1.58 1.56 1.55
1H (CDCl3, 400.12 MHz)
1.00 1.00 1.02 1.01 1.00 1.04 1.03 1.16 1.19 1.10 1.09 3.04 3.03 4.13
(ppm)

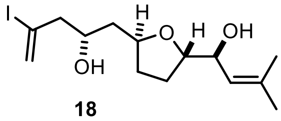
I
H
O
H
OH
OH

18

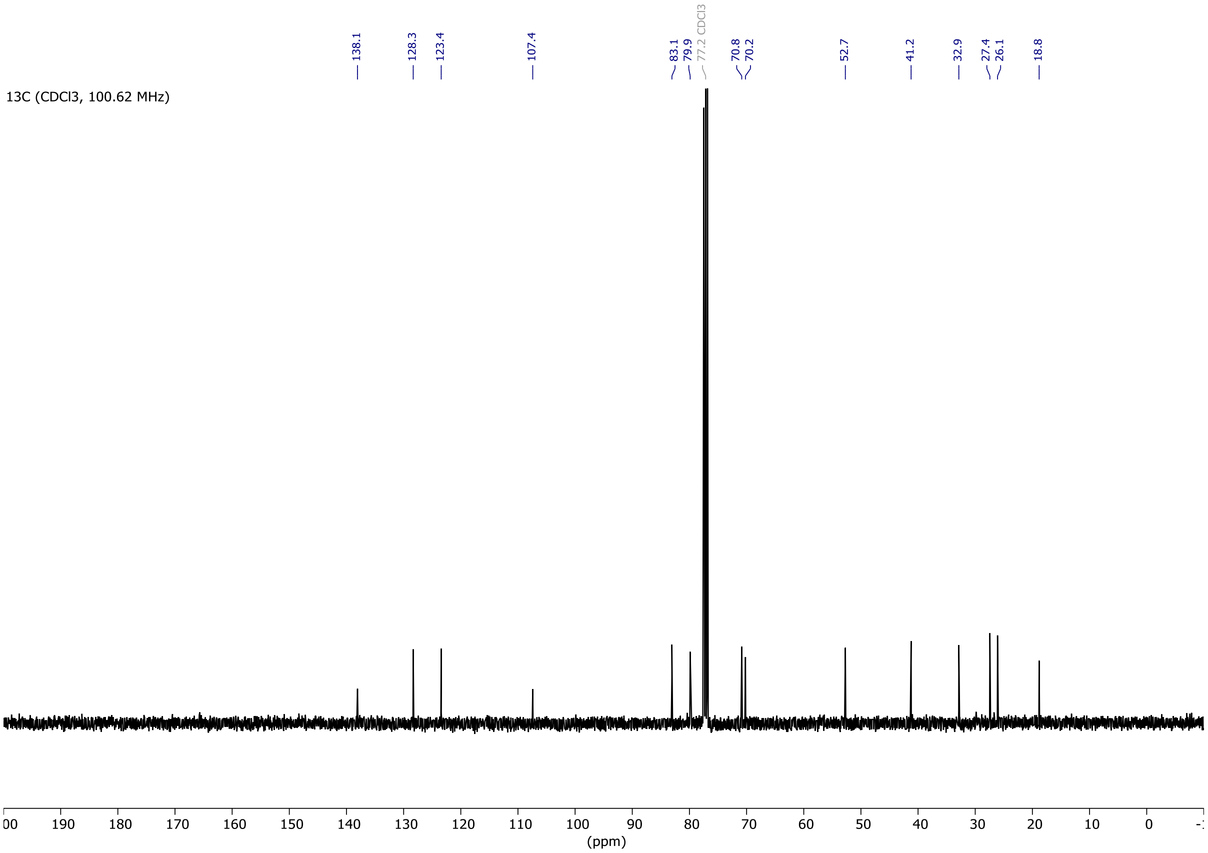
138.1 128.3 123.4 107.4 83.1 79.9 77.2 CDCl3 70.8 70.2 52.7 41.2 32.9 27.4 26.1 18.8
13C (CDCl3, 100.62 MHz)
(ppm)

1H (CDCl3, 400.12 MHz)

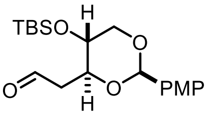

S11

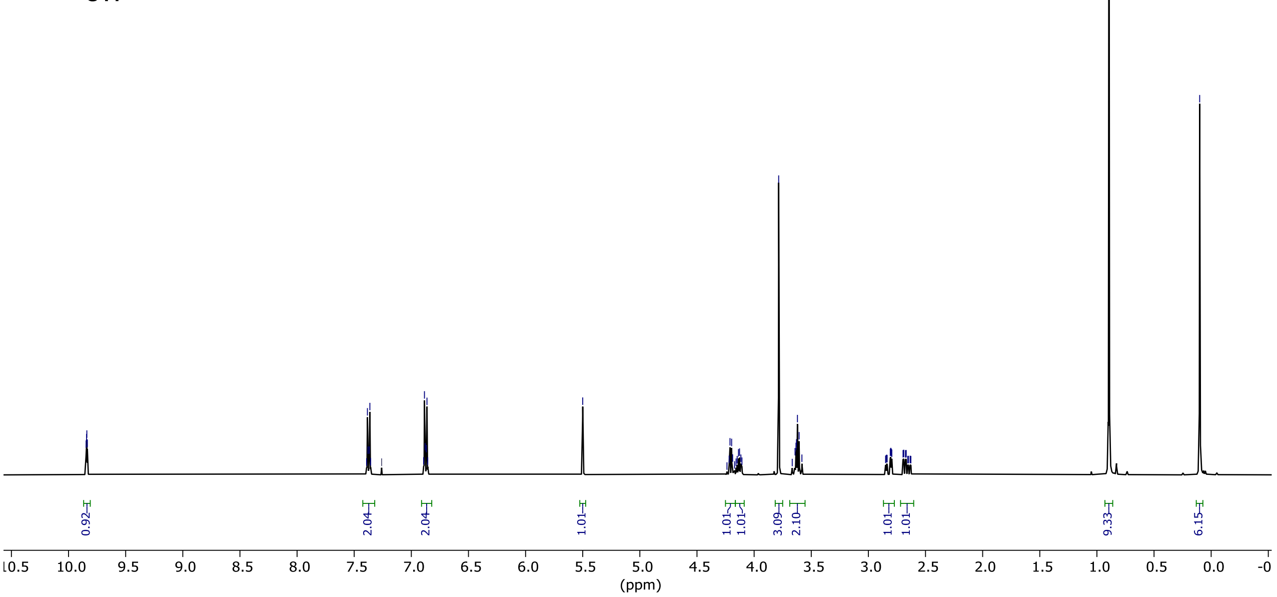

13C (CDCl3, 100.62 MHz)

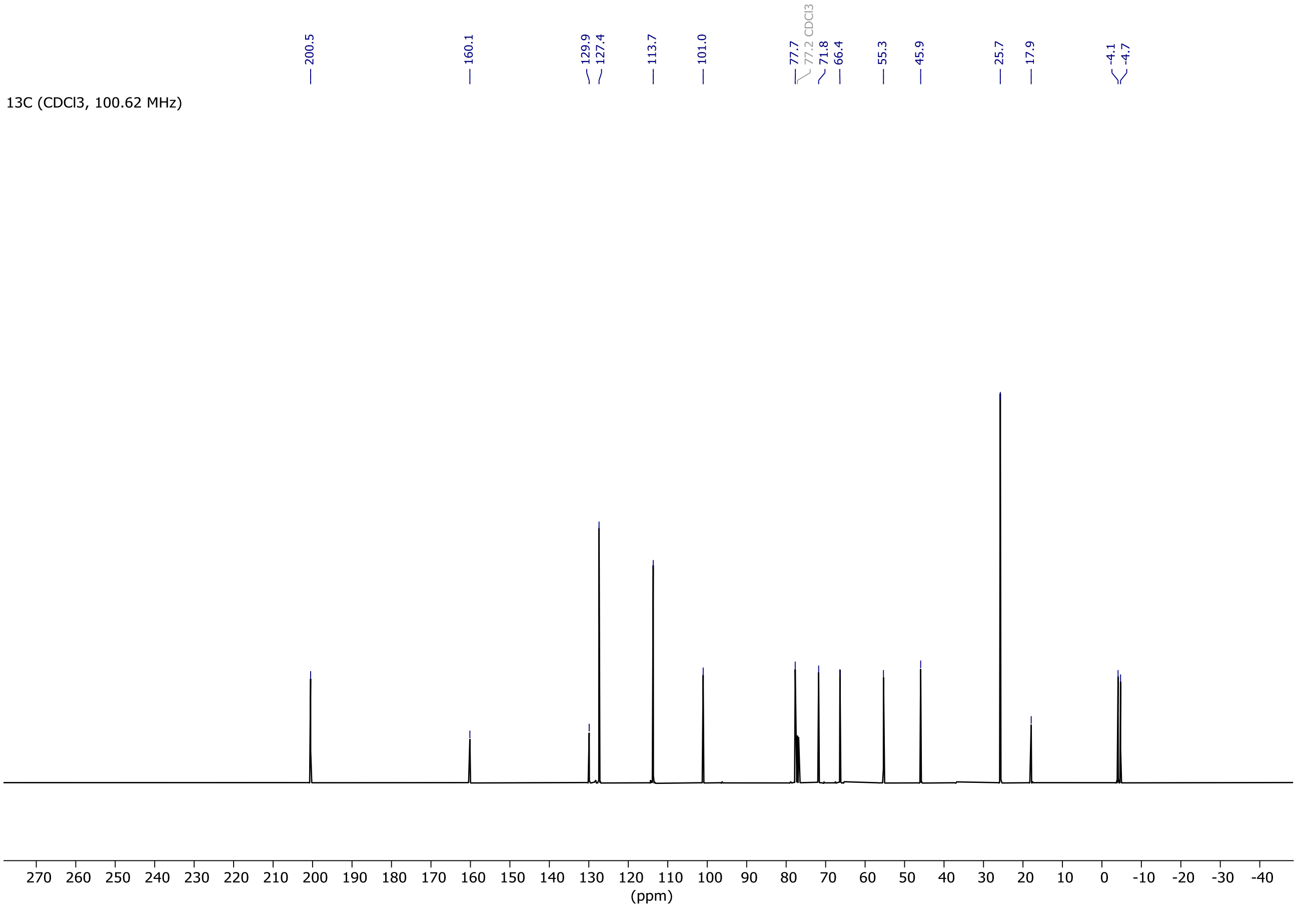

1H (MeOD, 400.12 MHz)

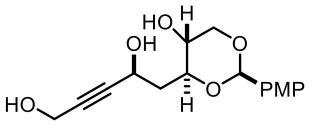

26

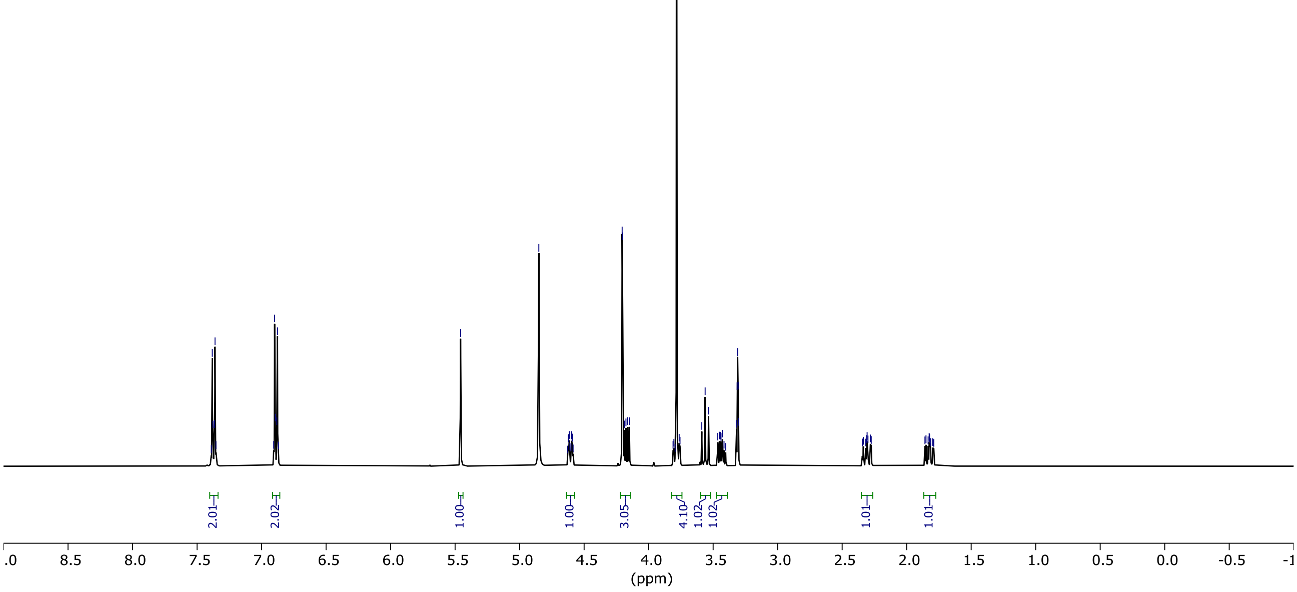

13C (MeOD, 100.62 MHz)

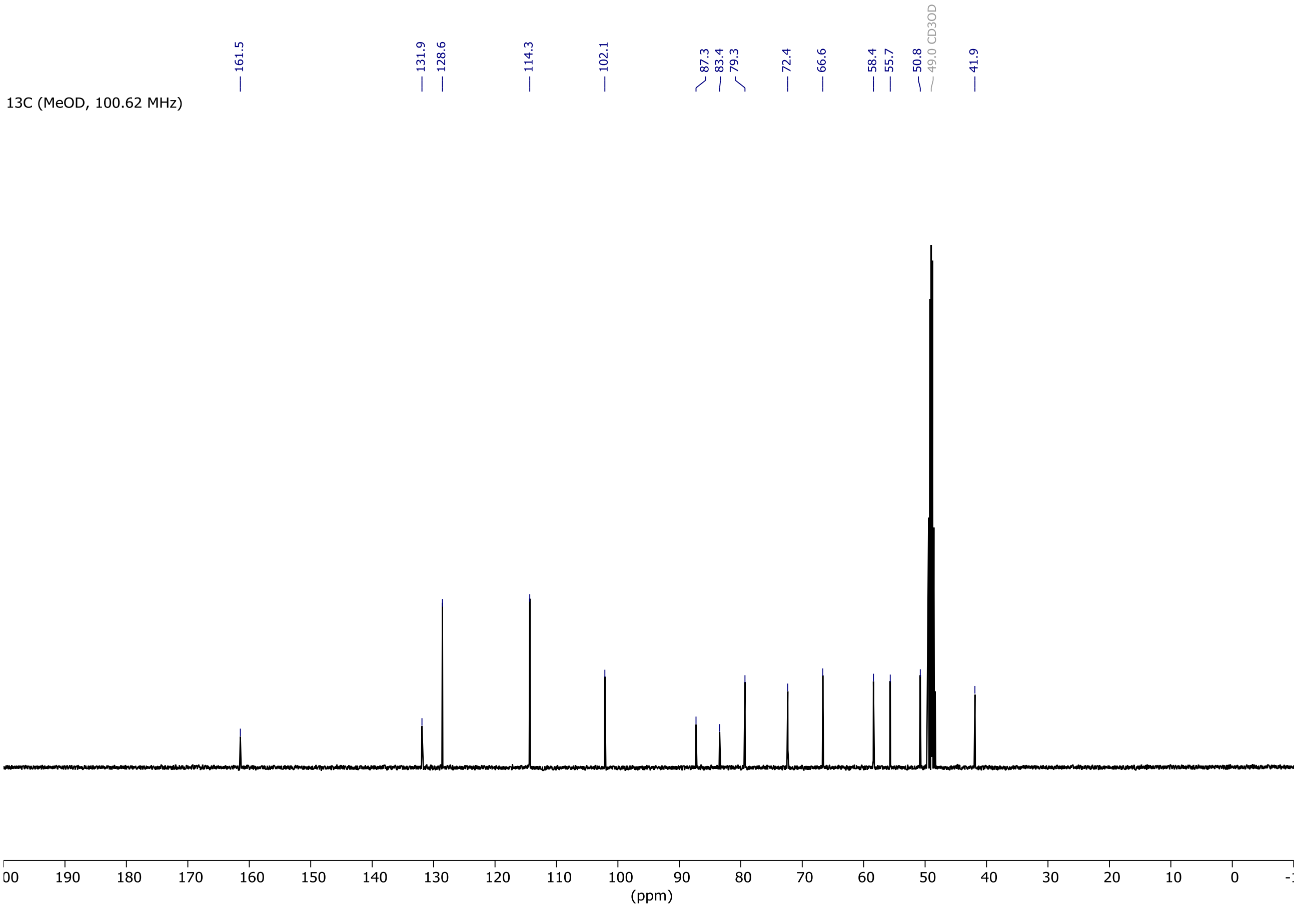

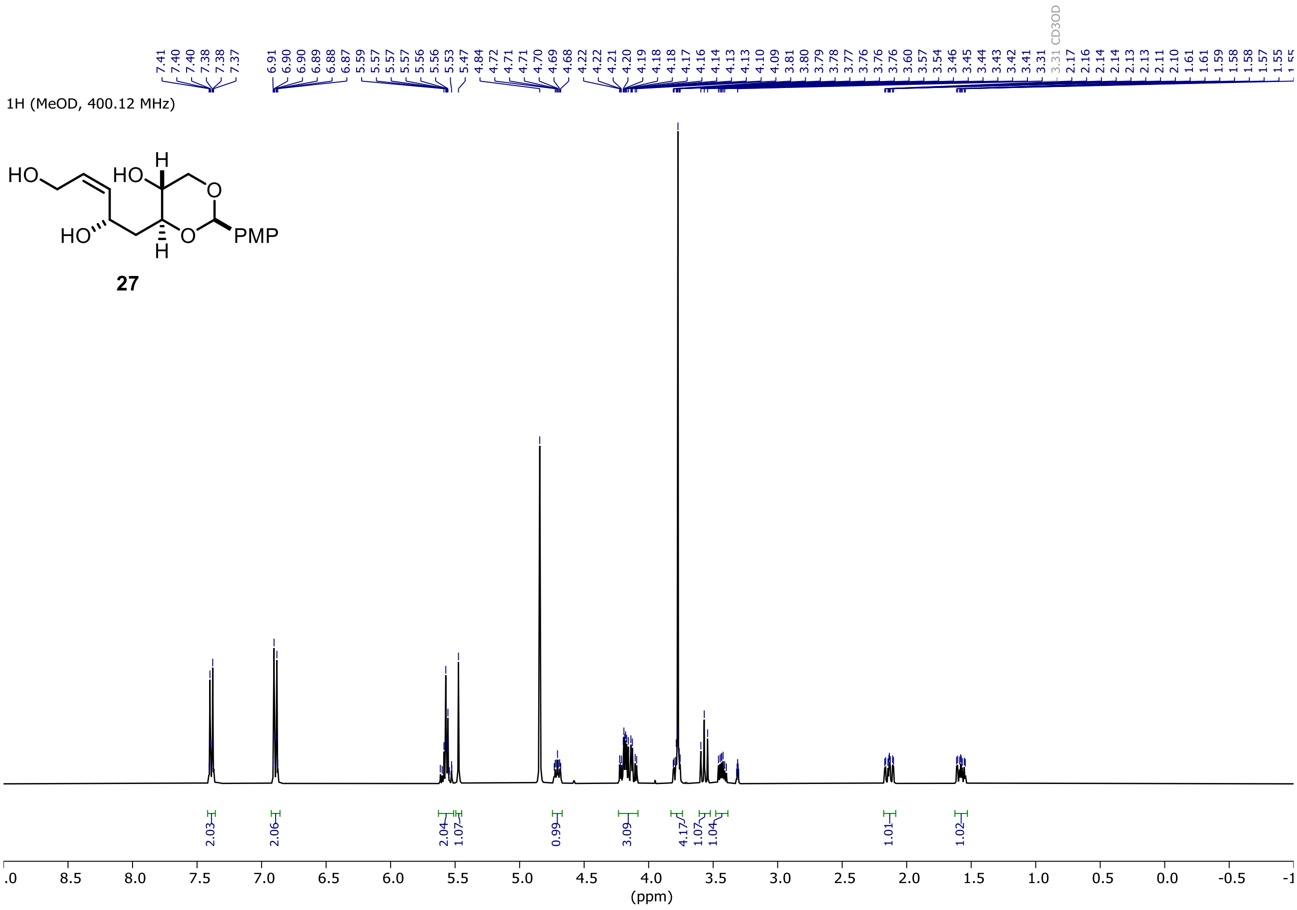
1H (MeOD, 400.12 MHz)
7.41 7.40 7.40 7.38 7.38 7.37 6.91 6.90 6.90 6.89 6.88 6.87 5.59 5.57 5.57 5.57 5.56 5.56 5.53 5.47 4.84 4.72 4.71 4.71 4.70 4.69 4.68 4.22 4.22 4.21 4.20 4.19 4.18 4.18 4.17 4.16 4.14 4.13 4.13 4.10 4.09 3.81 3.80 3.79 3.78 3.77 3.76 3.76 3.60 3.57 3.54 3.46 3.45 3.44 3.43 3.42 3.41 3.31 3.31 CD3OD 2.17 2.16 2.14 2.14 2.13 2.13 2.11 2.10 1.61 1.61 1.59 1.58 1.58 1.57 1.55
HO
HO
HO
H
H
O
O
PMP
27
2.03
2.06
2.04
1.07
0.99
3.09
4.17
1.07
1.04
1.01
1.02
(ppm)

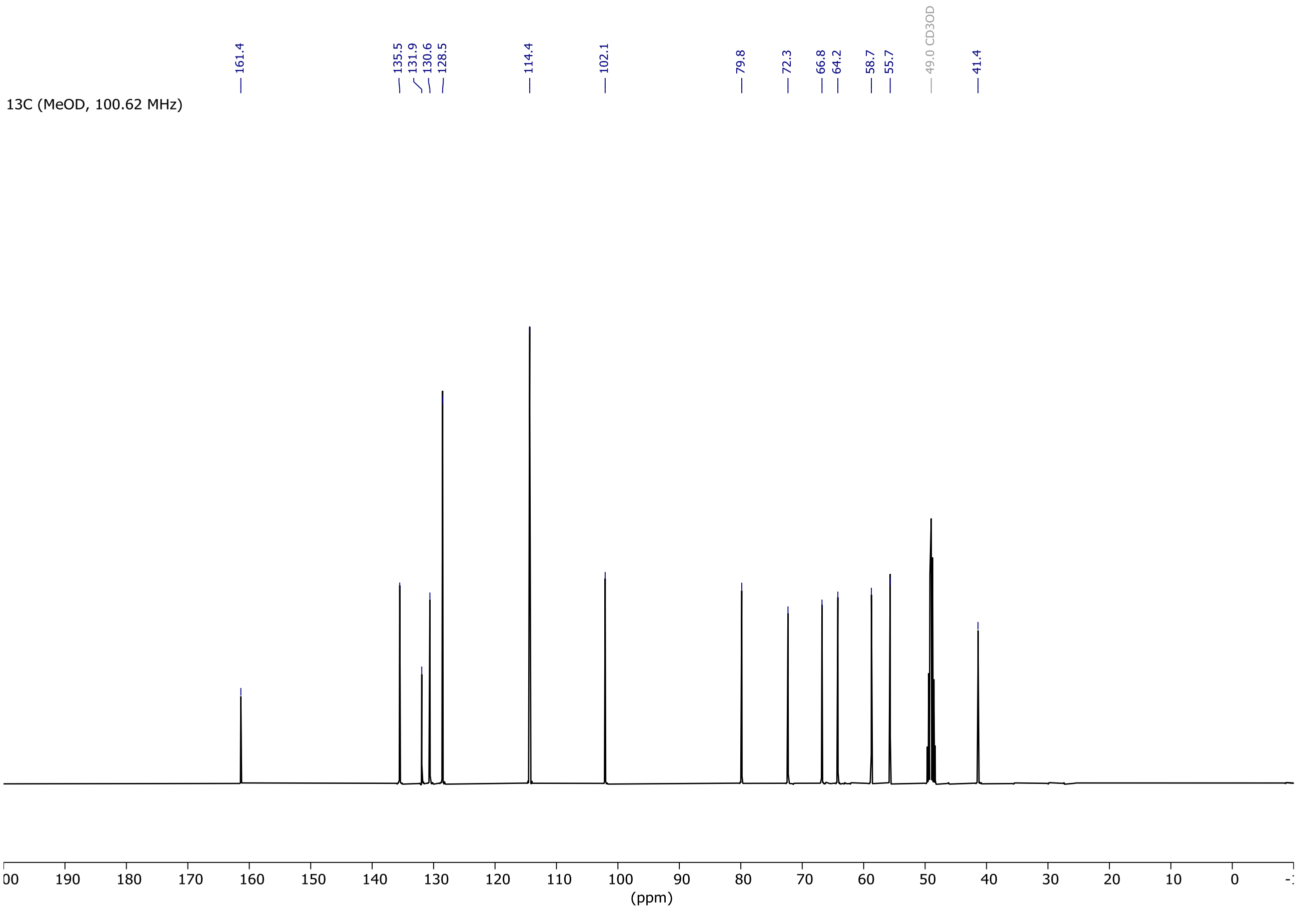
13C (MeOD, 100.62 MHz)
161.4
135.5 131.9 130.6 128.5
114.4
102.1
79.8
72.3
66.8 64.2
58.7 55.7
49.0 CD3OD
41.4
(ppm)

1H (MeOD, 400.12 MHz)

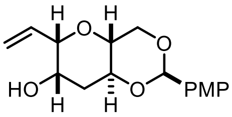

28

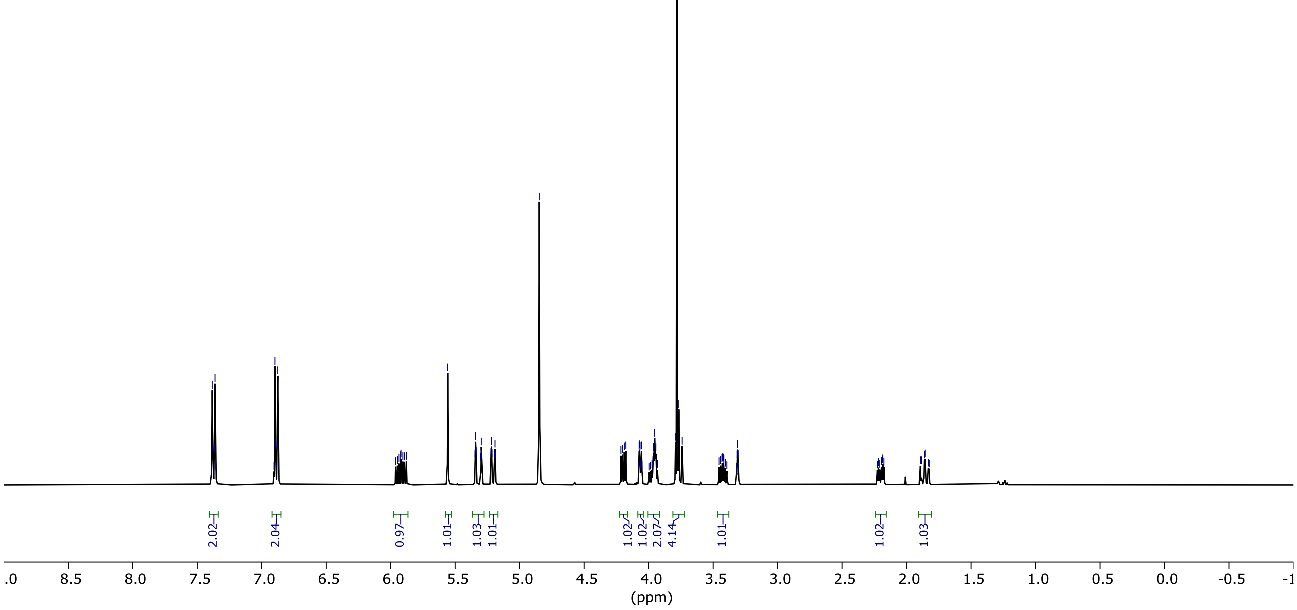

13C (MeOD, 100.62 MHz)

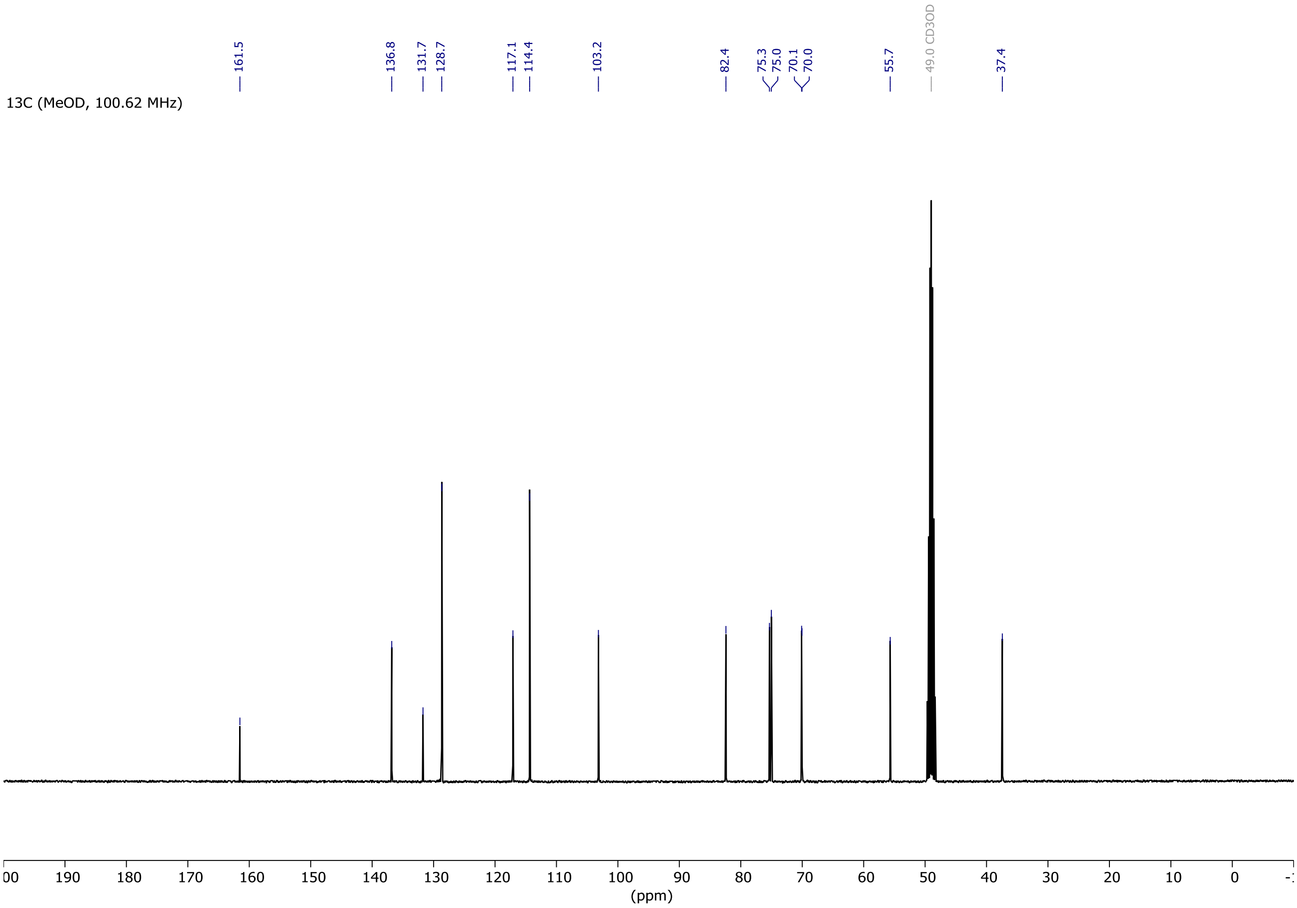

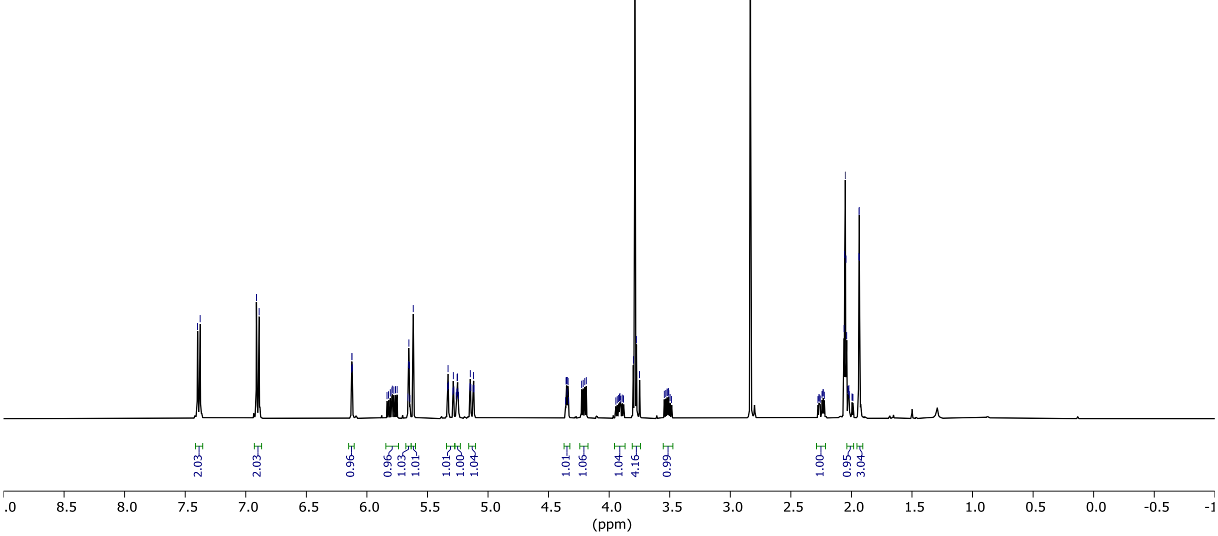

1H (Acetone, 400.12 MHz)

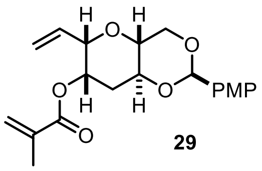

29

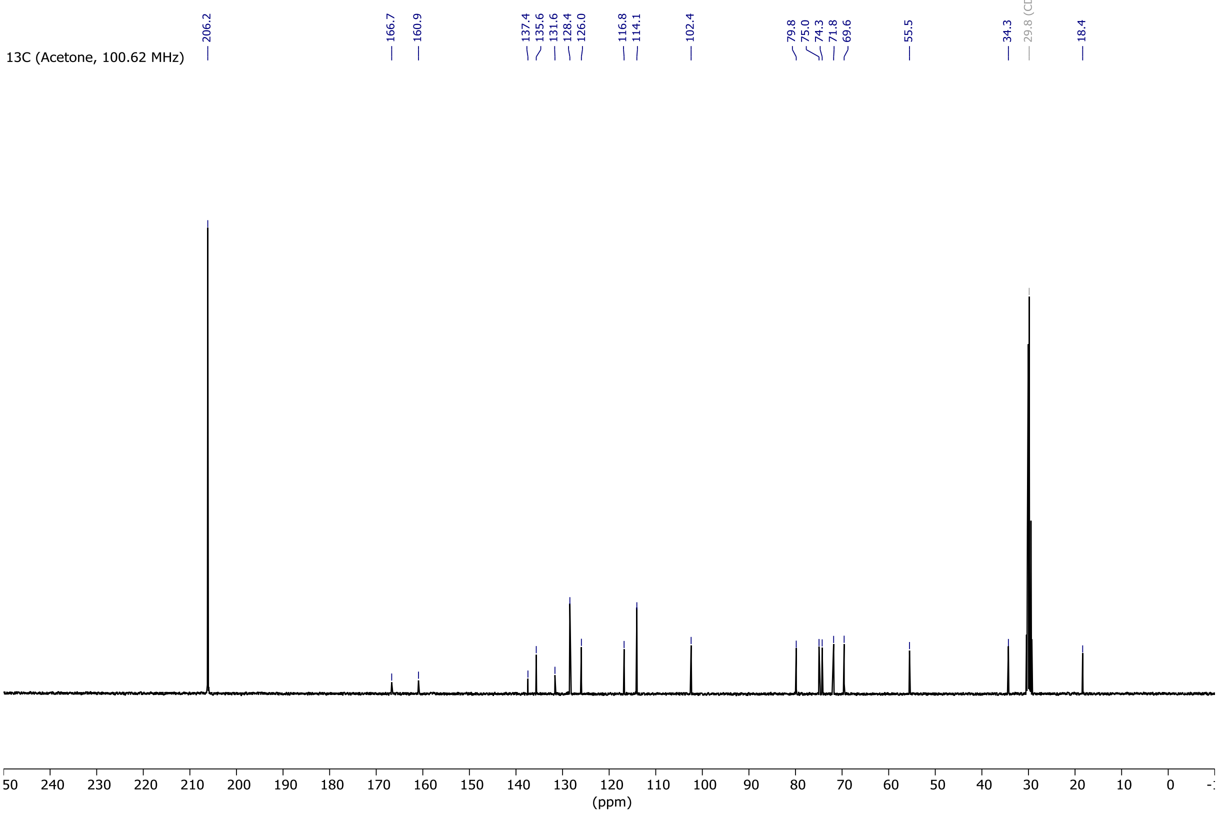

13C (Acetone, 100.62 MHz)

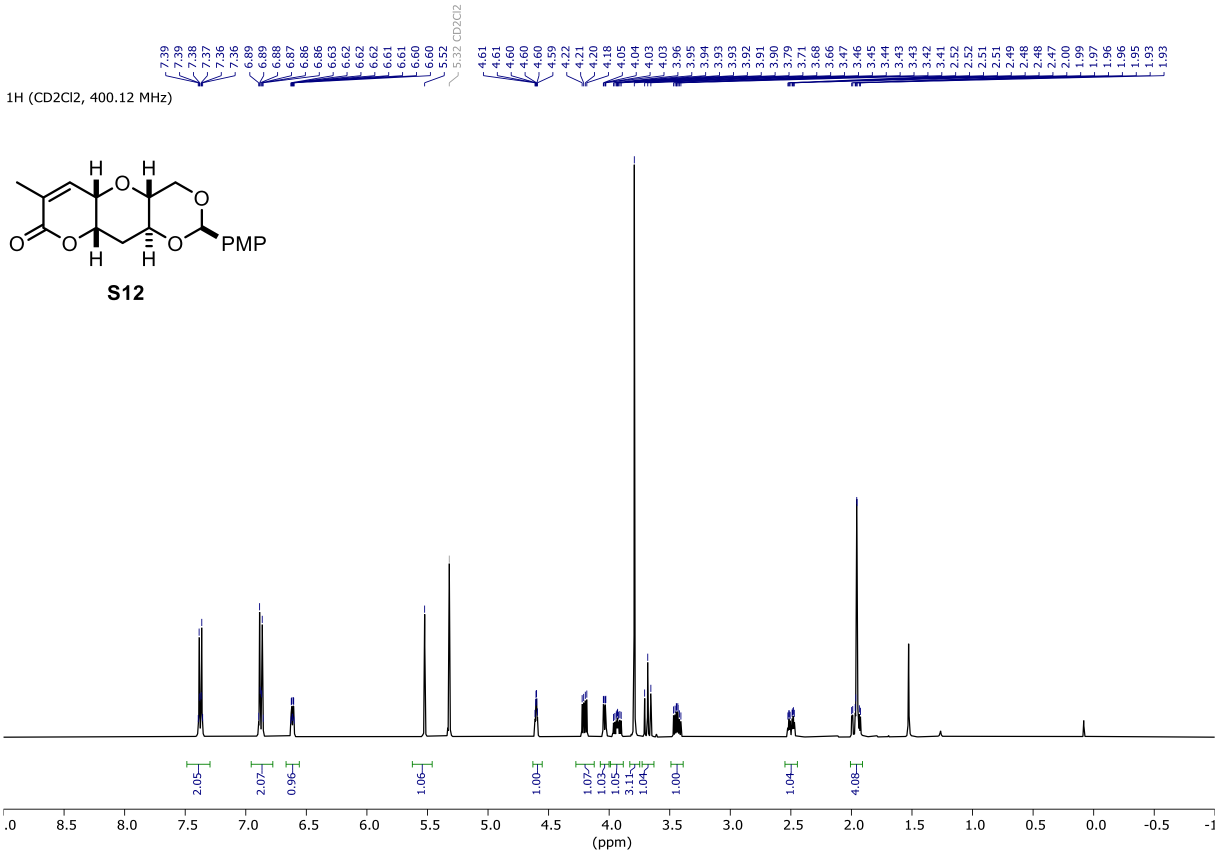

7.39 7.39 7.38 7.37 7.36 7.36 6.89 6.88 6.87 6.86 6.86 6.63 6.62 6.62 6.62 6.61 6.61 6.60 6.60 5.52 5.32 CD2Cl2 4.61 4.61 4.60 4.60 4.60 4.59 4.22 4.21 4.20 4.18 4.05 4.04 4.03 4.03 3.96 3.95 3.94 3.93 3.93 3.92 3.91 3.90 3.79 3.71 3.68 3.66 3.47 3.46 3.45 3.44 3.43 3.43 3.42 3.41 2.52 2.52 2.51 2.51 2.49 2.48 2.48 2.47 2.00 1.99 1.97 1.96 1.96 1.95 1.93 1.93
1H (CD2Cl2, 400.12 MHz)
PMP
S12
2.05 2.07 0.96 1.06 1.00 1.07 1.03 1.05 3.11 1.04 1.00 1.04 4.08
(ppm)

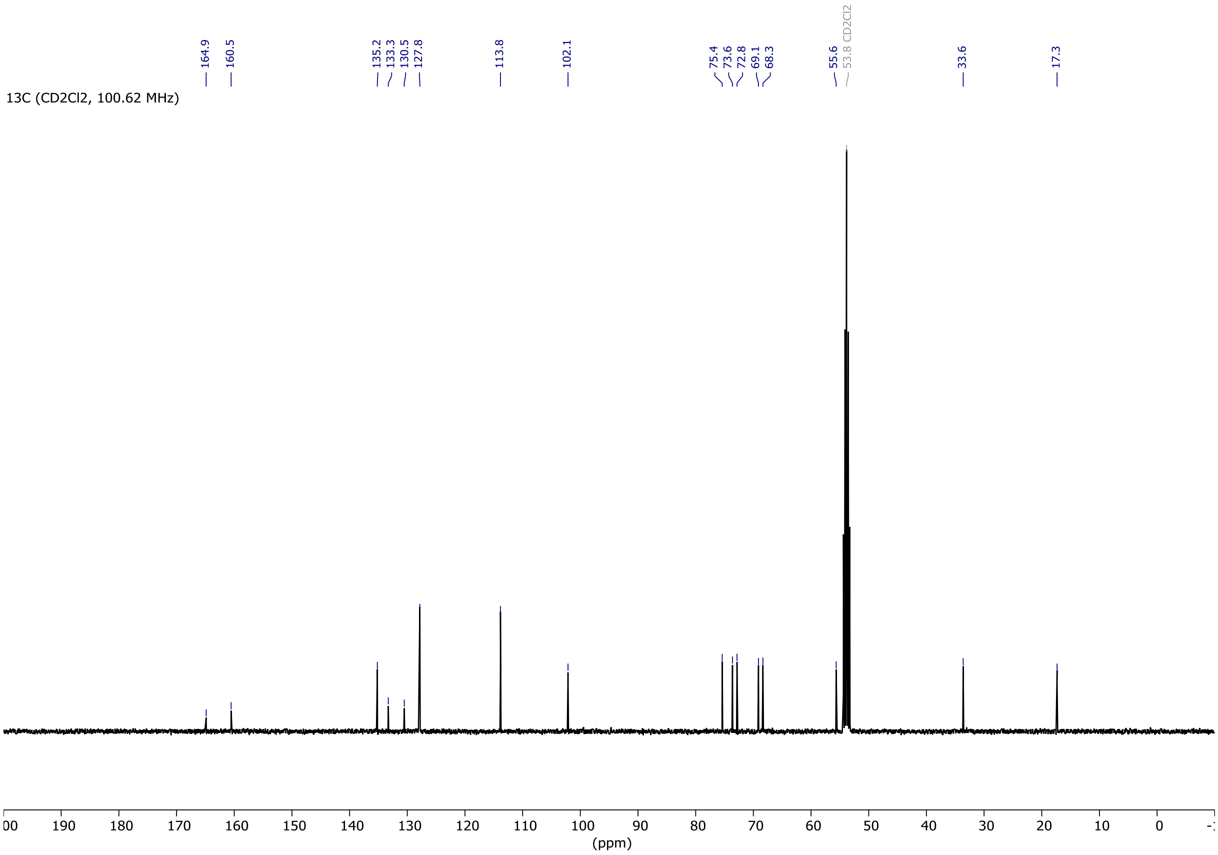

164.9 160.5 135.2 133.3 130.5 127.8 113.8 102.1 75.4 73.6 72.8 69.1 68.3 55.6 53.8 CD2Cl2 33.6 17.3
13C (CD2Cl2, 100.62 MHz)
(ppm)

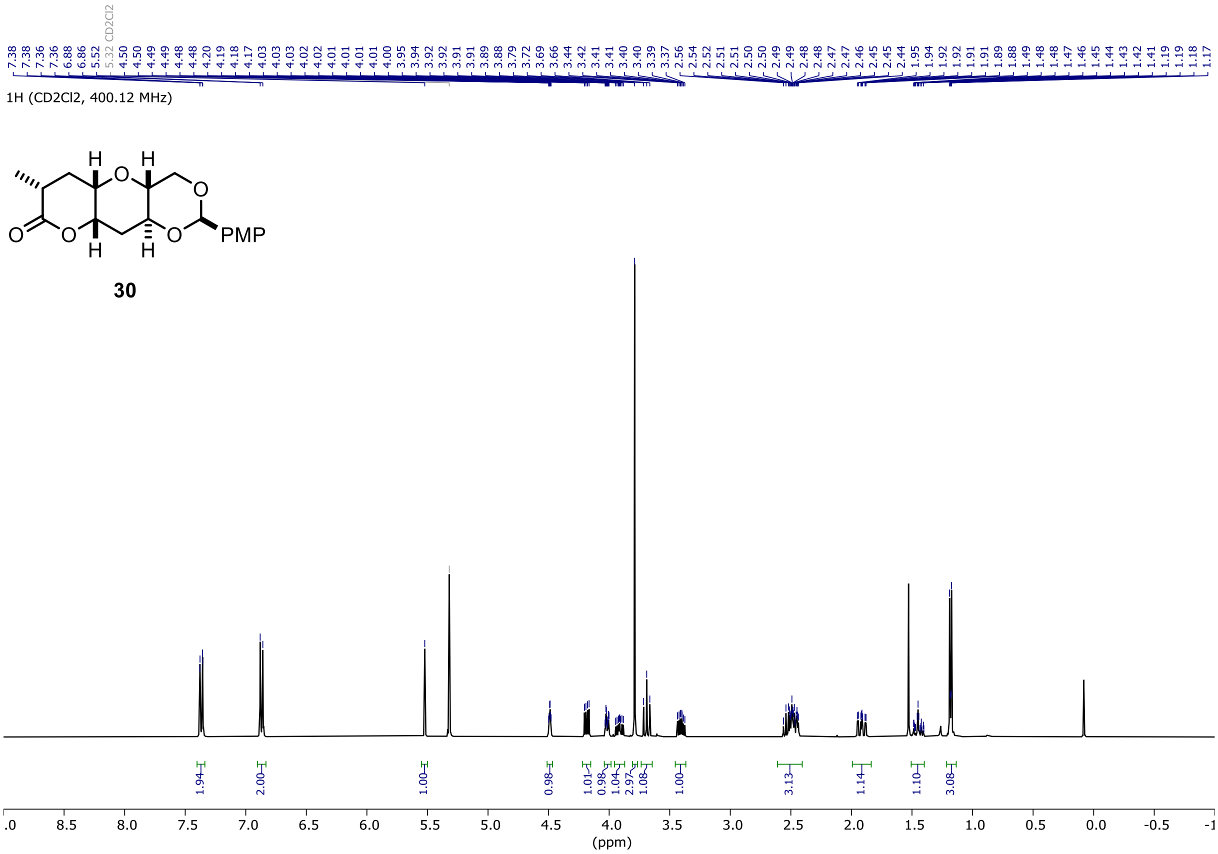
7.38 7.38 7.36 7.36 6.88 6.86 5.52 5.32 CD2Cl2 4.50 4.50 4.49 4.49 4.48 4.48 4.20 4.19 4.18 4.17 4.03 4.03 4.03 4.02 4.02 4.01 4.01 4.01 4.00 3.95 3.94 3.92 3.92 3.91 3.91 3.89 3.88 3.79 3.72 3.69 3.66 3.44 3.42 3.41 3.41 3.40 3.40 3.39 3.37 2.56 2.54 2.52 2.51 2.51 2.50 2.49 2.49 2.48 2.48 2.47 2.47 2.46 2.45 2.45 2.44 1.95 1.94 1.92 1.92 1.91 1.91 1.89 1.88 1.49 1.48 1.48 1.47 1.46 1.45 1.44 1.43 1.42 1.41 1.19 1.19 1.18 1.17
1H (CD2Cl2, 400.12 MHz)
1.94 2.00 1.00 0.98 1.01 0.98 1.04 2.97 1.08 1.00 3.13 1.14 1.10 3.08
(ppm)
H
O
H
O
O
O
O
H
H
PMP

30

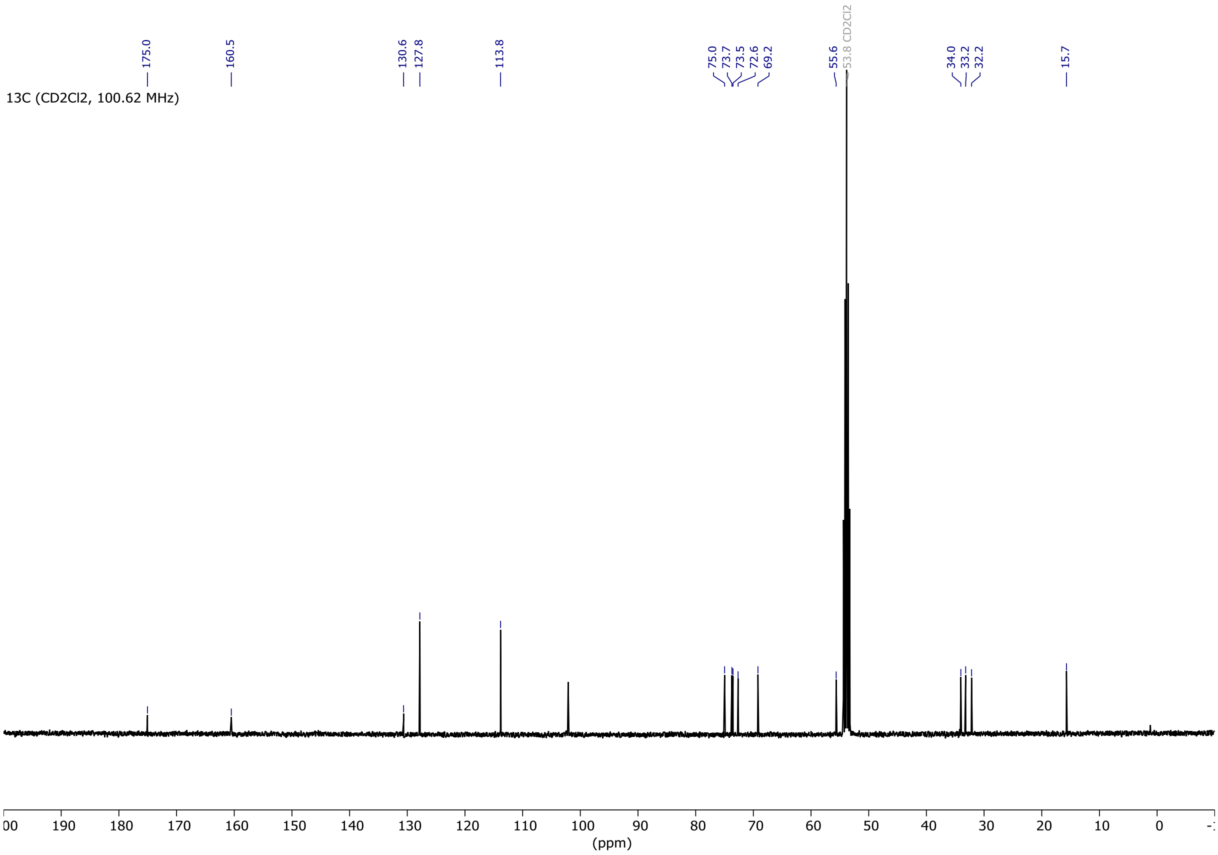
175.0 160.5 130.6 127.8 113.8 75.0 73.7 73.5 72.6 69.2 55.6 53.8 CD2Cl2 34.0 33.2 32.2 15.7
13C (CD2Cl2, 100.62 MHz)
(ppm)

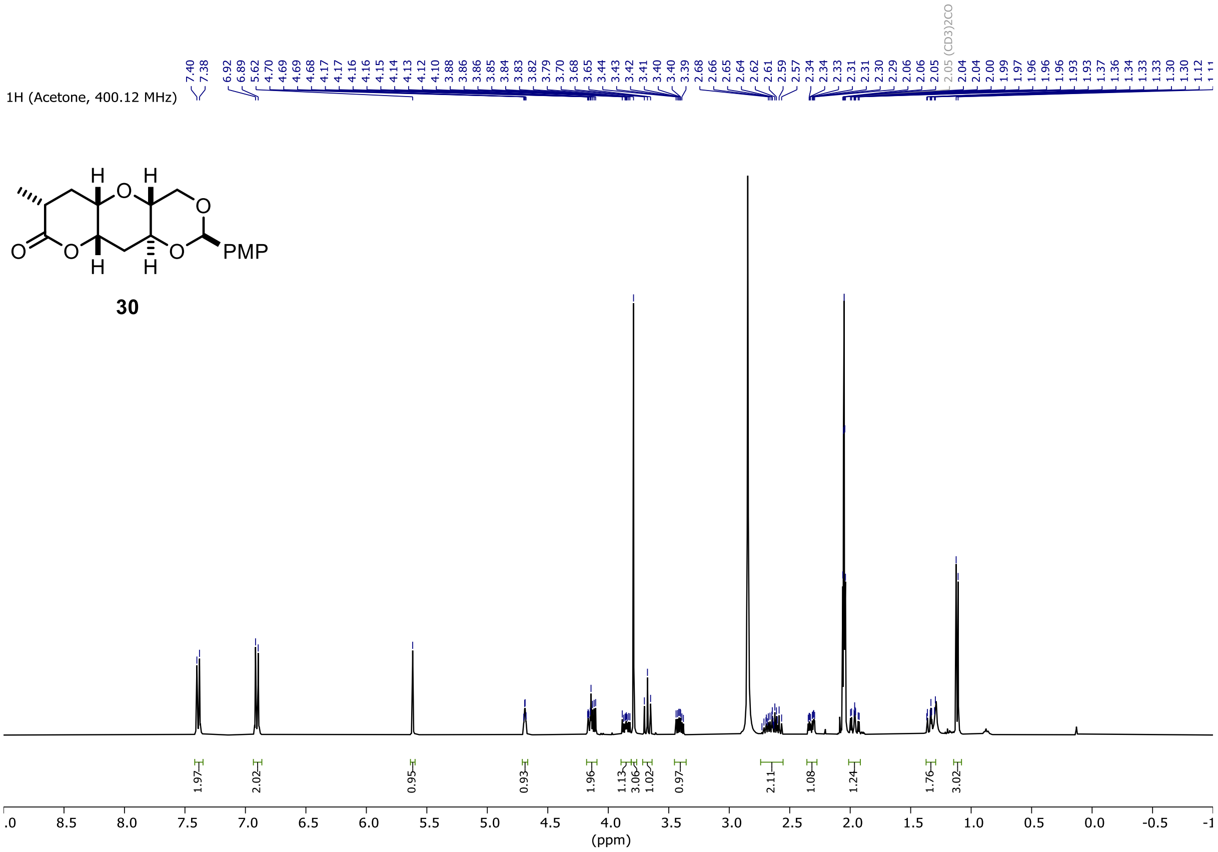

30

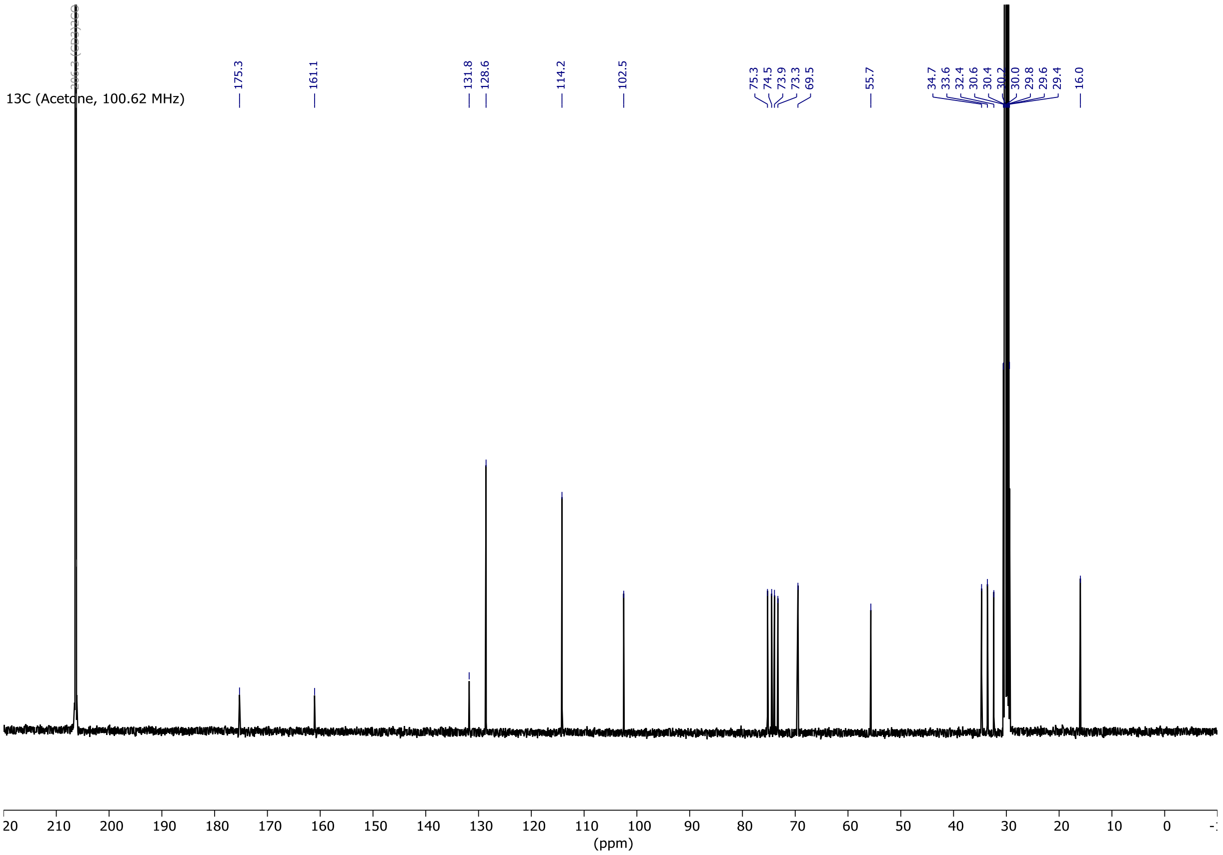

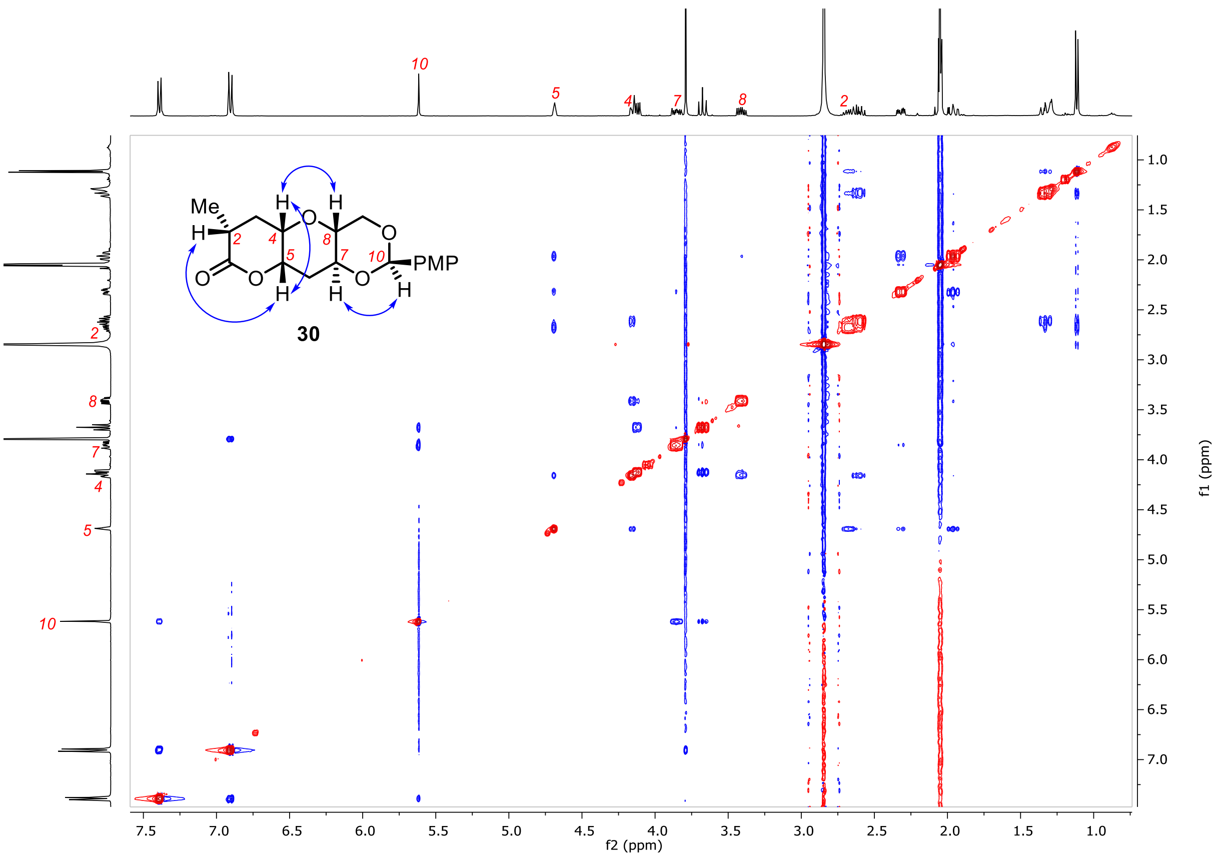

Figure S9. NOESY spectrum of compound **30** (400 MHz, [D$_6$]-acetone).

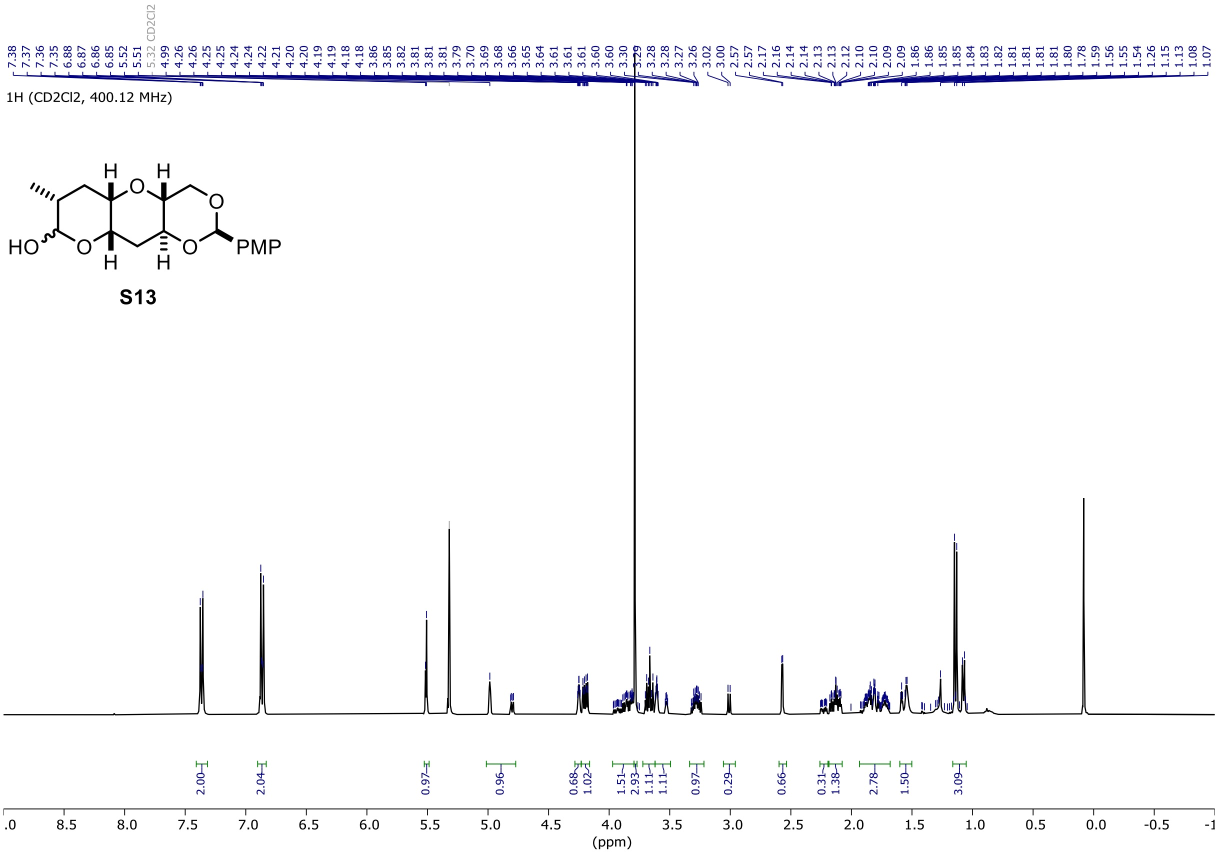
7.38 7.37 7.36 7.35 6.88 6.87 6.86 6.85 5.52 5.51 5.32 CD2Cl2 4.99 4.26 4.26 4.25 4.25 4.24 4.24 4.22 4.21 4.20 4.19 4.19 4.18 4.18 3.86 3.85 3.82 3.81 3.81 3.79 3.70 3.69 3.68 3.66 3.65 3.64 3.61 3.61 3.61 3.60 3.60 3.30 3.29 3.28 3.28 3.27 3.26 3.02 3.00 2.57 2.57 2.17 2.16 2.14 2.14 2.13 2.13 2.12 2.10 2.10 2.09 1.86 1.86 1.85 1.85 1.84 1.83 1.82 1.81 1.81 1.81 1.81 1.80 1.78 1.59 1.56 1.55 1.54 1.26 1.15 1.13 1.08 1.07
1H (CD2Cl2, 400.12 MHz)
H
H
O
O
HO
O
H
H
O
PMP
S13
2.00 2.04 0.97 0.96 0.68 1.02 1.51 2.93 1.11 1.11 0.97 0.29 0.66 0.31 1.38 2.78 1.50 3.09
(ppm)

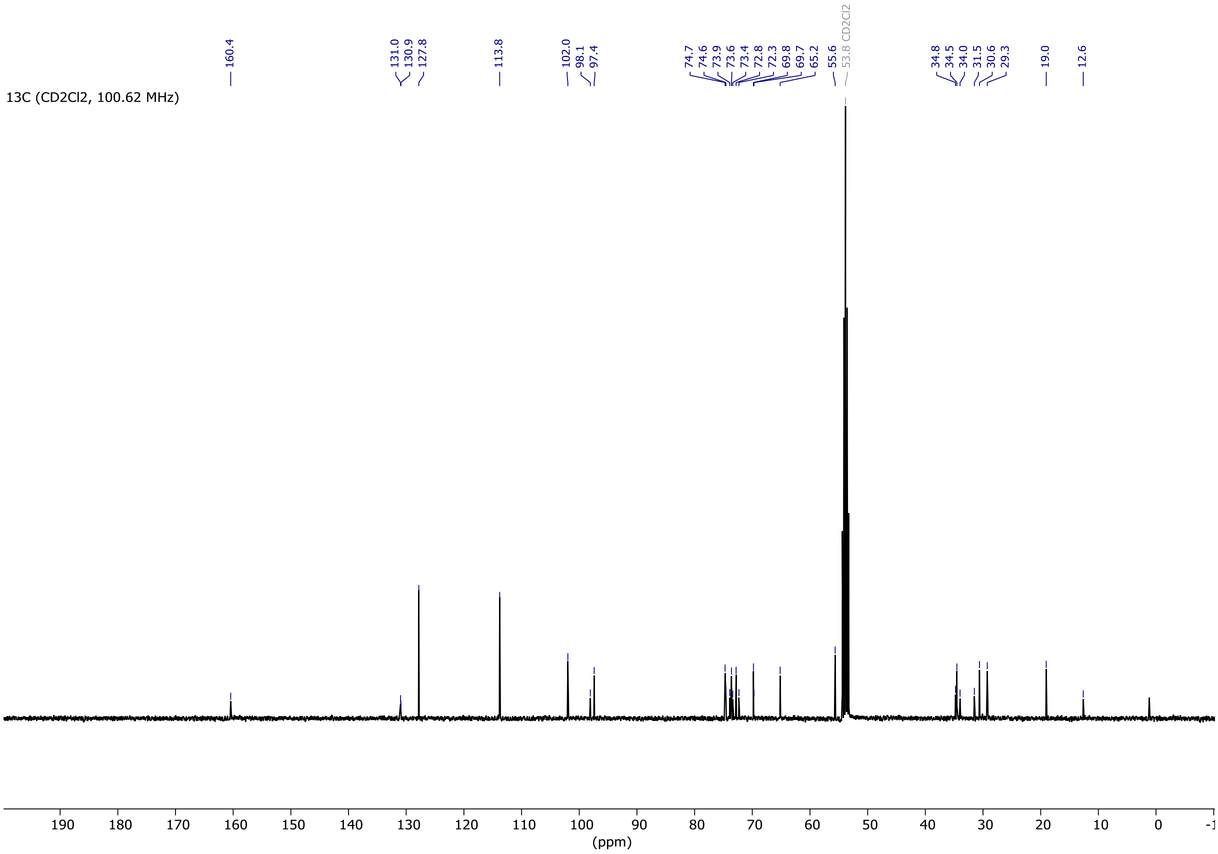
160.4 131.0 130.9 127.8 113.8 102.0 98.1 97.4 74.7 74.6 73.9 73.6 73.4 72.8 72.3 69.8 69.7 65.2 55.6 53.8 CD2Cl2 34.8 34.5 34.0 31.5 30.6 29.3 19.0 12.6
13C (CD2Cl2, 100.62 MHz)
(ppm)

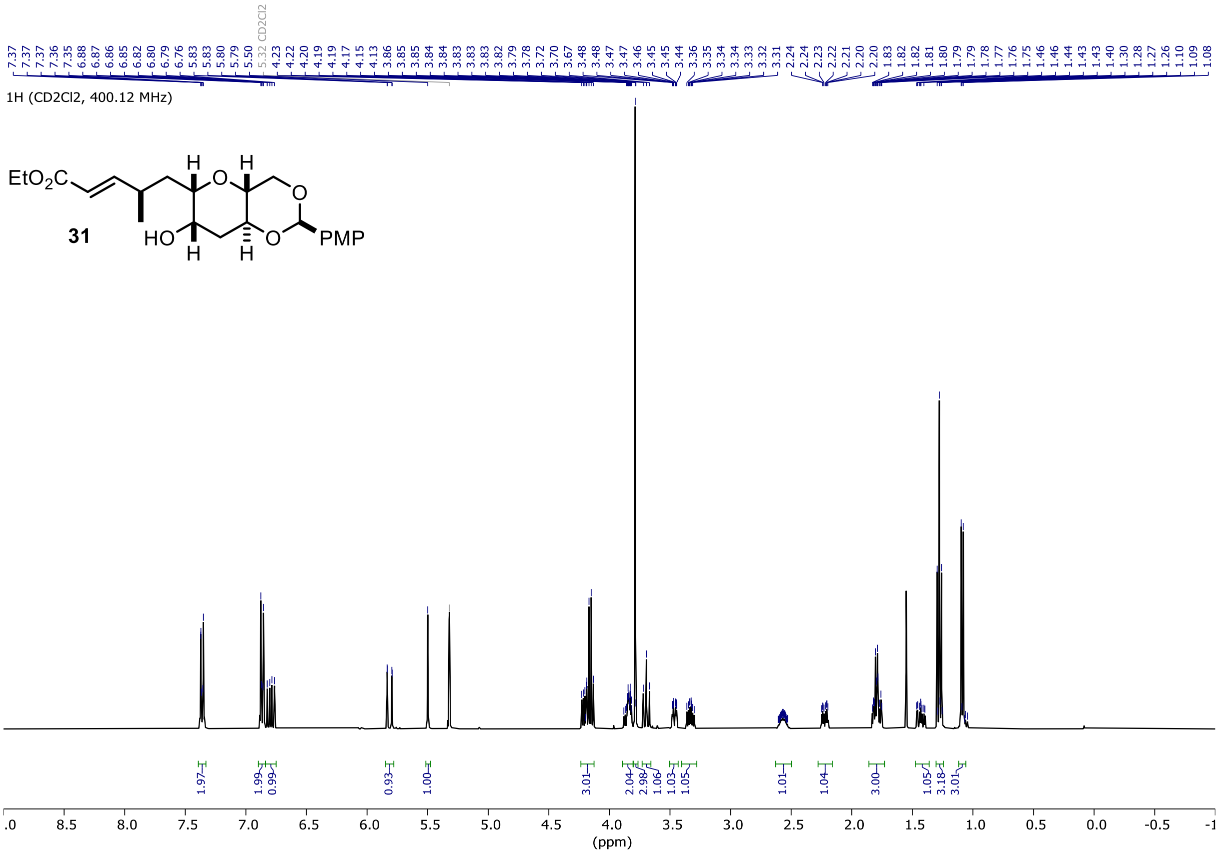
7.37 7.37 7.37 7.36 7.35 6.88 6.87 6.86 6.85 6.82 6.80 6.79 6.76 5.83 5.83 5.80 5.79 5.50 5.32 CD2Cl2 4.23 4.22 4.20 4.19 4.19 4.17 4.15 4.13 3.86 3.85 3.85 3.84 3.84 3.83 3.83 3.83 3.82 3.79 3.78 3.72 3.70 3.67 3.48 3.48 3.47 3.46 3.45 3.45 3.44 3.36 3.35 3.34 3.34 3.33 3.32 3.31 2.24 2.24 2.23 2.22 2.21 2.20 2.20 1.83 1.82 1.82 1.81 1.80 1.79 1.79 1.78 1.77 1.76 1.75 1.46 1.44 1.43 1.40 1.30 1.28 1.27 1.26 1.10 1.09 1.08
1H (CD2Cl2, 400.12 MHz)
EtO2C
H
O
H
O
31
HO
H
H
O
PMP
1.97 1.99 0.99 0.93 1.00 3.01 2.04 2.98 1.06 1.03 1.05 1.01 1.04 3.00 1.05 3.18 3.01
.0 8.5 8.0 7.5 7.0 6.5 6.0 5.5 5.0 4.5 4.0 3.5 3.0 2.5 2.0 1.5 1.0 0.5 0.0 -0.5 -1
(ppm)

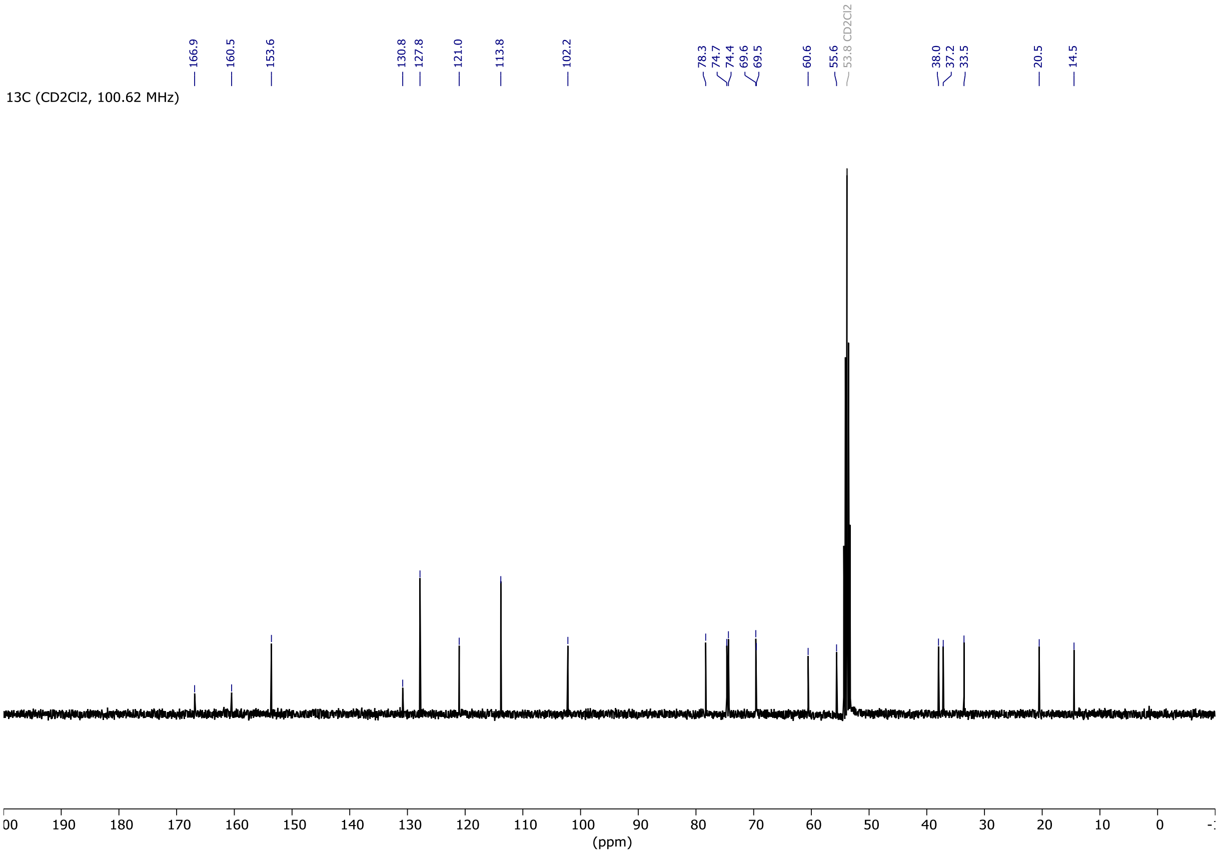
166.9 160.5 153.6 130.8 127.8 121.0 113.8 102.2 78.3 74.7 74.4 69.6 69.5 60.6 55.6 53.8 CD2Cl2 38.0 37.2 33.5 20.5 14.5
13C (CD2Cl2, 100.62 MHz)
00 190 180 170 160 150 140 130 120 110 100 90 80 70 60 50 40 30 20 10 0
(ppm)

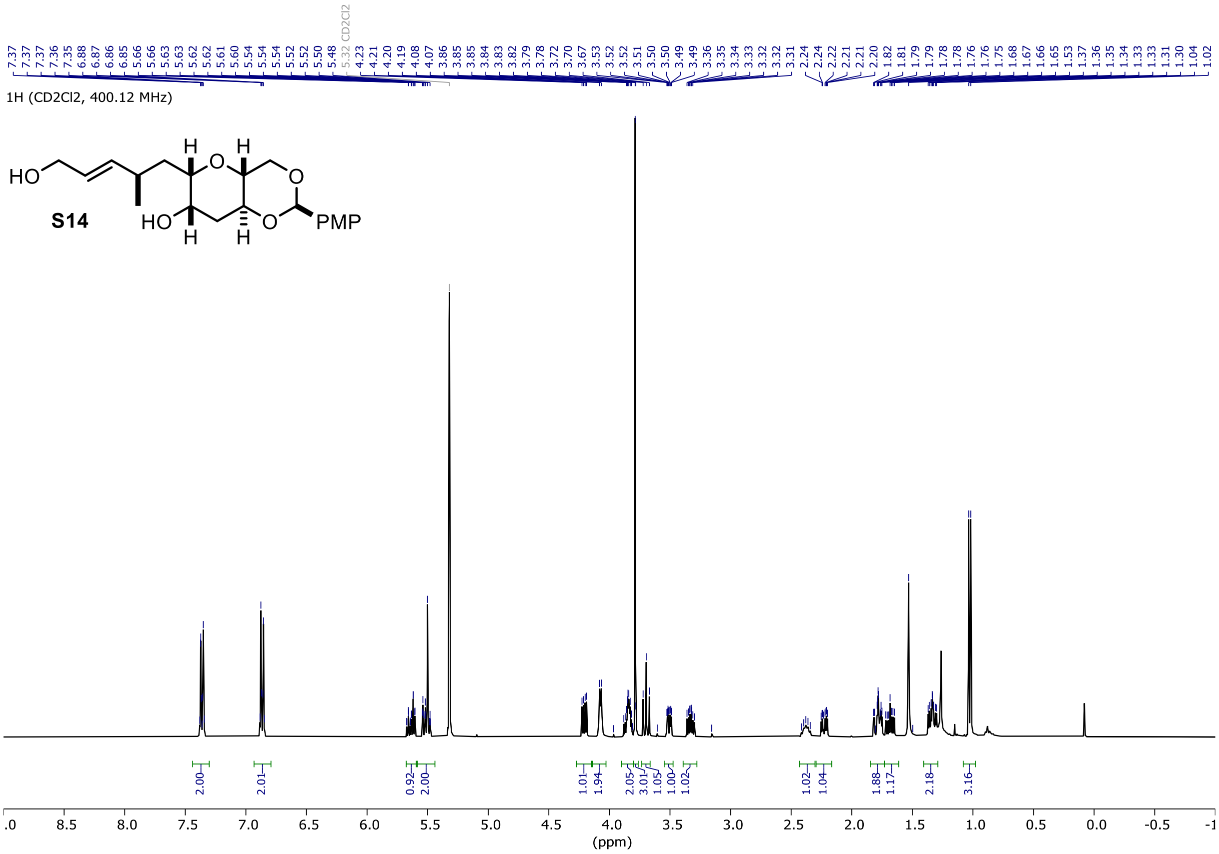
7.37 7.37 7.36 7.35 6.88 6.87 6.86 6.85 5.66 5.66 5.63 5.63 5.62 5.62 5.61 5.60 5.54 5.54 5.52 5.52 5.50 5.48 5.32 CD2Cl2 4.23 4.21 4.20 4.19 4.08 4.07 3.86 3.85 3.85 3.84 3.83 3.82 3.79 3.78 3.72 3.70 3.67 3.53 3.52 3.52 3.51 3.50 3.50 3.49 3.49 3.36 3.35 3.34 3.33 3.32 3.32 3.31 2.24 2.24 2.22 2.21 2.21 2.20 1.82 1.81 1.79 1.79 1.78 1.76 1.76 1.75 1.68 1.67 1.66 1.65 1.53 1.37 1.36 1.35 1.34 1.33 1.33 1.31 1.30 1.04 1.02
1H (CD2Cl2, 400.12 MHz)
HO
O
H
H
O
HO
H
H
O
PMP
S14
2.00
2.01
0.92
2.00
1.01
1.94
2.05
3.01
1.05
1.00
1.02
1.02
1.04
1.88
1.17
2.18
3.16
9.0 8.5 8.0 7.5 7.0 6.5 6.0 5.5 5.0 4.5 4.0 3.5 3.0 2.5 2.0 1.5 1.0 0.5 0.0 -0.5 -1
(ppm)

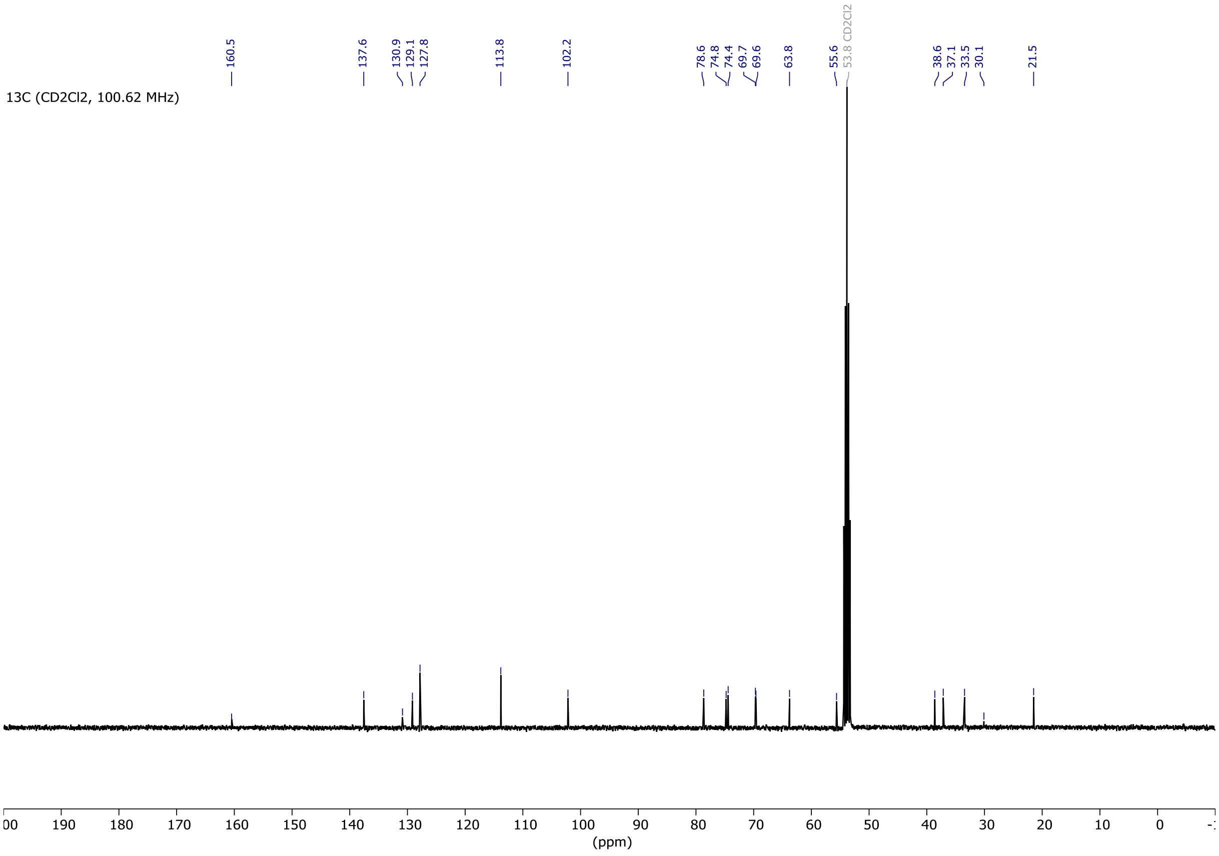
160.5 137.6 130.9 129.1 127.8 113.8 102.2 78.6 74.8 74.4 69.7 69.6 63.8 55.6 53.8 CD2Cl2 38.6 37.1 33.5 30.1 21.5
13C (CD2Cl2, 100.62 MHz)
200 190 180 170 160 150 140 130 120 110 100 90 80 70 60 50 40 30 20 10 0
(ppm)

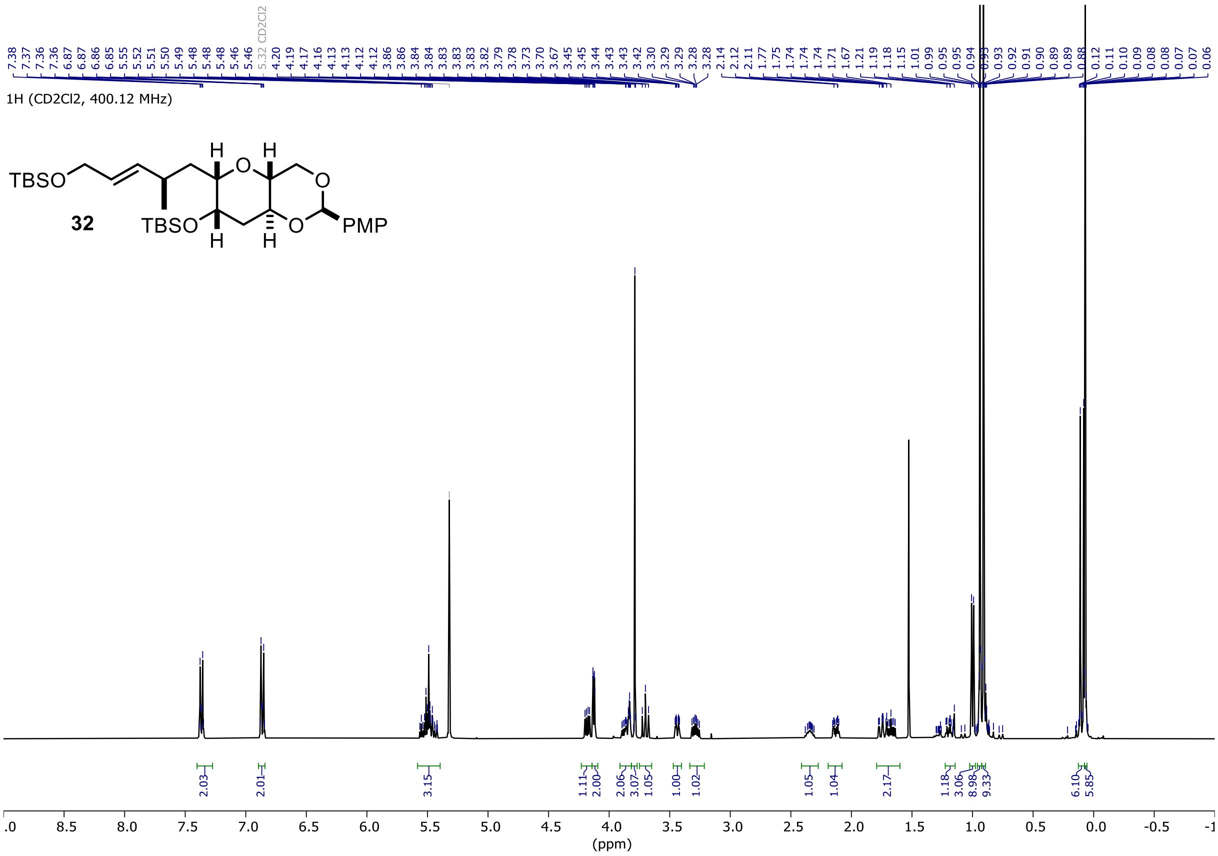
7.38 7.37 7.36 7.36 6.87 6.86 6.85 5.55 5.52 5.51 5.50 5.49 5.48 5.48 5.48 5.46 5.46 5.32 CD2Cl2 4.20 4.19 4.17 4.16 4.13 4.13 4.12 4.12 3.86 3.86 3.84 3.84 3.83 3.83 3.83 3.82 3.79 3.78 3.73 3.70 3.67 3.45 3.45 3.44 3.43 3.43 3.42 3.30 3.29 3.29 3.28 3.28 2.14 2.12 2.11 1.77 1.75 1.74 1.74 1.74 1.71 1.67 1.21 1.19 1.18 1.15 1.01 0.99 0.95 0.95 0.94 0.93 0.93 0.92 0.91 0.90 0.89 0.88 0.12 0.11 0.10 0.09 0.08 0.08 0.07 0.06
1H (CD2Cl2, 400.12 MHz)
TBSO
32
TBSO
H
O
H
O
O
PMP
2.03 2.01 3.15 1.11 2.00 2.06 3.07 1.05 1.00 1.02 1.05 1.04 2.17 1.18 3.06 8.98 9.33 6.10 5.85
(ppm)

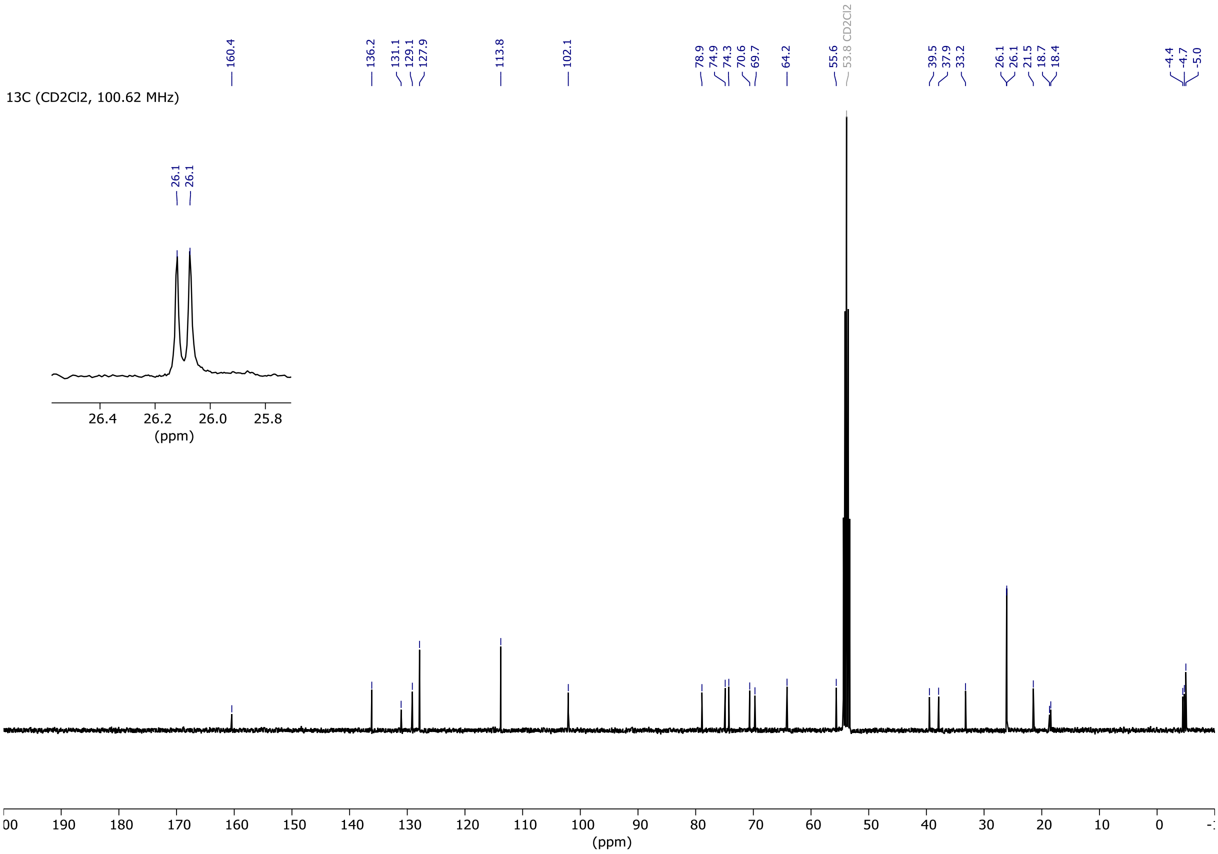
160.4 136.2 131.1 129.1 127.9 113.8 102.1 78.9 74.9 74.3 70.6 69.7 64.2 55.6 53.8 CD2Cl2 39.5 37.9 33.2 26.1 26.1 21.7 18.7 18.4 -4.4 -4.7 -5.0
13C (CD2Cl2, 100.62 MHz)
26.1 26.1
(ppm)
(ppm)

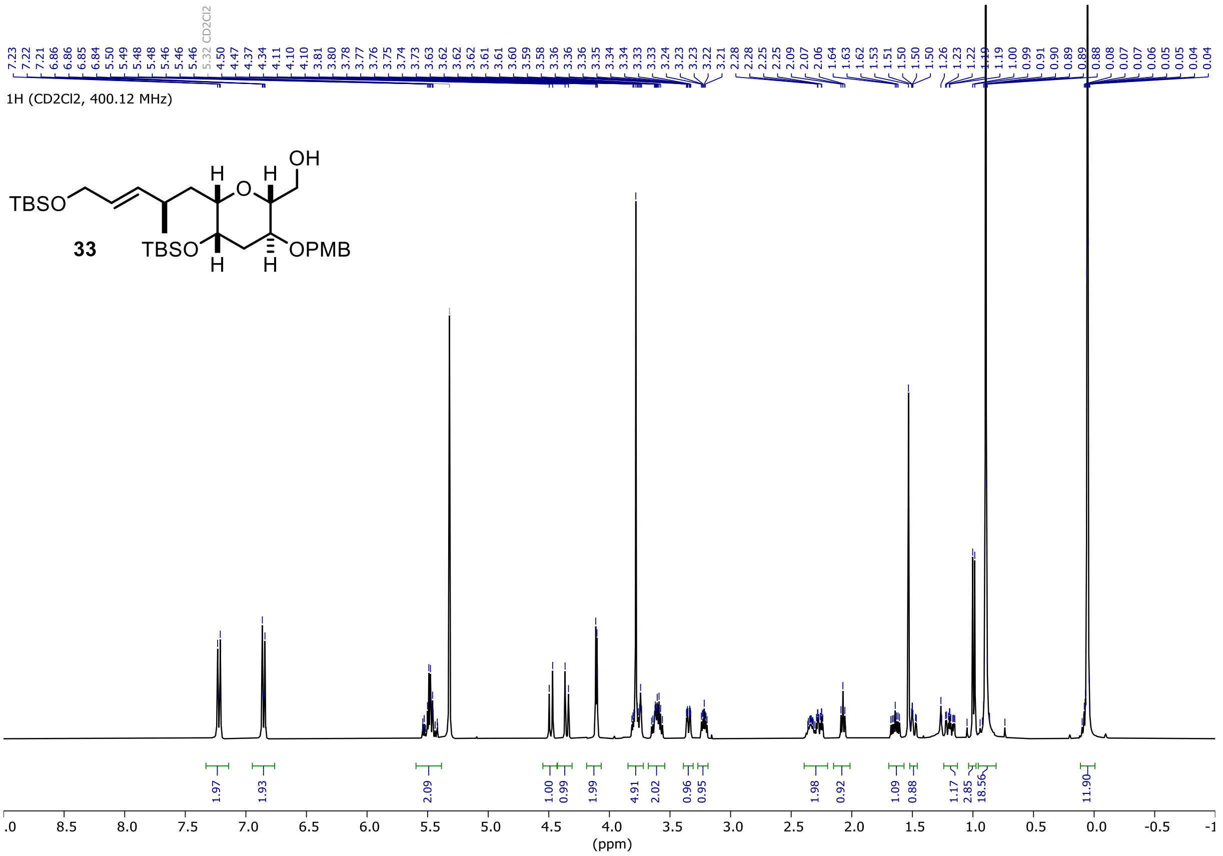
1H (CD2Cl2, 400.12 MHz)
TBSO
33
TBSO
OH
OPMB
(ppm)

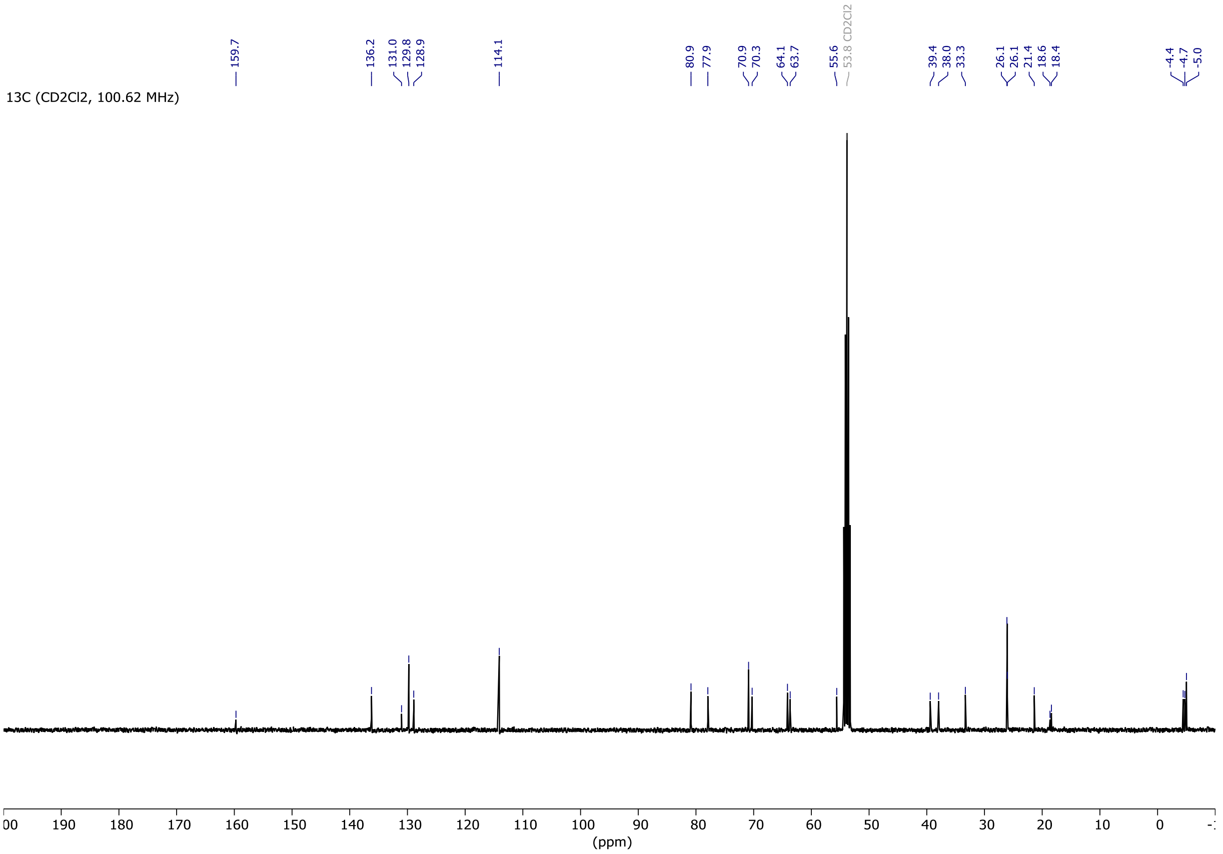
13C (CD2Cl2, 100.62 MHz)
159.7
136.2
131.0
129.8
128.9
114.1
80.9
77.9
70.9
70.3
64.1
63.7
55.6
53.8 CD2Cl2
39.4
38.0
33.3
26.1
26.1
21.4
18.6
18.4
-4.4
-4.7
-5.0
(ppm)

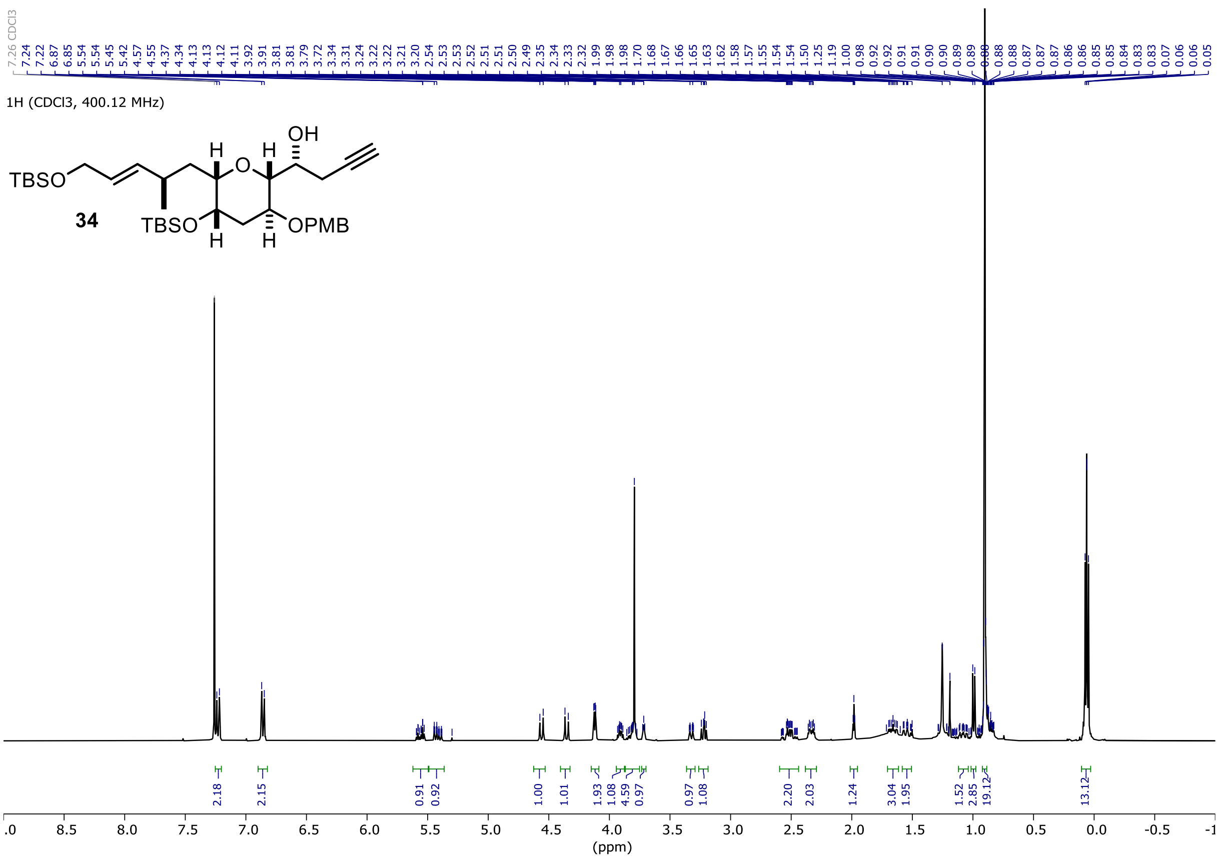
7.26 CDCl3
7.24 7.22 6.87 6.85 5.54 5.54 5.45 5.42 4.57 4.55 4.37 4.34 4.13 4.13 4.12 4.11 3.92 3.91 3.81 3.81 3.79 3.72 3.34 3.31 3.24 3.22 3.22 3.21 3.20 2.54 2.53 2.53 2.52 2.51 2.51 2.50 2.49 2.35 2.34 2.33 2.32 1.99 1.98 1.98 1.70 1.68 1.67 1.66 1.65 1.63 1.62 1.58 1.57 1.55 1.54 1.54 1.50 1.25 1.19 1.00 0.98 0.92 0.92 0.91 0.91 0.90 0.90 0.89 0.89 0.88 0.88 0.87 0.87 0.87 0.86 0.86 0.85 0.85 0.84 0.83 0.83 0.07 0.06 0.06 0.05
1H (CDCl3, 400.12 MHz)
OH
H
H
TBSO
34
TBSO
H
H
OPMB
2.18
2.15
0.91
0.92
1.00
1.01
1.93
1.08
4.59
0.97
0.97
1.08
2.20
2.03
1.24
3.04
1.95
1.52
2.85
19.12
13.12
(ppm)

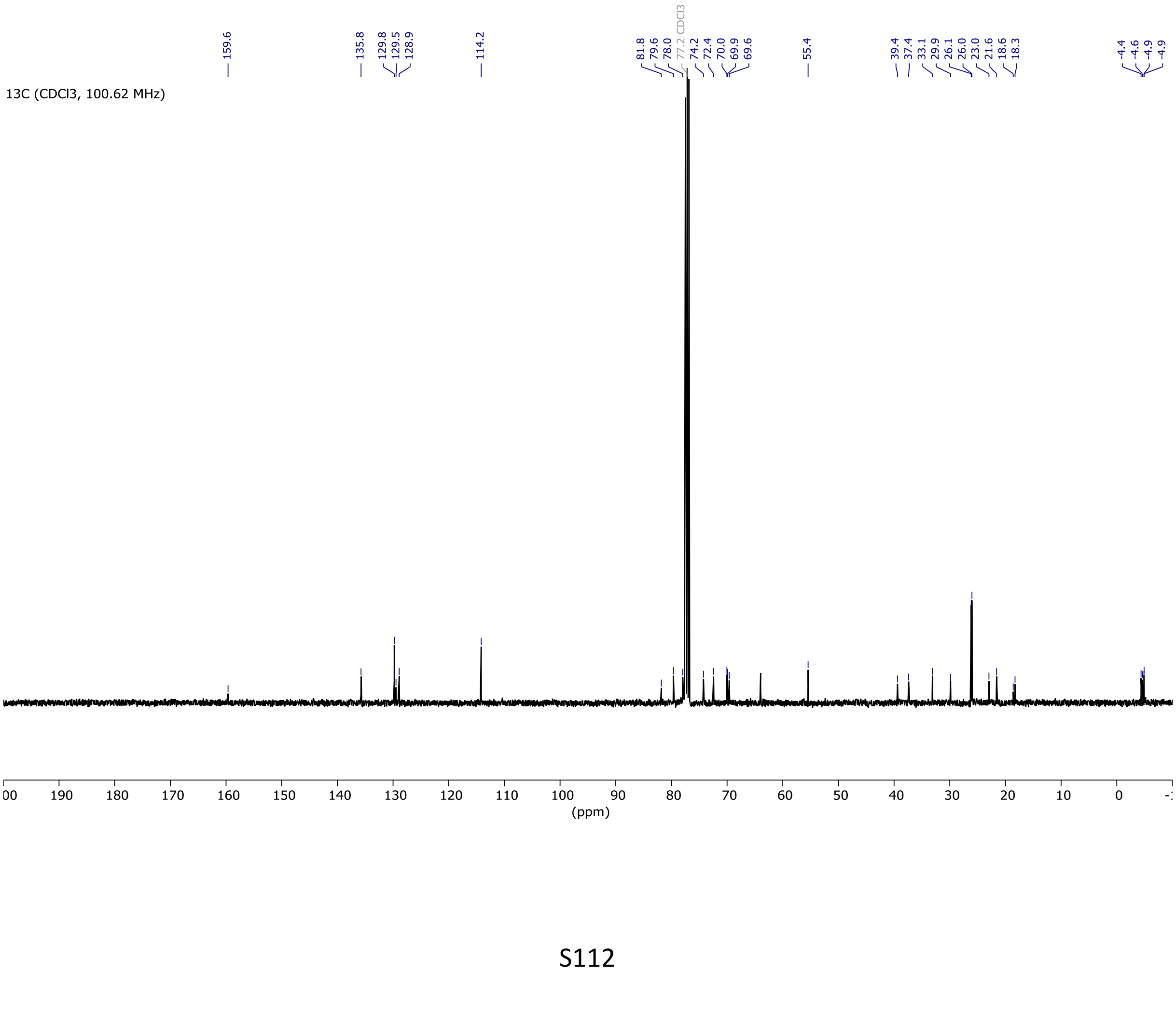
159.6
135.8
129.8
129.5
128.9
114.2
81.8
79.6
78.0
77.2 CDCl3
74.2
72.4
70.0
69.9
69.6
55.4
39.4
37.4
33.1
29.9
26.1
26.0
23.0
21.6
18.6
18.3
-4.4
-4.6
-4.9
-4.9
13C (CDCl3, 100.62 MHz)
(ppm)

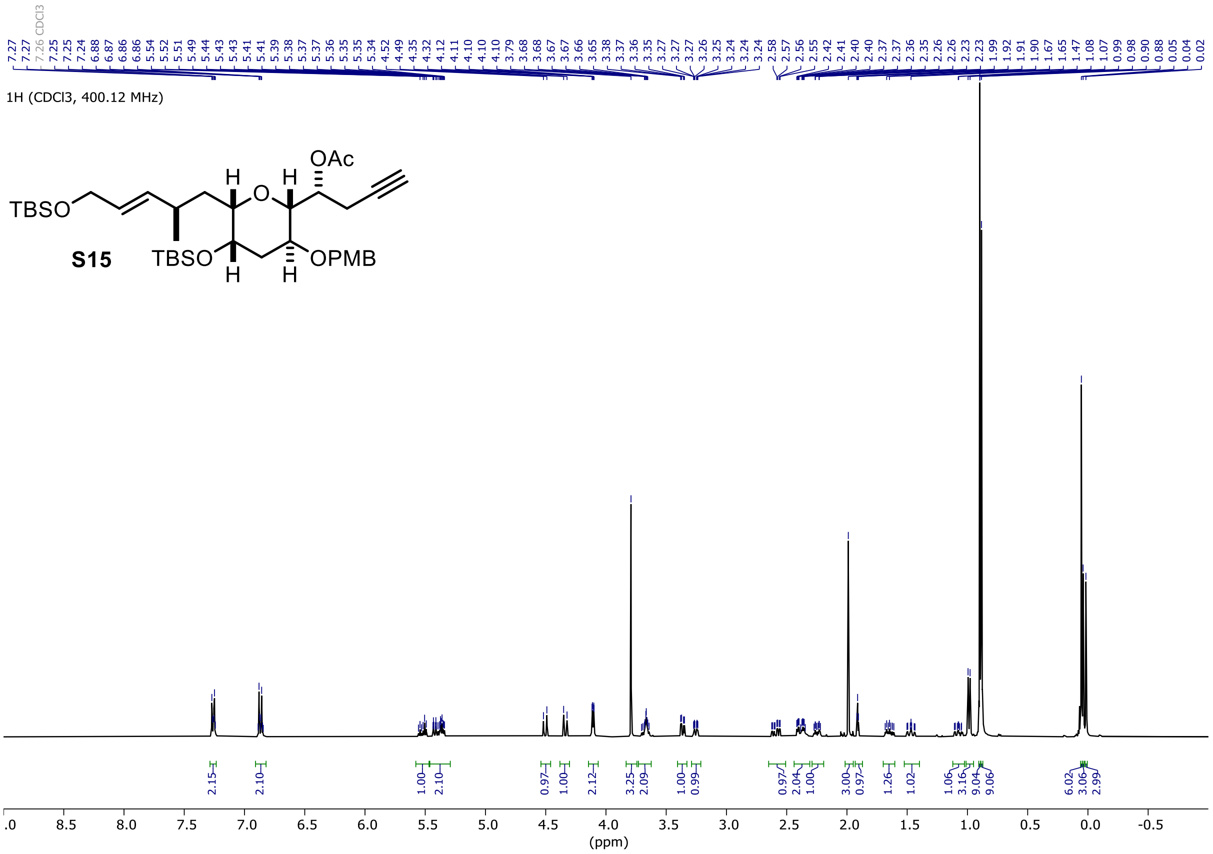

7.27 7.27 7.26 CDCl3 7.25 7.25 7.24 6.88 6.87 6.86 6.86 5.54 5.52 5.51 5.49 5.44 5.43 5.43 5.41 5.39 5.38 5.37 5.37 5.36 5.35 5.35 5.34 4.52 4.49 4.35 4.32 4.12 4.11 4.10 4.10 3.79 3.68 3.68 3.67 3.67 3.66 3.65 3.38 3.37 3.36 3.35 3.27 3.27 3.26 3.25 3.24 3.24 3.24 2.58 2.57 2.56 2.55 2.42 2.41 2.40 2.40 2.37 2.37 2.37 2.36 2.35 2.26 2.26 2.23 2.23 1.99 1.92 1.91 1.90 1.67 1.65 1.47 1.08 1.07 0.99 0.98 0.90 0.88 0.05 0.04 0.02
1H (CDCl3, 400.12 MHz)
OAc
TBSO
H
O
H
S15
TBSO
H
H
OPMB
2.15 2.10 1.00 2.10 0.97 1.00 2.12 3.25 2.09 1.00 0.99 0.97 2.04 1.00 3.00 0.97 1.26 1.02 1.06 3.16 9.04 9.06 6.02 3.06 2.99
.0 8.5 8.0 7.5 7.0 6.5 6.0 5.5 5.0 4.5 4.0 3.5 3.0 2.5 2.0 1.5 1.0 0.5 0.0 -0.5
(ppm)

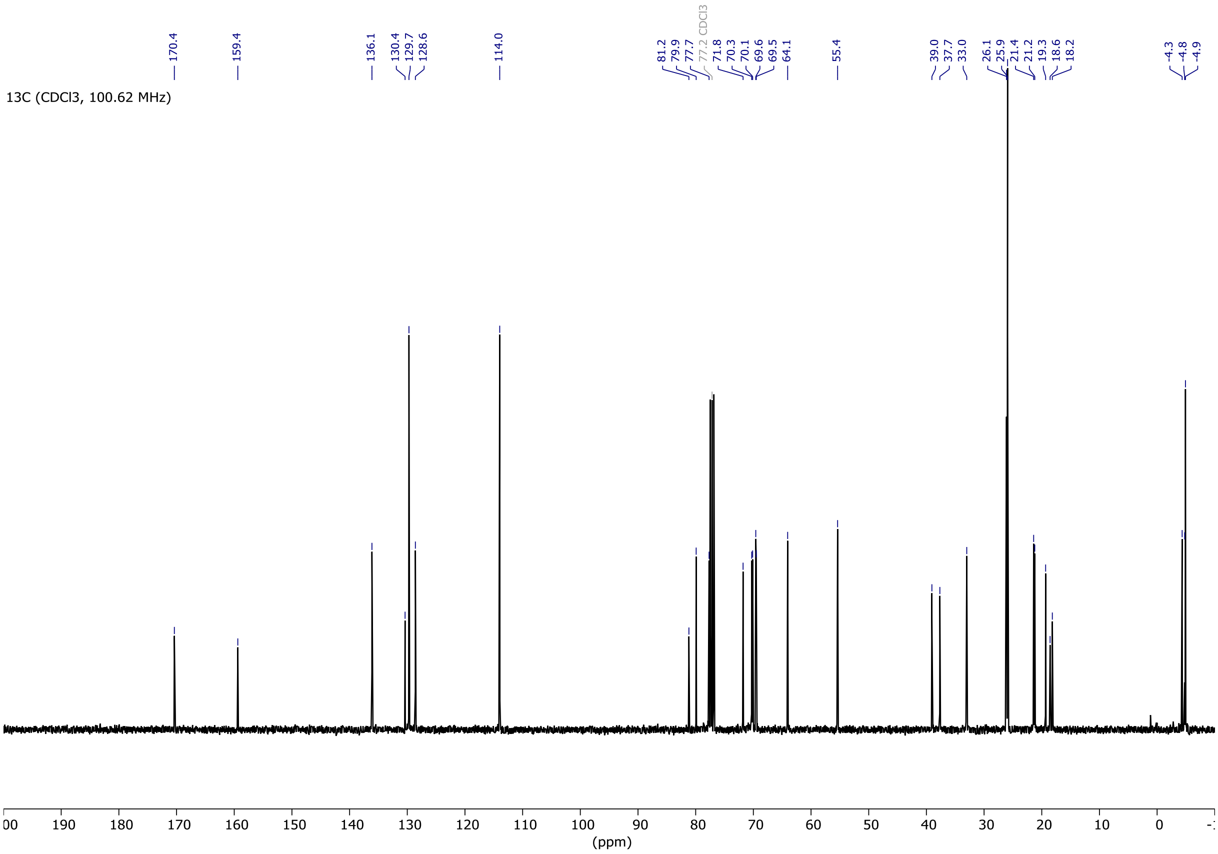

170.4 159.4 136.1 130.4 129.7 128.6 114.0 81.2 79.9 77.7 77.2 CDCl3 71.8 70.3 70.1 69.6 69.5 64.1 55.4 39.0 37.7 33.0 26.1 25.9 21.4 21.2 19.3 18.6 18.2 -4.3 -4.8 -4.9
13C (CDCl3, 100.62 MHz)
00 190 180 170 160 150 140 130 120 110 100 90 80 70 60 50 40 30 20 10 0
(ppm)

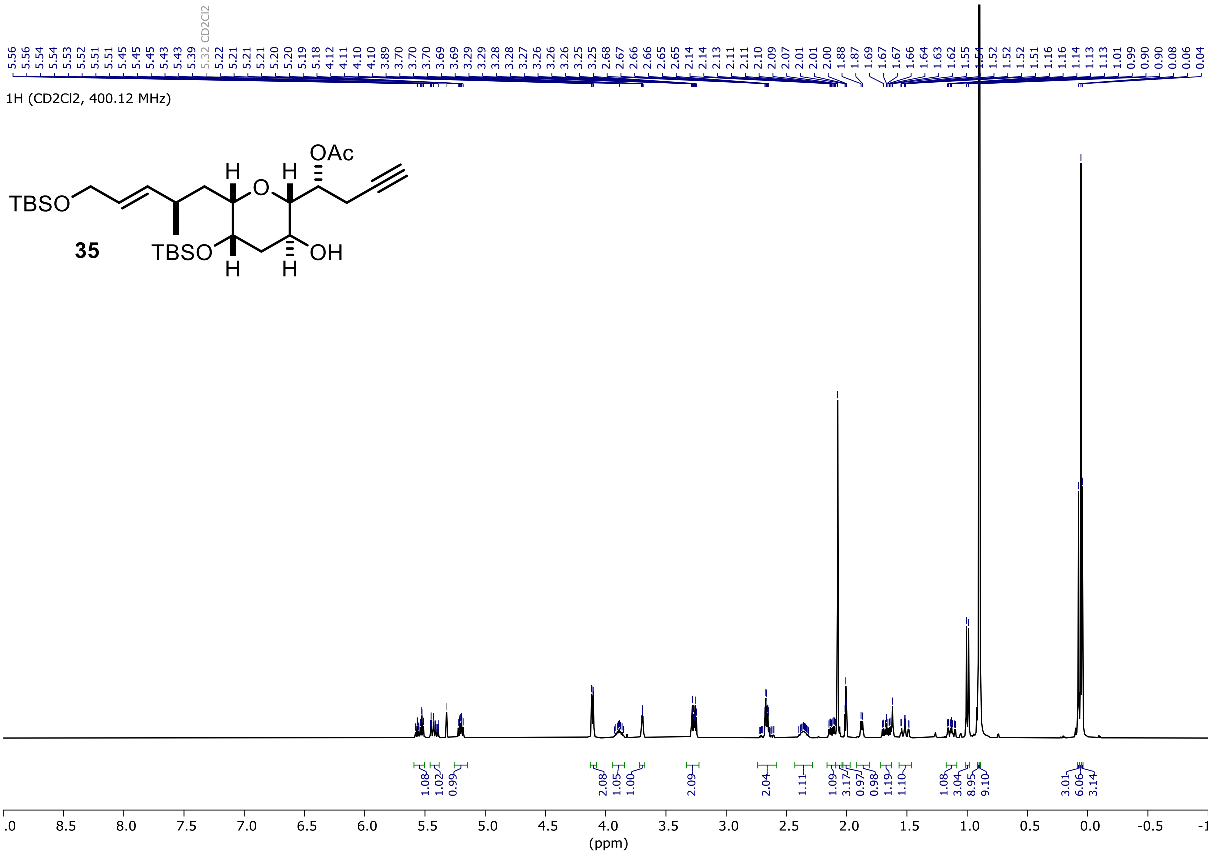
1H (CD2Cl2, 400.12 MHz)
5.32 CD2Cl2
(ppm)
TBSO
OAc
H
O
H
35
TBSO
H
H
OH

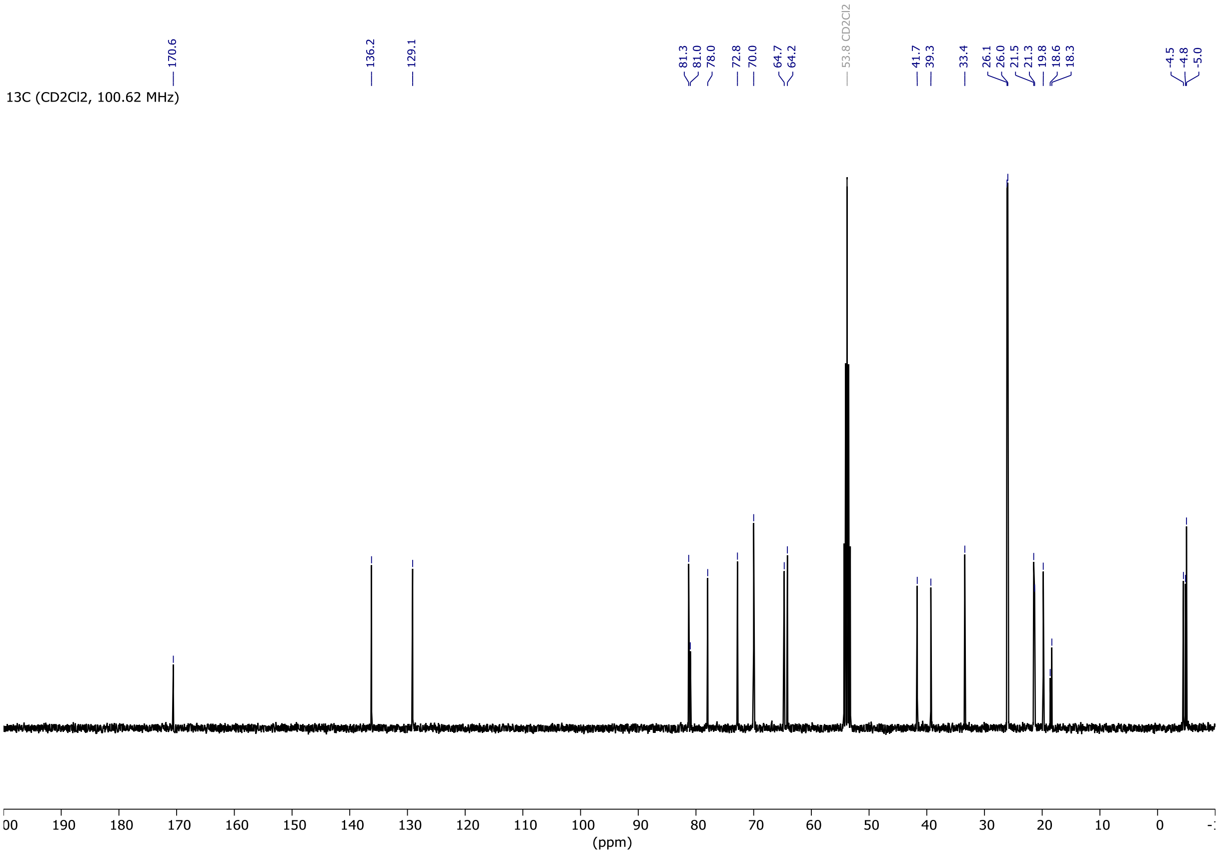
13C (CD2Cl2, 100.62 MHz)
170.6
136.2
129.1
81.3
81.0
78.0
72.8
70.0
64.7
64.2
53.8 CD2Cl2
41.7
39.3
33.4
26.1
26.0
21.5
21.3
19.8
18.6
18.3
-4.5
-4.8
-5.0
(ppm)

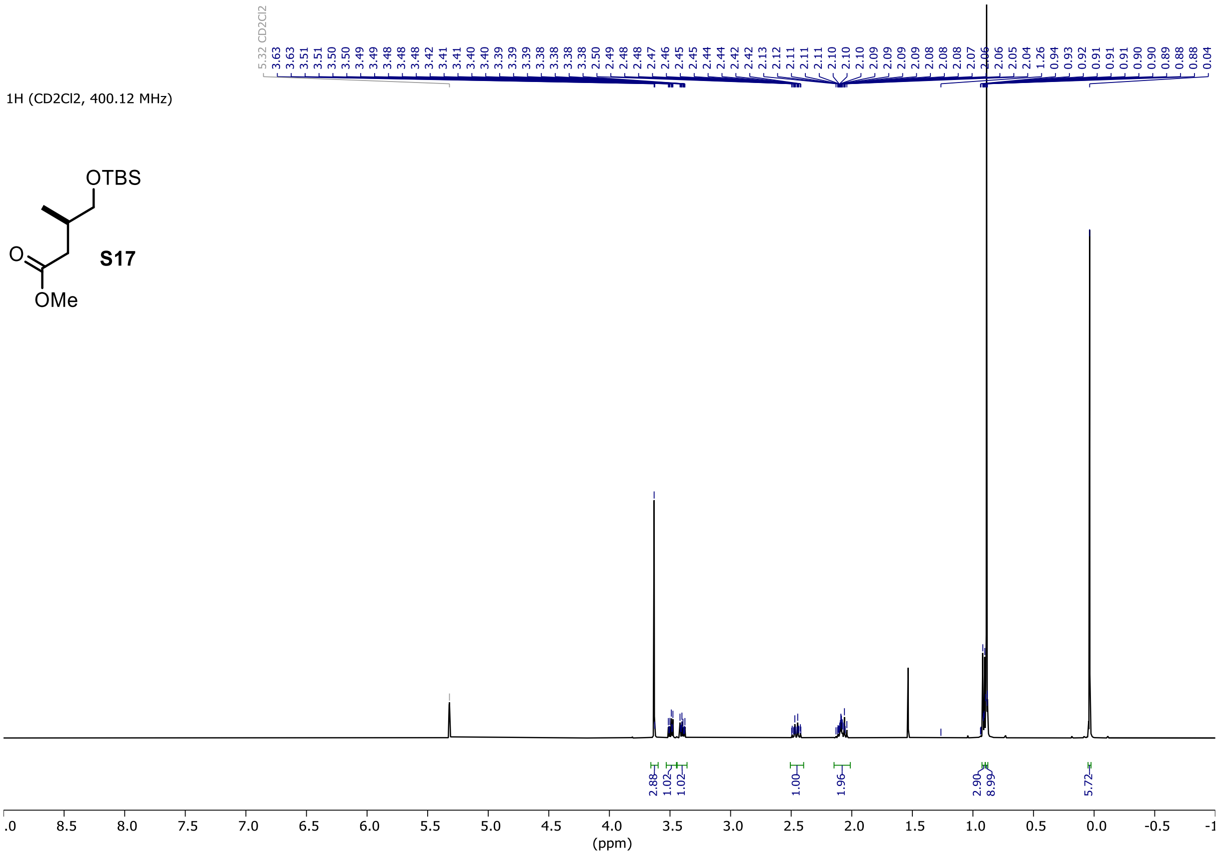
1H (CD2Cl2, 400.12 MHz)
OTBS
O
OMe
S17
(ppm)

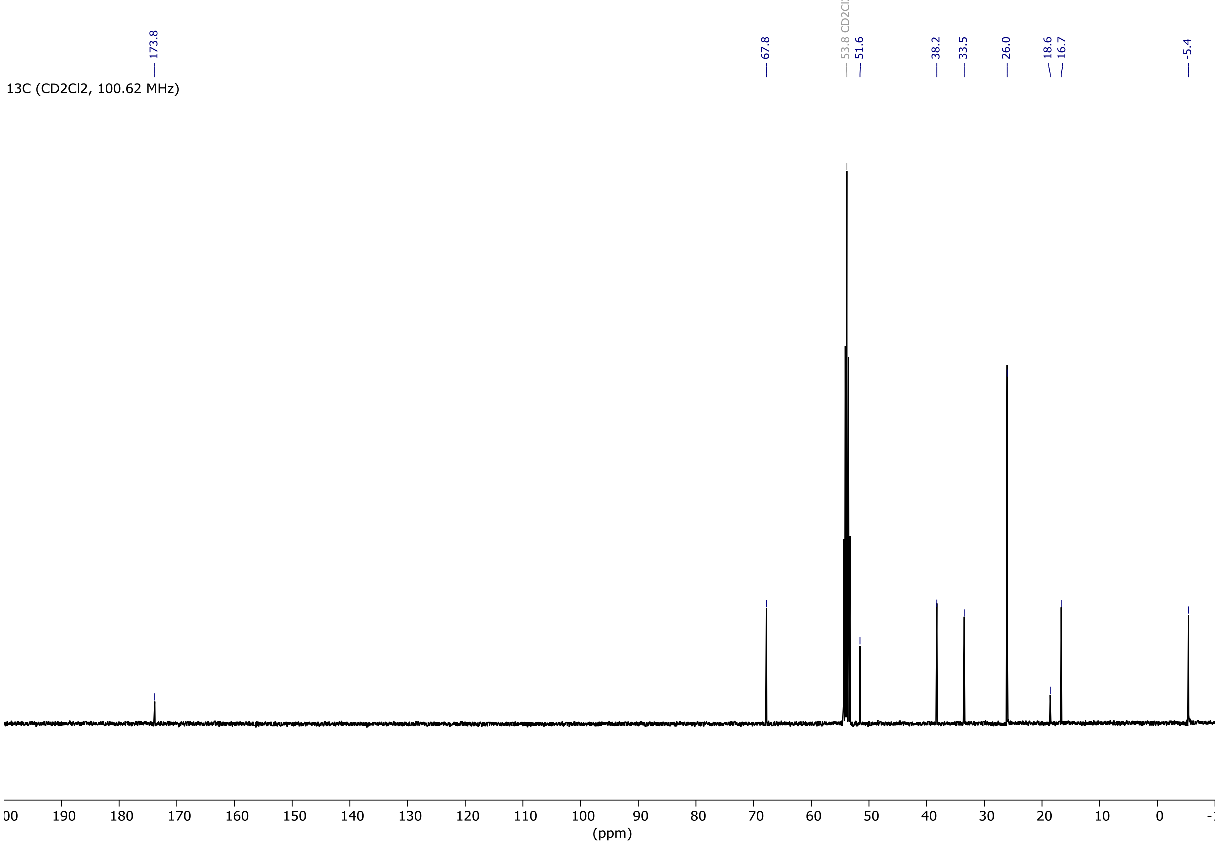
13C (CD2Cl2, 100.62 MHz)
173.8
67.8
53.8 CD2Cl2
51.6
38.2
33.5
26.0
18.6
16.7
-5.4
(ppm)

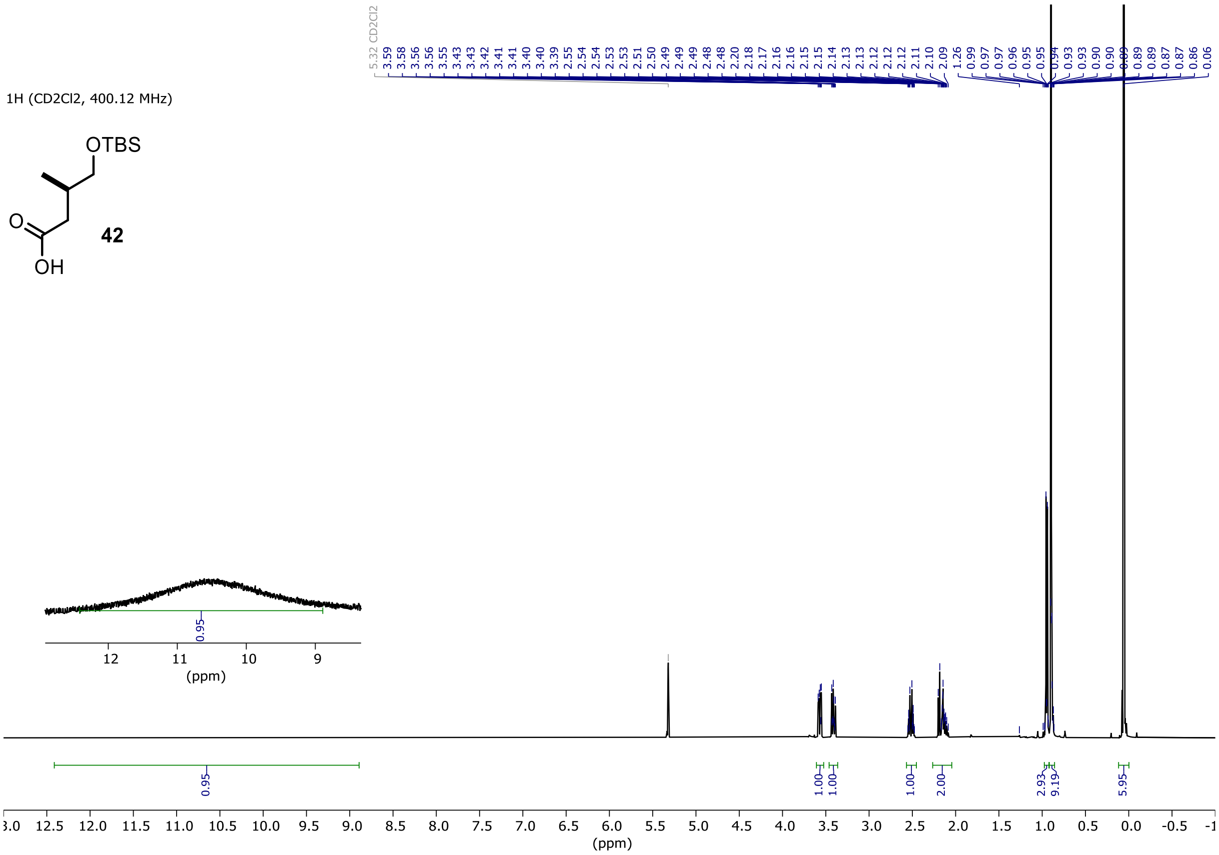
1H (CD2Cl2, 400.12 MHz)
OTBS
42
OH

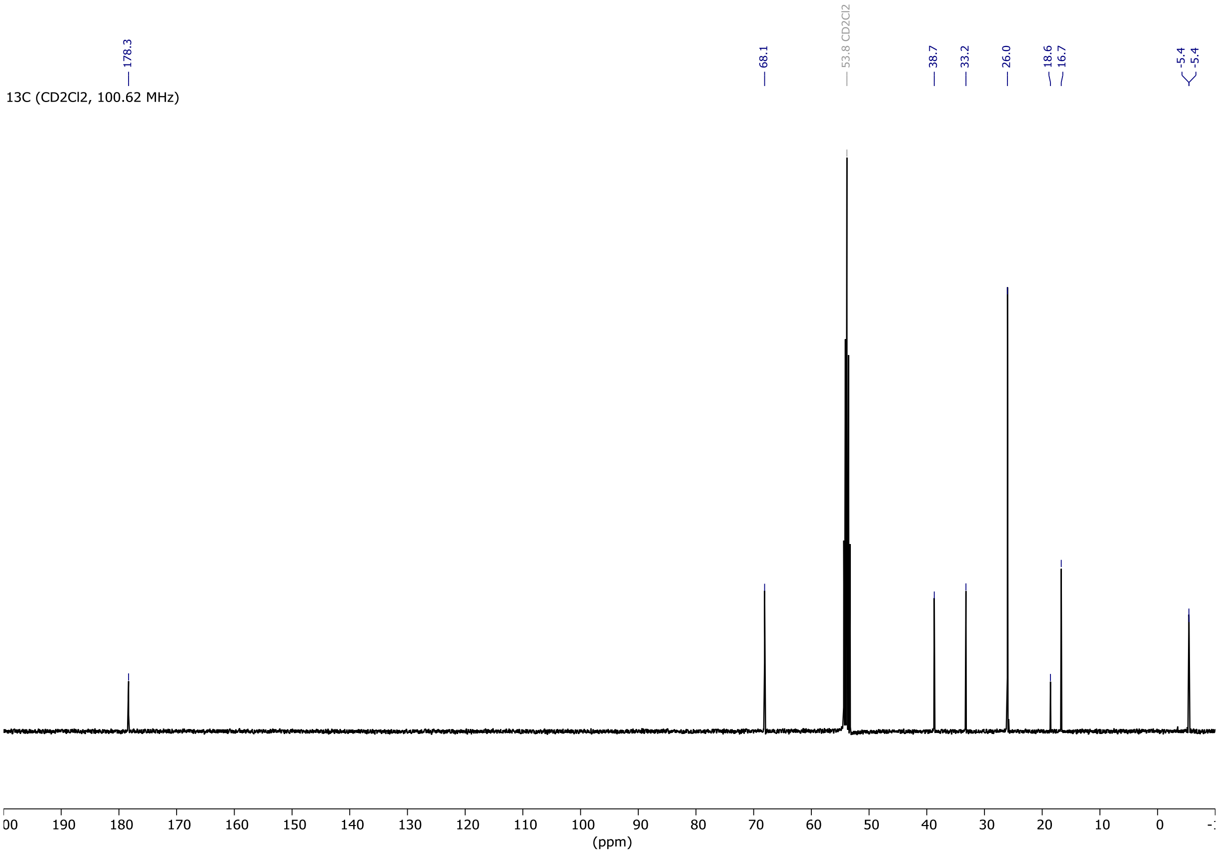
13C (CD2Cl2, 100.62 MHz)
178.3
68.1
53.8 CD2Cl2
38.7
33.2
26.0
18.6
16.7
-5.4
-5.4

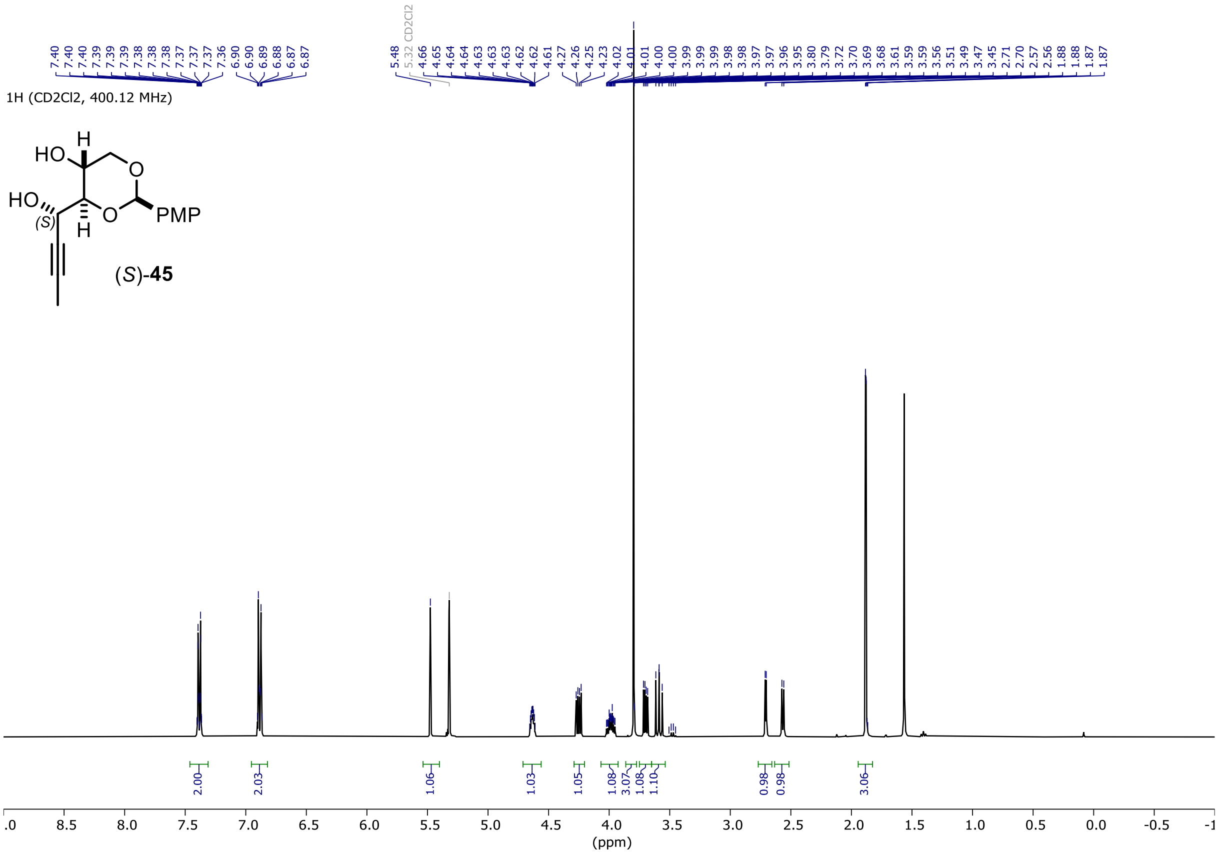
1H (CD2Cl2, 400.12 MHz)
(S)-45

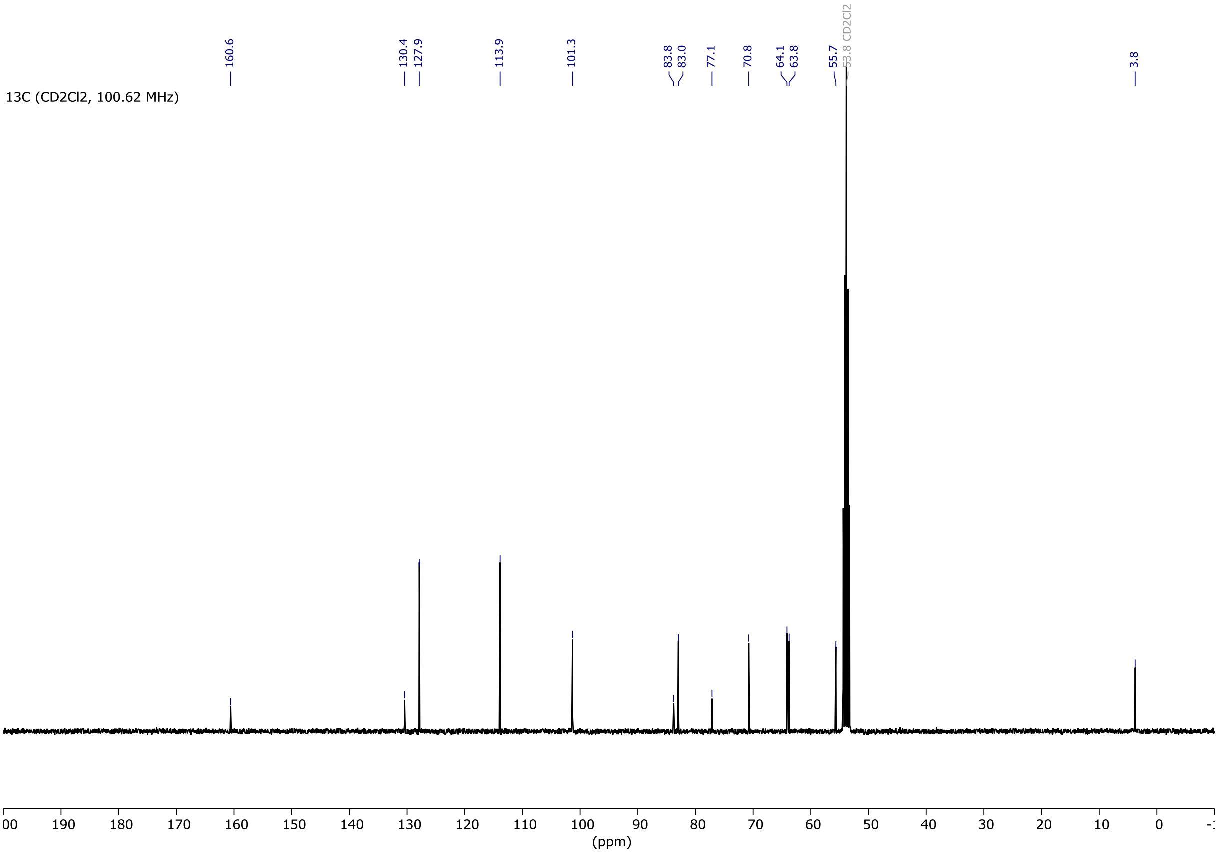
13C (CD2Cl2, 100.62 MHz)
160.6
130.4
127.9
113.9
101.3
83.8
83.0
77.1
70.8
64.1
63.8
55.7
53.8 CD2Cl2
3.8
(ppm)

1H (CD2Cl2, 400.12 MHz)

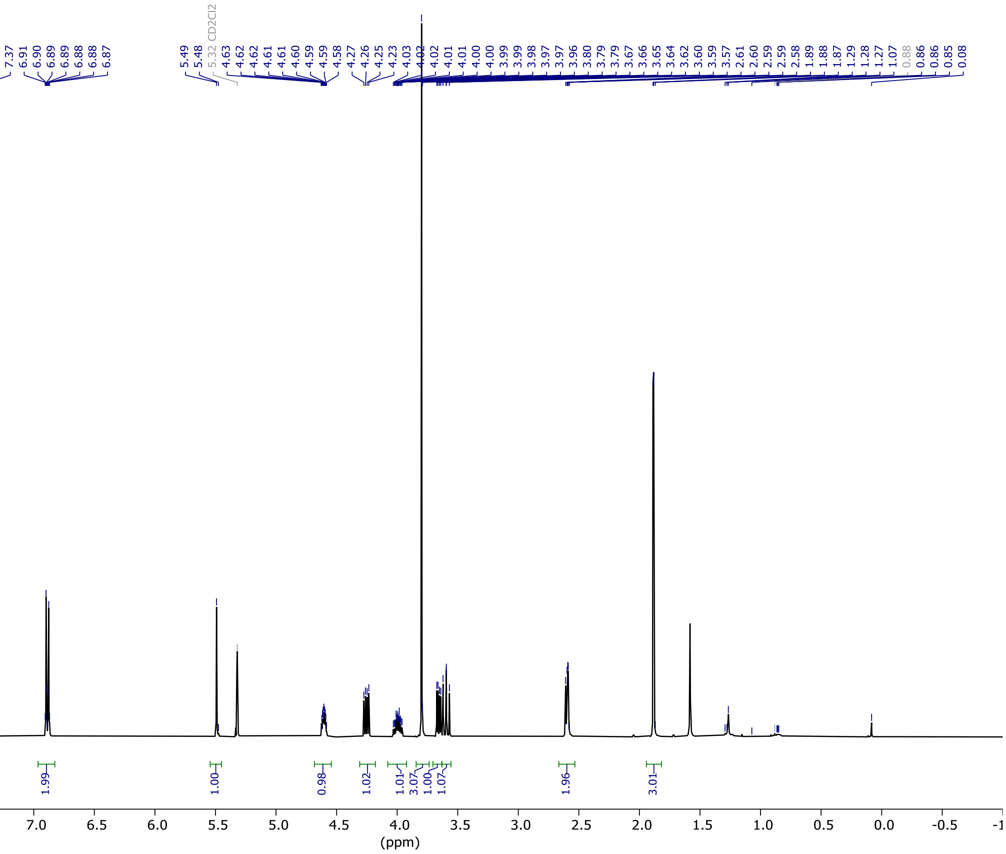

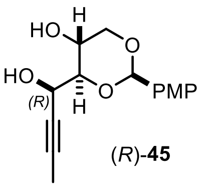

(*R*)-**45**

13C (CD2Cl2, 100.62 MHz)

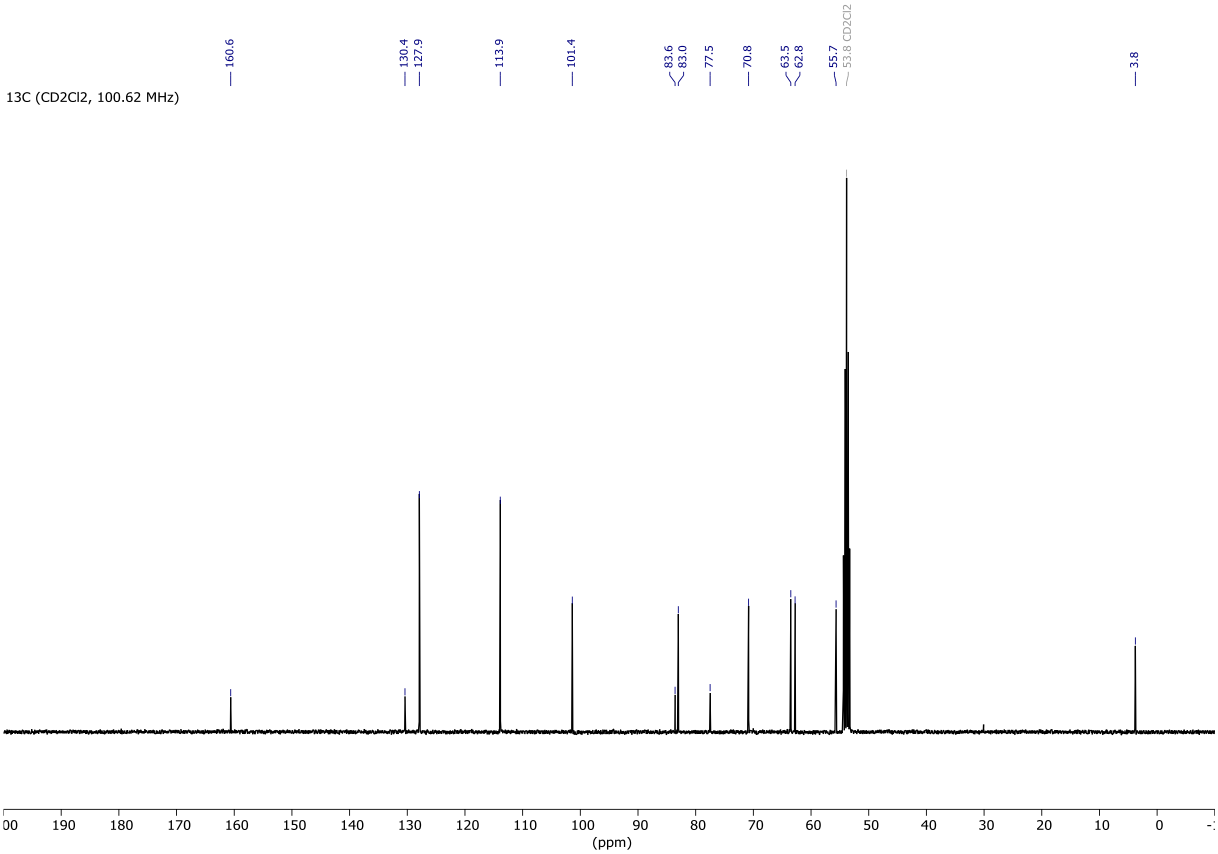

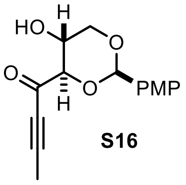

S16

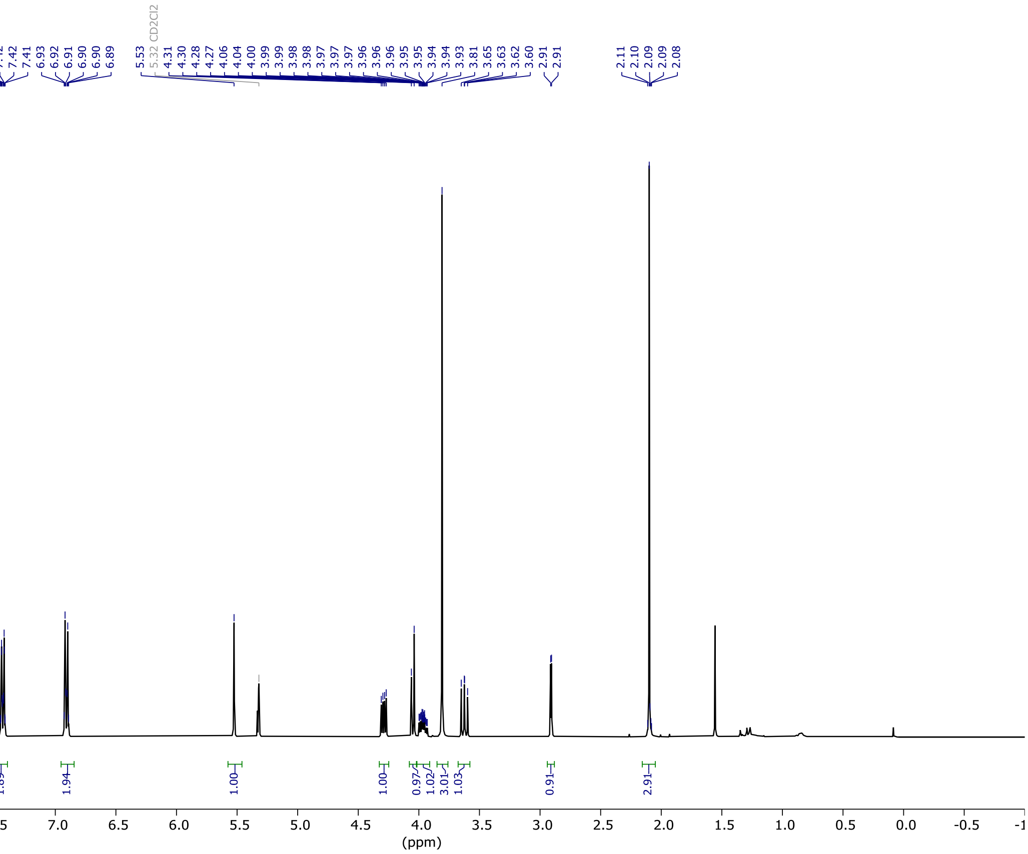

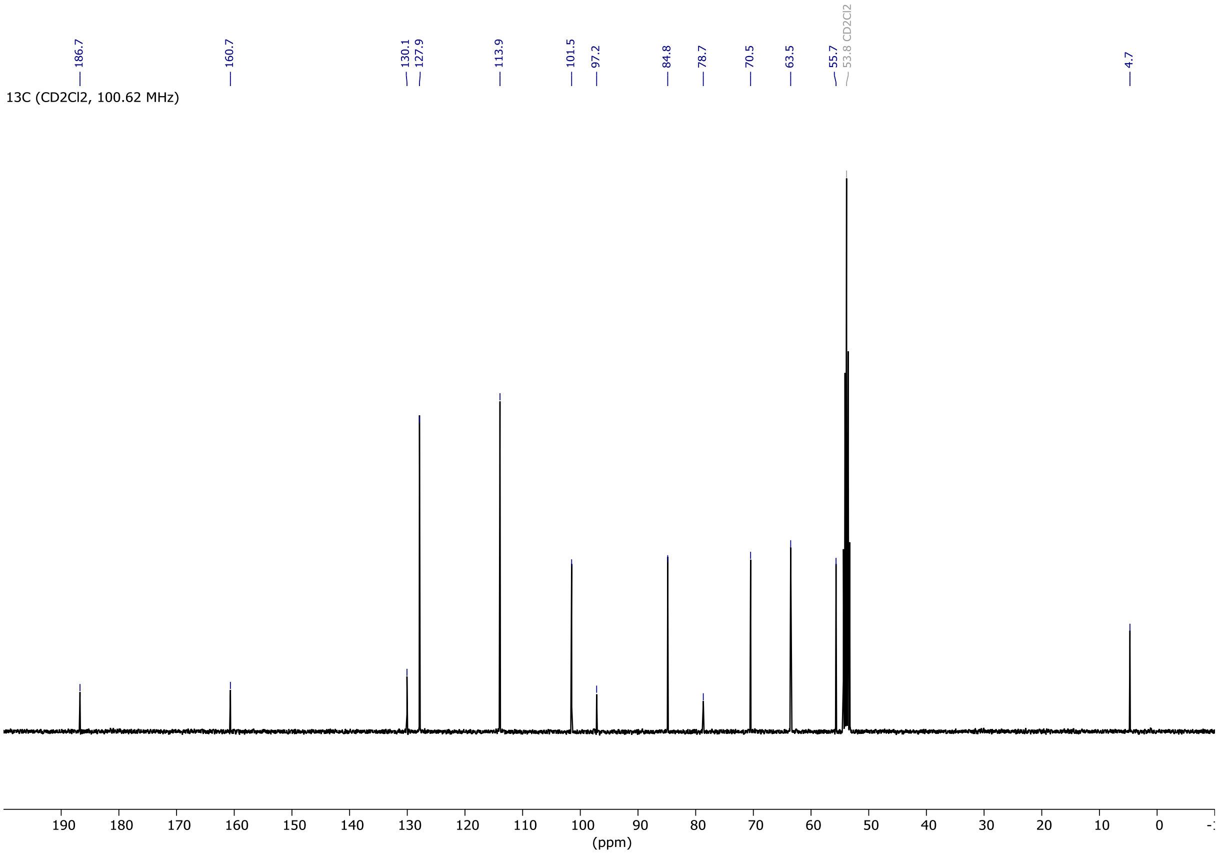

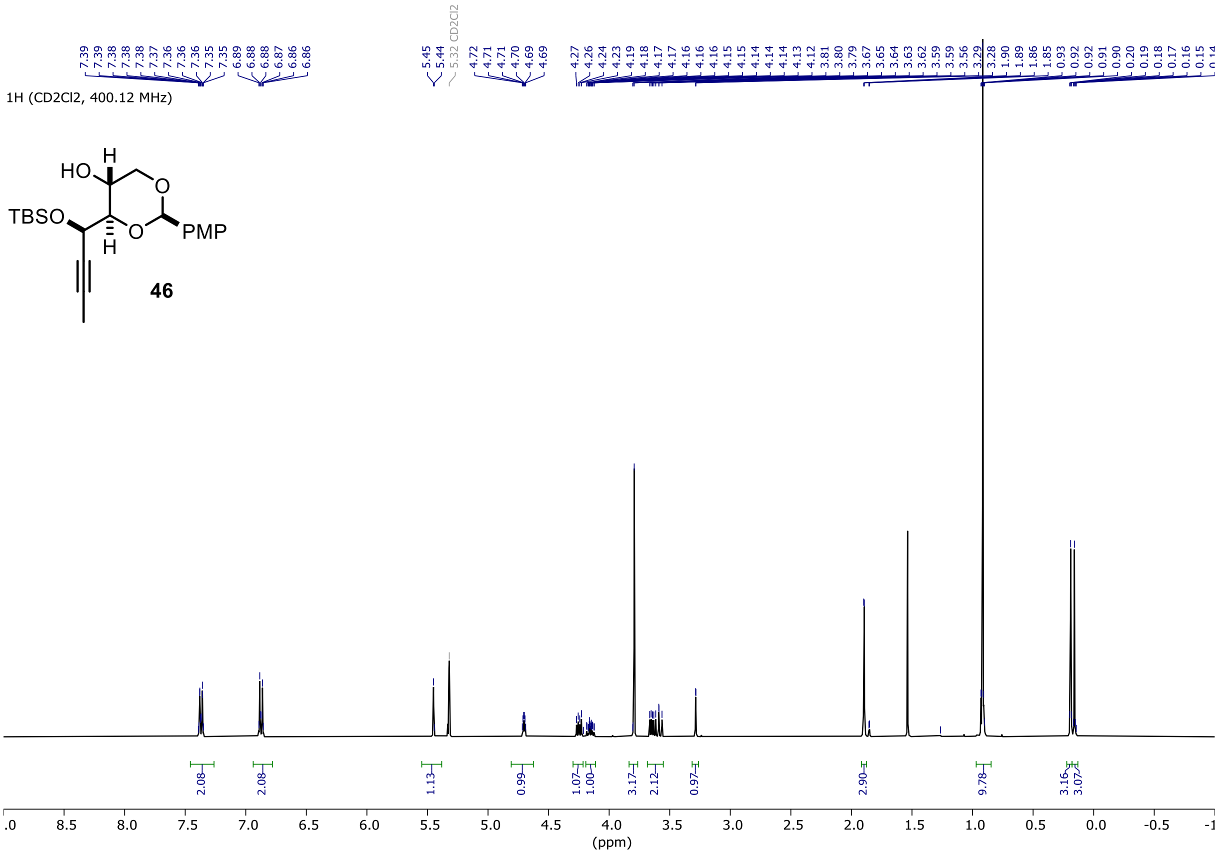
7.39 7.39 7.38 7.38 7.37 7.36 7.36 7.35 6.89 6.88 6.87 6.86 6.86
5.45 5.44 5.32 CD2Cl2 4.72 4.71 4.70 4.69 4.69
4.27 4.26 4.24 4.23 4.19 4.18 4.17 4.17 4.16 4.16 4.15 4.15 4.14 4.14 4.13 4.12 3.81 3.80 3.79 3.67 3.65 3.64 3.63 3.62 3.59 3.56 3.29 3.28 1.90 1.89 1.86 1.85 0.93 0.92 0.92 0.91 0.90 0.20 0.19 0.18 0.17 0.16 0.15
1H (CD2Cl2, 400.12 MHz)
HO
TBSO
PMP
46
2.08 2.08 1.13 0.99 1.07 1.00 3.17 2.12 0.97 2.90 9.78 3.16 3.07
(ppm)

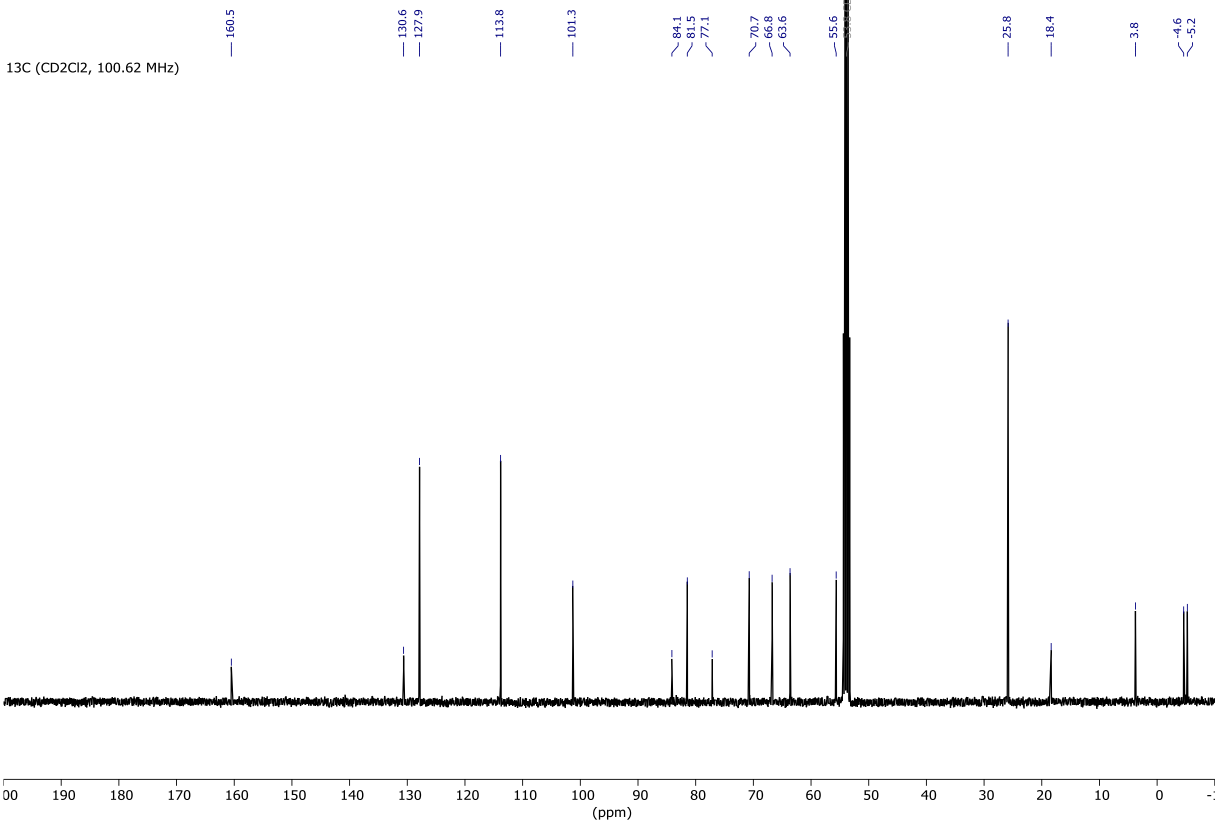
160.5 130.6 127.9 113.8 101.3 84.1 81.5 77.1 70.7 66.8 63.6 55.6 25.8 18.4 3.8 -4.6 -5.2
13C (CD2Cl2, 100.62 MHz)
(ppm)

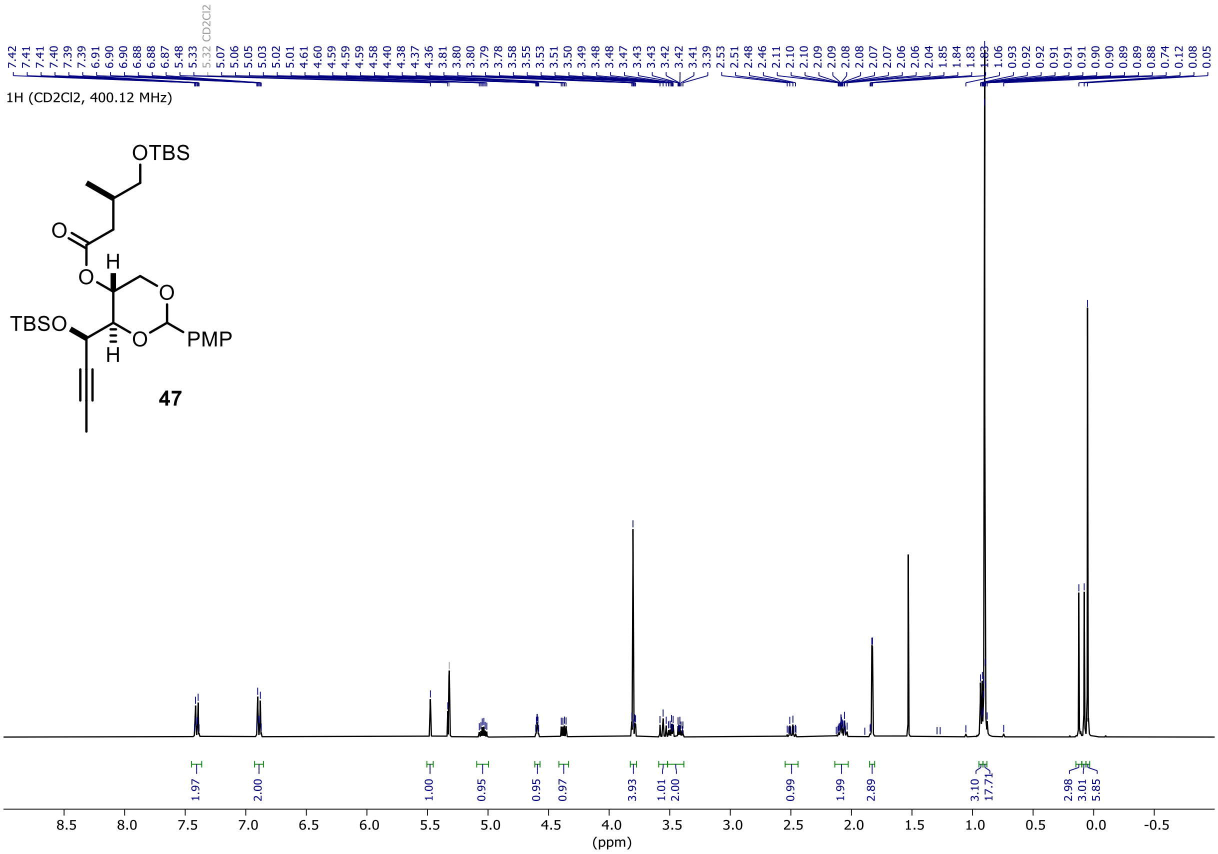
1H (CD2Cl2, 400.12 MHz)
OTBS
O
O
H
O
TBSO
O
H
PMP
47
7.42 7.41 7.41 7.40 7.39 7.39 6.91 6.90 6.90 6.88 6.88 6.87 5.48 5.33 5.32 CD2Cl2 5.07 5.06 5.05 5.03 5.02 5.01 4.61 4.60 4.59 4.59 4.59 4.58 4.40 4.38 4.37 4.36 3.81 3.80 3.80 3.79 3.78 3.58 3.55 3.53 3.51 3.50 3.49 3.48 3.48 3.47 3.43 3.43 3.42 3.42 3.41 3.39 2.53 2.51 2.48 2.46 2.11 2.10 2.10 2.09 2.09 2.08 2.08 2.07 2.07 2.06 2.06 2.04 1.85 1.84 1.83 1.83 1.06 0.93 0.92 0.92 0.91 0.91 0.91 0.90 0.90 0.89 0.88 0.74 0.12 0.08 0.05
1.97 2.00 1.00 0.95 0.95 0.97 3.93 1.01 2.00 0.99 1.99 2.89 3.10 17.71 2.98 3.01 5.85
8.5 8.0 7.5 7.0 6.5 6.0 5.5 5.0 4.5 4.0 3.5 3.0 2.5 2.0 1.5 1.0 0.5 0.0 -0.5
(ppm)

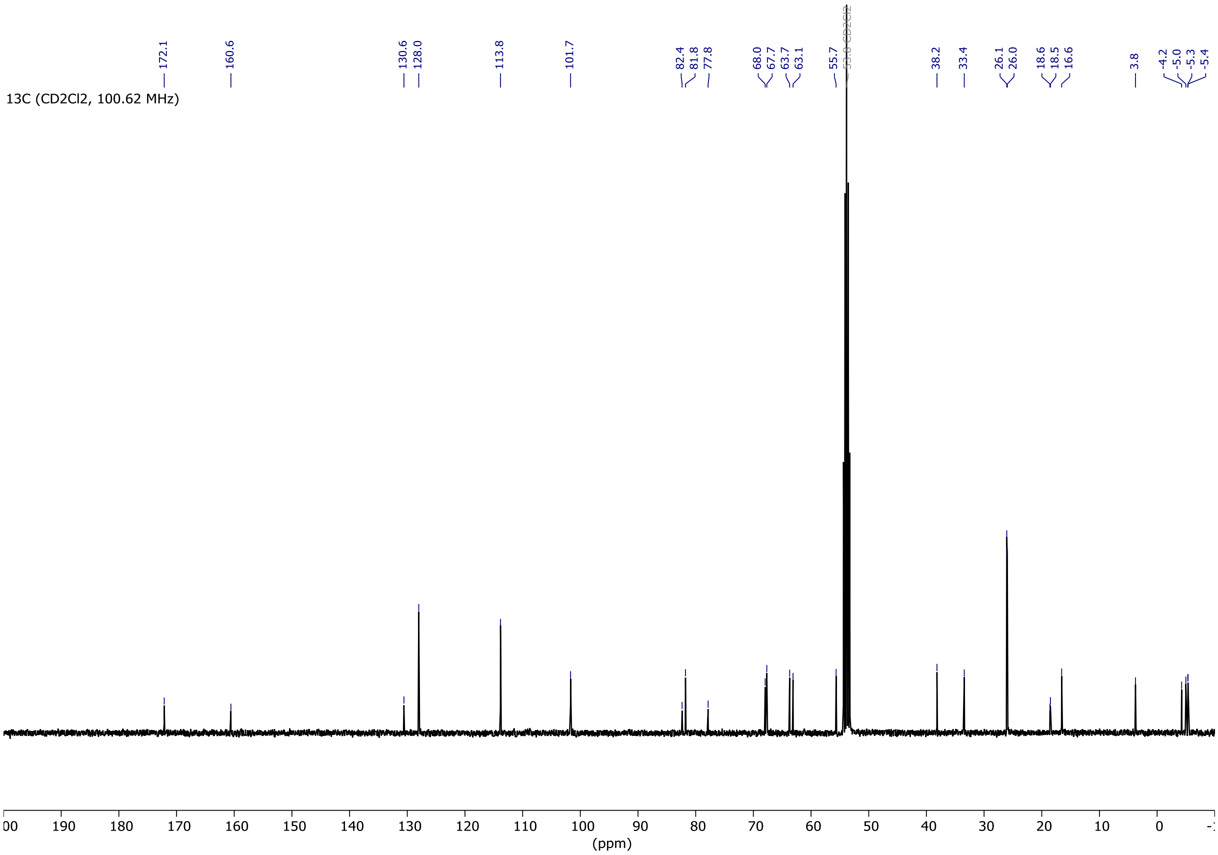
13C (CD2Cl2, 100.62 MHz)
172.1 160.6 130.6 128.0 113.8 101.7 82.4 81.8 77.8 68.0 67.7 63.7 63.1 55.7 53.8 CD2Cl2 38.2 33.4 26.1 26.0 18.6 18.5 16.6 3.8 -4.2 -5.0 -5.3 -5.4
200 190 180 170 160 150 140 130 120 110 100 90 80 70 60 50 40 30 20 10 0
(ppm)

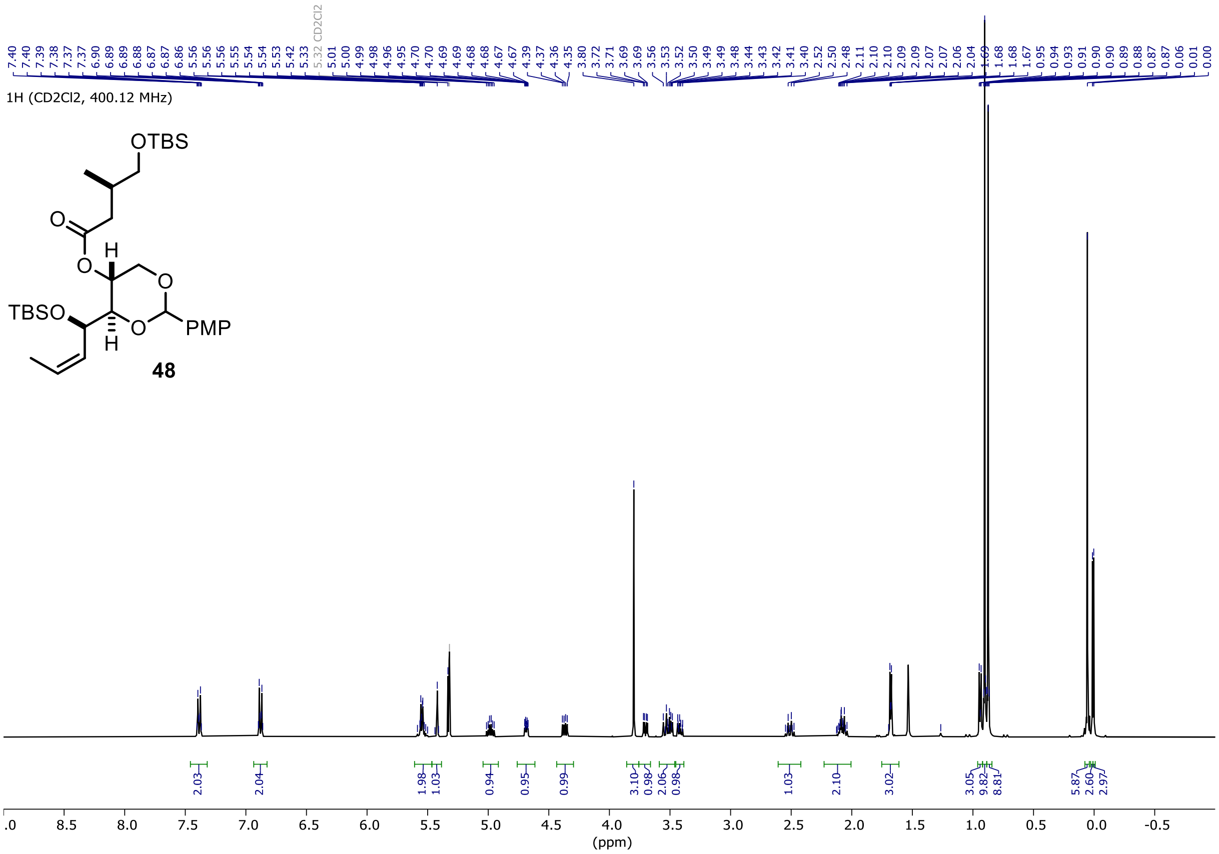
1H (CD2Cl2, 400.12 MHz)
OTBS
O
H
O
O
TBSO
O
PMP
H
48
(ppm)

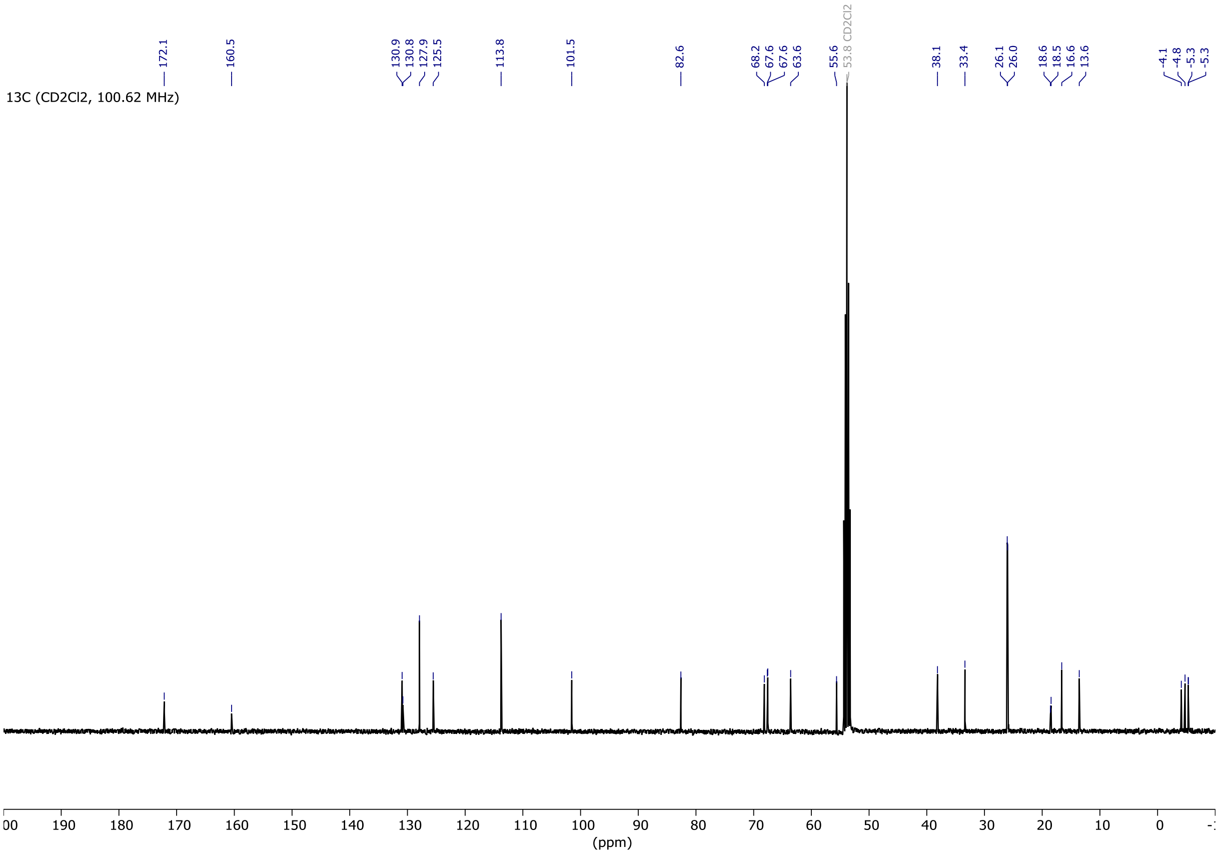
13C (CD2Cl2, 100.62 MHz)
172.1
160.5
130.9
130.8
127.9
125.5
113.8
101.5
82.6
68.2
67.6
67.6
63.6
55.6
53.8 CD2Cl2
38.1
33.4
26.1
26.0
18.6
18.5
16.6
13.6
-4.1
-4.8
-5.3
-5.3
(ppm)

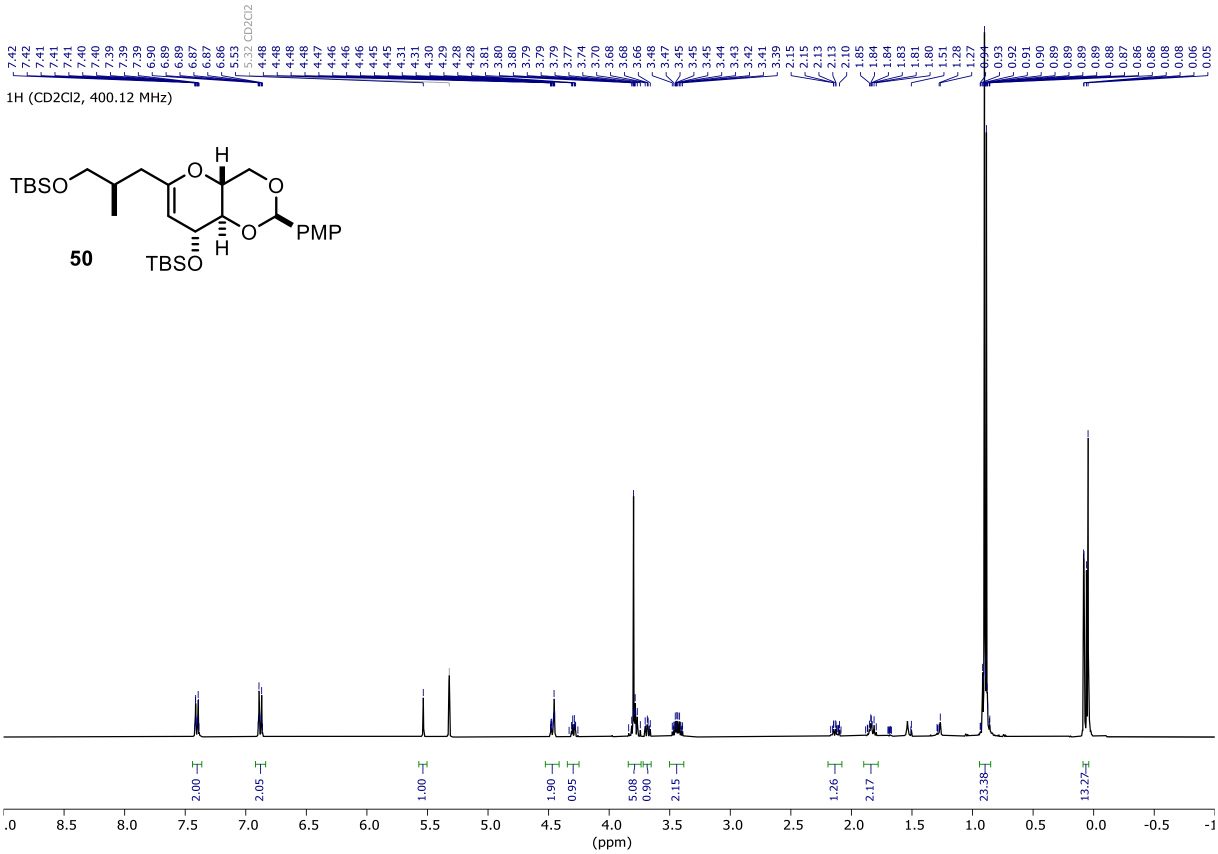

7.42 7.42 7.41 7.41 7.41 7.40 7.40 7.39 7.39 7.39 6.90 6.89 6.89 6.87 6.87 6.86 5.53 5.32 CD2Cl2 4.48 4.48 4.48 4.47 4.46 4.46 4.45 4.45 4.31 4.31 4.30 4.29 4.28 4.28 3.81 3.80 3.80 3.79 3.79 3.77 3.74 3.70 3.68 3.68 3.66 3.48 3.47 3.45 3.45 3.45 3.44 3.43 3.42 3.41 3.39 2.15 2.15 2.13 2.13 2.10 1.85 1.84 1.84 1.83 1.81 1.80 1.51 1.28 1.27 0.94 0.93 0.92 0.91 0.90 0.89 0.89 0.89 0.89 0.88 0.87 0.86 0.86 0.08 0.06 0.05
1H (CD2Cl2, 400.12 MHz)
TBSO
TBSO
PMP
50
2.00
2.05
1.00
1.90
0.95
5.08
0.90
2.15
1.26
2.17
23.38
13.27
.0 8.5 8.0 7.5 7.0 6.5 6.0 5.5 5.0 4.5 4.0 3.5 3.0 2.5 2.0 1.5 1.0 0.5 0.0 -0.5 -1
(ppm)

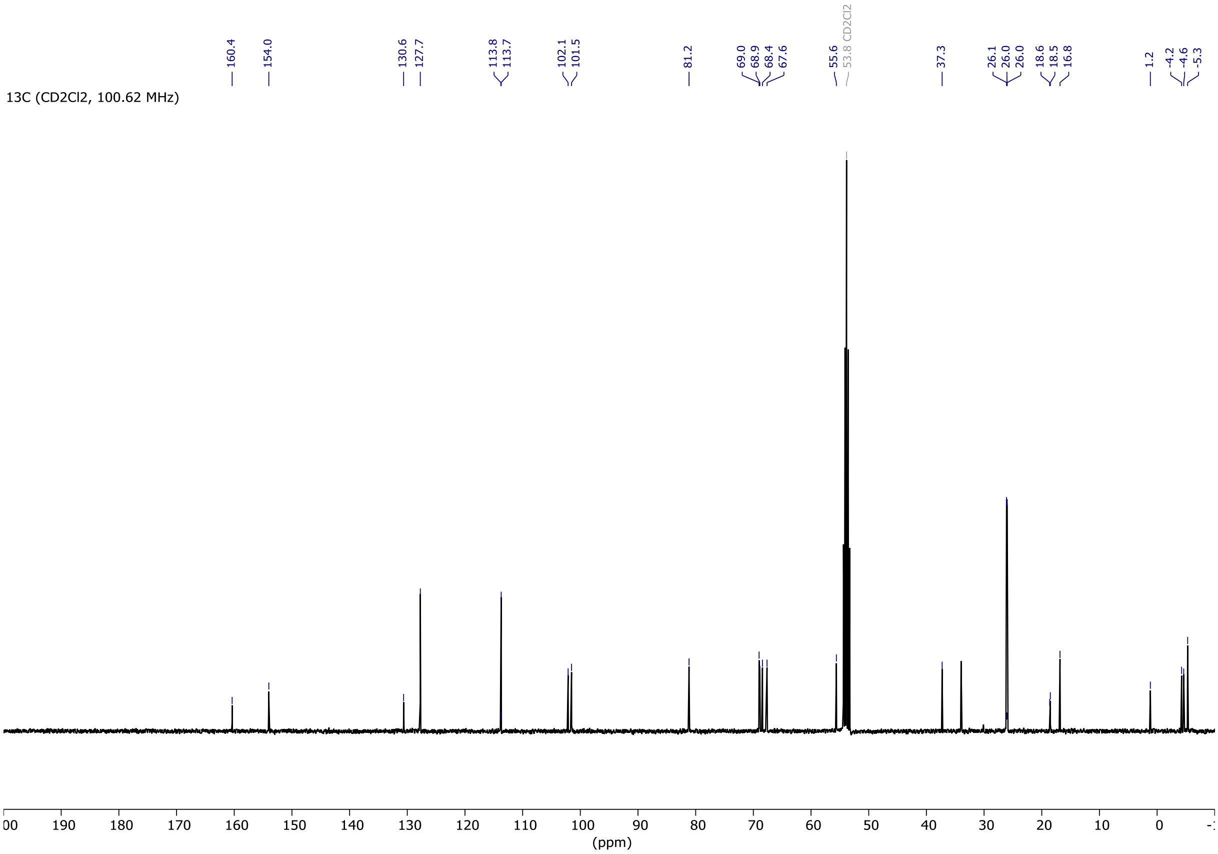

160.4
154.0
130.6
127.7
113.8
113.7
102.1
101.5
81.2
69.0
68.9
68.4
67.6
55.6
53.8 CD2Cl2
37.3
26.1
26.0
26.0
18.6
18.5
16.8
1.2
-4.2
-4.6
-5.3
13C (CD2Cl2, 100.62 MHz)
00 190 180 170 160 150 140 130 120 110 100 90 80 70 60 50 40 30 20 10 0
(ppm)

1H (CD2Cl2, 400.12 MHz)

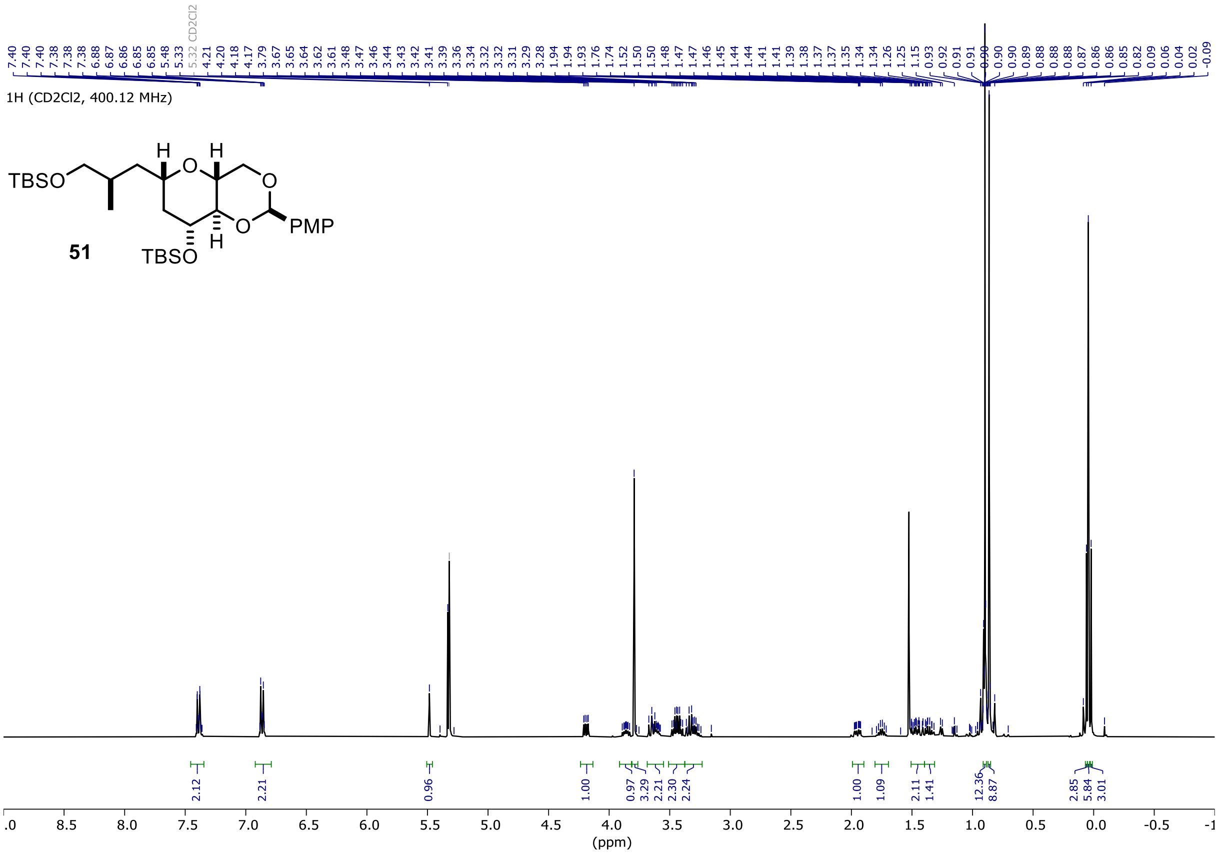

13C (CD2Cl2, 100.62 MHz)

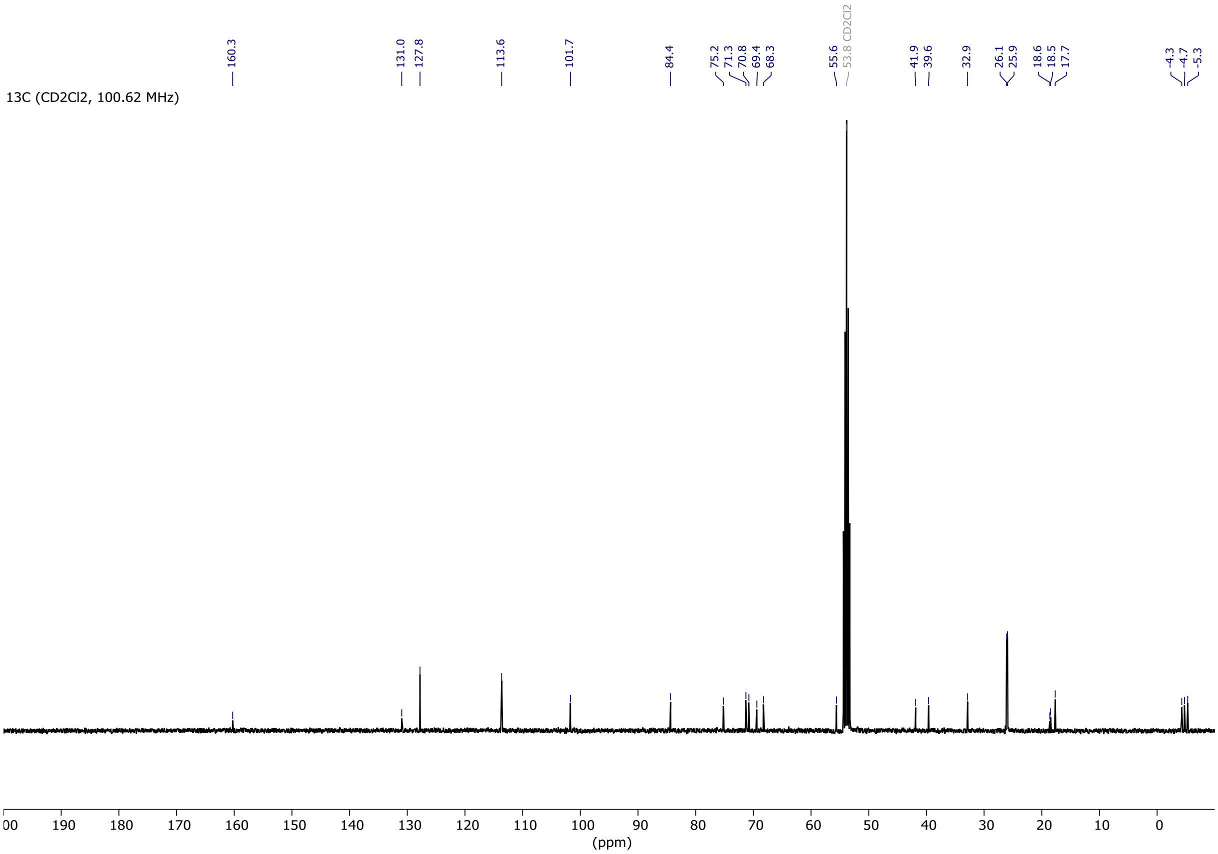

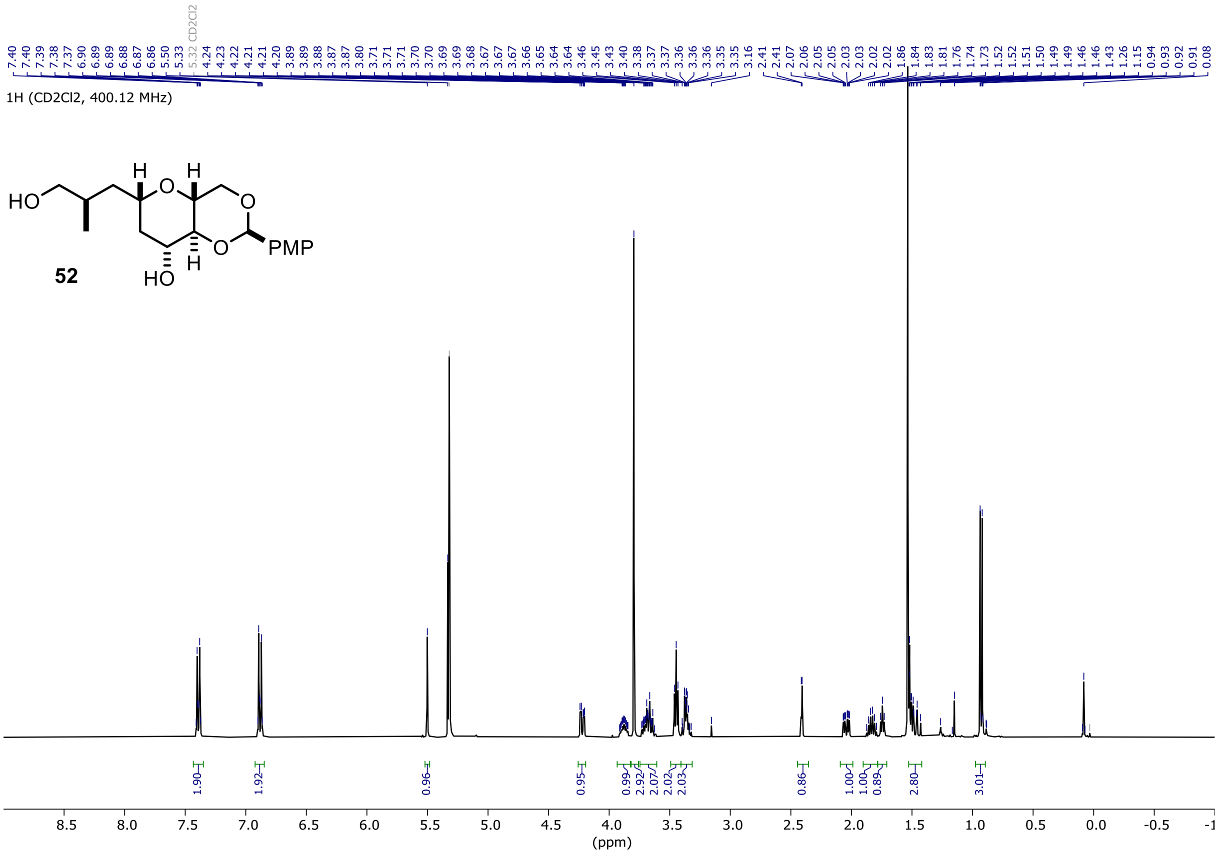
7.40 7.40 7.39 7.38 7.37 6.90 6.89 6.89 6.88 6.87 6.86 5.50 5.33 5.32 CD2Cl2 4.24 4.23 4.22 4.21 4.21 4.20 3.89 3.89 3.88 3.87 3.80 3.71 3.71 3.71 3.70 3.69 3.69 3.68 3.67 3.67 3.67 3.66 3.65 3.64 3.64 3.46 3.45 3.43 3.40 3.38 3.37 3.37 3.36 3.36 3.36 3.35 3.35 3.16 2.41 2.07 2.06 2.05 2.05 2.03 2.03 2.02 2.02 1.86 1.84 1.83 1.81 1.76 1.74 1.73 1.52 1.52 1.51 1.50 1.49 1.46 1.43 1.26 1.15 0.94 0.93 0.92 0.91 0.08
1H (CD2Cl2, 400.12 MHz)
HO
H
O
H
O
O
PMP
HO
H
52
1.90
1.92
0.96
0.95
0.99
2.92
2.07
2.02
2.03
0.86
1.00
1.00
0.89
2.80
3.01
8.5 8.0 7.5 7.0 6.5 6.0 5.5 5.0 4.5 4.0 3.5 3.0 2.5 2.0 1.5 1.0 0.5 0.0 -0.5 -1
(ppm)

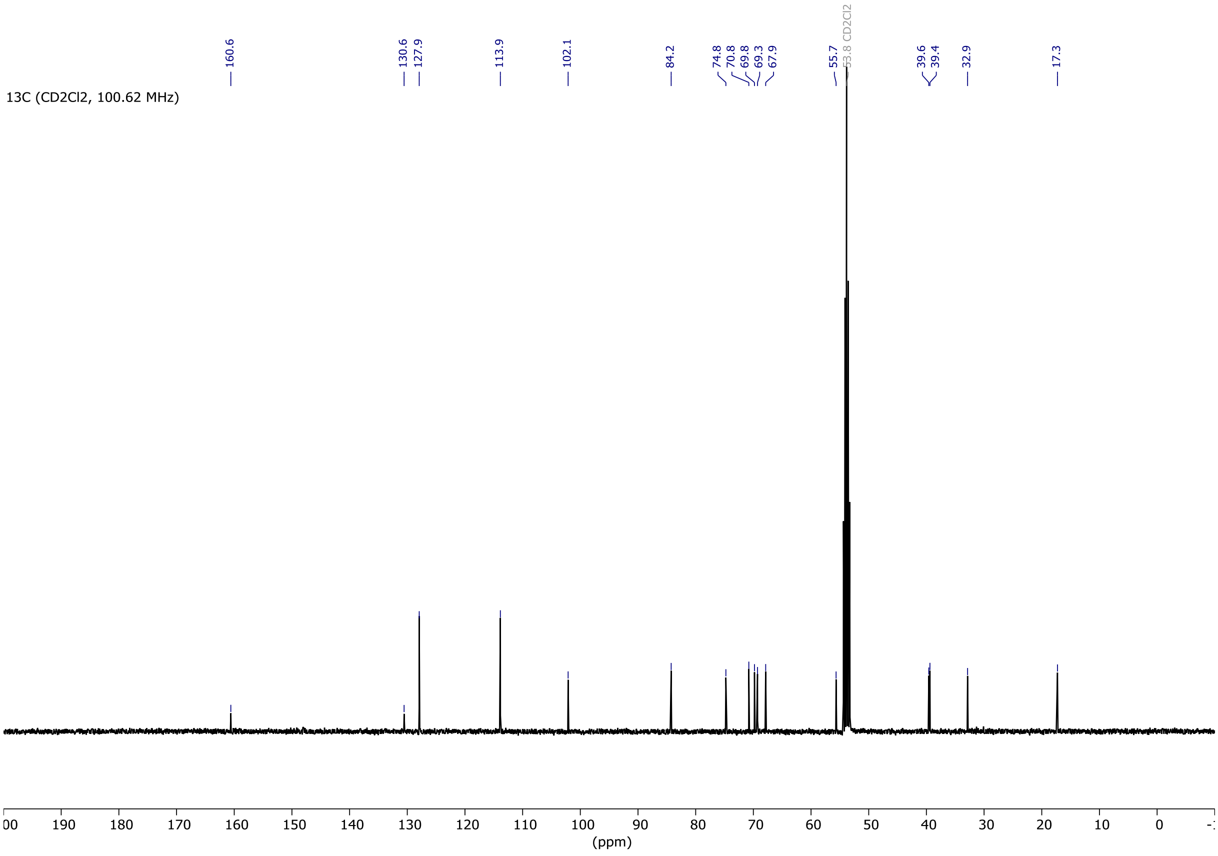
160.6
130.6
127.9
113.9
102.1
84.2
74.8
70.8
69.8
69.3
67.9
55.7
53.8 CD2Cl2
39.6
39.4
32.9
17.3
13C (CD2Cl2, 100.62 MHz)
00 190 180 170 160 150 140 130 120 110 100 90 80 70 60 50 40 30 20 10 0
(ppm)

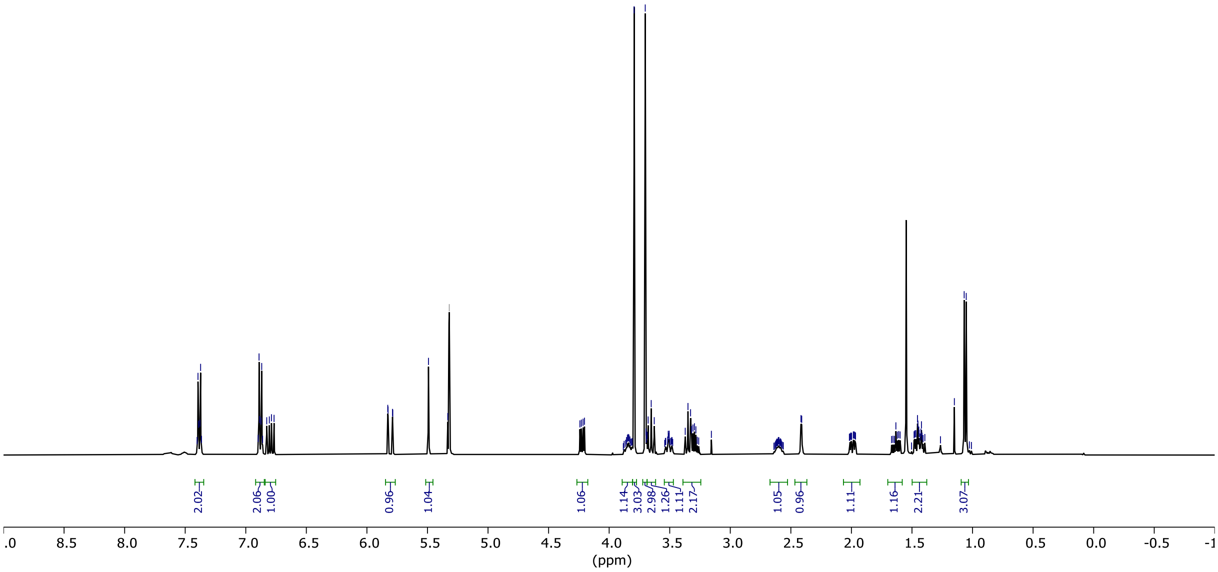
7.40 7.39 7.39 7.38 7.37 7.37 6.90 6.89 6.89 6.87 6.86 6.83 6.81 6.79 6.77 5.83 5.83 5.79 5.79 5.49 5.33 5.32 CD2Cl2 4.24 4.23 4.22 4.20 3.85 3.84 3.84 3.83 3.79 3.70 3.69 3.69 3.68 3.65 3.63 3.52 3.51 3.51 3.50 3.48 3.37 3.35 3.33 3.32 3.31 3.30 3.28 3.27 3.16 2.42 2.41 2.02 2.01 2.00 1.98 1.98 1.97 1.97 1.67 1.66 1.64 1.63 1.62 1.61 1.60 1.48 1.47 1.45 1.44 1.43 1.42 1.41 1.39 1.26 1.15 1.07 1.05
1H (CD2Cl2, 400.12 MHz)
2.02 2.06 1.00 0.96 1.04 1.06 1.14 3.03 2.98 1.26 1.11 2.17 1.05 0.96 1.11 1.16 2.21 3.07
(ppm)

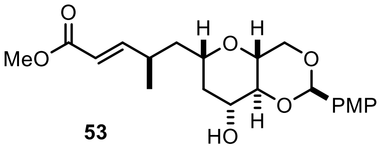
MeO
O
H
H
O
O
O
H
HO
PMP

53

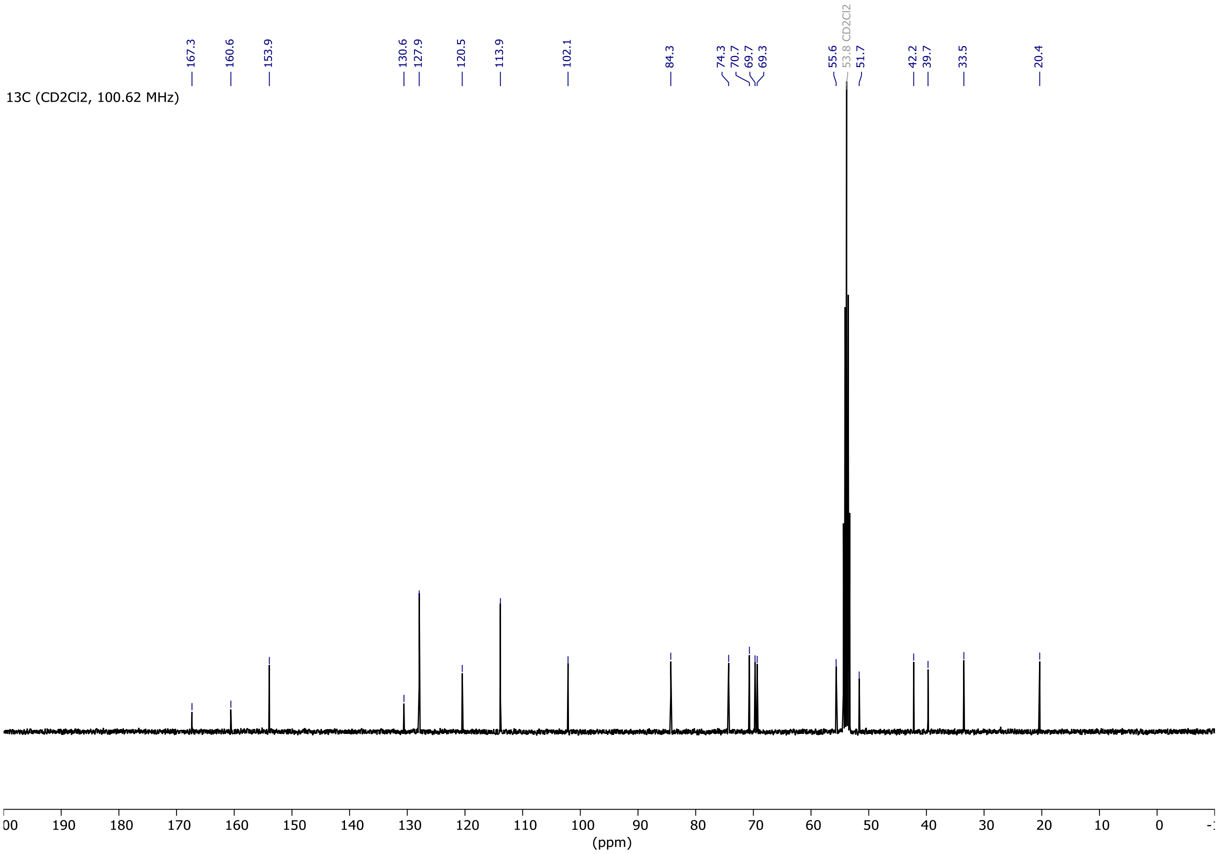
167.3 160.6 153.9 130.6 127.9 120.5 113.9 102.1 84.3 74.3 70.7 69.7 69.3 55.6 53.8 CD2Cl2 51.7 42.2 39.7 33.5 20.4
13C (CD2Cl2, 100.62 MHz)
(ppm)

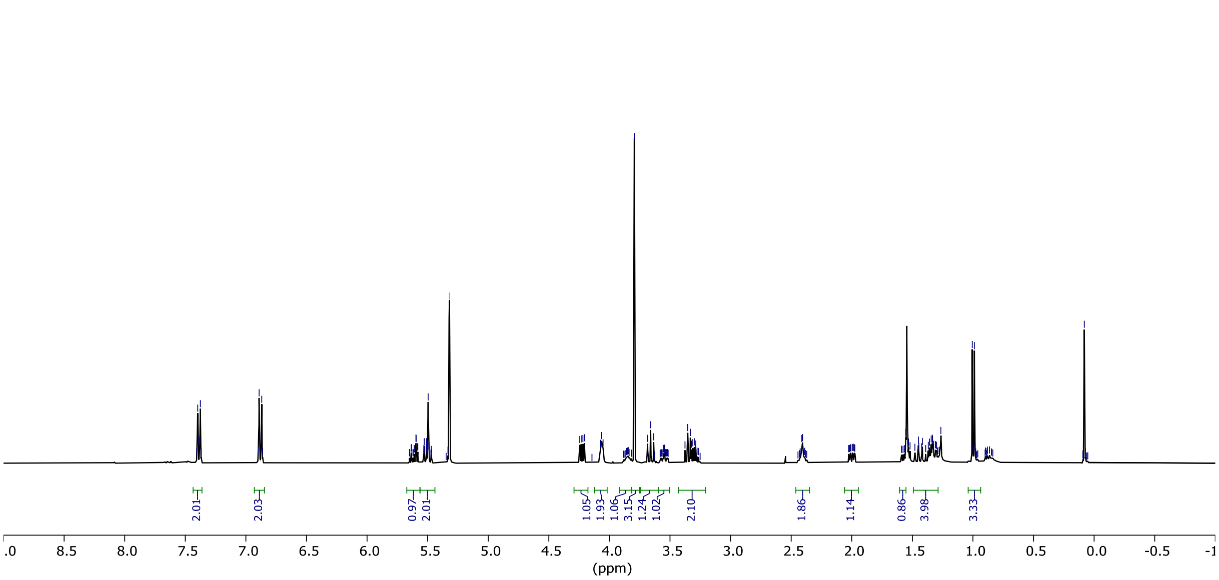
1H (CD2Cl2, 400.12 MHz)
(ppm)

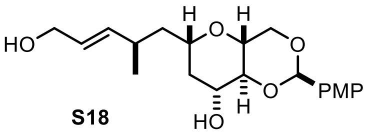
HO
O
O
O
PMP
HO
H
H
H

S18

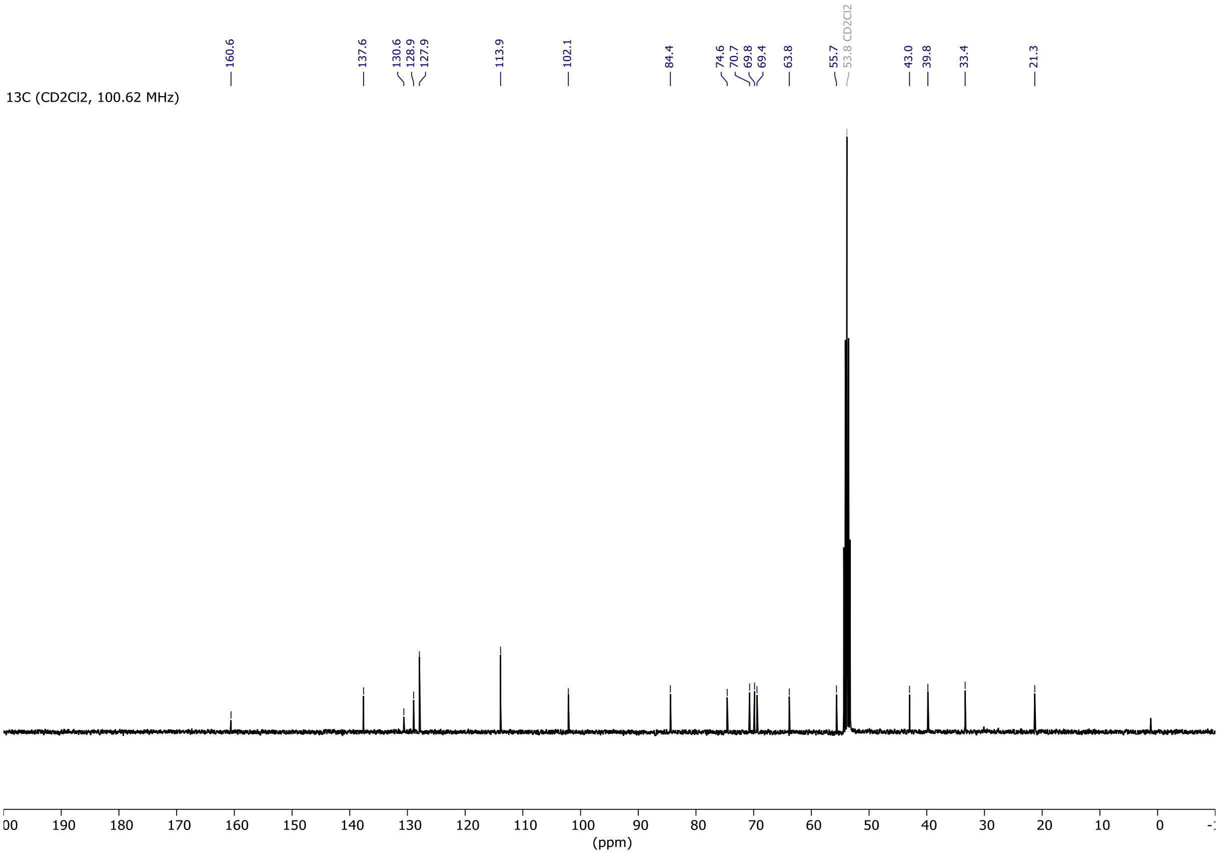
160.6
137.6
130.6
128.9
127.9
113.9
102.1
84.4
74.6
70.7
69.8
69.4
63.8
55.7
53.8 CD2Cl2
43.0
39.8
33.4
21.3
13C (CD2Cl2, 100.62 MHz)
(ppm)

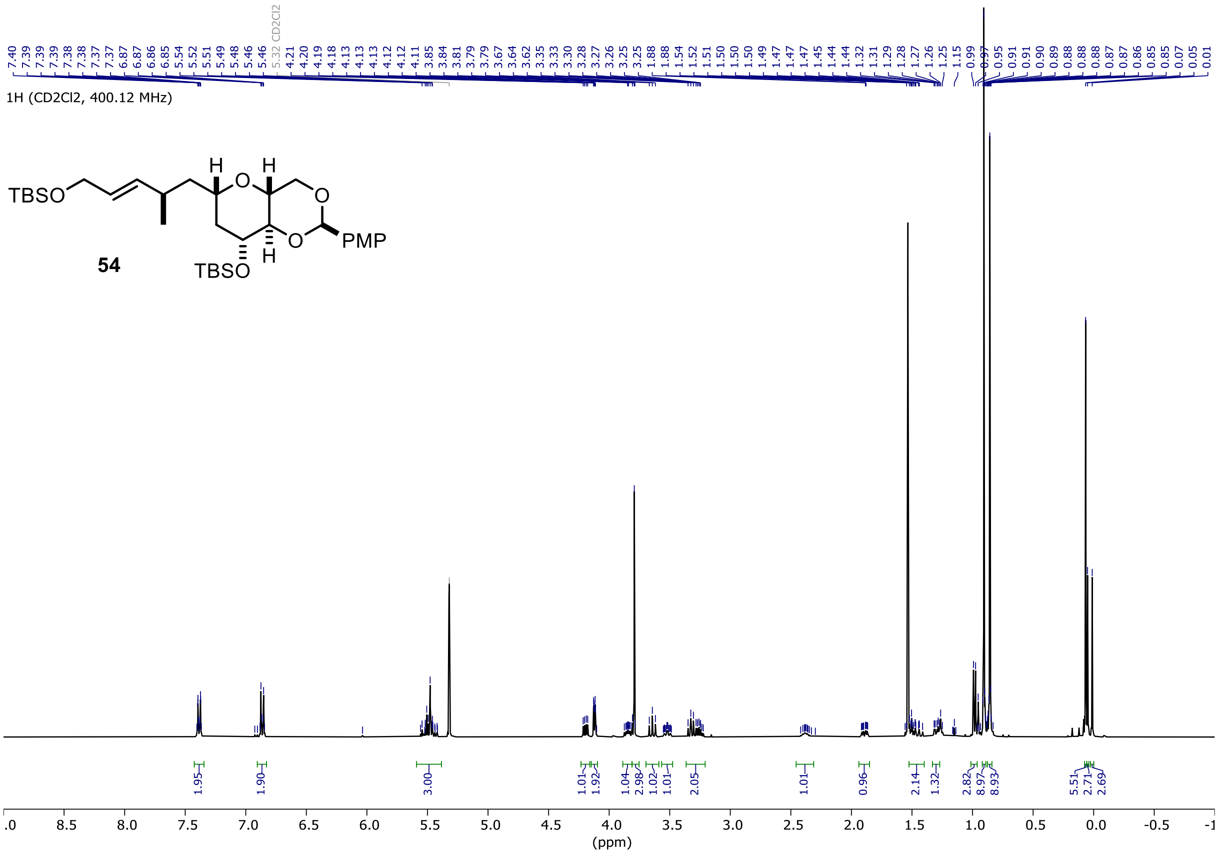
1H (CD2Cl2, 400.12 MHz)
TBSO
TBSO
PMP
54
(ppm)

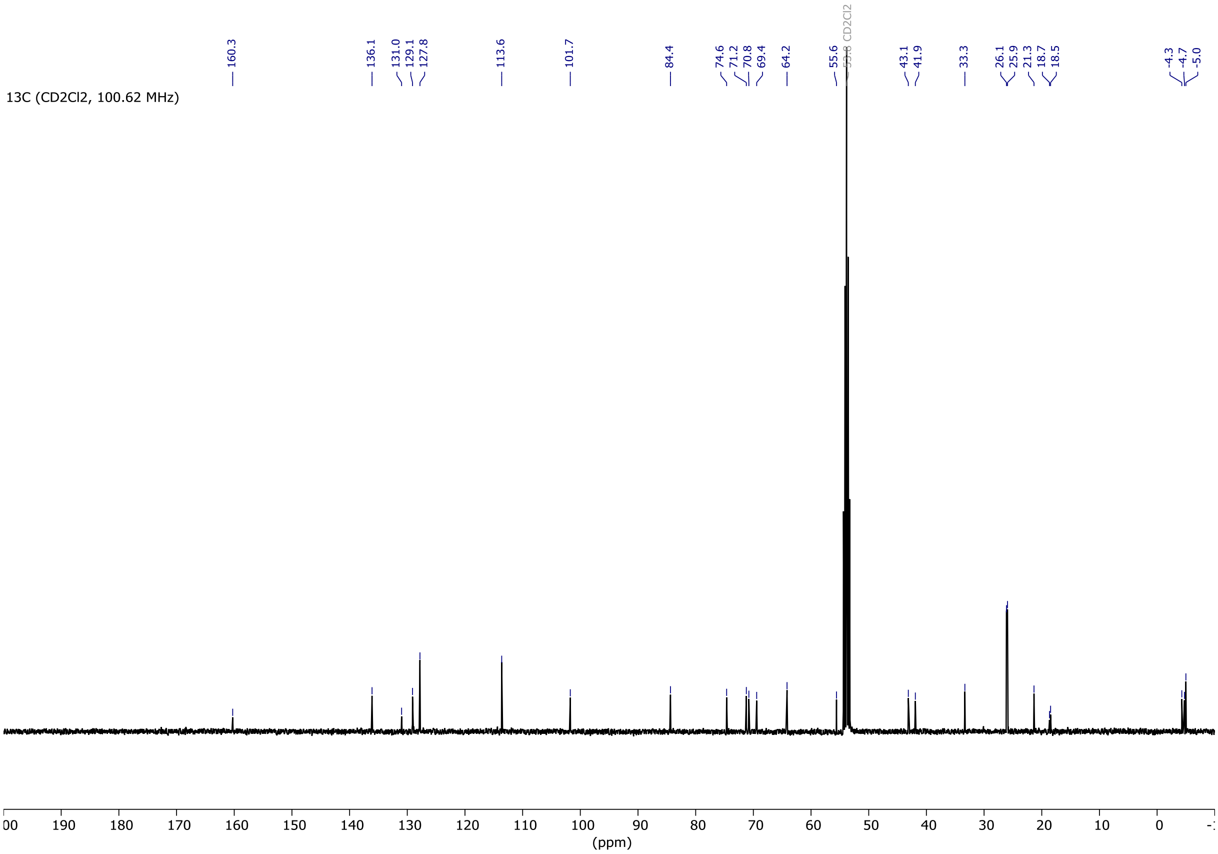
13C (CD2Cl2, 100.62 MHz)
160.3
136.1
131.0
129.1
127.8
113.6
101.7
84.4
74.6
71.2
70.8
69.4
64.2
55.6
43.1
41.9
33.3
26.1
25.9
21.3
18.7
18.5
-4.3
-4.7
-5.0
(ppm)

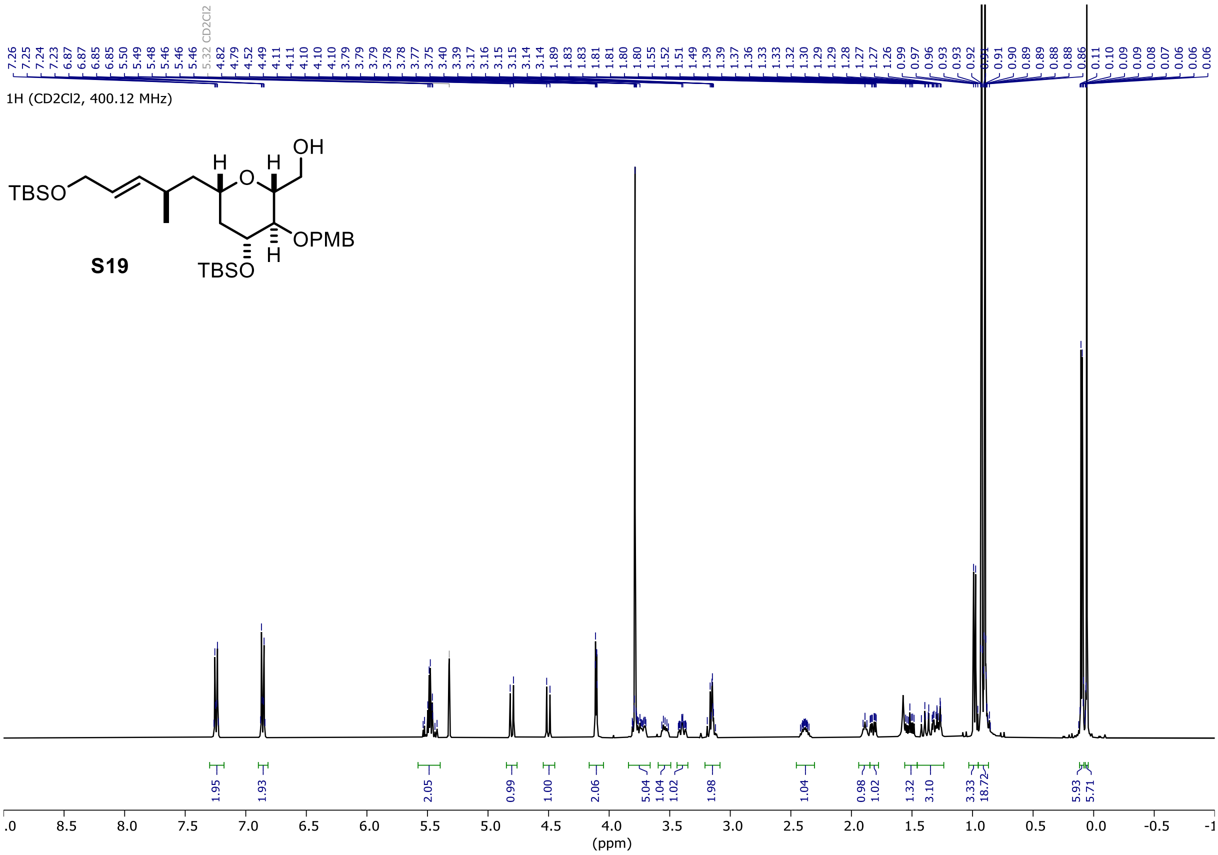
1H (CD2Cl2, 400.12 MHz)
S19
(ppm)

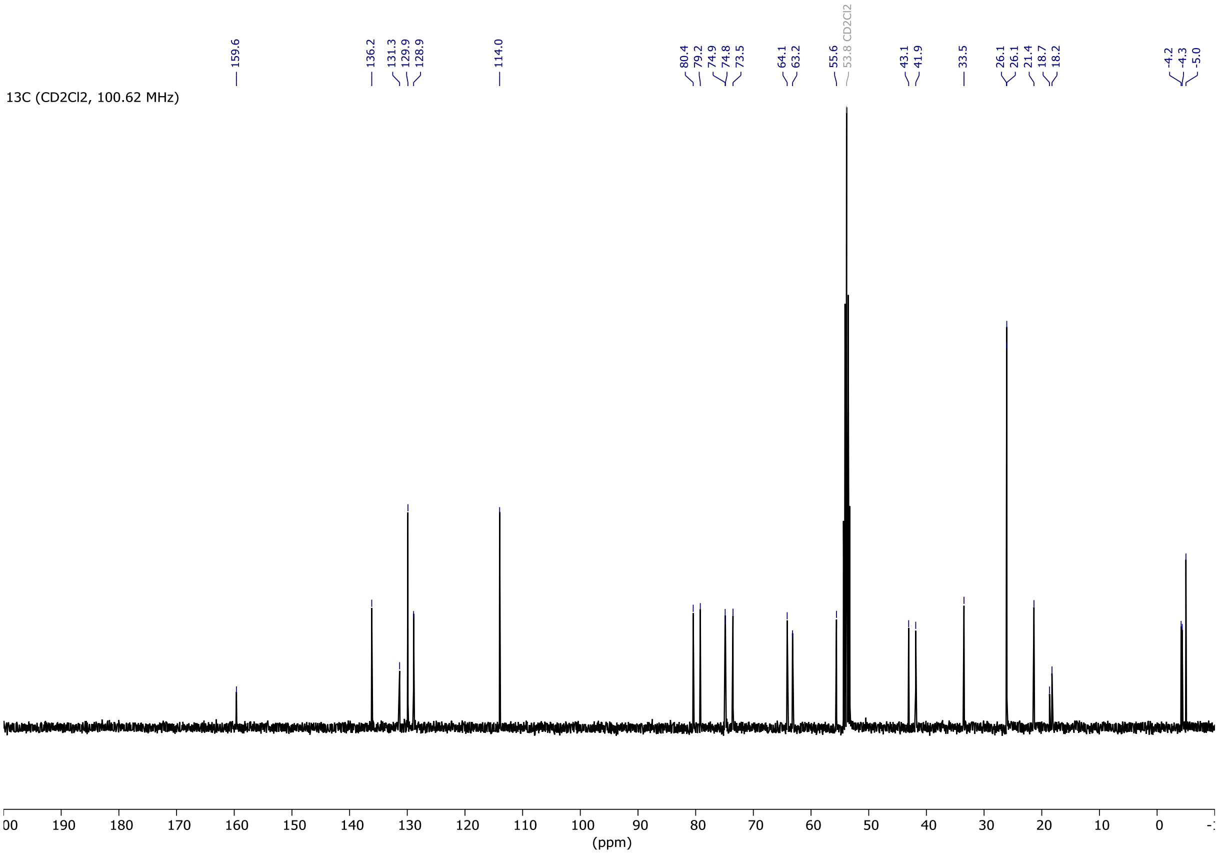
13C (CD2Cl2, 100.62 MHz)
159.6
136.2
131.3
129.9
128.9
114.0
80.4
79.2
74.9
74.8
73.5
64.1
63.2
55.6
53.8 CD2Cl2
43.1
41.9
33.5
26.1
26.1
21.4
18.7
18.2
-4.2
-4.3
-5.0
(ppm)

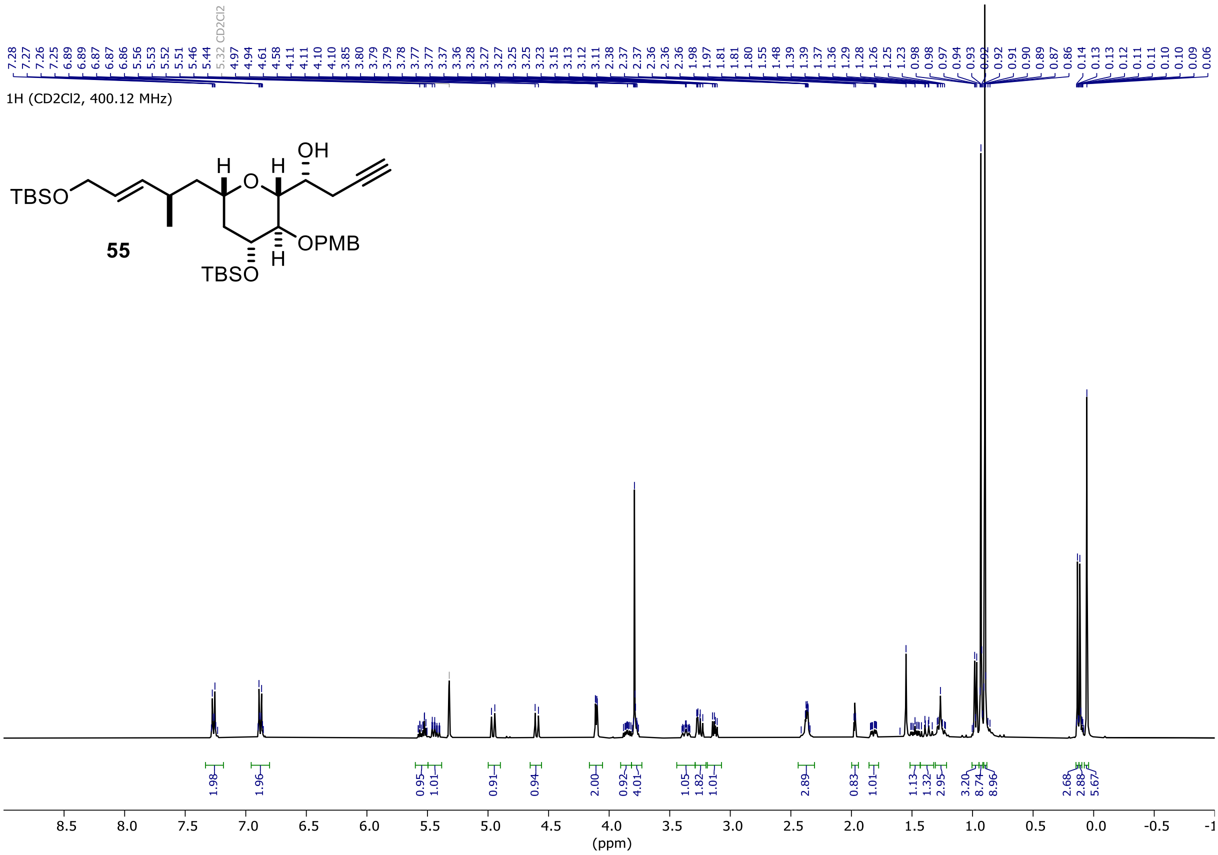
7.28 7.27 7.26 7.25 6.89 6.87 6.87 6.86 5.56 5.53 5.52 5.51 5.46 5.44 5.32 CD2Cl2 4.97 4.94 4.61 4.58 4.11 4.11 4.10 4.10 3.85 3.80 3.79 3.79 3.78 3.77 3.77 3.37 3.36 3.28 3.27 3.27 3.25 3.25 3.23 3.15 3.13 3.13 3.12 3.11 2.38 2.37 2.37 2.36 2.36 1.98 1.97 1.81 1.81 1.80 1.55 1.48 1.39 1.39 1.37 1.36 1.29 1.28 1.26 1.25 1.23 0.98 0.98 0.97 0.94 0.93 0.92 0.92 0.91 0.90 0.89 0.87 0.86 0.14 0.13 0.13 0.12 0.11 0.11 0.10 0.10 0.09 0.06
1H (CD2Cl2, 400.12 MHz)
TBSO
OH
H
H
O
OPMB
H
TBSO
55
1.98 1.96 0.95 1.01 0.91 0.94 2.00 0.92 4.01 1.05 1.82 1.01 2.89 0.83 1.01 1.13 1.32 2.95 3.20 8.74 8.96 2.68 2.88 5.67
8.5 8.0 7.5 7.0 6.5 6.0 5.5 5.0 4.5 4.0 3.5 3.0 2.5 2.0 1.5 1.0 0.5 0.0 -0.5
(ppm)

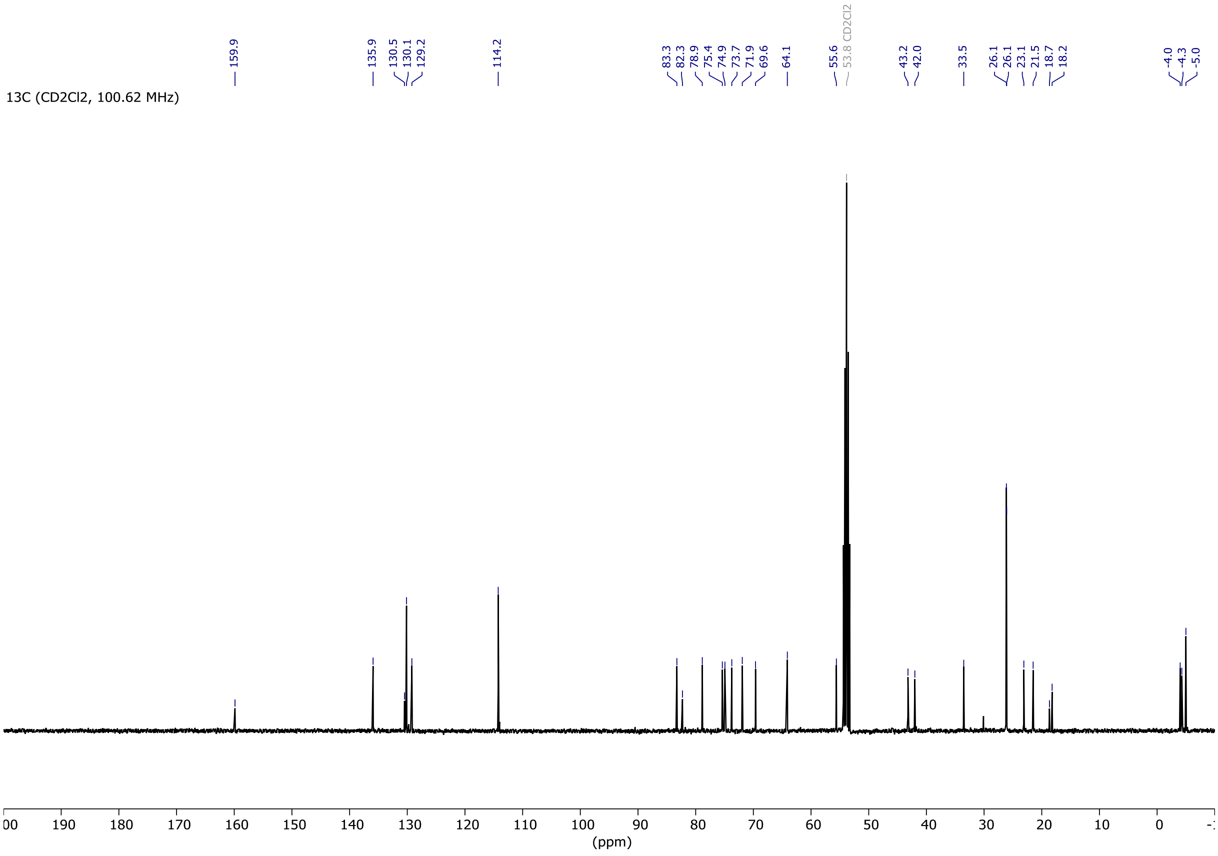
159.9 135.9 130.5 130.1 129.2 114.2 83.3 82.3 78.9 75.4 74.9 73.7 71.9 69.6 64.1 55.6 53.8 CD2Cl2 43.2 42.0 33.5 26.1 26.1 23.1 21.5 18.7 18.2 -4.0 -4.3 -5.0
13C (CD2Cl2, 100.62 MHz)
190 180 170 160 150 140 130 120 110 100 90 80 70 60 50 40 30 20 10 0
(ppm)

1H (CD2Cl2, 600.20 MHz)

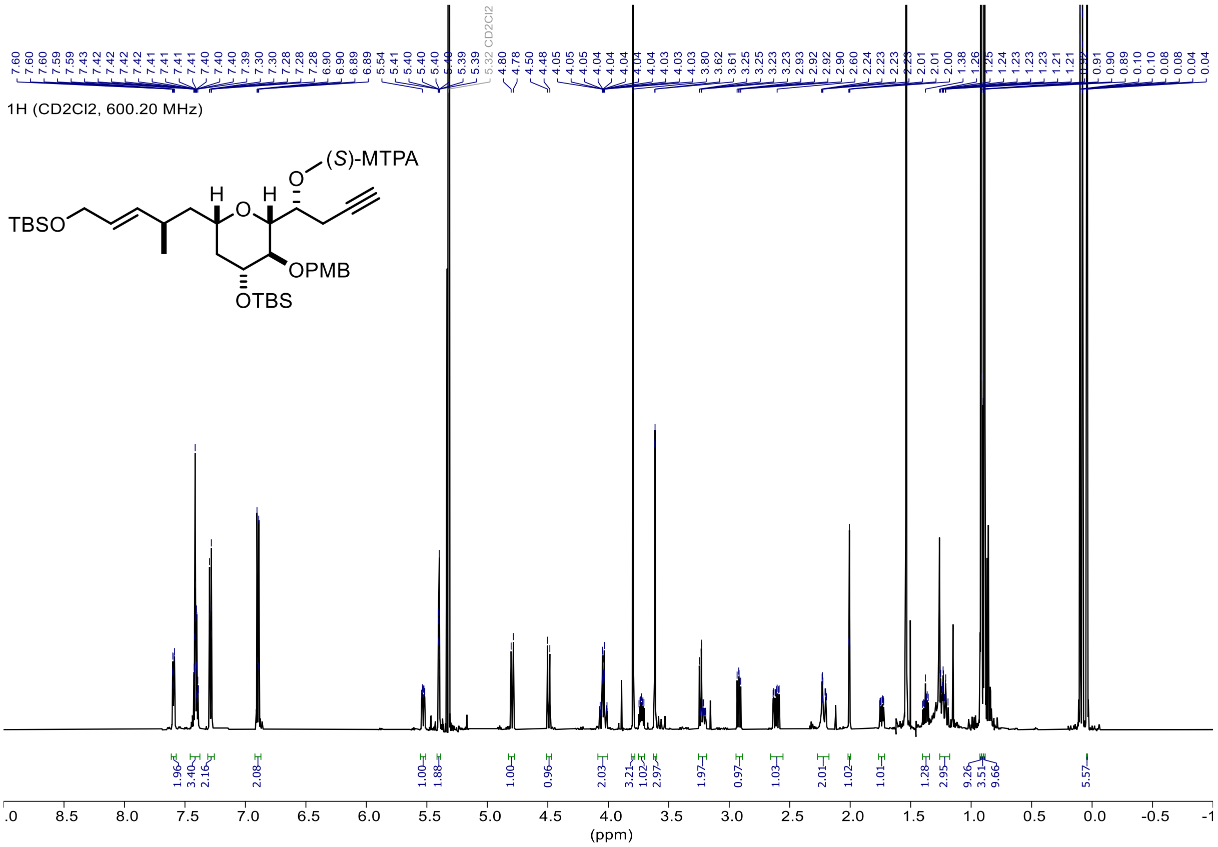

1H (CD2Cl2, 600.20 MHz)

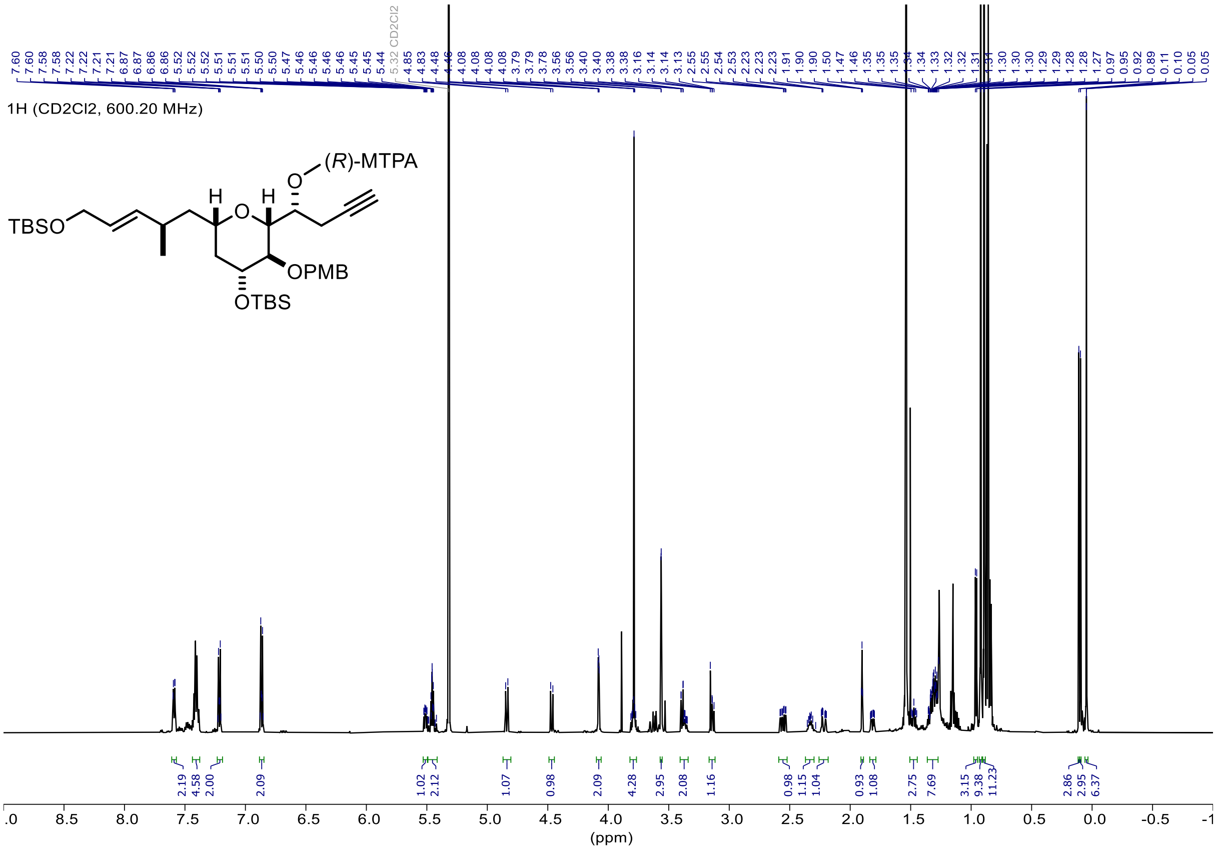

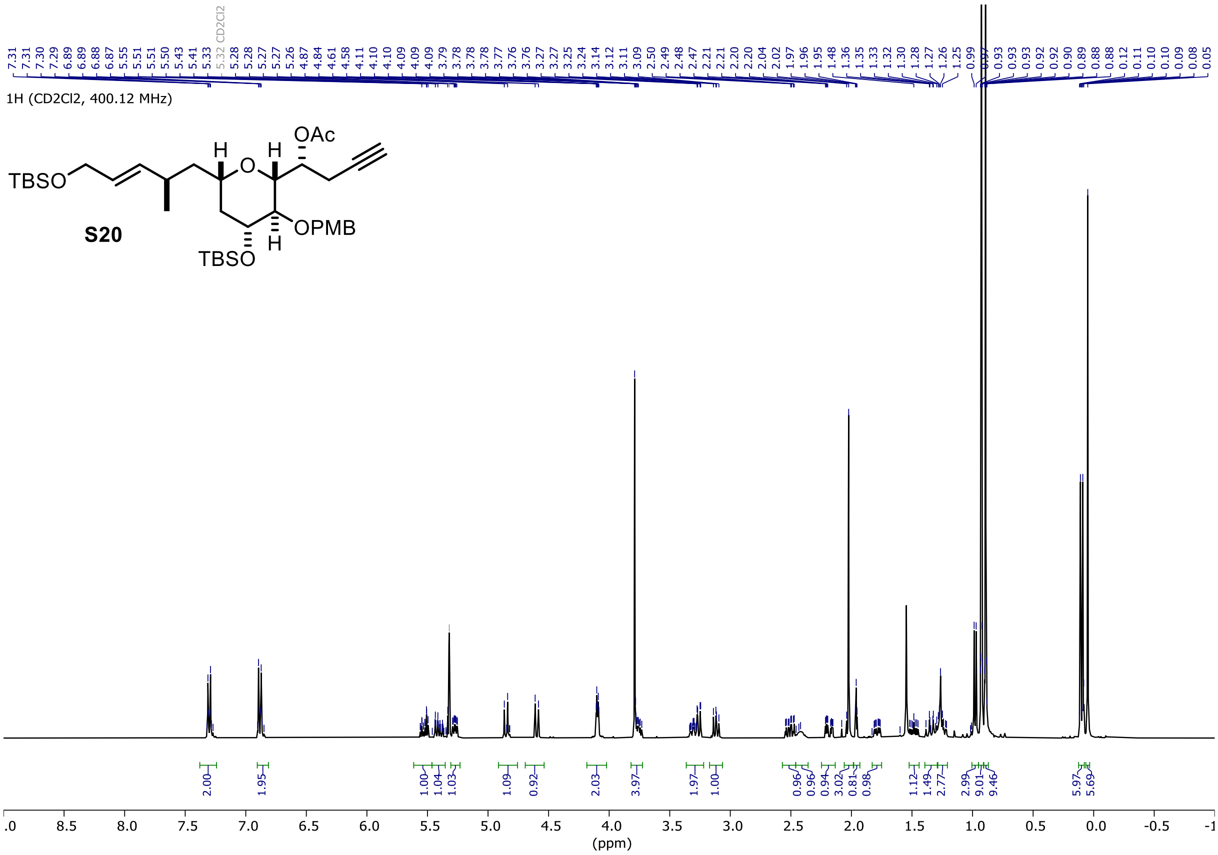
7.31 7.31 7.30 7.29 6.89 6.89 6.88 6.87 5.55 5.51 5.51 5.50 5.43 5.41 5.33 5.32 CD2Cl2 5.28 5.28 5.27 5.26 4.87 4.84 4.61 4.58 4.11 4.10 4.09 4.09 4.09 3.79 3.78 3.78 3.77 3.76 3.76 3.27 3.27 3.25 3.24 3.14 3.12 3.11 3.09 2.50 2.49 2.48 2.47 2.21 2.21 2.20 2.20 2.04 2.02 1.97 1.96 1.95 1.48 1.36 1.35 1.33 1.32 1.30 1.28 1.27 1.26 1.25 0.99 0.97 0.93 0.93 0.93 0.92 0.92 0.90 0.89 0.88 0.88 0.12 0.11 0.10 0.10 0.09 0.08 0.05
1H (CD2Cl2, 400.12 MHz)
OAc
H
O
H
TBSO
OPMB
S20
TBSO
H
2.00 1.95 1.00 1.04 1.03 1.09 0.92 2.03 3.97 1.97 1.00 0.96 0.96 0.94 3.02 0.81 0.98 1.12 1.49 2.77 2.99 9.01 9.46 5.97 5.69
(ppm)

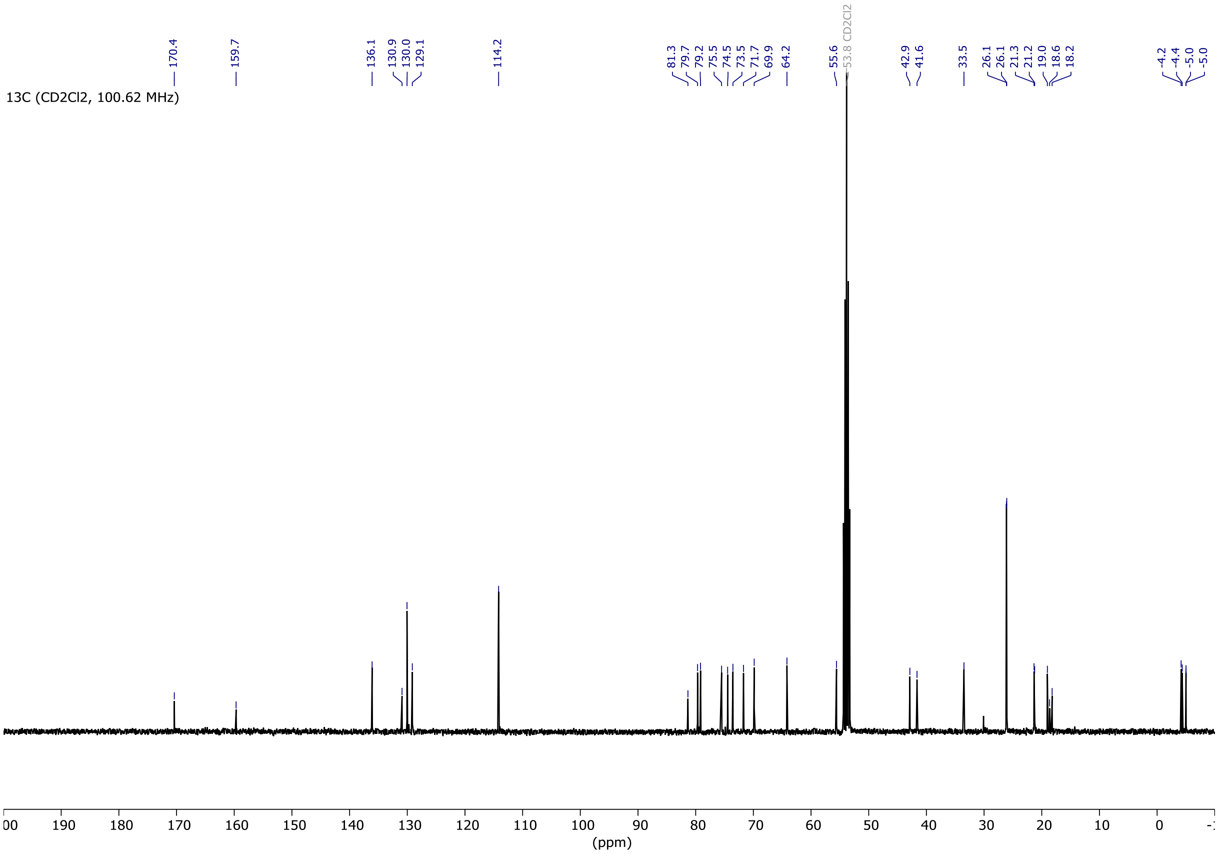
170.4 159.7 136.1 130.9 130.0 129.1 114.2 81.3 79.7 79.2 75.5 74.5 73.5 71.7 69.9 64.2 55.6 53.8 CD2Cl2 42.9 41.6 33.5 26.1 26.1 21.3 21.2 19.0 18.6 18.2 -4.2 -4.4 -5.0 -5.0
13C (CD2Cl2, 100.62 MHz)
(ppm)

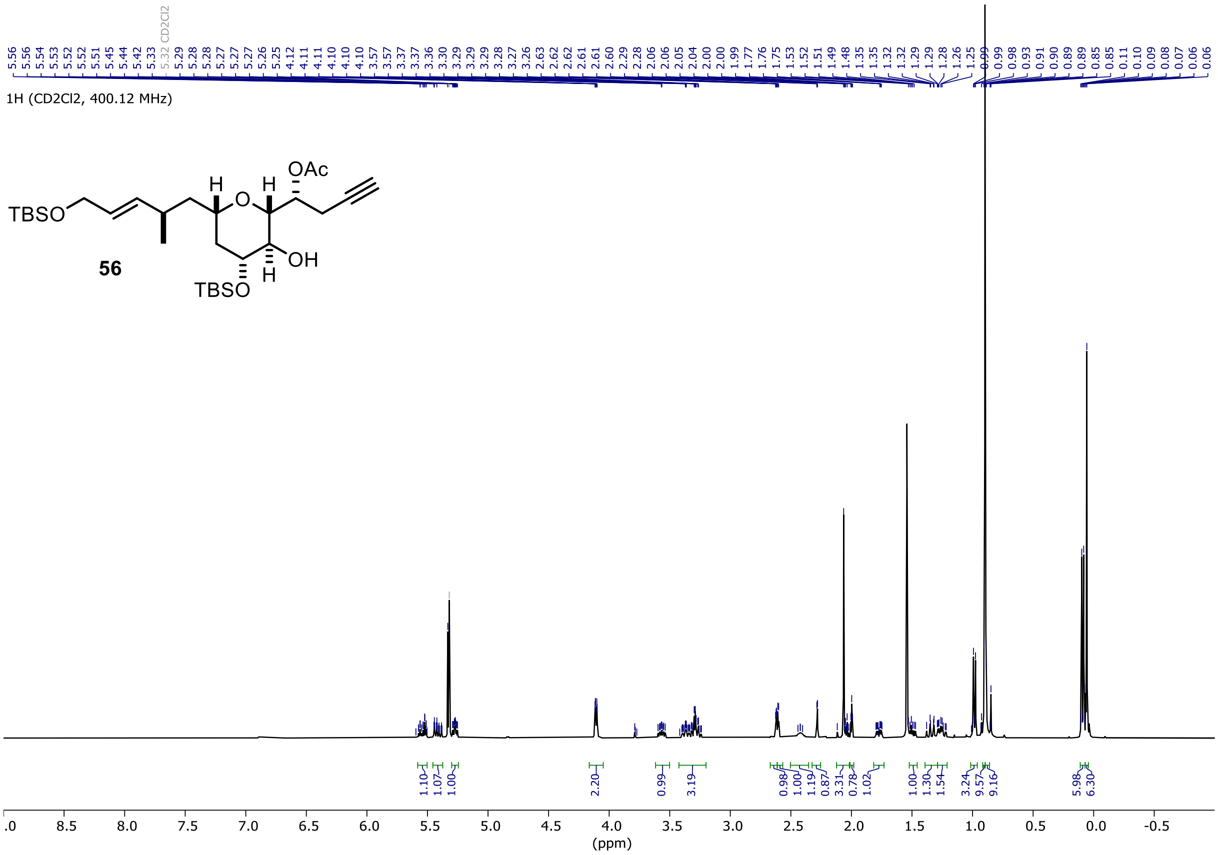
5.56 5.56 5.54 5.53 5.52 5.52 5.51 5.45 5.44 5.42 5.33 5.32 CD2Cl2 5.29 5.28 5.28 5.27 5.27 5.26 5.25 4.12 4.11 4.11 4.10 4.10 3.57 3.57 3.37 3.37 3.36 3.30 3.29 3.29 3.28 3.27 3.26 2.63 2.62 2.62 2.61 2.60 2.29 2.28 2.06 2.06 2.05 2.04 2.00 1.99 1.77 1.76 1.75 1.53 1.52 1.51 1.49 1.48 1.35 1.35 1.32 1.32 1.29 1.29 1.28 1.26 1.25 0.99 0.99 0.98 0.93 0.91 0.90 0.89 0.89 0.85 0.85 0.11 0.10 0.09 0.08 0.07 0.06 0.06
1H (CD2Cl2, 400.12 MHz)
OAc
H
O
H
TBSO
56
OH
TBSO
H
1.10 1.07 1.00 2.20 0.99 3.19 0.98 1.00 1.19 0.87 3.31 0.78 1.02 1.00 1.30 1.54 3.24 9.57 9.16 5.98 6.30
8.5 8.0 7.5 7.0 6.5 6.0 5.5 5.0 4.5 4.0 3.5 3.0 2.5 2.0 1.5 1.0 0.5 0.0 -0.5
(ppm)

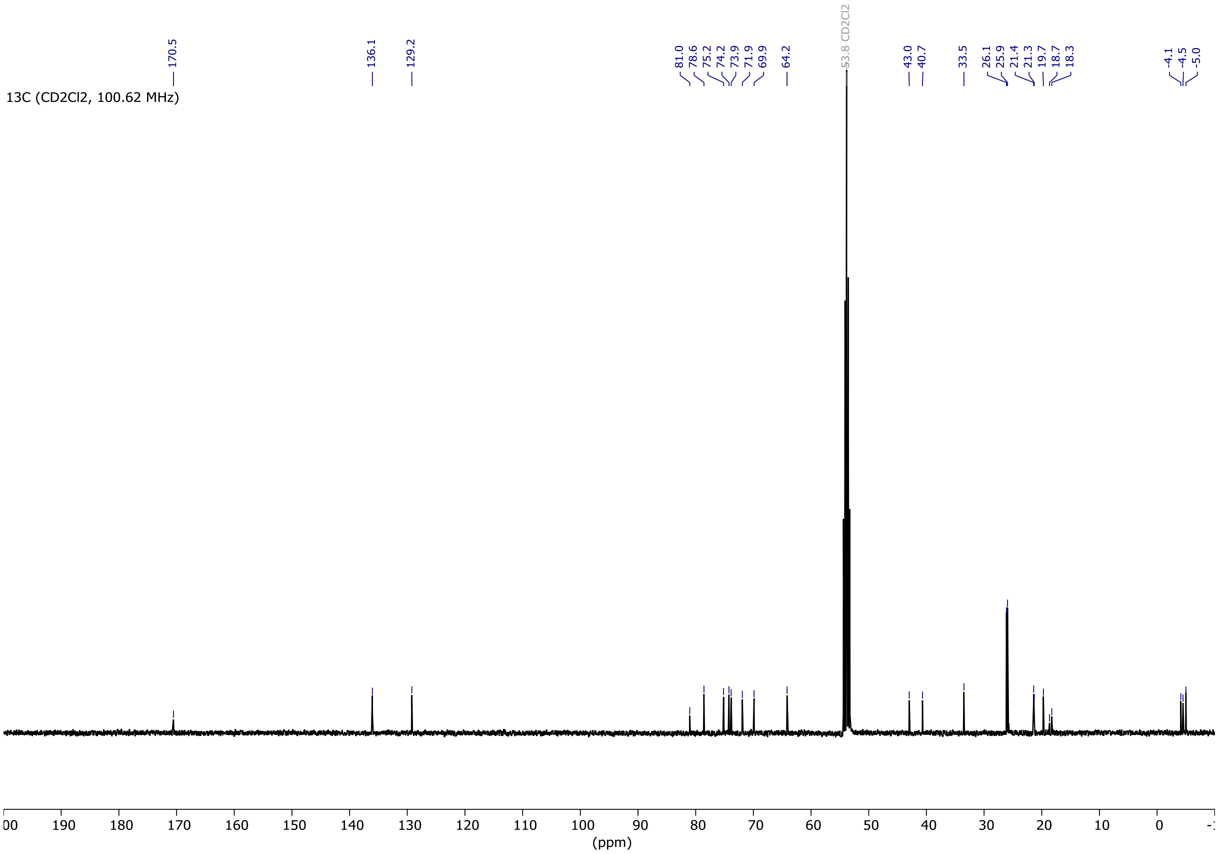
170.5 136.1 129.2 81.0 78.6 75.2 74.2 73.9 71.9 69.9 64.2 53.8 CD2Cl2 43.0 40.7 33.5 26.1 25.9 21.4 21.3 19.7 18.7 18.3 -4.1 -4.5 -5.0
13C (CD2Cl2, 100.62 MHz)
190 180 170 160 150 140 130 120 110 100 90 80 70 60 50 40 30 20 10 0
(ppm)

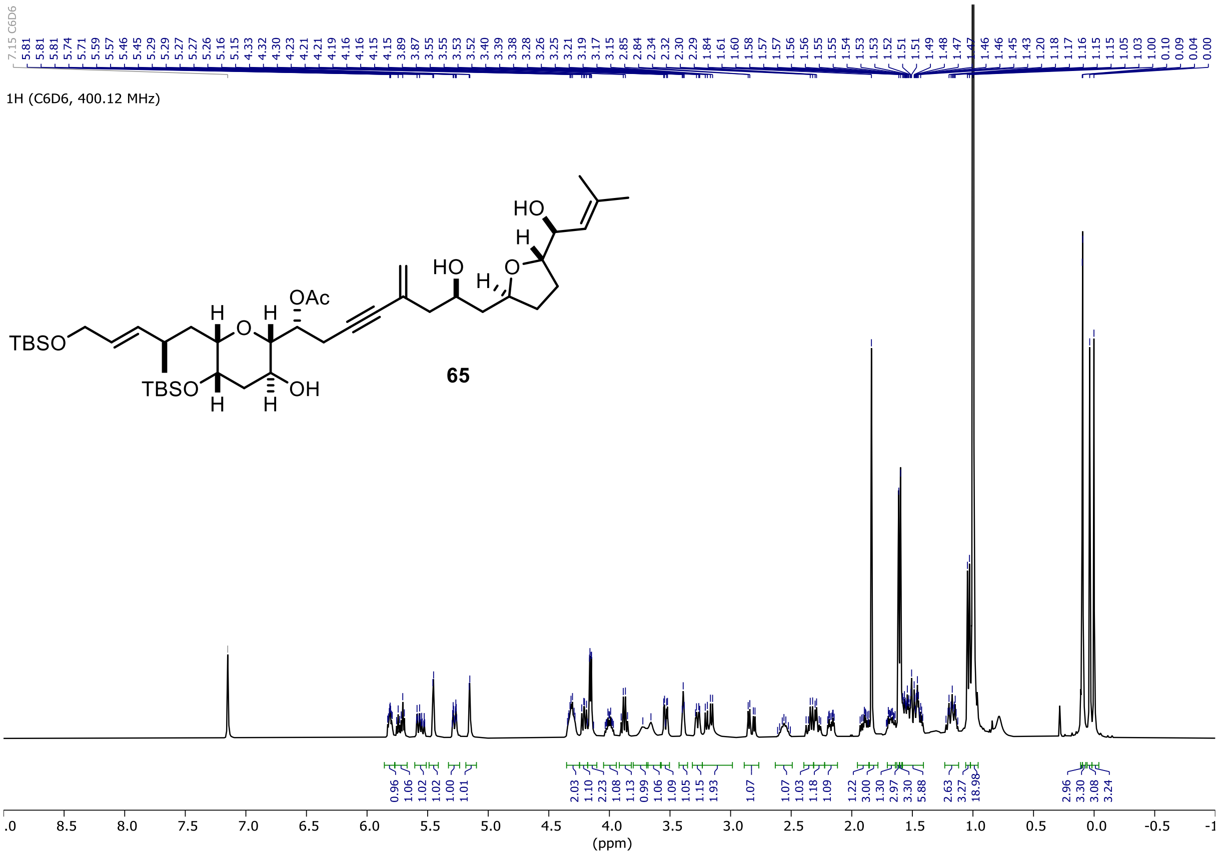
1H (C6D6, 400.12 MHz)
TBSO
TBSO
OAc
HO
HO
OH
65
(ppm)

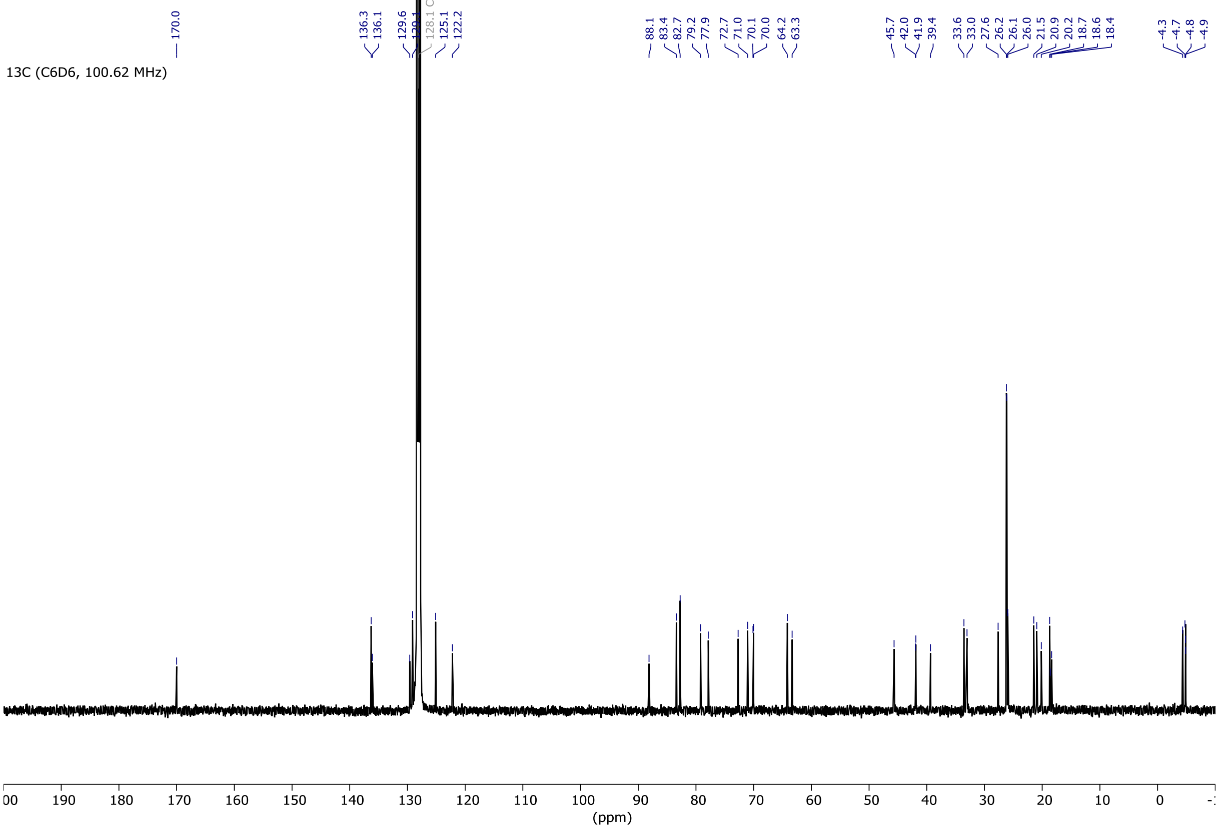
13C (C6D6, 100.62 MHz)
(ppm)

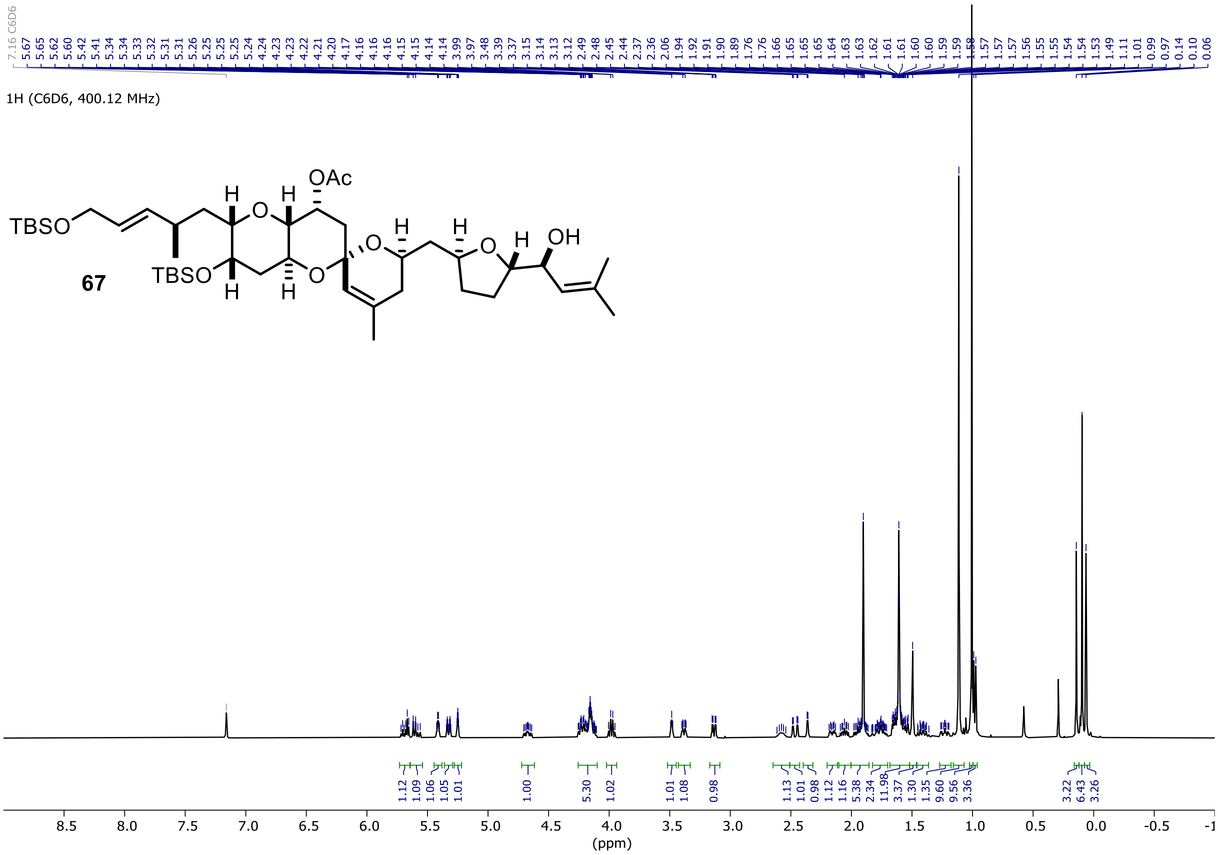
7.16 C6D6
5.67 5.65 5.62 5.60 5.42 5.41 5.34 5.34 5.33 5.32 5.31 5.26 5.25 5.25 5.25 5.24 4.24 4.23 4.22 4.21 4.20 4.17 4.16 4.16 4.15 4.15 4.14 4.14 3.99 3.97 3.48 3.39 3.37 3.15 3.14 3.13 3.12 2.49 2.48 2.45 2.44 2.37 2.36 2.06 1.94 1.92 1.91 1.90 1.89 1.76 1.76 1.66 1.65 1.65 1.65 1.64 1.63 1.63 1.62 1.61 1.61 1.60 1.59 1.59 1.58 1.57 1.57 1.57 1.56 1.55 1.55 1.54 1.54 1.53 1.49 1.11 1.01 0.99 0.97 0.14 0.10 0.06
1H (C6D6, 400.12 MHz)
OAc
TBSO
TBSO
H
O
OH
67
1.12 1.09 1.06 1.05 1.01 1.00 5.30 1.02 1.01 1.08 0.98 1.13 1.01 0.98 1.12 1.16 5.38 2.34 11.98 3.37 1.30 1.35 9.60 9.56 3.36 3.22 6.43 3.26
8.5 8.0 7.5 7.0 6.5 6.0 5.5 5.0 4.5 4.0 3.5 3.0 2.5 2.0 1.5 1.0 0.5 0.0 -0.5
(ppm)

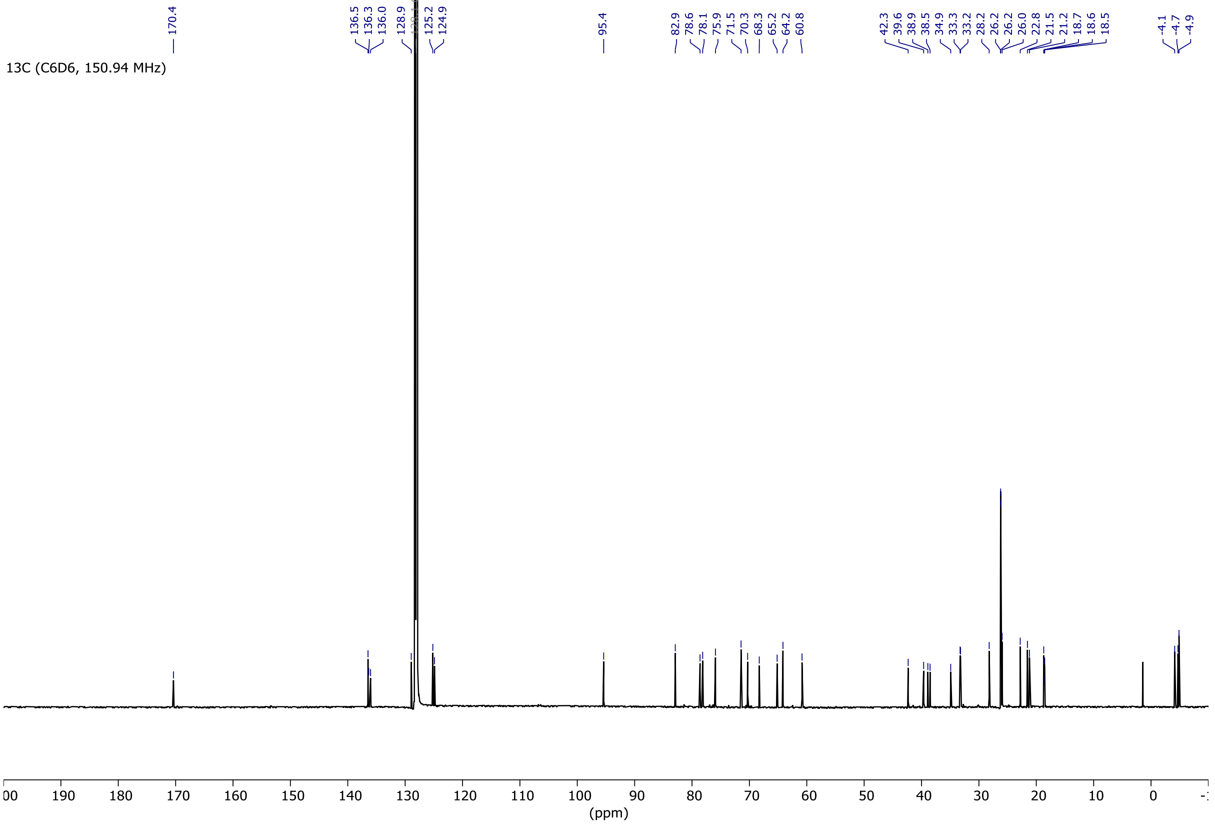
170.4
136.5 136.3 136.0 128.9 125.2 124.9
95.4
82.9 78.6 78.1 75.9 71.5 70.3 68.3 65.2 64.2 60.8
42.3 39.6 38.9 38.5 34.9 33.3 33.2 28.2 26.2 26.2 26.0 22.8 21.5 21.2 18.7 18.6 18.5
-4.1 -4.7 -4.9
13C (C6D6, 150.94 MHz)
190 180 170 160 150 140 130 120 110 100 90 80 70 60 50 40 30 20 10 0
(ppm)

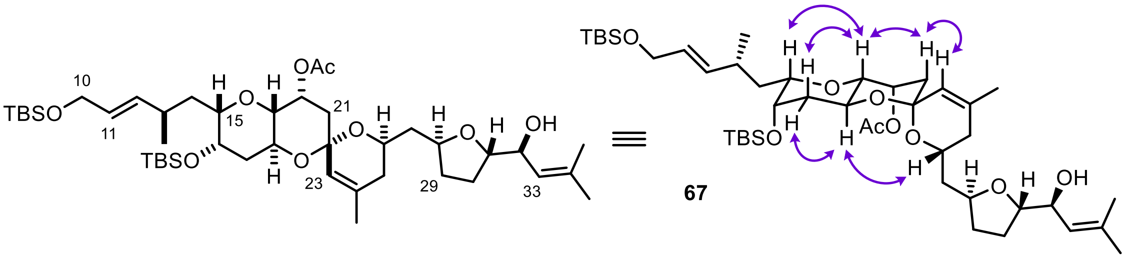

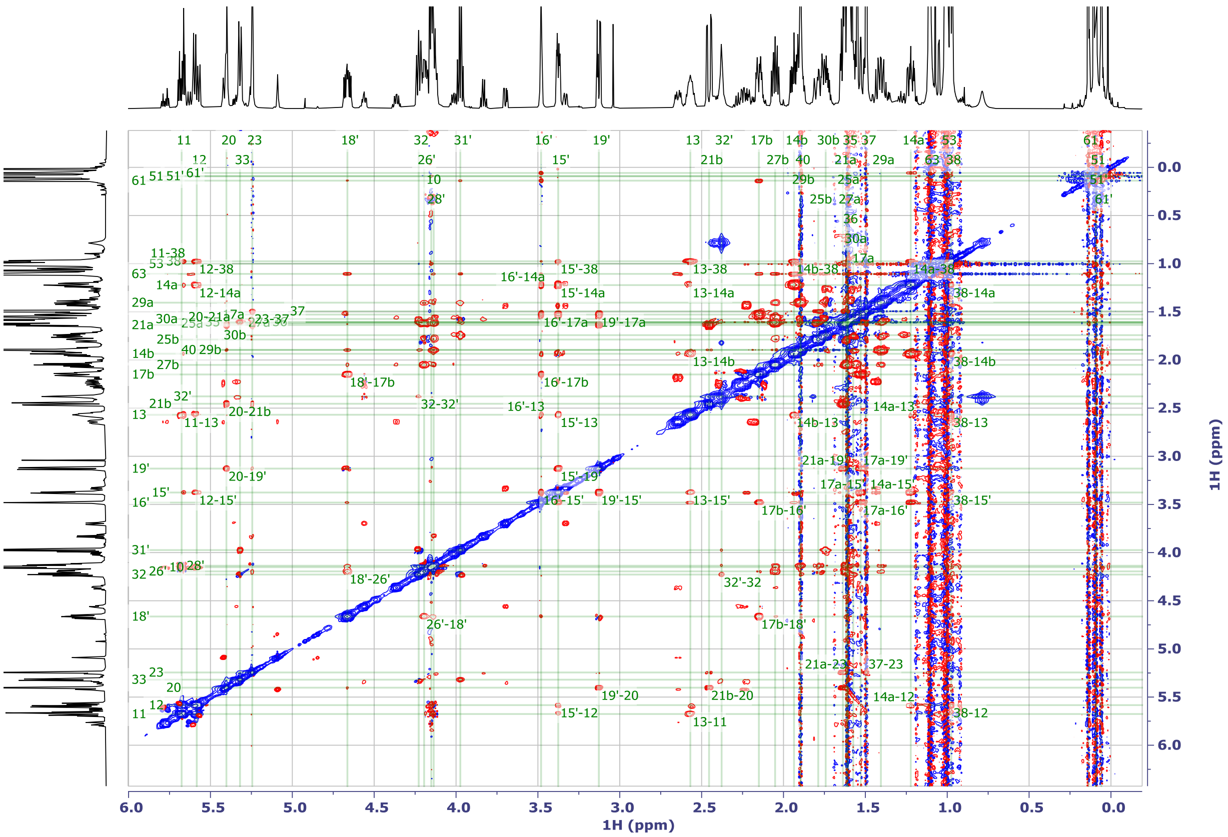

yamyb27501.15.ser — yamyb27501 (9066) — 5 mm, C6D6, 10 mg — 1H (roesyadjsph) @ 298.0 K — AV600neo, cryoBBO — 27.09.22 19:14:16

Figure S10. ROESY-spectrum of spiroketal **67** recorded on a 600 MHz NMR spectrometer. Important NOEs are indicated in the insert by purple double arrows.

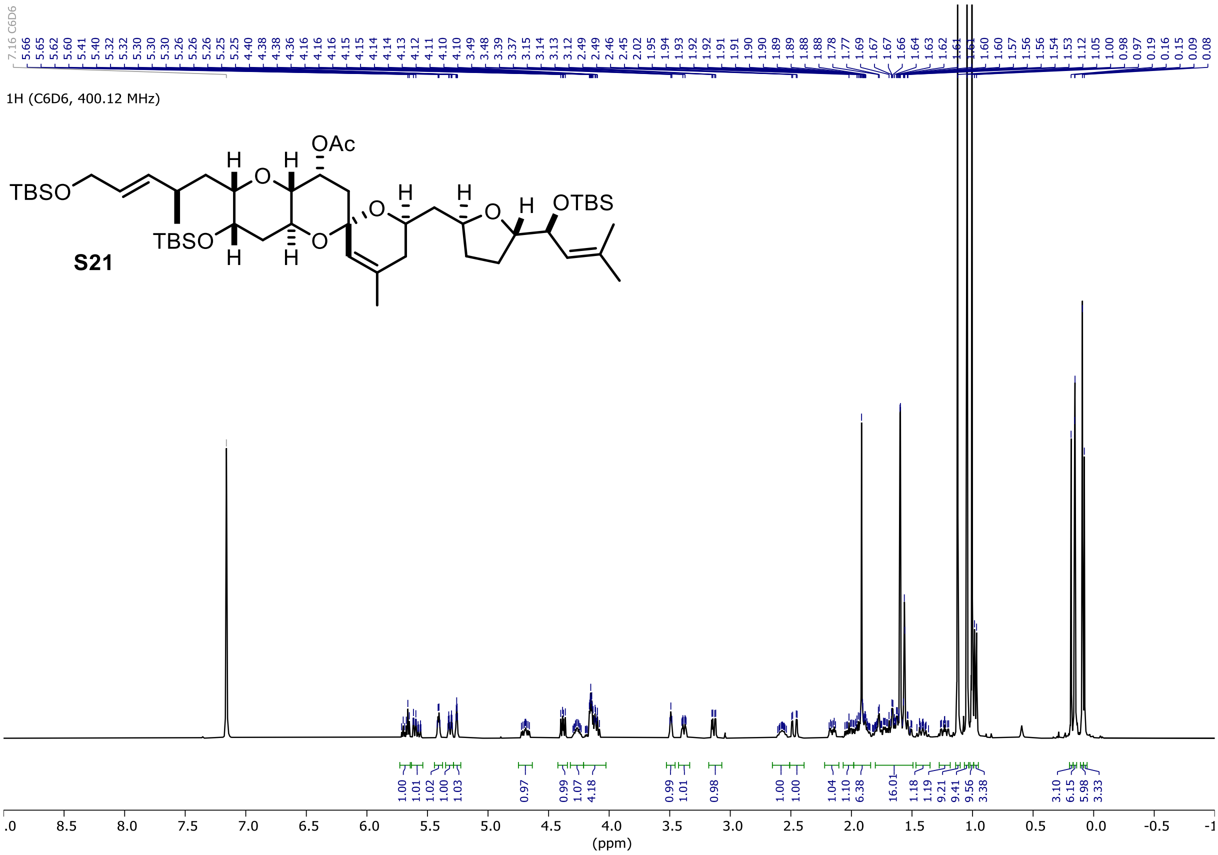
1H (C6D6, 400.12 MHz)
TBSO
TBSO
OAc
OTBS
S21
(ppm)

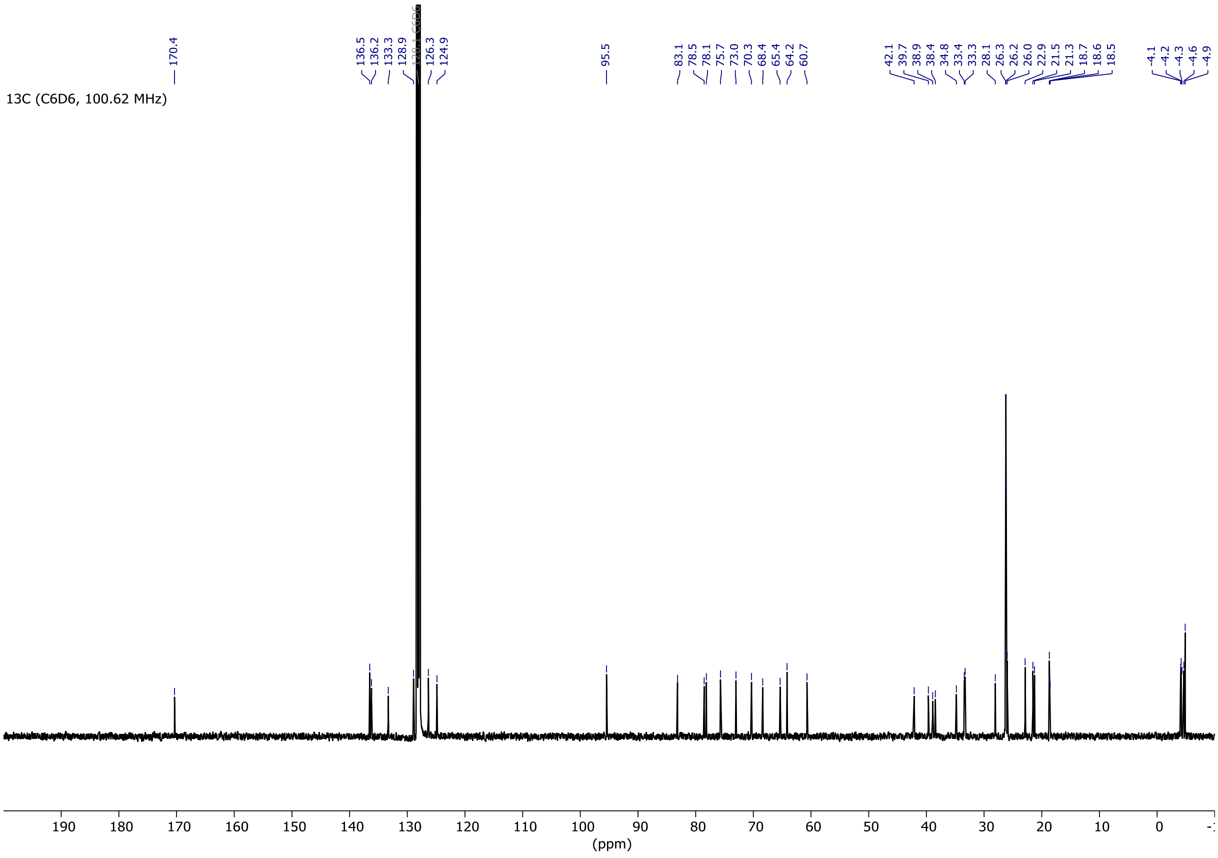
13C (C6D6, 100.62 MHz)
170.4
136.5
136.2
133.3
128.9
126.3
124.9
95.5
83.1
78.5
78.1
75.7
73.0
70.3
68.4
65.4
64.2
60.7
42.1
39.7
38.9
38.4
34.8
33.4
33.3
28.1
26.3
26.2
26.0
22.9
21.5
21.3
18.7
18.6
18.5
-4.1
-4.2
-4.3
-4.6
-4.9
(ppm)

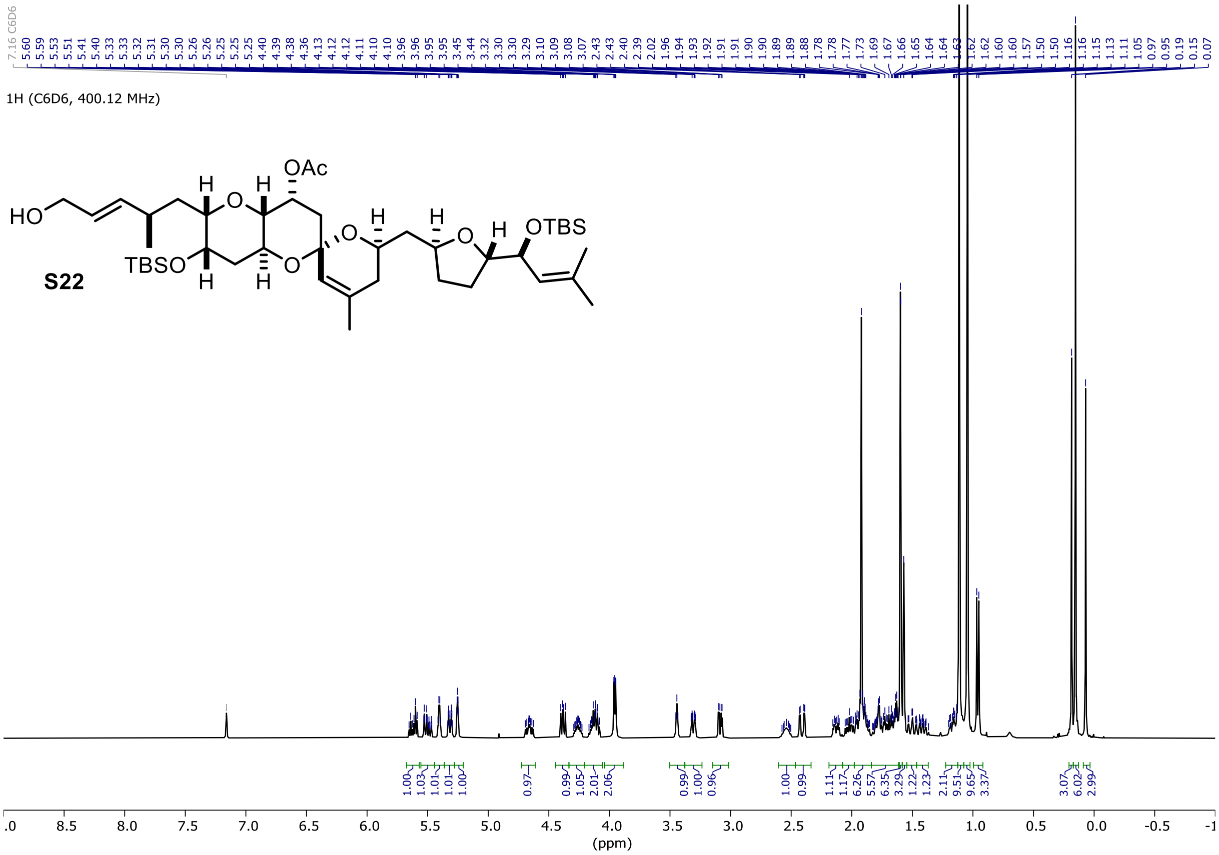
7.16 C6D6
5.60 5.59 5.53 5.51 5.41 5.40 5.33 5.33 5.32 5.31 5.30 5.30 5.26 5.26 5.25 5.25 5.25 4.40 4.39 4.38 4.36 4.13 4.12 4.12 4.11 4.10 4.10 3.96 3.95 3.95 3.45 3.44 3.32 3.30 3.30 3.29 3.10 3.09 3.08 3.07 2.43 2.43 2.40 2.39 2.02 1.96 1.94 1.93 1.92 1.91 1.91 1.90 1.90 1.89 1.89 1.88 1.78 1.78 1.77 1.73 1.69 1.67 1.66 1.65 1.64 1.64 1.63 1.62 1.62 1.60 1.60 1.57 1.50 1.50 1.16 1.16 1.15 1.13 1.11 1.05 0.97 0.95 0.19 0.15 0.07
1H (C6D6, 400.12 MHz)
OAc
HO
H
O
TBSO
S22
OTBS
1.00 1.03 1.01 1.01 1.00 0.97 0.99 1.05 2.01 2.06 0.99 1.00 0.96 1.00 0.99 1.11 1.17 6.26 5.57 6.35 3.29 1.22 1.23 2.11 9.51 9.65 3.37 3.07 6.02 2.99
(ppm)

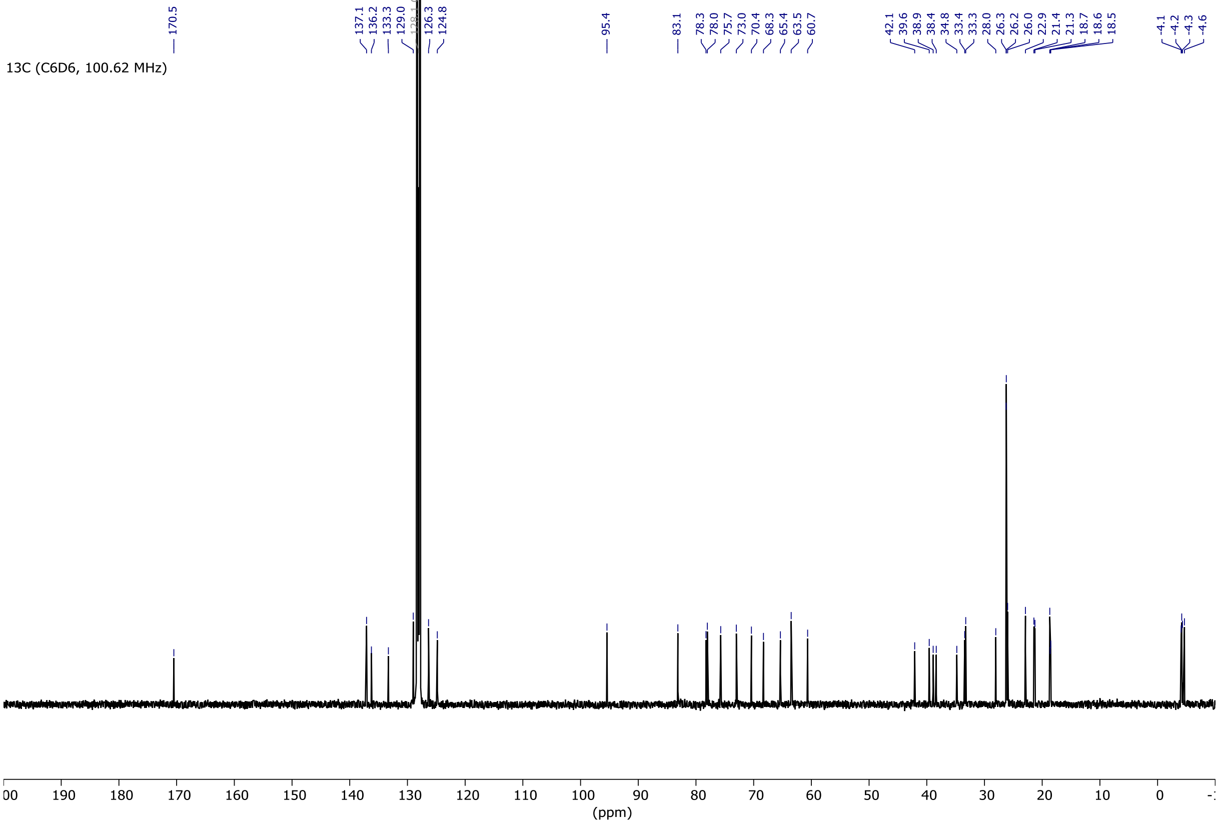
170.5 137.1 136.2 133.3 129.0 128.1 C6D6 126.3 124.8 95.4 83.1 78.3 78.0 75.7 73.0 70.4 68.3 65.4 63.5 60.7 42.1 39.6 38.9 38.4 34.8 33.4 33.3 28.0 26.3 26.2 26.0 22.9 21.4 21.3 18.7 18.6 18.5 -4.1 -4.2 -4.3 -4.6
13C (C6D6, 100.62 MHz)
(ppm)

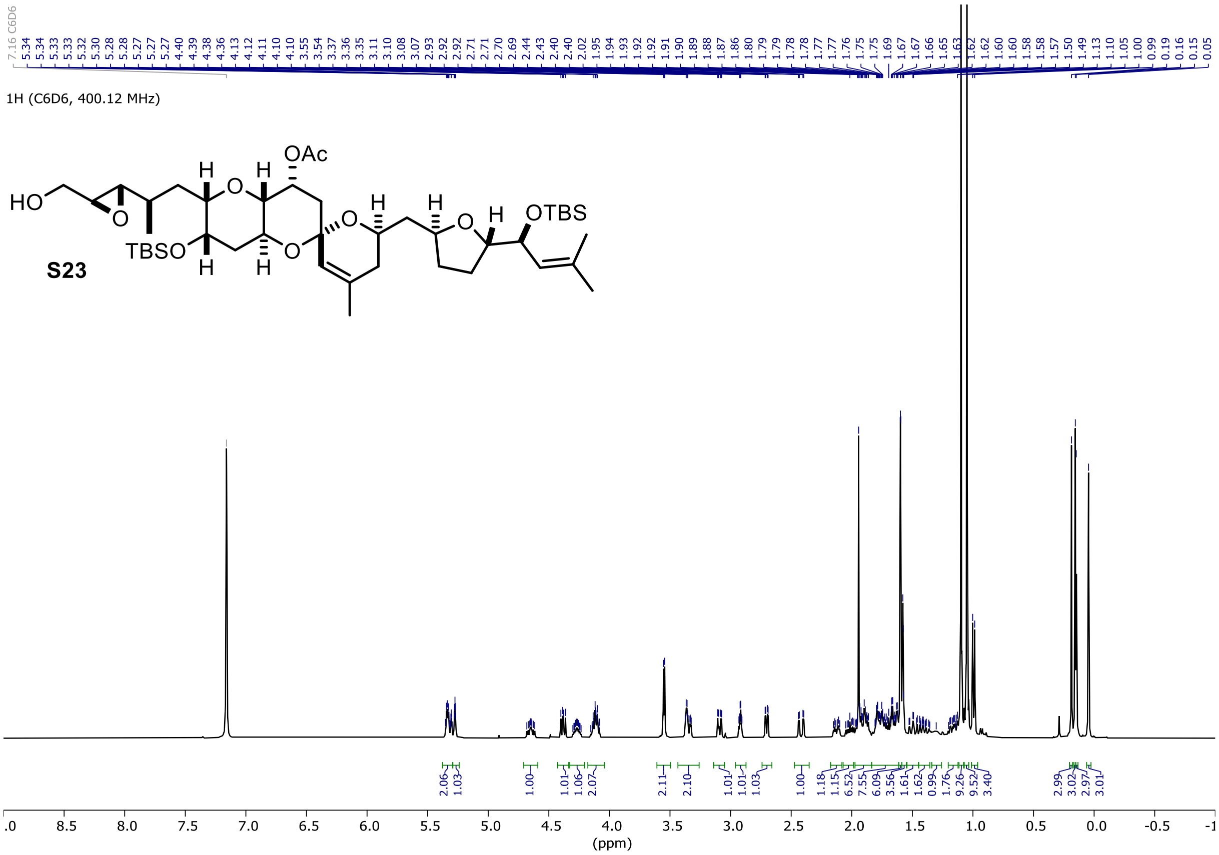
1H (C6D6, 400.12 MHz)
OAc
HO
TBSO
OTBS
S23
(ppm)

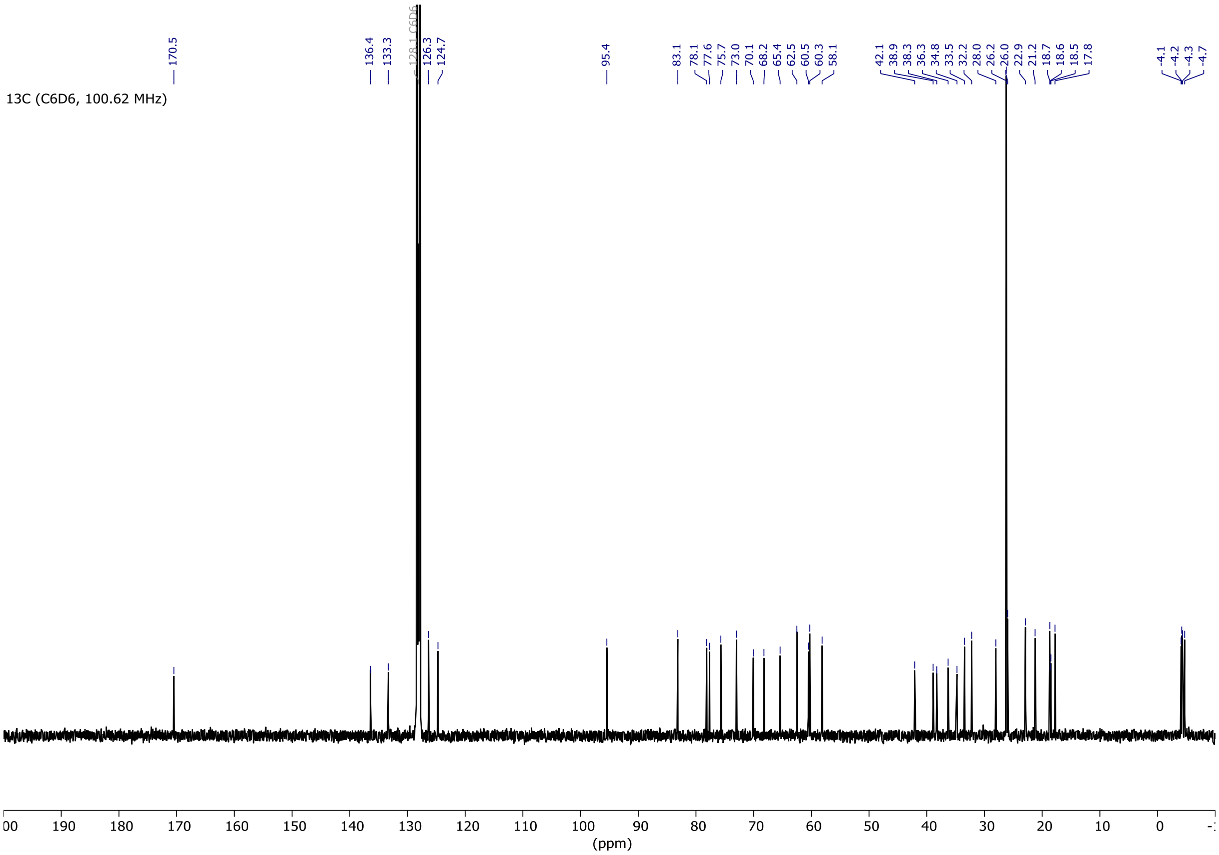
13C (C6D6, 100.62 MHz)
(ppm)

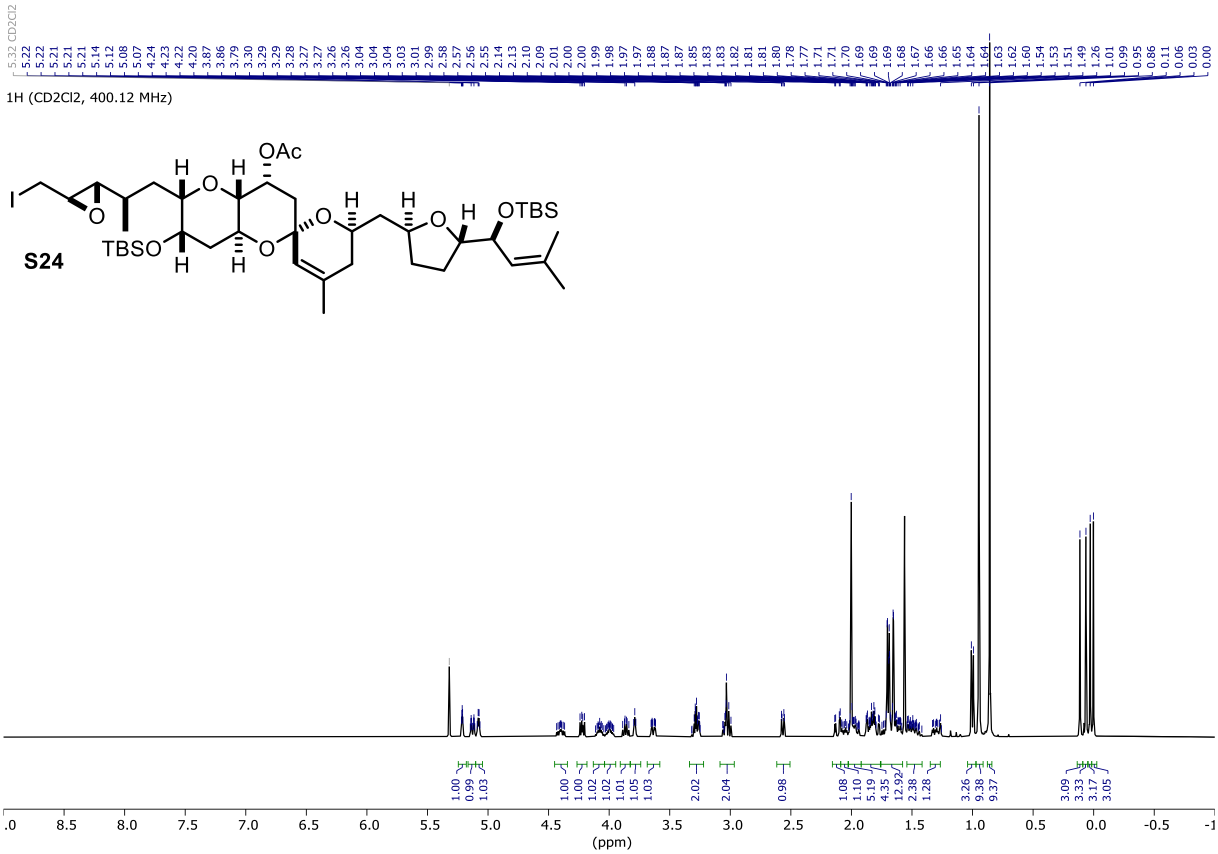
1H (CD2Cl2, 400.12 MHz)
OAc
OTBS
TBSO
S24
(ppm)

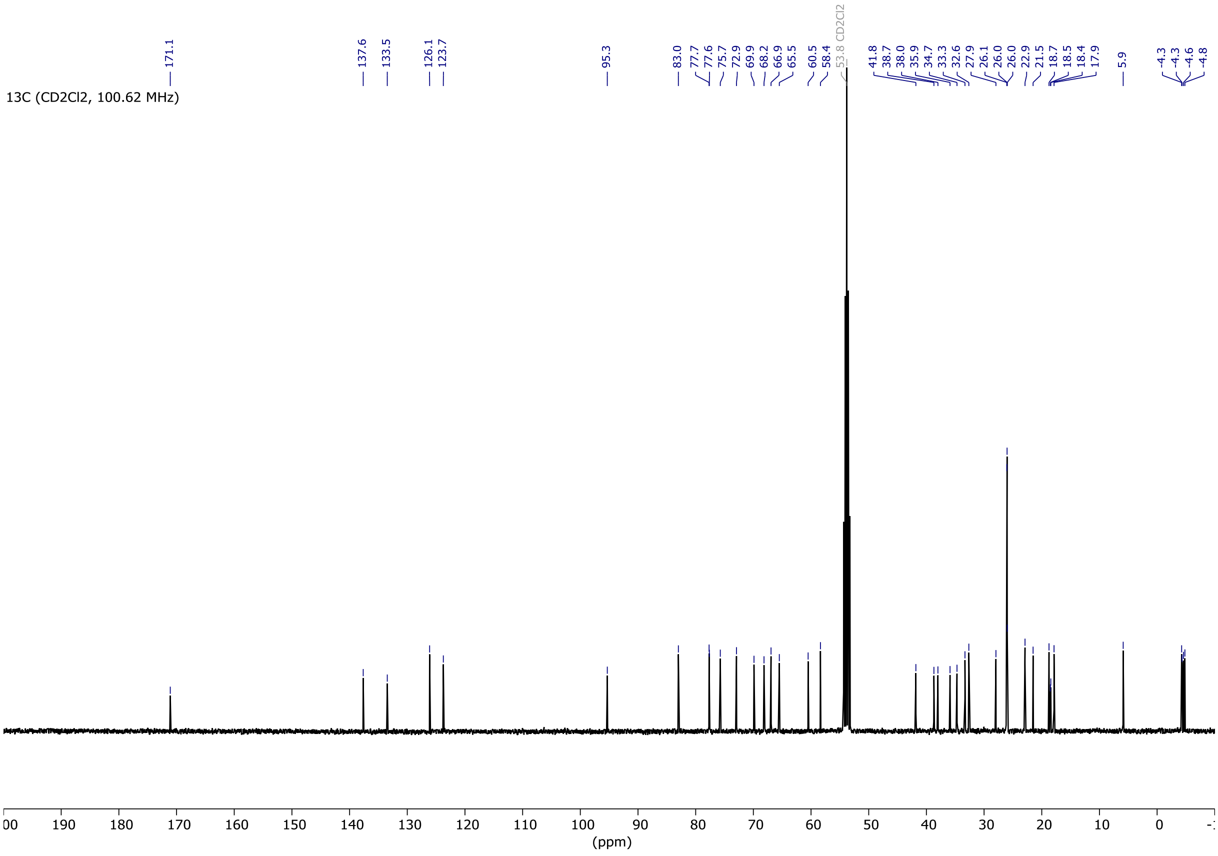
13C (CD2Cl2, 100.62 MHz)
171.1
137.6
133.5
126.1
123.7
95.3
83.0
77.7
77.6
75.7
72.9
69.9
68.2
66.9
65.5
60.5
58.4
53.8 CD2Cl2
41.8
38.7
38.0
35.9
34.7
33.3
32.6
27.9
26.1
26.0
22.9
21.5
18.7
18.5
18.4
17.9
5.9
-4.3
-4.3
-4.6
-4.8
(ppm)

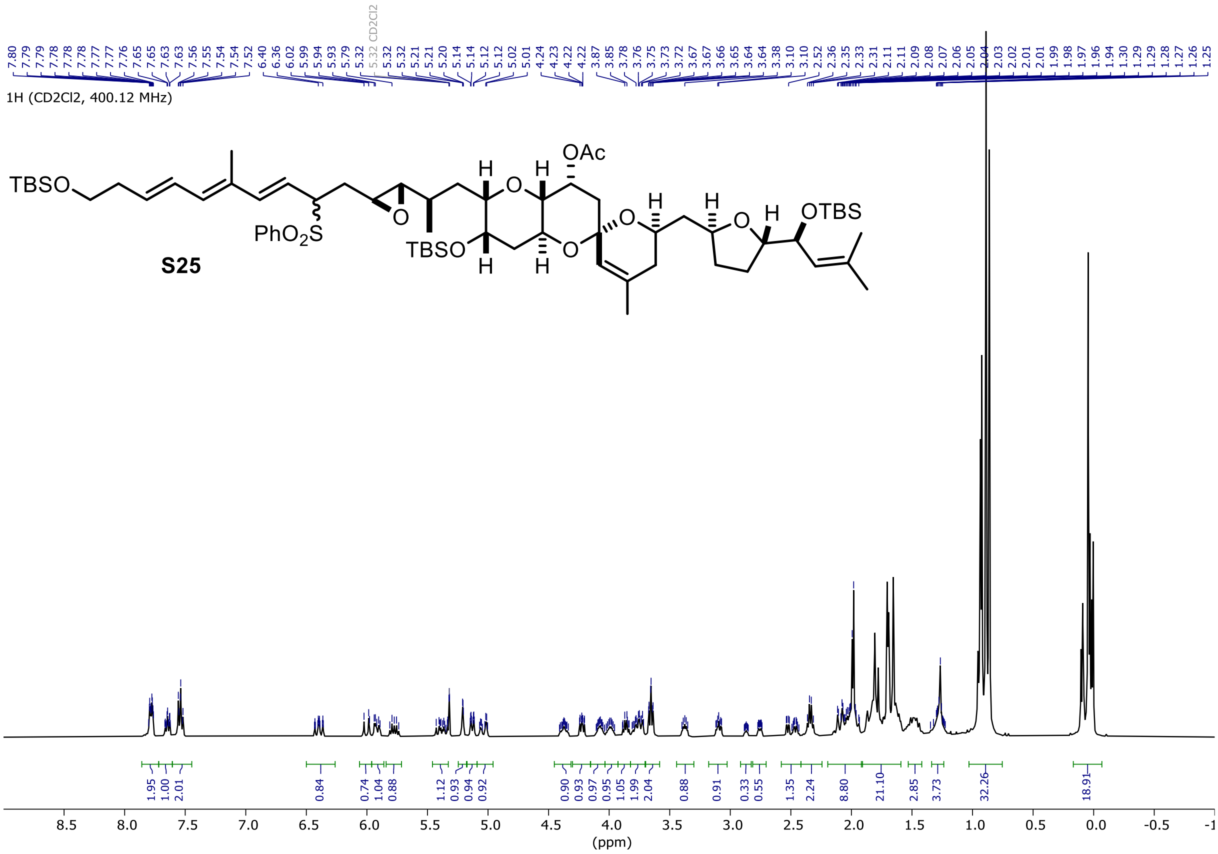
7.80 7.79 7.79 7.78 7.78 7.77 7.77 7.76 7.65 7.63 7.56 7.55 7.54 7.52 6.40 6.36 6.02 5.99 5.94 5.93 5.79 5.32 CD2Cl2 5.32 5.32 5.21 5.20 5.14 5.14 5.12 5.12 5.02 5.01 4.24 4.23 4.22 4.22 3.87 3.85 3.78 3.76 3.75 3.73 3.72 3.67 3.67 3.66 3.65 3.64 3.38 3.10 3.10 2.52 2.36 2.35 2.33 2.31 2.11 2.11 2.09 2.08 2.07 2.06 2.05 2.04 2.03 2.02 2.01 1.99 1.98 1.97 1.96 1.94 1.30 1.29 1.29 1.28 1.27 1.26 1.25
1H (CD2Cl2, 400.12 MHz)
TBSO
OAc
PhO2S
TBSO
OTBS
S25
1.95 1.00 2.01 0.84 0.74 1.04 0.88 1.12 0.93 0.94 0.92 0.90 0.93 0.97 0.95 1.05 1.99 2.04 0.88 0.91 0.33 0.55 1.35 2.24 8.80 21.10 2.85 3.73 32.26 18.91
8.5 8.0 7.5 7.0 6.5 6.0 5.5 5.0 4.5 4.0 3.5 3.0 2.5 2.0 1.5 1.0 0.5 0.0 -0.5
(ppm)

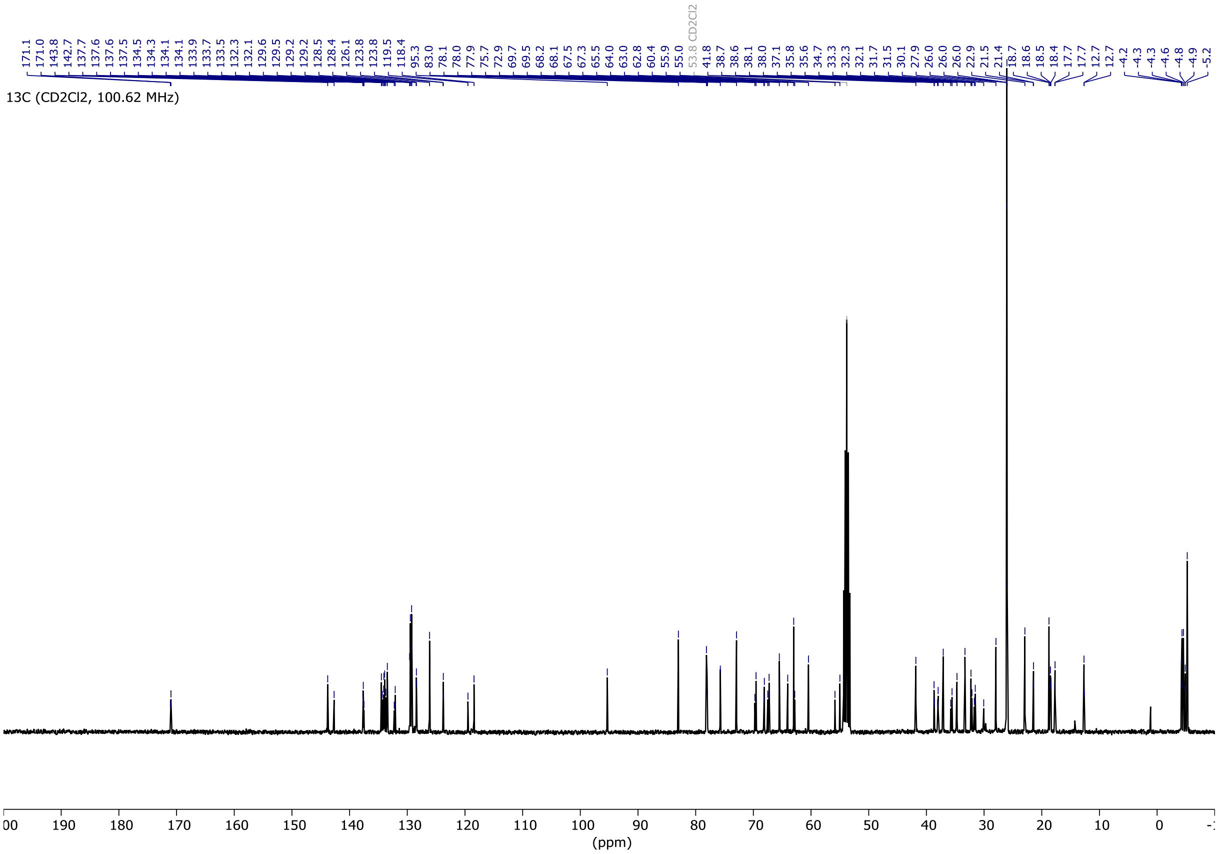
171.1 171.0 143.8 142.7 137.7 137.6 137.5 134.5 134.3 134.1 134.1 133.9 133.7 133.5 132.3 132.1 129.6 129.5 129.2 128.5 128.4 126.1 123.8 123.8 119.5 118.4 95.3 83.0 78.1 78.0 77.9 75.7 72.9 69.7 69.5 68.2 68.1 67.5 67.3 65.5 64.0 63.0 62.8 60.4 55.9 55.0 53.8 CD2Cl2 41.8 38.7 38.6 38.1 38.0 37.1 35.8 35.6 34.7 33.3 32.3 32.1 31.7 31.5 30.1 27.9 26.0 26.0 22.9 21.5 21.4 18.7 18.6 18.5 18.4 17.7 17.7 12.7 12.7 -4.2 -4.3 -4.6 -4.8 -4.9 -5.2
13C (CD2Cl2, 100.62 MHz)
190 180 170 160 150 140 130 120 110 100 90 80 70 60 50 40 30 20 10 0
(ppm)

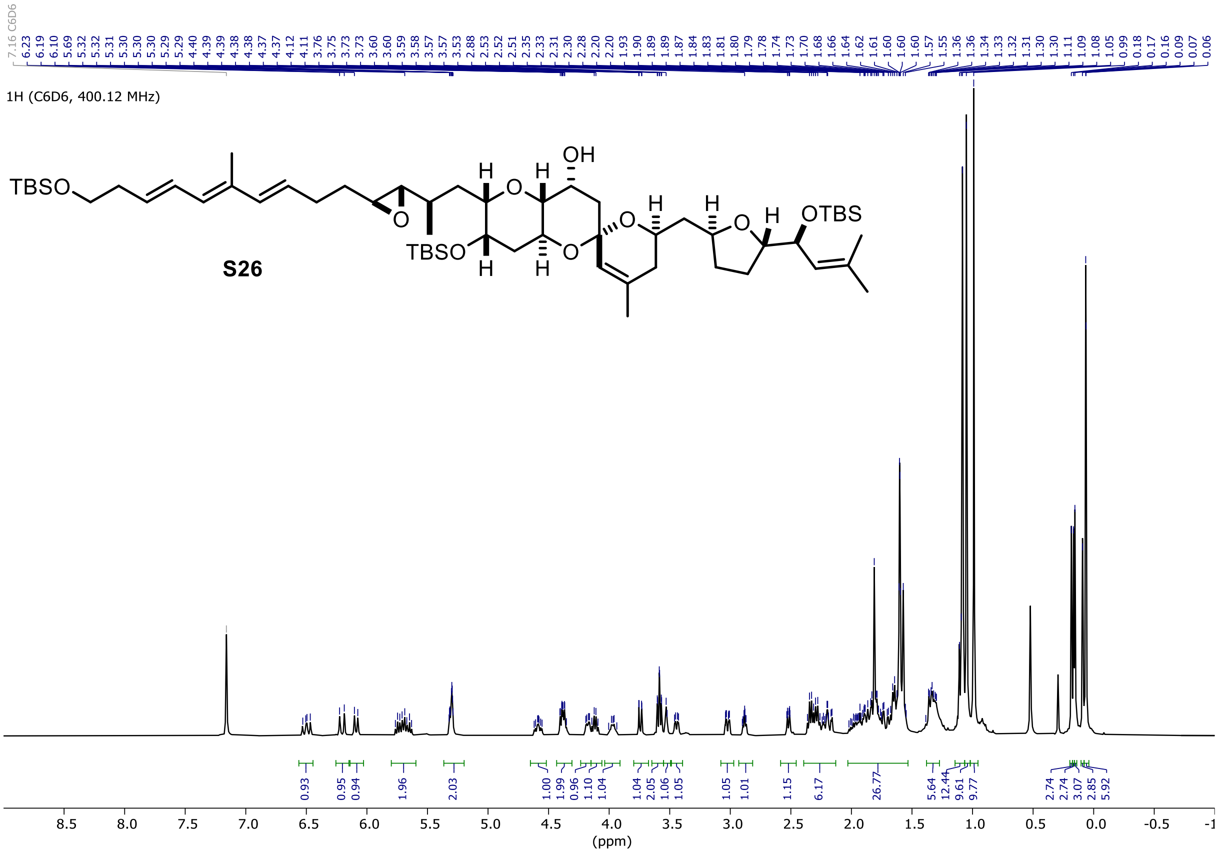
7.16 C6D6
6.23
6.19
6.10
5.69
5.32
5.32
5.31
5.30
5.30
5.30
5.29
4.40
4.39
4.39
4.38
4.38
4.37
4.37
4.12
4.11
3.76
3.75
3.73
3.73
3.60
3.59
3.58
3.57
3.57
3.53
2.88
2.53
2.52
2.51
2.35
2.33
2.31
2.30
2.28
2.20
2.20
1.93
1.90
1.89
1.89
1.87
1.84
1.83
1.81
1.80
1.79
1.78
1.74
1.73
1.70
1.68
1.66
1.64
1.62
1.61
1.60
1.60
1.60
1.60
1.57
1.55
1.36
1.36
1.34
1.33
1.32
1.31
1.30
1.30
1.11
1.09
1.08
1.05
0.99
0.18
0.17
0.16
0.09
0.07
0.06
1H (C6D6, 400.12 MHz)
TBSO
OH
H
O
H
O
H
H
OTBS
TBSO
H
H
S26
0.93
0.95
0.94
1.96
2.03
1.00
1.99
0.96
1.10
1.04
1.04
2.05
1.06
1.05
1.05
1.01
1.15
6.17
26.77
5.64
12.44
9.61
9.77
2.74
2.74
3.07
2.85
5.92
8.5
8.0
7.5
7.0
6.5
6.0
5.5
5.0
4.5
4.0
3.5
3.0
2.5
2.0
1.5
1.0
0.5
0.0
-0.5
(ppm)

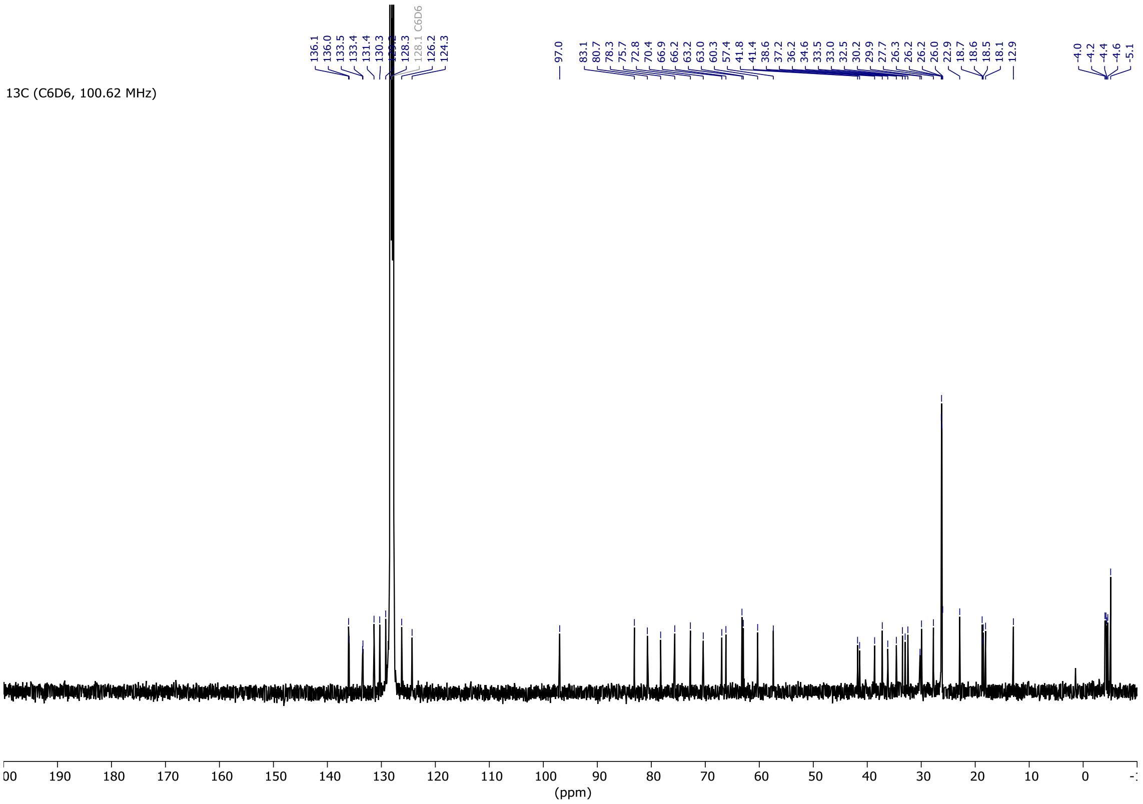
136.1
136.0
133.5
133.4
131.4
130.3
128.3
128.1 C6D6
126.2
124.3
97.0
83.1
80.7
78.3
75.7
72.8
70.4
66.9
66.2
63.2
63.0
60.3
57.4
41.8
41.4
38.6
37.2
36.2
34.6
33.5
33.0
32.5
30.2
29.9
27.7
26.3
26.2
26.2
26.0
22.9
18.7
18.6
18.5
18.1
12.9
-4.0
-4.2
-4.4
-4.6
-5.1
13C (C6D6, 100.62 MHz)
190
180
170
160
150
140
130
120
110
100
90
80
70
60
50
40
30
20
10
0
(ppm)

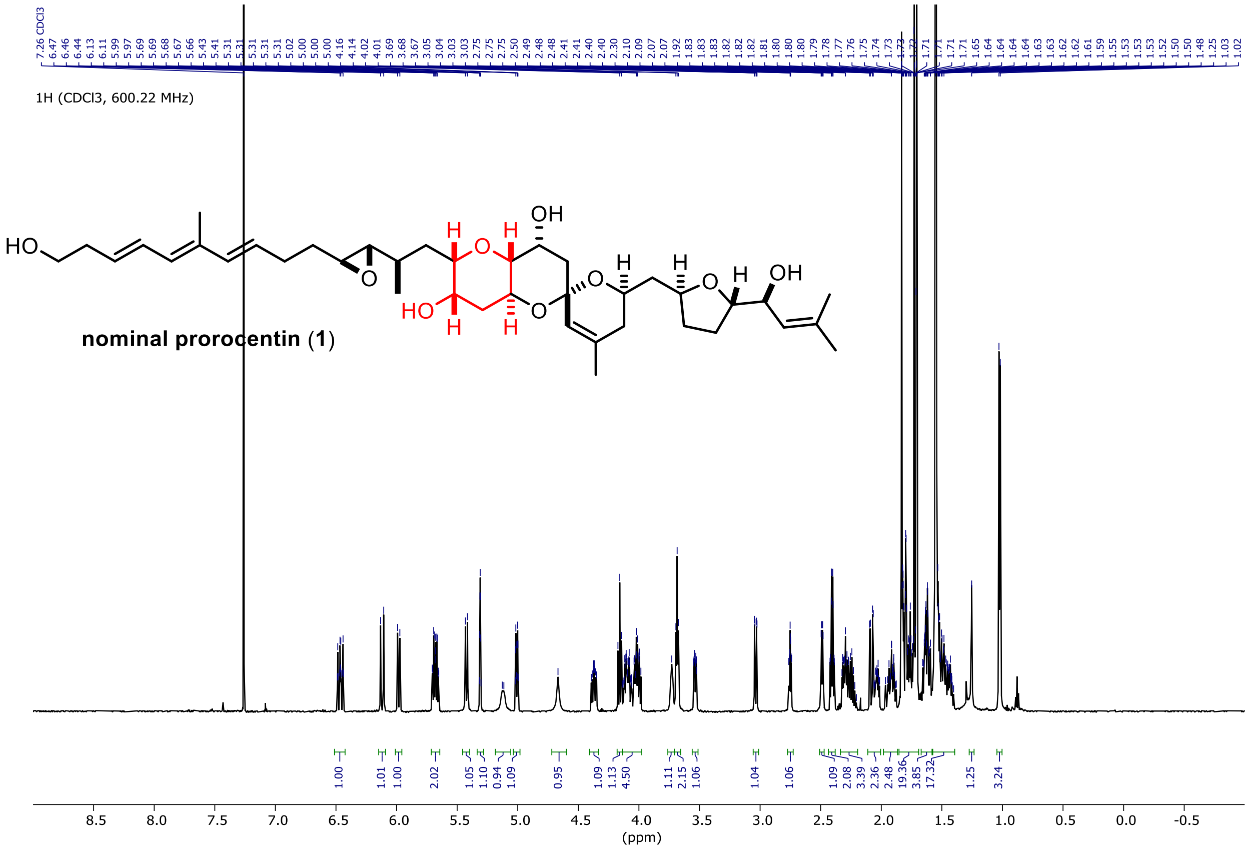
1H (CDCl3, 600.22 MHz)
HO
OH
O
HO
H
OH
nominal prorocentin (1)
(ppm)

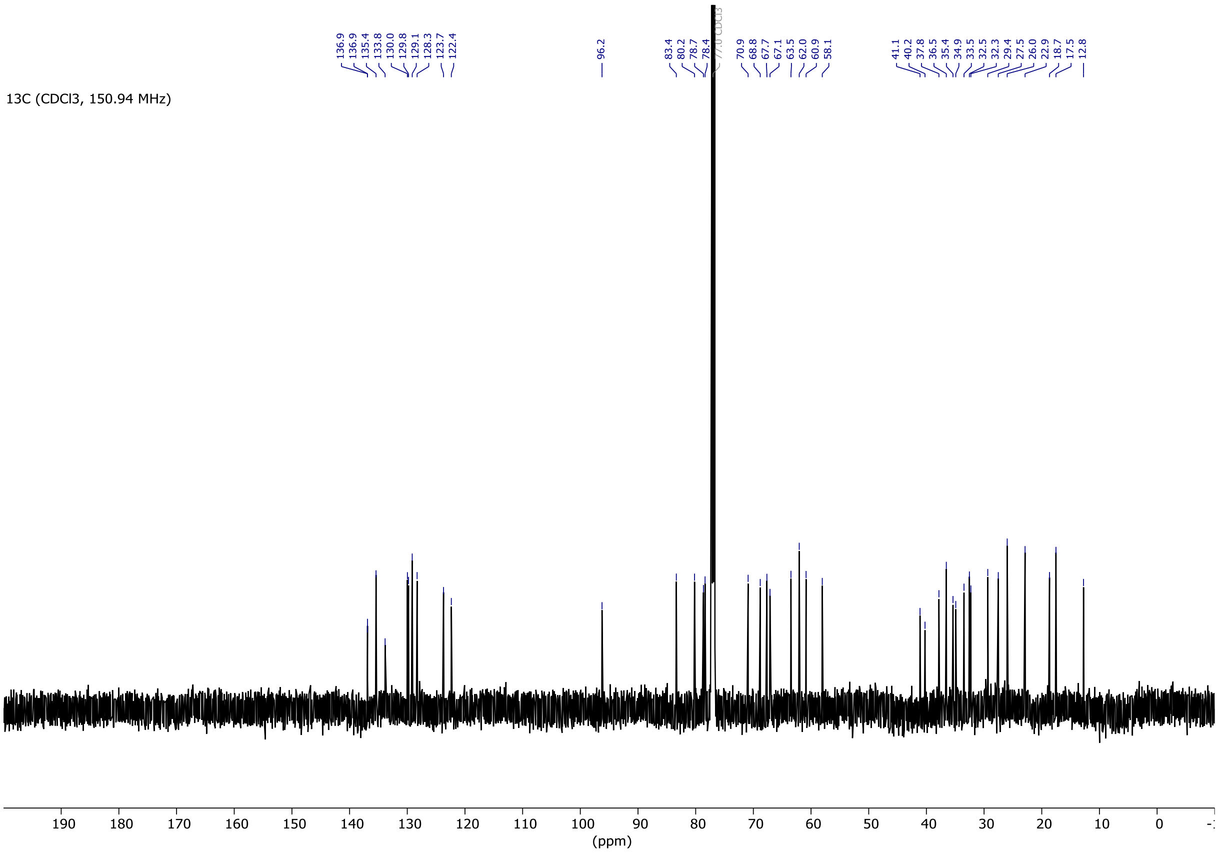
13C (CDCl3, 150.94 MHz)
136.9
136.9
135.4
133.8
130.0
129.8
129.1
128.3
123.7
122.4
96.2
83.4
80.2
78.7
78.4
77.0 CDCl3
70.9
68.8
67.7
67.1
63.5
62.0
60.9
58.1
41.1
40.2
37.8
36.5
35.4
34.9
33.5
32.5
32.3
29.4
27.5
26.0
22.9
18.7
17.5
12.8
(ppm)

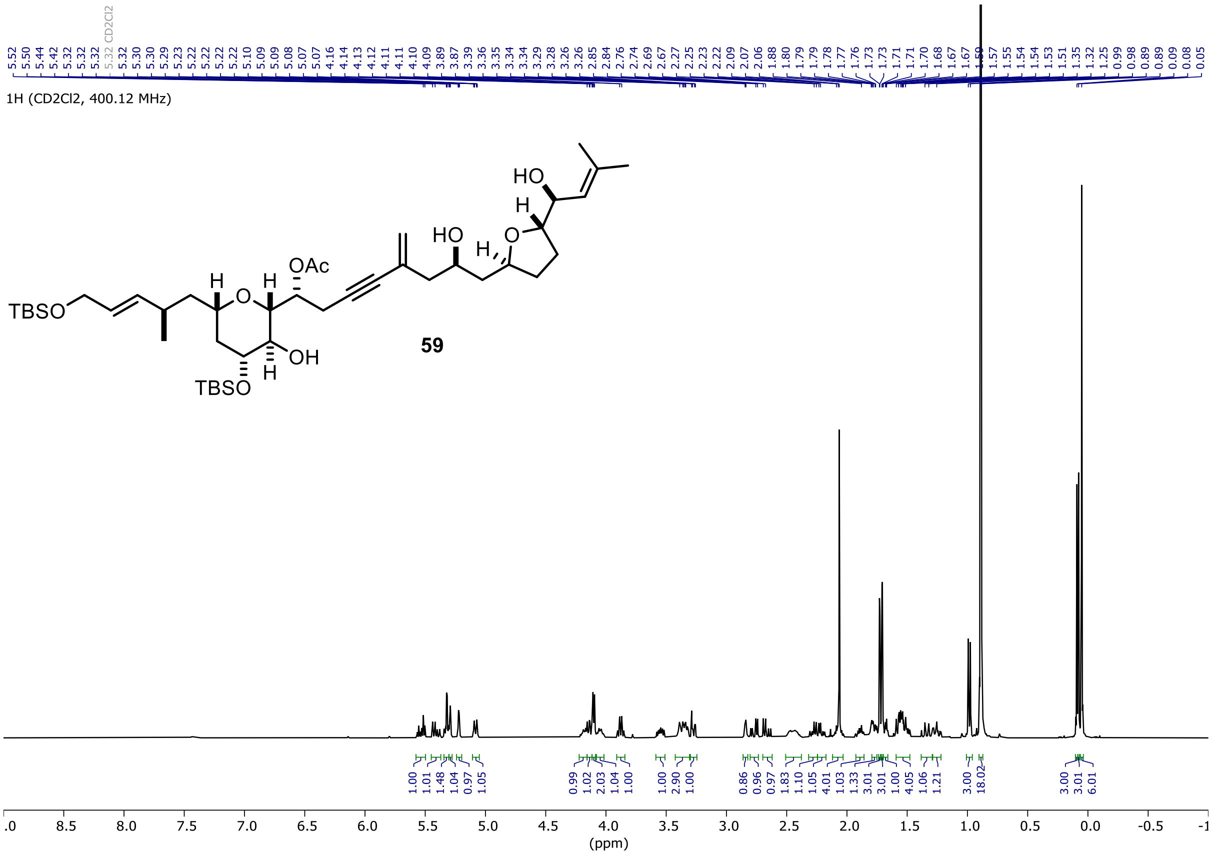
5.52 5.50 5.44 5.42 5.32 5.32 5.32 CD2Cl2 5.32 5.30 5.30 5.29 5.23 5.22 5.22 5.22 5.22 5.10 5.09 5.08 5.07 5.07 4.16 4.14 4.13 4.12 4.11 4.10 4.09 3.89 3.87 3.39 3.36 3.35 3.35 3.34 3.29 3.28 3.26 3.26 2.85 2.84 2.76 2.74 2.69 2.67 2.27 2.25 2.23 2.22 2.09 2.07 2.06 1.88 1.80 1.79 1.79 1.78 1.77 1.76 1.73 1.73 1.71 1.71 1.70 1.68 1.67 1.67 1.59 1.57 1.55 1.54 1.53 1.51 1.35 1.32 1.25 0.99 0.98 0.89 0.89 0.09 0.08 0.05
1H (CD2Cl2, 400.12 MHz)
HO
H
O
HO
H
OAc
H
O
H
TBSO
OH
H
TBSO
59
1.00 1.01 1.48 1.04 0.97 1.05 0.99 1.02 2.03 1.04 1.00 1.00 2.90 1.00 0.86 0.96 0.97 1.83 1.10 1.05 4.01 1.03 1.33 3.01 3.01 1.00 4.05 1.06 1.21 3.00 18.02 3.00 3.01 6.01
(ppm)

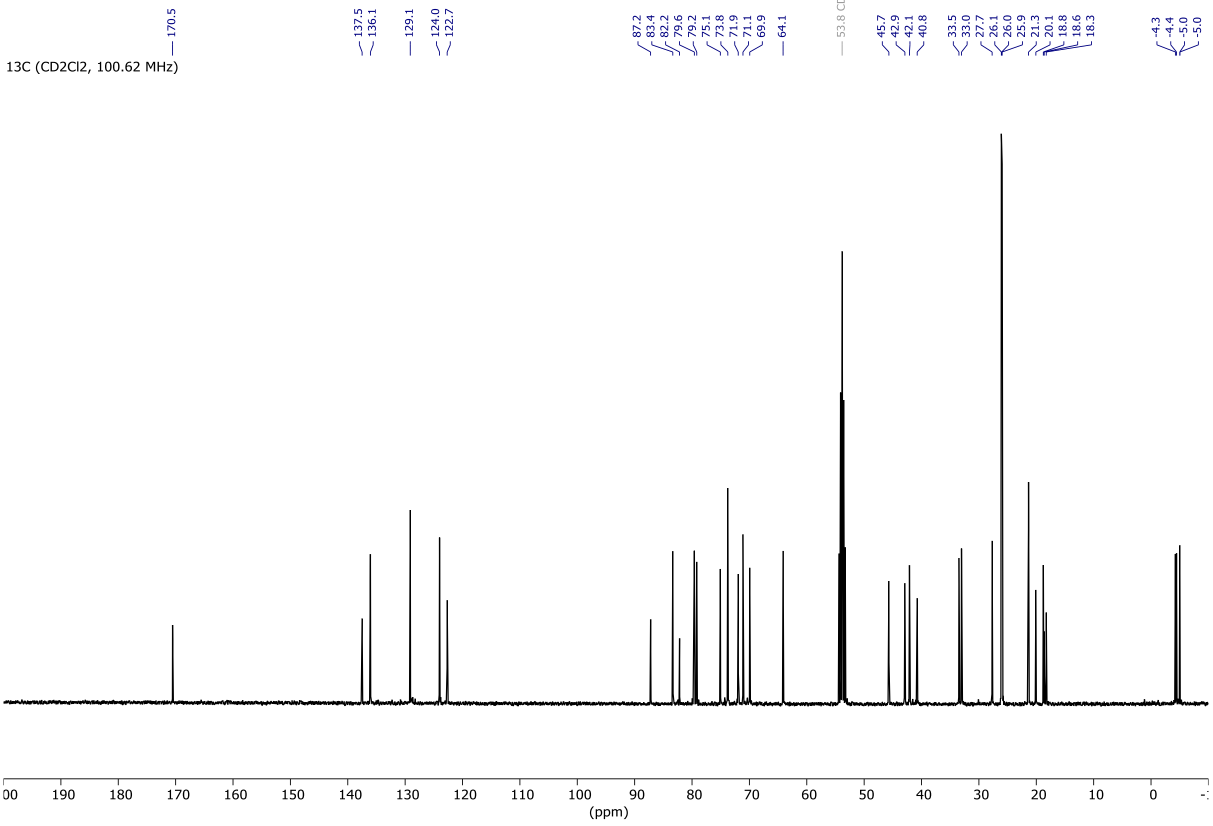
170.5 137.5 136.1 129.1 124.0 122.7 87.2 83.4 82.2 79.6 79.2 75.1 73.8 71.9 71.1 69.9 64.1 53.8 CD2Cl2 45.7 42.9 42.1 40.8 33.5 33.0 27.7 26.1 26.0 25.9 21.3 20.1 18.8 18.6 18.3 -4.3 -4.4 -5.0 -5.0
13C (CD2Cl2, 100.62 MHz)
(ppm)

1H (CD2Cl2, 600.20 MHz)

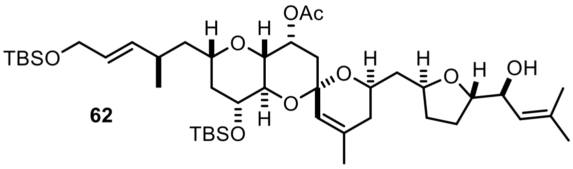

62

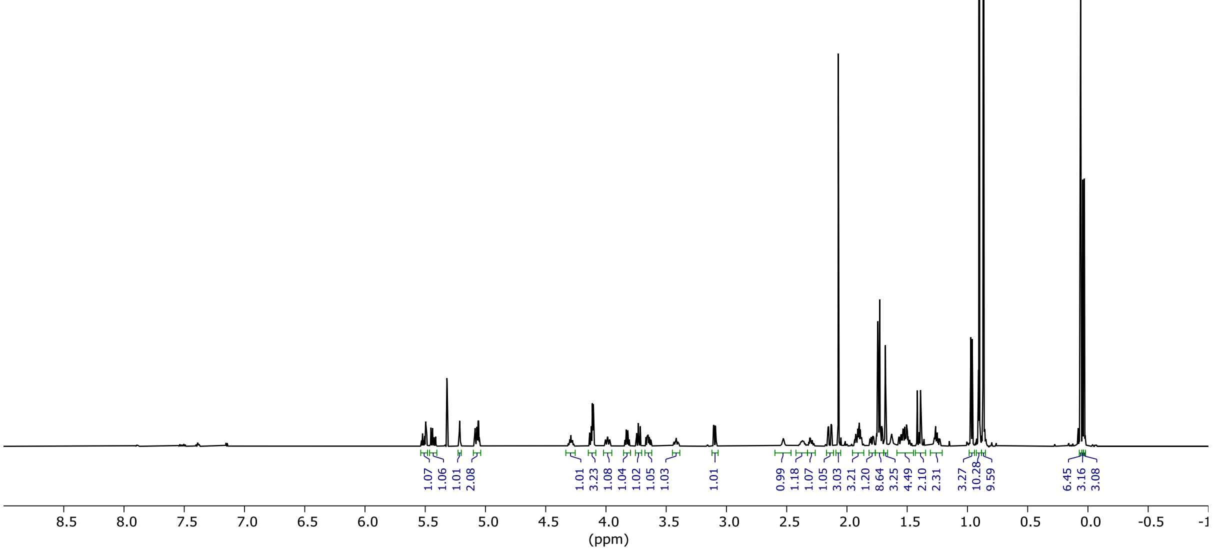

13C (CD2Cl2, 150.94 MHz)

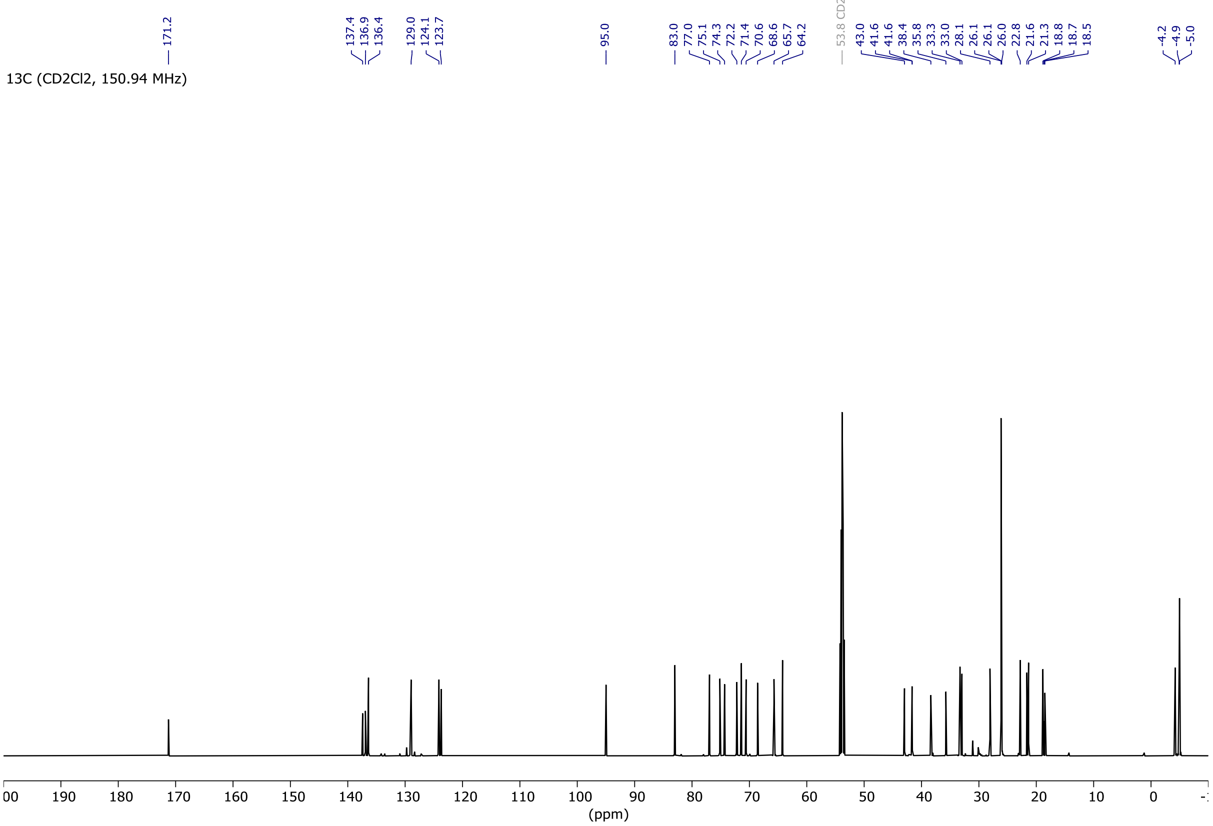

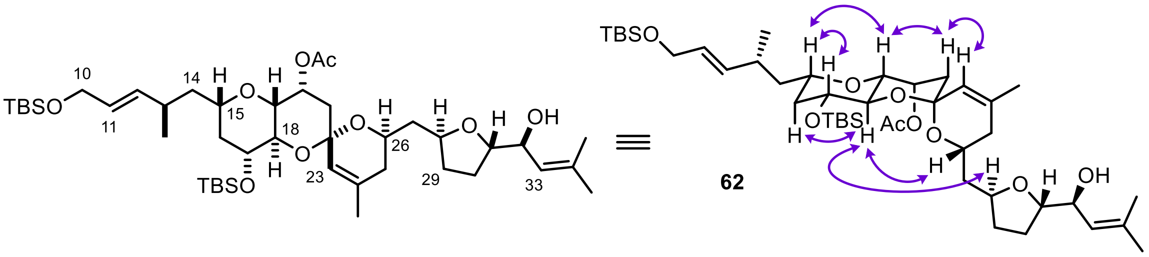

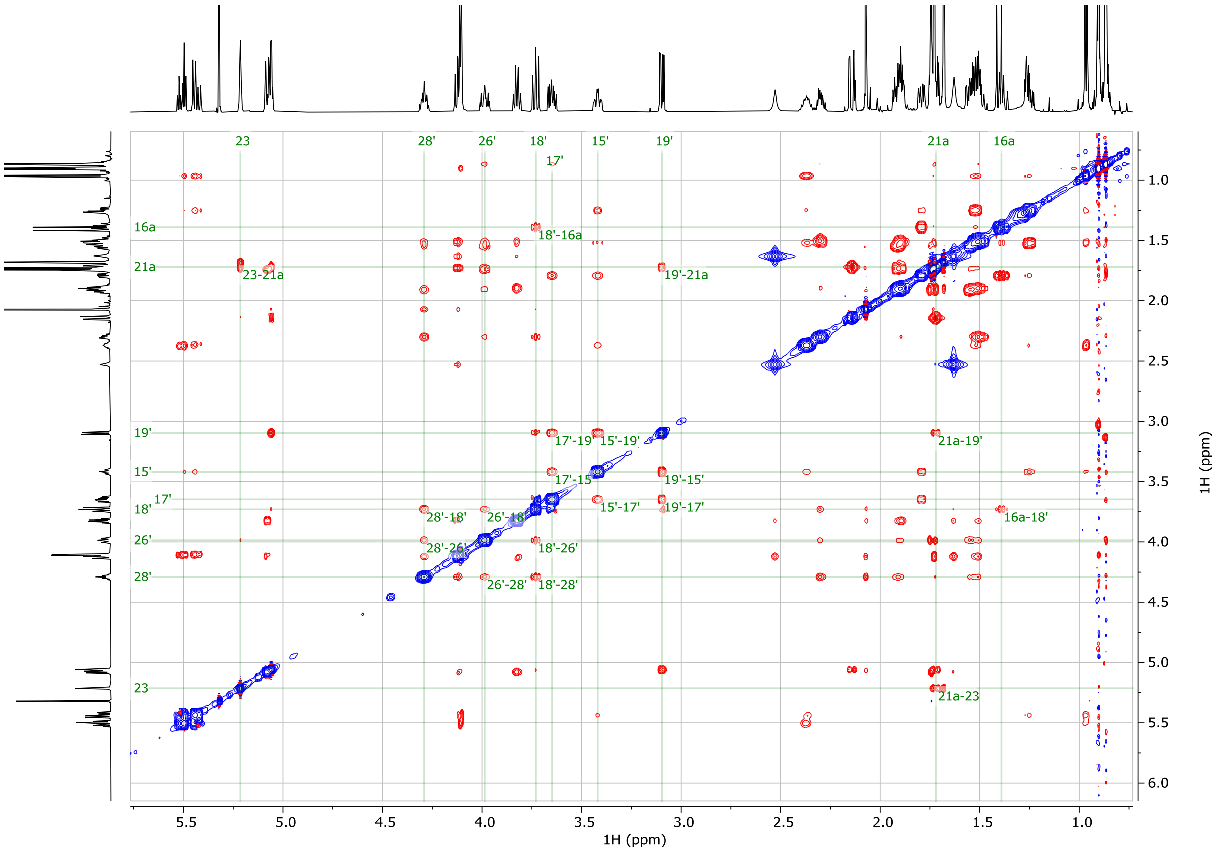

Figure S11. ROESY-spectrum of spiroketal **62** recorded on a 600 MHz NMR spectrometer. Important NOEs are indicated (purple) in the insert.

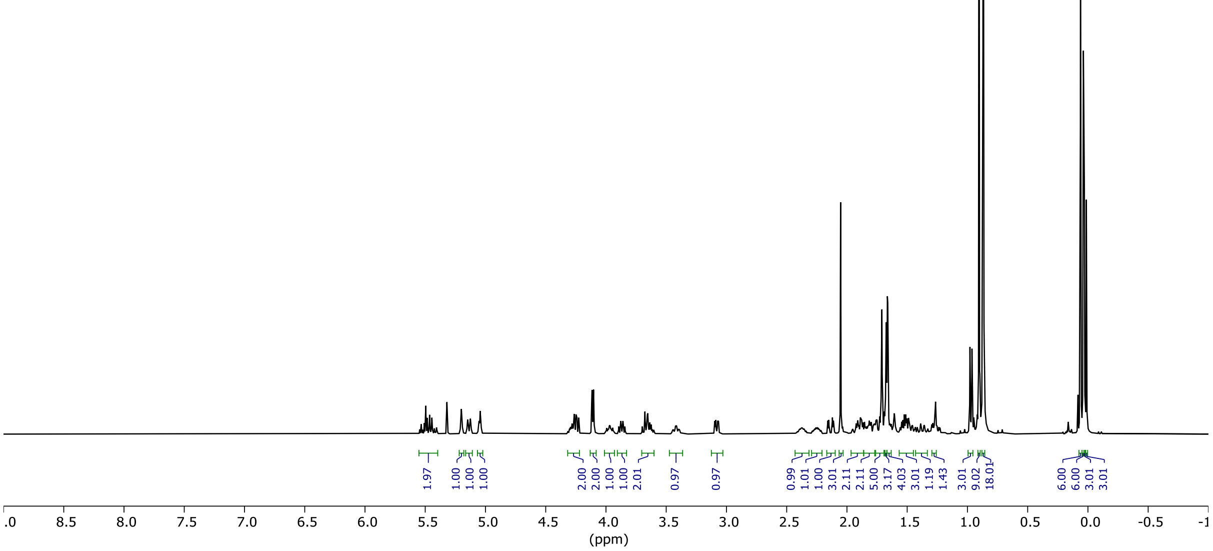

5.51 5.50 5.48 5.47 5.46 5.44 5.32 CD2Cl2 5.21 5.20 5.20 5.20 5.19 5.15 5.15 5.14 5.13 5.13 5.12 5.05 5.04 4.26 4.25 4.24 4.23 4.12 4.10 3.88 3.87 3.86 3.86 3.68 3.66 3.65 3.65 3.64 3.10 3.09 3.07 3.07 2.16 2.15 2.12 2.11 2.05 1.91 1.89 1.88 1.87 1.85 1.81 1.80 1.76 1.75 1.73 1.72 1.71 1.71 1.67 1.67 1.66 1.66 1.65 1.64 1.55 1.54 1.52 1.51 1.50 1.50 1.49 1.49 1.48 1.29 1.27 1.26 0.98 0.96 0.90 0.87 0.87 0.87 0.06 0.04 0.03 0.01
1H (CD2Cl2, 400.12 MHz)
1.97 1.00 1.00 1.00 2.00 2.00 1.00 1.00 2.01 0.97 0.97 0.99 1.01 1.00 3.01 2.11 2.11 5.00 3.17 4.03 3.01 1.19 1.43 3.01 9.02 18.01 6.00 6.00 3.01 3.01
(ppm)

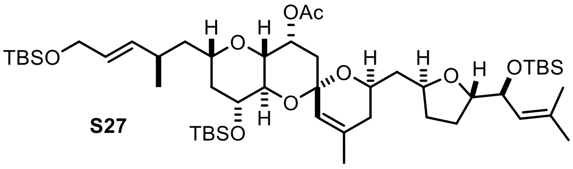

TBSO
OAc
TBSO
OTBS

S27

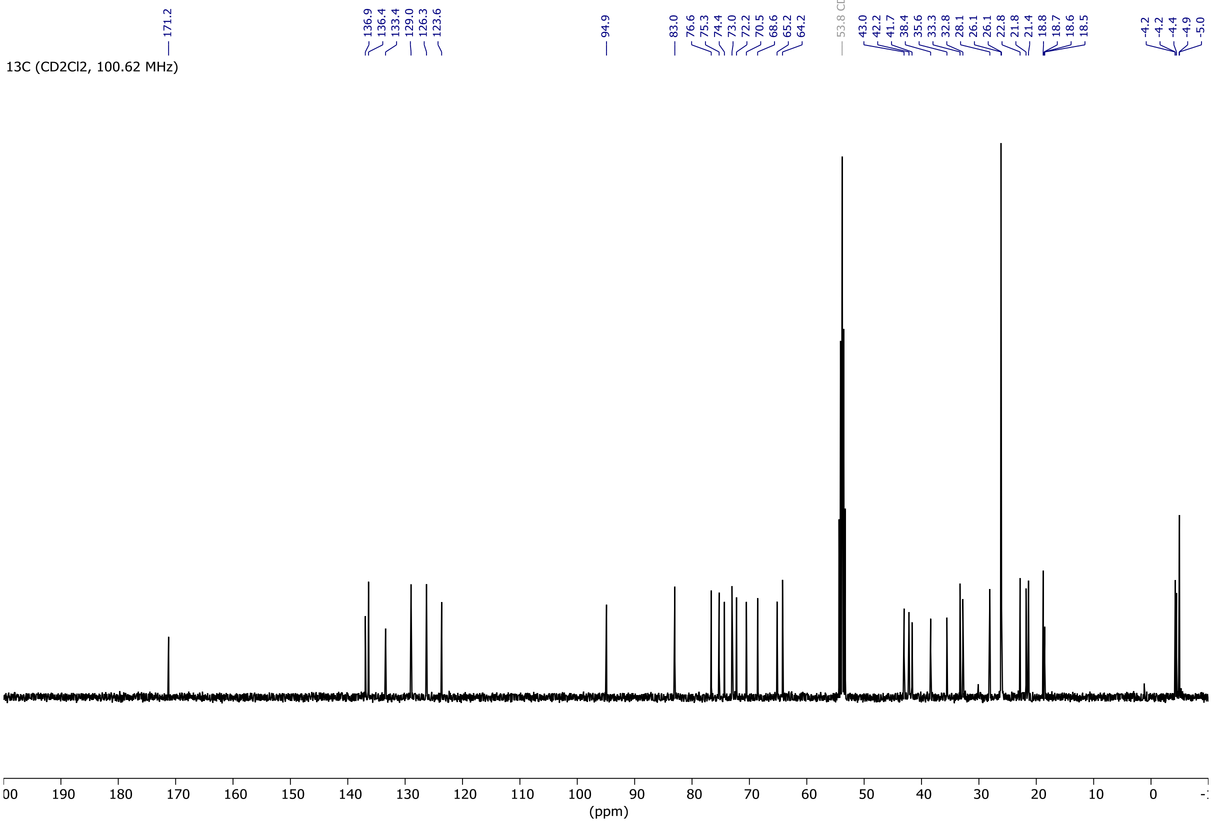

171.2 136.9 136.4 133.4 129.0 126.3 123.6 94.9 83.0 76.6 75.3 74.4 73.0 72.2 70.5 68.6 65.2 64.2 53.8 CD2Cl2 43.0 42.2 41.7 38.4 35.6 33.3 32.8 28.1 26.1 26.1 22.8 21.8 21.4 18.8 18.7 18.6 18.5 -4.2 -4.2 -4.4 -4.9 -5.0
13C (CD2Cl2, 100.62 MHz)
(ppm)

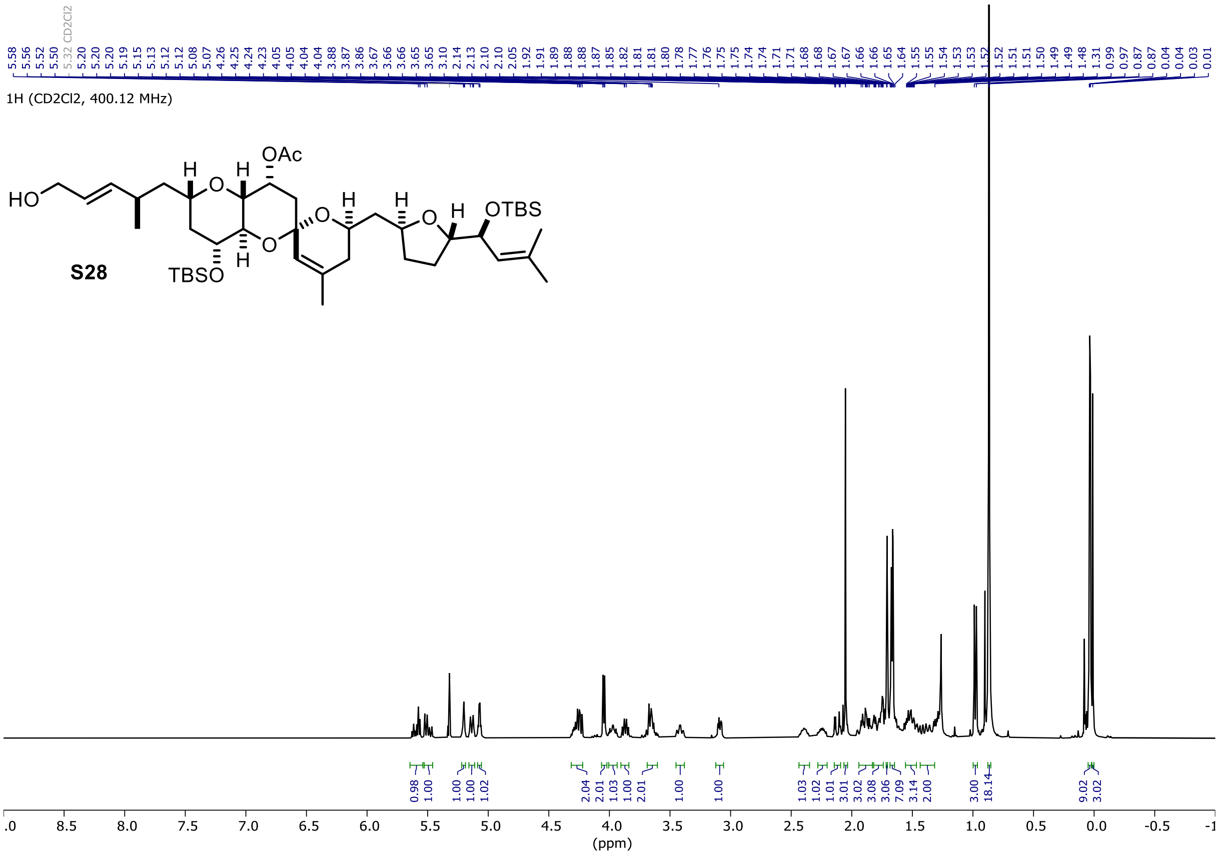
5.58 5.56 5.52 5.50 5.32 CD2Cl2 5.20 5.20 5.20 5.19 5.15 5.13 5.12 5.12 5.08 5.07 4.26 4.25 4.24 4.23 4.05 4.05 4.04 4.04 3.88 3.87 3.86 3.67 3.66 3.66 3.65 3.65 3.10 2.14 2.13 2.10 2.10 2.05 1.92 1.91 1.89 1.88 1.88 1.87 1.85 1.82 1.81 1.81 1.80 1.78 1.77 1.76 1.75 1.75 1.74 1.74 1.71 1.71 1.68 1.67 1.67 1.66 1.66 1.65 1.64 1.55 1.55 1.54 1.53 1.53 1.52 1.52 1.51 1.51 1.50 1.49 1.48 1.31 0.99 0.97 0.87 0.87 0.04 0.04 0.03 0.01
1H (CD2Cl2, 400.12 MHz)
HO
H
O
H
OAc
O
H
H
O
H
OTBS
S28
TBSO
H
O
0.98 1.00 1.00 1.00 1.02 2.04 2.01 1.03 1.00 2.01 1.00 1.00 1.03 1.02 1.01 3.01 3.02 3.08 3.06 7.09 3.14 2.00 3.00 18.14 9.02 3.02
8.5 8.0 7.5 7.0 6.5 6.0 5.5 5.0 4.5 4.0 3.5 3.0 2.5 2.0 1.5 1.0 0.5 0.0 -0.5
(ppm)

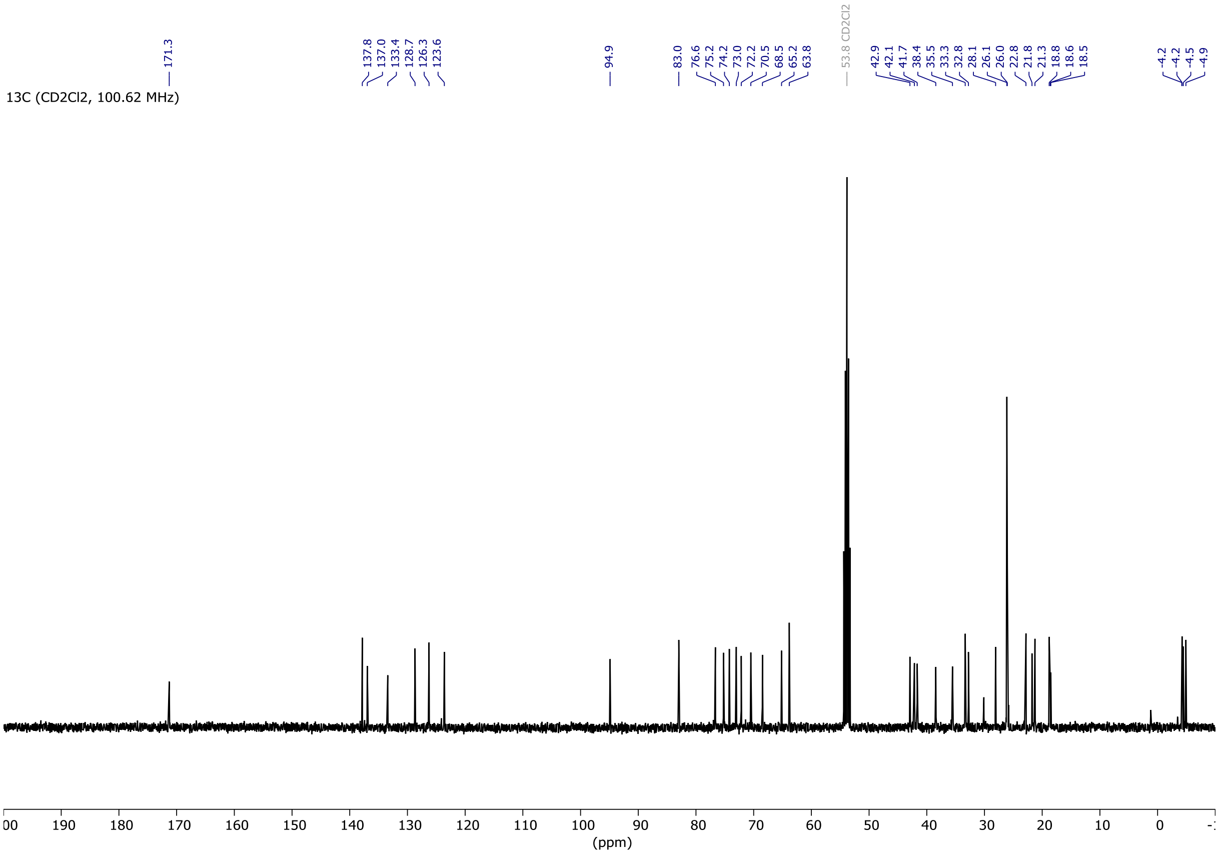
171.3 137.8 137.0 133.4 128.7 126.3 123.6 94.9 83.0 76.6 75.2 74.2 73.0 72.2 70.5 68.5 65.2 63.8 53.8 CD2Cl2 42.9 42.1 41.7 38.4 35.5 33.3 32.8 28.1 26.1 26.0 22.8 21.8 21.3 18.8 18.5 -4.2 -4.2 -4.5 -4.9
13C (CD2Cl2, 100.62 MHz)
190 180 170 160 150 140 130 120 110 100 90 80 70 60 50 40 30 20 10 0
(ppm)

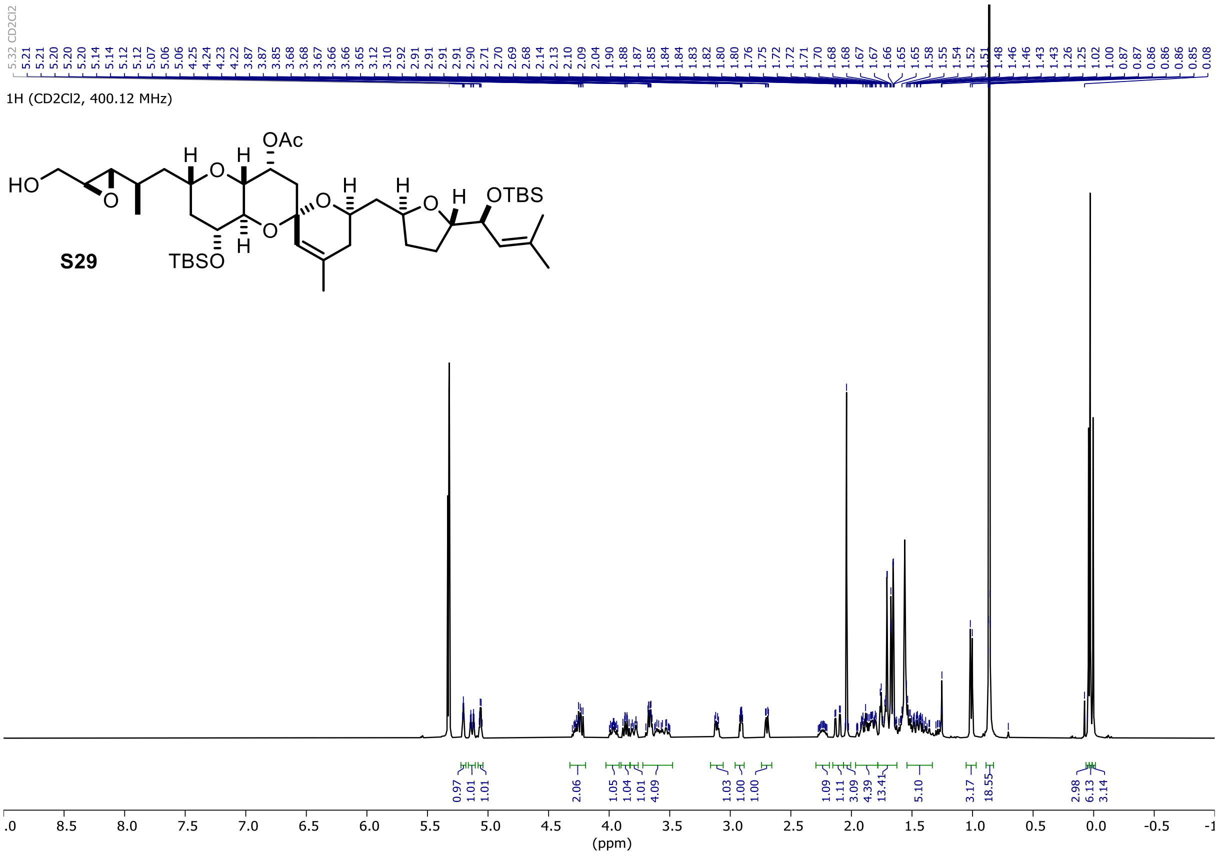
1H (CD2Cl2, 400.12 MHz)
S29
(ppm)

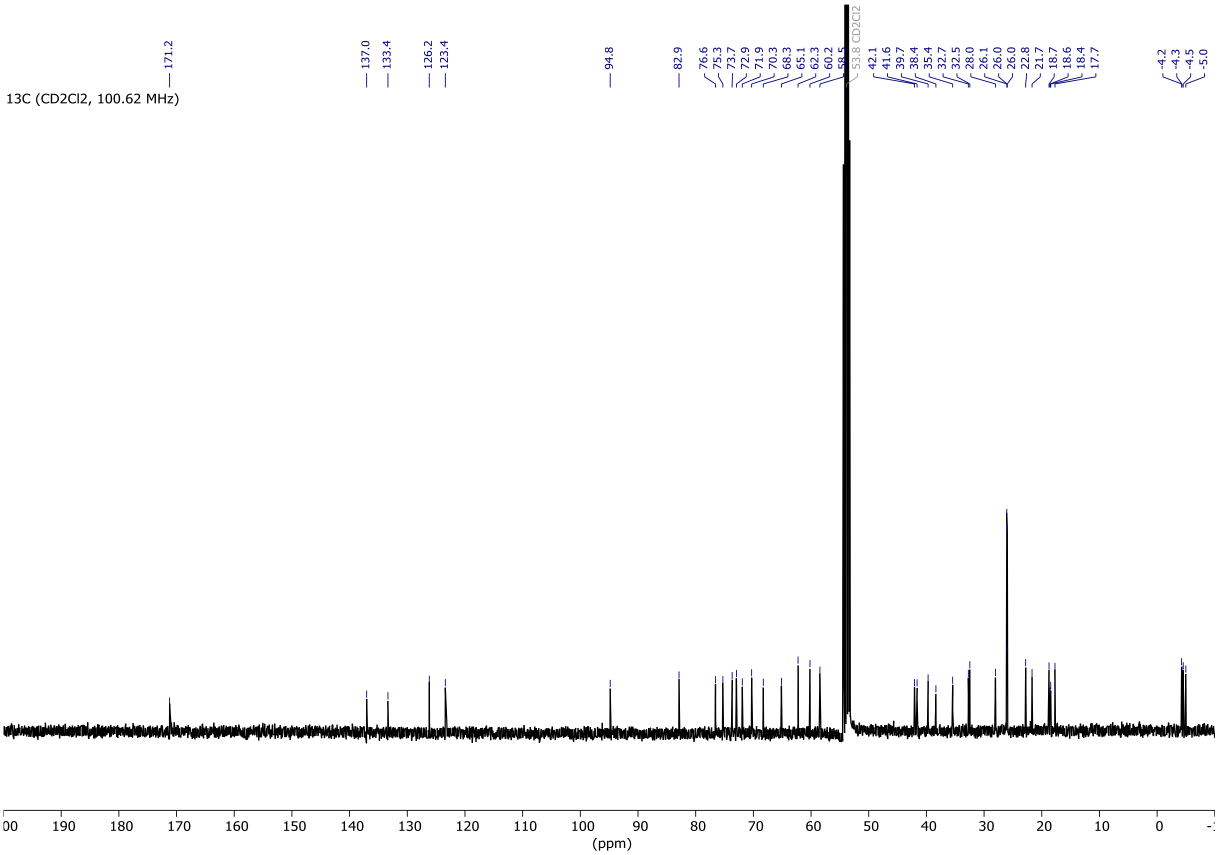
13C (CD2Cl2, 100.62 MHz)
(ppm)

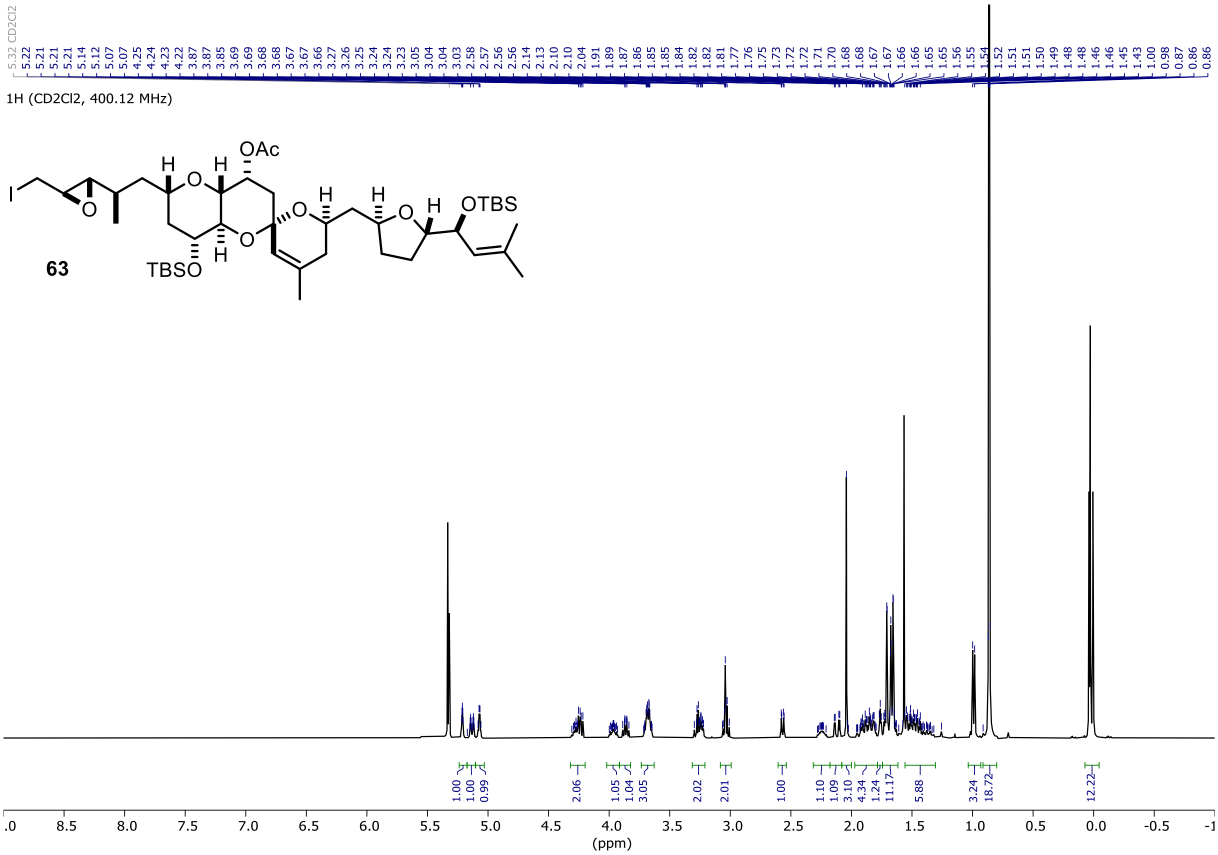
1H (CD2Cl2, 400.12 MHz)
OAc
OTBS
TBSO
(ppm)

63

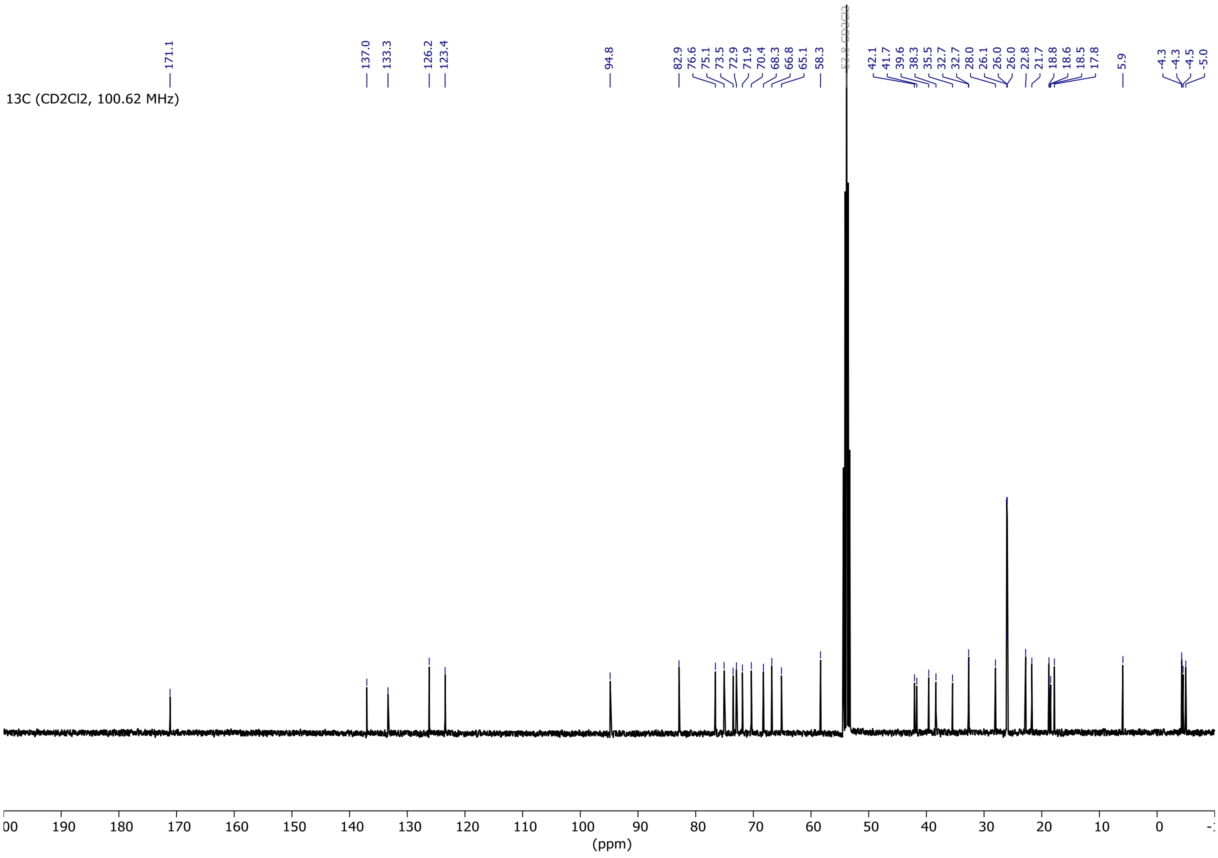
13C (CD2Cl2, 100.62 MHz)
(ppm)

1H (CD2Cl2, 400.12 MHz)

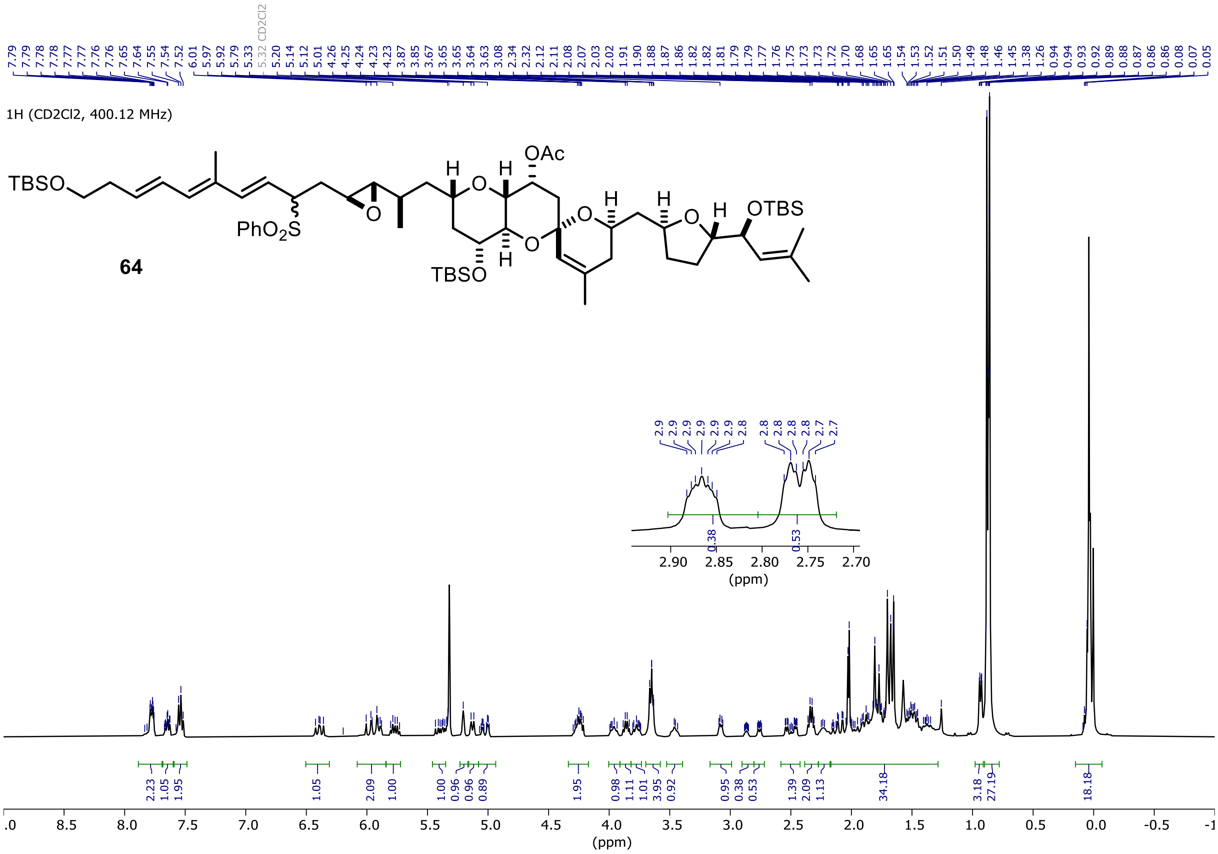

64

13C (CD2Cl2, 100.62 MHz)

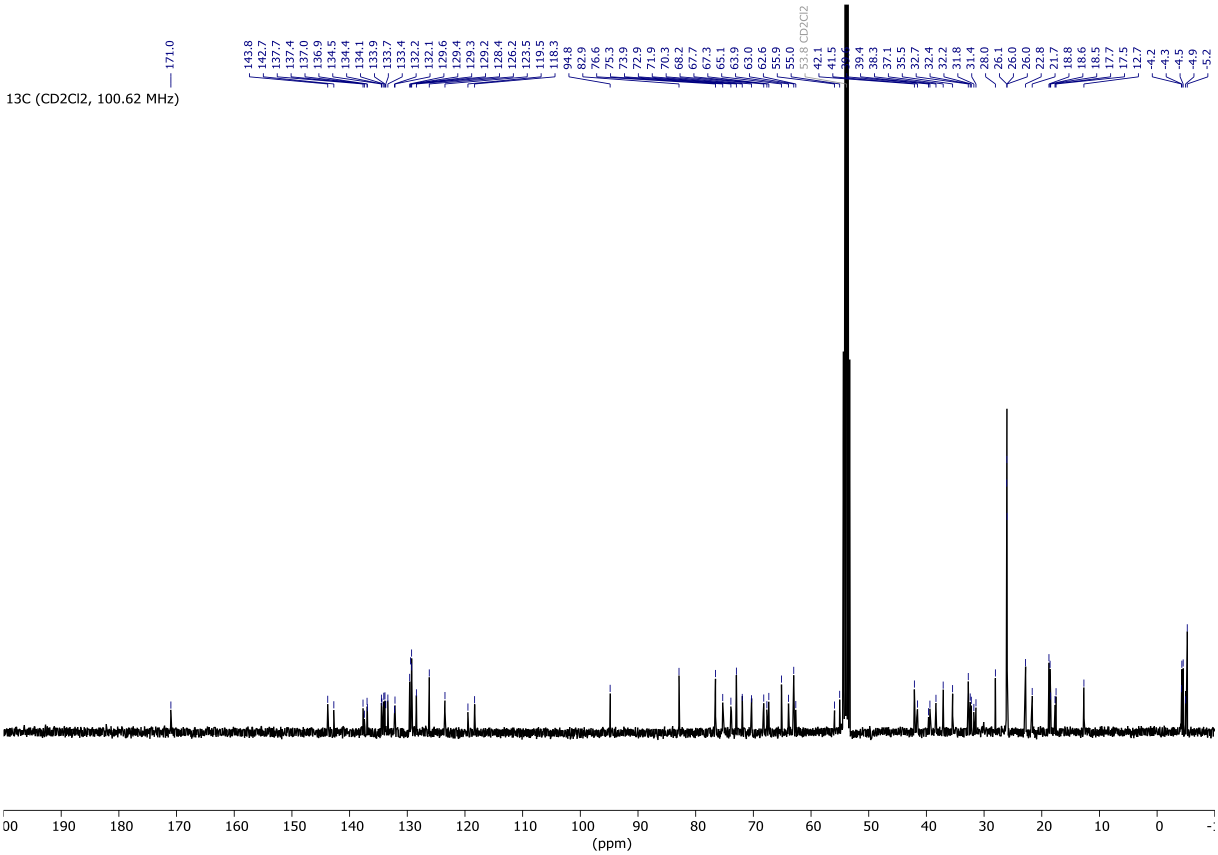

1H (CD2Cl2, 400.12 MHz)

S30

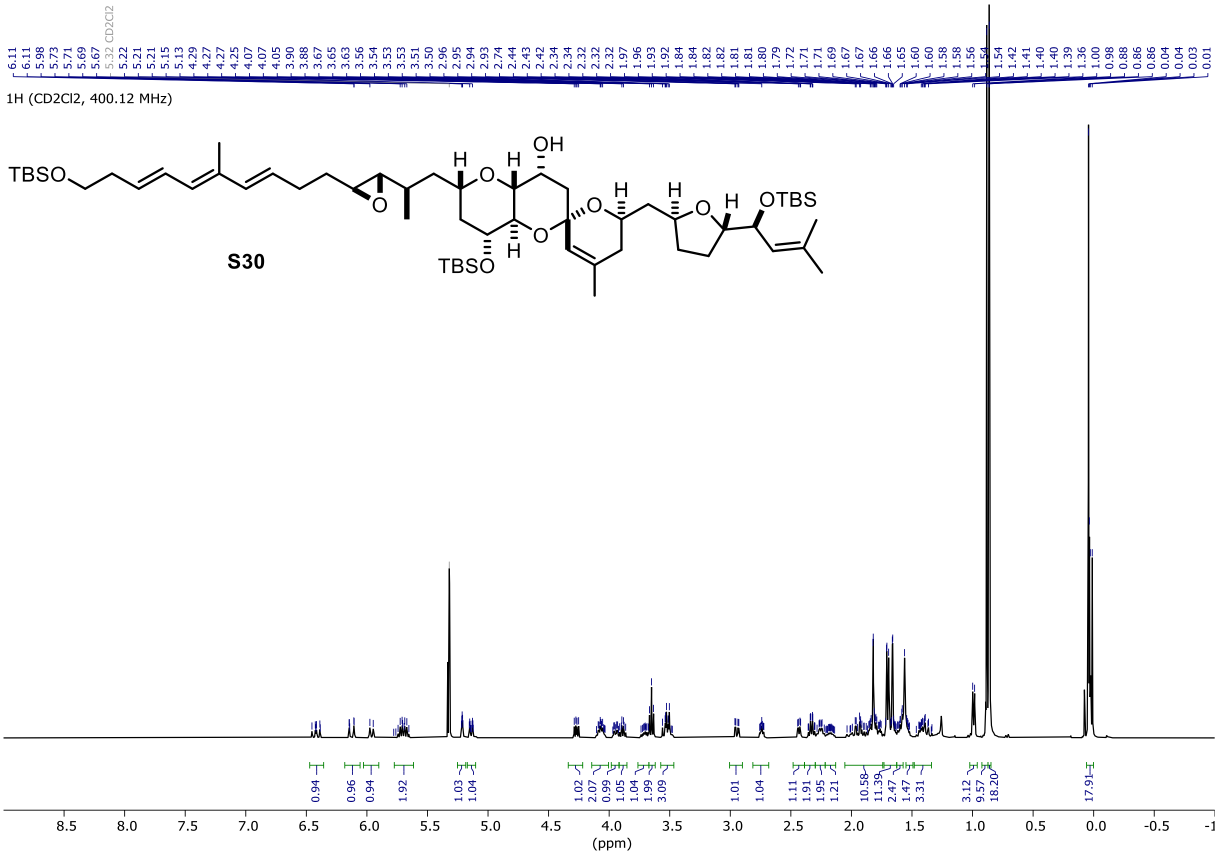

13C (CD2Cl2, 100.62 MHz)

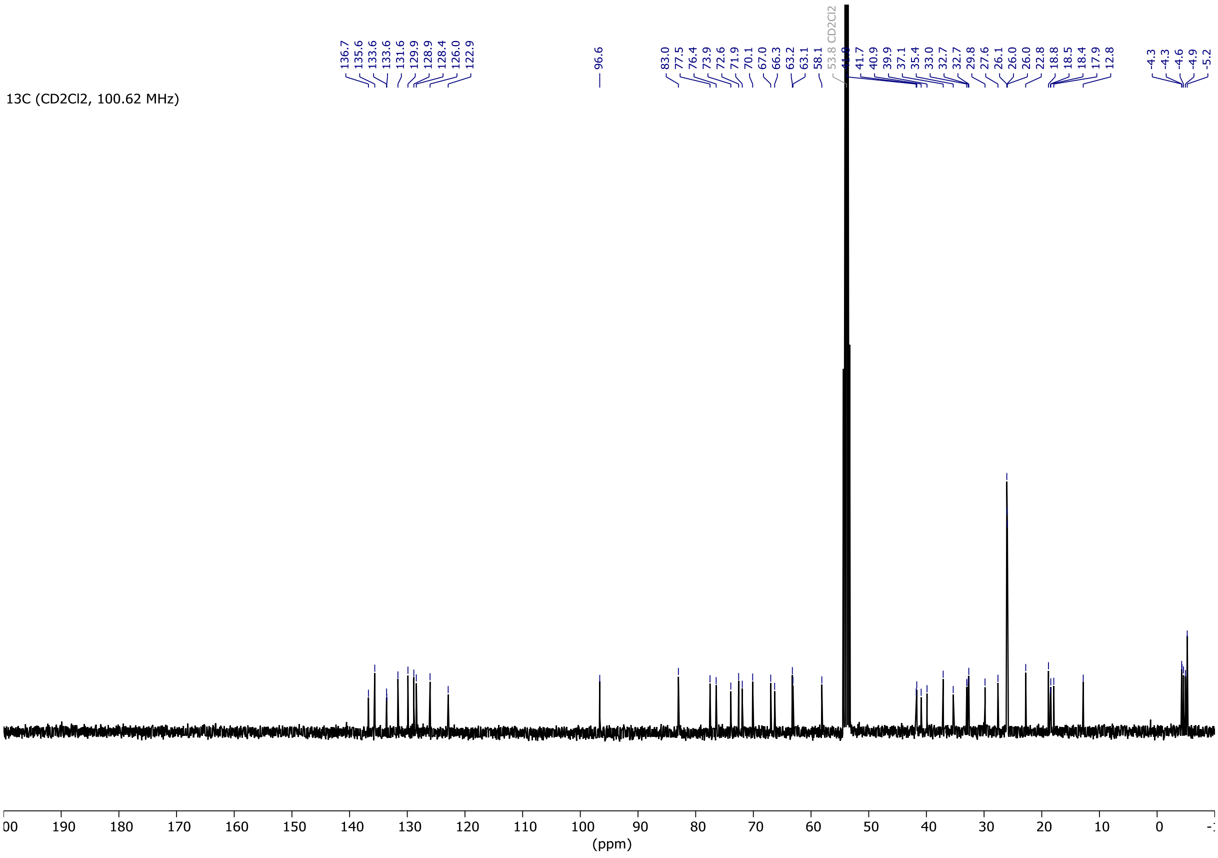

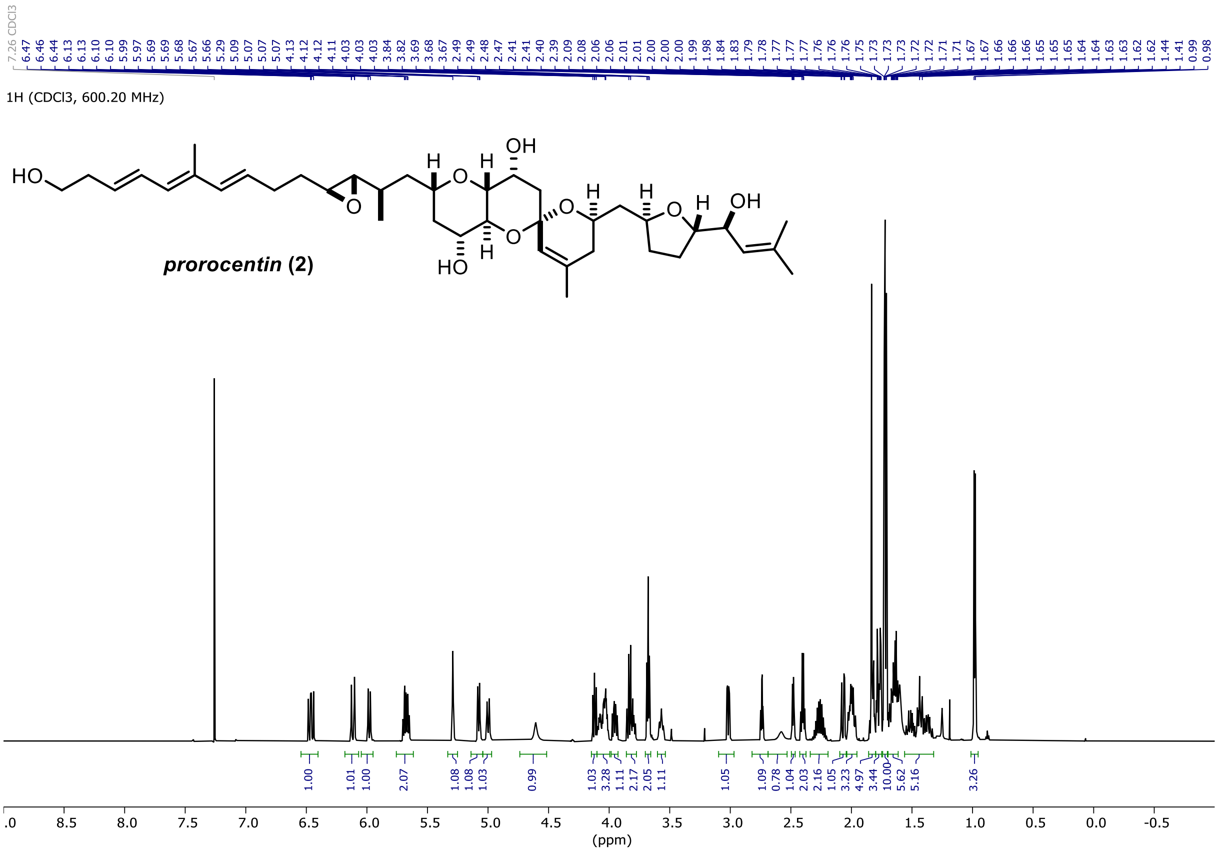
1H (CDCl3, 600.20 MHz)
prorocentin (2)
(ppm)

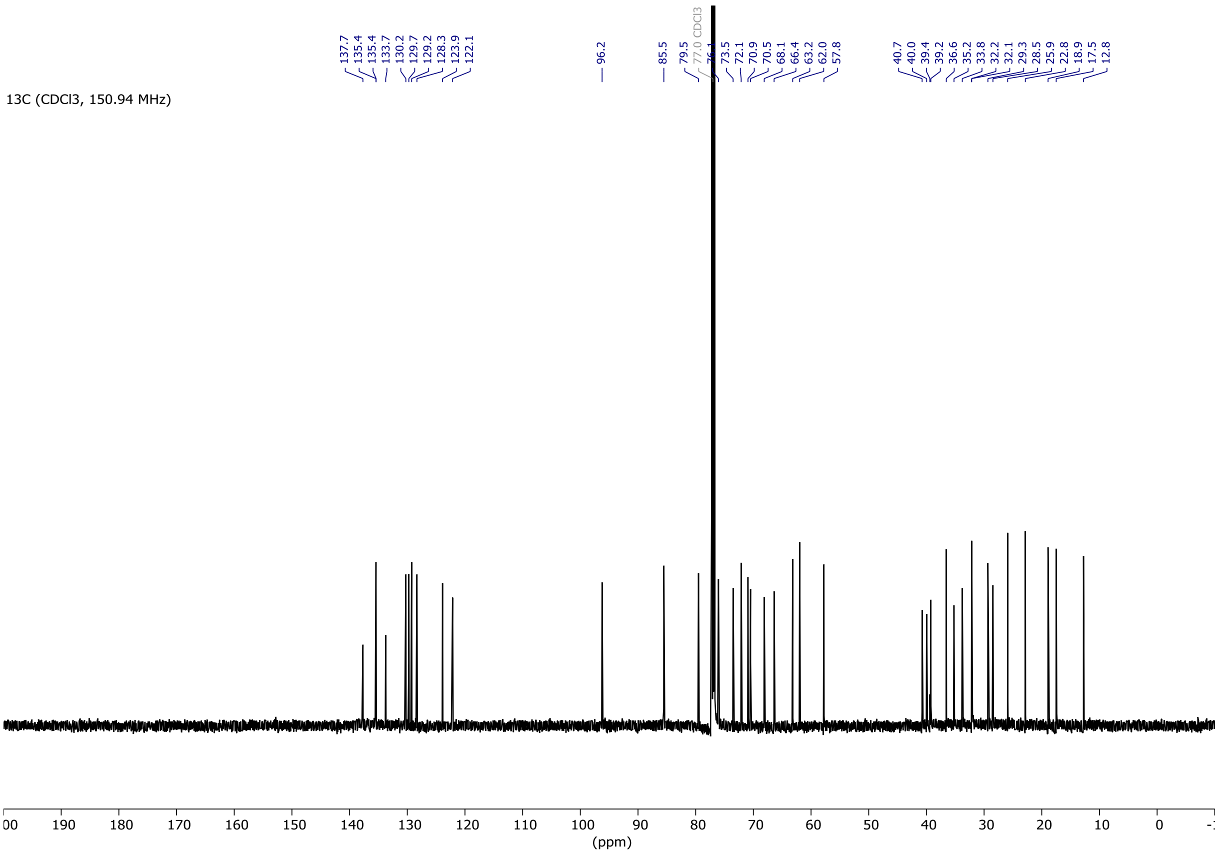
13C (CDCl3, 150.94 MHz)
(ppm)

REFERENCES

1. Lu, C.-K.; Chou, H.-N.; Lee, C.-K.; Lee, T.-H., Prorocentin, a New Polyketide from the Marine Dinoflagellate Prorocentrum lima. *Org. Lett.* **2005,** *7* (18), 3893-3896.
2. Grimblat, N.; Zanardi, M. M.; Sarotti, A. M., Beyond DP4: an Improved Probability for the Stereochemical Assignment of Isomeric Compounds using Quantum Chemical Calculations of NMR Shifts. *J. Org. Chem.* **2015,** *80* (24), 12526-12534.
3. Grimblat, N.; Sarotti, A. M., Computational Chemistry to the Rescue: Modern Toolboxes for the Assignment of Complex Molecules by GIAO NMR Calculations. *Chem. Eur. J.* **2016,** *22* (35), 12246-12261.
4. Marcarino, M. O.; Cicetti, S.; Zanardi, M. M.; Sarotti, A. M., A critical review on the use of DP4+ in the structural elucidation of natural products: the good, the bad and the ugly. A practical guide. *Nat. Prod. Rep.* **2022,** *39* (1), 58-76.
5. Note that in this type of analysis, the probabilities of isomers **1**-**6** sum up to 100%. The most likely candidate gets a very high score, whereas the other compounds get scores close to 0%. For an instructive case, see the Supporting Information of the following publication: Liu, Y.-F.; Zhang, Y.-H.; Shao, C.-L.; Cao, F.; Wang, C.-Y., Microketides A and B, Polyketides from a Gorgonian-Derived Microsphaeropsis sp. Fungus. *J. Nat. Prod*. **2020**, *83* (4), 1300-1304.
6. Fulmer, G. R.; Miller, A. J. M.; Sherden, N. H.; Gottlieb, H. E.; Nudelman, A.; Stoltz, B. M.; Bercaw, J. E.; Goldberg, K. I., NMR Chemical Shifts of Trace Impurities: Common Laboratory Solvents, Organics, and Gases in Deuterated Solvents Relevant to the Organometallic Chemist. *Organometallics* **2010,** *29* (9), 2176-2179.
7. Jensen, J. T. Studies towards the total synthesis of latrunculin A and latrunculin B. Doctoral thesis, Universität Dortmund, 2005.
8. Brasseur, D.; Marek, I.; Normant, J.-F., Diastereoselective synthesis of heterosubstituted organogembismetallic reagents. Application to a new propargylmetalation reaction of vinyl metals. *Tetrahedron* **1996,** *52* (21), 7235-7250.
9. Hoye, T. R.; Jeffrey, C. S.; Shao, F., Mosher ester analysis for the determination of absolute configuration of stereogenic (chiral) carbinol carbons. *Nature Protocols* **2007,** *2* (10), 2451-2458.
10. Inoue, M.; Wang, J.; Wang, G.-X.; Ogasawara, Y.; Hirama, M., Divergent synthesis of the tetracyclic ethers of 6-X-7-6 ring systems. *Tetrahedron* **2003,** *59* (30), 5645-5659.
11. Steib, P.; Breit, B., Concise Total Synthesis of (−)-Vermiculine through a Rhodium-Catalyzed C_2-Symmetric Dimerization Strategy. *Chem. Eur. J.* **2019,** *25* (14), 3532-3535.
12. Barnett, D. S.; Schaus, S. E., Asymmetric Propargylation of Ketones Using Allenylboronates Catalyzed by Chiral Biphenols. *Org. Lett.* **2011,** *13* (15), 4020-4023.
13. Gu, X.; Zhao, J.; Chen, L.; Li, Y.; Yu, B.; Tian, X.; Min, Z.; Xu, S.; Gu, H.; Sun, J.; Lu, X.; Chang, M.; Wang, X.; Zhao, L.; Ye, S.; Yang, H.; Tian, Y.; Gao, F.; Gai, Y.; Jia, G.; Wu, J.; Wang, Y.; Zhang, J.; Zhang, X.; Liu, W.; Gu, X.; Luo, X.; Dong, H.; Wang, H.; Schenkel, B.; Venturoni, F.; Filipponi, P.; Guelat, B.; Allmendinger, T.; Wietfeld, B.; Hoehn, P.; Kovacic, N.; Hermann, L.; Schlama, T.; Ruch, T.; Derrien, N.; Piechon, P.; Kleinbeck, F., Application of Transition-Metal Catalysis, Biocatalysis, and Flow Chemistry as State-of-the-Art Technologies in the Synthesis of LCZ696. *J. Org. Chem.* **2020,** *85* (11), 6844-6853.
14. Inoue, M. I., Masafumi; Yamashita, Shuji; Hirama, Masahiro, a concise route to two distinct E-ring structures of ciguatoxins. *Heterocycles* **2007,** *72*, 327-338.
15. Wang, S.-Y.; Song, P.; Chan, L.-Y.; Loh, T.-P., Total Synthesis of Phytophthora Mating Hormone α1. *Org. Lett.* **2010,** *12* (22), 5166-5169.
16. Cannizzo, L. F.; Grubbs, R. H., In situ preparation of (μ-chloro)(μ-methylene)bis(cyclopentadienyl)(dimethylaluminum)titanium (Tebbe's reagent). *J. Org. Chem.* **1985,** *50* (13), 2386-2387.
